# Genetic Networks in Mouse Retinal Ganglion Cells

**DOI:** 10.3389/fgene.2016.00169

**Published:** 2016-09-28

**Authors:** Felix L. Struebing, Richard K. Lee, Robert W. Williams, Eldon E. Geisert

**Affiliations:** ^1^Department of Ophthalmology, Emory University School of MedicineAtlanta, GA, USA; ^2^Bascom Palmer Eye Institute, University of Miami Miller School of MedicineMiami, FL, USA; ^3^Department of Genetics, Genomics and Informatics, University of Tennessee Health Science CenterMemphis, TN, USA

**Keywords:** retinal ganglion cells, gene regulatory networks, transcription factors, recombinant inbred strain, subtypes

## Abstract

Retinal ganglion cells (RGCs) are the output neuron of the eye, transmitting visual information from the retina through the optic nerve to the brain. The importance of RGCs for vision is demonstrated in blinding diseases where RGCs are lost, such as in glaucoma or after optic nerve injury. In the present study, we hypothesize that normal RGC function is transcriptionally regulated. To test our hypothesis, we examine large retinal expression microarray datasets from recombinant inbred mouse strains in GeneNetwork and define transcriptional networks of RGCs and their subtypes. Two major and functionally distinct transcriptional networks centering around *Thy1* and *Tubb3* (Class III beta-tubulin) were identified. Each network is independently regulated and modulated by unique genomic loci. Meta-analysis of publically available data confirms that RGC subtypes are differentially susceptible to death, with alpha-RGCs and intrinsically photosensitive RGCs (ipRGCs) being less sensitive to cell death than other RGC subtypes in a mouse model of glaucoma.

## Introduction

The retinal ganglion cell (RGC) is the final output neuron of the retina, projecting through the optic nerve to the brain, where it targets a number of functionally distinct areas: for visual perception, RGC axons travel to the lateral geniculate nucleus (Chalupa and Günhan, 2004); for the regulation of circadian rhythms, they pass through the suprachiasmatic nucleus (Guido et al., 2010); for eye movements, a group of RGC axons terminates in the superior colliculus (Triplett et al., 2014); and for the pupillary light reflex, RCG axons terminate in the pretectal area (Young and Lund, 1998). Each of these areas receives input from distinct subtypes of RGCs with unique morphological and molecular signatures. At the present time, over 30 subtypes of RGCs (Baden et al., 2016) are estimated to exist. They all receive inputs from other types of retinal neurons (bipolar cells and amacrine cells), and most of them express similar groups of genes that may serve as general RGC markers (Raymond et al., 2008; Rodriguez et al., 2014). Identifying gene expression patterns in RGCs and their subtypes is currently an active area of research, as demonstrated by the discovery of new subtypes of ganglion cells based on gene expression (Macosko et al., 2015; Sanes and Masland, 2015).

The death of RGCs in glaucoma or after injury eventually leads to loss of vision (Templeton et al., 2009; Zode et al., 2011; Munguba et al., 2014; Nuschke et al., 2015). However, the susceptibility of RGC subtypes to death differs among the distinct subtypes. Some RGCs are resistant to injury, while others appear to be more sensitive to insult, indicating differential gene expression and response to injury among subtypes (Chang et al., 2013; Duan et al., 2015; Puyang et al., 2015). The present study focuses on transcriptional networks within RGCs of the mouse, using gene expression data measured across 55 strains of recombinant inbred BXD mice (King et al., 2015) as well as the bioinformatic tools from GeneNetwork (Williams and Mulligan, 2012). The analysis begins by examining genes correlated with two relatively general RGC markers, *Thy1* and *Tubb3*. Each of these markers forms a unique network of genes that appears to function independently across many of the RGC subtypes. These networks are functionally different to the point of having distinct transcription factor binding sites. Subtype-specific networks partially overlapping with the *Thy1*-network are also present. In a meta-analysis of previously published data from a microarray study of a mouse glaucoma model (Howell et al., 2011), we examine the differential effects of this disease state on transcriptional networks in RGC subtypes and confirm intrinsically photosensitive RGCs (ipRGCs) and alpha-RGCs as more resistant to cell death (Duan et al., 2015).

The systems genetics and bioinformatics approach used in the present study demonstrates how signatures of RGCs and their subtypes can be extracted from a complex neural tissue such as the retina.

## Results

### RGC markers segregate into two major correlation networks

The present study examines the correlation of gene expression in the retina across the BXD recombinant inbred strain set to define gene networks active in RGCs. The BXD strain set is derived from two parental strains, the C57BL/6J mouse and the DBA/2J mouse. Natural variation in gene expression across strains can be used to identify co-regulated genes with a similar expression pattern, allowing for the construction of genetic networks (Williams et al., 2001; Geisert et al., 2009; Templeton et al., 2013a; Keeley et al., 2014).

The data used in this study consist of whole retinal samples collected from 55 BXD strains. They can be found on www.genenetwork.org under the identifier “DoD Retina Normal Affy MoGene 2.0 ST (May15) RMA Gene Level”. Two features of this dataset enhance the quality of the analysis: The first is that the retina is a tissue that can be consistently isolated with minimal contamination by other tissues. The second is the quality of the RNA with an average RNA Integrity Score of 9.43 and a standard error of 0.037 across the 220 samples isolated for this dataset.

**Figure 1:**
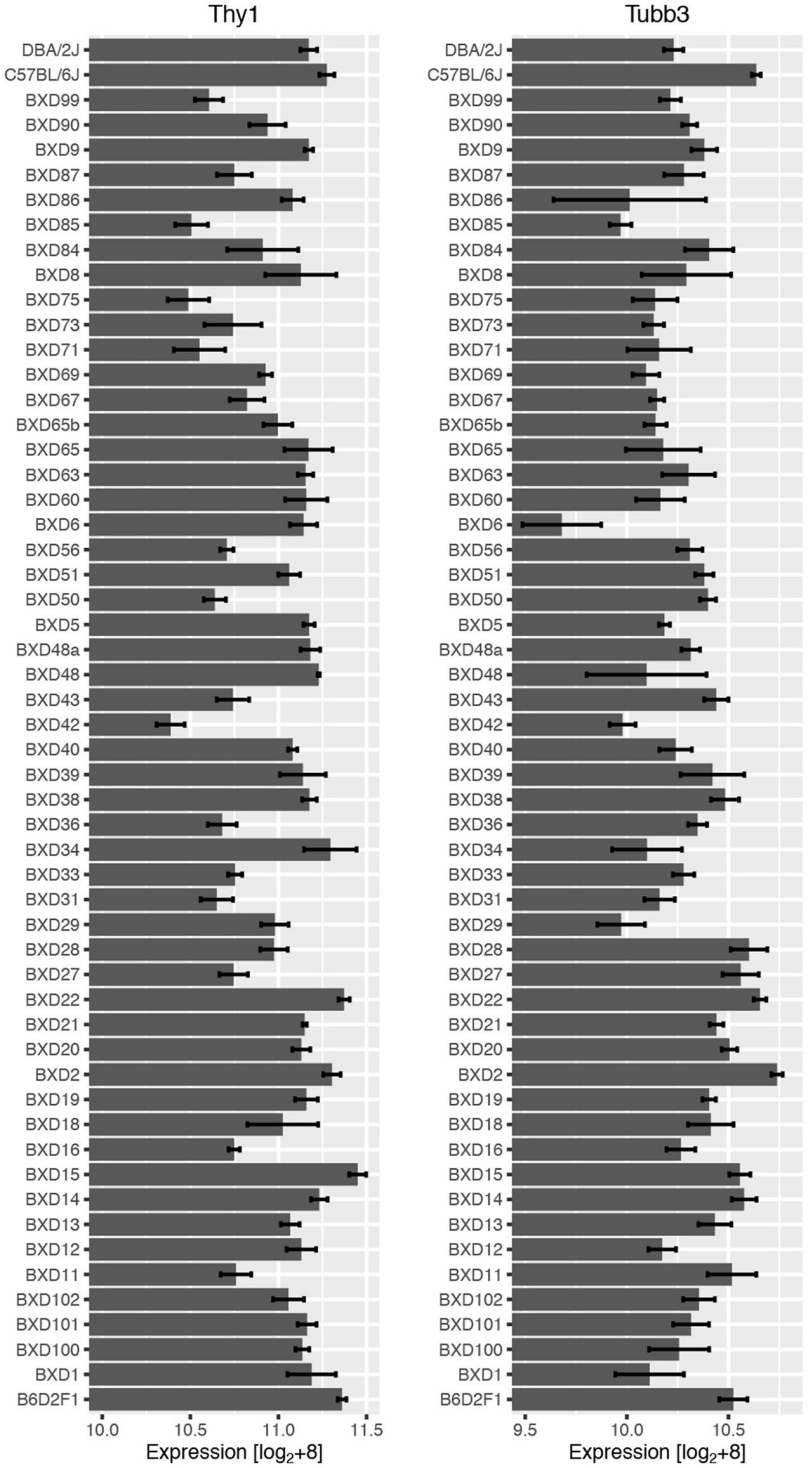
**RNA expression across the BXD RI strain set for *Thy1* (left) and *Tubb3* (right) with the means and standard errors**. There was an approximately 2-fold difference in mean expression levels for both genes. This data is given as raw expression values on a log_2_ scale +8.

The analysis began with two well-characterized markers for RGCs, *Thy1* and *Tubb3*, as they both exhibited substantial variability in mRNA expression levels across BXD strains (Figure 1). Expression of *Thy1* ranged from 10.39 in BXD42 to 11.45 in BXD15 (this data is presented on a log_2_ scale and the difference in expression is equivalent to an over 2-fold change). A similar variability in gene expression was observed for *Tubb3*, with expression levels ranging from 9.68 (BXD6) to 10.74 (BXD2). When correlations for *Thy1* and *Tubb3* were made across all microarray data, both produced a highly correlated group of genes. For *Thy1*, the top 100 correlates had an absolute *r*-value (Pearson) greater than 0.89 (Bonferroni-adjusted *p* < 1e^−12^) and the top 2000 correlates all had an absolute *r*-value greater than 0.77 (adj. *p* < 1e^−8^). If we examine the Pearson correlation values above 0.60, which corresponds to an adjusted *p*-value of 0.02, then *Thy1* has a total of 8596 correlates. This tightly correlated list of genes forms a potential network, and indicates that the genes in this network are co-regulated across the BXD strains (King et al., 2015). Within the list of the top 2000 *Thy1*-correlates, we found several other well-characterized RGC markers, including *Rbfox3* (producing NeuN, Neuronal Nuclei), *Pou4f1* (producing BRN3A), and *Pou4f2* (producing BRN3B) (Table 1). Interestingly, the *Thy1* correlate list did not contain other known RGC markers, including *Tubb3*.

**Table 1:** **List of all RGC marker genes used in this manuscript**.

**Symbol**	**RGC Subtype Labeled**	**References**
Thy1	pan-RGC	Barnstable and Drager, 1984; Raymond et al., 2008
Rbfox3 (NeuN)	pan-RGC	Wolf et al., 1996; Schlamp et al., 2013; Kim et al., 2014
Pou4f2 (Brn3b)	RGC (about 50–60% of total population)	Xiang et al., 1993; Erkman et al., 1996; Jain et al., 2012
	ipRGCs (71% of all Melanopsin-positive cells)	
Pou4f1 (Brn3a)	RGC (about 60–70% of total population)	Erkman et al., 1996; Jain et al., 2012
Tubb3 (class III beta-tubulin)	pan-RGC	Mellough et al., 2004
Rbpms (Retina binding protein with multiple splicing)	pan-RGC	Piri et al., 2006; Rodriguez et al., 2014
Nefl (Neurofilament light)	RGC (~85% of all RGCs)	Ruiz-Ederra et al., 2004
Chrna6	RGC	Mackey et al., 2012; Munguba et al., 2013
Slc17a6 (Vglut2)	RGC	Bai et al., 2001; Mimura et al., 2002; Wässle et al., 2006
Nrn1	RGCs	Picard et al., 2014; Sharma et al., 2015
Calb2	pan-RGC (87% of all RGCs) transient OFF α-RGCs (tOFF-αRGCs, Huberman et al., 2008)	Huberman et al., 2008; Mojumder et al., 2008; Haverkamp et al., 2009
Sncg (gamma-synuclein)	RGC	Buckingham et al., 2008
Opn4 (Melanopsin)	ipRGC	Semo et al., 2005
Jam2	J-RGC (5% of all RGCs)	Daniele et al., 2007; Kim et al., 2008
Spp1 (Osteopontin)	alpha-RGC	Ju et al., 2000; Sanes and Masland, 2015
Kcng4	alpha-RGC	Duan et al., 2014; Sanes and Masland, 2015
Cartpt	ooDSGC	Adams et al., 1999; Kay et al., 2011
Hoxd10	ON-DSGC	Dhande et al., 2013

For *Tubb3*, the top 100 genes had an absolute *r* value greater than 0.71 (adj. *p* < 1e^−6^), and the top 2000 genes showed values greater than 0.52 (adj. *p* = 0.12) and did not contain *Thy1, Pou4f1, Pou4f2*, or *Rbfox3.* This was also true for the 1387 correlates with Pearson *r* > 0.60 (equal to a significant Bonferroni-adjusted *p*-value of 0.02). However, other RGC markers were present in the *Tubb3* correlation list, including the newly described *Rbpms* (RNA binding protein with multiple splicing) as well as *Calb2* (Calbindin 2) and *Chrna6* (Cholinergic receptor nicotinic alpha 6).

These two networks are relatively independent, with minimal overlap. When examining the correlates of the *Thy1*-network relative to the *Tubb3*-network, only one gene is shared within their top 100 correlations (1%), 31 genes (1.55%) are present in the top 2000 correlations, and 51 genes (0.05%) are in common with the 9982 genes found in both the *Thy1* (8596 genes) and the *Tubb3* (1386 genes) correlation lists with a Pearson correlation above 0.6.

**Figure 2:**
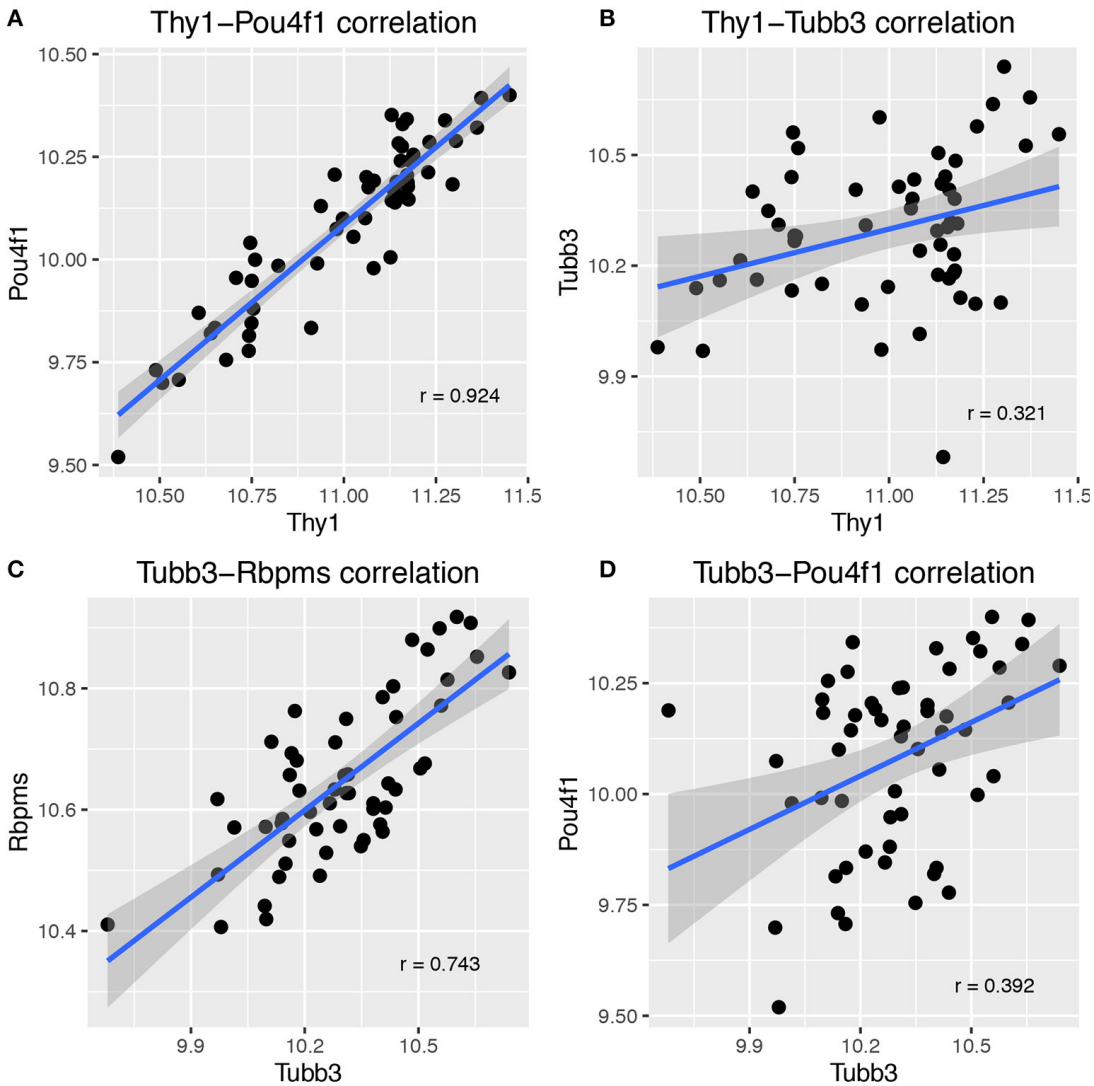
**Scatterplots illustrating the correlation between major RGC markers**. There was a tight correlation between *Thy1* and *Pou4f1* **(A)**, which are part of the same network. Correlation dropped greatly for *Thy1* and *Tubb3* **(B)**, which are not part of the same network. Similarly, *Tubb3* correlated well with Rbpms **(C)** but not with *Pou4f1* **(D)**. Pearson's correlation coefficient is given in each plot on the lower right half. Each dot represents one BXD RI strain, and the confidence interval for the smoothing function (dark gray areas surrounding the blue line) is 0.95.

The basis for the segregation of genes into two distinct networks is illustrated by plotting the correlations for combinations of genes from both networks. In Figure 2, the expression of *Thy1, Pou4f1, Tubb3*, and *Rbpms* across BXD strains is displayed in scatterplots. These plots demonstrate the tight correlation for *Thy1-Pou4f1* (Figure 2A) and *Tubb3-Rbpms* (Figure 2C). They further show the lack of correlation between *Thy1* and *Tubb3* (Figure 2B) as well as *Tubb3* and *Pou4f1* (Figure 2D). The presence of additional RGC markers in the correlation lists of *Thy1* and *Tubb3* together with the minimal overlap of genes indicated the presence of at least two RGC-specific transcriptional networks in mouse RGCs.

Since we selected *Thy1* and *Tubb3* to serve as primary RGC markers for this analysis based upon literature evidence, we wondered if an alternative unbiased approach would validate our findings. As an independent examination of these networks, we performed weighted gene correlation network analysis (WGCNA) on the whole microarray dataset (Langfelder and Horvath, 2008). This method relies on unsupervised clustering of co-expressed genes across all of the BXD RI strains into so-called modules or eigengenes and thus represents an unbiased approach to test the assumption that *Thy1*- and *Tubb3*- networks exist independently from each other. WGCNA created 18 modules of co-expressed genes. Module #1 contained 1741 of the top 2000 *Thy1* correlates, whereas only 131 genes from the top 2000 *Tubb3* correlates were assigned to this module. The majority of the *Tubb3* correlates belonged to modules #4 (*n* = 279), #5 (*n* = 886), and #6 (*n* = 327), none of which contained any of the *Thy1* correlates. This minor overlap in module affiliation between genes of both networks reaffirms our finding that the *Thy1*- and *Tubb3*-network function individually.

**Figure 3:**
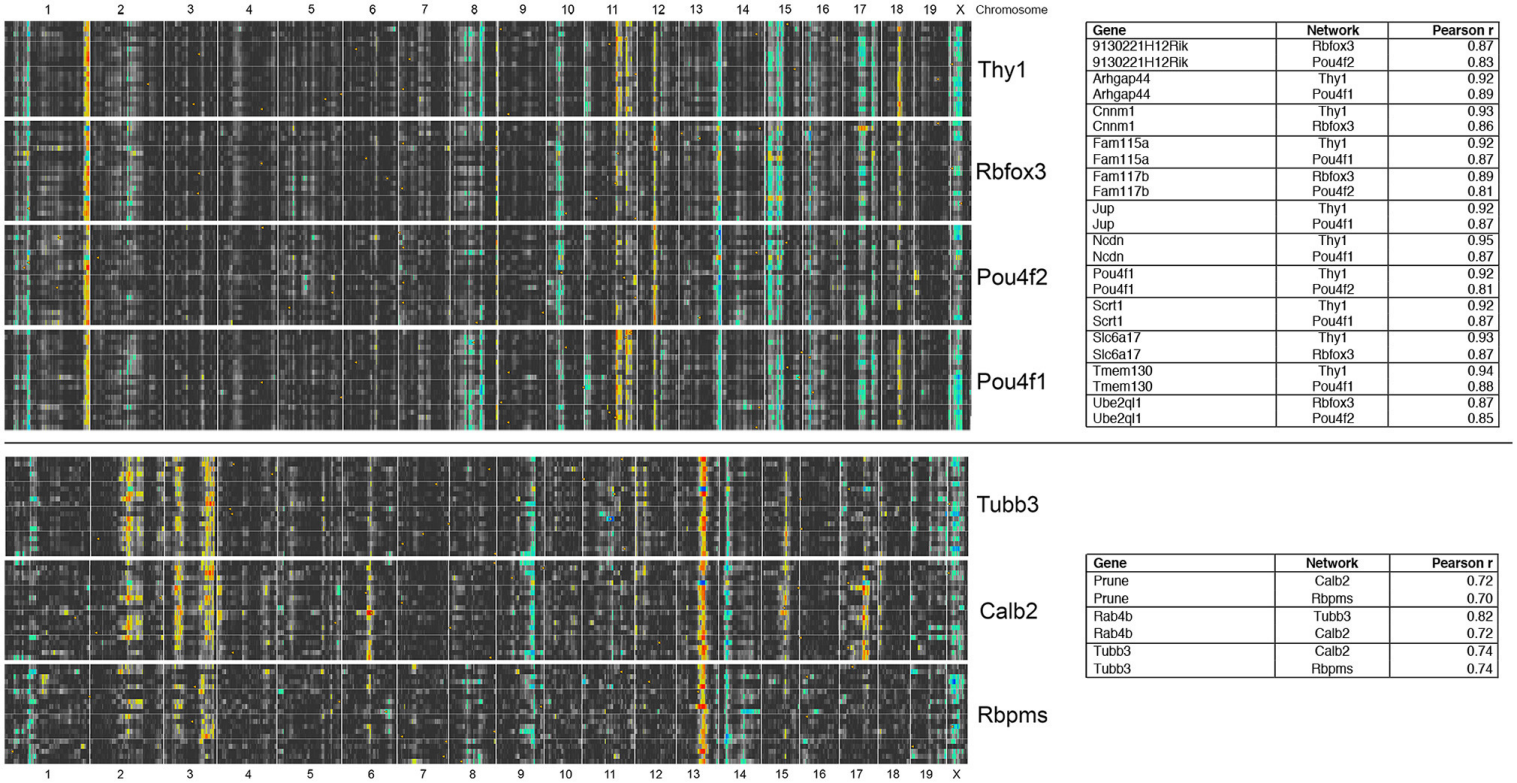
**RGC markers segregate into two major correlation networks**. For each of the general RGC markers on the right, the eQTL curve for the 20 highest correlated genes was plotted as a heat map. In these, the LRS/LOD score is given in pseudocolors: A yellow to red gradient identifies a transcript whose expression is higher in strains with a B haplotype at that locus (allele origin from C57BL/6J), whereas a green to blue gradient represents a transcript whose expression is higher in strains with the D haplotype (allele origin from DBA/2J). There are several strong and sharp trans-bands extending across *Thy1, Rbfox3, Pou4f2*, and *Pou4f1*, such as on distal Chromosome 1 or 13. There are also trans-bands extending across *Tubb3, Calb2*, and *Rbpms* on mid Chr. 13 and proximal Chr. 14. No overlap is present between the genes separated by the black line, indicating that RGC markers segregate into two major independently regulated gene networks. The panel on the right lists genes that are found in more than one of the top 20 correlations. For example, *Arhgap44* is present in the 20 highest correlates for both *Thy1* and *Pou4f1*.

To define the genomic location of upstream modulators for both the *Thy1*- and the *Tubb3*-network, we examined interval maps for the 7 RGC marker genes found in both correlation lists (*Thy1, Rbfox3, Pou4f2, Pou4f1, Tubb3, Calb2*, and *Rbpms*). When we investigated each marker's signature quantitative trait locus (QTL), we found that *Thy1, Rbfox3, Pou4f2*, and *Pou4f1*, had similar interval maps. The same was true for *Tubb3, Calb2*, and *Rbpms*. To further investigate this phenomenon, we plotted multiple interval maps as heat maps for each marker and its 20 highest correlated genes (Figure 3). In these heat maps, rows correspond to the QTL curve of a single correlated gene plotted across the entire genome. Two color gradients were used to characterize differential expression between strains: A yellow to red gradient identified a transcript whose expression was higher in strains with a B haplotype (allele origin from C57BL/6J), whereas a green to blue gradient represented a transcript whose expression was higher in strains with the D haplotype (allele origin from DBA/2J). The linkage significance (LRS or LOD-score) increased with color intensity. In other words: A deeply colored vertical line characterized a genomic locus that may contain a regulatory element responsible for the differential expression of these genes.

These bands are thought to identify a genomic locus modulating the expression of the genes in the network across the BXD RI strains. Likely candidates for modulating gene expression include transcription factors, micro RNAs or long noncoding RNAs (Geisert et al., 2009; Templeton et al., 2013b; Williams and Auwerx, 2015). Since the analysis is only correlational in nature, we cannot exclude the possibility that loci for individual networks do not have regulatory roles, as they could just be co-regulated with the other identified networks. When comparing heat maps for both networks, no overlap in patterns was observed. Thus, the RGC marker genes segregated into two independently regulated gene networks. From here on, we will refer to these networks as the *Thy1*-network and the *Tubb3*-network. This is an arbitrary nomenclature, mirroring the most prominent RGC marker for each of both networks. For the *Thy1*-network, the strongest modulatory signature was localized at the distal end of Chromosome 1. For members of the *Tubb3*- network, the most prominent genomic signature was on mid-distal Chromosome 13. Several candidate genes exist in those loci; a comprehensive analysis is attached as Supplemental Material (Supplemental Tables 1, 2).

**Figure 4:**
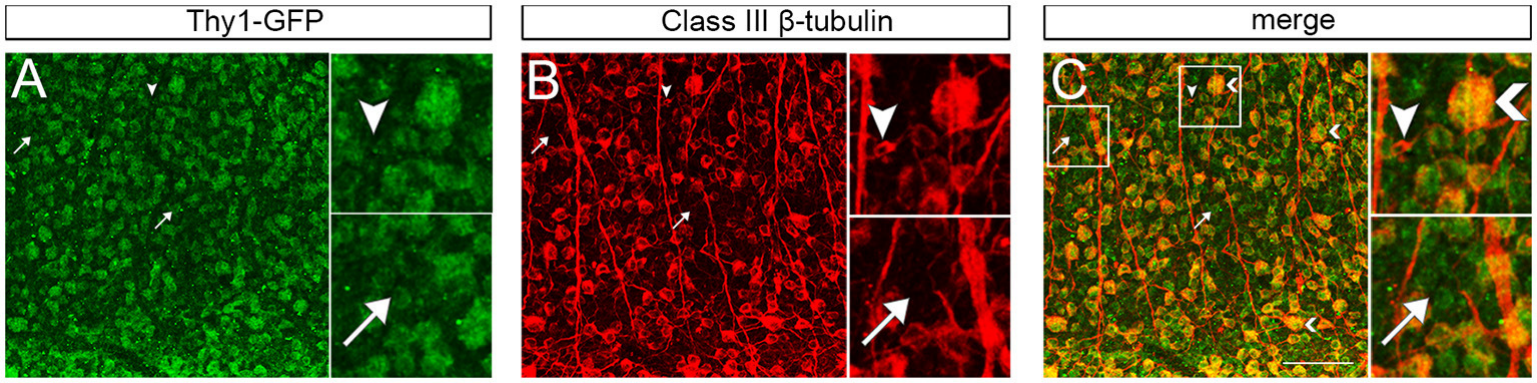
**Maximum intensity projections of confocal z-stacks taken from retinal whole-mounts showing the co-localization of THY1 (A) and Class III beta-tubulin (B) in the retinal ganglion cell layer from a C57BL/6 mouse**. Most cells were double-stained for both RGC markers **(C)**, but some cells only expressed Class III beta-tubulin (arrowhead) or THY1 (arrow). Furthermore, the staining intensity was different across cells, and some cells had large somata and were more intensely stained than others (large arrowhead in “merge”). Staining with the secondary antibodies only did not result in unspecific fluorescence (data not shown). Scale bar in C = 100 μm.

There are three potential scenarios that would allow two specific genetic networks to exist within a single cell population. The first is that the mRNA levels of each marker differ across the entire retina. The second explanation is that their mRNA expression levels differ in specific RGC subtypes, leading to unique expression of each protein within each RGC subtype. The third potential mechanism involves differences in the percentage of each of the RGC subtypes from strain to stain. To further examine this, we immunostained retinas with THY1 and Class III beta tubulin. In these retinal whole mounts, the more than 90% of labeled cells were double-labeled of RGCs were both THY1- and Class III beta tubulin- positive (Figure 4), indicating that the two markers are co-localized and expressed in the same cell. Despite their protein co-expression, their mRNA level may differ from cell to cell. When examining the double stained cells, the intensity of each stain varied from ganglion cell to ganglion cell. Some cells had approximately equal labeling for THY1 and Class III beta tubulin, while in other cells THY1 staining was more intense than Class III beta tubulin. In a few cells, the labeling of Class III beta tubulin was more intense. It also appeared that some cells were labeled by only one of the two markers. Thus, even though both markers are co-expressed in many RGCs, their expression levels do not consistently correlate with each other, corroborating that their expression is independently regulated.

**Figure 5:**
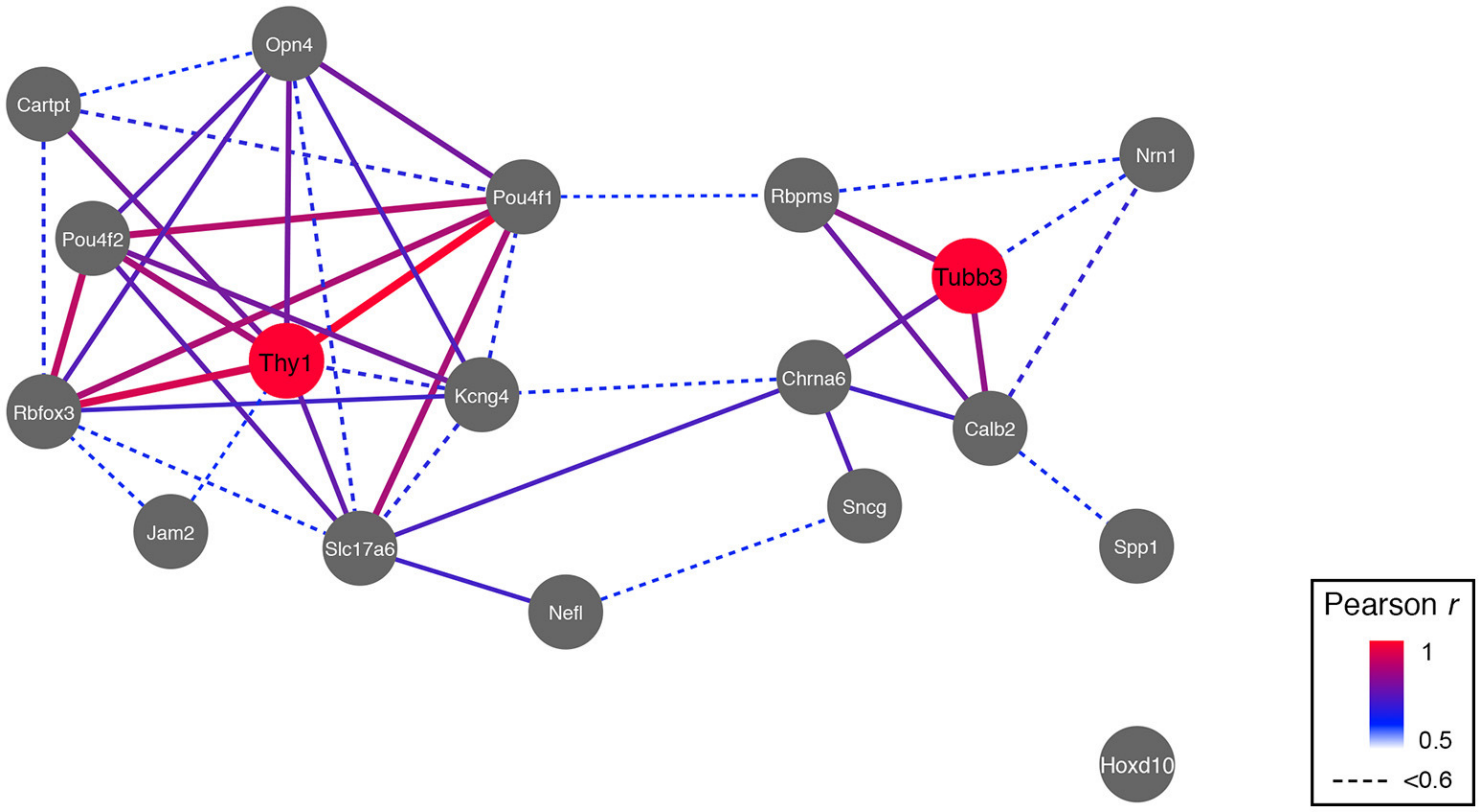
**RGC markers segregate into two networks, and two major hubs are formed around *Thy1* and *Tubb3***. The only connection between the *Thy1*- and the *Tubb3*-network is through two of their correlates, *Slc17a6* and *Chrna6*. Pearson's correlation coefficient was mapped to line color and thickness (high correlation = thicker and red bars).

Since multiple subtypes of RGCs are known to exist, one could predict that there are also multiple genetic networks within each of these subtypes. We compiled an extended list of RGC markers from the literature. This list consisted of 17 proteins and their respective genes, 6 of which are known to be relatively specific for a single RGC subtype (Table 1). Calculating the correlation in expression across the BXD RI strain set and displaying these relations in a network graph revealed that indeed two major network hubs formed around *Thy1* and *Tubb3* based on the highest correlations surrounding these two markers (Figure 5). The only significant connection between *Thy1* and *Tubb3* existed through 2 of their respective correlates, *Slc17a6* (VGLUT2), and *Chrna6*. Except for *Hoxd10* (a marker of ON-directionally selective RGCs), all the other RGC markers had a direct connection to either *Thy1* or *Tubb3*.

### Functional differences of RGC networks

Following the identification of two gene networks related to normal RGC function, we asked whether both networks had any functional differences.

In order to facilitate this analysis, we grouped genes with a similar heat map pattern belonging to the same network into one “synthetic trait” using principal component analysis. These “synthetic traits” provide for more robust functional network analysis than a single marker gene, and they can be used to generate correlation lists specific to a group of genes by reducing the dimensionality of the data (Yin et al., 2012; Vanderlinden et al., 2013; Graybeal et al., 2014).

The synthetic trait for the *Thy1*-network was constructed using *Thy1* and its direct correlates *Rbfox3, Pou4f1*, and *Pou4f2*, while the *Tubb3*-network was constructed using *Tubb3, Calb2*, and *Rbpms*. When we compared the correlations of these networks using WebGestalt-based gene ontology (GO) analysis (Wang et al., 2013), each of the two synthetic traits was enriched in genes with very different molecular and cellular functions. The *Thy1*-network was significantly enriched in genes involved in neuron development, synaptic transmission, cation transmembrane transporter activity and voltage-gated channel activity (adj. *p* < 0.0000001 for all), strongly indicating that this network was highly neuron-specific (Supplemental Figure S1). Some examples of the genes associated with neuronal development and synaptic transmission included: *Tnr, NeuroD2, L1cam, Syn2, Grn4*, and *Gabbr1* (see Supplemental Table 3 for the whole list). A close examination of the highest (*r* > 0.9) correlates also revealed that at least 6 genes (*Gsk3a, Srgap1, Arhgap44, Ncdn, L1cam*, and *Lrrc4b)* were functionally associated with neurite outgrowth.

The *Tubb3* synthetic network contained genes that were associated with completely different biological functions (Supplemental Figure S2). Several members of the tubulin family of proteins were at the top of the correlation list, such as *Tubg2, Tuba4a, Tubg1, Tubb5*, and *Tuba1b*. Gene Ontology analysis revealed enrichment in GO terms “guanyl nucleotide binding” and “protein polymerization” (both with adj. *p* < 0.001), including the genes *Rab11b, Bab8a, Tufm, Rabb1b, Arf6*, and *Rab4b*. The highest correlates of the *Tubb3* synthetic trait were two genes encoding proteins from the Rab family (*Rab15* and *Rab4b*).

### Distinct groups of transcription factor binding sites are associated with the two RGC networks

Since the *Thy1*- and the *Tubb3*-network were functionally enriched for distinct GO terms, we asked if this difference would be reflected in their transcription factor binding site (TFBS) distribution. We searched the promoter sequences for genes in the *Thy1*-network using the TRANSFAC FMatch algorithm (Matys et al., 2006) for over-represented TFBS in comparison to the *Tubb3*-network and vice versa (Tables 2a,b). Genes of the *Thy1*-network were ~4 times more enriched in TFBS for *p53* and *Dec1*, two master regulators of cell cycle progression (Qian et al., 2012). The *Thy1*-network was also more enriched in TFBS for PPAR gamma and estrogen receptor alpha as well as effectors SP1 and AP1. In contrast, the promoters of genes in the *Tubb3*-network were significantly enriched in TFBS of developmental origin, such as *Pax6, Six6*, proteins of the *Sox* and *Oct* family, as well as *Pou4f1*.

**Table 2a:** **Transcription factor binding site enrichment for genes of the *Thy1*-network**.

**Transcription Factor**	**Enrichment probability (fold) vs. Tubb3**	**Matched promoters *p*-value**
P53	3.6921	1.17E-04
TBP	4.7405	2.47E-03
PPARgamma:RXR-alpha	1.1771	1.07E-02
PPAR direct repeat	1.7507	1.34E-02
LXR, PXR, CAR, COU	1.9258	1.93E-02
DEC1	4.4442	2.11E-02
FOXJ1	4.4442	2.11E-02
AP-1	1.2523	3.47E-02
SP1	1.1402	4.62E-02
ER-alpha	2.4295	5.18E-02

**Table 2b:** **Transcription factor binding site enrichment for genes of the *Tubb3*-network**.

**Transcription Factor**	**Enrichment probability (fold) vs. Thy1**	**Matched promoters *p*-value**
Pax-6	1.3845	2.13E-05
OTX	1.8106	5.81E-05
SRY	1.428	3.26E-04
Oct1	1.6876	2.71E-03
FOXO1	1.1574	3.08E-03
Sox1	3.0377	3.10E-03
Nkx6-2	1.3845	3.16E-03
Tst-1	1.2976	3.55E-03
Oct4 (POU5F1)	1.8934	3.90E-03
Foxc1	1.0931	4.14E-03
Foxm1	1.0472	5.44E-03
SIX6 secondary motif	1.6129	5.65E-03
NF-AT	1.2657	6.66E-03
Pitx3	1.2304	7.34E-03
Brn-2	1.6214	9.59E-03
POU4F1	4.219	9.66E-03
c-Myc:Max	1.8081	1.14E-02
Bach1	10.1255	1.25E-02
Dlx2	1.4746	1.36E-02
POU2F1	1.1806	1.45E-02

### Genomic regulation of subtype-specific RGC markers

Since our analysis revealed two distinct molecular networks governing normal RGC function, we hypothesized that subtype-specific RGC markers would contain regulatory signatures from either one or both RGC networks.

**Figure 6:**
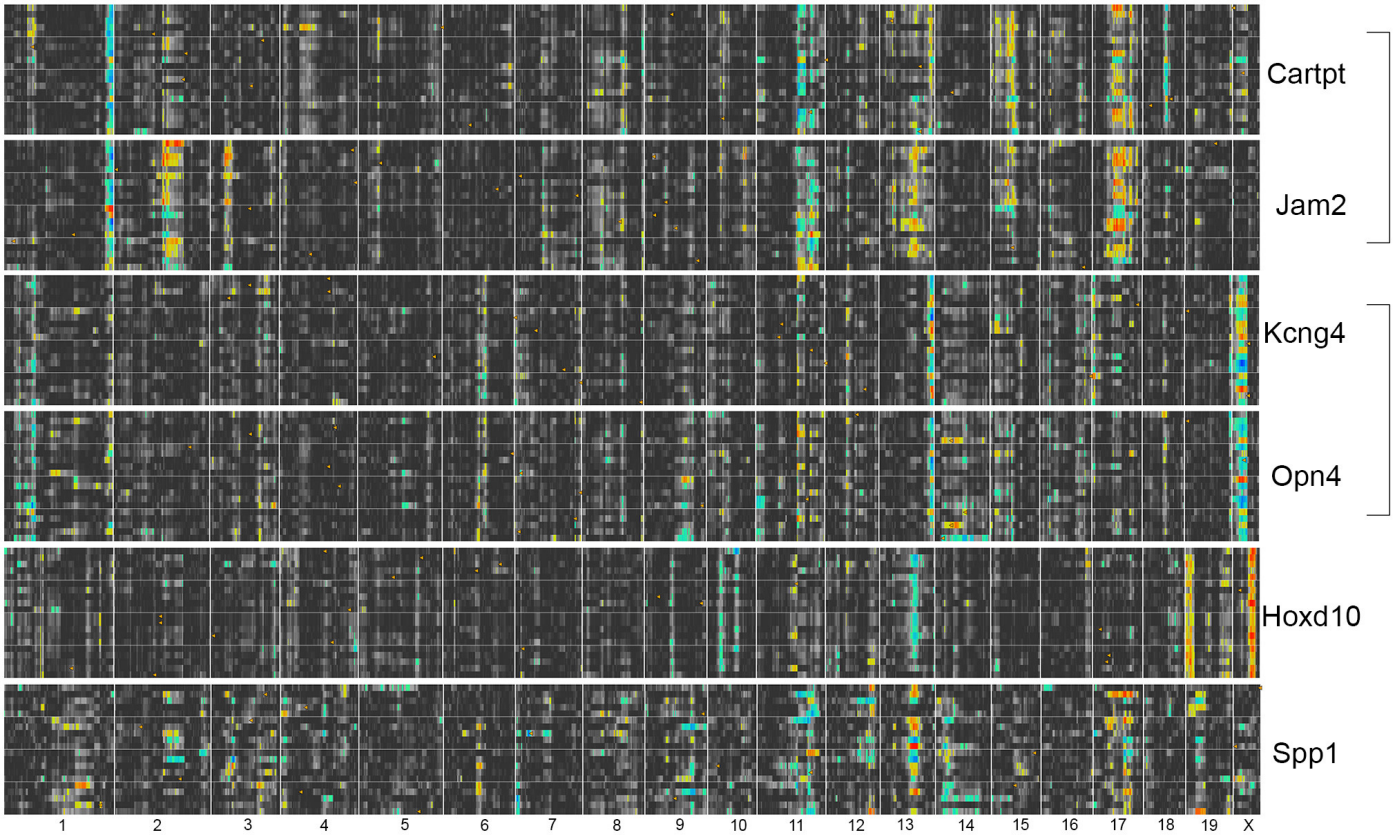
**Heat maps for RGC subtype-specific marker genes**. *Cartpt/Jam2* share the trans-band on distal Chromosome 1 with the *Thy1*-network, whereas *Kcng4/Opn4* share the *Thy1*-network trans-band from Chromosome 13. There is no obvious overlap of trans-bands with either network for *Hoxd10*. For *Spp1*, no obvious trans-bands can be appreciated, suggesting that this gene is not part of a transcriptional network in its normal state.

Thus, we generated heat maps of the highest correlates of each subtype-specific marker (*Cartpt2, Jam2, Kcng4, Opn4, Spp1*, and *Hoxd10*, see Figure 5). This comprehensive analysis revealed that two pairs of genes were regulated in a similar fashion: *Cartpt* and *Jam2* as well as *Kcng4* and *Opn4* showed very similar heat maps to each other. When we compared the heat maps for these 4 subtype-specific markers, we found that some of their bands were at identical loci as bands observed in the *Thy1*-network (Figure 6). While *Cartpt/Jam2* had the distinct band from distal Chromosome 1, *Kcng4*/*Opn4* shared a band on distal Chromosome 13. It was also noticed that the allelic distribution of correlates for *Cartpt/Jam2* seen as a blue line on distal Chromosome 1 was diametrical to the original *Thy1*-correlates, where the line was mostly yellow-red (see Figure 3). This simply implies that the top 20 correlates of *Cartpt* and *Jam2* are in fact correlated inversely to *Cartpt* or *Jam2* itself, whereas the top 20 correlates of *Thy1* are positively correlated to *Thy1* itself.

*Hoxd10* had very distinct trans-QTL bands that were not found elsewhere, and its correlates also did not contain any RGC-enriched genes. Since no connection of *Hoxd10* to either the *Thy1*- or the *Tubb3*-network (see Figure 3) was observed, the lack of overlap with RGC-enriched genes casts doubt on the specificity of this marker for the identification of RGC subtypes based on gene expression. The eQTL pattern of *Spp1* was the most complex of all markers, as it lacked any clearly detectable *trans*-band.

### Overlap of correlates of subtype-specific markers with the *Thy1*-network and the *Tubb3*-network

When we took the top 2000 correlates (an arbitrary cut-off) of each of the six subtype specific markers (*Cartpt, Jam2, Kcng4, Opn4, Hoxd10*, and *Spp1*) and examined the distributions of these markers across the *Thy1*- and the *Tubb3*-networks, we were able to see the relative interplay between these networks (Table 3). For *Spp1* and *Hoxd10*, the majority of the overlap was seen with genes from the *Tubb3*-network. The picture was quite different for the remaining subtype-specific markers. For *Cartpt, Opn4, Kcng4*, and *Jam2*, the overlap was dominated by similarities with the *Thy1*-network. This analysis was also conducted using only the genes with a Pearson correlation value above 0.6 (adj. *p* < 0.02) and it displayed a similar trend (Table 3). Taken together these data indicate that each of the RGC subtypes has a unique interplay with the *Thy1*- and the *Tubb3*-networks. The dramatic differences in overlap between correlates may reflect the differential expression of the *Thy1*- and *Tubb3*-networks in different RGC subtypes. These relationships can also be seen in the network map (Figure 5).

**Table 3:** **Overlap of subtype-specific RGC markers with genes from the *Thy1*- and the *Tubb3*-network**.

**Subtype Marker**	**Top 2000**		
	***Thy1***	***Tubb3***	**Total Overlap (of 4000)**
Cartpt	1022	54	1076 (27%)
Jam2	382	25	407 (10.2%)
Kcng4	326	241	567 (14.2%)
Opn4	713	185	898 (22.5%)
Hoxd10	10	19	29 (0.7%)
Spp1	29	756	1785 (19.6%)
**Subtype Marker**	**Pearson *r* > 0.6**		
	***Thy1***	***Tubb3***	**Total Overlap (of 9982)**
Cartpt	2482	0	2482 (24.9%)
Jam2	1030	3	1033 (10.3%)
Kcng4	886	207	1093 (10.9%)
Opn4	1086	56	1142 (11.4%)
Hoxd10	4	17	21 (0.2%)
Spp1	13	9	22 (0.2%)

For the top table, the top 2000 correlates of both networks served as comparison, whereas a Pearson r cutoff of >0.6 was chosen for the lower table, corresponding to a Bonferroni-corrected value of p < 0.02.

### Susceptibility of RGC marker genes to glaucomatous nerve damage

**Figure 7:**
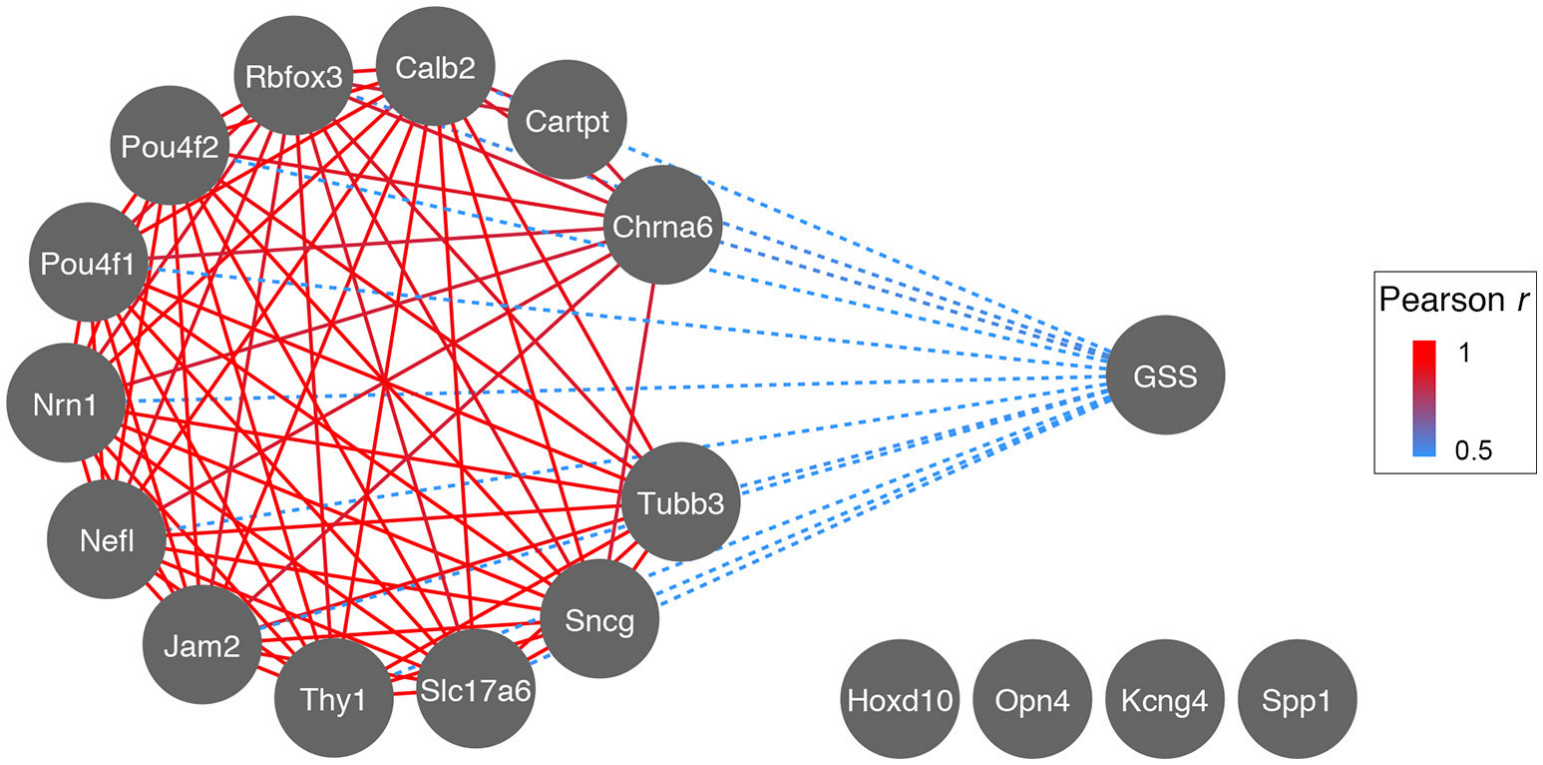
**Correlation network of Glaucoma Severity Score (GSS) to RGC markers during glaucoma progression**. The GSS is a visual grading system identifying axonal damage in the optic nerve and consists of 4 stages: no damage, light, medium, and severe damage. These stages are inversely correlated (dashed blue) to the expression levels of most RGC markers, suggesting that as RGCs die and axonal damage increases, mRNA expression of RGC marker genes decreases (most likely due to decrease in RGC number). The decrease of RGC marker gene expression is strongly correlated across glaucoma stages (red, *r* for all >0.9). Markers for ipRGCs (*Opn4*) and alpha-RGCs (*Kcng4* and *Spp1*) do not correlate to GSS, possibly indicating their preferential survival.

The identification of RGC subtypes has raised the question whether or not there is a difference in susceptibility to nerve damage, subsequent cell survival, or axon regeneration. In order to test this hypothesis on a transcriptional level, we used publicly available microarray data generated from DBA/2J glaucomatous eyes (Howell et al., 2011) and correlated glaucoma severity score (GSS) with expression levels of RGC markers (Figure 7). As expected, expression levels of all general RGC markers decreased as nerve damage increased (implicated by the negative correlation between GSS and RGC markers in Figure 7, dashed blue line). This was also the case for two subtype-specific markers, *Jam2* and *Cartpt*. Interestingly, expression levels of markers for alpha-RGCs (*Kcng4* and *Spp1*) and ipRGCs (*Opn4*) did not correlate to GSS (cutoff *r* < 0.6), suggesting that these two RGC subtypes are differentially susceptible to nerve damage.

## Discussion

Using the DoD CDMRP Retina expression microarray dataset to examine correlates of known RGC markers across the BXD RI strain set, this study revealed two distinct gene networks regulating normal RGC function. Detecting these transcriptional networks with high statistical power required tissue from 55 RI strains with 4 biological replicates per strain. The bioinformatic tools on GeneNetwork allowed us to extract gene networks from a complex structure such as the mouse retina (Geisert et al., 2009; Keeley et al., 2014). Interestingly, almost all of the general RGC markers described in the literature (see Table 1) selectively group with one of two genetic networks, the *Thy1*-network or the *Tubb3*-network. Both *Thy1* (Barnstable and Drager, 1984) and *Tubb3* (Snow and Robson, 1994) are believed to be generalized markers for RGCs. Nonetheless, these markers segregate into two distinct genetic networks. Two scenarios could explain the biological basis of correlated genes that are co-regulated in BXD RI mice: (I) differences in cell number of each RGC subtype across the BXD strains, or (II), differences in gene expression within individual RGC subtypes (Geisert et al., 2009; Keeley et al., 2014). Currently, we are not able to distinguish between these two potential explanations. Our analysis also revealed that these networks have unique biological functions.

Both the *Thy1*- and the *Tubb3*-networks contained large groups of genes that were consistent with a neuronal phenotype. However, they were each enriched in distinct functional roles. The *Thy1*-network appeared to be functionally involved in neuronal development and maintenance, as well as axon guidance. Some of the highest correlates of this network (*Gsk3a, Srgap1, Arhgap44, Ncdn, L1cam*, and *Lrrc4b)* were all previously reported to affect dendrite or axon pathfinding and outgrowth (Schwaibold and Brandt, 2008; Cherry et al., 2011; Ip et al., 2011; Galic et al., 2014). Therefore, this network could be directly involved in RGC process extension, potentially including dendrites and axons projecting to the brain. Furthermore, the network was enriched in transmembrane ion transporters. This included several calcium- and sodium-gated channel subunits, all of which have been previously localized to RGCs (Lipton and Tauck, 1987; Farrell et al., 2014).

The *Tubb3*-network contained genes that differed in function from those of the *Thy1*-network. The biological processes enriched in this group included protein polymerization and organic substrate metabolic processes. Under molecular processes, the *Tubb3*-network was enriched for GTP binding proteins and GTPase activity, two mechanisms known to be essential for cellular transport involving the cytoskeleton (Roychowdhury and Rasenick, 2008; Schappi et al., 2014). The highest two correlates of the Tubb3-network were *Rab15* and *Rab4b*, two G-proteins that are known to play an important role in endosome formation and vesicle movement along actin and tubulin networks (Zuk and Elferink, 2000; Falk et al., 2014). Several other genes in this list were associated with intracellular trafficking, such as sorting nexin 32 (*Snx32*, a molecule that links transport vesicles to dynactin (Wassmer et al., 2009), vesicle associated membrane protein 1 (*Vamp1*, a SNARE protein important for linking vesicles to the target membrane; Hasan et al., 2010), *Pigu* (a GPI anchor protein; Guo et al., 2004), and *Tmem9* (part of the lysosomal membrane; Kveine et al., 2002). These genes were highly correlated with and specific to the *Tubb3*-network.

These findings suggest that the *Tubb3*-network is functionally associated with cytoskeleton and vesicle transport in RGCs. Since neurons are known for the significant amount of cytoskeletal proteins necessary to maintain dendritic and axonal integrity (Kevenaar and Hoogenraad, 2015), it can be hypothesized that the *Tubb3*-network is involved in maintenance of these structural elements.

Taken together, this analysis indicates that each network has distinct molecular functions and that both of these networks can exist independently within a single RGC.

The functional differences between networks were mirrored by the transcription factor binding site (TFBS) analysis for promoters of their respective genes. Members of the *Thy1*-network showed significant enrichment in TFBS for proteins regulating cell cycle, growth and apoptosis (Tu et al., 2013). Genes belonging to the *Tubb3*-network, however, showed an over-representation of TFBS for transcription factors known to establish cell fate decisions during development, particularly in later stages. Proteins such as OTX1, PAX6, SIX6, or POU4F1 belonged to the latter group (Zagozewski et al., 2014). *Pou4f1* was not originally found in the correlates of the *Tubb3*-network, which might seem surprising. A possible explanation for this is that the *Thy1*-network, which correlates well with the expression of *Pou4f1* across the BXD strains, acts upstream of the *Tubb3*-network. In a developmental sense, this could suggest that members of the *Thy1*-network establish expression of *Pou4f1*, which can then bind to members of the *Tubb3*-network to influence their transcription. Because *Pou4f1* was originally not found to be part of the *Tubb3*-network but the *Thy1*-network instead, we hypothesize here that basic cell cycle tasks such as survival decisions are managed by members of the *Thy1*-network, while members of the *Tubb3*-network regulate the fine-tuning of RGC development, possibly even influencing subtype commitments.

The identification of neuronal subtypes has gained a considerable amount of attention in neuroscience across a variety of scientific disciplines (Hoshino, 2012; Russ and Kaltschmidt, 2014). This is especially the case for RGC subtypes (Kay et al., 2011; Dhande et al., 2013; Sanes and Masland, 2015). The establishment of transgenic mouse models has recently transformed the field, and new paradigms are starting to emerge. For example, it is becoming increasingly evident that RGC subtypes seem to be differentially susceptible to nerve injury to the point of having different chances of survival or regeneration (Kay et al., 2011; Dhande et al., 2013; Pérez de Sevilla Müller et al., 2014; Duan et al., 2015; Sanes and Masland, 2015). In order to develop new therapeutic approaches to nerve regeneration, it may be necessary to tease apart the transcriptional programs of injury-susceptible and non-susceptible RGC subtypes.

When we investigated the heat maps of subtype-specific gene correlates, we found that two pairs—*Kcng4/Opn4*, or *Jam2/Cartpt*—showed similarity to two different heat map bands from the *Thy1*-network. *Kcng4* marks alpha-RGCs, while *Opn4* marks ipRGCs, and these two subtypes were recently found to be most resistant to optic nerve crush (Pérez de Sevilla Müller et al., 2014; Duan et al., 2015). Since the regulatory pattern of those two gene pairs was very similar based on heat map analysis of their co-varying genes, it would be interesting to systematically investigate if these upstream modulatory loci are responsible for increased neuronal survival or regeneration. Bioinformatic analysis of RGC marker susceptibility to glaucomatous nerve damage supported the notion that alpha-RGCs and ipRGCs were also most resistant to neurodegeneration. The decrease in mRNA expression of alpha-RGC markers (*Kcng4, Spp1*) and ipRCG markers (*Opn4*) did not correlate with GSS (a visual grading scale for optic nerve axon damage), whereas the overwhelming majority of RGC markers did.

Interestingly, the discovery of *Spp1* marking a particular RGC subtype had only been made after crush injury to the optic nerve in a mouse model (Pérez de Sevilla Müller et al., 2014; Duan et al., 2015). In this study, it was found that alpha-RGCs secreting Osteopontin (the protein made from the gene *Spp1*) were most resistant to optic nerve crush among all RGC subtypes, and that this protective effect was due to Osteopontin. We were not able to identify putative upstream modulatory loci for this gene due to the lack of distinct trans-QTL bands. This suggests that *Spp1* is part of a genetic network that needs to be activated by neuronal injury before it can be co-regulated in a more RGC-specific way. This phenomenon has been observed elsewhere for other genes following retinal injury (Templeton et al., 2013b).

In summary, we have identified multiple loci modulating RGC function in the BXD mouse strain set, and we have provided *in silico* evidence for the differential susceptibility of RGC subtypes to neurodegeneration and cell death. One caveat of this study is the fact that the performed analysis is only a correlative one. Proving a biological cause will require experimental manipulation of the mentioned genes and examination of the effects *in vivo* or *in vitro*. Nevertheless, our findings enhance the understanding of the RGC's normal transcriptome, as they are the first to describe gene regulatory networks for some of their subtypes. They may serve others and us as a reference for future studies on RGC subtype identification and their susceptibility to injury.

## Materials and methods

### Animals

All of the procedures involving mice were approved by IACUC at Emory University and adhered to the ARVO Statement for the Use of Animals in Research.

### Microarray datasets

Two microarray datasets were used in this project as part of a comprehensive meta-analysis.

- The Department of Defense (DoD) Normal Retina Database (May2015). This is the most comprehensive retina microarray dataset and creation is described in King et al. (2015). The DoD Normal Retina Dataset consists of 222 microarrays from 55 different strains BXD mice, the parental strain C57BL/6J, the parental strain DBA/2J and an F1 cross.
- The Howell et al. (2011), DBA/2J Glaucoma Retina M430 2.0 (Sep11) RMA database. This dataset consists of retinal tissue dissected from 40 DBA/2J mouse eyes at 10.5 months of age showing varying levels of RGC damage due to naturally occuring glaucoma in this mouse strain (as graded by visual inspection of the optic nerve). Twenty eyes served as negative controls. The generation of this dataset is described in Howell et al. (2011).

All of the used datasets are publicly accessible through www.genenetwork.org.

### Statistical analysis and plot generation

GeneNetwork provided the platform for correlation analysis, principal component generation, and linkage analysis. In general, datasets were queried for gene symbols, downloaded from GeneNetwork, and additional analysis was performed in R whenever necessary. *P*-values mentioned in relation to Pearson's coefficient throughout this paper are based on pair-wise comparisons. All *p*-values were Bonferroni-adjusted for 36,012 genes, which is equal to the number of genes captured on the microarray after accounting for replicated and wrongly annotated probes. Plots were generated with R and the dplyr and ggplot2 packages. Cytoscape version 3.2.1 was used to generate gene network graphs.

### Gene ontology analysis

GO analysis was performed using WebGestalt (Wang et al., 2013). The top 500 genes of each network were used to compile a gene list and duplicates were removed. GO term enrichment was calculated using Affymetrix MouseGene 2.0 ST probe set IDs against a background dataset from the same chip. GO trees are appended as expanded view data. All *p*-values presented in the GO analysis are corrected for multiple comparisons using the Bonferroni method.

### Candidate gene analysis

A list of candidate genes for putative upstream modulators of both networks was created by extracting genes with cis-QTLs from the loci showing trans-bands. For a cis-QTL considered to be a good candidate gene, it should fulfill three conditions: (i) be expressed above background, (ii) correlate well with the trait used to create the trans-bands, and (iii) have a nucleotide variation that alters either protein structure or other regulatory elements (such as promoters or enhancers). These lists are appended as Supplemental Tables.

### Transcription factor binding site analysis

Gene lists including the top 500 correlated genes in each network were compiled, duplicates were removed, and uploaded to BIOBASE. The TRANSFAC FMatch algorithm was used to search for overrepresented neuron-specific transcription factor binding sites in the gene lists using the “best supported promoter” model for each gene (Matys et al., 2006). As a background dataset for both networks, the opposite network was chosen. Resulting *p*-values were Bonferroni-adjusted. The enrichment window for a gene's promoter was chosen to be −5000 to +100 bp of its best supported transcription start site. “Minimize false positives” was selected as cut-off method. The TRANSFAC data version was 2016.2, and only high-quality matrices were used for the analysis.

### WGCNA analysis

Weighted gene network analysis was performed using the WGCNA package in the R environment (Langfelder and Horvath, 2008). A soft thresholding power of 10 was used to calculate the adjacency matrix based on the criterion of scale-free topology (Zhang and Horvath, 2005). Modules were identified using the following parameters for the blockwiseModules function: minimum module size of 100, merge cut height of 0.25, and maximum block size of 2000. The top 2000 correlates of *Thy1* and *Tubb3* were then each merged with their module affiliation by Affymetrix Probe Set ID and the resulting list was checked for overlaps.

### Immunohistochemistry

Two 60 day-old Thy1-CFP C57BL/6 transgenic mouse (Jax identifier B6.Cg-Tg(Thy1-CFP)23Jrs/J) were deeply anesthetized with a mixture of 13 mg/kg of xylazine and 87 mg/kg of ketamine and intracardially perfused with 0.9% saline followed by 4% paraformaldehyde. Retinas were dissected and flat-mounted on glass slides following a standard protocol. Retinas were blocked in 4% bovine serum albumin in PBS overnight. One retina was stained with anti-TUJ1 (a gift from Anthony Frankforter, dilution 1:1000) and anti-GFP (Invitrogen, 1:500) antibodies overnight and then labeled with appropriate Alexa Fluor-conjugated secondary antibodies. One retina served as negative secondary antibody control (data not shown). High-resolution Z-stacks were captured on a Nikon confocal microscope with the Nikon C1 software throughout the entire ganglion cell layer only. Z-stacks were collapsed using FiJi (Schindelin et al., 2012) and the whole image was adjusted for contrast and brightness using Adobe Photoshop.

## Accessibility of data

The data presented in this article is publicly available under www.genenetwork.org and can be downloaded under http://genenetwork.org/webqtl/main.py?FormID=sharinginfo&GN_AccessionId=709.

## Author contributions

FS, EG provided the initial design of the study. FS performed experiments and bioinformatic analyses with assistance from EG. FS, EG wrote the paper with input from RW and RL. RW supplied the datasets through the GeneNetwork platform and is responsible for its maintenance. RL has provided intellectual framework for this project as part of an ongoing collaborative effort between his laboratory and the laboratories of EG, RW.

### Conflict of interest statement

The authors declare that the research was conducted in the absence of any commercial or financial relationships that could be construed as a potential conflict of interest.
