# Supplementary material for: Genetic Networks in Mouse Retinal Ganglion Cells
Source: Front Genet. 2016 Sep 28;7:169. doi: 10.3389/fgene.2016.00169 (PMC5039302; doi:10.3389/fgene.2016.00169)
Supplement: Supplementary file 3 [file DataSheet1.ZIP › Thy1-network GO/files/final_sig_file_1470844800.html]

Anchored HTML File of EIDs


|  |  |
| --- | --- |
|  | WEB-based GEne SeT AnaLysis Toolkit |
|  |  |
|  | ***Translating gene lists into biological insights...*** |
|  |  |

---

  

| **Database:biological process      &nbspName:nervous system development      &nbspID:GO:0007399** | | | | | | |
| --- | --- | --- | --- | --- | --- | --- |
| C=1372; O=87; E=30.60; R=2.84; rawP=3.96e-19; adjP=7.55e-16 | | | | | | |
| Index | UserID | Value | Gene Symbol | Gene Name | EntrezGene | Ensembl |
| 1 | 17309287 | NA | Pou4f1 | POU domain, class 4, transcription factor 1 | 18996 | ENSMUSG00000048349 |
| 2 | 17419097 | NA | Bai2 | brain-specific angiogenesis inhibitor 2 | 230775 | ENSMUSG00000028782 |
| 3 | 17516462 | NA | Thy1 | thymus cell antigen 1, theta | 21838 | ENSMUSG00000032011 |
| 4 | 17439511 | NA | Prdm8 | PR domain containing 8 | 77630 | ENSMUSG00000035456 |
| 5 | 17314164 | NA | Mapk8ip2 | mitogen-activated protein kinase 8 interacting protein 2 | 60597 | ENSMUSG00000022619 |
| 6 | 17354629 | NA | Ndst1 | N-deacetylase/N-sulfotransferase (heparan glucosaminyl) 1 | 15531 | ENSMUSG00000054008 |
| 7 | 17503122 | NA | Cacna1a | calcium channel, voltage-dependent, P/Q type, alpha 1A subunit | 12286 | ENSMUSG00000034656 |
| 8 | 17376685 | NA | Plcb1 | phospholipase C, beta 1 | 18795 | ENSMUSG00000051177 |
| 9 | 17487884 | NA | Gsk3a | glycogen synthase kinase 3 alpha | 606496 | ENSMUSG00000057177 |
| 10 | 17506808 | NA | Trim67 | tripartite motif-containing 67 | 330863 | ENSMUSG00000036913 |
| 11 | 17342065 | NA | Mapk8ip3 | mitogen-activated protein kinase 8 interacting protein 3 | 30957 | ENSMUSG00000024163 |
| 12 | 17494596 | NA | Tpp1 | tripeptidyl peptidase I | 12751 | ENSMUSG00000030894 |
| 13 | 17226891 | NA | Cntn2 | contactin 2 | 21367 | ENSMUSG00000053024 |
| 14 | 17509907 | NA | Gatad2a | GATA zinc finger domain containing 2A | 234366 | ENSMUSG00000036180 |
| 15 | 17253376 | NA | Sez6 | seizure related gene 6 | 20370 | ENSMUSG00000000632 |
| 16 | 17223283 | NA | Satb2 | special AT-rich sequence binding protein 2 | 212712 | ENSMUSG00000038331 |
| 17 | 17268786 | NA | Neurod2 | neurogenic differentiation 2 | 18013 | ENSMUSG00000038255 |
| 18 | 17475564 | NA | Numbl | numb-like | 18223 | ENSMUSG00000063160 |
| 19 | 17484419 | NA | Kndc1 | kinase non-catalytic C-lobe domain (KIND) containing 1 | 76484 | ENSMUSG00000066129 |
| 20 | 17517073 | NA | Drd2 | dopamine receptor D2 | 13489 | ENSMUSG00000032259 |
| 21 | 17505689 | NA | Aars | alanyl-tRNA synthetase | 234734 | ENSMUSG00000031960 |
| 22 | 17461897 | NA | Atg7 | autophagy related 7 | 74244 | ENSMUSG00000030314 |
| 23 | 17542419 | NA | L1cam | L1 cell adhesion molecule | 16728 | ENSMUSG00000031391 |
| 24 | 17321582 | NA | Faim2 | Fas apoptotic inhibitory molecule 2 | 72393 | ENSMUSG00000023011 |
| 25 | 17469814 | NA | Atp2b2 | ATPase, Ca++ transporting, plasma membrane 2 | 11941 | ENSMUSG00000030302 |
| 26 | 17487249 | NA | Mark4 | MAP/microtubule affinity-regulating kinase 4 | 232944 | ENSMUSG00000030397 |
| 27 | 17292753 | NA | Gprin1 | G protein-regulated inducer of neurite outgrowth 1 | 26913 | ENSMUSG00000069227 |
| 28 | 17536720 | NA | Nlgn3 | neuroligin 3 | 245537 | ENSMUSG00000031302 |
| 29 | 17346231 | NA | Ptprs | protein tyrosine phosphatase, receptor type, S | 19280 | ENSMUSG00000013236 |
| 30 | 17314872 | NA | Smarcd1 | SWI/SNF related, matrix associated, actin dependent regulator of chromatin, subfamily d, member 1 | 83797 | ENSMUSG00000023018 |
| 31 | 17450536 | NA | Barhl2 | BarH-like 2 (Drosophila) | 104382 | ENSMUSG00000034384 |
| 32 | 17317327 | NA | Mtss1 | metastasis suppressor 1 | 211401 | ENSMUSG00000022353 |
| 33 | 17502039 | NA | Rab3a | RAB3A, member RAS oncogene family | 19339 | ENSMUSG00000031840 |
| 34 | 17332495 | NA | Dscam | Down syndrome cell adhesion molecule | 13508 | ENSMUSG00000050272 |
| 35 | 17307905 | NA | Dpysl2 | dihydropyrimidinase-like 2 | 12934 | ENSMUSG00000022048 |
| 36 | 17453430 | NA | Limk1 | LIM-domain containing, protein kinase | 16885 | ENSMUSG00000029674 |
| 37 | 17430140 | NA | Ncdn | neurochondrin | 26562 | ENSMUSG00000028833 |
| 38 | 17452038 | NA | Dtx1 | deltex 1 homolog (Drosophila) | 14357 | ENSMUSG00000029603 |
| 39 | 17257197 | NA | Mapt | microtubule-associated protein tau | 17762 | ENSMUSG00000018411 |
| 40 | 17292011 | NA | Tfap2a | transcription factor AP-2, alpha | 21418 | ENSMUSG00000021359 |
| 41 | 17485152 | NA | Brsk2 | BR serine/threonine kinase 2 | 75770 | ENSMUSG00000053046 |
| 42 | 17293045 | NA | Spock1 | sparc/osteonectin, cwcv and kazal-like domains proteoglycan 1 | 20745 | ENSMUSG00000056222 |
| 43 | 17397645 | NA | Smad9 | SMAD family member 9 | 55994 | ENSMUSG00000027796 |
| 44 | 17269439 | NA | Hap1 | huntingtin-associated protein 1 | 15114 | ENSMUSG00000006930 |
| 45 | 17215820 | NA | Gpc1 | glypican 1 | 14733 | ENSMUSG00000034220 |
| 46 | 17451140 | NA | Ulk1 | unc-51 like kinase 1 | 22241 | ENSMUSG00000029512 |
| 47 | 17368550 | NA | Rxra | retinoid X receptor alpha | 20181 | ENSMUSG00000015846 |
| 48 | 17384021 | NA | Stxbp1 | syntaxin binding protein 1 | 20910 | ENSMUSG00000026797 |
| 49 | 17520198 | NA | Rasgrf1 | RAS protein-specific guanine nucleotide-releasing factor 1 | 19417 | ENSMUSG00000032356 |
| 50 | 17302600 | NA | Slitrk5 | SLIT and NTRK-like family, member 5 | 75409 | ENSMUSG00000033214 |
| 51 | 17489052 | NA | Aplp1 | amyloid beta (A4) precursor-like protein 1 | 11803 | ENSMUSG00000006651 |
| 52 | 17382496 | NA | Grin1 | glutamate receptor, ionotropic, NMDA1 (zeta 1) | 14810 | ENSMUSG00000026959 |
| 53 | 17367921 | NA | Grin1 | glutamate receptor, ionotropic, NMDA1 (zeta 1) | 14810 | ENSMUSG00000026959 |
| 54 | 17357959 | NA | Gnaq | guanine nucleotide binding protein, alpha q polypeptide | 14682 | ENSMUSG00000024639 |
| 55 | 17266107 | NA | Abr | active BCR-related gene | 109934 | ENSMUSG00000017631 |
| 56 | 17335204 | NA | Anks1 | ankyrin repeat and SAM domain containing 1 | 224650 | ENSMUSG00000024219 |
| 57 | 17272926 | NA | Nptx1 | neuronal pentraxin 1 | 18164 | ENSMUSG00000025582 |
| 58 | 17301823 | NA | Gfra2 | glial cell line derived neurotrophic factor family receptor alpha 2 | 14586 | ENSMUSG00000022103 |
| 59 | 17211347 | NA | Tfap2b | transcription factor AP-2 beta | 21419 | ENSMUSG00000025927 |
| 60 | 17406760 | NA | Sema4a | sema domain, immunoglobulin domain (Ig), transmembrane domain (TM) and short cytoplasmic domain, (semaphorin) 4A | 20351 | ENSMUSG00000028064 |
| 61 | 17524895 | NA | Elavl3 | ELAV (embryonic lethal, abnormal vision, Drosophila)-like 3 (Hu antigen C) | 15571 | ENSMUSG00000003410 |
| 62 | 17346155 | NA | Sema6b | sema domain, transmembrane domain (TM), and cytoplasmic domain, (semaphorin) 6B | 20359 | ENSMUSG00000001227 |
| 63 | 17435834 | NA | Dpysl5 | dihydropyrimidinase-like 5 | 65254 | ENSMUSG00000029168 |
| 64 | 17354299 | NA | Sema6a | sema domain, transmembrane domain (TM), and cytoplasmic domain, (semaphorin) 6A | 20358 | ENSMUSG00000019647 |
| 65 | 17385374 | NA | Nr4a2 | nuclear receptor subfamily 4, group A, member 2 | 18227 | ENSMUSG00000026826 |
| 66 | 17235663 | NA | Pip5k1c | phosphatidylinositol-4-phosphate 5-kinase, type 1 gamma | 18717 | ENSMUSG00000034902 |
| 67 | 17378721 | NA | Src | Rous sarcoma oncogene | 20779 | ENSMUSG00000027646 |
| 68 | 17500301 | NA | Gpr124 | G protein-coupled receptor 124 | 78560 | ENSMUSG00000031486 |
| 69 | 17407124 | NA | Chrnb2 | cholinergic receptor, nicotinic, beta polypeptide 2 (neuronal) | 11444 | ENSMUSG00000027950 |
| 70 | 17486930 | NA | Grlf1 | glucocorticoid receptor DNA binding factor 1 | 232906 | ENSMUSG00000058230 |
| 71 | 17441595 | NA | Tbx3 | T-box 3 | 21386 | ENSMUSG00000018604 |
| 72 | 17335145 | NA | Pacsin1 | protein kinase C and casein kinase substrate in neurons 1 | 23969 | ENSMUSG00000040276 |
| 73 | 17243229 | NA | Atcay | ataxia, cerebellar, Cayman type homolog (human) | 16467 | ENSMUSG00000034958 |
| 74 | 17345542 | NA | Ppp2r5d | protein phosphatase 2, regulatory subunit B (B56), delta isoform | 21770 | ENSMUSG00000059409 |
| 75 | 17364665 | NA | Slit1 | slit homolog 1 (Drosophila) | 20562 | ENSMUSG00000025020 |
| 76 | 17515277 | NA | Smarca4 | SWI/SNF related, matrix associated, actin dependent regulator of chromatin, subfamily a, member 4 | 20586 | ENSMUSG00000032187 |
| 77 | 17510696 | NA | Pou4f2 | POU domain, class 4, transcription factor 2 | 18997 | ENSMUSG00000031688 |
| 78 | 17256579 | NA | Cntnap1 | contactin associated protein-like 1 | 53321 | ENSMUSG00000017167 |
| 79 | 17477468 | NA | Lrrc4b | leucine rich repeat containing 4B | 272381 | ENSMUSG00000047085 |
| 80 | 17336987 | NA | Bag6 | BCL2-associated athanogene 6 | 224727 | ENSMUSG00000024392 |
| 81 | 17401650 | NA | Amigo1 | adhesion molecule with Ig like domain 1 | 229715 | ENSMUSG00000050947 |
| 82 | 17356924 | NA | Nrxn2 | neurexin II | 18190 | ENSMUSG00000033768 |
| 83 | 17283897 | NA | Ccdc85c | coiled-coil domain containing 85C | 668158 | ENSMUSG00000084883 |
| 84 | 17485574 | NA | Mboat7 | membrane bound O-acyltransferase domain containing 7 | 77582 | ENSMUSG00000035596 |
| 85 | 17260261 | NA | Camk2b | calcium/calmodulin-dependent protein kinase II, beta | 12323 | ENSMUSG00000057897 |
| 86 | 17267039 | NA | Lhx1 | LIM homeobox protein 1 | 16869 | ENSMUSG00000018698 |
| 87 | 17259810 | NA | Inpp5j | inositol polyphosphate 5-phosphatase J | 170835 | ENSMUSG00000034570 |
| 88 | 17515843 | NA | Kirrel3 | kin of IRRE like 3 (Drosophila) | 67703 | ENSMUSG00000032036 |

  
  

| **Database:biological process      &nbspName:multicellular organismal signaling      &nbspID:GO:0035637** | | | | | | |
| --- | --- | --- | --- | --- | --- | --- |
| C=576; O=53; E=12.85; R=4.13; rawP=1.71e-18; adjP=1.63e-15 | | | | | | |
| Index | UserID | Value | Gene Symbol | Gene Name | EntrezGene | Ensembl |
| 1 | 17318923 | NA | Cacng2 | calcium channel, voltage-dependent, gamma subunit 2 | 12300 | ENSMUSG00000019146 |
| 2 | 17368550 | NA | Rxra | retinoid X receptor alpha | 20181 | ENSMUSG00000015846 |
| 3 | 17461923 | NA | Syn2 | synapsin II | 20965 | ENSMUSG00000009394 |
| 4 | 17384021 | NA | Stxbp1 | syntaxin binding protein 1 | 20910 | ENSMUSG00000026797 |
| 5 | 17342719 | NA | Grm4 | glutamate receptor, metabotropic 4 | 268934 | ENSMUSG00000063239 |
| 6 | 17436545 | NA | Nat8l | N-acetyltransferase 8-like | 269642 | ENSMUSG00000048142 |
| 7 | 17520198 | NA | Rasgrf1 | RAS protein-specific guanine nucleotide-releasing factor 1 | 19417 | ENSMUSG00000032356 |
| 8 | 17529218 | NA | Htr1b | 5-hydroxytryptamine (serotonin) receptor 1B | 15551 | ENSMUSG00000049511 |
| 9 | 17337513 | NA | Gabbr1 | gamma-aminobutyric acid (GABA) B receptor, 1 | 54393 | ENSMUSG00000024462 |
| 10 | 17243717 | NA | Syn3 | synapsin III | 27204 | ENSMUSG00000059602 |
| 11 | 17314164 | NA | Mapk8ip2 | mitogen-activated protein kinase 8 interacting protein 2 | 60597 | ENSMUSG00000022619 |
| 12 | 17509944 | NA | Ncan | neurocan | 13004 | ENSMUSG00000002341 |
| 13 | 17502603 | NA | Rasd2 | RASD family, member 2 | 75141 | ENSMUSG00000034472 |
| 14 | 17530733 | NA | Grm2 | glutamate receptor, metabotropic 2 | 108068 | ENSMUSG00000023192 |
| 15 | 17503122 | NA | Cacna1a | calcium channel, voltage-dependent, P/Q type, alpha 1A subunit | 12286 | ENSMUSG00000034656 |
| 16 | 17310772 | NA | Ctnnd2 | catenin (cadherin associated protein), delta 2 | 18163 | ENSMUSG00000022240 |
| 17 | 17329516 | NA | Fgf12 | fibroblast growth factor 12 | 14167 | ENSMUSG00000022523 |
| 18 | 17400521 | NA | Sv2a | synaptic vesicle glycoprotein 2 a | 64051 | ENSMUSG00000038486 |
| 19 | 17308939 | NA | Pcdh8 | protocadherin 8 | 18530 | ENSMUSG00000036422 |
| 20 | 17504130 | NA | Cx3cl1 | chemokine (C-X3-C motif) ligand 1 | 20312 | ENSMUSG00000031778 |
| 21 | 17382496 | NA | Grin1 | glutamate receptor, ionotropic, NMDA1 (zeta 1) | 14810 | ENSMUSG00000026959 |
| 22 | 17367921 | NA | Grin1 | glutamate receptor, ionotropic, NMDA1 (zeta 1) | 14810 | ENSMUSG00000026959 |
| 23 | 17226891 | NA | Cntn2 | contactin 2 | 21367 | ENSMUSG00000053024 |
| 24 | 17243057 | NA | Ap3d1 | adaptor-related protein complex 3, delta 1 subunit | 11776 | ENSMUSG00000020198 |
| 25 | 17446123 | NA | Kcnh2 | potassium voltage-gated channel, subfamily H (eag-related), member 2 | 16511 | ENSMUSG00000038319 |
| 26 | 17253376 | NA | Sez6 | seizure related gene 6 | 20370 | ENSMUSG00000000632 |
| 27 | 17263011 | NA | Glra1 | glycine receptor, alpha 1 subunit | 14654 | ENSMUSG00000000263 |
| 28 | 17269464 | NA | Jup | junction plakoglobin | 16480 | ENSMUSG00000001552 |
| 29 | 17268786 | NA | Neurod2 | neurogenic differentiation 2 | 18013 | ENSMUSG00000038255 |
| 30 | 17517073 | NA | Drd2 | dopamine receptor D2 | 13489 | ENSMUSG00000032259 |
| 31 | 17469814 | NA | Atp2b2 | ATPase, Ca++ transporting, plasma membrane 2 | 11941 | ENSMUSG00000030302 |
| 32 | 17435528 | NA | Dpp6 | dipeptidylpeptidase 6 | 13483 | ENSMUSG00000061576 |
| 33 | 17536720 | NA | Nlgn3 | neuroligin 3 | 245537 | ENSMUSG00000031302 |
| 34 | 17502039 | NA | Rab3a | RAB3A, member RAS oncogene family | 19339 | ENSMUSG00000031840 |
| 35 | 17407124 | NA | Chrnb2 | cholinergic receptor, nicotinic, beta polypeptide 2 (neuronal) | 11444 | ENSMUSG00000027950 |
| 36 | 17461868 | NA | Slc6a1 | solute carrier family 6 (neurotransmitter transporter, GABA), member 1 | 232333 | ENSMUSG00000030310 |
| 37 | 17307905 | NA | Dpysl2 | dihydropyrimidinase-like 2 | 12934 | ENSMUSG00000022048 |
| 38 | 17239234 | NA | Grm1 | glutamate receptor, metabotropic 1 | 14816 | ENSMUSG00000019828 |
| 39 | 17361454 | NA | Cnih2 | cornichon homolog 2 (Drosophila) | 12794 | ENSMUSG00000024873 |
| 40 | 17430140 | NA | Ncdn | neurochondrin | 26562 | ENSMUSG00000028833 |
| 41 | 17496763 | NA | Stx1b | syntaxin 1B | 56216 | ENSMUSG00000030806 |
| 42 | 17441453 | NA | Nos1 | nitric oxide synthase 1, neuronal | 18125 | ENSMUSG00000029361 |
| 43 | 17540501 | NA | Syn1 | synapsin I | 20964 | ENSMUSG00000037217 |
| 44 | 17256579 | NA | Cntnap1 | contactin associated protein-like 1 | 53321 | ENSMUSG00000017167 |
| 45 | 17521327 | NA | Cacna2d2 | calcium channel, voltage-dependent, alpha 2/delta subunit 2 | 56808 | ENSMUSG00000010066 |
| 46 | 17477468 | NA | Lrrc4b | leucine rich repeat containing 4B | 272381 | ENSMUSG00000047085 |
| 47 | 17477714 | NA | Slc17a7 | solute carrier family 17 (sodium-dependent inorganic phosphate cotransporter), member 7 | 72961 | ENSMUSG00000070570 |
| 48 | 17401650 | NA | Amigo1 | adhesion molecule with Ig like domain 1 | 229715 | ENSMUSG00000050947 |
| 49 | 17516731 | NA | Scn2b | sodium channel, voltage-gated, type II, beta | 72821 | ENSMUSG00000070304 |
| 50 | 17532879 | NA | Syp | synaptophysin | 20977 | ENSMUSG00000031144 |
| 51 | 17356924 | NA | Nrxn2 | neurexin II | 18190 | ENSMUSG00000033768 |
| 52 | 17215820 | NA | Gpc1 | glypican 1 | 14733 | ENSMUSG00000034220 |
| 53 | 17260261 | NA | Camk2b | calcium/calmodulin-dependent protein kinase II, beta | 12323 | ENSMUSG00000057897 |
| 54 | 17394079 | NA | Rims4 | regulating synaptic membrane exocytosis 4 | 241770 | ENSMUSG00000035226 |

  
  

| **Database:biological process      &nbspName:localization      &nbspID:GO:0051179** | | | | | | |
| --- | --- | --- | --- | --- | --- | --- |
| C=3912; O=165; E=87.26; R=1.89; rawP=4.77e-18; adjP=3.03e-15 | | | | | | |
| Index | UserID | Value | Gene Symbol | Gene Name | EntrezGene | Ensembl |
| 1 | 17318923 | NA | Cacng2 | calcium channel, voltage-dependent, gamma subunit 2 | 12300 | ENSMUSG00000019146 |
| 2 | 17309287 | NA | Pou4f1 | POU domain, class 4, transcription factor 1 | 18996 | ENSMUSG00000048349 |
| 3 | 17378922 | NA | Slc32a1 | solute carrier family 32 (GABA vesicular transporter), member 1 | 22348 | ENSMUSG00000037771 |
| 4 | 17334545 | NA | Clcn7 | chloride channel 7 | 26373 | ENSMUSG00000036636 |
| 5 | 17461852 | NA | Slc6a11 | solute carrier family 6 (neurotransmitter transporter, GABA), member 11 | 243616 | ENSMUSG00000030307 |
| 6 | 17243717 | NA | Syn3 | synapsin III | 27204 | ENSMUSG00000059602 |
| 7 | 17399496 | NA | Adar | adenosine deaminase, RNA-specific | 56417 | ENSMUSG00000027951 |
| 8 | 17376685 | NA | Plcb1 | phospholipase C, beta 1 | 18795 | ENSMUSG00000051177 |
| 9 | 17329516 | NA | Fgf12 | fibroblast growth factor 12 | 14167 | ENSMUSG00000022523 |
| 10 | 17342065 | NA | Mapk8ip3 | mitogen-activated protein kinase 8 interacting protein 3 | 30957 | ENSMUSG00000024163 |
| 11 | 17226891 | NA | Cntn2 | contactin 2 | 21367 | ENSMUSG00000053024 |
| 12 | 17252995 | NA | Slc43a2 | solute carrier family 43, member 2 | 215113 | ENSMUSG00000038178 |
| 13 | 17322600 | NA | Mgrn1 | mahogunin, ring finger 1 | 17237 | ENSMUSG00000022517 |
| 14 | 17416325 | NA | Dhcr24 | 24-dehydrocholesterol reductase | 74754 | ENSMUSG00000034926 |
| 15 | 17223283 | NA | Satb2 | special AT-rich sequence binding protein 2 | 212712 | ENSMUSG00000038331 |
| 16 | 17370234 | NA | Dab2ip | disabled 2 interacting protein | 69601 | ENSMUSG00000026883 |
| 17 | 17361223 | NA | Adrbk1 | adrenergic receptor kinase, beta 1 | 110355 | ENSMUSG00000024858 |
| 18 | 17469814 | NA | Atp2b2 | ATPase, Ca++ transporting, plasma membrane 2 | 11941 | ENSMUSG00000030302 |
| 19 | 17401394 | NA | Kcnd3 | potassium voltage-gated channel, Shal-related family, member 3 | 56543 | ENSMUSG00000040896 |
| 20 | 17487805 | NA | Atp1a3 | ATPase, Na+/K+ transporting, alpha 3 polypeptide | 232975 | ENSMUSG00000040907 |
| 21 | 17450536 | NA | Barhl2 | BarH-like 2 (Drosophila) | 104382 | ENSMUSG00000034384 |
| 22 | 17502039 | NA | Rab3a | RAB3A, member RAS oncogene family | 19339 | ENSMUSG00000031840 |
| 23 | 17307905 | NA | Dpysl2 | dihydropyrimidinase-like 2 | 12934 | ENSMUSG00000022048 |
| 24 | 17290259 | NA | Ucn3 | urocortin 3 | 83428 | ENSMUSG00000044988 |
| 25 | 17526175 | NA | Abcg4 | ATP-binding cassette, sub-family G (WHITE), member 4 | 192663 | ENSMUSG00000032131 |
| 26 | 17540501 | NA | Syn1 | synapsin I | 20964 | ENSMUSG00000037217 |
| 27 | 17298874 | NA | Grid1 | glutamate receptor, ionotropic, delta 1 | 14803 | ENSMUSG00000041078 |
| 28 | 17292011 | NA | Tfap2a | transcription factor AP-2, alpha | 21418 | ENSMUSG00000021359 |
| 29 | 17521327 | NA | Cacna2d2 | calcium channel, voltage-dependent, alpha 2/delta subunit 2 | 56808 | ENSMUSG00000010066 |
| 30 | 17473155 | NA | Cacng7 | calcium channel, voltage-dependent, gamma subunit 7 | 81904 | ENSMUSG00000069806 |
| 31 | 17501692 | NA | Atp13a1 | ATPase type 13A1 | 170759 | ENSMUSG00000031862 |
| 32 | 17477714 | NA | Slc17a7 | solute carrier family 17 (sodium-dependent inorganic phosphate cotransporter), member 7 | 72961 | ENSMUSG00000070570 |
| 33 | 17361494 | NA | Pacs1 | phosphofurin acidic cluster sorting protein 1 | 107975 | ENSMUSG00000024855 |
| 34 | 17243162 | NA | Slc39a3 | solute carrier family 39 (zinc transporter), member 3 | 106947 | ENSMUSG00000046822 |
| 35 | 17317801 | NA | Kcnk9 | potassium channel, subfamily K, member 9 | 223604 | ENSMUSG00000036760 |
| 36 | 17527520 | NA | Scamp5 | secretory carrier membrane protein 5 | 56807 | ENSMUSG00000040722 |
| 37 | 17269439 | NA | Hap1 | huntingtin-associated protein 1 | 15114 | ENSMUSG00000006930 |
| 38 | 17532879 | NA | Syp | synaptophysin | 20977 | ENSMUSG00000031144 |
| 39 | 17535808 | NA | Gdi1 | guanosine diphosphate (GDP) dissociation inhibitor 1 | 14567 | ENSMUSG00000015291 |
| 40 | 17490452 | NA | Ap2a1 | adaptor protein complex AP-2, alpha 1 subunit | 11771 | ENSMUSG00000060279 |
| 41 | 17237915 | NA | Agap2 | ArfGAP with GTPase domain, ankyrin repeat and PH domain 2 | 216439 | ENSMUSG00000025422 |
| 42 | 17373530 | NA | Syt13 | synaptotagmin XIII | 80976 | ENSMUSG00000027220 |
| 43 | 17313106 | NA | Cacna1i | calcium channel, voltage-dependent, alpha 1I subunit | 239556 | ENSMUSG00000022416 |
| 44 | 17451140 | NA | Ulk1 | unc-51 like kinase 1 | 22241 | ENSMUSG00000029512 |
| 45 | 17279434 | NA | Pacs2 | phosphofurin acidic cluster sorting protein 2 | 217893 | ENSMUSG00000021143 |
| 46 | 17417702 | NA | Slc6a9 | solute carrier family 6 (neurotransmitter transporter, glycine), member 9 | 14664 | ENSMUSG00000028542 |
| 47 | 17477454 | NA | Syt3 | synaptotagmin III | 20981 | ENSMUSG00000030731 |
| 48 | 17452139 | NA | Rph3a | rabphilin 3A | 19894 | ENSMUSG00000029608 |
| 49 | 17337513 | NA | Gabbr1 | gamma-aminobutyric acid (GABA) B receptor, 1 | 54393 | ENSMUSG00000024462 |
| 50 | 17489052 | NA | Aplp1 | amyloid beta (A4) precursor-like protein 1 | 11803 | ENSMUSG00000006651 |
| 51 | 17432808 | NA | Mfn2 | mitofusin 2 | 170731 | ENSMUSG00000029020 |
| 52 | 17504130 | NA | Cx3cl1 | chemokine (C-X3-C motif) ligand 1 | 20312 | ENSMUSG00000031778 |
| 53 | 17468364 | NA | Rab11fip5 | RAB11 family interacting protein 5 (class I) | 52055 | ENSMUSG00000051343 |
| 54 | 17382496 | NA | Grin1 | glutamate receptor, ionotropic, NMDA1 (zeta 1) | 14810 | ENSMUSG00000026959 |
| 55 | 17367921 | NA | Grin1 | glutamate receptor, ionotropic, NMDA1 (zeta 1) | 14810 | ENSMUSG00000026959 |
| 56 | 17266107 | NA | Abr | active BCR-related gene | 109934 | ENSMUSG00000017631 |
| 57 | 17245539 | NA | Srgap1 | SLIT-ROBO Rho GTPase activating protein 1 | 117600 | ENSMUSG00000020121 |
| 58 | 17347948 | NA | Kcnk12 | potassium channel, subfamily K, member 12 | 210741 | ENSMUSG00000050138 |
| 59 | 17334205 | NA | Abca3 | ATP-binding cassette, sub-family A (ABC1), member 3 | 27410 | ENSMUSG00000024130 |
| 60 | 17335204 | NA | Anks1 | ankyrin repeat and SAM domain containing 1 | 224650 | ENSMUSG00000024219 |
| 61 | 17376167 | NA | Sirpa | signal-regulatory protein alpha | 19261 | ENSMUSG00000037902 |
| 62 | 17446123 | NA | Kcnh2 | potassium voltage-gated channel, subfamily H (eag-related), member 2 | 16511 | ENSMUSG00000038319 |
| 63 | 17319207 | NA | Csnk1e | casein kinase 1, epsilon | 27373 | ENSMUSG00000022433 |
| 64 | 17269464 | NA | Jup | junction plakoglobin | 16480 | ENSMUSG00000001552 |
| 65 | 17404570 | NA | Slc7a14 | solute carrier family 7 (cationic amino acid transporter, y+ system), member 14 | 241919 | ENSMUSG00000069072 |
| 66 | 17211347 | NA | Tfap2b | transcription factor AP-2 beta | 21419 | ENSMUSG00000025927 |
| 67 | 17406760 | NA | Sema4a | sema domain, immunoglobulin domain (Ig), transmembrane domain (TM) and short cytoplasmic domain, (semaphorin) 4A | 20351 | ENSMUSG00000028064 |
| 68 | 17385374 | NA | Nr4a2 | nuclear receptor subfamily 4, group A, member 2 | 18227 | ENSMUSG00000026826 |
| 69 | 17222625 | NA | Tgfbrap1 | transforming growth factor, beta receptor associated protein 1 | 73122 | ENSMUSG00000070939 |
| 70 | 17354299 | NA | Sema6a | sema domain, transmembrane domain (TM), and cytoplasmic domain, (semaphorin) 6A | 20358 | ENSMUSG00000019647 |
| 71 | 17435528 | NA | Dpp6 | dipeptidylpeptidase 6 | 13483 | ENSMUSG00000061576 |
| 72 | 17535607 | NA | Slc6a8 | solute carrier family 6 (neurotransmitter transporter, creatine), member 8 | 102857 | ENSMUSG00000019558 |
| 73 | 17407124 | NA | Chrnb2 | cholinergic receptor, nicotinic, beta polypeptide 2 (neuronal) | 11444 | ENSMUSG00000027950 |
| 74 | 17500005 | NA | Ank1 | ankyrin 1, erythroid | 11733 | ENSMUSG00000031543 |
| 75 | 17369305 | NA | Usp20 | ubiquitin specific peptidase 20 | 74270 | ENSMUSG00000026854 |
| 76 | 17354653 | NA | Tcof1 | Treacher Collins Franceschetti syndrome 1, homolog | 21453 | ENSMUSG00000024613 |
| 77 | 17361454 | NA | Cnih2 | cornichon homolog 2 (Drosophila) | 12794 | ENSMUSG00000024873 |
| 78 | 17335145 | NA | Pacsin1 | protein kinase C and casein kinase substrate in neurons 1 | 23969 | ENSMUSG00000040276 |
| 79 | 17243229 | NA | Atcay | ataxia, cerebellar, Cayman type homolog (human) | 16467 | ENSMUSG00000034958 |
| 80 | 17357486 | NA | Syt7 | synaptotagmin VII | 54525 | ENSMUSG00000024743 |
| 81 | 17498607 | NA | Mcoln1 | mucolipin 1 | 94178 | ENSMUSG00000004567 |
| 82 | 17249811 | NA | Slc36a1 | solute carrier family 36 (proton/amino acid symporter), member 1 | 215335 | ENSMUSG00000020261 |
| 83 | 17503333 | NA | Tnpo2 | transportin 2 (importin 3, karyopherin beta 2b) | 212999 | ENSMUSG00000031691 |
| 84 | 17317904 | NA | Slc45a4 | solute carrier family 45, member 4 | 106068 | ENSMUSG00000079020 |
| 85 | 17503884 | NA | Gnao1 | guanine nucleotide binding protein, alpha O | 14681 | ENSMUSG00000031748 |
| 86 | 17383858 | NA | Dnm1 | dynamin 1 | 13429 | ENSMUSG00000026825 |
| 87 | 17364665 | NA | Slit1 | slit homolog 1 (Drosophila) | 20562 | ENSMUSG00000025020 |
| 88 | 17224500 | NA | Atg9a | autophagy related 9A | 245860 | ENSMUSG00000033124 |
| 89 | 17256579 | NA | Cntnap1 | contactin associated protein-like 1 | 53321 | ENSMUSG00000017167 |
| 90 | 17497904 | NA | Slc25a22 | solute carrier family 25 (mitochondrial carrier, glutamate), member 22 | 68267 | ENSMUSG00000019082 |
| 91 | 17356924 | NA | Nrxn2 | neurexin II | 18190 | ENSMUSG00000033768 |
| 92 | 17234936 | NA | Hcn2 | hyperpolarization-activated, cyclic nucleotide-gated K+ 2 | 15166 | ENSMUSG00000020331 |
| 93 | 17313504 | NA | Srebf2 | sterol regulatory element binding factor 2 | 20788 | ENSMUSG00000022463 |
| 94 | 17267039 | NA | Lhx1 | LIM homeobox protein 1 | 16869 | ENSMUSG00000018698 |
| 95 | 17515843 | NA | Kirrel3 | kin of IRRE like 3 (Drosophila) | 67703 | ENSMUSG00000032036 |
| 96 | 17346427 | NA | Khsrp | KH-type splicing regulatory protein | 16549 | ENSMUSG00000007670 |
| 97 | 17516462 | NA | Thy1 | thymus cell antigen 1, theta | 21838 | ENSMUSG00000032011 |
| 98 | 17314051 | NA | Panx2 | pannexin 2 | 406218 | ENSMUSG00000058441 |
| 99 | 17461923 | NA | Syn2 | synapsin II | 20965 | ENSMUSG00000009394 |
| 100 | 17436545 | NA | Nat8l | N-acetyltransferase 8-like | 269642 | ENSMUSG00000048142 |
| 101 | 17388406 | NA | Slc35c1 | solute carrier family 35, member C1 | 228368 | ENSMUSG00000049922 |
| 102 | 17529218 | NA | Htr1b | 5-hydroxytryptamine (serotonin) receptor 1B | 15551 | ENSMUSG00000049511 |
| 103 | 17284065 | NA | Cdc42bpb | CDC42 binding protein kinase beta | 217866 | ENSMUSG00000021279 |
| 104 | 17238605 | NA | Itga7 | integrin alpha 7 | 16404 | ENSMUSG00000025348 |
| 105 | 17314164 | NA | Mapk8ip2 | mitogen-activated protein kinase 8 interacting protein 2 | 60597 | ENSMUSG00000022619 |
| 106 | 17503122 | NA | Cacna1a | calcium channel, voltage-dependent, P/Q type, alpha 1A subunit | 12286 | ENSMUSG00000034656 |
| 107 | 17487884 | NA | Gsk3a | glycogen synthase kinase 3 alpha | 606496 | ENSMUSG00000057177 |
| 108 | 17400521 | NA | Sv2a | synaptic vesicle glycoprotein 2 a | 64051 | ENSMUSG00000038486 |
| 109 | 17233306 | NA | Slc35f1 | solute carrier family 35, member F1 | 215085 | ENSMUSG00000038602 |
| 110 | 17263011 | NA | Glra1 | glycine receptor, alpha 1 subunit | 14654 | ENSMUSG00000000263 |
| 111 | 17517073 | NA | Drd2 | dopamine receptor D2 | 13489 | ENSMUSG00000032259 |
| 112 | 17461897 | NA | Atg7 | autophagy related 7 | 74244 | ENSMUSG00000030314 |
| 113 | 17536720 | NA | Nlgn3 | neuroligin 3 | 245537 | ENSMUSG00000031302 |
| 114 | 17478181 | NA | Kcnc1 | potassium voltage gated channel, Shaw-related subfamily, member 1 | 16502 | ENSMUSG00000058975 |
| 115 | 17271158 | NA | Cacng5 | calcium channel, voltage-dependent, gamma subunit 5 | 140723 | ENSMUSG00000040373 |
| 116 | 17332495 | NA | Dscam | Down syndrome cell adhesion molecule | 13508 | ENSMUSG00000050272 |
| 117 | 17406925 | NA | Hcn3 | hyperpolarization-activated, cyclic nucleotide-gated K+ 3 | 15168 | ENSMUSG00000028051 |
| 118 | 17473161 | NA | Cacng8 | calcium channel, voltage-dependent, gamma subunit 8 | 81905 | ENSMUSG00000053395 |
| 119 | 17496763 | NA | Stx1b | syntaxin 1B | 56216 | ENSMUSG00000030806 |
| 120 | 17259344 | NA | Hgs | HGF-regulated tyrosine kinase substrate | 15239 | ENSMUSG00000025793 |
| 121 | 17441453 | NA | Nos1 | nitric oxide synthase 1, neuronal | 18125 | ENSMUSG00000029361 |
| 122 | 17257197 | NA | Mapt | microtubule-associated protein tau | 17762 | ENSMUSG00000018411 |
| 123 | 17409005 | NA | Slc6a17 | solute carrier family 6 (neurotransmitter transporter), member 17 | 229706 | ENSMUSG00000027894 |
| 124 | 17235300 | NA | Apc2 | adenomatosis polyposis coli 2 | 23805 | ENSMUSG00000020135 |
| 125 | 17454574 | NA | Ttyh3 | tweety homolog 3 (Drosophila) | 78339 | ENSMUSG00000036565 |
| 126 | 17523281 | NA | Trak1 | trafficking protein, kinesin binding 1 | 67095 | ENSMUSG00000032536 |
| 127 | 17314636 | NA | Cacnb3 | calcium channel, voltage-dependent, beta 3 subunit | 12297 | ENSMUSG00000003352 |
| 128 | 17237336 | NA | Kcnc2 | potassium voltage gated channel, Shaw-related subfamily, member 2 | 268345 | ENSMUSG00000035681 |
| 129 | 17394079 | NA | Rims4 | regulating synaptic membrane exocytosis 4 | 241770 | ENSMUSG00000035226 |
| 130 | 17384021 | NA | Stxbp1 | syntaxin binding protein 1 | 20910 | ENSMUSG00000026797 |
| 131 | 17308299 | NA | Slc39a14 | solute carrier family 39 (zinc transporter), member 14 | 213053 | ENSMUSG00000022094 |
| 132 | 17369147 | NA | Nup188 | nucleoporin 188 | 227699 | ENSMUSG00000052533 |
| 133 | 17535627 | NA | Abcd1 | ATP-binding cassette, sub-family D (ALD), member 1 | 11666 | ENSMUSG00000031378 |
| 134 | 17530733 | NA | Grm2 | glutamate receptor, metabotropic 2 | 108068 | ENSMUSG00000023192 |
| 135 | 17237937 | NA | B4galnt1 | beta-1,4-N-acetyl-galactosaminyl transferase 1 | 14421 | ENSMUSG00000006731 |
| 136 | 17474067 | NA | Slc8a2 | solute carrier family 8 (sodium/calcium exchanger), member 2 | 110891 | ENSMUSG00000030376 |
| 137 | 17465620 | NA | Podxl | podocalyxin-like | 27205 | ENSMUSG00000025608 |
| 138 | 17357959 | NA | Gnaq | guanine nucleotide binding protein, alpha q polypeptide | 14682 | ENSMUSG00000024639 |
| 139 | 17377144 | NA | Slc24a3 | solute carrier family 24 (sodium/potassium/calcium exchanger), member 3 | 94249 | ENSMUSG00000063873 |
| 140 | 17451482 | NA | Svop | SV2 related protein | 68666 | ENSMUSG00000042078 |
| 141 | 17243057 | NA | Ap3d1 | adaptor-related protein complex 3, delta 1 subunit | 11776 | ENSMUSG00000020198 |
| 142 | 17419587 | NA | Slc9a1 | solute carrier family 9 (sodium/hydrogen exchanger), member 1 | 20544 | ENSMUSG00000028854 |
| 143 | 17361779 | NA | Scyl1 | SCY1-like 1 (S. cerevisiae) | 78891 | ENSMUSG00000024941 |
| 144 | 17370883 | NA | Kcnj3 | potassium inwardly-rectifying channel, subfamily J, member 3 | 16519 | ENSMUSG00000026824 |
| 145 | 17235663 | NA | Pip5k1c | phosphatidylinositol-4-phosphate 5-kinase, type 1 gamma | 18717 | ENSMUSG00000034902 |
| 146 | 17378721 | NA | Src | Rous sarcoma oncogene | 20779 | ENSMUSG00000027646 |
| 147 | 17517634 | NA | Sin3a | transcriptional regulator, SIN3A (yeast) | 20466 | ENSMUSG00000042557 |
| 148 | 17452719 | NA | Abcb9 | ATP-binding cassette, sub-family B (MDR/TAP), member 9 | 56325 | ENSMUSG00000029408 |
| 149 | 17500301 | NA | Gpr124 | G protein-coupled receptor 124 | 78560 | ENSMUSG00000031486 |
| 150 | 17471155 | NA | Kcna1 | potassium voltage-gated channel, shaker-related subfamily, member 1 | 16485 | ENSMUSG00000047976 |
| 151 | 17450059 | NA | Sec31a | Sec31 homolog A (S. cerevisiae) | 69162 | ENSMUSG00000035325 |
| 152 | 17461868 | NA | Slc6a1 | solute carrier family 6 (neurotransmitter transporter, GABA), member 1 | 232333 | ENSMUSG00000030310 |
| 153 | 17415863 | NA | Cachd1 | cache domain containing 1 | 320508 | ENSMUSG00000028532 |
| 154 | 17441595 | NA | Tbx3 | T-box 3 | 21386 | ENSMUSG00000018604 |
| 155 | 17490312 | NA | Nr1h2 | nuclear receptor subfamily 1, group H, member 2 | 22260 | ENSMUSG00000060601 |
| 156 | 17222925 | NA | Mfsd6 | major facilitator superfamily domain containing 6 | 98682 | ENSMUSG00000041439 |
| 157 | 17297462 | NA | Sec24c | Sec24 related gene family, member C (S. cerevisiae) | 218811 | ENSMUSG00000039367 |
| 158 | 17361470 | NA | Klc2 | kinesin light chain 2 | 16594 | ENSMUSG00000024862 |
| 159 | 17359583 | NA | Cnnm1 | cyclin M1 | 83674 | ENSMUSG00000025189 |
| 160 | 17336987 | NA | Bag6 | BCL2-associated athanogene 6 | 224727 | ENSMUSG00000024392 |
| 161 | 17433287 | NA | Slc45a1 | solute carrier family 45, member 1 | 242773 | ENSMUSG00000039838 |
| 162 | 17516731 | NA | Scn2b | sodium channel, voltage-gated, type II, beta | 72821 | ENSMUSG00000070304 |
| 163 | 17533640 | NA | Cdk16 | cyclin-dependent kinase 16 | 18555 | ENSMUSG00000031065 |
| 164 | 17360440 | NA | Adra2a | adrenergic receptor, alpha 2a | 11551 | ENSMUSG00000033717 |
| 165 | 17260261 | NA | Camk2b | calcium/calmodulin-dependent protein kinase II, beta | 12323 | ENSMUSG00000057897 |
| 166 | 17322359 | NA | Zfp385a | zinc finger protein 385A | 29813 | ENSMUSG00000000552 |

  
  

| **Database:biological process      &nbspName:transport      &nbspID:GO:0006810** | | | | | | |
| --- | --- | --- | --- | --- | --- | --- |
| C=3144; O=142; E=70.13; R=2.02; rawP=1.00e-17; adjP=4.77e-15 | | | | | | |
| Index | UserID | Value | Gene Symbol | Gene Name | EntrezGene | Ensembl |
| 1 | 17318923 | NA | Cacng2 | calcium channel, voltage-dependent, gamma subunit 2 | 12300 | ENSMUSG00000019146 |
| 2 | 17378922 | NA | Slc32a1 | solute carrier family 32 (GABA vesicular transporter), member 1 | 22348 | ENSMUSG00000037771 |
| 3 | 17334545 | NA | Clcn7 | chloride channel 7 | 26373 | ENSMUSG00000036636 |
| 4 | 17461852 | NA | Slc6a11 | solute carrier family 6 (neurotransmitter transporter, GABA), member 11 | 243616 | ENSMUSG00000030307 |
| 5 | 17243717 | NA | Syn3 | synapsin III | 27204 | ENSMUSG00000059602 |
| 6 | 17399496 | NA | Adar | adenosine deaminase, RNA-specific | 56417 | ENSMUSG00000027951 |
| 7 | 17376685 | NA | Plcb1 | phospholipase C, beta 1 | 18795 | ENSMUSG00000051177 |
| 8 | 17329516 | NA | Fgf12 | fibroblast growth factor 12 | 14167 | ENSMUSG00000022523 |
| 9 | 17342065 | NA | Mapk8ip3 | mitogen-activated protein kinase 8 interacting protein 3 | 30957 | ENSMUSG00000024163 |
| 10 | 17226891 | NA | Cntn2 | contactin 2 | 21367 | ENSMUSG00000053024 |
| 11 | 17252995 | NA | Slc43a2 | solute carrier family 43, member 2 | 215113 | ENSMUSG00000038178 |
| 12 | 17322600 | NA | Mgrn1 | mahogunin, ring finger 1 | 17237 | ENSMUSG00000022517 |
| 13 | 17370234 | NA | Dab2ip | disabled 2 interacting protein | 69601 | ENSMUSG00000026883 |
| 14 | 17361223 | NA | Adrbk1 | adrenergic receptor kinase, beta 1 | 110355 | ENSMUSG00000024858 |
| 15 | 17469814 | NA | Atp2b2 | ATPase, Ca++ transporting, plasma membrane 2 | 11941 | ENSMUSG00000030302 |
| 16 | 17401394 | NA | Kcnd3 | potassium voltage-gated channel, Shal-related family, member 3 | 56543 | ENSMUSG00000040896 |
| 17 | 17487805 | NA | Atp1a3 | ATPase, Na+/K+ transporting, alpha 3 polypeptide | 232975 | ENSMUSG00000040907 |
| 18 | 17502039 | NA | Rab3a | RAB3A, member RAS oncogene family | 19339 | ENSMUSG00000031840 |
| 19 | 17307905 | NA | Dpysl2 | dihydropyrimidinase-like 2 | 12934 | ENSMUSG00000022048 |
| 20 | 17290259 | NA | Ucn3 | urocortin 3 | 83428 | ENSMUSG00000044988 |
| 21 | 17526175 | NA | Abcg4 | ATP-binding cassette, sub-family G (WHITE), member 4 | 192663 | ENSMUSG00000032131 |
| 22 | 17540501 | NA | Syn1 | synapsin I | 20964 | ENSMUSG00000037217 |
| 23 | 17298874 | NA | Grid1 | glutamate receptor, ionotropic, delta 1 | 14803 | ENSMUSG00000041078 |
| 24 | 17521327 | NA | Cacna2d2 | calcium channel, voltage-dependent, alpha 2/delta subunit 2 | 56808 | ENSMUSG00000010066 |
| 25 | 17473155 | NA | Cacng7 | calcium channel, voltage-dependent, gamma subunit 7 | 81904 | ENSMUSG00000069806 |
| 26 | 17501692 | NA | Atp13a1 | ATPase type 13A1 | 170759 | ENSMUSG00000031862 |
| 27 | 17477714 | NA | Slc17a7 | solute carrier family 17 (sodium-dependent inorganic phosphate cotransporter), member 7 | 72961 | ENSMUSG00000070570 |
| 28 | 17361494 | NA | Pacs1 | phosphofurin acidic cluster sorting protein 1 | 107975 | ENSMUSG00000024855 |
| 29 | 17317801 | NA | Kcnk9 | potassium channel, subfamily K, member 9 | 223604 | ENSMUSG00000036760 |
| 30 | 17243162 | NA | Slc39a3 | solute carrier family 39 (zinc transporter), member 3 | 106947 | ENSMUSG00000046822 |
| 31 | 17527520 | NA | Scamp5 | secretory carrier membrane protein 5 | 56807 | ENSMUSG00000040722 |
| 32 | 17269439 | NA | Hap1 | huntingtin-associated protein 1 | 15114 | ENSMUSG00000006930 |
| 33 | 17532879 | NA | Syp | synaptophysin | 20977 | ENSMUSG00000031144 |
| 34 | 17535808 | NA | Gdi1 | guanosine diphosphate (GDP) dissociation inhibitor 1 | 14567 | ENSMUSG00000015291 |
| 35 | 17490452 | NA | Ap2a1 | adaptor protein complex AP-2, alpha 1 subunit | 11771 | ENSMUSG00000060279 |
| 36 | 17237915 | NA | Agap2 | ArfGAP with GTPase domain, ankyrin repeat and PH domain 2 | 216439 | ENSMUSG00000025422 |
| 37 | 17373530 | NA | Syt13 | synaptotagmin XIII | 80976 | ENSMUSG00000027220 |
| 38 | 17313106 | NA | Cacna1i | calcium channel, voltage-dependent, alpha 1I subunit | 239556 | ENSMUSG00000022416 |
| 39 | 17451140 | NA | Ulk1 | unc-51 like kinase 1 | 22241 | ENSMUSG00000029512 |
| 40 | 17279434 | NA | Pacs2 | phosphofurin acidic cluster sorting protein 2 | 217893 | ENSMUSG00000021143 |
| 41 | 17417702 | NA | Slc6a9 | solute carrier family 6 (neurotransmitter transporter, glycine), member 9 | 14664 | ENSMUSG00000028542 |
| 42 | 17477454 | NA | Syt3 | synaptotagmin III | 20981 | ENSMUSG00000030731 |
| 43 | 17452139 | NA | Rph3a | rabphilin 3A | 19894 | ENSMUSG00000029608 |
| 44 | 17337513 | NA | Gabbr1 | gamma-aminobutyric acid (GABA) B receptor, 1 | 54393 | ENSMUSG00000024462 |
| 45 | 17489052 | NA | Aplp1 | amyloid beta (A4) precursor-like protein 1 | 11803 | ENSMUSG00000006651 |
| 46 | 17432808 | NA | Mfn2 | mitofusin 2 | 170731 | ENSMUSG00000029020 |
| 47 | 17504130 | NA | Cx3cl1 | chemokine (C-X3-C motif) ligand 1 | 20312 | ENSMUSG00000031778 |
| 48 | 17468364 | NA | Rab11fip5 | RAB11 family interacting protein 5 (class I) | 52055 | ENSMUSG00000051343 |
| 49 | 17382496 | NA | Grin1 | glutamate receptor, ionotropic, NMDA1 (zeta 1) | 14810 | ENSMUSG00000026959 |
| 50 | 17367921 | NA | Grin1 | glutamate receptor, ionotropic, NMDA1 (zeta 1) | 14810 | ENSMUSG00000026959 |
| 51 | 17266107 | NA | Abr | active BCR-related gene | 109934 | ENSMUSG00000017631 |
| 52 | 17347948 | NA | Kcnk12 | potassium channel, subfamily K, member 12 | 210741 | ENSMUSG00000050138 |
| 53 | 17334205 | NA | Abca3 | ATP-binding cassette, sub-family A (ABC1), member 3 | 27410 | ENSMUSG00000024130 |
| 54 | 17376167 | NA | Sirpa | signal-regulatory protein alpha | 19261 | ENSMUSG00000037902 |
| 55 | 17446123 | NA | Kcnh2 | potassium voltage-gated channel, subfamily H (eag-related), member 2 | 16511 | ENSMUSG00000038319 |
| 56 | 17269464 | NA | Jup | junction plakoglobin | 16480 | ENSMUSG00000001552 |
| 57 | 17404570 | NA | Slc7a14 | solute carrier family 7 (cationic amino acid transporter, y+ system), member 14 | 241919 | ENSMUSG00000069072 |
| 58 | 17211347 | NA | Tfap2b | transcription factor AP-2 beta | 21419 | ENSMUSG00000025927 |
| 59 | 17222625 | NA | Tgfbrap1 | transforming growth factor, beta receptor associated protein 1 | 73122 | ENSMUSG00000070939 |
| 60 | 17435528 | NA | Dpp6 | dipeptidylpeptidase 6 | 13483 | ENSMUSG00000061576 |
| 61 | 17535607 | NA | Slc6a8 | solute carrier family 6 (neurotransmitter transporter, creatine), member 8 | 102857 | ENSMUSG00000019558 |
| 62 | 17407124 | NA | Chrnb2 | cholinergic receptor, nicotinic, beta polypeptide 2 (neuronal) | 11444 | ENSMUSG00000027950 |
| 63 | 17500005 | NA | Ank1 | ankyrin 1, erythroid | 11733 | ENSMUSG00000031543 |
| 64 | 17369305 | NA | Usp20 | ubiquitin specific peptidase 20 | 74270 | ENSMUSG00000026854 |
| 65 | 17354653 | NA | Tcof1 | Treacher Collins Franceschetti syndrome 1, homolog | 21453 | ENSMUSG00000024613 |
| 66 | 17361454 | NA | Cnih2 | cornichon homolog 2 (Drosophila) | 12794 | ENSMUSG00000024873 |
| 67 | 17335145 | NA | Pacsin1 | protein kinase C and casein kinase substrate in neurons 1 | 23969 | ENSMUSG00000040276 |
| 68 | 17243229 | NA | Atcay | ataxia, cerebellar, Cayman type homolog (human) | 16467 | ENSMUSG00000034958 |
| 69 | 17357486 | NA | Syt7 | synaptotagmin VII | 54525 | ENSMUSG00000024743 |
| 70 | 17498607 | NA | Mcoln1 | mucolipin 1 | 94178 | ENSMUSG00000004567 |
| 71 | 17249811 | NA | Slc36a1 | solute carrier family 36 (proton/amino acid symporter), member 1 | 215335 | ENSMUSG00000020261 |
| 72 | 17503333 | NA | Tnpo2 | transportin 2 (importin 3, karyopherin beta 2b) | 212999 | ENSMUSG00000031691 |
| 73 | 17317904 | NA | Slc45a4 | solute carrier family 45, member 4 | 106068 | ENSMUSG00000079020 |
| 74 | 17503884 | NA | Gnao1 | guanine nucleotide binding protein, alpha O | 14681 | ENSMUSG00000031748 |
| 75 | 17383858 | NA | Dnm1 | dynamin 1 | 13429 | ENSMUSG00000026825 |
| 76 | 17256579 | NA | Cntnap1 | contactin associated protein-like 1 | 53321 | ENSMUSG00000017167 |
| 77 | 17497904 | NA | Slc25a22 | solute carrier family 25 (mitochondrial carrier, glutamate), member 22 | 68267 | ENSMUSG00000019082 |
| 78 | 17356924 | NA | Nrxn2 | neurexin II | 18190 | ENSMUSG00000033768 |
| 79 | 17234936 | NA | Hcn2 | hyperpolarization-activated, cyclic nucleotide-gated K+ 2 | 15166 | ENSMUSG00000020331 |
| 80 | 17313504 | NA | Srebf2 | sterol regulatory element binding factor 2 | 20788 | ENSMUSG00000022463 |
| 81 | 17346427 | NA | Khsrp | KH-type splicing regulatory protein | 16549 | ENSMUSG00000007670 |
| 82 | 17461923 | NA | Syn2 | synapsin II | 20965 | ENSMUSG00000009394 |
| 83 | 17314051 | NA | Panx2 | pannexin 2 | 406218 | ENSMUSG00000058441 |
| 84 | 17516462 | NA | Thy1 | thymus cell antigen 1, theta | 21838 | ENSMUSG00000032011 |
| 85 | 17436545 | NA | Nat8l | N-acetyltransferase 8-like | 269642 | ENSMUSG00000048142 |
| 86 | 17529218 | NA | Htr1b | 5-hydroxytryptamine (serotonin) receptor 1B | 15551 | ENSMUSG00000049511 |
| 87 | 17388406 | NA | Slc35c1 | solute carrier family 35, member C1 | 228368 | ENSMUSG00000049922 |
| 88 | 17314164 | NA | Mapk8ip2 | mitogen-activated protein kinase 8 interacting protein 2 | 60597 | ENSMUSG00000022619 |
| 89 | 17503122 | NA | Cacna1a | calcium channel, voltage-dependent, P/Q type, alpha 1A subunit | 12286 | ENSMUSG00000034656 |
| 90 | 17487884 | NA | Gsk3a | glycogen synthase kinase 3 alpha | 606496 | ENSMUSG00000057177 |
| 91 | 17400521 | NA | Sv2a | synaptic vesicle glycoprotein 2 a | 64051 | ENSMUSG00000038486 |
| 92 | 17233306 | NA | Slc35f1 | solute carrier family 35, member F1 | 215085 | ENSMUSG00000038602 |
| 93 | 17263011 | NA | Glra1 | glycine receptor, alpha 1 subunit | 14654 | ENSMUSG00000000263 |
| 94 | 17517073 | NA | Drd2 | dopamine receptor D2 | 13489 | ENSMUSG00000032259 |
| 95 | 17461897 | NA | Atg7 | autophagy related 7 | 74244 | ENSMUSG00000030314 |
| 96 | 17536720 | NA | Nlgn3 | neuroligin 3 | 245537 | ENSMUSG00000031302 |
| 97 | 17478181 | NA | Kcnc1 | potassium voltage gated channel, Shaw-related subfamily, member 1 | 16502 | ENSMUSG00000058975 |
| 98 | 17271158 | NA | Cacng5 | calcium channel, voltage-dependent, gamma subunit 5 | 140723 | ENSMUSG00000040373 |
| 99 | 17406925 | NA | Hcn3 | hyperpolarization-activated, cyclic nucleotide-gated K+ 3 | 15168 | ENSMUSG00000028051 |
| 100 | 17473161 | NA | Cacng8 | calcium channel, voltage-dependent, gamma subunit 8 | 81905 | ENSMUSG00000053395 |
| 101 | 17259344 | NA | Hgs | HGF-regulated tyrosine kinase substrate | 15239 | ENSMUSG00000025793 |
| 102 | 17496763 | NA | Stx1b | syntaxin 1B | 56216 | ENSMUSG00000030806 |
| 103 | 17441453 | NA | Nos1 | nitric oxide synthase 1, neuronal | 18125 | ENSMUSG00000029361 |
| 104 | 17257197 | NA | Mapt | microtubule-associated protein tau | 17762 | ENSMUSG00000018411 |
| 105 | 17409005 | NA | Slc6a17 | solute carrier family 6 (neurotransmitter transporter), member 17 | 229706 | ENSMUSG00000027894 |
| 106 | 17235300 | NA | Apc2 | adenomatosis polyposis coli 2 | 23805 | ENSMUSG00000020135 |
| 107 | 17454574 | NA | Ttyh3 | tweety homolog 3 (Drosophila) | 78339 | ENSMUSG00000036565 |
| 108 | 17523281 | NA | Trak1 | trafficking protein, kinesin binding 1 | 67095 | ENSMUSG00000032536 |
| 109 | 17314636 | NA | Cacnb3 | calcium channel, voltage-dependent, beta 3 subunit | 12297 | ENSMUSG00000003352 |
| 110 | 17237336 | NA | Kcnc2 | potassium voltage gated channel, Shaw-related subfamily, member 2 | 268345 | ENSMUSG00000035681 |
| 111 | 17394079 | NA | Rims4 | regulating synaptic membrane exocytosis 4 | 241770 | ENSMUSG00000035226 |
| 112 | 17384021 | NA | Stxbp1 | syntaxin binding protein 1 | 20910 | ENSMUSG00000026797 |
| 113 | 17308299 | NA | Slc39a14 | solute carrier family 39 (zinc transporter), member 14 | 213053 | ENSMUSG00000022094 |
| 114 | 17369147 | NA | Nup188 | nucleoporin 188 | 227699 | ENSMUSG00000052533 |
| 115 | 17535627 | NA | Abcd1 | ATP-binding cassette, sub-family D (ALD), member 1 | 11666 | ENSMUSG00000031378 |
| 116 | 17530733 | NA | Grm2 | glutamate receptor, metabotropic 2 | 108068 | ENSMUSG00000023192 |
| 117 | 17474067 | NA | Slc8a2 | solute carrier family 8 (sodium/calcium exchanger), member 2 | 110891 | ENSMUSG00000030376 |
| 118 | 17357959 | NA | Gnaq | guanine nucleotide binding protein, alpha q polypeptide | 14682 | ENSMUSG00000024639 |
| 119 | 17377144 | NA | Slc24a3 | solute carrier family 24 (sodium/potassium/calcium exchanger), member 3 | 94249 | ENSMUSG00000063873 |
| 120 | 17451482 | NA | Svop | SV2 related protein | 68666 | ENSMUSG00000042078 |
| 121 | 17243057 | NA | Ap3d1 | adaptor-related protein complex 3, delta 1 subunit | 11776 | ENSMUSG00000020198 |
| 122 | 17419587 | NA | Slc9a1 | solute carrier family 9 (sodium/hydrogen exchanger), member 1 | 20544 | ENSMUSG00000028854 |
| 123 | 17361779 | NA | Scyl1 | SCY1-like 1 (S. cerevisiae) | 78891 | ENSMUSG00000024941 |
| 124 | 17370883 | NA | Kcnj3 | potassium inwardly-rectifying channel, subfamily J, member 3 | 16519 | ENSMUSG00000026824 |
| 125 | 17235663 | NA | Pip5k1c | phosphatidylinositol-4-phosphate 5-kinase, type 1 gamma | 18717 | ENSMUSG00000034902 |
| 126 | 17378721 | NA | Src | Rous sarcoma oncogene | 20779 | ENSMUSG00000027646 |
| 127 | 17452719 | NA | Abcb9 | ATP-binding cassette, sub-family B (MDR/TAP), member 9 | 56325 | ENSMUSG00000029408 |
| 128 | 17471155 | NA | Kcna1 | potassium voltage-gated channel, shaker-related subfamily, member 1 | 16485 | ENSMUSG00000047976 |
| 129 | 17450059 | NA | Sec31a | Sec31 homolog A (S. cerevisiae) | 69162 | ENSMUSG00000035325 |
| 130 | 17461868 | NA | Slc6a1 | solute carrier family 6 (neurotransmitter transporter, GABA), member 1 | 232333 | ENSMUSG00000030310 |
| 131 | 17415863 | NA | Cachd1 | cache domain containing 1 | 320508 | ENSMUSG00000028532 |
| 132 | 17441595 | NA | Tbx3 | T-box 3 | 21386 | ENSMUSG00000018604 |
| 133 | 17490312 | NA | Nr1h2 | nuclear receptor subfamily 1, group H, member 2 | 22260 | ENSMUSG00000060601 |
| 134 | 17222925 | NA | Mfsd6 | major facilitator superfamily domain containing 6 | 98682 | ENSMUSG00000041439 |
| 135 | 17297462 | NA | Sec24c | Sec24 related gene family, member C (S. cerevisiae) | 218811 | ENSMUSG00000039367 |
| 136 | 17361470 | NA | Klc2 | kinesin light chain 2 | 16594 | ENSMUSG00000024862 |
| 137 | 17359583 | NA | Cnnm1 | cyclin M1 | 83674 | ENSMUSG00000025189 |
| 138 | 17336987 | NA | Bag6 | BCL2-associated athanogene 6 | 224727 | ENSMUSG00000024392 |
| 139 | 17433287 | NA | Slc45a1 | solute carrier family 45, member 1 | 242773 | ENSMUSG00000039838 |
| 140 | 17516731 | NA | Scn2b | sodium channel, voltage-gated, type II, beta | 72821 | ENSMUSG00000070304 |
| 141 | 17533640 | NA | Cdk16 | cyclin-dependent kinase 16 | 18555 | ENSMUSG00000031065 |
| 142 | 17360440 | NA | Adra2a | adrenergic receptor, alpha 2a | 11551 | ENSMUSG00000033717 |
| 143 | 17260261 | NA | Camk2b | calcium/calmodulin-dependent protein kinase II, beta | 12323 | ENSMUSG00000057897 |

  
  

| **Database:biological process      &nbspName:establishment of localization      &nbspID:GO:0051234** | | | | | | |
| --- | --- | --- | --- | --- | --- | --- |
| C=3199; O=143; E=71.36; R=2.00; rawP=1.81e-17; adjP=6.90e-15 | | | | | | |
| Index | UserID | Value | Gene Symbol | Gene Name | EntrezGene | Ensembl |
| 1 | 17318923 | NA | Cacng2 | calcium channel, voltage-dependent, gamma subunit 2 | 12300 | ENSMUSG00000019146 |
| 2 | 17378922 | NA | Slc32a1 | solute carrier family 32 (GABA vesicular transporter), member 1 | 22348 | ENSMUSG00000037771 |
| 3 | 17334545 | NA | Clcn7 | chloride channel 7 | 26373 | ENSMUSG00000036636 |
| 4 | 17461852 | NA | Slc6a11 | solute carrier family 6 (neurotransmitter transporter, GABA), member 11 | 243616 | ENSMUSG00000030307 |
| 5 | 17243717 | NA | Syn3 | synapsin III | 27204 | ENSMUSG00000059602 |
| 6 | 17399496 | NA | Adar | adenosine deaminase, RNA-specific | 56417 | ENSMUSG00000027951 |
| 7 | 17376685 | NA | Plcb1 | phospholipase C, beta 1 | 18795 | ENSMUSG00000051177 |
| 8 | 17329516 | NA | Fgf12 | fibroblast growth factor 12 | 14167 | ENSMUSG00000022523 |
| 9 | 17342065 | NA | Mapk8ip3 | mitogen-activated protein kinase 8 interacting protein 3 | 30957 | ENSMUSG00000024163 |
| 10 | 17226891 | NA | Cntn2 | contactin 2 | 21367 | ENSMUSG00000053024 |
| 11 | 17252995 | NA | Slc43a2 | solute carrier family 43, member 2 | 215113 | ENSMUSG00000038178 |
| 12 | 17322600 | NA | Mgrn1 | mahogunin, ring finger 1 | 17237 | ENSMUSG00000022517 |
| 13 | 17370234 | NA | Dab2ip | disabled 2 interacting protein | 69601 | ENSMUSG00000026883 |
| 14 | 17361223 | NA | Adrbk1 | adrenergic receptor kinase, beta 1 | 110355 | ENSMUSG00000024858 |
| 15 | 17469814 | NA | Atp2b2 | ATPase, Ca++ transporting, plasma membrane 2 | 11941 | ENSMUSG00000030302 |
| 16 | 17401394 | NA | Kcnd3 | potassium voltage-gated channel, Shal-related family, member 3 | 56543 | ENSMUSG00000040896 |
| 17 | 17487805 | NA | Atp1a3 | ATPase, Na+/K+ transporting, alpha 3 polypeptide | 232975 | ENSMUSG00000040907 |
| 18 | 17502039 | NA | Rab3a | RAB3A, member RAS oncogene family | 19339 | ENSMUSG00000031840 |
| 19 | 17307905 | NA | Dpysl2 | dihydropyrimidinase-like 2 | 12934 | ENSMUSG00000022048 |
| 20 | 17290259 | NA | Ucn3 | urocortin 3 | 83428 | ENSMUSG00000044988 |
| 21 | 17526175 | NA | Abcg4 | ATP-binding cassette, sub-family G (WHITE), member 4 | 192663 | ENSMUSG00000032131 |
| 22 | 17540501 | NA | Syn1 | synapsin I | 20964 | ENSMUSG00000037217 |
| 23 | 17298874 | NA | Grid1 | glutamate receptor, ionotropic, delta 1 | 14803 | ENSMUSG00000041078 |
| 24 | 17521327 | NA | Cacna2d2 | calcium channel, voltage-dependent, alpha 2/delta subunit 2 | 56808 | ENSMUSG00000010066 |
| 25 | 17473155 | NA | Cacng7 | calcium channel, voltage-dependent, gamma subunit 7 | 81904 | ENSMUSG00000069806 |
| 26 | 17501692 | NA | Atp13a1 | ATPase type 13A1 | 170759 | ENSMUSG00000031862 |
| 27 | 17477714 | NA | Slc17a7 | solute carrier family 17 (sodium-dependent inorganic phosphate cotransporter), member 7 | 72961 | ENSMUSG00000070570 |
| 28 | 17361494 | NA | Pacs1 | phosphofurin acidic cluster sorting protein 1 | 107975 | ENSMUSG00000024855 |
| 29 | 17317801 | NA | Kcnk9 | potassium channel, subfamily K, member 9 | 223604 | ENSMUSG00000036760 |
| 30 | 17243162 | NA | Slc39a3 | solute carrier family 39 (zinc transporter), member 3 | 106947 | ENSMUSG00000046822 |
| 31 | 17527520 | NA | Scamp5 | secretory carrier membrane protein 5 | 56807 | ENSMUSG00000040722 |
| 32 | 17269439 | NA | Hap1 | huntingtin-associated protein 1 | 15114 | ENSMUSG00000006930 |
| 33 | 17532879 | NA | Syp | synaptophysin | 20977 | ENSMUSG00000031144 |
| 34 | 17535808 | NA | Gdi1 | guanosine diphosphate (GDP) dissociation inhibitor 1 | 14567 | ENSMUSG00000015291 |
| 35 | 17490452 | NA | Ap2a1 | adaptor protein complex AP-2, alpha 1 subunit | 11771 | ENSMUSG00000060279 |
| 36 | 17237915 | NA | Agap2 | ArfGAP with GTPase domain, ankyrin repeat and PH domain 2 | 216439 | ENSMUSG00000025422 |
| 37 | 17373530 | NA | Syt13 | synaptotagmin XIII | 80976 | ENSMUSG00000027220 |
| 38 | 17313106 | NA | Cacna1i | calcium channel, voltage-dependent, alpha 1I subunit | 239556 | ENSMUSG00000022416 |
| 39 | 17451140 | NA | Ulk1 | unc-51 like kinase 1 | 22241 | ENSMUSG00000029512 |
| 40 | 17279434 | NA | Pacs2 | phosphofurin acidic cluster sorting protein 2 | 217893 | ENSMUSG00000021143 |
| 41 | 17417702 | NA | Slc6a9 | solute carrier family 6 (neurotransmitter transporter, glycine), member 9 | 14664 | ENSMUSG00000028542 |
| 42 | 17477454 | NA | Syt3 | synaptotagmin III | 20981 | ENSMUSG00000030731 |
| 43 | 17452139 | NA | Rph3a | rabphilin 3A | 19894 | ENSMUSG00000029608 |
| 44 | 17337513 | NA | Gabbr1 | gamma-aminobutyric acid (GABA) B receptor, 1 | 54393 | ENSMUSG00000024462 |
| 45 | 17489052 | NA | Aplp1 | amyloid beta (A4) precursor-like protein 1 | 11803 | ENSMUSG00000006651 |
| 46 | 17432808 | NA | Mfn2 | mitofusin 2 | 170731 | ENSMUSG00000029020 |
| 47 | 17504130 | NA | Cx3cl1 | chemokine (C-X3-C motif) ligand 1 | 20312 | ENSMUSG00000031778 |
| 48 | 17468364 | NA | Rab11fip5 | RAB11 family interacting protein 5 (class I) | 52055 | ENSMUSG00000051343 |
| 49 | 17382496 | NA | Grin1 | glutamate receptor, ionotropic, NMDA1 (zeta 1) | 14810 | ENSMUSG00000026959 |
| 50 | 17367921 | NA | Grin1 | glutamate receptor, ionotropic, NMDA1 (zeta 1) | 14810 | ENSMUSG00000026959 |
| 51 | 17266107 | NA | Abr | active BCR-related gene | 109934 | ENSMUSG00000017631 |
| 52 | 17347948 | NA | Kcnk12 | potassium channel, subfamily K, member 12 | 210741 | ENSMUSG00000050138 |
| 53 | 17334205 | NA | Abca3 | ATP-binding cassette, sub-family A (ABC1), member 3 | 27410 | ENSMUSG00000024130 |
| 54 | 17376167 | NA | Sirpa | signal-regulatory protein alpha | 19261 | ENSMUSG00000037902 |
| 55 | 17446123 | NA | Kcnh2 | potassium voltage-gated channel, subfamily H (eag-related), member 2 | 16511 | ENSMUSG00000038319 |
| 56 | 17269464 | NA | Jup | junction plakoglobin | 16480 | ENSMUSG00000001552 |
| 57 | 17404570 | NA | Slc7a14 | solute carrier family 7 (cationic amino acid transporter, y+ system), member 14 | 241919 | ENSMUSG00000069072 |
| 58 | 17211347 | NA | Tfap2b | transcription factor AP-2 beta | 21419 | ENSMUSG00000025927 |
| 59 | 17222625 | NA | Tgfbrap1 | transforming growth factor, beta receptor associated protein 1 | 73122 | ENSMUSG00000070939 |
| 60 | 17435528 | NA | Dpp6 | dipeptidylpeptidase 6 | 13483 | ENSMUSG00000061576 |
| 61 | 17535607 | NA | Slc6a8 | solute carrier family 6 (neurotransmitter transporter, creatine), member 8 | 102857 | ENSMUSG00000019558 |
| 62 | 17407124 | NA | Chrnb2 | cholinergic receptor, nicotinic, beta polypeptide 2 (neuronal) | 11444 | ENSMUSG00000027950 |
| 63 | 17500005 | NA | Ank1 | ankyrin 1, erythroid | 11733 | ENSMUSG00000031543 |
| 64 | 17369305 | NA | Usp20 | ubiquitin specific peptidase 20 | 74270 | ENSMUSG00000026854 |
| 65 | 17354653 | NA | Tcof1 | Treacher Collins Franceschetti syndrome 1, homolog | 21453 | ENSMUSG00000024613 |
| 66 | 17361454 | NA | Cnih2 | cornichon homolog 2 (Drosophila) | 12794 | ENSMUSG00000024873 |
| 67 | 17335145 | NA | Pacsin1 | protein kinase C and casein kinase substrate in neurons 1 | 23969 | ENSMUSG00000040276 |
| 68 | 17243229 | NA | Atcay | ataxia, cerebellar, Cayman type homolog (human) | 16467 | ENSMUSG00000034958 |
| 69 | 17357486 | NA | Syt7 | synaptotagmin VII | 54525 | ENSMUSG00000024743 |
| 70 | 17498607 | NA | Mcoln1 | mucolipin 1 | 94178 | ENSMUSG00000004567 |
| 71 | 17249811 | NA | Slc36a1 | solute carrier family 36 (proton/amino acid symporter), member 1 | 215335 | ENSMUSG00000020261 |
| 72 | 17503333 | NA | Tnpo2 | transportin 2 (importin 3, karyopherin beta 2b) | 212999 | ENSMUSG00000031691 |
| 73 | 17317904 | NA | Slc45a4 | solute carrier family 45, member 4 | 106068 | ENSMUSG00000079020 |
| 74 | 17503884 | NA | Gnao1 | guanine nucleotide binding protein, alpha O | 14681 | ENSMUSG00000031748 |
| 75 | 17383858 | NA | Dnm1 | dynamin 1 | 13429 | ENSMUSG00000026825 |
| 76 | 17364665 | NA | Slit1 | slit homolog 1 (Drosophila) | 20562 | ENSMUSG00000025020 |
| 77 | 17256579 | NA | Cntnap1 | contactin associated protein-like 1 | 53321 | ENSMUSG00000017167 |
| 78 | 17497904 | NA | Slc25a22 | solute carrier family 25 (mitochondrial carrier, glutamate), member 22 | 68267 | ENSMUSG00000019082 |
| 79 | 17356924 | NA | Nrxn2 | neurexin II | 18190 | ENSMUSG00000033768 |
| 80 | 17234936 | NA | Hcn2 | hyperpolarization-activated, cyclic nucleotide-gated K+ 2 | 15166 | ENSMUSG00000020331 |
| 81 | 17313504 | NA | Srebf2 | sterol regulatory element binding factor 2 | 20788 | ENSMUSG00000022463 |
| 82 | 17346427 | NA | Khsrp | KH-type splicing regulatory protein | 16549 | ENSMUSG00000007670 |
| 83 | 17461923 | NA | Syn2 | synapsin II | 20965 | ENSMUSG00000009394 |
| 84 | 17314051 | NA | Panx2 | pannexin 2 | 406218 | ENSMUSG00000058441 |
| 85 | 17516462 | NA | Thy1 | thymus cell antigen 1, theta | 21838 | ENSMUSG00000032011 |
| 86 | 17436545 | NA | Nat8l | N-acetyltransferase 8-like | 269642 | ENSMUSG00000048142 |
| 87 | 17529218 | NA | Htr1b | 5-hydroxytryptamine (serotonin) receptor 1B | 15551 | ENSMUSG00000049511 |
| 88 | 17388406 | NA | Slc35c1 | solute carrier family 35, member C1 | 228368 | ENSMUSG00000049922 |
| 89 | 17314164 | NA | Mapk8ip2 | mitogen-activated protein kinase 8 interacting protein 2 | 60597 | ENSMUSG00000022619 |
| 90 | 17503122 | NA | Cacna1a | calcium channel, voltage-dependent, P/Q type, alpha 1A subunit | 12286 | ENSMUSG00000034656 |
| 91 | 17487884 | NA | Gsk3a | glycogen synthase kinase 3 alpha | 606496 | ENSMUSG00000057177 |
| 92 | 17400521 | NA | Sv2a | synaptic vesicle glycoprotein 2 a | 64051 | ENSMUSG00000038486 |
| 93 | 17233306 | NA | Slc35f1 | solute carrier family 35, member F1 | 215085 | ENSMUSG00000038602 |
| 94 | 17263011 | NA | Glra1 | glycine receptor, alpha 1 subunit | 14654 | ENSMUSG00000000263 |
| 95 | 17517073 | NA | Drd2 | dopamine receptor D2 | 13489 | ENSMUSG00000032259 |
| 96 | 17461897 | NA | Atg7 | autophagy related 7 | 74244 | ENSMUSG00000030314 |
| 97 | 17536720 | NA | Nlgn3 | neuroligin 3 | 245537 | ENSMUSG00000031302 |
| 98 | 17478181 | NA | Kcnc1 | potassium voltage gated channel, Shaw-related subfamily, member 1 | 16502 | ENSMUSG00000058975 |
| 99 | 17271158 | NA | Cacng5 | calcium channel, voltage-dependent, gamma subunit 5 | 140723 | ENSMUSG00000040373 |
| 100 | 17406925 | NA | Hcn3 | hyperpolarization-activated, cyclic nucleotide-gated K+ 3 | 15168 | ENSMUSG00000028051 |
| 101 | 17473161 | NA | Cacng8 | calcium channel, voltage-dependent, gamma subunit 8 | 81905 | ENSMUSG00000053395 |
| 102 | 17259344 | NA | Hgs | HGF-regulated tyrosine kinase substrate | 15239 | ENSMUSG00000025793 |
| 103 | 17496763 | NA | Stx1b | syntaxin 1B | 56216 | ENSMUSG00000030806 |
| 104 | 17441453 | NA | Nos1 | nitric oxide synthase 1, neuronal | 18125 | ENSMUSG00000029361 |
| 105 | 17257197 | NA | Mapt | microtubule-associated protein tau | 17762 | ENSMUSG00000018411 |
| 106 | 17409005 | NA | Slc6a17 | solute carrier family 6 (neurotransmitter transporter), member 17 | 229706 | ENSMUSG00000027894 |
| 107 | 17235300 | NA | Apc2 | adenomatosis polyposis coli 2 | 23805 | ENSMUSG00000020135 |
| 108 | 17454574 | NA | Ttyh3 | tweety homolog 3 (Drosophila) | 78339 | ENSMUSG00000036565 |
| 109 | 17523281 | NA | Trak1 | trafficking protein, kinesin binding 1 | 67095 | ENSMUSG00000032536 |
| 110 | 17314636 | NA | Cacnb3 | calcium channel, voltage-dependent, beta 3 subunit | 12297 | ENSMUSG00000003352 |
| 111 | 17237336 | NA | Kcnc2 | potassium voltage gated channel, Shaw-related subfamily, member 2 | 268345 | ENSMUSG00000035681 |
| 112 | 17394079 | NA | Rims4 | regulating synaptic membrane exocytosis 4 | 241770 | ENSMUSG00000035226 |
| 113 | 17384021 | NA | Stxbp1 | syntaxin binding protein 1 | 20910 | ENSMUSG00000026797 |
| 114 | 17308299 | NA | Slc39a14 | solute carrier family 39 (zinc transporter), member 14 | 213053 | ENSMUSG00000022094 |
| 115 | 17369147 | NA | Nup188 | nucleoporin 188 | 227699 | ENSMUSG00000052533 |
| 116 | 17535627 | NA | Abcd1 | ATP-binding cassette, sub-family D (ALD), member 1 | 11666 | ENSMUSG00000031378 |
| 117 | 17530733 | NA | Grm2 | glutamate receptor, metabotropic 2 | 108068 | ENSMUSG00000023192 |
| 118 | 17474067 | NA | Slc8a2 | solute carrier family 8 (sodium/calcium exchanger), member 2 | 110891 | ENSMUSG00000030376 |
| 119 | 17357959 | NA | Gnaq | guanine nucleotide binding protein, alpha q polypeptide | 14682 | ENSMUSG00000024639 |
| 120 | 17377144 | NA | Slc24a3 | solute carrier family 24 (sodium/potassium/calcium exchanger), member 3 | 94249 | ENSMUSG00000063873 |
| 121 | 17451482 | NA | Svop | SV2 related protein | 68666 | ENSMUSG00000042078 |
| 122 | 17243057 | NA | Ap3d1 | adaptor-related protein complex 3, delta 1 subunit | 11776 | ENSMUSG00000020198 |
| 123 | 17419587 | NA | Slc9a1 | solute carrier family 9 (sodium/hydrogen exchanger), member 1 | 20544 | ENSMUSG00000028854 |
| 124 | 17361779 | NA | Scyl1 | SCY1-like 1 (S. cerevisiae) | 78891 | ENSMUSG00000024941 |
| 125 | 17370883 | NA | Kcnj3 | potassium inwardly-rectifying channel, subfamily J, member 3 | 16519 | ENSMUSG00000026824 |
| 126 | 17235663 | NA | Pip5k1c | phosphatidylinositol-4-phosphate 5-kinase, type 1 gamma | 18717 | ENSMUSG00000034902 |
| 127 | 17378721 | NA | Src | Rous sarcoma oncogene | 20779 | ENSMUSG00000027646 |
| 128 | 17452719 | NA | Abcb9 | ATP-binding cassette, sub-family B (MDR/TAP), member 9 | 56325 | ENSMUSG00000029408 |
| 129 | 17471155 | NA | Kcna1 | potassium voltage-gated channel, shaker-related subfamily, member 1 | 16485 | ENSMUSG00000047976 |
| 130 | 17450059 | NA | Sec31a | Sec31 homolog A (S. cerevisiae) | 69162 | ENSMUSG00000035325 |
| 131 | 17461868 | NA | Slc6a1 | solute carrier family 6 (neurotransmitter transporter, GABA), member 1 | 232333 | ENSMUSG00000030310 |
| 132 | 17415863 | NA | Cachd1 | cache domain containing 1 | 320508 | ENSMUSG00000028532 |
| 133 | 17441595 | NA | Tbx3 | T-box 3 | 21386 | ENSMUSG00000018604 |
| 134 | 17490312 | NA | Nr1h2 | nuclear receptor subfamily 1, group H, member 2 | 22260 | ENSMUSG00000060601 |
| 135 | 17222925 | NA | Mfsd6 | major facilitator superfamily domain containing 6 | 98682 | ENSMUSG00000041439 |
| 136 | 17297462 | NA | Sec24c | Sec24 related gene family, member C (S. cerevisiae) | 218811 | ENSMUSG00000039367 |
| 137 | 17361470 | NA | Klc2 | kinesin light chain 2 | 16594 | ENSMUSG00000024862 |
| 138 | 17359583 | NA | Cnnm1 | cyclin M1 | 83674 | ENSMUSG00000025189 |
| 139 | 17336987 | NA | Bag6 | BCL2-associated athanogene 6 | 224727 | ENSMUSG00000024392 |
| 140 | 17433287 | NA | Slc45a1 | solute carrier family 45, member 1 | 242773 | ENSMUSG00000039838 |
| 141 | 17516731 | NA | Scn2b | sodium channel, voltage-gated, type II, beta | 72821 | ENSMUSG00000070304 |
| 142 | 17533640 | NA | Cdk16 | cyclin-dependent kinase 16 | 18555 | ENSMUSG00000031065 |
| 143 | 17360440 | NA | Adra2a | adrenergic receptor, alpha 2a | 11551 | ENSMUSG00000033717 |
| 144 | 17260261 | NA | Camk2b | calcium/calmodulin-dependent protein kinase II, beta | 12323 | ENSMUSG00000057897 |

  
  

| **Database:biological process      &nbspName:transmission of nerve impulse      &nbspID:GO:0019226** | | | | | | |
| --- | --- | --- | --- | --- | --- | --- |
| C=552; O=50; E=12.31; R=4.06; rawP=3.29e-17; adjP=1.05e-14 | | | | | | |
| Index | UserID | Value | Gene Symbol | Gene Name | EntrezGene | Ensembl |
| 1 | 17318923 | NA | Cacng2 | calcium channel, voltage-dependent, gamma subunit 2 | 12300 | ENSMUSG00000019146 |
| 2 | 17368550 | NA | Rxra | retinoid X receptor alpha | 20181 | ENSMUSG00000015846 |
| 3 | 17461923 | NA | Syn2 | synapsin II | 20965 | ENSMUSG00000009394 |
| 4 | 17384021 | NA | Stxbp1 | syntaxin binding protein 1 | 20910 | ENSMUSG00000026797 |
| 5 | 17342719 | NA | Grm4 | glutamate receptor, metabotropic 4 | 268934 | ENSMUSG00000063239 |
| 6 | 17436545 | NA | Nat8l | N-acetyltransferase 8-like | 269642 | ENSMUSG00000048142 |
| 7 | 17520198 | NA | Rasgrf1 | RAS protein-specific guanine nucleotide-releasing factor 1 | 19417 | ENSMUSG00000032356 |
| 8 | 17529218 | NA | Htr1b | 5-hydroxytryptamine (serotonin) receptor 1B | 15551 | ENSMUSG00000049511 |
| 9 | 17337513 | NA | Gabbr1 | gamma-aminobutyric acid (GABA) B receptor, 1 | 54393 | ENSMUSG00000024462 |
| 10 | 17243717 | NA | Syn3 | synapsin III | 27204 | ENSMUSG00000059602 |
| 11 | 17314164 | NA | Mapk8ip2 | mitogen-activated protein kinase 8 interacting protein 2 | 60597 | ENSMUSG00000022619 |
| 12 | 17509944 | NA | Ncan | neurocan | 13004 | ENSMUSG00000002341 |
| 13 | 17502603 | NA | Rasd2 | RASD family, member 2 | 75141 | ENSMUSG00000034472 |
| 14 | 17530733 | NA | Grm2 | glutamate receptor, metabotropic 2 | 108068 | ENSMUSG00000023192 |
| 15 | 17503122 | NA | Cacna1a | calcium channel, voltage-dependent, P/Q type, alpha 1A subunit | 12286 | ENSMUSG00000034656 |
| 16 | 17310772 | NA | Ctnnd2 | catenin (cadherin associated protein), delta 2 | 18163 | ENSMUSG00000022240 |
| 17 | 17329516 | NA | Fgf12 | fibroblast growth factor 12 | 14167 | ENSMUSG00000022523 |
| 18 | 17400521 | NA | Sv2a | synaptic vesicle glycoprotein 2 a | 64051 | ENSMUSG00000038486 |
| 19 | 17308939 | NA | Pcdh8 | protocadherin 8 | 18530 | ENSMUSG00000036422 |
| 20 | 17504130 | NA | Cx3cl1 | chemokine (C-X3-C motif) ligand 1 | 20312 | ENSMUSG00000031778 |
| 21 | 17382496 | NA | Grin1 | glutamate receptor, ionotropic, NMDA1 (zeta 1) | 14810 | ENSMUSG00000026959 |
| 22 | 17367921 | NA | Grin1 | glutamate receptor, ionotropic, NMDA1 (zeta 1) | 14810 | ENSMUSG00000026959 |
| 23 | 17226891 | NA | Cntn2 | contactin 2 | 21367 | ENSMUSG00000053024 |
| 24 | 17243057 | NA | Ap3d1 | adaptor-related protein complex 3, delta 1 subunit | 11776 | ENSMUSG00000020198 |
| 25 | 17253376 | NA | Sez6 | seizure related gene 6 | 20370 | ENSMUSG00000000632 |
| 26 | 17263011 | NA | Glra1 | glycine receptor, alpha 1 subunit | 14654 | ENSMUSG00000000263 |
| 27 | 17268786 | NA | Neurod2 | neurogenic differentiation 2 | 18013 | ENSMUSG00000038255 |
| 28 | 17517073 | NA | Drd2 | dopamine receptor D2 | 13489 | ENSMUSG00000032259 |
| 29 | 17469814 | NA | Atp2b2 | ATPase, Ca++ transporting, plasma membrane 2 | 11941 | ENSMUSG00000030302 |
| 30 | 17435528 | NA | Dpp6 | dipeptidylpeptidase 6 | 13483 | ENSMUSG00000061576 |
| 31 | 17536720 | NA | Nlgn3 | neuroligin 3 | 245537 | ENSMUSG00000031302 |
| 32 | 17502039 | NA | Rab3a | RAB3A, member RAS oncogene family | 19339 | ENSMUSG00000031840 |
| 33 | 17407124 | NA | Chrnb2 | cholinergic receptor, nicotinic, beta polypeptide 2 (neuronal) | 11444 | ENSMUSG00000027950 |
| 34 | 17461868 | NA | Slc6a1 | solute carrier family 6 (neurotransmitter transporter, GABA), member 1 | 232333 | ENSMUSG00000030310 |
| 35 | 17307905 | NA | Dpysl2 | dihydropyrimidinase-like 2 | 12934 | ENSMUSG00000022048 |
| 36 | 17239234 | NA | Grm1 | glutamate receptor, metabotropic 1 | 14816 | ENSMUSG00000019828 |
| 37 | 17361454 | NA | Cnih2 | cornichon homolog 2 (Drosophila) | 12794 | ENSMUSG00000024873 |
| 38 | 17430140 | NA | Ncdn | neurochondrin | 26562 | ENSMUSG00000028833 |
| 39 | 17496763 | NA | Stx1b | syntaxin 1B | 56216 | ENSMUSG00000030806 |
| 40 | 17441453 | NA | Nos1 | nitric oxide synthase 1, neuronal | 18125 | ENSMUSG00000029361 |
| 41 | 17540501 | NA | Syn1 | synapsin I | 20964 | ENSMUSG00000037217 |
| 42 | 17256579 | NA | Cntnap1 | contactin associated protein-like 1 | 53321 | ENSMUSG00000017167 |
| 43 | 17521327 | NA | Cacna2d2 | calcium channel, voltage-dependent, alpha 2/delta subunit 2 | 56808 | ENSMUSG00000010066 |
| 44 | 17477468 | NA | Lrrc4b | leucine rich repeat containing 4B | 272381 | ENSMUSG00000047085 |
| 45 | 17477714 | NA | Slc17a7 | solute carrier family 17 (sodium-dependent inorganic phosphate cotransporter), member 7 | 72961 | ENSMUSG00000070570 |
| 46 | 17401650 | NA | Amigo1 | adhesion molecule with Ig like domain 1 | 229715 | ENSMUSG00000050947 |
| 47 | 17532879 | NA | Syp | synaptophysin | 20977 | ENSMUSG00000031144 |
| 48 | 17356924 | NA | Nrxn2 | neurexin II | 18190 | ENSMUSG00000033768 |
| 49 | 17215820 | NA | Gpc1 | glypican 1 | 14733 | ENSMUSG00000034220 |
| 50 | 17260261 | NA | Camk2b | calcium/calmodulin-dependent protein kinase II, beta | 12323 | ENSMUSG00000057897 |
| 51 | 17394079 | NA | Rims4 | regulating synaptic membrane exocytosis 4 | 241770 | ENSMUSG00000035226 |

  
  

| **Database:biological process      &nbspName:synaptic transmission      &nbspID:GO:0007268** | | | | | | |
| --- | --- | --- | --- | --- | --- | --- |
| C=440; O=44; E=9.81; R=4.48; rawP=7.70e-17; adjP=2.10e-14 | | | | | | |
| Index | UserID | Value | Gene Symbol | Gene Name | EntrezGene | Ensembl |
| 1 | 17461923 | NA | Syn2 | synapsin II | 20965 | ENSMUSG00000009394 |
| 2 | 17384021 | NA | Stxbp1 | syntaxin binding protein 1 | 20910 | ENSMUSG00000026797 |
| 3 | 17342719 | NA | Grm4 | glutamate receptor, metabotropic 4 | 268934 | ENSMUSG00000063239 |
| 4 | 17436545 | NA | Nat8l | N-acetyltransferase 8-like | 269642 | ENSMUSG00000048142 |
| 5 | 17520198 | NA | Rasgrf1 | RAS protein-specific guanine nucleotide-releasing factor 1 | 19417 | ENSMUSG00000032356 |
| 6 | 17529218 | NA | Htr1b | 5-hydroxytryptamine (serotonin) receptor 1B | 15551 | ENSMUSG00000049511 |
| 7 | 17337513 | NA | Gabbr1 | gamma-aminobutyric acid (GABA) B receptor, 1 | 54393 | ENSMUSG00000024462 |
| 8 | 17243717 | NA | Syn3 | synapsin III | 27204 | ENSMUSG00000059602 |
| 9 | 17314164 | NA | Mapk8ip2 | mitogen-activated protein kinase 8 interacting protein 2 | 60597 | ENSMUSG00000022619 |
| 10 | 17509944 | NA | Ncan | neurocan | 13004 | ENSMUSG00000002341 |
| 11 | 17502603 | NA | Rasd2 | RASD family, member 2 | 75141 | ENSMUSG00000034472 |
| 12 | 17530733 | NA | Grm2 | glutamate receptor, metabotropic 2 | 108068 | ENSMUSG00000023192 |
| 13 | 17503122 | NA | Cacna1a | calcium channel, voltage-dependent, P/Q type, alpha 1A subunit | 12286 | ENSMUSG00000034656 |
| 14 | 17310772 | NA | Ctnnd2 | catenin (cadherin associated protein), delta 2 | 18163 | ENSMUSG00000022240 |
| 15 | 17329516 | NA | Fgf12 | fibroblast growth factor 12 | 14167 | ENSMUSG00000022523 |
| 16 | 17400521 | NA | Sv2a | synaptic vesicle glycoprotein 2 a | 64051 | ENSMUSG00000038486 |
| 17 | 17308939 | NA | Pcdh8 | protocadherin 8 | 18530 | ENSMUSG00000036422 |
| 18 | 17504130 | NA | Cx3cl1 | chemokine (C-X3-C motif) ligand 1 | 20312 | ENSMUSG00000031778 |
| 19 | 17382496 | NA | Grin1 | glutamate receptor, ionotropic, NMDA1 (zeta 1) | 14810 | ENSMUSG00000026959 |
| 20 | 17367921 | NA | Grin1 | glutamate receptor, ionotropic, NMDA1 (zeta 1) | 14810 | ENSMUSG00000026959 |
| 21 | 17226891 | NA | Cntn2 | contactin 2 | 21367 | ENSMUSG00000053024 |
| 22 | 17243057 | NA | Ap3d1 | adaptor-related protein complex 3, delta 1 subunit | 11776 | ENSMUSG00000020198 |
| 23 | 17263011 | NA | Glra1 | glycine receptor, alpha 1 subunit | 14654 | ENSMUSG00000000263 |
| 24 | 17253376 | NA | Sez6 | seizure related gene 6 | 20370 | ENSMUSG00000000632 |
| 25 | 17268786 | NA | Neurod2 | neurogenic differentiation 2 | 18013 | ENSMUSG00000038255 |
| 26 | 17517073 | NA | Drd2 | dopamine receptor D2 | 13489 | ENSMUSG00000032259 |
| 27 | 17469814 | NA | Atp2b2 | ATPase, Ca++ transporting, plasma membrane 2 | 11941 | ENSMUSG00000030302 |
| 28 | 17536720 | NA | Nlgn3 | neuroligin 3 | 245537 | ENSMUSG00000031302 |
| 29 | 17502039 | NA | Rab3a | RAB3A, member RAS oncogene family | 19339 | ENSMUSG00000031840 |
| 30 | 17407124 | NA | Chrnb2 | cholinergic receptor, nicotinic, beta polypeptide 2 (neuronal) | 11444 | ENSMUSG00000027950 |
| 31 | 17461868 | NA | Slc6a1 | solute carrier family 6 (neurotransmitter transporter, GABA), member 1 | 232333 | ENSMUSG00000030310 |
| 32 | 17307905 | NA | Dpysl2 | dihydropyrimidinase-like 2 | 12934 | ENSMUSG00000022048 |
| 33 | 17239234 | NA | Grm1 | glutamate receptor, metabotropic 1 | 14816 | ENSMUSG00000019828 |
| 34 | 17361454 | NA | Cnih2 | cornichon homolog 2 (Drosophila) | 12794 | ENSMUSG00000024873 |
| 35 | 17430140 | NA | Ncdn | neurochondrin | 26562 | ENSMUSG00000028833 |
| 36 | 17496763 | NA | Stx1b | syntaxin 1B | 56216 | ENSMUSG00000030806 |
| 37 | 17441453 | NA | Nos1 | nitric oxide synthase 1, neuronal | 18125 | ENSMUSG00000029361 |
| 38 | 17540501 | NA | Syn1 | synapsin I | 20964 | ENSMUSG00000037217 |
| 39 | 17477468 | NA | Lrrc4b | leucine rich repeat containing 4B | 272381 | ENSMUSG00000047085 |
| 40 | 17521327 | NA | Cacna2d2 | calcium channel, voltage-dependent, alpha 2/delta subunit 2 | 56808 | ENSMUSG00000010066 |
| 41 | 17477714 | NA | Slc17a7 | solute carrier family 17 (sodium-dependent inorganic phosphate cotransporter), member 7 | 72961 | ENSMUSG00000070570 |
| 42 | 17532879 | NA | Syp | synaptophysin | 20977 | ENSMUSG00000031144 |
| 43 | 17356924 | NA | Nrxn2 | neurexin II | 18190 | ENSMUSG00000033768 |
| 44 | 17260261 | NA | Camk2b | calcium/calmodulin-dependent protein kinase II, beta | 12323 | ENSMUSG00000057897 |
| 45 | 17394079 | NA | Rims4 | regulating synaptic membrane exocytosis 4 | 241770 | ENSMUSG00000035226 |

  
  

| **Database:biological process      &nbspName:neuron development      &nbspID:GO:0048666** | | | | | | |
| --- | --- | --- | --- | --- | --- | --- |
| C=628; O=52; E=14.01; R=3.71; rawP=3.24e-16; adjP=7.72e-14 | | | | | | |
| Index | UserID | Value | Gene Symbol | Gene Name | EntrezGene | Ensembl |
| 1 | 17309287 | NA | Pou4f1 | POU domain, class 4, transcription factor 1 | 18996 | ENSMUSG00000048349 |
| 2 | 17516462 | NA | Thy1 | thymus cell antigen 1, theta | 21838 | ENSMUSG00000032011 |
| 3 | 17384021 | NA | Stxbp1 | syntaxin binding protein 1 | 20910 | ENSMUSG00000026797 |
| 4 | 17520198 | NA | Rasgrf1 | RAS protein-specific guanine nucleotide-releasing factor 1 | 19417 | ENSMUSG00000032356 |
| 5 | 17439511 | NA | Prdm8 | PR domain containing 8 | 77630 | ENSMUSG00000035456 |
| 6 | 17314164 | NA | Mapk8ip2 | mitogen-activated protein kinase 8 interacting protein 2 | 60597 | ENSMUSG00000022619 |
| 7 | 17503122 | NA | Cacna1a | calcium channel, voltage-dependent, P/Q type, alpha 1A subunit | 12286 | ENSMUSG00000034656 |
| 8 | 17302600 | NA | Slitrk5 | SLIT and NTRK-like family, member 5 | 75409 | ENSMUSG00000033214 |
| 9 | 17506808 | NA | Trim67 | tripartite motif-containing 67 | 330863 | ENSMUSG00000036913 |
| 10 | 17342065 | NA | Mapk8ip3 | mitogen-activated protein kinase 8 interacting protein 3 | 30957 | ENSMUSG00000024163 |
| 11 | 17382496 | NA | Grin1 | glutamate receptor, ionotropic, NMDA1 (zeta 1) | 14810 | ENSMUSG00000026959 |
| 12 | 17367921 | NA | Grin1 | glutamate receptor, ionotropic, NMDA1 (zeta 1) | 14810 | ENSMUSG00000026959 |
| 13 | 17226891 | NA | Cntn2 | contactin 2 | 21367 | ENSMUSG00000053024 |
| 14 | 17357959 | NA | Gnaq | guanine nucleotide binding protein, alpha q polypeptide | 14682 | ENSMUSG00000024639 |
| 15 | 17335204 | NA | Anks1 | ankyrin repeat and SAM domain containing 1 | 224650 | ENSMUSG00000024219 |
| 16 | 17272926 | NA | Nptx1 | neuronal pentraxin 1 | 18164 | ENSMUSG00000025582 |
| 17 | 17253376 | NA | Sez6 | seizure related gene 6 | 20370 | ENSMUSG00000000632 |
| 18 | 17268786 | NA | Neurod2 | neurogenic differentiation 2 | 18013 | ENSMUSG00000038255 |
| 19 | 17406760 | NA | Sema4a | sema domain, immunoglobulin domain (Ig), transmembrane domain (TM) and short cytoplasmic domain, (semaphorin) 4A | 20351 | ENSMUSG00000028064 |
| 20 | 17475564 | NA | Numbl | numb-like | 18223 | ENSMUSG00000063160 |
| 21 | 17484419 | NA | Kndc1 | kinase non-catalytic C-lobe domain (KIND) containing 1 | 76484 | ENSMUSG00000066129 |
| 22 | 17517073 | NA | Drd2 | dopamine receptor D2 | 13489 | ENSMUSG00000032259 |
| 23 | 17461897 | NA | Atg7 | autophagy related 7 | 74244 | ENSMUSG00000030314 |
| 24 | 17435834 | NA | Dpysl5 | dihydropyrimidinase-like 5 | 65254 | ENSMUSG00000029168 |
| 25 | 17542419 | NA | L1cam | L1 cell adhesion molecule | 16728 | ENSMUSG00000031391 |
| 26 | 17469814 | NA | Atp2b2 | ATPase, Ca++ transporting, plasma membrane 2 | 11941 | ENSMUSG00000030302 |
| 27 | 17385374 | NA | Nr4a2 | nuclear receptor subfamily 4, group A, member 2 | 18227 | ENSMUSG00000026826 |
| 28 | 17354299 | NA | Sema6a | sema domain, transmembrane domain (TM), and cytoplasmic domain, (semaphorin) 6A | 20358 | ENSMUSG00000019647 |
| 29 | 17235663 | NA | Pip5k1c | phosphatidylinositol-4-phosphate 5-kinase, type 1 gamma | 18717 | ENSMUSG00000034902 |
| 30 | 17292753 | NA | Gprin1 | G protein-regulated inducer of neurite outgrowth 1 | 26913 | ENSMUSG00000069227 |
| 31 | 17536720 | NA | Nlgn3 | neuroligin 3 | 245537 | ENSMUSG00000031302 |
| 32 | 17450536 | NA | Barhl2 | BarH-like 2 (Drosophila) | 104382 | ENSMUSG00000034384 |
| 33 | 17502039 | NA | Rab3a | RAB3A, member RAS oncogene family | 19339 | ENSMUSG00000031840 |
| 34 | 17332495 | NA | Dscam | Down syndrome cell adhesion molecule | 13508 | ENSMUSG00000050272 |
| 35 | 17407124 | NA | Chrnb2 | cholinergic receptor, nicotinic, beta polypeptide 2 (neuronal) | 11444 | ENSMUSG00000027950 |
| 36 | 17453430 | NA | Limk1 | LIM-domain containing, protein kinase | 16885 | ENSMUSG00000029674 |
| 37 | 17335145 | NA | Pacsin1 | protein kinase C and casein kinase substrate in neurons 1 | 23969 | ENSMUSG00000040276 |
| 38 | 17243229 | NA | Atcay | ataxia, cerebellar, Cayman type homolog (human) | 16467 | ENSMUSG00000034958 |
| 39 | 17430140 | NA | Ncdn | neurochondrin | 26562 | ENSMUSG00000028833 |
| 40 | 17345542 | NA | Ppp2r5d | protein phosphatase 2, regulatory subunit B (B56), delta isoform | 21770 | ENSMUSG00000059409 |
| 41 | 17364665 | NA | Slit1 | slit homolog 1 (Drosophila) | 20562 | ENSMUSG00000025020 |
| 42 | 17257197 | NA | Mapt | microtubule-associated protein tau | 17762 | ENSMUSG00000018411 |
| 43 | 17510696 | NA | Pou4f2 | POU domain, class 4, transcription factor 2 | 18997 | ENSMUSG00000031688 |
| 44 | 17256579 | NA | Cntnap1 | contactin associated protein-like 1 | 53321 | ENSMUSG00000017167 |
| 45 | 17292011 | NA | Tfap2a | transcription factor AP-2, alpha | 21418 | ENSMUSG00000021359 |
| 46 | 17485152 | NA | Brsk2 | BR serine/threonine kinase 2 | 75770 | ENSMUSG00000053046 |
| 47 | 17401650 | NA | Amigo1 | adhesion molecule with Ig like domain 1 | 229715 | ENSMUSG00000050947 |
| 48 | 17293045 | NA | Spock1 | sparc/osteonectin, cwcv and kazal-like domains proteoglycan 1 | 20745 | ENSMUSG00000056222 |
| 49 | 17269439 | NA | Hap1 | huntingtin-associated protein 1 | 15114 | ENSMUSG00000006930 |
| 50 | 17260261 | NA | Camk2b | calcium/calmodulin-dependent protein kinase II, beta | 12323 | ENSMUSG00000057897 |
| 51 | 17267039 | NA | Lhx1 | LIM homeobox protein 1 | 16869 | ENSMUSG00000018698 |
| 52 | 17259810 | NA | Inpp5j | inositol polyphosphate 5-phosphatase J | 170835 | ENSMUSG00000034570 |
| 53 | 17451140 | NA | Ulk1 | unc-51 like kinase 1 | 22241 | ENSMUSG00000029512 |

  
  

| **Database:biological process      &nbspName:neuron projection development      &nbspID:GO:0031175** | | | | | | |
| --- | --- | --- | --- | --- | --- | --- |
| C=522; O=46; E=11.64; R=3.95; rawP=1.86e-15; adjP=3.94e-13 | | | | | | |
| Index | UserID | Value | Gene Symbol | Gene Name | EntrezGene | Ensembl |
| 1 | 17516462 | NA | Thy1 | thymus cell antigen 1, theta | 21838 | ENSMUSG00000032011 |
| 2 | 17384021 | NA | Stxbp1 | syntaxin binding protein 1 | 20910 | ENSMUSG00000026797 |
| 3 | 17520198 | NA | Rasgrf1 | RAS protein-specific guanine nucleotide-releasing factor 1 | 19417 | ENSMUSG00000032356 |
| 4 | 17439511 | NA | Prdm8 | PR domain containing 8 | 77630 | ENSMUSG00000035456 |
| 5 | 17314164 | NA | Mapk8ip2 | mitogen-activated protein kinase 8 interacting protein 2 | 60597 | ENSMUSG00000022619 |
| 6 | 17503122 | NA | Cacna1a | calcium channel, voltage-dependent, P/Q type, alpha 1A subunit | 12286 | ENSMUSG00000034656 |
| 7 | 17302600 | NA | Slitrk5 | SLIT and NTRK-like family, member 5 | 75409 | ENSMUSG00000033214 |
| 8 | 17506808 | NA | Trim67 | tripartite motif-containing 67 | 330863 | ENSMUSG00000036913 |
| 9 | 17342065 | NA | Mapk8ip3 | mitogen-activated protein kinase 8 interacting protein 3 | 30957 | ENSMUSG00000024163 |
| 10 | 17382496 | NA | Grin1 | glutamate receptor, ionotropic, NMDA1 (zeta 1) | 14810 | ENSMUSG00000026959 |
| 11 | 17367921 | NA | Grin1 | glutamate receptor, ionotropic, NMDA1 (zeta 1) | 14810 | ENSMUSG00000026959 |
| 12 | 17226891 | NA | Cntn2 | contactin 2 | 21367 | ENSMUSG00000053024 |
| 13 | 17272926 | NA | Nptx1 | neuronal pentraxin 1 | 18164 | ENSMUSG00000025582 |
| 14 | 17253376 | NA | Sez6 | seizure related gene 6 | 20370 | ENSMUSG00000000632 |
| 15 | 17406760 | NA | Sema4a | sema domain, immunoglobulin domain (Ig), transmembrane domain (TM) and short cytoplasmic domain, (semaphorin) 4A | 20351 | ENSMUSG00000028064 |
| 16 | 17475564 | NA | Numbl | numb-like | 18223 | ENSMUSG00000063160 |
| 17 | 17484419 | NA | Kndc1 | kinase non-catalytic C-lobe domain (KIND) containing 1 | 76484 | ENSMUSG00000066129 |
| 18 | 17517073 | NA | Drd2 | dopamine receptor D2 | 13489 | ENSMUSG00000032259 |
| 19 | 17461897 | NA | Atg7 | autophagy related 7 | 74244 | ENSMUSG00000030314 |
| 20 | 17435834 | NA | Dpysl5 | dihydropyrimidinase-like 5 | 65254 | ENSMUSG00000029168 |
| 21 | 17542419 | NA | L1cam | L1 cell adhesion molecule | 16728 | ENSMUSG00000031391 |
| 22 | 17385374 | NA | Nr4a2 | nuclear receptor subfamily 4, group A, member 2 | 18227 | ENSMUSG00000026826 |
| 23 | 17354299 | NA | Sema6a | sema domain, transmembrane domain (TM), and cytoplasmic domain, (semaphorin) 6A | 20358 | ENSMUSG00000019647 |
| 24 | 17235663 | NA | Pip5k1c | phosphatidylinositol-4-phosphate 5-kinase, type 1 gamma | 18717 | ENSMUSG00000034902 |
| 25 | 17292753 | NA | Gprin1 | G protein-regulated inducer of neurite outgrowth 1 | 26913 | ENSMUSG00000069227 |
| 26 | 17536720 | NA | Nlgn3 | neuroligin 3 | 245537 | ENSMUSG00000031302 |
| 27 | 17502039 | NA | Rab3a | RAB3A, member RAS oncogene family | 19339 | ENSMUSG00000031840 |
| 28 | 17450536 | NA | Barhl2 | BarH-like 2 (Drosophila) | 104382 | ENSMUSG00000034384 |
| 29 | 17332495 | NA | Dscam | Down syndrome cell adhesion molecule | 13508 | ENSMUSG00000050272 |
| 30 | 17407124 | NA | Chrnb2 | cholinergic receptor, nicotinic, beta polypeptide 2 (neuronal) | 11444 | ENSMUSG00000027950 |
| 31 | 17453430 | NA | Limk1 | LIM-domain containing, protein kinase | 16885 | ENSMUSG00000029674 |
| 32 | 17335145 | NA | Pacsin1 | protein kinase C and casein kinase substrate in neurons 1 | 23969 | ENSMUSG00000040276 |
| 33 | 17243229 | NA | Atcay | ataxia, cerebellar, Cayman type homolog (human) | 16467 | ENSMUSG00000034958 |
| 34 | 17430140 | NA | Ncdn | neurochondrin | 26562 | ENSMUSG00000028833 |
| 35 | 17345542 | NA | Ppp2r5d | protein phosphatase 2, regulatory subunit B (B56), delta isoform | 21770 | ENSMUSG00000059409 |
| 36 | 17364665 | NA | Slit1 | slit homolog 1 (Drosophila) | 20562 | ENSMUSG00000025020 |
| 37 | 17257197 | NA | Mapt | microtubule-associated protein tau | 17762 | ENSMUSG00000018411 |
| 38 | 17510696 | NA | Pou4f2 | POU domain, class 4, transcription factor 2 | 18997 | ENSMUSG00000031688 |
| 39 | 17256579 | NA | Cntnap1 | contactin associated protein-like 1 | 53321 | ENSMUSG00000017167 |
| 40 | 17485152 | NA | Brsk2 | BR serine/threonine kinase 2 | 75770 | ENSMUSG00000053046 |
| 41 | 17401650 | NA | Amigo1 | adhesion molecule with Ig like domain 1 | 229715 | ENSMUSG00000050947 |
| 42 | 17293045 | NA | Spock1 | sparc/osteonectin, cwcv and kazal-like domains proteoglycan 1 | 20745 | ENSMUSG00000056222 |
| 43 | 17269439 | NA | Hap1 | huntingtin-associated protein 1 | 15114 | ENSMUSG00000006930 |
| 44 | 17260261 | NA | Camk2b | calcium/calmodulin-dependent protein kinase II, beta | 12323 | ENSMUSG00000057897 |
| 45 | 17267039 | NA | Lhx1 | LIM homeobox protein 1 | 16869 | ENSMUSG00000018698 |
| 46 | 17259810 | NA | Inpp5j | inositol polyphosphate 5-phosphatase J | 170835 | ENSMUSG00000034570 |
| 47 | 17451140 | NA | Ulk1 | unc-51 like kinase 1 | 22241 | ENSMUSG00000029512 |

  
  

| **Database:biological process      &nbspName:cell-cell signaling      &nbspID:GO:0007267** | | | | | | |
| --- | --- | --- | --- | --- | --- | --- |
| C=724; O=54; E=16.15; R=3.34; rawP=6.96e-15; adjP=1.33e-12 | | | | | | |
| Index | UserID | Value | Gene Symbol | Gene Name | EntrezGene | Ensembl |
| 1 | 17314051 | NA | Panx2 | pannexin 2 | 406218 | ENSMUSG00000058441 |
| 2 | 17461923 | NA | Syn2 | synapsin II | 20965 | ENSMUSG00000009394 |
| 3 | 17384021 | NA | Stxbp1 | syntaxin binding protein 1 | 20910 | ENSMUSG00000026797 |
| 4 | 17342719 | NA | Grm4 | glutamate receptor, metabotropic 4 | 268934 | ENSMUSG00000063239 |
| 5 | 17436545 | NA | Nat8l | N-acetyltransferase 8-like | 269642 | ENSMUSG00000048142 |
| 6 | 17520198 | NA | Rasgrf1 | RAS protein-specific guanine nucleotide-releasing factor 1 | 19417 | ENSMUSG00000032356 |
| 7 | 17529218 | NA | Htr1b | 5-hydroxytryptamine (serotonin) receptor 1B | 15551 | ENSMUSG00000049511 |
| 8 | 17337513 | NA | Gabbr1 | gamma-aminobutyric acid (GABA) B receptor, 1 | 54393 | ENSMUSG00000024462 |
| 9 | 17243717 | NA | Syn3 | synapsin III | 27204 | ENSMUSG00000059602 |
| 10 | 17314164 | NA | Mapk8ip2 | mitogen-activated protein kinase 8 interacting protein 2 | 60597 | ENSMUSG00000022619 |
| 11 | 17509944 | NA | Ncan | neurocan | 13004 | ENSMUSG00000002341 |
| 12 | 17502603 | NA | Rasd2 | RASD family, member 2 | 75141 | ENSMUSG00000034472 |
| 13 | 17530733 | NA | Grm2 | glutamate receptor, metabotropic 2 | 108068 | ENSMUSG00000023192 |
| 14 | 17503122 | NA | Cacna1a | calcium channel, voltage-dependent, P/Q type, alpha 1A subunit | 12286 | ENSMUSG00000034656 |
| 15 | 17310772 | NA | Ctnnd2 | catenin (cadherin associated protein), delta 2 | 18163 | ENSMUSG00000022240 |
| 16 | 17329516 | NA | Fgf12 | fibroblast growth factor 12 | 14167 | ENSMUSG00000022523 |
| 17 | 17400521 | NA | Sv2a | synaptic vesicle glycoprotein 2 a | 64051 | ENSMUSG00000038486 |
| 18 | 17308939 | NA | Pcdh8 | protocadherin 8 | 18530 | ENSMUSG00000036422 |
| 19 | 17504130 | NA | Cx3cl1 | chemokine (C-X3-C motif) ligand 1 | 20312 | ENSMUSG00000031778 |
| 20 | 17382496 | NA | Grin1 | glutamate receptor, ionotropic, NMDA1 (zeta 1) | 14810 | ENSMUSG00000026959 |
| 21 | 17367921 | NA | Grin1 | glutamate receptor, ionotropic, NMDA1 (zeta 1) | 14810 | ENSMUSG00000026959 |
| 22 | 17226891 | NA | Cntn2 | contactin 2 | 21367 | ENSMUSG00000053024 |
| 23 | 17243057 | NA | Ap3d1 | adaptor-related protein complex 3, delta 1 subunit | 11776 | ENSMUSG00000020198 |
| 24 | 17253376 | NA | Sez6 | seizure related gene 6 | 20370 | ENSMUSG00000000632 |
| 25 | 17263011 | NA | Glra1 | glycine receptor, alpha 1 subunit | 14654 | ENSMUSG00000000263 |
| 26 | 17418732 | NA | Dlgap3 | discs, large (Drosophila) homolog-associated protein 3 | 242667 | ENSMUSG00000042388 |
| 27 | 17268786 | NA | Neurod2 | neurogenic differentiation 2 | 18013 | ENSMUSG00000038255 |
| 28 | 17211347 | NA | Tfap2b | transcription factor AP-2 beta | 21419 | ENSMUSG00000025927 |
| 29 | 17517073 | NA | Drd2 | dopamine receptor D2 | 13489 | ENSMUSG00000032259 |
| 30 | 17469814 | NA | Atp2b2 | ATPase, Ca++ transporting, plasma membrane 2 | 11941 | ENSMUSG00000030302 |
| 31 | 17536720 | NA | Nlgn3 | neuroligin 3 | 245537 | ENSMUSG00000031302 |
| 32 | 17502039 | NA | Rab3a | RAB3A, member RAS oncogene family | 19339 | ENSMUSG00000031840 |
| 33 | 17407124 | NA | Chrnb2 | cholinergic receptor, nicotinic, beta polypeptide 2 (neuronal) | 11444 | ENSMUSG00000027950 |
| 34 | 17461868 | NA | Slc6a1 | solute carrier family 6 (neurotransmitter transporter, GABA), member 1 | 232333 | ENSMUSG00000030310 |
| 35 | 17307905 | NA | Dpysl2 | dihydropyrimidinase-like 2 | 12934 | ENSMUSG00000022048 |
| 36 | 17290259 | NA | Ucn3 | urocortin 3 | 83428 | ENSMUSG00000044988 |
| 37 | 17239234 | NA | Grm1 | glutamate receptor, metabotropic 1 | 14816 | ENSMUSG00000019828 |
| 38 | 17361454 | NA | Cnih2 | cornichon homolog 2 (Drosophila) | 12794 | ENSMUSG00000024873 |
| 39 | 17441595 | NA | Tbx3 | T-box 3 | 21386 | ENSMUSG00000018604 |
| 40 | 17430140 | NA | Ncdn | neurochondrin | 26562 | ENSMUSG00000028833 |
| 41 | 17357486 | NA | Syt7 | synaptotagmin VII | 54525 | ENSMUSG00000024743 |
| 42 | 17496763 | NA | Stx1b | syntaxin 1B | 56216 | ENSMUSG00000030806 |
| 43 | 17441453 | NA | Nos1 | nitric oxide synthase 1, neuronal | 18125 | ENSMUSG00000029361 |
| 44 | 17540501 | NA | Syn1 | synapsin I | 20964 | ENSMUSG00000037217 |
| 45 | 17292011 | NA | Tfap2a | transcription factor AP-2, alpha | 21418 | ENSMUSG00000021359 |
| 46 | 17521327 | NA | Cacna2d2 | calcium channel, voltage-dependent, alpha 2/delta subunit 2 | 56808 | ENSMUSG00000010066 |
| 47 | 17477468 | NA | Lrrc4b | leucine rich repeat containing 4B | 272381 | ENSMUSG00000047085 |
| 48 | 17477714 | NA | Slc17a7 | solute carrier family 17 (sodium-dependent inorganic phosphate cotransporter), member 7 | 72961 | ENSMUSG00000070570 |
| 49 | 17532879 | NA | Syp | synaptophysin | 20977 | ENSMUSG00000031144 |
| 50 | 17356924 | NA | Nrxn2 | neurexin II | 18190 | ENSMUSG00000033768 |
| 51 | 17533640 | NA | Cdk16 | cyclin-dependent kinase 16 | 18555 | ENSMUSG00000031065 |
| 52 | 17360440 | NA | Adra2a | adrenergic receptor, alpha 2a | 11551 | ENSMUSG00000033717 |
| 53 | 17260261 | NA | Camk2b | calcium/calmodulin-dependent protein kinase II, beta | 12323 | ENSMUSG00000057897 |
| 54 | 17394079 | NA | Rims4 | regulating synaptic membrane exocytosis 4 | 241770 | ENSMUSG00000035226 |
| 55 | 17267039 | NA | Lhx1 | LIM homeobox protein 1 | 16869 | ENSMUSG00000018698 |

  
  

| **Database:molecular function      &nbspName:metal ion transmembrane transporter activity      &nbspID:GO:0046873** | | | | | | |
| --- | --- | --- | --- | --- | --- | --- |
| C=333; O=33; E=7.45; R=4.43; rawP=8.98e-13; adjP=3.61e-10 | | | | | | |
| Index | UserID | Value | Gene Symbol | Gene Name | EntrezGene | Ensembl |
| 1 | 17417702 | NA | Slc6a9 | solute carrier family 6 (neurotransmitter transporter, glycine), member 9 | 14664 | ENSMUSG00000028542 |
| 2 | 17318923 | NA | Cacng2 | calcium channel, voltage-dependent, gamma subunit 2 | 12300 | ENSMUSG00000019146 |
| 3 | 17378922 | NA | Slc32a1 | solute carrier family 32 (GABA vesicular transporter), member 1 | 22348 | ENSMUSG00000037771 |
| 4 | 17308299 | NA | Slc39a14 | solute carrier family 39 (zinc transporter), member 14 | 213053 | ENSMUSG00000022094 |
| 5 | 17461852 | NA | Slc6a11 | solute carrier family 6 (neurotransmitter transporter, GABA), member 11 | 243616 | ENSMUSG00000030307 |
| 6 | 17503122 | NA | Cacna1a | calcium channel, voltage-dependent, P/Q type, alpha 1A subunit | 12286 | ENSMUSG00000034656 |
| 7 | 17474067 | NA | Slc8a2 | solute carrier family 8 (sodium/calcium exchanger), member 2 | 110891 | ENSMUSG00000030376 |
| 8 | 17382496 | NA | Grin1 | glutamate receptor, ionotropic, NMDA1 (zeta 1) | 14810 | ENSMUSG00000026959 |
| 9 | 17367921 | NA | Grin1 | glutamate receptor, ionotropic, NMDA1 (zeta 1) | 14810 | ENSMUSG00000026959 |
| 10 | 17377144 | NA | Slc24a3 | solute carrier family 24 (sodium/potassium/calcium exchanger), member 3 | 94249 | ENSMUSG00000063873 |
| 11 | 17446123 | NA | Kcnh2 | potassium voltage-gated channel, subfamily H (eag-related), member 2 | 16511 | ENSMUSG00000038319 |
| 12 | 17419587 | NA | Slc9a1 | solute carrier family 9 (sodium/hydrogen exchanger), member 1 | 20544 | ENSMUSG00000028854 |
| 13 | 17370883 | NA | Kcnj3 | potassium inwardly-rectifying channel, subfamily J, member 3 | 16519 | ENSMUSG00000026824 |
| 14 | 17469814 | NA | Atp2b2 | ATPase, Ca++ transporting, plasma membrane 2 | 11941 | ENSMUSG00000030302 |
| 15 | 17401394 | NA | Kcnd3 | potassium voltage-gated channel, Shal-related family, member 3 | 56543 | ENSMUSG00000040896 |
| 16 | 17487805 | NA | Atp1a3 | ATPase, Na+/K+ transporting, alpha 3 polypeptide | 232975 | ENSMUSG00000040907 |
| 17 | 17478181 | NA | Kcnc1 | potassium voltage gated channel, Shaw-related subfamily, member 1 | 16502 | ENSMUSG00000058975 |
| 18 | 17535607 | NA | Slc6a8 | solute carrier family 6 (neurotransmitter transporter, creatine), member 8 | 102857 | ENSMUSG00000019558 |
| 19 | 17271158 | NA | Cacng5 | calcium channel, voltage-dependent, gamma subunit 5 | 140723 | ENSMUSG00000040373 |
| 20 | 17471155 | NA | Kcna1 | potassium voltage-gated channel, shaker-related subfamily, member 1 | 16485 | ENSMUSG00000047976 |
| 21 | 17461868 | NA | Slc6a1 | solute carrier family 6 (neurotransmitter transporter, GABA), member 1 | 232333 | ENSMUSG00000030310 |
| 22 | 17406925 | NA | Hcn3 | hyperpolarization-activated, cyclic nucleotide-gated K+ 3 | 15168 | ENSMUSG00000028051 |
| 23 | 17473161 | NA | Cacng8 | calcium channel, voltage-dependent, gamma subunit 8 | 81905 | ENSMUSG00000053395 |
| 24 | 17409005 | NA | Slc6a17 | solute carrier family 6 (neurotransmitter transporter), member 17 | 229706 | ENSMUSG00000027894 |
| 25 | 17473155 | NA | Cacng7 | calcium channel, voltage-dependent, gamma subunit 7 | 81904 | ENSMUSG00000069806 |
| 26 | 17521327 | NA | Cacna2d2 | calcium channel, voltage-dependent, alpha 2/delta subunit 2 | 56808 | ENSMUSG00000010066 |
| 27 | 17477714 | NA | Slc17a7 | solute carrier family 17 (sodium-dependent inorganic phosphate cotransporter), member 7 | 72961 | ENSMUSG00000070570 |
| 28 | 17317801 | NA | Kcnk9 | potassium channel, subfamily K, member 9 | 223604 | ENSMUSG00000036760 |
| 29 | 17243162 | NA | Slc39a3 | solute carrier family 39 (zinc transporter), member 3 | 106947 | ENSMUSG00000046822 |
| 30 | 17516731 | NA | Scn2b | sodium channel, voltage-gated, type II, beta | 72821 | ENSMUSG00000070304 |
| 31 | 17314636 | NA | Cacnb3 | calcium channel, voltage-dependent, beta 3 subunit | 12297 | ENSMUSG00000003352 |
| 32 | 17237336 | NA | Kcnc2 | potassium voltage gated channel, Shaw-related subfamily, member 2 | 268345 | ENSMUSG00000035681 |
| 33 | 17234936 | NA | Hcn2 | hyperpolarization-activated, cyclic nucleotide-gated K+ 2 | 15166 | ENSMUSG00000020331 |
| 34 | 17313106 | NA | Cacna1i | calcium channel, voltage-dependent, alpha 1I subunit | 239556 | ENSMUSG00000022416 |

  
  

| **Database:molecular function      &nbspName:substrate-specific transmembrane transporter activity      &nbspID:GO:0022891** | | | | | | |
| --- | --- | --- | --- | --- | --- | --- |
| C=759; O=49; E=16.98; R=2.89; rawP=2.85e-11; adjP=2.88e-09 | | | | | | |
| Index | UserID | Value | Gene Symbol | Gene Name | EntrezGene | Ensembl |
| 1 | 17417702 | NA | Slc6a9 | solute carrier family 6 (neurotransmitter transporter, glycine), member 9 | 14664 | ENSMUSG00000028542 |
| 2 | 17318923 | NA | Cacng2 | calcium channel, voltage-dependent, gamma subunit 2 | 12300 | ENSMUSG00000019146 |
| 3 | 17378922 | NA | Slc32a1 | solute carrier family 32 (GABA vesicular transporter), member 1 | 22348 | ENSMUSG00000037771 |
| 4 | 17314051 | NA | Panx2 | pannexin 2 | 406218 | ENSMUSG00000058441 |
| 5 | 17334545 | NA | Clcn7 | chloride channel 7 | 26373 | ENSMUSG00000036636 |
| 6 | 17308299 | NA | Slc39a14 | solute carrier family 39 (zinc transporter), member 14 | 213053 | ENSMUSG00000022094 |
| 7 | 17461852 | NA | Slc6a11 | solute carrier family 6 (neurotransmitter transporter, GABA), member 11 | 243616 | ENSMUSG00000030307 |
| 8 | 17503122 | NA | Cacna1a | calcium channel, voltage-dependent, P/Q type, alpha 1A subunit | 12286 | ENSMUSG00000034656 |
| 9 | 17474067 | NA | Slc8a2 | solute carrier family 8 (sodium/calcium exchanger), member 2 | 110891 | ENSMUSG00000030376 |
| 10 | 17382496 | NA | Grin1 | glutamate receptor, ionotropic, NMDA1 (zeta 1) | 14810 | ENSMUSG00000026959 |
| 11 | 17367921 | NA | Grin1 | glutamate receptor, ionotropic, NMDA1 (zeta 1) | 14810 | ENSMUSG00000026959 |
| 12 | 17377144 | NA | Slc24a3 | solute carrier family 24 (sodium/potassium/calcium exchanger), member 3 | 94249 | ENSMUSG00000063873 |
| 13 | 17347948 | NA | Kcnk12 | potassium channel, subfamily K, member 12 | 210741 | ENSMUSG00000050138 |
| 14 | 17451482 | NA | Svop | SV2 related protein | 68666 | ENSMUSG00000042078 |
| 15 | 17252995 | NA | Slc43a2 | solute carrier family 43, member 2 | 215113 | ENSMUSG00000038178 |
| 16 | 17446123 | NA | Kcnh2 | potassium voltage-gated channel, subfamily H (eag-related), member 2 | 16511 | ENSMUSG00000038319 |
| 17 | 17263011 | NA | Glra1 | glycine receptor, alpha 1 subunit | 14654 | ENSMUSG00000000263 |
| 18 | 17419587 | NA | Slc9a1 | solute carrier family 9 (sodium/hydrogen exchanger), member 1 | 20544 | ENSMUSG00000028854 |
| 19 | 17404570 | NA | Slc7a14 | solute carrier family 7 (cationic amino acid transporter, y+ system), member 14 | 241919 | ENSMUSG00000069072 |
| 20 | 17370883 | NA | Kcnj3 | potassium inwardly-rectifying channel, subfamily J, member 3 | 16519 | ENSMUSG00000026824 |
| 21 | 17469814 | NA | Atp2b2 | ATPase, Ca++ transporting, plasma membrane 2 | 11941 | ENSMUSG00000030302 |
| 22 | 17401394 | NA | Kcnd3 | potassium voltage-gated channel, Shal-related family, member 3 | 56543 | ENSMUSG00000040896 |
| 23 | 17487805 | NA | Atp1a3 | ATPase, Na+/K+ transporting, alpha 3 polypeptide | 232975 | ENSMUSG00000040907 |
| 24 | 17478181 | NA | Kcnc1 | potassium voltage gated channel, Shaw-related subfamily, member 1 | 16502 | ENSMUSG00000058975 |
| 25 | 17271158 | NA | Cacng5 | calcium channel, voltage-dependent, gamma subunit 5 | 140723 | ENSMUSG00000040373 |
| 26 | 17535607 | NA | Slc6a8 | solute carrier family 6 (neurotransmitter transporter, creatine), member 8 | 102857 | ENSMUSG00000019558 |
| 27 | 17452719 | NA | Abcb9 | ATP-binding cassette, sub-family B (MDR/TAP), member 9 | 56325 | ENSMUSG00000029408 |
| 28 | 17471155 | NA | Kcna1 | potassium voltage-gated channel, shaker-related subfamily, member 1 | 16485 | ENSMUSG00000047976 |
| 29 | 17407124 | NA | Chrnb2 | cholinergic receptor, nicotinic, beta polypeptide 2 (neuronal) | 11444 | ENSMUSG00000027950 |
| 30 | 17461868 | NA | Slc6a1 | solute carrier family 6 (neurotransmitter transporter, GABA), member 1 | 232333 | ENSMUSG00000030310 |
| 31 | 17406925 | NA | Hcn3 | hyperpolarization-activated, cyclic nucleotide-gated K+ 3 | 15168 | ENSMUSG00000028051 |
| 32 | 17473161 | NA | Cacng8 | calcium channel, voltage-dependent, gamma subunit 8 | 81905 | ENSMUSG00000053395 |
| 33 | 17498607 | NA | Mcoln1 | mucolipin 1 | 94178 | ENSMUSG00000004567 |
| 34 | 17249811 | NA | Slc36a1 | solute carrier family 36 (proton/amino acid symporter), member 1 | 215335 | ENSMUSG00000020261 |
| 35 | 17298874 | NA | Grid1 | glutamate receptor, ionotropic, delta 1 | 14803 | ENSMUSG00000041078 |
| 36 | 17409005 | NA | Slc6a17 | solute carrier family 6 (neurotransmitter transporter), member 17 | 229706 | ENSMUSG00000027894 |
| 37 | 17473155 | NA | Cacng7 | calcium channel, voltage-dependent, gamma subunit 7 | 81904 | ENSMUSG00000069806 |
| 38 | 17521327 | NA | Cacna2d2 | calcium channel, voltage-dependent, alpha 2/delta subunit 2 | 56808 | ENSMUSG00000010066 |
| 39 | 17433287 | NA | Slc45a1 | solute carrier family 45, member 1 | 242773 | ENSMUSG00000039838 |
| 40 | 17497904 | NA | Slc25a22 | solute carrier family 25 (mitochondrial carrier, glutamate), member 22 | 68267 | ENSMUSG00000019082 |
| 41 | 17501692 | NA | Atp13a1 | ATPase type 13A1 | 170759 | ENSMUSG00000031862 |
| 42 | 17477714 | NA | Slc17a7 | solute carrier family 17 (sodium-dependent inorganic phosphate cotransporter), member 7 | 72961 | ENSMUSG00000070570 |
| 43 | 17317801 | NA | Kcnk9 | potassium channel, subfamily K, member 9 | 223604 | ENSMUSG00000036760 |
| 44 | 17243162 | NA | Slc39a3 | solute carrier family 39 (zinc transporter), member 3 | 106947 | ENSMUSG00000046822 |
| 45 | 17454574 | NA | Ttyh3 | tweety homolog 3 (Drosophila) | 78339 | ENSMUSG00000036565 |
| 46 | 17516731 | NA | Scn2b | sodium channel, voltage-gated, type II, beta | 72821 | ENSMUSG00000070304 |
| 47 | 17314636 | NA | Cacnb3 | calcium channel, voltage-dependent, beta 3 subunit | 12297 | ENSMUSG00000003352 |
| 48 | 17237336 | NA | Kcnc2 | potassium voltage gated channel, Shaw-related subfamily, member 2 | 268345 | ENSMUSG00000035681 |
| 49 | 17234936 | NA | Hcn2 | hyperpolarization-activated, cyclic nucleotide-gated K+ 2 | 15166 | ENSMUSG00000020331 |
| 50 | 17313106 | NA | Cacna1i | calcium channel, voltage-dependent, alpha 1I subunit | 239556 | ENSMUSG00000022416 |

  
  

| **Database:molecular function      &nbspName:ion transmembrane transporter activity      &nbspID:GO:0015075** | | | | | | |
| --- | --- | --- | --- | --- | --- | --- |
| C=710; O=47; E=15.88; R=2.96; rawP=3.19e-11; adjP=2.88e-09 | | | | | | |
| Index | UserID | Value | Gene Symbol | Gene Name | EntrezGene | Ensembl |
| 1 | 17417702 | NA | Slc6a9 | solute carrier family 6 (neurotransmitter transporter, glycine), member 9 | 14664 | ENSMUSG00000028542 |
| 2 | 17318923 | NA | Cacng2 | calcium channel, voltage-dependent, gamma subunit 2 | 12300 | ENSMUSG00000019146 |
| 3 | 17378922 | NA | Slc32a1 | solute carrier family 32 (GABA vesicular transporter), member 1 | 22348 | ENSMUSG00000037771 |
| 4 | 17314051 | NA | Panx2 | pannexin 2 | 406218 | ENSMUSG00000058441 |
| 5 | 17334545 | NA | Clcn7 | chloride channel 7 | 26373 | ENSMUSG00000036636 |
| 6 | 17308299 | NA | Slc39a14 | solute carrier family 39 (zinc transporter), member 14 | 213053 | ENSMUSG00000022094 |
| 7 | 17461852 | NA | Slc6a11 | solute carrier family 6 (neurotransmitter transporter, GABA), member 11 | 243616 | ENSMUSG00000030307 |
| 8 | 17503122 | NA | Cacna1a | calcium channel, voltage-dependent, P/Q type, alpha 1A subunit | 12286 | ENSMUSG00000034656 |
| 9 | 17474067 | NA | Slc8a2 | solute carrier family 8 (sodium/calcium exchanger), member 2 | 110891 | ENSMUSG00000030376 |
| 10 | 17382496 | NA | Grin1 | glutamate receptor, ionotropic, NMDA1 (zeta 1) | 14810 | ENSMUSG00000026959 |
| 11 | 17367921 | NA | Grin1 | glutamate receptor, ionotropic, NMDA1 (zeta 1) | 14810 | ENSMUSG00000026959 |
| 12 | 17377144 | NA | Slc24a3 | solute carrier family 24 (sodium/potassium/calcium exchanger), member 3 | 94249 | ENSMUSG00000063873 |
| 13 | 17347948 | NA | Kcnk12 | potassium channel, subfamily K, member 12 | 210741 | ENSMUSG00000050138 |
| 14 | 17451482 | NA | Svop | SV2 related protein | 68666 | ENSMUSG00000042078 |
| 15 | 17252995 | NA | Slc43a2 | solute carrier family 43, member 2 | 215113 | ENSMUSG00000038178 |
| 16 | 17446123 | NA | Kcnh2 | potassium voltage-gated channel, subfamily H (eag-related), member 2 | 16511 | ENSMUSG00000038319 |
| 17 | 17263011 | NA | Glra1 | glycine receptor, alpha 1 subunit | 14654 | ENSMUSG00000000263 |
| 18 | 17419587 | NA | Slc9a1 | solute carrier family 9 (sodium/hydrogen exchanger), member 1 | 20544 | ENSMUSG00000028854 |
| 19 | 17404570 | NA | Slc7a14 | solute carrier family 7 (cationic amino acid transporter, y+ system), member 14 | 241919 | ENSMUSG00000069072 |
| 20 | 17370883 | NA | Kcnj3 | potassium inwardly-rectifying channel, subfamily J, member 3 | 16519 | ENSMUSG00000026824 |
| 21 | 17469814 | NA | Atp2b2 | ATPase, Ca++ transporting, plasma membrane 2 | 11941 | ENSMUSG00000030302 |
| 22 | 17401394 | NA | Kcnd3 | potassium voltage-gated channel, Shal-related family, member 3 | 56543 | ENSMUSG00000040896 |
| 23 | 17487805 | NA | Atp1a3 | ATPase, Na+/K+ transporting, alpha 3 polypeptide | 232975 | ENSMUSG00000040907 |
| 24 | 17478181 | NA | Kcnc1 | potassium voltage gated channel, Shaw-related subfamily, member 1 | 16502 | ENSMUSG00000058975 |
| 25 | 17271158 | NA | Cacng5 | calcium channel, voltage-dependent, gamma subunit 5 | 140723 | ENSMUSG00000040373 |
| 26 | 17535607 | NA | Slc6a8 | solute carrier family 6 (neurotransmitter transporter, creatine), member 8 | 102857 | ENSMUSG00000019558 |
| 27 | 17471155 | NA | Kcna1 | potassium voltage-gated channel, shaker-related subfamily, member 1 | 16485 | ENSMUSG00000047976 |
| 28 | 17407124 | NA | Chrnb2 | cholinergic receptor, nicotinic, beta polypeptide 2 (neuronal) | 11444 | ENSMUSG00000027950 |
| 29 | 17461868 | NA | Slc6a1 | solute carrier family 6 (neurotransmitter transporter, GABA), member 1 | 232333 | ENSMUSG00000030310 |
| 30 | 17406925 | NA | Hcn3 | hyperpolarization-activated, cyclic nucleotide-gated K+ 3 | 15168 | ENSMUSG00000028051 |
| 31 | 17473161 | NA | Cacng8 | calcium channel, voltage-dependent, gamma subunit 8 | 81905 | ENSMUSG00000053395 |
| 32 | 17498607 | NA | Mcoln1 | mucolipin 1 | 94178 | ENSMUSG00000004567 |
| 33 | 17249811 | NA | Slc36a1 | solute carrier family 36 (proton/amino acid symporter), member 1 | 215335 | ENSMUSG00000020261 |
| 34 | 17298874 | NA | Grid1 | glutamate receptor, ionotropic, delta 1 | 14803 | ENSMUSG00000041078 |
| 35 | 17409005 | NA | Slc6a17 | solute carrier family 6 (neurotransmitter transporter), member 17 | 229706 | ENSMUSG00000027894 |
| 36 | 17473155 | NA | Cacng7 | calcium channel, voltage-dependent, gamma subunit 7 | 81904 | ENSMUSG00000069806 |
| 37 | 17521327 | NA | Cacna2d2 | calcium channel, voltage-dependent, alpha 2/delta subunit 2 | 56808 | ENSMUSG00000010066 |
| 38 | 17497904 | NA | Slc25a22 | solute carrier family 25 (mitochondrial carrier, glutamate), member 22 | 68267 | ENSMUSG00000019082 |
| 39 | 17501692 | NA | Atp13a1 | ATPase type 13A1 | 170759 | ENSMUSG00000031862 |
| 40 | 17477714 | NA | Slc17a7 | solute carrier family 17 (sodium-dependent inorganic phosphate cotransporter), member 7 | 72961 | ENSMUSG00000070570 |
| 41 | 17317801 | NA | Kcnk9 | potassium channel, subfamily K, member 9 | 223604 | ENSMUSG00000036760 |
| 42 | 17243162 | NA | Slc39a3 | solute carrier family 39 (zinc transporter), member 3 | 106947 | ENSMUSG00000046822 |
| 43 | 17454574 | NA | Ttyh3 | tweety homolog 3 (Drosophila) | 78339 | ENSMUSG00000036565 |
| 44 | 17516731 | NA | Scn2b | sodium channel, voltage-gated, type II, beta | 72821 | ENSMUSG00000070304 |
| 45 | 17314636 | NA | Cacnb3 | calcium channel, voltage-dependent, beta 3 subunit | 12297 | ENSMUSG00000003352 |
| 46 | 17237336 | NA | Kcnc2 | potassium voltage gated channel, Shaw-related subfamily, member 2 | 268345 | ENSMUSG00000035681 |
| 47 | 17234936 | NA | Hcn2 | hyperpolarization-activated, cyclic nucleotide-gated K+ 2 | 15166 | ENSMUSG00000020331 |
| 48 | 17313106 | NA | Cacna1i | calcium channel, voltage-dependent, alpha 1I subunit | 239556 | ENSMUSG00000022416 |

  
  

| **Database:molecular function      &nbspName:inorganic cation transmembrane transporter activity      &nbspID:GO:0022890** | | | | | | |
| --- | --- | --- | --- | --- | --- | --- |
| C=405; O=34; E=9.06; R=3.75; rawP=4.13e-11; adjP=2.88e-09 | | | | | | |
| Index | UserID | Value | Gene Symbol | Gene Name | EntrezGene | Ensembl |
| 1 | 17417702 | NA | Slc6a9 | solute carrier family 6 (neurotransmitter transporter, glycine), member 9 | 14664 | ENSMUSG00000028542 |
| 2 | 17318923 | NA | Cacng2 | calcium channel, voltage-dependent, gamma subunit 2 | 12300 | ENSMUSG00000019146 |
| 3 | 17378922 | NA | Slc32a1 | solute carrier family 32 (GABA vesicular transporter), member 1 | 22348 | ENSMUSG00000037771 |
| 4 | 17308299 | NA | Slc39a14 | solute carrier family 39 (zinc transporter), member 14 | 213053 | ENSMUSG00000022094 |
| 5 | 17461852 | NA | Slc6a11 | solute carrier family 6 (neurotransmitter transporter, GABA), member 11 | 243616 | ENSMUSG00000030307 |
| 6 | 17503122 | NA | Cacna1a | calcium channel, voltage-dependent, P/Q type, alpha 1A subunit | 12286 | ENSMUSG00000034656 |
| 7 | 17474067 | NA | Slc8a2 | solute carrier family 8 (sodium/calcium exchanger), member 2 | 110891 | ENSMUSG00000030376 |
| 8 | 17382496 | NA | Grin1 | glutamate receptor, ionotropic, NMDA1 (zeta 1) | 14810 | ENSMUSG00000026959 |
| 9 | 17367921 | NA | Grin1 | glutamate receptor, ionotropic, NMDA1 (zeta 1) | 14810 | ENSMUSG00000026959 |
| 10 | 17377144 | NA | Slc24a3 | solute carrier family 24 (sodium/potassium/calcium exchanger), member 3 | 94249 | ENSMUSG00000063873 |
| 11 | 17446123 | NA | Kcnh2 | potassium voltage-gated channel, subfamily H (eag-related), member 2 | 16511 | ENSMUSG00000038319 |
| 12 | 17419587 | NA | Slc9a1 | solute carrier family 9 (sodium/hydrogen exchanger), member 1 | 20544 | ENSMUSG00000028854 |
| 13 | 17370883 | NA | Kcnj3 | potassium inwardly-rectifying channel, subfamily J, member 3 | 16519 | ENSMUSG00000026824 |
| 14 | 17469814 | NA | Atp2b2 | ATPase, Ca++ transporting, plasma membrane 2 | 11941 | ENSMUSG00000030302 |
| 15 | 17401394 | NA | Kcnd3 | potassium voltage-gated channel, Shal-related family, member 3 | 56543 | ENSMUSG00000040896 |
| 16 | 17487805 | NA | Atp1a3 | ATPase, Na+/K+ transporting, alpha 3 polypeptide | 232975 | ENSMUSG00000040907 |
| 17 | 17478181 | NA | Kcnc1 | potassium voltage gated channel, Shaw-related subfamily, member 1 | 16502 | ENSMUSG00000058975 |
| 18 | 17535607 | NA | Slc6a8 | solute carrier family 6 (neurotransmitter transporter, creatine), member 8 | 102857 | ENSMUSG00000019558 |
| 19 | 17271158 | NA | Cacng5 | calcium channel, voltage-dependent, gamma subunit 5 | 140723 | ENSMUSG00000040373 |
| 20 | 17471155 | NA | Kcna1 | potassium voltage-gated channel, shaker-related subfamily, member 1 | 16485 | ENSMUSG00000047976 |
| 21 | 17461868 | NA | Slc6a1 | solute carrier family 6 (neurotransmitter transporter, GABA), member 1 | 232333 | ENSMUSG00000030310 |
| 22 | 17406925 | NA | Hcn3 | hyperpolarization-activated, cyclic nucleotide-gated K+ 3 | 15168 | ENSMUSG00000028051 |
| 23 | 17473161 | NA | Cacng8 | calcium channel, voltage-dependent, gamma subunit 8 | 81905 | ENSMUSG00000053395 |
| 24 | 17249811 | NA | Slc36a1 | solute carrier family 36 (proton/amino acid symporter), member 1 | 215335 | ENSMUSG00000020261 |
| 25 | 17409005 | NA | Slc6a17 | solute carrier family 6 (neurotransmitter transporter), member 17 | 229706 | ENSMUSG00000027894 |
| 26 | 17473155 | NA | Cacng7 | calcium channel, voltage-dependent, gamma subunit 7 | 81904 | ENSMUSG00000069806 |
| 27 | 17521327 | NA | Cacna2d2 | calcium channel, voltage-dependent, alpha 2/delta subunit 2 | 56808 | ENSMUSG00000010066 |
| 28 | 17477714 | NA | Slc17a7 | solute carrier family 17 (sodium-dependent inorganic phosphate cotransporter), member 7 | 72961 | ENSMUSG00000070570 |
| 29 | 17317801 | NA | Kcnk9 | potassium channel, subfamily K, member 9 | 223604 | ENSMUSG00000036760 |
| 30 | 17243162 | NA | Slc39a3 | solute carrier family 39 (zinc transporter), member 3 | 106947 | ENSMUSG00000046822 |
| 31 | 17516731 | NA | Scn2b | sodium channel, voltage-gated, type II, beta | 72821 | ENSMUSG00000070304 |
| 32 | 17314636 | NA | Cacnb3 | calcium channel, voltage-dependent, beta 3 subunit | 12297 | ENSMUSG00000003352 |
| 33 | 17237336 | NA | Kcnc2 | potassium voltage gated channel, Shaw-related subfamily, member 2 | 268345 | ENSMUSG00000035681 |
| 34 | 17234936 | NA | Hcn2 | hyperpolarization-activated, cyclic nucleotide-gated K+ 2 | 15166 | ENSMUSG00000020331 |
| 35 | 17313106 | NA | Cacna1i | calcium channel, voltage-dependent, alpha 1I subunit | 239556 | ENSMUSG00000022416 |

  
  

| **Database:molecular function      &nbspName:substrate-specific transporter activity      &nbspID:GO:0022892** | | | | | | |
| --- | --- | --- | --- | --- | --- | --- |
| C=870; O=53; E=19.46; R=2.72; rawP=3.64e-11; adjP=2.88e-09 | | | | | | |
| Index | UserID | Value | Gene Symbol | Gene Name | EntrezGene | Ensembl |
| 1 | 17417702 | NA | Slc6a9 | solute carrier family 6 (neurotransmitter transporter, glycine), member 9 | 14664 | ENSMUSG00000028542 |
| 2 | 17318923 | NA | Cacng2 | calcium channel, voltage-dependent, gamma subunit 2 | 12300 | ENSMUSG00000019146 |
| 3 | 17378922 | NA | Slc32a1 | solute carrier family 32 (GABA vesicular transporter), member 1 | 22348 | ENSMUSG00000037771 |
| 4 | 17314051 | NA | Panx2 | pannexin 2 | 406218 | ENSMUSG00000058441 |
| 5 | 17334545 | NA | Clcn7 | chloride channel 7 | 26373 | ENSMUSG00000036636 |
| 6 | 17308299 | NA | Slc39a14 | solute carrier family 39 (zinc transporter), member 14 | 213053 | ENSMUSG00000022094 |
| 7 | 17461852 | NA | Slc6a11 | solute carrier family 6 (neurotransmitter transporter, GABA), member 11 | 243616 | ENSMUSG00000030307 |
| 8 | 17535627 | NA | Abcd1 | ATP-binding cassette, sub-family D (ALD), member 1 | 11666 | ENSMUSG00000031378 |
| 9 | 17503122 | NA | Cacna1a | calcium channel, voltage-dependent, P/Q type, alpha 1A subunit | 12286 | ENSMUSG00000034656 |
| 10 | 17474067 | NA | Slc8a2 | solute carrier family 8 (sodium/calcium exchanger), member 2 | 110891 | ENSMUSG00000030376 |
| 11 | 17382496 | NA | Grin1 | glutamate receptor, ionotropic, NMDA1 (zeta 1) | 14810 | ENSMUSG00000026959 |
| 12 | 17367921 | NA | Grin1 | glutamate receptor, ionotropic, NMDA1 (zeta 1) | 14810 | ENSMUSG00000026959 |
| 13 | 17377144 | NA | Slc24a3 | solute carrier family 24 (sodium/potassium/calcium exchanger), member 3 | 94249 | ENSMUSG00000063873 |
| 14 | 17347948 | NA | Kcnk12 | potassium channel, subfamily K, member 12 | 210741 | ENSMUSG00000050138 |
| 15 | 17451482 | NA | Svop | SV2 related protein | 68666 | ENSMUSG00000042078 |
| 16 | 17252995 | NA | Slc43a2 | solute carrier family 43, member 2 | 215113 | ENSMUSG00000038178 |
| 17 | 17243057 | NA | Ap3d1 | adaptor-related protein complex 3, delta 1 subunit | 11776 | ENSMUSG00000020198 |
| 18 | 17446123 | NA | Kcnh2 | potassium voltage-gated channel, subfamily H (eag-related), member 2 | 16511 | ENSMUSG00000038319 |
| 19 | 17263011 | NA | Glra1 | glycine receptor, alpha 1 subunit | 14654 | ENSMUSG00000000263 |
| 20 | 17419587 | NA | Slc9a1 | solute carrier family 9 (sodium/hydrogen exchanger), member 1 | 20544 | ENSMUSG00000028854 |
| 21 | 17404570 | NA | Slc7a14 | solute carrier family 7 (cationic amino acid transporter, y+ system), member 14 | 241919 | ENSMUSG00000069072 |
| 22 | 17370883 | NA | Kcnj3 | potassium inwardly-rectifying channel, subfamily J, member 3 | 16519 | ENSMUSG00000026824 |
| 23 | 17469814 | NA | Atp2b2 | ATPase, Ca++ transporting, plasma membrane 2 | 11941 | ENSMUSG00000030302 |
| 24 | 17401394 | NA | Kcnd3 | potassium voltage-gated channel, Shal-related family, member 3 | 56543 | ENSMUSG00000040896 |
| 25 | 17487805 | NA | Atp1a3 | ATPase, Na+/K+ transporting, alpha 3 polypeptide | 232975 | ENSMUSG00000040907 |
| 26 | 17478181 | NA | Kcnc1 | potassium voltage gated channel, Shaw-related subfamily, member 1 | 16502 | ENSMUSG00000058975 |
| 27 | 17271158 | NA | Cacng5 | calcium channel, voltage-dependent, gamma subunit 5 | 140723 | ENSMUSG00000040373 |
| 28 | 17535607 | NA | Slc6a8 | solute carrier family 6 (neurotransmitter transporter, creatine), member 8 | 102857 | ENSMUSG00000019558 |
| 29 | 17452719 | NA | Abcb9 | ATP-binding cassette, sub-family B (MDR/TAP), member 9 | 56325 | ENSMUSG00000029408 |
| 30 | 17471155 | NA | Kcna1 | potassium voltage-gated channel, shaker-related subfamily, member 1 | 16485 | ENSMUSG00000047976 |
| 31 | 17407124 | NA | Chrnb2 | cholinergic receptor, nicotinic, beta polypeptide 2 (neuronal) | 11444 | ENSMUSG00000027950 |
| 32 | 17461868 | NA | Slc6a1 | solute carrier family 6 (neurotransmitter transporter, GABA), member 1 | 232333 | ENSMUSG00000030310 |
| 33 | 17406925 | NA | Hcn3 | hyperpolarization-activated, cyclic nucleotide-gated K+ 3 | 15168 | ENSMUSG00000028051 |
| 34 | 17473161 | NA | Cacng8 | calcium channel, voltage-dependent, gamma subunit 8 | 81905 | ENSMUSG00000053395 |
| 35 | 17498607 | NA | Mcoln1 | mucolipin 1 | 94178 | ENSMUSG00000004567 |
| 36 | 17249811 | NA | Slc36a1 | solute carrier family 36 (proton/amino acid symporter), member 1 | 215335 | ENSMUSG00000020261 |
| 37 | 17503333 | NA | Tnpo2 | transportin 2 (importin 3, karyopherin beta 2b) | 212999 | ENSMUSG00000031691 |
| 38 | 17298874 | NA | Grid1 | glutamate receptor, ionotropic, delta 1 | 14803 | ENSMUSG00000041078 |
| 39 | 17409005 | NA | Slc6a17 | solute carrier family 6 (neurotransmitter transporter), member 17 | 229706 | ENSMUSG00000027894 |
| 40 | 17521327 | NA | Cacna2d2 | calcium channel, voltage-dependent, alpha 2/delta subunit 2 | 56808 | ENSMUSG00000010066 |
| 41 | 17473155 | NA | Cacng7 | calcium channel, voltage-dependent, gamma subunit 7 | 81904 | ENSMUSG00000069806 |
| 42 | 17433287 | NA | Slc45a1 | solute carrier family 45, member 1 | 242773 | ENSMUSG00000039838 |
| 43 | 17497904 | NA | Slc25a22 | solute carrier family 25 (mitochondrial carrier, glutamate), member 22 | 68267 | ENSMUSG00000019082 |
| 44 | 17501692 | NA | Atp13a1 | ATPase type 13A1 | 170759 | ENSMUSG00000031862 |
| 45 | 17477714 | NA | Slc17a7 | solute carrier family 17 (sodium-dependent inorganic phosphate cotransporter), member 7 | 72961 | ENSMUSG00000070570 |
| 46 | 17317801 | NA | Kcnk9 | potassium channel, subfamily K, member 9 | 223604 | ENSMUSG00000036760 |
| 47 | 17243162 | NA | Slc39a3 | solute carrier family 39 (zinc transporter), member 3 | 106947 | ENSMUSG00000046822 |
| 48 | 17454574 | NA | Ttyh3 | tweety homolog 3 (Drosophila) | 78339 | ENSMUSG00000036565 |
| 49 | 17516731 | NA | Scn2b | sodium channel, voltage-gated, type II, beta | 72821 | ENSMUSG00000070304 |
| 50 | 17314636 | NA | Cacnb3 | calcium channel, voltage-dependent, beta 3 subunit | 12297 | ENSMUSG00000003352 |
| 51 | 17237336 | NA | Kcnc2 | potassium voltage gated channel, Shaw-related subfamily, member 2 | 268345 | ENSMUSG00000035681 |
| 52 | 17234936 | NA | Hcn2 | hyperpolarization-activated, cyclic nucleotide-gated K+ 2 | 15166 | ENSMUSG00000020331 |
| 53 | 17490452 | NA | Ap2a1 | adaptor protein complex AP-2, alpha 1 subunit | 11771 | ENSMUSG00000060279 |
| 54 | 17313106 | NA | Cacna1i | calcium channel, voltage-dependent, alpha 1I subunit | 239556 | ENSMUSG00000022416 |

  
  

| **Database:molecular function      &nbspName:protein binding      &nbspID:GO:0005515** | | | | | | |
| --- | --- | --- | --- | --- | --- | --- |
| C=6076; O=201; E=135.91; R=1.48; rawP=4.30e-11; adjP=2.88e-09 | | | | | | |
| Index | UserID | Value | Gene Symbol | Gene Name | EntrezGene | Ensembl |
| 1 | 17318923 | NA | Cacng2 | calcium channel, voltage-dependent, gamma subunit 2 | 12300 | ENSMUSG00000019146 |
| 2 | 17309287 | NA | Pou4f1 | POU domain, class 4, transcription factor 1 | 18996 | ENSMUSG00000048349 |
| 3 | 17334545 | NA | Clcn7 | chloride channel 7 | 26373 | ENSMUSG00000036636 |
| 4 | 17439511 | NA | Prdm8 | PR domain containing 8 | 77630 | ENSMUSG00000035456 |
| 5 | 17354831 | NA | Ablim3 | actin binding LIM protein family, member 3 | 319713 | ENSMUSG00000032735 |
| 6 | 17436999 | NA | Crmp1 | collapsin response mediator protein 1 | 12933 | ENSMUSG00000029121 |
| 7 | 17399496 | NA | Adar | adenosine deaminase, RNA-specific | 56417 | ENSMUSG00000027951 |
| 8 | 17376685 | NA | Plcb1 | phospholipase C, beta 1 | 18795 | ENSMUSG00000051177 |
| 9 | 17329516 | NA | Fgf12 | fibroblast growth factor 12 | 14167 | ENSMUSG00000022523 |
| 10 | 17506808 | NA | Trim67 | tripartite motif-containing 67 | 330863 | ENSMUSG00000036913 |
| 11 | 17342065 | NA | Mapk8ip3 | mitogen-activated protein kinase 8 interacting protein 3 | 30957 | ENSMUSG00000024163 |
| 12 | 17406892 | NA | Rusc1 | RUN and SH3 domain containing 1 | 72296 | ENSMUSG00000041263 |
| 13 | 17226891 | NA | Cntn2 | contactin 2 | 21367 | ENSMUSG00000053024 |
| 14 | 17334275 | NA | Caskin1 | CASK interacting protein 1 | 268932 | ENSMUSG00000033597 |
| 15 | 17318523 | NA | Scrt1 | scratch homolog 1, zinc finger protein (Drosophila) | 170729 | ENSMUSG00000048385 |
| 16 | 17522338 | NA | Scap | SREBF chaperone | 235623 | ENSMUSG00000032485 |
| 17 | 17322600 | NA | Mgrn1 | mahogunin, ring finger 1 | 17237 | ENSMUSG00000022517 |
| 18 | 17416325 | NA | Dhcr24 | 24-dehydrocholesterol reductase | 74754 | ENSMUSG00000034926 |
| 19 | 17223283 | NA | Satb2 | special AT-rich sequence binding protein 2 | 212712 | ENSMUSG00000038331 |
| 20 | 17339313 | NA | Epb4.1l3 | erythrocyte protein band 4.1-like 3 | 13823 | ENSMUSG00000024044 |
| 21 | 17424279 | NA | Cntfr | ciliary neurotrophic factor receptor | 12804 | ENSMUSG00000028444 |
| 22 | 17368079 | NA | Fbxw5 | F-box and WD-40 domain protein 5 | 30839 | ENSMUSG00000015095 |
| 23 | 17370234 | NA | Dab2ip | disabled 2 interacting protein | 69601 | ENSMUSG00000026883 |
| 24 | 17361223 | NA | Adrbk1 | adrenergic receptor kinase, beta 1 | 110355 | ENSMUSG00000024858 |
| 25 | 17320813 | NA | Nell2 | NEL-like 2 (chicken) | 54003 | ENSMUSG00000022454 |
| 26 | 17469814 | NA | Atp2b2 | ATPase, Ca++ transporting, plasma membrane 2 | 11941 | ENSMUSG00000030302 |
| 27 | 17487249 | NA | Mark4 | MAP/microtubule affinity-regulating kinase 4 | 232944 | ENSMUSG00000030397 |
| 28 | 17292753 | NA | Gprin1 | G protein-regulated inducer of neurite outgrowth 1 | 26913 | ENSMUSG00000069227 |
| 29 | 17329074 | NA | Map6d1 | MAP6 domain containing 1 | 208158 | ENSMUSG00000041205 |
| 30 | 17314872 | NA | Smarcd1 | SWI/SNF related, matrix associated, actin dependent regulator of chromatin, subfamily d, member 1 | 83797 | ENSMUSG00000023018 |
| 31 | 17317327 | NA | Mtss1 | metastasis suppressor 1 | 211401 | ENSMUSG00000022353 |
| 32 | 17502039 | NA | Rab3a | RAB3A, member RAS oncogene family | 19339 | ENSMUSG00000031840 |
| 33 | 17242707 | NA | BC005764 | cDNA sequence BC005764 | 216152 | ENSMUSG00000035835 |
| 34 | 17307905 | NA | Dpysl2 | dihydropyrimidinase-like 2 | 12934 | ENSMUSG00000022048 |
| 35 | 17290259 | NA | Ucn3 | urocortin 3 | 83428 | ENSMUSG00000044988 |
| 36 | 17453430 | NA | Limk1 | LIM-domain containing, protein kinase | 16885 | ENSMUSG00000029674 |
| 37 | 17239234 | NA | Grm1 | glutamate receptor, metabotropic 1 | 14816 | ENSMUSG00000019828 |
| 38 | 17526175 | NA | Abcg4 | ATP-binding cassette, sub-family G (WHITE), member 4 | 192663 | ENSMUSG00000032131 |
| 39 | 17253276 | NA | Git1 | G protein-coupled receptor kinase-interactor 1 | 216963 | ENSMUSG00000011877 |
| 40 | 17430140 | NA | Ncdn | neurochondrin | 26562 | ENSMUSG00000028833 |
| 41 | 17236102 | NA | Btbd11 | BTB (POZ) domain containing 11 | 74007 | ENSMUSG00000020042 |
| 42 | 17452038 | NA | Dtx1 | deltex 1 homolog (Drosophila) | 14357 | ENSMUSG00000029603 |
| 43 | 17540501 | NA | Syn1 | synapsin I | 20964 | ENSMUSG00000037217 |
| 44 | 17298874 | NA | Grid1 | glutamate receptor, ionotropic, delta 1 | 14803 | ENSMUSG00000041078 |
| 45 | 17292011 | NA | Tfap2a | transcription factor AP-2, alpha | 21418 | ENSMUSG00000021359 |
| 46 | 17361494 | NA | Pacs1 | phosphofurin acidic cluster sorting protein 1 | 107975 | ENSMUSG00000024855 |
| 47 | 17444202 | NA | Foxk1 | forkhead box K1 | 17425 | ENSMUSG00000056493 |
| 48 | 17397645 | NA | Smad9 | SMAD family member 9 | 55994 | ENSMUSG00000027796 |
| 49 | 17319339 | NA | Npcd | neuronal pentraxin chromo domain | 504193 | ENSMUSG00000089837 ENSMUSG00000022421 |
| 50 | 17317801 | NA | Kcnk9 | potassium channel, subfamily K, member 9 | 223604 | ENSMUSG00000036760 |
| 51 | 17269439 | NA | Hap1 | huntingtin-associated protein 1 | 15114 | ENSMUSG00000006930 |
| 52 | 17532879 | NA | Syp | synaptophysin | 20977 | ENSMUSG00000031144 |
| 53 | 17215820 | NA | Gpc1 | glypican 1 | 14733 | ENSMUSG00000034220 |
| 54 | 17490452 | NA | Ap2a1 | adaptor protein complex AP-2, alpha 1 subunit | 11771 | ENSMUSG00000060279 |
| 55 | 17237915 | NA | Agap2 | ArfGAP with GTPase domain, ankyrin repeat and PH domain 2 | 216439 | ENSMUSG00000025422 |
| 56 | 17376993 | NA | Pcsk2 | proprotein convertase subtilisin/kexin type 2 | 18549 | ENSMUSG00000027419 |
| 57 | 17488312 | NA | Map3k10 | mitogen-activated protein kinase kinase kinase 10 | 269881 | ENSMUSG00000040390 |
| 58 | 17451140 | NA | Ulk1 | unc-51 like kinase 1 | 22241 | ENSMUSG00000029512 |
| 59 | 17477454 | NA | Syt3 | synaptotagmin III | 20981 | ENSMUSG00000030731 |
| 60 | 17368550 | NA | Rxra | retinoid X receptor alpha | 20181 | ENSMUSG00000015846 |
| 61 | 17452139 | NA | Rph3a | rabphilin 3A | 19894 | ENSMUSG00000029608 |
| 62 | 17337513 | NA | Gabbr1 | gamma-aminobutyric acid (GABA) B receptor, 1 | 54393 | ENSMUSG00000024462 |
| 63 | 17498906 | NA | Myo16 | myosin XVI | 244281 | ENSMUSG00000039057 |
| 64 | 17502603 | NA | Rasd2 | RASD family, member 2 | 75141 | ENSMUSG00000034472 |
| 65 | 17510145 | NA | Pik3r2 | phosphatidylinositol 3-kinase, regulatory subunit, polypeptide 2 (p85 beta) | 18709 | ENSMUSG00000031834 |
| 66 | 17235037 | NA | Arid3a | AT rich interactive domain 3A (BRIGHT-like) | 13496 | ENSMUSG00000019564 |
| 67 | 17489052 | NA | Aplp1 | amyloid beta (A4) precursor-like protein 1 | 11803 | ENSMUSG00000006651 |
| 68 | 17432808 | NA | Mfn2 | mitofusin 2 | 170731 | ENSMUSG00000029020 |
| 69 | 17504130 | NA | Cx3cl1 | chemokine (C-X3-C motif) ligand 1 | 20312 | ENSMUSG00000031778 |
| 70 | 17468364 | NA | Rab11fip5 | RAB11 family interacting protein 5 (class I) | 52055 | ENSMUSG00000051343 |
| 71 | 17382496 | NA | Grin1 | glutamate receptor, ionotropic, NMDA1 (zeta 1) | 14810 | ENSMUSG00000026959 |
| 72 | 17367921 | NA | Grin1 | glutamate receptor, ionotropic, NMDA1 (zeta 1) | 14810 | ENSMUSG00000026959 |
| 73 | 17245539 | NA | Srgap1 | SLIT-ROBO Rho GTPase activating protein 1 | 117600 | ENSMUSG00000020121 |
| 74 | 17335204 | NA | Anks1 | ankyrin repeat and SAM domain containing 1 | 224650 | ENSMUSG00000024219 |
| 75 | 17268849 | NA | Med24 | mediator complex subunit 24 | 23989 | ENSMUSG00000017210 |
| 76 | 17376167 | NA | Sirpa | signal-regulatory protein alpha | 19261 | ENSMUSG00000037902 |
| 77 | 17322700 | NA | Rbfox1 | RNA binding protein, fox-1 homolog (C. elegans) 1 | 268859 | ENSMUSG00000008658 |
| 78 | 17272147 | NA | Wbp2 | WW domain binding protein 2 | 22378 | ENSMUSG00000034341 |
| 79 | 17319207 | NA | Csnk1e | casein kinase 1, epsilon | 27373 | ENSMUSG00000022433 |
| 80 | 17269464 | NA | Jup | junction plakoglobin | 16480 | ENSMUSG00000001552 |
| 81 | 17418732 | NA | Dlgap3 | discs, large (Drosophila) homolog-associated protein 3 | 242667 | ENSMUSG00000042388 |
| 82 | 17211347 | NA | Tfap2b | transcription factor AP-2 beta | 21419 | ENSMUSG00000025927 |
| 83 | 17518007 | NA | Pkm | pyruvate kinase, muscle | 18746 | ENSMUSG00000032294 |
| 84 | 17259177 | NA | Rptor | regulatory associated protein of MTOR, complex 1 | 74370 | ENSMUSG00000025583 |
| 85 | 17435834 | NA | Dpysl5 | dihydropyrimidinase-like 5 | 65254 | ENSMUSG00000029168 |
| 86 | 17535572 | NA | Atp2b3 | ATPase, Ca++ transporting, plasma membrane 3 | 320707 | ENSMUSG00000031376 |
| 87 | 17385374 | NA | Nr4a2 | nuclear receptor subfamily 4, group A, member 2 | 18227 | ENSMUSG00000026826 |
| 88 | 17222625 | NA | Tgfbrap1 | transforming growth factor, beta receptor associated protein 1 | 73122 | ENSMUSG00000070939 |
| 89 | 17354299 | NA | Sema6a | sema domain, transmembrane domain (TM), and cytoplasmic domain, (semaphorin) 6A | 20358 | ENSMUSG00000019647 |
| 90 | 17453383 | NA | Clip2 | CAP-GLY domain containing linker protein 2 | 269713 | ENSMUSG00000063146 |
| 91 | 17262065 | NA | Cyfip2 | cytoplasmic FMR1 interacting protein 2 | 76884 | ENSMUSG00000020340 |
| 92 | 17532953 | NA | Tfe3 | transcription factor E3 | 209446 | ENSMUSG00000000134 |
| 93 | 17407124 | NA | Chrnb2 | cholinergic receptor, nicotinic, beta polypeptide 2 (neuronal) | 11444 | ENSMUSG00000027950 |
| 94 | 17474389 | NA | Mypop | Myb-related transcription factor, partner of profilin | 232934 | ENSMUSG00000048481 |
| 95 | 17425095 | NA | Gabbr2 | gamma-aminobutyric acid (GABA) B receptor, 2 | 242425 | ENSMUSG00000039809 |
| 96 | 17500005 | NA | Ank1 | ankyrin 1, erythroid | 11733 | ENSMUSG00000031543 |
| 97 | 17226757 | NA | Rassf5 | Ras association (RalGDS/AF-6) domain family member 5 | 54354 | ENSMUSG00000026430 |
| 98 | 17369305 | NA | Usp20 | ubiquitin specific peptidase 20 | 74270 | ENSMUSG00000026854 |
| 99 | 17408211 | NA | Pde4dip | phosphodiesterase 4D interacting protein (myomegalin) | 83679 | ENSMUSG00000038170 |
| 100 | 17335145 | NA | Pacsin1 | protein kinase C and casein kinase substrate in neurons 1 | 23969 | ENSMUSG00000040276 |
| 101 | 17243229 | NA | Atcay | ataxia, cerebellar, Cayman type homolog (human) | 16467 | ENSMUSG00000034958 |
| 102 | 17295987 | NA | Rgs7bp | regulator of G-protein signalling 7 binding protein | 52882 | ENSMUSG00000021719 |
| 103 | 17503884 | NA | Gnao1 | guanine nucleotide binding protein, alpha O | 14681 | ENSMUSG00000031748 |
| 104 | 17248691 | NA | Ebf1 | early B cell factor 1 | 13591 | ENSMUSG00000057098 |
| 105 | 17383858 | NA | Dnm1 | dynamin 1 | 13429 | ENSMUSG00000026825 |
| 106 | 17364665 | NA | Slit1 | slit homolog 1 (Drosophila) | 20562 | ENSMUSG00000025020 |
| 107 | 17515277 | NA | Smarca4 | SWI/SNF related, matrix associated, actin dependent regulator of chromatin, subfamily a, member 4 | 20586 | ENSMUSG00000032187 |
| 108 | 17490432 | NA | Med25 | mediator of RNA polymerase II transcription, subunit 25 homolog (yeast) | 75613 | ENSMUSG00000002968 |
| 109 | 17256579 | NA | Cntnap1 | contactin associated protein-like 1 | 53321 | ENSMUSG00000017167 |
| 110 | 17504399 | NA | Cdh5 | cadherin 5 | 12562 | ENSMUSG00000031871 |
| 111 | 17542695 | NA | G6pdx | glucose-6-phosphate dehydrogenase X-linked | 14381 | ENSMUSG00000031400 |
| 112 | 17477468 | NA | Lrrc4b | leucine rich repeat containing 4B | 272381 | ENSMUSG00000047085 |
| 113 | 17368685 | NA | Ralgds | ral guanine nucleotide dissociation stimulator | 19730 | ENSMUSG00000026821 |
| 114 | 17516837 | NA | Bace1 | beta-site APP cleaving enzyme 1 | 23821 | ENSMUSG00000032086 |
| 115 | 17548746 | NA | Bace1 | beta-site APP cleaving enzyme 1 | 23821 | ENSMUSG00000032086 |
| 116 | 17356924 | NA | Nrxn2 | neurexin II | 18190 | ENSMUSG00000033768 |
| 117 | 17234936 | NA | Hcn2 | hyperpolarization-activated, cyclic nucleotide-gated K+ 2 | 15166 | ENSMUSG00000020331 |
| 118 | 17313504 | NA | Srebf2 | sterol regulatory element binding factor 2 | 20788 | ENSMUSG00000022463 |
| 119 | 17272817 | NA | Rbfox3 | RNA binding protein, fox-1 homolog (C. elegans) 3 | 52897 | ENSMUSG00000025576 |
| 120 | 17267039 | NA | Lhx1 | LIM homeobox protein 1 | 16869 | ENSMUSG00000018698 |
| 121 | 17259810 | NA | Inpp5j | inositol polyphosphate 5-phosphatase J | 170835 | ENSMUSG00000034570 |
| 122 | 17515843 | NA | Kirrel3 | kin of IRRE like 3 (Drosophila) | 67703 | ENSMUSG00000032036 |
| 123 | 17235556 | NA | Zbtb7a | zinc finger and BTB domain containing 7a | 16969 | ENSMUSG00000035011 |
| 124 | 17516462 | NA | Thy1 | thymus cell antigen 1, theta | 21838 | ENSMUSG00000032011 |
| 125 | 17314051 | NA | Panx2 | pannexin 2 | 406218 | ENSMUSG00000058441 |
| 126 | 17461923 | NA | Syn2 | synapsin II | 20965 | ENSMUSG00000009394 |
| 127 | 17284065 | NA | Cdc42bpb | CDC42 binding protein kinase beta | 217866 | ENSMUSG00000021279 |
| 128 | 17238605 | NA | Itga7 | integrin alpha 7 | 16404 | ENSMUSG00000025348 |
| 129 | 17314164 | NA | Mapk8ip2 | mitogen-activated protein kinase 8 interacting protein 2 | 60597 | ENSMUSG00000022619 |
| 130 | 17503122 | NA | Cacna1a | calcium channel, voltage-dependent, P/Q type, alpha 1A subunit | 12286 | ENSMUSG00000034656 |
| 131 | 17328870 | NA | Zdhhc8 | zinc finger, DHHC domain containing 8 | 27801 | ENSMUSG00000060166 |
| 132 | 17270028 | NA | Mpp2 | membrane protein, palmitoylated 2 (MAGUK p55 subfamily member 2) | 50997 | ENSMUSG00000017314 |
| 133 | 17487884 | NA | Gsk3a | glycogen synthase kinase 3 alpha | 606496 | ENSMUSG00000057177 |
| 134 | 17319045 | NA | Elfn2 | leucine rich repeat and fibronectin type III, extracellular 2 | 207393 | ENSMUSG00000043460 |
| 135 | 17388261 | NA | Atg13 | autophagy related 13 | 51897 | ENSMUSG00000027244 |
| 136 | 17505623 | NA | Mtss1l | metastasis suppressor 1-like | 244654 | ENSMUSG00000033763 |
| 137 | 17509907 | NA | Gatad2a | GATA zinc finger domain containing 2A | 234366 | ENSMUSG00000036180 |
| 138 | 17540059 | NA | Porcn | porcupine homolog (Drosophila) | 53627 | ENSMUSG00000031169 |
| 139 | 17308413 | NA | Epb4.9 | erythrocyte protein band 4.9 | 13829 | ENSMUSG00000022099 |
| 140 | 17268120 | NA | Pdk2 | pyruvate dehydrogenase kinase, isoenzyme 2 | 18604 | ENSMUSG00000038967 |
| 141 | 17268786 | NA | Neurod2 | neurogenic differentiation 2 | 18013 | ENSMUSG00000038255 |
| 142 | 17517073 | NA | Drd2 | dopamine receptor D2 | 13489 | ENSMUSG00000032259 |
| 143 | 17425058 | NA | Coro2a | coronin, actin binding protein 2A | 107684 | ENSMUSG00000028337 |
| 144 | 17484419 | NA | Kndc1 | kinase non-catalytic C-lobe domain (KIND) containing 1 | 76484 | ENSMUSG00000066129 |
| 145 | 17461897 | NA | Atg7 | autophagy related 7 | 74244 | ENSMUSG00000030314 |
| 146 | 17542419 | NA | L1cam | L1 cell adhesion molecule | 16728 | ENSMUSG00000031391 |
| 147 | 17536720 | NA | Nlgn3 | neuroligin 3 | 245537 | ENSMUSG00000031302 |
| 148 | 17332495 | NA | Dscam | Down syndrome cell adhesion molecule | 13508 | ENSMUSG00000050272 |
| 149 | 17406925 | NA | Hcn3 | hyperpolarization-activated, cyclic nucleotide-gated K+ 3 | 15168 | ENSMUSG00000028051 |
| 150 | 17496763 | NA | Stx1b | syntaxin 1B | 56216 | ENSMUSG00000030806 |
| 151 | 17259344 | NA | Hgs | HGF-regulated tyrosine kinase substrate | 15239 | ENSMUSG00000025793 |
| 152 | 17338136 | NA | Cul7 | cullin 7 | 66515 | ENSMUSG00000038545 |
| 153 | 17441453 | NA | Nos1 | nitric oxide synthase 1, neuronal | 18125 | ENSMUSG00000029361 |
| 154 | 17257197 | NA | Mapt | microtubule-associated protein tau | 17762 | ENSMUSG00000018411 |
| 155 | 17436877 | NA | Ablim2 | actin-binding LIM protein 2 | 231148 | ENSMUSG00000029095 |
| 156 | 17485152 | NA | Brsk2 | BR serine/threonine kinase 2 | 75770 | ENSMUSG00000053046 |
| 157 | 17235300 | NA | Apc2 | adenomatosis polyposis coli 2 | 23805 | ENSMUSG00000020135 |
| 158 | 17523281 | NA | Trak1 | trafficking protein, kinesin binding 1 | 67095 | ENSMUSG00000032536 |
| 159 | 17314636 | NA | Cacnb3 | calcium channel, voltage-dependent, beta 3 subunit | 12297 | ENSMUSG00000003352 |
| 160 | 17420316 | NA | Rap1gap | Rap1 GTPase-activating protein | 110351 | ENSMUSG00000041351 |
| 161 | 17394079 | NA | Rims4 | regulating synaptic membrane exocytosis 4 | 241770 | ENSMUSG00000035226 |
| 162 | 17273714 | NA | Adcy3 | adenylate cyclase 3 | 104111 | ENSMUSG00000020654 |
| 163 | 17477946 | NA | Mamstr | MEF2 activating motif and SAP domain containing transcriptional regulator | 74490 | ENSMUSG00000042918 |
| 164 | 17384021 | NA | Stxbp1 | syntaxin binding protein 1 | 20910 | ENSMUSG00000026797 |
| 165 | 17491285 | NA | Ptpn5 | protein tyrosine phosphatase, non-receptor type 5 | 19259 | ENSMUSG00000030854 |
| 166 | 17520198 | NA | Rasgrf1 | RAS protein-specific guanine nucleotide-releasing factor 1 | 19417 | ENSMUSG00000032356 |
| 167 | 17432299 | NA | Dnajc16 | DnaJ (Hsp40) homolog, subfamily C, member 16 | 214063 | ENSMUSG00000040697 |
| 168 | 17535627 | NA | Abcd1 | ATP-binding cassette, sub-family D (ALD), member 1 | 11666 | ENSMUSG00000031378 |
| 169 | 17474157 | NA | Strn4 | striatin, calmodulin binding protein 4 | 97387 | ENSMUSG00000030374 |
| 170 | 17292346 | NA | Phf2 | PHD finger protein 2 | 18676 | ENSMUSG00000038025 |
| 171 | 17357959 | NA | Gnaq | guanine nucleotide binding protein, alpha q polypeptide | 14682 | ENSMUSG00000024639 |
| 172 | 17383320 | NA | Brd3 | bromodomain containing 3 | 67382 | ENSMUSG00000026918 |
| 173 | 17513806 | NA | Cbfa2t3 | core-binding factor, runt domain, alpha subunit 2, translocated to, 3 (human) | 12398 | ENSMUSG00000006362 |
| 174 | 17266452 | NA | Sarm1 | sterile alpha and HEAT/Armadillo motif containing 1 | 237868 | ENSMUSG00000050132 |
| 175 | 17346387 | NA | Mllt1 | myeloid/lymphoid or mixed-lineage leukemia (trithorax homolog, Drosophila); translocated to, 1 | 64144 | ENSMUSG00000024212 |
| 176 | 17368855 | NA | Rapgef1 | Rap guanine nucleotide exchange factor (GEF) 1 | 107746 | ENSMUSG00000039844 |
| 177 | 17271622 | NA | Cdc42ep4 | CDC42 effector protein (Rho GTPase binding) 4 | 56699 | ENSMUSG00000041598 |
| 178 | 17398785 | NA | Arhgef11 | Rho guanine nucleotide exchange factor (GEF) 11 | 213498 | ENSMUSG00000041977 |
| 179 | 17419587 | NA | Slc9a1 | solute carrier family 9 (sodium/hydrogen exchanger), member 1 | 20544 | ENSMUSG00000028854 |
| 180 | 17489713 | NA | Lrp3 | low density lipoprotein receptor-related protein 3 | 435965 | ENSMUSG00000001802 |
| 181 | 17361779 | NA | Scyl1 | SCY1-like 1 (S. cerevisiae) | 78891 | ENSMUSG00000024941 |
| 182 | 17234116 | NA | Ccdc6 | coiled-coil domain containing 6 | 76551 | ENSMUSG00000048701 |
| 183 | 17346155 | NA | Sema6b | sema domain, transmembrane domain (TM), and cytoplasmic domain, (semaphorin) 6B | 20359 | ENSMUSG00000001227 |
| 184 | 17383798 | NA | Fibcd1 | fibrinogen C domain containing 1 | 98970 | ENSMUSG00000026841 |
| 185 | 17496452 | NA | Taok2 | TAO kinase 2 | 381921 | ENSMUSG00000059981 |
| 186 | 17378721 | NA | Src | Rous sarcoma oncogene | 20779 | ENSMUSG00000027646 |
| 187 | 17517634 | NA | Sin3a | transcriptional regulator, SIN3A (yeast) | 20466 | ENSMUSG00000042557 |
| 188 | 17452719 | NA | Abcb9 | ATP-binding cassette, sub-family B (MDR/TAP), member 9 | 56325 | ENSMUSG00000029408 |
| 189 | 17399044 | NA | Smg5 | Smg-5 homolog, nonsense mediated mRNA decay factor (C. elegans) | 229512 | ENSMUSG00000001415 |
| 190 | 17450059 | NA | Sec31a | Sec31 homolog A (S. cerevisiae) | 69162 | ENSMUSG00000035325 |
| 191 | 17242729 | NA | Med16 | mediator complex subunit 16 | 216154 | ENSMUSG00000013833 |
| 192 | 17325206 | NA | Adcy5 | adenylate cyclase 5 | 224129 | ENSMUSG00000022840 |
| 193 | 17490312 | NA | Nr1h2 | nuclear receptor subfamily 1, group H, member 2 | 22260 | ENSMUSG00000060601 |
| 194 | 17264960 | NA | Fgf11 | fibroblast growth factor 11 | 14166 | ENSMUSG00000042826 |
| 195 | 17345542 | NA | Ppp2r5d | protein phosphatase 2, regulatory subunit B (B56), delta isoform | 21770 | ENSMUSG00000059409 |
| 196 | 17219519 | NA | Igsf8 | immunoglobulin superfamily, member 8 | 140559 | ENSMUSG00000038034 |
| 197 | 17359960 | NA | Gbf1 | golgi-specific brefeldin A-resistance factor 1 | 107338 | ENSMUSG00000025224 |
| 198 | 17336987 | NA | Bag6 | BCL2-associated athanogene 6 | 224727 | ENSMUSG00000024392 |
| 199 | 17338210 | NA | Trerf1 | transcriptional regulating factor 1 | 224829 | ENSMUSG00000064043 |
| 200 | 17227589 | NA | Lhx9 | LIM homeobox protein 9 | 16876 | ENSMUSG00000019230 |
| 201 | 17360440 | NA | Adra2a | adrenergic receptor, alpha 2a | 11551 | ENSMUSG00000033717 |
| 202 | 17260261 | NA | Camk2b | calcium/calmodulin-dependent protein kinase II, beta | 12323 | ENSMUSG00000057897 |
| 203 | 17345740 | NA | Foxp4 | forkhead box P4 | 74123 | ENSMUSG00000023991 |

  
  

| **Database:molecular function      &nbspName:transporter activity      &nbspID:GO:0005215** | | | | | | |
| --- | --- | --- | --- | --- | --- | --- |
| C=1044; O=58; E=23.35; R=2.48; rawP=1.50e-10; adjP=8.61e-09 | | | | | | |
| Index | UserID | Value | Gene Symbol | Gene Name | EntrezGene | Ensembl |
| 1 | 17417702 | NA | Slc6a9 | solute carrier family 6 (neurotransmitter transporter, glycine), member 9 | 14664 | ENSMUSG00000028542 |
| 2 | 17318923 | NA | Cacng2 | calcium channel, voltage-dependent, gamma subunit 2 | 12300 | ENSMUSG00000019146 |
| 3 | 17477454 | NA | Syt3 | synaptotagmin III | 20981 | ENSMUSG00000030731 |
| 4 | 17378922 | NA | Slc32a1 | solute carrier family 32 (GABA vesicular transporter), member 1 | 22348 | ENSMUSG00000037771 |
| 5 | 17452139 | NA | Rph3a | rabphilin 3A | 19894 | ENSMUSG00000029608 |
| 6 | 17314051 | NA | Panx2 | pannexin 2 | 406218 | ENSMUSG00000058441 |
| 7 | 17334545 | NA | Clcn7 | chloride channel 7 | 26373 | ENSMUSG00000036636 |
| 8 | 17308299 | NA | Slc39a14 | solute carrier family 39 (zinc transporter), member 14 | 213053 | ENSMUSG00000022094 |
| 9 | 17461852 | NA | Slc6a11 | solute carrier family 6 (neurotransmitter transporter, GABA), member 11 | 243616 | ENSMUSG00000030307 |
| 10 | 17535627 | NA | Abcd1 | ATP-binding cassette, sub-family D (ALD), member 1 | 11666 | ENSMUSG00000031378 |
| 11 | 17503122 | NA | Cacna1a | calcium channel, voltage-dependent, P/Q type, alpha 1A subunit | 12286 | ENSMUSG00000034656 |
| 12 | 17474067 | NA | Slc8a2 | solute carrier family 8 (sodium/calcium exchanger), member 2 | 110891 | ENSMUSG00000030376 |
| 13 | 17400521 | NA | Sv2a | synaptic vesicle glycoprotein 2 a | 64051 | ENSMUSG00000038486 |
| 14 | 17382496 | NA | Grin1 | glutamate receptor, ionotropic, NMDA1 (zeta 1) | 14810 | ENSMUSG00000026959 |
| 15 | 17367921 | NA | Grin1 | glutamate receptor, ionotropic, NMDA1 (zeta 1) | 14810 | ENSMUSG00000026959 |
| 16 | 17377144 | NA | Slc24a3 | solute carrier family 24 (sodium/potassium/calcium exchanger), member 3 | 94249 | ENSMUSG00000063873 |
| 17 | 17347948 | NA | Kcnk12 | potassium channel, subfamily K, member 12 | 210741 | ENSMUSG00000050138 |
| 18 | 17451482 | NA | Svop | SV2 related protein | 68666 | ENSMUSG00000042078 |
| 19 | 17252995 | NA | Slc43a2 | solute carrier family 43, member 2 | 215113 | ENSMUSG00000038178 |
| 20 | 17243057 | NA | Ap3d1 | adaptor-related protein complex 3, delta 1 subunit | 11776 | ENSMUSG00000020198 |
| 21 | 17446123 | NA | Kcnh2 | potassium voltage-gated channel, subfamily H (eag-related), member 2 | 16511 | ENSMUSG00000038319 |
| 22 | 17263011 | NA | Glra1 | glycine receptor, alpha 1 subunit | 14654 | ENSMUSG00000000263 |
| 23 | 17419587 | NA | Slc9a1 | solute carrier family 9 (sodium/hydrogen exchanger), member 1 | 20544 | ENSMUSG00000028854 |
| 24 | 17404570 | NA | Slc7a14 | solute carrier family 7 (cationic amino acid transporter, y+ system), member 14 | 241919 | ENSMUSG00000069072 |
| 25 | 17370883 | NA | Kcnj3 | potassium inwardly-rectifying channel, subfamily J, member 3 | 16519 | ENSMUSG00000026824 |
| 26 | 17469814 | NA | Atp2b2 | ATPase, Ca++ transporting, plasma membrane 2 | 11941 | ENSMUSG00000030302 |
| 27 | 17401394 | NA | Kcnd3 | potassium voltage-gated channel, Shal-related family, member 3 | 56543 | ENSMUSG00000040896 |
| 28 | 17487805 | NA | Atp1a3 | ATPase, Na+/K+ transporting, alpha 3 polypeptide | 232975 | ENSMUSG00000040907 |
| 29 | 17478181 | NA | Kcnc1 | potassium voltage gated channel, Shaw-related subfamily, member 1 | 16502 | ENSMUSG00000058975 |
| 30 | 17271158 | NA | Cacng5 | calcium channel, voltage-dependent, gamma subunit 5 | 140723 | ENSMUSG00000040373 |
| 31 | 17535607 | NA | Slc6a8 | solute carrier family 6 (neurotransmitter transporter, creatine), member 8 | 102857 | ENSMUSG00000019558 |
| 32 | 17452719 | NA | Abcb9 | ATP-binding cassette, sub-family B (MDR/TAP), member 9 | 56325 | ENSMUSG00000029408 |
| 33 | 17471155 | NA | Kcna1 | potassium voltage-gated channel, shaker-related subfamily, member 1 | 16485 | ENSMUSG00000047976 |
| 34 | 17461868 | NA | Slc6a1 | solute carrier family 6 (neurotransmitter transporter, GABA), member 1 | 232333 | ENSMUSG00000030310 |
| 35 | 17407124 | NA | Chrnb2 | cholinergic receptor, nicotinic, beta polypeptide 2 (neuronal) | 11444 | ENSMUSG00000027950 |
| 36 | 17406925 | NA | Hcn3 | hyperpolarization-activated, cyclic nucleotide-gated K+ 3 | 15168 | ENSMUSG00000028051 |
| 37 | 17473161 | NA | Cacng8 | calcium channel, voltage-dependent, gamma subunit 8 | 81905 | ENSMUSG00000053395 |
| 38 | 17357486 | NA | Syt7 | synaptotagmin VII | 54525 | ENSMUSG00000024743 |
| 39 | 17498607 | NA | Mcoln1 | mucolipin 1 | 94178 | ENSMUSG00000004567 |
| 40 | 17249811 | NA | Slc36a1 | solute carrier family 36 (proton/amino acid symporter), member 1 | 215335 | ENSMUSG00000020261 |
| 41 | 17503333 | NA | Tnpo2 | transportin 2 (importin 3, karyopherin beta 2b) | 212999 | ENSMUSG00000031691 |
| 42 | 17298874 | NA | Grid1 | glutamate receptor, ionotropic, delta 1 | 14803 | ENSMUSG00000041078 |
| 43 | 17409005 | NA | Slc6a17 | solute carrier family 6 (neurotransmitter transporter), member 17 | 229706 | ENSMUSG00000027894 |
| 44 | 17521327 | NA | Cacna2d2 | calcium channel, voltage-dependent, alpha 2/delta subunit 2 | 56808 | ENSMUSG00000010066 |
| 45 | 17473155 | NA | Cacng7 | calcium channel, voltage-dependent, gamma subunit 7 | 81904 | ENSMUSG00000069806 |
| 46 | 17433287 | NA | Slc45a1 | solute carrier family 45, member 1 | 242773 | ENSMUSG00000039838 |
| 47 | 17497904 | NA | Slc25a22 | solute carrier family 25 (mitochondrial carrier, glutamate), member 22 | 68267 | ENSMUSG00000019082 |
| 48 | 17501692 | NA | Atp13a1 | ATPase type 13A1 | 170759 | ENSMUSG00000031862 |
| 49 | 17477714 | NA | Slc17a7 | solute carrier family 17 (sodium-dependent inorganic phosphate cotransporter), member 7 | 72961 | ENSMUSG00000070570 |
| 50 | 17317801 | NA | Kcnk9 | potassium channel, subfamily K, member 9 | 223604 | ENSMUSG00000036760 |
| 51 | 17243162 | NA | Slc39a3 | solute carrier family 39 (zinc transporter), member 3 | 106947 | ENSMUSG00000046822 |
| 52 | 17454574 | NA | Ttyh3 | tweety homolog 3 (Drosophila) | 78339 | ENSMUSG00000036565 |
| 53 | 17516731 | NA | Scn2b | sodium channel, voltage-gated, type II, beta | 72821 | ENSMUSG00000070304 |
| 54 | 17532879 | NA | Syp | synaptophysin | 20977 | ENSMUSG00000031144 |
| 55 | 17314636 | NA | Cacnb3 | calcium channel, voltage-dependent, beta 3 subunit | 12297 | ENSMUSG00000003352 |
| 56 | 17237336 | NA | Kcnc2 | potassium voltage gated channel, Shaw-related subfamily, member 2 | 268345 | ENSMUSG00000035681 |
| 57 | 17234936 | NA | Hcn2 | hyperpolarization-activated, cyclic nucleotide-gated K+ 2 | 15166 | ENSMUSG00000020331 |
| 58 | 17490452 | NA | Ap2a1 | adaptor protein complex AP-2, alpha 1 subunit | 11771 | ENSMUSG00000060279 |
| 59 | 17313106 | NA | Cacna1i | calcium channel, voltage-dependent, alpha 1I subunit | 239556 | ENSMUSG00000022416 |

  
  

| **Database:molecular function      &nbspName:transmembrane transporter activity      &nbspID:GO:0022857** | | | | | | |
| --- | --- | --- | --- | --- | --- | --- |
| C=836; O=50; E=18.70; R=2.67; rawP=2.60e-10; adjP=1.31e-08 | | | | | | |
| Index | UserID | Value | Gene Symbol | Gene Name | EntrezGene | Ensembl |
| 1 | 17417702 | NA | Slc6a9 | solute carrier family 6 (neurotransmitter transporter, glycine), member 9 | 14664 | ENSMUSG00000028542 |
| 2 | 17318923 | NA | Cacng2 | calcium channel, voltage-dependent, gamma subunit 2 | 12300 | ENSMUSG00000019146 |
| 3 | 17378922 | NA | Slc32a1 | solute carrier family 32 (GABA vesicular transporter), member 1 | 22348 | ENSMUSG00000037771 |
| 4 | 17314051 | NA | Panx2 | pannexin 2 | 406218 | ENSMUSG00000058441 |
| 5 | 17334545 | NA | Clcn7 | chloride channel 7 | 26373 | ENSMUSG00000036636 |
| 6 | 17308299 | NA | Slc39a14 | solute carrier family 39 (zinc transporter), member 14 | 213053 | ENSMUSG00000022094 |
| 7 | 17461852 | NA | Slc6a11 | solute carrier family 6 (neurotransmitter transporter, GABA), member 11 | 243616 | ENSMUSG00000030307 |
| 8 | 17503122 | NA | Cacna1a | calcium channel, voltage-dependent, P/Q type, alpha 1A subunit | 12286 | ENSMUSG00000034656 |
| 9 | 17474067 | NA | Slc8a2 | solute carrier family 8 (sodium/calcium exchanger), member 2 | 110891 | ENSMUSG00000030376 |
| 10 | 17400521 | NA | Sv2a | synaptic vesicle glycoprotein 2 a | 64051 | ENSMUSG00000038486 |
| 11 | 17382496 | NA | Grin1 | glutamate receptor, ionotropic, NMDA1 (zeta 1) | 14810 | ENSMUSG00000026959 |
| 12 | 17367921 | NA | Grin1 | glutamate receptor, ionotropic, NMDA1 (zeta 1) | 14810 | ENSMUSG00000026959 |
| 13 | 17377144 | NA | Slc24a3 | solute carrier family 24 (sodium/potassium/calcium exchanger), member 3 | 94249 | ENSMUSG00000063873 |
| 14 | 17347948 | NA | Kcnk12 | potassium channel, subfamily K, member 12 | 210741 | ENSMUSG00000050138 |
| 15 | 17451482 | NA | Svop | SV2 related protein | 68666 | ENSMUSG00000042078 |
| 16 | 17252995 | NA | Slc43a2 | solute carrier family 43, member 2 | 215113 | ENSMUSG00000038178 |
| 17 | 17446123 | NA | Kcnh2 | potassium voltage-gated channel, subfamily H (eag-related), member 2 | 16511 | ENSMUSG00000038319 |
| 18 | 17263011 | NA | Glra1 | glycine receptor, alpha 1 subunit | 14654 | ENSMUSG00000000263 |
| 19 | 17419587 | NA | Slc9a1 | solute carrier family 9 (sodium/hydrogen exchanger), member 1 | 20544 | ENSMUSG00000028854 |
| 20 | 17404570 | NA | Slc7a14 | solute carrier family 7 (cationic amino acid transporter, y+ system), member 14 | 241919 | ENSMUSG00000069072 |
| 21 | 17370883 | NA | Kcnj3 | potassium inwardly-rectifying channel, subfamily J, member 3 | 16519 | ENSMUSG00000026824 |
| 22 | 17469814 | NA | Atp2b2 | ATPase, Ca++ transporting, plasma membrane 2 | 11941 | ENSMUSG00000030302 |
| 23 | 17401394 | NA | Kcnd3 | potassium voltage-gated channel, Shal-related family, member 3 | 56543 | ENSMUSG00000040896 |
| 24 | 17487805 | NA | Atp1a3 | ATPase, Na+/K+ transporting, alpha 3 polypeptide | 232975 | ENSMUSG00000040907 |
| 25 | 17478181 | NA | Kcnc1 | potassium voltage gated channel, Shaw-related subfamily, member 1 | 16502 | ENSMUSG00000058975 |
| 26 | 17271158 | NA | Cacng5 | calcium channel, voltage-dependent, gamma subunit 5 | 140723 | ENSMUSG00000040373 |
| 27 | 17535607 | NA | Slc6a8 | solute carrier family 6 (neurotransmitter transporter, creatine), member 8 | 102857 | ENSMUSG00000019558 |
| 28 | 17452719 | NA | Abcb9 | ATP-binding cassette, sub-family B (MDR/TAP), member 9 | 56325 | ENSMUSG00000029408 |
| 29 | 17471155 | NA | Kcna1 | potassium voltage-gated channel, shaker-related subfamily, member 1 | 16485 | ENSMUSG00000047976 |
| 30 | 17407124 | NA | Chrnb2 | cholinergic receptor, nicotinic, beta polypeptide 2 (neuronal) | 11444 | ENSMUSG00000027950 |
| 31 | 17461868 | NA | Slc6a1 | solute carrier family 6 (neurotransmitter transporter, GABA), member 1 | 232333 | ENSMUSG00000030310 |
| 32 | 17406925 | NA | Hcn3 | hyperpolarization-activated, cyclic nucleotide-gated K+ 3 | 15168 | ENSMUSG00000028051 |
| 33 | 17473161 | NA | Cacng8 | calcium channel, voltage-dependent, gamma subunit 8 | 81905 | ENSMUSG00000053395 |
| 34 | 17498607 | NA | Mcoln1 | mucolipin 1 | 94178 | ENSMUSG00000004567 |
| 35 | 17249811 | NA | Slc36a1 | solute carrier family 36 (proton/amino acid symporter), member 1 | 215335 | ENSMUSG00000020261 |
| 36 | 17298874 | NA | Grid1 | glutamate receptor, ionotropic, delta 1 | 14803 | ENSMUSG00000041078 |
| 37 | 17409005 | NA | Slc6a17 | solute carrier family 6 (neurotransmitter transporter), member 17 | 229706 | ENSMUSG00000027894 |
| 38 | 17521327 | NA | Cacna2d2 | calcium channel, voltage-dependent, alpha 2/delta subunit 2 | 56808 | ENSMUSG00000010066 |
| 39 | 17473155 | NA | Cacng7 | calcium channel, voltage-dependent, gamma subunit 7 | 81904 | ENSMUSG00000069806 |
| 40 | 17433287 | NA | Slc45a1 | solute carrier family 45, member 1 | 242773 | ENSMUSG00000039838 |
| 41 | 17497904 | NA | Slc25a22 | solute carrier family 25 (mitochondrial carrier, glutamate), member 22 | 68267 | ENSMUSG00000019082 |
| 42 | 17501692 | NA | Atp13a1 | ATPase type 13A1 | 170759 | ENSMUSG00000031862 |
| 43 | 17477714 | NA | Slc17a7 | solute carrier family 17 (sodium-dependent inorganic phosphate cotransporter), member 7 | 72961 | ENSMUSG00000070570 |
| 44 | 17317801 | NA | Kcnk9 | potassium channel, subfamily K, member 9 | 223604 | ENSMUSG00000036760 |
| 45 | 17243162 | NA | Slc39a3 | solute carrier family 39 (zinc transporter), member 3 | 106947 | ENSMUSG00000046822 |
| 46 | 17454574 | NA | Ttyh3 | tweety homolog 3 (Drosophila) | 78339 | ENSMUSG00000036565 |
| 47 | 17516731 | NA | Scn2b | sodium channel, voltage-gated, type II, beta | 72821 | ENSMUSG00000070304 |
| 48 | 17314636 | NA | Cacnb3 | calcium channel, voltage-dependent, beta 3 subunit | 12297 | ENSMUSG00000003352 |
| 49 | 17237336 | NA | Kcnc2 | potassium voltage gated channel, Shaw-related subfamily, member 2 | 268345 | ENSMUSG00000035681 |
| 50 | 17234936 | NA | Hcn2 | hyperpolarization-activated, cyclic nucleotide-gated K+ 2 | 15166 | ENSMUSG00000020331 |
| 51 | 17313106 | NA | Cacna1i | calcium channel, voltage-dependent, alpha 1I subunit | 239556 | ENSMUSG00000022416 |

  
  

| **Database:molecular function      &nbspName:cation transmembrane transporter activity      &nbspID:GO:0008324** | | | | | | |
| --- | --- | --- | --- | --- | --- | --- |
| C=496; O=36; E=11.09; R=3.24; rawP=6.32e-10; adjP=2.82e-08 | | | | | | |
| Index | UserID | Value | Gene Symbol | Gene Name | EntrezGene | Ensembl |
| 1 | 17417702 | NA | Slc6a9 | solute carrier family 6 (neurotransmitter transporter, glycine), member 9 | 14664 | ENSMUSG00000028542 |
| 2 | 17318923 | NA | Cacng2 | calcium channel, voltage-dependent, gamma subunit 2 | 12300 | ENSMUSG00000019146 |
| 3 | 17378922 | NA | Slc32a1 | solute carrier family 32 (GABA vesicular transporter), member 1 | 22348 | ENSMUSG00000037771 |
| 4 | 17308299 | NA | Slc39a14 | solute carrier family 39 (zinc transporter), member 14 | 213053 | ENSMUSG00000022094 |
| 5 | 17461852 | NA | Slc6a11 | solute carrier family 6 (neurotransmitter transporter, GABA), member 11 | 243616 | ENSMUSG00000030307 |
| 6 | 17503122 | NA | Cacna1a | calcium channel, voltage-dependent, P/Q type, alpha 1A subunit | 12286 | ENSMUSG00000034656 |
| 7 | 17474067 | NA | Slc8a2 | solute carrier family 8 (sodium/calcium exchanger), member 2 | 110891 | ENSMUSG00000030376 |
| 8 | 17382496 | NA | Grin1 | glutamate receptor, ionotropic, NMDA1 (zeta 1) | 14810 | ENSMUSG00000026959 |
| 9 | 17367921 | NA | Grin1 | glutamate receptor, ionotropic, NMDA1 (zeta 1) | 14810 | ENSMUSG00000026959 |
| 10 | 17377144 | NA | Slc24a3 | solute carrier family 24 (sodium/potassium/calcium exchanger), member 3 | 94249 | ENSMUSG00000063873 |
| 11 | 17446123 | NA | Kcnh2 | potassium voltage-gated channel, subfamily H (eag-related), member 2 | 16511 | ENSMUSG00000038319 |
| 12 | 17419587 | NA | Slc9a1 | solute carrier family 9 (sodium/hydrogen exchanger), member 1 | 20544 | ENSMUSG00000028854 |
| 13 | 17370883 | NA | Kcnj3 | potassium inwardly-rectifying channel, subfamily J, member 3 | 16519 | ENSMUSG00000026824 |
| 14 | 17469814 | NA | Atp2b2 | ATPase, Ca++ transporting, plasma membrane 2 | 11941 | ENSMUSG00000030302 |
| 15 | 17401394 | NA | Kcnd3 | potassium voltage-gated channel, Shal-related family, member 3 | 56543 | ENSMUSG00000040896 |
| 16 | 17487805 | NA | Atp1a3 | ATPase, Na+/K+ transporting, alpha 3 polypeptide | 232975 | ENSMUSG00000040907 |
| 17 | 17478181 | NA | Kcnc1 | potassium voltage gated channel, Shaw-related subfamily, member 1 | 16502 | ENSMUSG00000058975 |
| 18 | 17535607 | NA | Slc6a8 | solute carrier family 6 (neurotransmitter transporter, creatine), member 8 | 102857 | ENSMUSG00000019558 |
| 19 | 17271158 | NA | Cacng5 | calcium channel, voltage-dependent, gamma subunit 5 | 140723 | ENSMUSG00000040373 |
| 20 | 17471155 | NA | Kcna1 | potassium voltage-gated channel, shaker-related subfamily, member 1 | 16485 | ENSMUSG00000047976 |
| 21 | 17407124 | NA | Chrnb2 | cholinergic receptor, nicotinic, beta polypeptide 2 (neuronal) | 11444 | ENSMUSG00000027950 |
| 22 | 17461868 | NA | Slc6a1 | solute carrier family 6 (neurotransmitter transporter, GABA), member 1 | 232333 | ENSMUSG00000030310 |
| 23 | 17406925 | NA | Hcn3 | hyperpolarization-activated, cyclic nucleotide-gated K+ 3 | 15168 | ENSMUSG00000028051 |
| 24 | 17473161 | NA | Cacng8 | calcium channel, voltage-dependent, gamma subunit 8 | 81905 | ENSMUSG00000053395 |
| 25 | 17498607 | NA | Mcoln1 | mucolipin 1 | 94178 | ENSMUSG00000004567 |
| 26 | 17249811 | NA | Slc36a1 | solute carrier family 36 (proton/amino acid symporter), member 1 | 215335 | ENSMUSG00000020261 |
| 27 | 17409005 | NA | Slc6a17 | solute carrier family 6 (neurotransmitter transporter), member 17 | 229706 | ENSMUSG00000027894 |
| 28 | 17473155 | NA | Cacng7 | calcium channel, voltage-dependent, gamma subunit 7 | 81904 | ENSMUSG00000069806 |
| 29 | 17521327 | NA | Cacna2d2 | calcium channel, voltage-dependent, alpha 2/delta subunit 2 | 56808 | ENSMUSG00000010066 |
| 30 | 17477714 | NA | Slc17a7 | solute carrier family 17 (sodium-dependent inorganic phosphate cotransporter), member 7 | 72961 | ENSMUSG00000070570 |
| 31 | 17317801 | NA | Kcnk9 | potassium channel, subfamily K, member 9 | 223604 | ENSMUSG00000036760 |
| 32 | 17243162 | NA | Slc39a3 | solute carrier family 39 (zinc transporter), member 3 | 106947 | ENSMUSG00000046822 |
| 33 | 17516731 | NA | Scn2b | sodium channel, voltage-gated, type II, beta | 72821 | ENSMUSG00000070304 |
| 34 | 17314636 | NA | Cacnb3 | calcium channel, voltage-dependent, beta 3 subunit | 12297 | ENSMUSG00000003352 |
| 35 | 17237336 | NA | Kcnc2 | potassium voltage gated channel, Shaw-related subfamily, member 2 | 268345 | ENSMUSG00000035681 |
| 36 | 17234936 | NA | Hcn2 | hyperpolarization-activated, cyclic nucleotide-gated K+ 2 | 15166 | ENSMUSG00000020331 |
| 37 | 17313106 | NA | Cacna1i | calcium channel, voltage-dependent, alpha 1I subunit | 239556 | ENSMUSG00000022416 |

  
  

| **Database:molecular function      &nbspName:voltage-gated channel activity      &nbspID:GO:0022832** | | | | | | |
| --- | --- | --- | --- | --- | --- | --- |
| C=166; O=20; E=3.71; R=5.39; rawP=1.01e-09; adjP=3.69e-08 | | | | | | |
| Index | UserID | Value | Gene Symbol | Gene Name | EntrezGene | Ensembl |
| 1 | 17318923 | NA | Cacng2 | calcium channel, voltage-dependent, gamma subunit 2 | 12300 | ENSMUSG00000019146 |
| 2 | 17478181 | NA | Kcnc1 | potassium voltage gated channel, Shaw-related subfamily, member 1 | 16502 | ENSMUSG00000058975 |
| 3 | 17271158 | NA | Cacng5 | calcium channel, voltage-dependent, gamma subunit 5 | 140723 | ENSMUSG00000040373 |
| 4 | 17334545 | NA | Clcn7 | chloride channel 7 | 26373 | ENSMUSG00000036636 |
| 5 | 17471155 | NA | Kcna1 | potassium voltage-gated channel, shaker-related subfamily, member 1 | 16485 | ENSMUSG00000047976 |
| 6 | 17406925 | NA | Hcn3 | hyperpolarization-activated, cyclic nucleotide-gated K+ 3 | 15168 | ENSMUSG00000028051 |
| 7 | 17473161 | NA | Cacng8 | calcium channel, voltage-dependent, gamma subunit 8 | 81905 | ENSMUSG00000053395 |
| 8 | 17503122 | NA | Cacna1a | calcium channel, voltage-dependent, P/Q type, alpha 1A subunit | 12286 | ENSMUSG00000034656 |
| 9 | 17382496 | NA | Grin1 | glutamate receptor, ionotropic, NMDA1 (zeta 1) | 14810 | ENSMUSG00000026959 |
| 10 | 17367921 | NA | Grin1 | glutamate receptor, ionotropic, NMDA1 (zeta 1) | 14810 | ENSMUSG00000026959 |
| 11 | 17473155 | NA | Cacng7 | calcium channel, voltage-dependent, gamma subunit 7 | 81904 | ENSMUSG00000069806 |
| 12 | 17521327 | NA | Cacna2d2 | calcium channel, voltage-dependent, alpha 2/delta subunit 2 | 56808 | ENSMUSG00000010066 |
| 13 | 17446123 | NA | Kcnh2 | potassium voltage-gated channel, subfamily H (eag-related), member 2 | 16511 | ENSMUSG00000038319 |
| 14 | 17317801 | NA | Kcnk9 | potassium channel, subfamily K, member 9 | 223604 | ENSMUSG00000036760 |
| 15 | 17516731 | NA | Scn2b | sodium channel, voltage-gated, type II, beta | 72821 | ENSMUSG00000070304 |
| 16 | 17370883 | NA | Kcnj3 | potassium inwardly-rectifying channel, subfamily J, member 3 | 16519 | ENSMUSG00000026824 |
| 17 | 17314636 | NA | Cacnb3 | calcium channel, voltage-dependent, beta 3 subunit | 12297 | ENSMUSG00000003352 |
| 18 | 17237336 | NA | Kcnc2 | potassium voltage gated channel, Shaw-related subfamily, member 2 | 268345 | ENSMUSG00000035681 |
| 19 | 17234936 | NA | Hcn2 | hyperpolarization-activated, cyclic nucleotide-gated K+ 2 | 15166 | ENSMUSG00000020331 |
| 20 | 17313106 | NA | Cacna1i | calcium channel, voltage-dependent, alpha 1I subunit | 239556 | ENSMUSG00000022416 |
| 21 | 17401394 | NA | Kcnd3 | potassium voltage-gated channel, Shal-related family, member 3 | 56543 | ENSMUSG00000040896 |

  
  

| **Database:cellular component      &nbspName:neuron projection      &nbspID:GO:0043005** | | | | | | |
| --- | --- | --- | --- | --- | --- | --- |
| C=779; O=79; E=17.63; R=4.48; rawP=8.55e-30; adjP=2.12e-27 | | | | | | |
| Index | UserID | Value | Gene Symbol | Gene Name | EntrezGene | Ensembl |
| 1 | 17309287 | NA | Pou4f1 | POU domain, class 4, transcription factor 1 | 18996 | ENSMUSG00000048349 |
| 2 | 17378922 | NA | Slc32a1 | solute carrier family 32 (GABA vesicular transporter), member 1 | 22348 | ENSMUSG00000037771 |
| 3 | 17346427 | NA | Khsrp | KH-type splicing regulatory protein | 16549 | ENSMUSG00000007670 |
| 4 | 17516462 | NA | Thy1 | thymus cell antigen 1, theta | 21838 | ENSMUSG00000032011 |
| 5 | 17342719 | NA | Grm4 | glutamate receptor, metabotropic 4 | 268934 | ENSMUSG00000063239 |
| 6 | 17314164 | NA | Mapk8ip2 | mitogen-activated protein kinase 8 interacting protein 2 | 60597 | ENSMUSG00000022619 |
| 7 | 17436999 | NA | Crmp1 | collapsin response mediator protein 1 | 12933 | ENSMUSG00000029121 |
| 8 | 17503122 | NA | Cacna1a | calcium channel, voltage-dependent, P/Q type, alpha 1A subunit | 12286 | ENSMUSG00000034656 |
| 9 | 17400521 | NA | Sv2a | synaptic vesicle glycoprotein 2 a | 64051 | ENSMUSG00000038486 |
| 10 | 17342065 | NA | Mapk8ip3 | mitogen-activated protein kinase 8 interacting protein 3 | 30957 | ENSMUSG00000024163 |
| 11 | 17226891 | NA | Cntn2 | contactin 2 | 21367 | ENSMUSG00000053024 |
| 12 | 17253376 | NA | Sez6 | seizure related gene 6 | 20370 | ENSMUSG00000000632 |
| 13 | 17339313 | NA | Epb4.1l3 | erythrocyte protein band 4.1-like 3 | 13823 | ENSMUSG00000024044 |
| 14 | 17484419 | NA | Kndc1 | kinase non-catalytic C-lobe domain (KIND) containing 1 | 76484 | ENSMUSG00000066129 |
| 15 | 17517073 | NA | Drd2 | dopamine receptor D2 | 13489 | ENSMUSG00000032259 |
| 16 | 17361223 | NA | Adrbk1 | adrenergic receptor kinase, beta 1 | 110355 | ENSMUSG00000024858 |
| 17 | 17320813 | NA | Nell2 | NEL-like 2 (chicken) | 54003 | ENSMUSG00000022454 |
| 18 | 17542419 | NA | L1cam | L1 cell adhesion molecule | 16728 | ENSMUSG00000031391 |
| 19 | 17469814 | NA | Atp2b2 | ATPase, Ca++ transporting, plasma membrane 2 | 11941 | ENSMUSG00000030302 |
| 20 | 17401394 | NA | Kcnd3 | potassium voltage-gated channel, Shal-related family, member 3 | 56543 | ENSMUSG00000040896 |
| 21 | 17487249 | NA | Mark4 | MAP/microtubule affinity-regulating kinase 4 | 232944 | ENSMUSG00000030397 |
| 22 | 17487805 | NA | Atp1a3 | ATPase, Na+/K+ transporting, alpha 3 polypeptide | 232975 | ENSMUSG00000040907 |
| 23 | 17478181 | NA | Kcnc1 | potassium voltage gated channel, Shaw-related subfamily, member 1 | 16502 | ENSMUSG00000058975 |
| 24 | 17271158 | NA | Cacng5 | calcium channel, voltage-dependent, gamma subunit 5 | 140723 | ENSMUSG00000040373 |
| 25 | 17332495 | NA | Dscam | Down syndrome cell adhesion molecule | 13508 | ENSMUSG00000050272 |
| 26 | 17307905 | NA | Dpysl2 | dihydropyrimidinase-like 2 | 12934 | ENSMUSG00000022048 |
| 27 | 17435570 | NA | Htr5a | 5-hydroxytryptamine (serotonin) receptor 5A | 15563 | ENSMUSG00000039106 |
| 28 | 17290259 | NA | Ucn3 | urocortin 3 | 83428 | ENSMUSG00000044988 |
| 29 | 17473161 | NA | Cacng8 | calcium channel, voltage-dependent, gamma subunit 8 | 81905 | ENSMUSG00000053395 |
| 30 | 17453430 | NA | Limk1 | LIM-domain containing, protein kinase | 16885 | ENSMUSG00000029674 |
| 31 | 17239234 | NA | Grm1 | glutamate receptor, metabotropic 1 | 14816 | ENSMUSG00000019828 |
| 32 | 17430140 | NA | Ncdn | neurochondrin | 26562 | ENSMUSG00000028833 |
| 33 | 17441453 | NA | Nos1 | nitric oxide synthase 1, neuronal | 18125 | ENSMUSG00000029361 |
| 34 | 17540501 | NA | Syn1 | synapsin I | 20964 | ENSMUSG00000037217 |
| 35 | 17300484 | NA | Cpne6 | copine VI | 12891 | ENSMUSG00000022212 |
| 36 | 17257197 | NA | Mapt | microtubule-associated protein tau | 17762 | ENSMUSG00000018411 |
| 37 | 17349607 | NA | Psd2 | pleckstrin and Sec7 domain containing 2 | 74002 | ENSMUSG00000024347 |
| 38 | 17477714 | NA | Slc17a7 | solute carrier family 17 (sodium-dependent inorganic phosphate cotransporter), member 7 | 72961 | ENSMUSG00000070570 |
| 39 | 17293045 | NA | Spock1 | sparc/osteonectin, cwcv and kazal-like domains proteoglycan 1 | 20745 | ENSMUSG00000056222 |
| 40 | 17319339 | NA | Npcd | neuronal pentraxin chromo domain | 504193 | ENSMUSG00000089837 ENSMUSG00000022421 |
| 41 | 17269439 | NA | Hap1 | huntingtin-associated protein 1 | 15114 | ENSMUSG00000006930 |
| 42 | 17532879 | NA | Syp | synaptophysin | 20977 | ENSMUSG00000031144 |
| 43 | 17535808 | NA | Gdi1 | guanosine diphosphate (GDP) dissociation inhibitor 1 | 14567 | ENSMUSG00000015291 |
| 44 | 17376993 | NA | Pcsk2 | proprotein convertase subtilisin/kexin type 2 | 18549 | ENSMUSG00000027419 |
| 45 | 17451140 | NA | Ulk1 | unc-51 like kinase 1 | 22241 | ENSMUSG00000029512 |
| 46 | 17368550 | NA | Rxra | retinoid X receptor alpha | 20181 | ENSMUSG00000015846 |
| 47 | 17452139 | NA | Rph3a | rabphilin 3A | 19894 | ENSMUSG00000029608 |
| 48 | 17520198 | NA | Rasgrf1 | RAS protein-specific guanine nucleotide-releasing factor 1 | 19417 | ENSMUSG00000032356 |
| 49 | 17342999 | NA | Cpne5 | copine V | 240058 | ENSMUSG00000024008 |
| 50 | 17337513 | NA | Gabbr1 | gamma-aminobutyric acid (GABA) B receptor, 1 | 54393 | ENSMUSG00000024462 |
| 51 | 17530733 | NA | Grm2 | glutamate receptor, metabotropic 2 | 108068 | ENSMUSG00000023192 |
| 52 | 17310772 | NA | Ctnnd2 | catenin (cadherin associated protein), delta 2 | 18163 | ENSMUSG00000022240 |
| 53 | 17474067 | NA | Slc8a2 | solute carrier family 8 (sodium/calcium exchanger), member 2 | 110891 | ENSMUSG00000030376 |
| 54 | 17474157 | NA | Strn4 | striatin, calmodulin binding protein 4 | 97387 | ENSMUSG00000030374 |
| 55 | 17382496 | NA | Grin1 | glutamate receptor, ionotropic, NMDA1 (zeta 1) | 14810 | ENSMUSG00000026959 |
| 56 | 17367921 | NA | Grin1 | glutamate receptor, ionotropic, NMDA1 (zeta 1) | 14810 | ENSMUSG00000026959 |
| 57 | 17279640 | NA | Ptprn2 | protein tyrosine phosphatase, receptor type, N polypeptide 2 | 19276 | ENSMUSG00000056553 |
| 58 | 17335204 | NA | Anks1 | ankyrin repeat and SAM domain containing 1 | 224650 | ENSMUSG00000024219 |
| 59 | 17243057 | NA | Ap3d1 | adaptor-related protein complex 3, delta 1 subunit | 11776 | ENSMUSG00000020198 |
| 60 | 17418732 | NA | Dlgap3 | discs, large (Drosophila) homolog-associated protein 3 | 242667 | ENSMUSG00000042388 |
| 61 | 17259177 | NA | Rptor | regulatory associated protein of MTOR, complex 1 | 74370 | ENSMUSG00000025583 |
| 62 | 17435834 | NA | Dpysl5 | dihydropyrimidinase-like 5 | 65254 | ENSMUSG00000029168 |
| 63 | 17354299 | NA | Sema6a | sema domain, transmembrane domain (TM), and cytoplasmic domain, (semaphorin) 6A | 20358 | ENSMUSG00000019647 |
| 64 | 17378721 | NA | Src | Rous sarcoma oncogene | 20779 | ENSMUSG00000027646 |
| 65 | 17471155 | NA | Kcna1 | potassium voltage-gated channel, shaker-related subfamily, member 1 | 16485 | ENSMUSG00000047976 |
| 66 | 17262065 | NA | Cyfip2 | cytoplasmic FMR1 interacting protein 2 | 76884 | ENSMUSG00000020340 |
| 67 | 17461868 | NA | Slc6a1 | solute carrier family 6 (neurotransmitter transporter, GABA), member 1 | 232333 | ENSMUSG00000030310 |
| 68 | 17500005 | NA | Ank1 | ankyrin 1, erythroid | 11733 | ENSMUSG00000031543 |
| 69 | 17425095 | NA | Gabbr2 | gamma-aminobutyric acid (GABA) B receptor, 2 | 242425 | ENSMUSG00000039809 |
| 70 | 17361454 | NA | Cnih2 | cornichon homolog 2 (Drosophila) | 12794 | ENSMUSG00000024873 |
| 71 | 17243229 | NA | Atcay | ataxia, cerebellar, Cayman type homolog (human) | 16467 | ENSMUSG00000034958 |
| 72 | 17503884 | NA | Gnao1 | guanine nucleotide binding protein, alpha O | 14681 | ENSMUSG00000031748 |
| 73 | 17361470 | NA | Klc2 | kinesin light chain 2 | 16594 | ENSMUSG00000024862 |
| 74 | 17256579 | NA | Cntnap1 | contactin associated protein-like 1 | 53321 | ENSMUSG00000017167 |
| 75 | 17516837 | NA | Bace1 | beta-site APP cleaving enzyme 1 | 23821 | ENSMUSG00000032086 |
| 76 | 17548746 | NA | Bace1 | beta-site APP cleaving enzyme 1 | 23821 | ENSMUSG00000032086 |
| 77 | 17234936 | NA | Hcn2 | hyperpolarization-activated, cyclic nucleotide-gated K+ 2 | 15166 | ENSMUSG00000020331 |
| 78 | 17313504 | NA | Srebf2 | sterol regulatory element binding factor 2 | 20788 | ENSMUSG00000022463 |
| 79 | 17259810 | NA | Inpp5j | inositol polyphosphate 5-phosphatase J | 170835 | ENSMUSG00000034570 |
| 80 | 17515843 | NA | Kirrel3 | kin of IRRE like 3 (Drosophila) | 67703 | ENSMUSG00000032036 |
| 81 | 17322359 | NA | Zfp385a | zinc finger protein 385A | 29813 | ENSMUSG00000000552 |

  
  

| **Database:cellular component      &nbspName:synapse      &nbspID:GO:0045202** | | | | | | |
| --- | --- | --- | --- | --- | --- | --- |
| C=548; O=56; E=12.40; R=4.51; rawP=2.41e-21; adjP=2.99e-19 | | | | | | |
| Index | UserID | Value | Gene Symbol | Gene Name | EntrezGene | Ensembl |
| 1 | 17477454 | NA | Syt3 | synaptotagmin III | 20981 | ENSMUSG00000030731 |
| 2 | 17378922 | NA | Slc32a1 | solute carrier family 32 (GABA vesicular transporter), member 1 | 22348 | ENSMUSG00000037771 |
| 3 | 17452139 | NA | Rph3a | rabphilin 3A | 19894 | ENSMUSG00000029608 |
| 4 | 17461923 | NA | Syn2 | synapsin II | 20965 | ENSMUSG00000009394 |
| 5 | 17342719 | NA | Grm4 | glutamate receptor, metabotropic 4 | 268934 | ENSMUSG00000063239 |
| 6 | 17337513 | NA | Gabbr1 | gamma-aminobutyric acid (GABA) B receptor, 1 | 54393 | ENSMUSG00000024462 |
| 7 | 17243717 | NA | Syn3 | synapsin III | 27204 | ENSMUSG00000059602 |
| 8 | 17314164 | NA | Mapk8ip2 | mitogen-activated protein kinase 8 interacting protein 2 | 60597 | ENSMUSG00000022619 |
| 9 | 17530733 | NA | Grm2 | glutamate receptor, metabotropic 2 | 108068 | ENSMUSG00000023192 |
| 10 | 17310772 | NA | Ctnnd2 | catenin (cadherin associated protein), delta 2 | 18163 | ENSMUSG00000022240 |
| 11 | 17400521 | NA | Sv2a | synaptic vesicle glycoprotein 2 a | 64051 | ENSMUSG00000038486 |
| 12 | 17308939 | NA | Pcdh8 | protocadherin 8 | 18530 | ENSMUSG00000036422 |
| 13 | 17382496 | NA | Grin1 | glutamate receptor, ionotropic, NMDA1 (zeta 1) | 14810 | ENSMUSG00000026959 |
| 14 | 17367921 | NA | Grin1 | glutamate receptor, ionotropic, NMDA1 (zeta 1) | 14810 | ENSMUSG00000026959 |
| 15 | 17406892 | NA | Rusc1 | RUN and SH3 domain containing 1 | 72296 | ENSMUSG00000041263 |
| 16 | 17475851 | NA | Lrfn1 | leucine rich repeat and fibronectin type III domain containing 1 | 80749 | NULL |
| 17 | 17226891 | NA | Cntn2 | contactin 2 | 21367 | ENSMUSG00000053024 |
| 18 | 17279640 | NA | Ptprn2 | protein tyrosine phosphatase, receptor type, N polypeptide 2 | 19276 | ENSMUSG00000056553 |
| 19 | 17451482 | NA | Svop | SV2 related protein | 68666 | ENSMUSG00000042078 |
| 20 | 17243057 | NA | Ap3d1 | adaptor-related protein complex 3, delta 1 subunit | 11776 | ENSMUSG00000020198 |
| 21 | 17253376 | NA | Sez6 | seizure related gene 6 | 20370 | ENSMUSG00000000632 |
| 22 | 17263011 | NA | Glra1 | glycine receptor, alpha 1 subunit | 14654 | ENSMUSG00000000263 |
| 23 | 17418732 | NA | Dlgap3 | discs, large (Drosophila) homolog-associated protein 3 | 242667 | ENSMUSG00000042388 |
| 24 | 17517073 | NA | Drd2 | dopamine receptor D2 | 13489 | ENSMUSG00000032259 |
| 25 | 17361223 | NA | Adrbk1 | adrenergic receptor kinase, beta 1 | 110355 | ENSMUSG00000024858 |
| 26 | 17542419 | NA | L1cam | L1 cell adhesion molecule | 16728 | ENSMUSG00000031391 |
| 27 | 17321582 | NA | Faim2 | Fas apoptotic inhibitory molecule 2 | 72393 | ENSMUSG00000023011 |
| 28 | 17378721 | NA | Src | Rous sarcoma oncogene | 20779 | ENSMUSG00000027646 |
| 29 | 17536720 | NA | Nlgn3 | neuroligin 3 | 245537 | ENSMUSG00000031302 |
| 30 | 17271158 | NA | Cacng5 | calcium channel, voltage-dependent, gamma subunit 5 | 140723 | ENSMUSG00000040373 |
| 31 | 17502039 | NA | Rab3a | RAB3A, member RAS oncogene family | 19339 | ENSMUSG00000031840 |
| 32 | 17262065 | NA | Cyfip2 | cytoplasmic FMR1 interacting protein 2 | 76884 | ENSMUSG00000020340 |
| 33 | 17407124 | NA | Chrnb2 | cholinergic receptor, nicotinic, beta polypeptide 2 (neuronal) | 11444 | ENSMUSG00000027950 |
| 34 | 17307905 | NA | Dpysl2 | dihydropyrimidinase-like 2 | 12934 | ENSMUSG00000022048 |
| 35 | 17425095 | NA | Gabbr2 | gamma-aminobutyric acid (GABA) B receptor, 2 | 242425 | ENSMUSG00000039809 |
| 36 | 17500005 | NA | Ank1 | ankyrin 1, erythroid | 11733 | ENSMUSG00000031543 |
| 37 | 17473161 | NA | Cacng8 | calcium channel, voltage-dependent, gamma subunit 8 | 81905 | ENSMUSG00000053395 |
| 38 | 17465332 | NA | Lrrc4 | leucine rich repeat containing 4 | 192198 | ENSMUSG00000049939 |
| 39 | 17239234 | NA | Grm1 | glutamate receptor, metabotropic 1 | 14816 | ENSMUSG00000019828 |
| 40 | 17361454 | NA | Cnih2 | cornichon homolog 2 (Drosophila) | 12794 | ENSMUSG00000024873 |
| 41 | 17335145 | NA | Pacsin1 | protein kinase C and casein kinase substrate in neurons 1 | 23969 | ENSMUSG00000040276 |
| 42 | 17357486 | NA | Syt7 | synaptotagmin VII | 54525 | ENSMUSG00000024743 |
| 43 | 17441453 | NA | Nos1 | nitric oxide synthase 1, neuronal | 18125 | ENSMUSG00000029361 |
| 44 | 17540501 | NA | Syn1 | synapsin I | 20964 | ENSMUSG00000037217 |
| 45 | 17298874 | NA | Grid1 | glutamate receptor, ionotropic, delta 1 | 14803 | ENSMUSG00000041078 |
| 46 | 17409005 | NA | Slc6a17 | solute carrier family 6 (neurotransmitter transporter), member 17 | 229706 | ENSMUSG00000027894 |
| 47 | 17477468 | NA | Lrrc4b | leucine rich repeat containing 4B | 272381 | ENSMUSG00000047085 |
| 48 | 17477714 | NA | Slc17a7 | solute carrier family 17 (sodium-dependent inorganic phosphate cotransporter), member 7 | 72961 | ENSMUSG00000070570 |
| 49 | 17293045 | NA | Spock1 | sparc/osteonectin, cwcv and kazal-like domains proteoglycan 1 | 20745 | ENSMUSG00000056222 |
| 50 | 17527520 | NA | Scamp5 | secretory carrier membrane protein 5 | 56807 | ENSMUSG00000040722 |
| 51 | 17317801 | NA | Kcnk9 | potassium channel, subfamily K, member 9 | 223604 | ENSMUSG00000036760 |
| 52 | 17529930 | NA | Clstn2 | calsyntenin 2 | 64085 | ENSMUSG00000032452 |
| 53 | 17532879 | NA | Syp | synaptophysin | 20977 | ENSMUSG00000031144 |
| 54 | 17356924 | NA | Nrxn2 | neurexin II | 18190 | ENSMUSG00000033768 |
| 55 | 17234936 | NA | Hcn2 | hyperpolarization-activated, cyclic nucleotide-gated K+ 2 | 15166 | ENSMUSG00000020331 |
| 56 | 17360440 | NA | Adra2a | adrenergic receptor, alpha 2a | 11551 | ENSMUSG00000033717 |
| 57 | 17394079 | NA | Rims4 | regulating synaptic membrane exocytosis 4 | 241770 | ENSMUSG00000035226 |

  
  

| **Database:cellular component      &nbspName:synapse part      &nbspID:GO:0044456** | | | | | | |
| --- | --- | --- | --- | --- | --- | --- |
| C=396; O=46; E=8.96; R=5.13; rawP=6.64e-20; adjP=4.35e-18 | | | | | | |
| Index | UserID | Value | Gene Symbol | Gene Name | EntrezGene | Ensembl |
| 1 | 17477454 | NA | Syt3 | synaptotagmin III | 20981 | ENSMUSG00000030731 |
| 2 | 17378922 | NA | Slc32a1 | solute carrier family 32 (GABA vesicular transporter), member 1 | 22348 | ENSMUSG00000037771 |
| 3 | 17452139 | NA | Rph3a | rabphilin 3A | 19894 | ENSMUSG00000029608 |
| 4 | 17461923 | NA | Syn2 | synapsin II | 20965 | ENSMUSG00000009394 |
| 5 | 17342719 | NA | Grm4 | glutamate receptor, metabotropic 4 | 268934 | ENSMUSG00000063239 |
| 6 | 17337513 | NA | Gabbr1 | gamma-aminobutyric acid (GABA) B receptor, 1 | 54393 | ENSMUSG00000024462 |
| 7 | 17243717 | NA | Syn3 | synapsin III | 27204 | ENSMUSG00000059602 |
| 8 | 17314164 | NA | Mapk8ip2 | mitogen-activated protein kinase 8 interacting protein 2 | 60597 | ENSMUSG00000022619 |
| 9 | 17530733 | NA | Grm2 | glutamate receptor, metabotropic 2 | 108068 | ENSMUSG00000023192 |
| 10 | 17310772 | NA | Ctnnd2 | catenin (cadherin associated protein), delta 2 | 18163 | ENSMUSG00000022240 |
| 11 | 17400521 | NA | Sv2a | synaptic vesicle glycoprotein 2 a | 64051 | ENSMUSG00000038486 |
| 12 | 17308939 | NA | Pcdh8 | protocadherin 8 | 18530 | ENSMUSG00000036422 |
| 13 | 17382496 | NA | Grin1 | glutamate receptor, ionotropic, NMDA1 (zeta 1) | 14810 | ENSMUSG00000026959 |
| 14 | 17367921 | NA | Grin1 | glutamate receptor, ionotropic, NMDA1 (zeta 1) | 14810 | ENSMUSG00000026959 |
| 15 | 17406892 | NA | Rusc1 | RUN and SH3 domain containing 1 | 72296 | ENSMUSG00000041263 |
| 16 | 17475851 | NA | Lrfn1 | leucine rich repeat and fibronectin type III domain containing 1 | 80749 | NULL |
| 17 | 17226891 | NA | Cntn2 | contactin 2 | 21367 | ENSMUSG00000053024 |
| 18 | 17279640 | NA | Ptprn2 | protein tyrosine phosphatase, receptor type, N polypeptide 2 | 19276 | ENSMUSG00000056553 |
| 19 | 17451482 | NA | Svop | SV2 related protein | 68666 | ENSMUSG00000042078 |
| 20 | 17243057 | NA | Ap3d1 | adaptor-related protein complex 3, delta 1 subunit | 11776 | ENSMUSG00000020198 |
| 21 | 17263011 | NA | Glra1 | glycine receptor, alpha 1 subunit | 14654 | ENSMUSG00000000263 |
| 22 | 17418732 | NA | Dlgap3 | discs, large (Drosophila) homolog-associated protein 3 | 242667 | ENSMUSG00000042388 |
| 23 | 17517073 | NA | Drd2 | dopamine receptor D2 | 13489 | ENSMUSG00000032259 |
| 24 | 17542419 | NA | L1cam | L1 cell adhesion molecule | 16728 | ENSMUSG00000031391 |
| 25 | 17321582 | NA | Faim2 | Fas apoptotic inhibitory molecule 2 | 72393 | ENSMUSG00000023011 |
| 26 | 17378721 | NA | Src | Rous sarcoma oncogene | 20779 | ENSMUSG00000027646 |
| 27 | 17271158 | NA | Cacng5 | calcium channel, voltage-dependent, gamma subunit 5 | 140723 | ENSMUSG00000040373 |
| 28 | 17502039 | NA | Rab3a | RAB3A, member RAS oncogene family | 19339 | ENSMUSG00000031840 |
| 29 | 17407124 | NA | Chrnb2 | cholinergic receptor, nicotinic, beta polypeptide 2 (neuronal) | 11444 | ENSMUSG00000027950 |
| 30 | 17307905 | NA | Dpysl2 | dihydropyrimidinase-like 2 | 12934 | ENSMUSG00000022048 |
| 31 | 17425095 | NA | Gabbr2 | gamma-aminobutyric acid (GABA) B receptor, 2 | 242425 | ENSMUSG00000039809 |
| 32 | 17500005 | NA | Ank1 | ankyrin 1, erythroid | 11733 | ENSMUSG00000031543 |
| 33 | 17473161 | NA | Cacng8 | calcium channel, voltage-dependent, gamma subunit 8 | 81905 | ENSMUSG00000053395 |
| 34 | 17465332 | NA | Lrrc4 | leucine rich repeat containing 4 | 192198 | ENSMUSG00000049939 |
| 35 | 17239234 | NA | Grm1 | glutamate receptor, metabotropic 1 | 14816 | ENSMUSG00000019828 |
| 36 | 17361454 | NA | Cnih2 | cornichon homolog 2 (Drosophila) | 12794 | ENSMUSG00000024873 |
| 37 | 17357486 | NA | Syt7 | synaptotagmin VII | 54525 | ENSMUSG00000024743 |
| 38 | 17441453 | NA | Nos1 | nitric oxide synthase 1, neuronal | 18125 | ENSMUSG00000029361 |
| 39 | 17540501 | NA | Syn1 | synapsin I | 20964 | ENSMUSG00000037217 |
| 40 | 17298874 | NA | Grid1 | glutamate receptor, ionotropic, delta 1 | 14803 | ENSMUSG00000041078 |
| 41 | 17477714 | NA | Slc17a7 | solute carrier family 17 (sodium-dependent inorganic phosphate cotransporter), member 7 | 72961 | ENSMUSG00000070570 |
| 42 | 17527520 | NA | Scamp5 | secretory carrier membrane protein 5 | 56807 | ENSMUSG00000040722 |
| 43 | 17317801 | NA | Kcnk9 | potassium channel, subfamily K, member 9 | 223604 | ENSMUSG00000036760 |
| 44 | 17529930 | NA | Clstn2 | calsyntenin 2 | 64085 | ENSMUSG00000032452 |
| 45 | 17532879 | NA | Syp | synaptophysin | 20977 | ENSMUSG00000031144 |
| 46 | 17356924 | NA | Nrxn2 | neurexin II | 18190 | ENSMUSG00000033768 |
| 47 | 17394079 | NA | Rims4 | regulating synaptic membrane exocytosis 4 | 241770 | ENSMUSG00000035226 |

  
  

| **Database:cellular component      &nbspName:cell projection      &nbspID:GO:0042995** | | | | | | |
| --- | --- | --- | --- | --- | --- | --- |
| C=1367; O=89; E=30.94; R=2.88; rawP=7.02e-20; adjP=4.35e-18 | | | | | | |
| Index | UserID | Value | Gene Symbol | Gene Name | EntrezGene | Ensembl |
| 1 | 17309287 | NA | Pou4f1 | POU domain, class 4, transcription factor 1 | 18996 | ENSMUSG00000048349 |
| 2 | 17378922 | NA | Slc32a1 | solute carrier family 32 (GABA vesicular transporter), member 1 | 22348 | ENSMUSG00000037771 |
| 3 | 17346427 | NA | Khsrp | KH-type splicing regulatory protein | 16549 | ENSMUSG00000007670 |
| 4 | 17516462 | NA | Thy1 | thymus cell antigen 1, theta | 21838 | ENSMUSG00000032011 |
| 5 | 17342719 | NA | Grm4 | glutamate receptor, metabotropic 4 | 268934 | ENSMUSG00000063239 |
| 6 | 17461852 | NA | Slc6a11 | solute carrier family 6 (neurotransmitter transporter, GABA), member 11 | 243616 | ENSMUSG00000030307 |
| 7 | 17314164 | NA | Mapk8ip2 | mitogen-activated protein kinase 8 interacting protein 2 | 60597 | ENSMUSG00000022619 |
| 8 | 17436999 | NA | Crmp1 | collapsin response mediator protein 1 | 12933 | ENSMUSG00000029121 |
| 9 | 17503122 | NA | Cacna1a | calcium channel, voltage-dependent, P/Q type, alpha 1A subunit | 12286 | ENSMUSG00000034656 |
| 10 | 17400521 | NA | Sv2a | synaptic vesicle glycoprotein 2 a | 64051 | ENSMUSG00000038486 |
| 11 | 17342065 | NA | Mapk8ip3 | mitogen-activated protein kinase 8 interacting protein 3 | 30957 | ENSMUSG00000024163 |
| 12 | 17308939 | NA | Pcdh8 | protocadherin 8 | 18530 | ENSMUSG00000036422 |
| 13 | 17226891 | NA | Cntn2 | contactin 2 | 21367 | ENSMUSG00000053024 |
| 14 | 17253376 | NA | Sez6 | seizure related gene 6 | 20370 | ENSMUSG00000000632 |
| 15 | 17339313 | NA | Epb4.1l3 | erythrocyte protein band 4.1-like 3 | 13823 | ENSMUSG00000024044 |
| 16 | 17484419 | NA | Kndc1 | kinase non-catalytic C-lobe domain (KIND) containing 1 | 76484 | ENSMUSG00000066129 |
| 17 | 17517073 | NA | Drd2 | dopamine receptor D2 | 13489 | ENSMUSG00000032259 |
| 18 | 17361223 | NA | Adrbk1 | adrenergic receptor kinase, beta 1 | 110355 | ENSMUSG00000024858 |
| 19 | 17320813 | NA | Nell2 | NEL-like 2 (chicken) | 54003 | ENSMUSG00000022454 |
| 20 | 17542419 | NA | L1cam | L1 cell adhesion molecule | 16728 | ENSMUSG00000031391 |
| 21 | 17469814 | NA | Atp2b2 | ATPase, Ca++ transporting, plasma membrane 2 | 11941 | ENSMUSG00000030302 |
| 22 | 17401394 | NA | Kcnd3 | potassium voltage-gated channel, Shal-related family, member 3 | 56543 | ENSMUSG00000040896 |
| 23 | 17487249 | NA | Mark4 | MAP/microtubule affinity-regulating kinase 4 | 232944 | ENSMUSG00000030397 |
| 24 | 17292753 | NA | Gprin1 | G protein-regulated inducer of neurite outgrowth 1 | 26913 | ENSMUSG00000069227 |
| 25 | 17487805 | NA | Atp1a3 | ATPase, Na+/K+ transporting, alpha 3 polypeptide | 232975 | ENSMUSG00000040907 |
| 26 | 17478181 | NA | Kcnc1 | potassium voltage gated channel, Shaw-related subfamily, member 1 | 16502 | ENSMUSG00000058975 |
| 27 | 17271158 | NA | Cacng5 | calcium channel, voltage-dependent, gamma subunit 5 | 140723 | ENSMUSG00000040373 |
| 28 | 17332495 | NA | Dscam | Down syndrome cell adhesion molecule | 13508 | ENSMUSG00000050272 |
| 29 | 17307905 | NA | Dpysl2 | dihydropyrimidinase-like 2 | 12934 | ENSMUSG00000022048 |
| 30 | 17435570 | NA | Htr5a | 5-hydroxytryptamine (serotonin) receptor 5A | 15563 | ENSMUSG00000039106 |
| 31 | 17290259 | NA | Ucn3 | urocortin 3 | 83428 | ENSMUSG00000044988 |
| 32 | 17473161 | NA | Cacng8 | calcium channel, voltage-dependent, gamma subunit 8 | 81905 | ENSMUSG00000053395 |
| 33 | 17453430 | NA | Limk1 | LIM-domain containing, protein kinase | 16885 | ENSMUSG00000029674 |
| 34 | 17239234 | NA | Grm1 | glutamate receptor, metabotropic 1 | 14816 | ENSMUSG00000019828 |
| 35 | 17430140 | NA | Ncdn | neurochondrin | 26562 | ENSMUSG00000028833 |
| 36 | 17441453 | NA | Nos1 | nitric oxide synthase 1, neuronal | 18125 | ENSMUSG00000029361 |
| 37 | 17540501 | NA | Syn1 | synapsin I | 20964 | ENSMUSG00000037217 |
| 38 | 17300484 | NA | Cpne6 | copine VI | 12891 | ENSMUSG00000022212 |
| 39 | 17257197 | NA | Mapt | microtubule-associated protein tau | 17762 | ENSMUSG00000018411 |
| 40 | 17349607 | NA | Psd2 | pleckstrin and Sec7 domain containing 2 | 74002 | ENSMUSG00000024347 |
| 41 | 17477714 | NA | Slc17a7 | solute carrier family 17 (sodium-dependent inorganic phosphate cotransporter), member 7 | 72961 | ENSMUSG00000070570 |
| 42 | 17293045 | NA | Spock1 | sparc/osteonectin, cwcv and kazal-like domains proteoglycan 1 | 20745 | ENSMUSG00000056222 |
| 43 | 17235300 | NA | Apc2 | adenomatosis polyposis coli 2 | 23805 | ENSMUSG00000020135 |
| 44 | 17319339 | NA | Npcd | neuronal pentraxin chromo domain | 504193 | ENSMUSG00000089837 ENSMUSG00000022421 |
| 45 | 17269439 | NA | Hap1 | huntingtin-associated protein 1 | 15114 | ENSMUSG00000006930 |
| 46 | 17532879 | NA | Syp | synaptophysin | 20977 | ENSMUSG00000031144 |
| 47 | 17535808 | NA | Gdi1 | guanosine diphosphate (GDP) dissociation inhibitor 1 | 14567 | ENSMUSG00000015291 |
| 48 | 17376993 | NA | Pcsk2 | proprotein convertase subtilisin/kexin type 2 | 18549 | ENSMUSG00000027419 |
| 49 | 17451140 | NA | Ulk1 | unc-51 like kinase 1 | 22241 | ENSMUSG00000029512 |
| 50 | 17368550 | NA | Rxra | retinoid X receptor alpha | 20181 | ENSMUSG00000015846 |
| 51 | 17452139 | NA | Rph3a | rabphilin 3A | 19894 | ENSMUSG00000029608 |
| 52 | 17520198 | NA | Rasgrf1 | RAS protein-specific guanine nucleotide-releasing factor 1 | 19417 | ENSMUSG00000032356 |
| 53 | 17342999 | NA | Cpne5 | copine V | 240058 | ENSMUSG00000024008 |
| 54 | 17337513 | NA | Gabbr1 | gamma-aminobutyric acid (GABA) B receptor, 1 | 54393 | ENSMUSG00000024462 |
| 55 | 17530733 | NA | Grm2 | glutamate receptor, metabotropic 2 | 108068 | ENSMUSG00000023192 |
| 56 | 17310772 | NA | Ctnnd2 | catenin (cadherin associated protein), delta 2 | 18163 | ENSMUSG00000022240 |
| 57 | 17474067 | NA | Slc8a2 | solute carrier family 8 (sodium/calcium exchanger), member 2 | 110891 | ENSMUSG00000030376 |
| 58 | 17465620 | NA | Podxl | podocalyxin-like | 27205 | ENSMUSG00000025608 |
| 59 | 17504130 | NA | Cx3cl1 | chemokine (C-X3-C motif) ligand 1 | 20312 | ENSMUSG00000031778 |
| 60 | 17474157 | NA | Strn4 | striatin, calmodulin binding protein 4 | 97387 | ENSMUSG00000030374 |
| 61 | 17382496 | NA | Grin1 | glutamate receptor, ionotropic, NMDA1 (zeta 1) | 14810 | ENSMUSG00000026959 |
| 62 | 17367921 | NA | Grin1 | glutamate receptor, ionotropic, NMDA1 (zeta 1) | 14810 | ENSMUSG00000026959 |
| 63 | 17279640 | NA | Ptprn2 | protein tyrosine phosphatase, receptor type, N polypeptide 2 | 19276 | ENSMUSG00000056553 |
| 64 | 17335204 | NA | Anks1 | ankyrin repeat and SAM domain containing 1 | 224650 | ENSMUSG00000024219 |
| 65 | 17243057 | NA | Ap3d1 | adaptor-related protein complex 3, delta 1 subunit | 11776 | ENSMUSG00000020198 |
| 66 | 17418732 | NA | Dlgap3 | discs, large (Drosophila) homolog-associated protein 3 | 242667 | ENSMUSG00000042388 |
| 67 | 17518007 | NA | Pkm | pyruvate kinase, muscle | 18746 | ENSMUSG00000032294 |
| 68 | 17259177 | NA | Rptor | regulatory associated protein of MTOR, complex 1 | 74370 | ENSMUSG00000025583 |
| 69 | 17435834 | NA | Dpysl5 | dihydropyrimidinase-like 5 | 65254 | ENSMUSG00000029168 |
| 70 | 17496452 | NA | Taok2 | TAO kinase 2 | 381921 | ENSMUSG00000059981 |
| 71 | 17354299 | NA | Sema6a | sema domain, transmembrane domain (TM), and cytoplasmic domain, (semaphorin) 6A | 20358 | ENSMUSG00000019647 |
| 72 | 17235663 | NA | Pip5k1c | phosphatidylinositol-4-phosphate 5-kinase, type 1 gamma | 18717 | ENSMUSG00000034902 |
| 73 | 17378721 | NA | Src | Rous sarcoma oncogene | 20779 | ENSMUSG00000027646 |
| 74 | 17471155 | NA | Kcna1 | potassium voltage-gated channel, shaker-related subfamily, member 1 | 16485 | ENSMUSG00000047976 |
| 75 | 17262065 | NA | Cyfip2 | cytoplasmic FMR1 interacting protein 2 | 76884 | ENSMUSG00000020340 |
| 76 | 17461868 | NA | Slc6a1 | solute carrier family 6 (neurotransmitter transporter, GABA), member 1 | 232333 | ENSMUSG00000030310 |
| 77 | 17425095 | NA | Gabbr2 | gamma-aminobutyric acid (GABA) B receptor, 2 | 242425 | ENSMUSG00000039809 |
| 78 | 17500005 | NA | Ank1 | ankyrin 1, erythroid | 11733 | ENSMUSG00000031543 |
| 79 | 17361454 | NA | Cnih2 | cornichon homolog 2 (Drosophila) | 12794 | ENSMUSG00000024873 |
| 80 | 17325206 | NA | Adcy5 | adenylate cyclase 5 | 224129 | ENSMUSG00000022840 |
| 81 | 17243229 | NA | Atcay | ataxia, cerebellar, Cayman type homolog (human) | 16467 | ENSMUSG00000034958 |
| 82 | 17503884 | NA | Gnao1 | guanine nucleotide binding protein, alpha O | 14681 | ENSMUSG00000031748 |
| 83 | 17361470 | NA | Klc2 | kinesin light chain 2 | 16594 | ENSMUSG00000024862 |
| 84 | 17256579 | NA | Cntnap1 | contactin associated protein-like 1 | 53321 | ENSMUSG00000017167 |
| 85 | 17516837 | NA | Bace1 | beta-site APP cleaving enzyme 1 | 23821 | ENSMUSG00000032086 |
| 86 | 17548746 | NA | Bace1 | beta-site APP cleaving enzyme 1 | 23821 | ENSMUSG00000032086 |
| 87 | 17234936 | NA | Hcn2 | hyperpolarization-activated, cyclic nucleotide-gated K+ 2 | 15166 | ENSMUSG00000020331 |
| 88 | 17313504 | NA | Srebf2 | sterol regulatory element binding factor 2 | 20788 | ENSMUSG00000022463 |
| 89 | 17259810 | NA | Inpp5j | inositol polyphosphate 5-phosphatase J | 170835 | ENSMUSG00000034570 |
| 90 | 17515843 | NA | Kirrel3 | kin of IRRE like 3 (Drosophila) | 67703 | ENSMUSG00000032036 |
| 91 | 17322359 | NA | Zfp385a | zinc finger protein 385A | 29813 | ENSMUSG00000000552 |

  
  

| **Database:cellular component      &nbspName:dendrite      &nbspID:GO:0030425** | | | | | | |
| --- | --- | --- | --- | --- | --- | --- |
| C=395; O=43; E=8.94; R=4.81; rawP=1.32e-17; adjP=6.55e-16 | | | | | | |
| Index | UserID | Value | Gene Symbol | Gene Name | EntrezGene | Ensembl |
| 1 | 17378922 | NA | Slc32a1 | solute carrier family 32 (GABA vesicular transporter), member 1 | 22348 | ENSMUSG00000037771 |
| 2 | 17346427 | NA | Khsrp | KH-type splicing regulatory protein | 16549 | ENSMUSG00000007670 |
| 3 | 17516462 | NA | Thy1 | thymus cell antigen 1, theta | 21838 | ENSMUSG00000032011 |
| 4 | 17342719 | NA | Grm4 | glutamate receptor, metabotropic 4 | 268934 | ENSMUSG00000063239 |
| 5 | 17337513 | NA | Gabbr1 | gamma-aminobutyric acid (GABA) B receptor, 1 | 54393 | ENSMUSG00000024462 |
| 6 | 17314164 | NA | Mapk8ip2 | mitogen-activated protein kinase 8 interacting protein 2 | 60597 | ENSMUSG00000022619 |
| 7 | 17436999 | NA | Crmp1 | collapsin response mediator protein 1 | 12933 | ENSMUSG00000029121 |
| 8 | 17503122 | NA | Cacna1a | calcium channel, voltage-dependent, P/Q type, alpha 1A subunit | 12286 | ENSMUSG00000034656 |
| 9 | 17310772 | NA | Ctnnd2 | catenin (cadherin associated protein), delta 2 | 18163 | ENSMUSG00000022240 |
| 10 | 17474067 | NA | Slc8a2 | solute carrier family 8 (sodium/calcium exchanger), member 2 | 110891 | ENSMUSG00000030376 |
| 11 | 17342065 | NA | Mapk8ip3 | mitogen-activated protein kinase 8 interacting protein 3 | 30957 | ENSMUSG00000024163 |
| 12 | 17474157 | NA | Strn4 | striatin, calmodulin binding protein 4 | 97387 | ENSMUSG00000030374 |
| 13 | 17382496 | NA | Grin1 | glutamate receptor, ionotropic, NMDA1 (zeta 1) | 14810 | ENSMUSG00000026959 |
| 14 | 17367921 | NA | Grin1 | glutamate receptor, ionotropic, NMDA1 (zeta 1) | 14810 | ENSMUSG00000026959 |
| 15 | 17253376 | NA | Sez6 | seizure related gene 6 | 20370 | ENSMUSG00000000632 |
| 16 | 17418732 | NA | Dlgap3 | discs, large (Drosophila) homolog-associated protein 3 | 242667 | ENSMUSG00000042388 |
| 17 | 17484419 | NA | Kndc1 | kinase non-catalytic C-lobe domain (KIND) containing 1 | 76484 | ENSMUSG00000066129 |
| 18 | 17517073 | NA | Drd2 | dopamine receptor D2 | 13489 | ENSMUSG00000032259 |
| 19 | 17361223 | NA | Adrbk1 | adrenergic receptor kinase, beta 1 | 110355 | ENSMUSG00000024858 |
| 20 | 17320813 | NA | Nell2 | NEL-like 2 (chicken) | 54003 | ENSMUSG00000022454 |
| 21 | 17259177 | NA | Rptor | regulatory associated protein of MTOR, complex 1 | 74370 | ENSMUSG00000025583 |
| 22 | 17435834 | NA | Dpysl5 | dihydropyrimidinase-like 5 | 65254 | ENSMUSG00000029168 |
| 23 | 17469814 | NA | Atp2b2 | ATPase, Ca++ transporting, plasma membrane 2 | 11941 | ENSMUSG00000030302 |
| 24 | 17378721 | NA | Src | Rous sarcoma oncogene | 20779 | ENSMUSG00000027646 |
| 25 | 17271158 | NA | Cacng5 | calcium channel, voltage-dependent, gamma subunit 5 | 140723 | ENSMUSG00000040373 |
| 26 | 17471155 | NA | Kcna1 | potassium voltage-gated channel, shaker-related subfamily, member 1 | 16485 | ENSMUSG00000047976 |
| 27 | 17307905 | NA | Dpysl2 | dihydropyrimidinase-like 2 | 12934 | ENSMUSG00000022048 |
| 28 | 17435570 | NA | Htr5a | 5-hydroxytryptamine (serotonin) receptor 5A | 15563 | ENSMUSG00000039106 |
| 29 | 17473161 | NA | Cacng8 | calcium channel, voltage-dependent, gamma subunit 8 | 81905 | ENSMUSG00000053395 |
| 30 | 17239234 | NA | Grm1 | glutamate receptor, metabotropic 1 | 14816 | ENSMUSG00000019828 |
| 31 | 17361454 | NA | Cnih2 | cornichon homolog 2 (Drosophila) | 12794 | ENSMUSG00000024873 |
| 32 | 17430140 | NA | Ncdn | neurochondrin | 26562 | ENSMUSG00000028833 |
| 33 | 17441453 | NA | Nos1 | nitric oxide synthase 1, neuronal | 18125 | ENSMUSG00000029361 |
| 34 | 17540501 | NA | Syn1 | synapsin I | 20964 | ENSMUSG00000037217 |
| 35 | 17349607 | NA | Psd2 | pleckstrin and Sec7 domain containing 2 | 74002 | ENSMUSG00000024347 |
| 36 | 17300484 | NA | Cpne6 | copine VI | 12891 | ENSMUSG00000022212 |
| 37 | 17319339 | NA | Npcd | neuronal pentraxin chromo domain | 504193 | ENSMUSG00000089837 ENSMUSG00000022421 |
| 38 | 17269439 | NA | Hap1 | huntingtin-associated protein 1 | 15114 | ENSMUSG00000006930 |
| 39 | 17234936 | NA | Hcn2 | hyperpolarization-activated, cyclic nucleotide-gated K+ 2 | 15166 | ENSMUSG00000020331 |
| 40 | 17313504 | NA | Srebf2 | sterol regulatory element binding factor 2 | 20788 | ENSMUSG00000022463 |
| 41 | 17259810 | NA | Inpp5j | inositol polyphosphate 5-phosphatase J | 170835 | ENSMUSG00000034570 |
| 42 | 17376993 | NA | Pcsk2 | proprotein convertase subtilisin/kexin type 2 | 18549 | ENSMUSG00000027419 |
| 43 | 17515843 | NA | Kirrel3 | kin of IRRE like 3 (Drosophila) | 67703 | ENSMUSG00000032036 |
| 44 | 17322359 | NA | Zfp385a | zinc finger protein 385A | 29813 | ENSMUSG00000000552 |

  
  

| **Database:cellular component      &nbspName:cell      &nbspID:GO:0005623** | | | | | | |
| --- | --- | --- | --- | --- | --- | --- |
| C=12714; O=363; E=287.79; R=1.26; rawP=3.28e-14; adjP=1.04e-12 | | | | | | |
| Index | UserID | Value | Gene Symbol | Gene Name | EntrezGene | Ensembl |
| 1 | 17378922 | NA | Slc32a1 | solute carrier family 32 (GABA vesicular transporter), member 1 | 22348 | ENSMUSG00000037771 |
| 2 | 17362646 | NA | Dagla | diacylglycerol lipase, alpha | 269060 | ENSMUSG00000035735 |
| 3 | 17439511 | NA | Prdm8 | PR domain containing 8 | 77630 | ENSMUSG00000035456 |
| 4 | 17461852 | NA | Slc6a11 | solute carrier family 6 (neurotransmitter transporter, GABA), member 11 | 243616 | ENSMUSG00000030307 |
| 5 | 17354831 | NA | Ablim3 | actin binding LIM protein family, member 3 | 319713 | ENSMUSG00000032735 |
| 6 | 17376685 | NA | Plcb1 | phospholipase C, beta 1 | 18795 | ENSMUSG00000051177 |
| 7 | 17418893 | NA | Trim62 | tripartite motif-containing 62 | 67525 | ENSMUSG00000041000 |
| 8 | 17372462 | NA | Zfp804a | zinc finger protein 804A | 241514 | ENSMUSG00000070866 |
| 9 | 17334275 | NA | Caskin1 | CASK interacting protein 1 | 268932 | ENSMUSG00000033597 |
| 10 | 17282420 | NA | Zfyve1 | zinc finger, FYVE domain containing 1 | 217695 | ENSMUSG00000042628 |
| 11 | 17252995 | NA | Slc43a2 | solute carrier family 43, member 2 | 215113 | ENSMUSG00000038178 |
| 12 | 17322600 | NA | Mgrn1 | mahogunin, ring finger 1 | 17237 | ENSMUSG00000022517 |
| 13 | 17462705 | NA | Foxj2 | forkhead box J2 | 60611 | ENSMUSG00000003154 |
| 14 | 17223283 | NA | Satb2 | special AT-rich sequence binding protein 2 | 212712 | ENSMUSG00000038331 |
| 15 | 17339313 | NA | Epb4.1l3 | erythrocyte protein band 4.1-like 3 | 13823 | ENSMUSG00000024044 |
| 16 | 17368079 | NA | Fbxw5 | F-box and WD-40 domain protein 5 | 30839 | ENSMUSG00000015095 |
| 17 | 17370234 | NA | Dab2ip | disabled 2 interacting protein | 69601 | ENSMUSG00000026883 |
| 18 | 17320813 | NA | Nell2 | NEL-like 2 (chicken) | 54003 | ENSMUSG00000022454 |
| 19 | 17469814 | NA | Atp2b2 | ATPase, Ca++ transporting, plasma membrane 2 | 11941 | ENSMUSG00000030302 |
| 20 | 17487249 | NA | Mark4 | MAP/microtubule affinity-regulating kinase 4 | 232944 | ENSMUSG00000030397 |
| 21 | 17487805 | NA | Atp1a3 | ATPase, Na+/K+ transporting, alpha 3 polypeptide | 232975 | ENSMUSG00000040907 |
| 22 | 17329074 | NA | Map6d1 | MAP6 domain containing 1 | 208158 | ENSMUSG00000041205 |
| 23 | 17314872 | NA | Smarcd1 | SWI/SNF related, matrix associated, actin dependent regulator of chromatin, subfamily d, member 1 | 83797 | ENSMUSG00000023018 |
| 24 | 17317327 | NA | Mtss1 | metastasis suppressor 1 | 211401 | ENSMUSG00000022353 |
| 25 | 17502039 | NA | Rab3a | RAB3A, member RAS oncogene family | 19339 | ENSMUSG00000031840 |
| 26 | 17290259 | NA | Ucn3 | urocortin 3 | 83428 | ENSMUSG00000044988 |
| 27 | 17453430 | NA | Limk1 | LIM-domain containing, protein kinase | 16885 | ENSMUSG00000029674 |
| 28 | 17239234 | NA | Grm1 | glutamate receptor, metabotropic 1 | 14816 | ENSMUSG00000019828 |
| 29 | 17409343 | NA | 5330417C22Rik | RIKEN cDNA 5330417C22 gene | 229722 | ENSMUSG00000040412 |
| 30 | 17224146 | NA | March4 | membrane-associated ring finger (C3HC4) 4 | 381270 | ENSMUSG00000039372 |
| 31 | 17430140 | NA | Ncdn | neurochondrin | 26562 | ENSMUSG00000028833 |
| 32 | 17452038 | NA | Dtx1 | deltex 1 homolog (Drosophila) | 14357 | ENSMUSG00000029603 |
| 33 | 17540501 | NA | Syn1 | synapsin I | 20964 | ENSMUSG00000037217 |
| 34 | 17349607 | NA | Psd2 | pleckstrin and Sec7 domain containing 2 | 74002 | ENSMUSG00000024347 |
| 35 | 17298874 | NA | Grid1 | glutamate receptor, ionotropic, delta 1 | 14803 | ENSMUSG00000041078 |
| 36 | 17413436 | NA | Tmem8b | transmembrane protein 8B | 242409 | ENSMUSG00000078716 |
| 37 | 17431720 | NA | Alpl | alkaline phosphatase, liver/bone/kidney | 11647 | ENSMUSG00000028766 |
| 38 | 17473155 | NA | Cacng7 | calcium channel, voltage-dependent, gamma subunit 7 | 81904 | ENSMUSG00000069806 |
| 39 | 17319339 | NA | Npcd | neuronal pentraxin chromo domain | 504193 | ENSMUSG00000089837 ENSMUSG00000022421 |
| 40 | 17397645 | NA | Smad9 | SMAD family member 9 | 55994 | ENSMUSG00000027796 |
| 41 | 17444202 | NA | Foxk1 | forkhead box K1 | 17425 | ENSMUSG00000056493 |
| 42 | 17269439 | NA | Hap1 | huntingtin-associated protein 1 | 15114 | ENSMUSG00000006930 |
| 43 | 17483447 | NA | Setd1a | SET domain containing 1A | 233904 | ENSMUSG00000042308 |
| 44 | 17527520 | NA | Scamp5 | secretory carrier membrane protein 5 | 56807 | ENSMUSG00000040722 |
| 45 | 17317801 | NA | Kcnk9 | potassium channel, subfamily K, member 9 | 223604 | ENSMUSG00000036760 |
| 46 | 17215820 | NA | Gpc1 | glypican 1 | 14733 | ENSMUSG00000034220 |
| 47 | 17518342 | NA | Megf11 | multiple EGF-like-domains 11 | 214058 | ENSMUSG00000036466 |
| 48 | 17344034 | NA | Skiv2l | superkiller viralicidic activity 2-like (S. cerevisiae) | 108077 | ENSMUSG00000040356 |
| 49 | 17535808 | NA | Gdi1 | guanosine diphosphate (GDP) dissociation inhibitor 1 | 14567 | ENSMUSG00000015291 |
| 50 | 17484409 | NA | Gpr123 | G protein-coupled receptor 123 | 52389 | ENSMUSG00000025475 |
| 51 | 17373530 | NA | Syt13 | synaptotagmin XIII | 80976 | ENSMUSG00000027220 |
| 52 | 17279434 | NA | Pacs2 | phosphofurin acidic cluster sorting protein 2 | 217893 | ENSMUSG00000021143 |
| 53 | 17451140 | NA | Ulk1 | unc-51 like kinase 1 | 22241 | ENSMUSG00000029512 |
| 54 | 17477454 | NA | Syt3 | synaptotagmin III | 20981 | ENSMUSG00000030731 |
| 55 | 17545450 | NA | Gpr173 | G-protein coupled receptor 173 | 70771 | ENSMUSG00000056679 |
| 56 | 17368550 | NA | Rxra | retinoid X receptor alpha | 20181 | ENSMUSG00000015846 |
| 57 | 17452139 | NA | Rph3a | rabphilin 3A | 19894 | ENSMUSG00000029608 |
| 58 | 17342999 | NA | Cpne5 | copine V | 240058 | ENSMUSG00000024008 |
| 59 | 17337513 | NA | Gabbr1 | gamma-aminobutyric acid (GABA) B receptor, 1 | 54393 | ENSMUSG00000024462 |
| 60 | 17498906 | NA | Myo16 | myosin XVI | 244281 | ENSMUSG00000039057 |
| 61 | 17510145 | NA | Pik3r2 | phosphatidylinositol 3-kinase, regulatory subunit, polypeptide 2 (p85 beta) | 18709 | ENSMUSG00000031834 |
| 62 | 17264124 | NA | Arhgap44 | Rho GTPase activating protein 44 | 216831 | ENSMUSG00000033389 |
| 63 | 17310772 | NA | Ctnnd2 | catenin (cadherin associated protein), delta 2 | 18163 | ENSMUSG00000022240 |
| 64 | 17489052 | NA | Aplp1 | amyloid beta (A4) precursor-like protein 1 | 11803 | ENSMUSG00000006651 |
| 65 | 17432808 | NA | Mfn2 | mitofusin 2 | 170731 | ENSMUSG00000029020 |
| 66 | 17468364 | NA | Rab11fip5 | RAB11 family interacting protein 5 (class I) | 52055 | ENSMUSG00000051343 |
| 67 | 17474400 | NA | Sympk | symplekin | 68188 | ENSMUSG00000023118 |
| 68 | 17214665 | NA | Sgpp2 | sphingosine-1-phosphate phosphotase 2 | 433323 | ENSMUSG00000032908 |
| 69 | 17335204 | NA | Anks1 | ankyrin repeat and SAM domain containing 1 | 224650 | ENSMUSG00000024219 |
| 70 | 17334205 | NA | Abca3 | ATP-binding cassette, sub-family A (ABC1), member 3 | 27410 | ENSMUSG00000024130 |
| 71 | 17322700 | NA | Rbfox1 | RNA binding protein, fox-1 homolog (C. elegans) 1 | 268859 | ENSMUSG00000008658 |
| 72 | 17446123 | NA | Kcnh2 | potassium voltage-gated channel, subfamily H (eag-related), member 2 | 16511 | ENSMUSG00000038319 |
| 73 | 17269464 | NA | Jup | junction plakoglobin | 16480 | ENSMUSG00000001552 |
| 74 | 17301823 | NA | Gfra2 | glial cell line derived neurotrophic factor family receptor alpha 2 | 14586 | ENSMUSG00000022103 |
| 75 | 17418732 | NA | Dlgap3 | discs, large (Drosophila) homolog-associated protein 3 | 242667 | ENSMUSG00000042388 |
| 76 | 17211347 | NA | Tfap2b | transcription factor AP-2 beta | 21419 | ENSMUSG00000025927 |
| 77 | 17259177 | NA | Rptor | regulatory associated protein of MTOR, complex 1 | 74370 | ENSMUSG00000025583 |
| 78 | 17435834 | NA | Dpysl5 | dihydropyrimidinase-like 5 | 65254 | ENSMUSG00000029168 |
| 79 | 17435528 | NA | Dpp6 | dipeptidylpeptidase 6 | 13483 | ENSMUSG00000061576 |
| 80 | 17354299 | NA | Sema6a | sema domain, transmembrane domain (TM), and cytoplasmic domain, (semaphorin) 6A | 20358 | ENSMUSG00000019647 |
| 81 | 17222625 | NA | Tgfbrap1 | transforming growth factor, beta receptor associated protein 1 | 73122 | ENSMUSG00000070939 |
| 82 | 17378242 | NA | Zfp341 | zinc finger protein 341 | 228807 | ENSMUSG00000059842 |
| 83 | 17535607 | NA | Slc6a8 | solute carrier family 6 (neurotransmitter transporter, creatine), member 8 | 102857 | ENSMUSG00000019558 |
| 84 | 17453383 | NA | Clip2 | CAP-GLY domain containing linker protein 2 | 269713 | ENSMUSG00000063146 |
| 85 | 17306666 | NA | Jph4 | junctophilin 4 | 319984 | ENSMUSG00000022208 |
| 86 | 17222465 | NA | Aff3 | AF4/FMR2 family, member 3 | 16764 | ENSMUSG00000037138 |
| 87 | 17407124 | NA | Chrnb2 | cholinergic receptor, nicotinic, beta polypeptide 2 (neuronal) | 11444 | ENSMUSG00000027950 |
| 88 | 17474389 | NA | Mypop | Myb-related transcription factor, partner of profilin | 232934 | ENSMUSG00000048481 |
| 89 | 17369305 | NA | Usp20 | ubiquitin specific peptidase 20 | 74270 | ENSMUSG00000026854 |
| 90 | 17226757 | NA | Rassf5 | Ras association (RalGDS/AF-6) domain family member 5 | 54354 | ENSMUSG00000026430 |
| 91 | 17500005 | NA | Ank1 | ankyrin 1, erythroid | 11733 | ENSMUSG00000031543 |
| 92 | 17361454 | NA | Cnih2 | cornichon homolog 2 (Drosophila) | 12794 | ENSMUSG00000024873 |
| 93 | 17440923 | NA | Ube3b | ubiquitin protein ligase E3B | 117146 | ENSMUSG00000029577 |
| 94 | 17357486 | NA | Syt7 | synaptotagmin VII | 54525 | ENSMUSG00000024743 |
| 95 | 17295987 | NA | Rgs7bp | regulator of G-protein signalling 7 binding protein | 52882 | ENSMUSG00000021719 |
| 96 | 17422138 | NA | Gpr153 | G protein-coupled receptor 153 | 100129 | ENSMUSG00000042804 |
| 97 | 17503333 | NA | Tnpo2 | transportin 2 (importin 3, karyopherin beta 2b) | 212999 | ENSMUSG00000031691 |
| 98 | 17451972 | NA | Plbd2 | phospholipase B domain containing 2 | 71772 | ENSMUSG00000029598 |
| 99 | 17490432 | NA | Med25 | mediator of RNA polymerase II transcription, subunit 25 homolog (yeast) | 75613 | ENSMUSG00000002968 |
| 100 | 17515277 | NA | Smarca4 | SWI/SNF related, matrix associated, actin dependent regulator of chromatin, subfamily a, member 4 | 20586 | ENSMUSG00000032187 |
| 101 | 17256579 | NA | Cntnap1 | contactin associated protein-like 1 | 53321 | ENSMUSG00000017167 |
| 102 | 17475218 | NA | Cic | capicua homolog (Drosophila) | 71722 | ENSMUSG00000005442 |
| 103 | 17475221 | NA | Cic | capicua homolog (Drosophila) | 71722 | ENSMUSG00000005442 |
| 104 | 17542695 | NA | G6pdx | glucose-6-phosphate dehydrogenase X-linked | 14381 | ENSMUSG00000031400 |
| 105 | 17477468 | NA | Lrrc4b | leucine rich repeat containing 4B | 272381 | ENSMUSG00000047085 |
| 106 | 17475182 | NA | Zfp526 | zinc finger protein 526 | 210172 | ENSMUSG00000046541 |
| 107 | 17504327 | NA | Ndrg4 | N-myc downstream regulated gene 4 | 234593 | ENSMUSG00000036564 |
| 108 | 17538891 | NA | Iqsec2 | IQ motif and Sec7 domain 2 | 245666 | ENSMUSG00000041115 |
| 109 | 17313504 | NA | Srebf2 | sterol regulatory element binding factor 2 | 20788 | ENSMUSG00000022463 |
| 110 | 17515843 | NA | Kirrel3 | kin of IRRE like 3 (Drosophila) | 67703 | ENSMUSG00000032036 |
| 111 | 17373521 | NA | Chst1 | carbohydrate (keratan sulfate Gal-6) sulfotransferase 1 | 76969 | ENSMUSG00000027221 |
| 112 | 17346427 | NA | Khsrp | KH-type splicing regulatory protein | 16549 | ENSMUSG00000007670 |
| 113 | 17342719 | NA | Grm4 | glutamate receptor, metabotropic 4 | 268934 | ENSMUSG00000063239 |
| 114 | 17314164 | NA | Mapk8ip2 | mitogen-activated protein kinase 8 interacting protein 2 | 60597 | ENSMUSG00000022619 |
| 115 | 17377661 | NA | Scrt2 | scratch homolog 2, zinc finger protein (Drosophila) | 545474 | ENSMUSG00000060257 |
| 116 | 17247117 | NA | Zmiz2 | zinc finger, MIZ-type containing 2 | 52915 | ENSMUSG00000041164 |
| 117 | 17503122 | NA | Cacna1a | calcium channel, voltage-dependent, P/Q type, alpha 1A subunit | 12286 | ENSMUSG00000034656 |
| 118 | 17400521 | NA | Sv2a | synaptic vesicle glycoprotein 2 a | 64051 | ENSMUSG00000038486 |
| 119 | 17494596 | NA | Tpp1 | tripeptidyl peptidase I | 12751 | ENSMUSG00000030894 |
| 120 | 17513297 | NA | Cdyl2 | chromodomain protein, Y chromosome-like 2 | 75796 | ENSMUSG00000031758 |
| 121 | 17540059 | NA | Porcn | porcupine homolog (Drosophila) | 53627 | ENSMUSG00000031169 |
| 122 | 17308413 | NA | Epb4.9 | erythrocyte protein band 4.9 | 13829 | ENSMUSG00000022099 |
| 123 | 17263011 | NA | Glra1 | glycine receptor, alpha 1 subunit | 14654 | ENSMUSG00000000263 |
| 124 | 17253376 | NA | Sez6 | seizure related gene 6 | 20370 | ENSMUSG00000000632 |
| 125 | 17268120 | NA | Pdk2 | pyruvate dehydrogenase kinase, isoenzyme 2 | 18604 | ENSMUSG00000038967 |
| 126 | 17222256 | NA | Actr1b | ARP1 actin-related protein 1B, centractin beta | 226977 | ENSMUSG00000037351 |
| 127 | 17327765 | NA | Coro7 | coronin 7 | 78885 | ENSMUSG00000039637 |
| 128 | 17268786 | NA | Neurod2 | neurogenic differentiation 2 | 18013 | ENSMUSG00000038255 |
| 129 | 17475564 | NA | Numbl | numb-like | 18223 | ENSMUSG00000063160 |
| 130 | 17258683 | NA | Mgat5b | mannoside acetylglucosaminyltransferase 5, isoenzyme B | 268510 | ENSMUSG00000043857 |
| 131 | 17484419 | NA | Kndc1 | kinase non-catalytic C-lobe domain (KIND) containing 1 | 76484 | ENSMUSG00000066129 |
| 132 | 17425058 | NA | Coro2a | coronin, actin binding protein 2A | 107684 | ENSMUSG00000028337 |
| 133 | 17517073 | NA | Drd2 | dopamine receptor D2 | 13489 | ENSMUSG00000032259 |
| 134 | 17321582 | NA | Faim2 | Fas apoptotic inhibitory molecule 2 | 72393 | ENSMUSG00000023011 |
| 135 | 17536720 | NA | Nlgn3 | neuroligin 3 | 245537 | ENSMUSG00000031302 |
| 136 | 17478181 | NA | Kcnc1 | potassium voltage gated channel, Shaw-related subfamily, member 1 | 16502 | ENSMUSG00000058975 |
| 137 | 17475616 | NA | Hipk4 | homeodomain interacting protein kinase 4 | 233020 | ENSMUSG00000040424 |
| 138 | 17435570 | NA | Htr5a | 5-hydroxytryptamine (serotonin) receptor 5A | 15563 | ENSMUSG00000039106 |
| 139 | 17465332 | NA | Lrrc4 | leucine rich repeat containing 4 | 192198 | ENSMUSG00000049939 |
| 140 | 17473161 | NA | Cacng8 | calcium channel, voltage-dependent, gamma subunit 8 | 81905 | ENSMUSG00000053395 |
| 141 | 17451356 | NA | Sgsm1 | small G protein signaling modulator 1 | 52850 | ENSMUSG00000042216 |
| 142 | 17539019 | NA | Ubqln2 | ubiquilin 2 | 54609 | ENSMUSG00000050148 |
| 143 | 17259344 | NA | Hgs | HGF-regulated tyrosine kinase substrate | 15239 | ENSMUSG00000025793 |
| 144 | 17441453 | NA | Nos1 | nitric oxide synthase 1, neuronal | 18125 | ENSMUSG00000029361 |
| 145 | 17257492 | NA | Dcaf7 | DDB1 and CUL4 associated factor 7 | 71833 | ENSMUSG00000049354 |
| 146 | 17300484 | NA | Cpne6 | copine VI | 12891 | ENSMUSG00000022212 |
| 147 | 17257197 | NA | Mapt | microtubule-associated protein tau | 17762 | ENSMUSG00000018411 |
| 148 | 17269583 | NA | Zfp385c | zinc finger protein 385C | 278304 | ENSMUSG00000014198 |
| 149 | 17436877 | NA | Ablim2 | actin-binding LIM protein 2 | 231148 | ENSMUSG00000029095 |
| 150 | 17409005 | NA | Slc6a17 | solute carrier family 6 (neurotransmitter transporter), member 17 | 229706 | ENSMUSG00000027894 |
| 151 | 17485152 | NA | Brsk2 | BR serine/threonine kinase 2 | 75770 | ENSMUSG00000053046 |
| 152 | 17235300 | NA | Apc2 | adenomatosis polyposis coli 2 | 23805 | ENSMUSG00000020135 |
| 153 | 17454574 | NA | Ttyh3 | tweety homolog 3 (Drosophila) | 78339 | ENSMUSG00000036565 |
| 154 | 17523281 | NA | Trak1 | trafficking protein, kinesin binding 1 | 67095 | ENSMUSG00000032536 |
| 155 | 17420316 | NA | Rap1gap | Rap1 GTPase-activating protein | 110351 | ENSMUSG00000041351 |
| 156 | 17531999 | NA | Rbms3 | RNA binding motif, single stranded interacting protein | 207181 | ENSMUSG00000039607 |
| 157 | 17384021 | NA | Stxbp1 | syntaxin binding protein 1 | 20910 | ENSMUSG00000026797 |
| 158 | 17520198 | NA | Rasgrf1 | RAS protein-specific guanine nucleotide-releasing factor 1 | 19417 | ENSMUSG00000032356 |
| 159 | 17491285 | NA | Ptpn5 | protein tyrosine phosphatase, non-receptor type 5 | 19259 | ENSMUSG00000030854 |
| 160 | 17530733 | NA | Grm2 | glutamate receptor, metabotropic 2 | 108068 | ENSMUSG00000023192 |
| 161 | 17535627 | NA | Abcd1 | ATP-binding cassette, sub-family D (ALD), member 1 | 11666 | ENSMUSG00000031378 |
| 162 | 17474067 | NA | Slc8a2 | solute carrier family 8 (sodium/calcium exchanger), member 2 | 110891 | ENSMUSG00000030376 |
| 163 | 17465620 | NA | Podxl | podocalyxin-like | 27205 | ENSMUSG00000025608 |
| 164 | 17357959 | NA | Gnaq | guanine nucleotide binding protein, alpha q polypeptide | 14682 | ENSMUSG00000024639 |
| 165 | 17451482 | NA | Svop | SV2 related protein | 68666 | ENSMUSG00000042078 |
| 166 | 17266452 | NA | Sarm1 | sterile alpha and HEAT/Armadillo motif containing 1 | 237868 | ENSMUSG00000050132 |
| 167 | 17531080 | NA | Rnf123 | ring finger protein 123 | 84585 | ENSMUSG00000041528 |
| 168 | 17419587 | NA | Slc9a1 | solute carrier family 9 (sodium/hydrogen exchanger), member 1 | 20544 | ENSMUSG00000028854 |
| 169 | 17235211 | NA | Midn | midnolin | 59090 | ENSMUSG00000035621 |
| 170 | 17496452 | NA | Taok2 | TAO kinase 2 | 381921 | ENSMUSG00000059981 |
| 171 | 17517634 | NA | Sin3a | transcriptional regulator, SIN3A (yeast) | 20466 | ENSMUSG00000042557 |
| 172 | 17452719 | NA | Abcb9 | ATP-binding cassette, sub-family B (MDR/TAP), member 9 | 56325 | ENSMUSG00000029408 |
| 173 | 17471155 | NA | Kcna1 | potassium voltage-gated channel, shaker-related subfamily, member 1 | 16485 | ENSMUSG00000047976 |
| 174 | 17450059 | NA | Sec31a | Sec31 homolog A (S. cerevisiae) | 69162 | ENSMUSG00000035325 |
| 175 | 17355463 | NA | Ctif | CBP80/20-dependent translation initiation factor | 269037 | ENSMUSG00000052928 |
| 176 | 17461868 | NA | Slc6a1 | solute carrier family 6 (neurotransmitter transporter, GABA), member 1 | 232333 | ENSMUSG00000030310 |
| 177 | 17251303 | NA | Pik3r5 | phosphoinositide-3-kinase, regulatory subunit 5, p101 | 320207 | ENSMUSG00000020901 |
| 178 | 17441595 | NA | Tbx3 | T-box 3 | 21386 | ENSMUSG00000018604 |
| 179 | 17407272 | NA | Ints3 | integrator complex subunit 3 | 229543 | ENSMUSG00000027933 |
| 180 | 17297462 | NA | Sec24c | Sec24 related gene family, member C (S. cerevisiae) | 218811 | ENSMUSG00000039367 |
| 181 | 17361470 | NA | Klc2 | kinesin light chain 2 | 16594 | ENSMUSG00000024862 |
| 182 | 17359583 | NA | Cnnm1 | cyclin M1 | 83674 | ENSMUSG00000025189 |
| 183 | 17336987 | NA | Bag6 | BCL2-associated athanogene 6 | 224727 | ENSMUSG00000024392 |
| 184 | 17345527 | NA | Klhdc3 | kelch domain containing 3 | 71765 | ENSMUSG00000063576 |
| 185 | 17533640 | NA | Cdk16 | cyclin-dependent kinase 16 | 18555 | ENSMUSG00000031065 |
| 186 | 17345740 | NA | Foxp4 | forkhead box P4 | 74123 | ENSMUSG00000023991 |
| 187 | 17309287 | NA | Pou4f1 | POU domain, class 4, transcription factor 1 | 18996 | ENSMUSG00000048349 |
| 188 | 17318923 | NA | Cacng2 | calcium channel, voltage-dependent, gamma subunit 2 | 12300 | ENSMUSG00000019146 |
| 189 | 17272798 | NA | Cant1 | calcium activated nucleotidase 1 | 76025 | ENSMUSG00000025575 |
| 190 | 17521371 | NA | Tmem115 | transmembrane protein 115 | 56395 | ENSMUSG00000010045 |
| 191 | 17334545 | NA | Clcn7 | chloride channel 7 | 26373 | ENSMUSG00000036636 |
| 192 | 17243717 | NA | Syn3 | synapsin III | 27204 | ENSMUSG00000059602 |
| 193 | 17436999 | NA | Crmp1 | collapsin response mediator protein 1 | 12933 | ENSMUSG00000029121 |
| 194 | 17399496 | NA | Adar | adenosine deaminase, RNA-specific | 56417 | ENSMUSG00000027951 |
| 195 | 17329516 | NA | Fgf12 | fibroblast growth factor 12 | 14167 | ENSMUSG00000022523 |
| 196 | 17342065 | NA | Mapk8ip3 | mitogen-activated protein kinase 8 interacting protein 3 | 30957 | ENSMUSG00000024163 |
| 197 | 17506808 | NA | Trim67 | tripartite motif-containing 67 | 330863 | ENSMUSG00000036913 |
| 198 | 17406892 | NA | Rusc1 | RUN and SH3 domain containing 1 | 72296 | ENSMUSG00000041263 |
| 199 | 17226891 | NA | Cntn2 | contactin 2 | 21367 | ENSMUSG00000053024 |
| 200 | 17318523 | NA | Scrt1 | scratch homolog 1, zinc finger protein (Drosophila) | 170729 | ENSMUSG00000048385 |
| 201 | 17395766 | NA | Eef1a2 | eukaryotic translation elongation factor 1 alpha 2 | 13628 | ENSMUSG00000016349 |
| 202 | 17522338 | NA | Scap | SREBF chaperone | 235623 | ENSMUSG00000032485 |
| 203 | 17505599 | NA | Vac14 | Vac14 homolog (S. cerevisiae) | 234729 | ENSMUSG00000010936 |
| 204 | 17416325 | NA | Dhcr24 | 24-dehydrocholesterol reductase | 74754 | ENSMUSG00000034926 |
| 205 | 17351262 | NA | St8sia3 | ST8 alpha-N-acetyl-neuraminide alpha-2,8-sialyltransferase 3 | 20451 | ENSMUSG00000056812 |
| 206 | 17424279 | NA | Cntfr | ciliary neurotrophic factor receptor | 12804 | ENSMUSG00000028444 |
| 207 | 17505689 | NA | Aars | alanyl-tRNA synthetase | 234734 | ENSMUSG00000031960 |
| 208 | 17361223 | NA | Adrbk1 | adrenergic receptor kinase, beta 1 | 110355 | ENSMUSG00000024858 |
| 209 | 17401394 | NA | Kcnd3 | potassium voltage-gated channel, Shal-related family, member 3 | 56543 | ENSMUSG00000040896 |
| 210 | 17292753 | NA | Gprin1 | G protein-regulated inducer of neurite outgrowth 1 | 26913 | ENSMUSG00000069227 |
| 211 | 17328958 | NA | Gp1bb | glycoprotein Ib, beta polypeptide | 14724 | ENSMUSG00000050761 |
| 212 | 17450536 | NA | Barhl2 | BarH-like 2 (Drosophila) | 104382 | ENSMUSG00000034384 |
| 213 | 17453222 | NA | Wbscr17 | Williams-Beuren syndrome chromosome region 17 homolog (human) | 212996 | ENSMUSG00000034040 |
| 214 | 17307905 | NA | Dpysl2 | dihydropyrimidinase-like 2 | 12934 | ENSMUSG00000022048 |
| 215 | 17253276 | NA | Git1 | G protein-coupled receptor kinase-interactor 1 | 216963 | ENSMUSG00000011877 |
| 216 | 17440538 | NA | Galnt9 | UDP-N-acetyl-alpha-D-galactosamine:polypeptide N-acetylgalactosaminyltransferase 9 | 231605 | ENSMUSG00000033316 |
| 217 | 17273348 | NA | Fasn | fatty acid synthase | 14104 | ENSMUSG00000025153 |
| 218 | 17521327 | NA | Cacna2d2 | calcium channel, voltage-dependent, alpha 2/delta subunit 2 | 56808 | ENSMUSG00000010066 |
| 219 | 17292011 | NA | Tfap2a | transcription factor AP-2, alpha | 21418 | ENSMUSG00000021359 |
| 220 | 17318895 | NA | Foxred2 | FAD-dependent oxidoreductase domain containing 2 | 239554 | ENSMUSG00000016552 |
| 221 | 17477714 | NA | Slc17a7 | solute carrier family 17 (sodium-dependent inorganic phosphate cotransporter), member 7 | 72961 | ENSMUSG00000070570 |
| 222 | 17361494 | NA | Pacs1 | phosphofurin acidic cluster sorting protein 1 | 107975 | ENSMUSG00000024855 |
| 223 | 17293045 | NA | Spock1 | sparc/osteonectin, cwcv and kazal-like domains proteoglycan 1 | 20745 | ENSMUSG00000056222 |
| 224 | 17532879 | NA | Syp | synaptophysin | 20977 | ENSMUSG00000031144 |
| 225 | 17483194 | NA | Sez6l2 | seizure related 6 homolog like 2 | 233878 | ENSMUSG00000030683 |
| 226 | 17397377 | NA | Pcdh10 | protocadherin 10 | 18526 | ENSMUSG00000049100 |
| 227 | 17302289 | NA | Pcdh17 | protocadherin 17 | 219228 | ENSMUSG00000035566 |
| 228 | 17490452 | NA | Ap2a1 | adaptor protein complex AP-2, alpha 1 subunit | 11771 | ENSMUSG00000060279 |
| 229 | 17288442 | NA | Irx2 | Iroquois related homeobox 2 (Drosophila) | 16372 | ENSMUSG00000001504 |
| 230 | 17376993 | NA | Pcsk2 | proprotein convertase subtilisin/kexin type 2 | 18549 | ENSMUSG00000027419 |
| 231 | 17237915 | NA | Agap2 | ArfGAP with GTPase domain, ankyrin repeat and PH domain 2 | 216439 | ENSMUSG00000025422 |
| 232 | 17488312 | NA | Map3k10 | mitogen-activated protein kinase kinase kinase 10 | 269881 | ENSMUSG00000040390 |
| 233 | 17268884 | NA | Nr1d1 | nuclear receptor subfamily 1, group D, member 1 | 217166 | ENSMUSG00000020889 |
| 234 | 17417702 | NA | Slc6a9 | solute carrier family 6 (neurotransmitter transporter, glycine), member 9 | 14664 | ENSMUSG00000028542 |
| 235 | 17510013 | NA | Upf1 | UPF1 regulator of nonsense transcripts homolog (yeast) | 19704 | ENSMUSG00000058301 |
| 236 | 17502603 | NA | Rasd2 | RASD family, member 2 | 75141 | ENSMUSG00000034472 |
| 237 | 17235037 | NA | Arid3a | AT rich interactive domain 3A (BRIGHT-like) | 13496 | ENSMUSG00000019564 |
| 238 | 17541383 | NA | Zdhhc9 | zinc finger, DHHC domain containing 9 | 208884 | ENSMUSG00000036985 |
| 239 | 17504130 | NA | Cx3cl1 | chemokine (C-X3-C motif) ligand 1 | 20312 | ENSMUSG00000031778 |
| 240 | 17382496 | NA | Grin1 | glutamate receptor, ionotropic, NMDA1 (zeta 1) | 14810 | ENSMUSG00000026959 |
| 241 | 17367921 | NA | Grin1 | glutamate receptor, ionotropic, NMDA1 (zeta 1) | 14810 | ENSMUSG00000026959 |
| 242 | 17475851 | NA | Lrfn1 | leucine rich repeat and fibronectin type III domain containing 1 | 80749 | NULL |
| 243 | 17266107 | NA | Abr | active BCR-related gene | 109934 | ENSMUSG00000017631 |
| 244 | 17279640 | NA | Ptprn2 | protein tyrosine phosphatase, receptor type, N polypeptide 2 | 19276 | ENSMUSG00000056553 |
| 245 | 17245539 | NA | Srgap1 | SLIT-ROBO Rho GTPase activating protein 1 | 117600 | ENSMUSG00000020121 |
| 246 | 17376167 | NA | Sirpa | signal-regulatory protein alpha | 19261 | ENSMUSG00000037902 |
| 247 | 17268849 | NA | Med24 | mediator complex subunit 24 | 23989 | ENSMUSG00000017210 |
| 248 | 17319207 | NA | Csnk1e | casein kinase 1, epsilon | 27373 | ENSMUSG00000022433 |
| 249 | 17518007 | NA | Pkm | pyruvate kinase, muscle | 18746 | ENSMUSG00000032294 |
| 250 | 17406760 | NA | Sema4a | sema domain, immunoglobulin domain (Ig), transmembrane domain (TM) and short cytoplasmic domain, (semaphorin) 4A | 20351 | ENSMUSG00000028064 |
| 251 | 17342509 | NA | Pigq | phosphatidylinositol glycan anchor biosynthesis, class Q | 14755 | ENSMUSG00000025728 |
| 252 | 17243330 | NA | Nfic | nuclear factor I/C | 18029 | ENSMUSG00000055053 |
| 253 | 17222156 | NA | Kansl3 | KAT8 regulatory NSL complex subunit 3 | 226976 | ENSMUSG00000010453 |
| 254 | 17385374 | NA | Nr4a2 | nuclear receptor subfamily 4, group A, member 2 | 18227 | ENSMUSG00000026826 |
| 255 | 17451223 | NA | Sez6l | seizure related 6 homolog like | 56747 | ENSMUSG00000058153 |
| 256 | 17436944 | NA | Ppp2r2c | protein phosphatase 2 (formerly 2A), regulatory subunit B (PR 52), gamma isoform | 269643 | ENSMUSG00000029120 |
| 257 | 17532953 | NA | Tfe3 | transcription factor E3 | 209446 | ENSMUSG00000000134 |
| 258 | 17262065 | NA | Cyfip2 | cytoplasmic FMR1 interacting protein 2 | 76884 | ENSMUSG00000020340 |
| 259 | 17425095 | NA | Gabbr2 | gamma-aminobutyric acid (GABA) B receptor, 2 | 242425 | ENSMUSG00000039809 |
| 260 | 17339554 | NA | Lbh | limb-bud and heart | 77889 | ENSMUSG00000024063 |
| 261 | 17354653 | NA | Tcof1 | Treacher Collins Franceschetti syndrome 1, homolog | 21453 | ENSMUSG00000024613 |
| 262 | 17408211 | NA | Pde4dip | phosphodiesterase 4D interacting protein (myomegalin) | 83679 | ENSMUSG00000038170 |
| 263 | 17335145 | NA | Pacsin1 | protein kinase C and casein kinase substrate in neurons 1 | 23969 | ENSMUSG00000040276 |
| 264 | 17243229 | NA | Atcay | ataxia, cerebellar, Cayman type homolog (human) | 16467 | ENSMUSG00000034958 |
| 265 | 17498607 | NA | Mcoln1 | mucolipin 1 | 94178 | ENSMUSG00000004567 |
| 266 | 17255788 | NA | Arhgap23 | Rho GTPase activating protein 23 | 58996 | ENSMUSG00000049807 |
| 267 | 17249811 | NA | Slc36a1 | solute carrier family 36 (proton/amino acid symporter), member 1 | 215335 | ENSMUSG00000020261 |
| 268 | 17503884 | NA | Gnao1 | guanine nucleotide binding protein, alpha O | 14681 | ENSMUSG00000031748 |
| 269 | 17248691 | NA | Ebf1 | early B cell factor 1 | 13591 | ENSMUSG00000057098 |
| 270 | 17383858 | NA | Dnm1 | dynamin 1 | 13429 | ENSMUSG00000026825 |
| 271 | 17364665 | NA | Slit1 | slit homolog 1 (Drosophila) | 20562 | ENSMUSG00000025020 |
| 272 | 17224500 | NA | Atg9a | autophagy related 9A | 245860 | ENSMUSG00000033124 |
| 273 | 17510696 | NA | Pou4f2 | POU domain, class 4, transcription factor 2 | 18997 | ENSMUSG00000031688 |
| 274 | 17504399 | NA | Cdh5 | cadherin 5 | 12562 | ENSMUSG00000031871 |
| 275 | 17498041 | NA | Dusp8 | dual specificity phosphatase 8 | 18218 | ENSMUSG00000037887 |
| 276 | 17497904 | NA | Slc25a22 | solute carrier family 25 (mitochondrial carrier, glutamate), member 22 | 68267 | ENSMUSG00000019082 |
| 277 | 17368685 | NA | Ralgds | ral guanine nucleotide dissociation stimulator | 19730 | ENSMUSG00000026821 |
| 278 | 17401650 | NA | Amigo1 | adhesion molecule with Ig like domain 1 | 229715 | ENSMUSG00000050947 |
| 279 | 17424128 | NA | Nol6 | nucleolar protein family 6 (RNA-associated) | 230082 | ENSMUSG00000028430 |
| 280 | 17529930 | NA | Clstn2 | calsyntenin 2 | 64085 | ENSMUSG00000032452 |
| 281 | 17516837 | NA | Bace1 | beta-site APP cleaving enzyme 1 | 23821 | ENSMUSG00000032086 |
| 282 | 17548746 | NA | Bace1 | beta-site APP cleaving enzyme 1 | 23821 | ENSMUSG00000032086 |
| 283 | 17234936 | NA | Hcn2 | hyperpolarization-activated, cyclic nucleotide-gated K+ 2 | 15166 | ENSMUSG00000020331 |
| 284 | 17272817 | NA | Rbfox3 | RNA binding protein, fox-1 homolog (C. elegans) 3 | 52897 | ENSMUSG00000025576 |
| 285 | 17267039 | NA | Lhx1 | LIM homeobox protein 1 | 16869 | ENSMUSG00000018698 |
| 286 | 17259810 | NA | Inpp5j | inositol polyphosphate 5-phosphatase J | 170835 | ENSMUSG00000034570 |
| 287 | 17235556 | NA | Zbtb7a | zinc finger and BTB domain containing 7a | 16969 | ENSMUSG00000035011 |
| 288 | 17242281 | NA | Adarb1 | adenosine deaminase, RNA-specific, B1 | 110532 | ENSMUSG00000020262 |
| 289 | 17454995 | NA | Tmem130 | transmembrane protein 130 | 243339 | ENSMUSG00000043388 |
| 290 | 17419097 | NA | Bai2 | brain-specific angiogenesis inhibitor 2 | 230775 | ENSMUSG00000028782 |
| 291 | 17516462 | NA | Thy1 | thymus cell antigen 1, theta | 21838 | ENSMUSG00000032011 |
| 292 | 17314051 | NA | Panx2 | pannexin 2 | 406218 | ENSMUSG00000058441 |
| 293 | 17461923 | NA | Syn2 | synapsin II | 20965 | ENSMUSG00000009394 |
| 294 | 17436545 | NA | Nat8l | N-acetyltransferase 8-like | 269642 | ENSMUSG00000048142 |
| 295 | 17388406 | NA | Slc35c1 | solute carrier family 35, member C1 | 228368 | ENSMUSG00000049922 |
| 296 | 17529218 | NA | Htr1b | 5-hydroxytryptamine (serotonin) receptor 1B | 15551 | ENSMUSG00000049511 |
| 297 | 17284065 | NA | Cdc42bpb | CDC42 binding protein kinase beta | 217866 | ENSMUSG00000021279 |
| 298 | 17238605 | NA | Itga7 | integrin alpha 7 | 16404 | ENSMUSG00000025348 |
| 299 | 17354629 | NA | Ndst1 | N-deacetylase/N-sulfotransferase (heparan glucosaminyl) 1 | 15531 | ENSMUSG00000054008 |
| 300 | 17328870 | NA | Zdhhc8 | zinc finger, DHHC domain containing 8 | 27801 | ENSMUSG00000060166 |
| 301 | 17270028 | NA | Mpp2 | membrane protein, palmitoylated 2 (MAGUK p55 subfamily member 2) | 50997 | ENSMUSG00000017314 |
| 302 | 17308939 | NA | Pcdh8 | protocadherin 8 | 18530 | ENSMUSG00000036422 |
| 303 | 17388261 | NA | Atg13 | autophagy related 13 | 51897 | ENSMUSG00000027244 |
| 304 | 17341608 | NA | Flywch1 | FLYWCH-type zinc finger 1 | 224613 | ENSMUSG00000040097 |
| 305 | 17509907 | NA | Gatad2a | GATA zinc finger domain containing 2A | 234366 | ENSMUSG00000036180 |
| 306 | 17303897 | NA | Ndst2 | N-deacetylase/N-sulfotransferase (heparan glucosaminyl) 2 | 17423 | ENSMUSG00000039308 |
| 307 | 17400617 | NA | Ankrd34a | ankyrin repeat domain 34A | 545554 | ENSMUSG00000049097 |
| 308 | 17461897 | NA | Atg7 | autophagy related 7 | 74244 | ENSMUSG00000030314 |
| 309 | 17488292 | NA | Pld3 | phospholipase D family, member 3 | 18807 | ENSMUSG00000003363 |
| 310 | 17542419 | NA | L1cam | L1 cell adhesion molecule | 16728 | ENSMUSG00000031391 |
| 311 | 17271158 | NA | Cacng5 | calcium channel, voltage-dependent, gamma subunit 5 | 140723 | ENSMUSG00000040373 |
| 312 | 17332495 | NA | Dscam | Down syndrome cell adhesion molecule | 13508 | ENSMUSG00000050272 |
| 313 | 17344251 | NA | Prrc2a | proline-rich coiled-coil 2A | 53761 | ENSMUSG00000024393 |
| 314 | 17378450 | NA | Mmp24 | matrix metallopeptidase 24 | 17391 | ENSMUSG00000027612 |
| 315 | 17338136 | NA | Cul7 | cullin 7 | 66515 | ENSMUSG00000038545 |
| 316 | 17314636 | NA | Cacnb3 | calcium channel, voltage-dependent, beta 3 subunit | 12297 | ENSMUSG00000003352 |
| 317 | 17273714 | NA | Adcy3 | adenylate cyclase 3 | 104111 | ENSMUSG00000020654 |
| 318 | 17477946 | NA | Mamstr | MEF2 activating motif and SAP domain containing transcriptional regulator | 74490 | ENSMUSG00000042918 |
| 319 | 17517752 | NA | Ulk3 | unc-51-like kinase 3 | 71742 | ENSMUSG00000032308 |
| 320 | 17515106 | NA | Pde4a | phosphodiesterase 4A, cAMP specific | 18577 | ENSMUSG00000032177 |
| 321 | 17243157 | NA | Diras1 | DIRAS family, GTP-binding RAS-like 1 | 208666 | ENSMUSG00000043670 |
| 322 | 17506443 | NA | Acsf3 | acyl-CoA synthetase family member 3 | 257633 | ENSMUSG00000015016 |
| 323 | 17290835 | NA | Pou6f2 | POU domain, class 6, transcription factor 2 | 218030 | ENSMUSG00000009734 |
| 324 | 17308299 | NA | Slc39a14 | solute carrier family 39 (zinc transporter), member 14 | 213053 | ENSMUSG00000022094 |
| 325 | 17369147 | NA | Nup188 | nucleoporin 188 | 227699 | ENSMUSG00000052533 |
| 326 | 17237937 | NA | B4galnt1 | beta-1,4-N-acetyl-galactosaminyl transferase 1 | 14421 | ENSMUSG00000006731 |
| 327 | 17283935 | NA | Wars | tryptophanyl-tRNA synthetase | 22375 | ENSMUSG00000021266 |
| 328 | 17474157 | NA | Strn4 | striatin, calmodulin binding protein 4 | 97387 | ENSMUSG00000030374 |
| 329 | 17292346 | NA | Phf2 | PHD finger protein 2 | 18676 | ENSMUSG00000038025 |
| 330 | 17383320 | NA | Brd3 | bromodomain containing 3 | 67382 | ENSMUSG00000026918 |
| 331 | 17377144 | NA | Slc24a3 | solute carrier family 24 (sodium/potassium/calcium exchanger), member 3 | 94249 | ENSMUSG00000063873 |
| 332 | 17513806 | NA | Cbfa2t3 | core-binding factor, runt domain, alpha subunit 2, translocated to, 3 (human) | 12398 | ENSMUSG00000006362 |
| 333 | 17346387 | NA | Mllt1 | myeloid/lymphoid or mixed-lineage leukemia (trithorax homolog, Drosophila); translocated to, 1 | 64144 | ENSMUSG00000024212 |
| 334 | 17243057 | NA | Ap3d1 | adaptor-related protein complex 3, delta 1 subunit | 11776 | ENSMUSG00000020198 |
| 335 | 17271622 | NA | Cdc42ep4 | CDC42 effector protein (Rho GTPase binding) 4 | 56699 | ENSMUSG00000041598 |
| 336 | 17272926 | NA | Nptx1 | neuronal pentraxin 1 | 18164 | ENSMUSG00000025582 |
| 337 | 17398785 | NA | Arhgef11 | Rho guanine nucleotide exchange factor (GEF) 11 | 213498 | ENSMUSG00000041977 |
| 338 | 17361779 | NA | Scyl1 | SCY1-like 1 (S. cerevisiae) | 78891 | ENSMUSG00000024941 |
| 339 | 17370883 | NA | Kcnj3 | potassium inwardly-rectifying channel, subfamily J, member 3 | 16519 | ENSMUSG00000026824 |
| 340 | 17234116 | NA | Ccdc6 | coiled-coil domain containing 6 | 76551 | ENSMUSG00000048701 |
| 341 | 17346155 | NA | Sema6b | sema domain, transmembrane domain (TM), and cytoplasmic domain, (semaphorin) 6B | 20359 | ENSMUSG00000001227 |
| 342 | 17516564 | NA | Hyou1 | hypoxia up-regulated 1 | 12282 | ENSMUSG00000032115 |
| 343 | 17346185 | NA | Dpp9 | dipeptidylpeptidase 9 | 224897 | ENSMUSG00000001229 |
| 344 | 17235663 | NA | Pip5k1c | phosphatidylinositol-4-phosphate 5-kinase, type 1 gamma | 18717 | ENSMUSG00000034902 |
| 345 | 17378721 | NA | Src | Rous sarcoma oncogene | 20779 | ENSMUSG00000027646 |
| 346 | 17313023 | NA | Gtpbp1 | GTP binding protein 1 | 14904 | ENSMUSG00000042535 |
| 347 | 17343405 | NA | Wiz | widely-interspaced zinc finger motifs | 22404 | ENSMUSG00000024050 |
| 348 | 17399044 | NA | Smg5 | Smg-5 homolog, nonsense mediated mRNA decay factor (C. elegans) | 229512 | ENSMUSG00000001415 |
| 349 | 17336660 | NA | Atf6b | activating transcription factor 6 beta | 12915 | ENSMUSG00000015461 |
| 350 | 17500301 | NA | Gpr124 | G protein-coupled receptor 124 | 78560 | ENSMUSG00000031486 |
| 351 | 17242729 | NA | Med16 | mediator complex subunit 16 | 216154 | ENSMUSG00000013833 |
| 352 | 17486930 | NA | Grlf1 | glucocorticoid receptor DNA binding factor 1 | 232906 | ENSMUSG00000058230 |
| 353 | 17334846 | NA | Tmem8 | transmembrane protein 8 (five membrane-spanning domains) | 60455 | ENSMUSG00000024180 |
| 354 | 17325206 | NA | Adcy5 | adenylate cyclase 5 | 224129 | ENSMUSG00000022840 |
| 355 | 17490312 | NA | Nr1h2 | nuclear receptor subfamily 1, group H, member 2 | 22260 | ENSMUSG00000060601 |
| 356 | 17264960 | NA | Fgf11 | fibroblast growth factor 11 | 14166 | ENSMUSG00000042826 |
| 357 | 17219519 | NA | Igsf8 | immunoglobulin superfamily, member 8 | 140559 | ENSMUSG00000038034 |
| 358 | 17359960 | NA | Gbf1 | golgi-specific brefeldin A-resistance factor 1 | 107338 | ENSMUSG00000025224 |
| 359 | 17516731 | NA | Scn2b | sodium channel, voltage-gated, type II, beta | 72821 | ENSMUSG00000070304 |
| 360 | 17338210 | NA | Trerf1 | transcriptional regulating factor 1 | 224829 | ENSMUSG00000064043 |
| 361 | 17227589 | NA | Lhx9 | LIM homeobox protein 9 | 16876 | ENSMUSG00000019230 |
| 362 | 17276622 | NA | Fntb | farnesyltransferase, CAAX box, beta | 110606 | ENSMUSG00000033373 |
| 363 | 17360440 | NA | Adra2a | adrenergic receptor, alpha 2a | 11551 | ENSMUSG00000033717 |
| 364 | 17260261 | NA | Camk2b | calcium/calmodulin-dependent protein kinase II, beta | 12323 | ENSMUSG00000057897 |
| 365 | 17322359 | NA | Zfp385a | zinc finger protein 385A | 29813 | ENSMUSG00000000552 |
| 366 | 17389647 | NA | Rasgrp1 | RAS guanyl releasing protein 1 | 19419 | ENSMUSG00000027347 |

  
  

| **Database:cellular component      &nbspName:cell part      &nbspID:GO:0044464** | | | | | | |
| --- | --- | --- | --- | --- | --- | --- |
| C=12713; O=363; E=287.76; R=1.26; rawP=3.23e-14; adjP=1.04e-12 | | | | | | |
| Index | UserID | Value | Gene Symbol | Gene Name | EntrezGene | Ensembl |
| 1 | 17378922 | NA | Slc32a1 | solute carrier family 32 (GABA vesicular transporter), member 1 | 22348 | ENSMUSG00000037771 |
| 2 | 17362646 | NA | Dagla | diacylglycerol lipase, alpha | 269060 | ENSMUSG00000035735 |
| 3 | 17439511 | NA | Prdm8 | PR domain containing 8 | 77630 | ENSMUSG00000035456 |
| 4 | 17461852 | NA | Slc6a11 | solute carrier family 6 (neurotransmitter transporter, GABA), member 11 | 243616 | ENSMUSG00000030307 |
| 5 | 17354831 | NA | Ablim3 | actin binding LIM protein family, member 3 | 319713 | ENSMUSG00000032735 |
| 6 | 17376685 | NA | Plcb1 | phospholipase C, beta 1 | 18795 | ENSMUSG00000051177 |
| 7 | 17418893 | NA | Trim62 | tripartite motif-containing 62 | 67525 | ENSMUSG00000041000 |
| 8 | 17372462 | NA | Zfp804a | zinc finger protein 804A | 241514 | ENSMUSG00000070866 |
| 9 | 17334275 | NA | Caskin1 | CASK interacting protein 1 | 268932 | ENSMUSG00000033597 |
| 10 | 17282420 | NA | Zfyve1 | zinc finger, FYVE domain containing 1 | 217695 | ENSMUSG00000042628 |
| 11 | 17252995 | NA | Slc43a2 | solute carrier family 43, member 2 | 215113 | ENSMUSG00000038178 |
| 12 | 17322600 | NA | Mgrn1 | mahogunin, ring finger 1 | 17237 | ENSMUSG00000022517 |
| 13 | 17462705 | NA | Foxj2 | forkhead box J2 | 60611 | ENSMUSG00000003154 |
| 14 | 17223283 | NA | Satb2 | special AT-rich sequence binding protein 2 | 212712 | ENSMUSG00000038331 |
| 15 | 17339313 | NA | Epb4.1l3 | erythrocyte protein band 4.1-like 3 | 13823 | ENSMUSG00000024044 |
| 16 | 17368079 | NA | Fbxw5 | F-box and WD-40 domain protein 5 | 30839 | ENSMUSG00000015095 |
| 17 | 17370234 | NA | Dab2ip | disabled 2 interacting protein | 69601 | ENSMUSG00000026883 |
| 18 | 17320813 | NA | Nell2 | NEL-like 2 (chicken) | 54003 | ENSMUSG00000022454 |
| 19 | 17469814 | NA | Atp2b2 | ATPase, Ca++ transporting, plasma membrane 2 | 11941 | ENSMUSG00000030302 |
| 20 | 17487249 | NA | Mark4 | MAP/microtubule affinity-regulating kinase 4 | 232944 | ENSMUSG00000030397 |
| 21 | 17487805 | NA | Atp1a3 | ATPase, Na+/K+ transporting, alpha 3 polypeptide | 232975 | ENSMUSG00000040907 |
| 22 | 17329074 | NA | Map6d1 | MAP6 domain containing 1 | 208158 | ENSMUSG00000041205 |
| 23 | 17314872 | NA | Smarcd1 | SWI/SNF related, matrix associated, actin dependent regulator of chromatin, subfamily d, member 1 | 83797 | ENSMUSG00000023018 |
| 24 | 17317327 | NA | Mtss1 | metastasis suppressor 1 | 211401 | ENSMUSG00000022353 |
| 25 | 17502039 | NA | Rab3a | RAB3A, member RAS oncogene family | 19339 | ENSMUSG00000031840 |
| 26 | 17290259 | NA | Ucn3 | urocortin 3 | 83428 | ENSMUSG00000044988 |
| 27 | 17453430 | NA | Limk1 | LIM-domain containing, protein kinase | 16885 | ENSMUSG00000029674 |
| 28 | 17239234 | NA | Grm1 | glutamate receptor, metabotropic 1 | 14816 | ENSMUSG00000019828 |
| 29 | 17409343 | NA | 5330417C22Rik | RIKEN cDNA 5330417C22 gene | 229722 | ENSMUSG00000040412 |
| 30 | 17224146 | NA | March4 | membrane-associated ring finger (C3HC4) 4 | 381270 | ENSMUSG00000039372 |
| 31 | 17430140 | NA | Ncdn | neurochondrin | 26562 | ENSMUSG00000028833 |
| 32 | 17452038 | NA | Dtx1 | deltex 1 homolog (Drosophila) | 14357 | ENSMUSG00000029603 |
| 33 | 17540501 | NA | Syn1 | synapsin I | 20964 | ENSMUSG00000037217 |
| 34 | 17349607 | NA | Psd2 | pleckstrin and Sec7 domain containing 2 | 74002 | ENSMUSG00000024347 |
| 35 | 17298874 | NA | Grid1 | glutamate receptor, ionotropic, delta 1 | 14803 | ENSMUSG00000041078 |
| 36 | 17413436 | NA | Tmem8b | transmembrane protein 8B | 242409 | ENSMUSG00000078716 |
| 37 | 17431720 | NA | Alpl | alkaline phosphatase, liver/bone/kidney | 11647 | ENSMUSG00000028766 |
| 38 | 17473155 | NA | Cacng7 | calcium channel, voltage-dependent, gamma subunit 7 | 81904 | ENSMUSG00000069806 |
| 39 | 17319339 | NA | Npcd | neuronal pentraxin chromo domain | 504193 | ENSMUSG00000089837 ENSMUSG00000022421 |
| 40 | 17397645 | NA | Smad9 | SMAD family member 9 | 55994 | ENSMUSG00000027796 |
| 41 | 17444202 | NA | Foxk1 | forkhead box K1 | 17425 | ENSMUSG00000056493 |
| 42 | 17269439 | NA | Hap1 | huntingtin-associated protein 1 | 15114 | ENSMUSG00000006930 |
| 43 | 17483447 | NA | Setd1a | SET domain containing 1A | 233904 | ENSMUSG00000042308 |
| 44 | 17527520 | NA | Scamp5 | secretory carrier membrane protein 5 | 56807 | ENSMUSG00000040722 |
| 45 | 17317801 | NA | Kcnk9 | potassium channel, subfamily K, member 9 | 223604 | ENSMUSG00000036760 |
| 46 | 17215820 | NA | Gpc1 | glypican 1 | 14733 | ENSMUSG00000034220 |
| 47 | 17518342 | NA | Megf11 | multiple EGF-like-domains 11 | 214058 | ENSMUSG00000036466 |
| 48 | 17344034 | NA | Skiv2l | superkiller viralicidic activity 2-like (S. cerevisiae) | 108077 | ENSMUSG00000040356 |
| 49 | 17535808 | NA | Gdi1 | guanosine diphosphate (GDP) dissociation inhibitor 1 | 14567 | ENSMUSG00000015291 |
| 50 | 17484409 | NA | Gpr123 | G protein-coupled receptor 123 | 52389 | ENSMUSG00000025475 |
| 51 | 17373530 | NA | Syt13 | synaptotagmin XIII | 80976 | ENSMUSG00000027220 |
| 52 | 17279434 | NA | Pacs2 | phosphofurin acidic cluster sorting protein 2 | 217893 | ENSMUSG00000021143 |
| 53 | 17451140 | NA | Ulk1 | unc-51 like kinase 1 | 22241 | ENSMUSG00000029512 |
| 54 | 17477454 | NA | Syt3 | synaptotagmin III | 20981 | ENSMUSG00000030731 |
| 55 | 17545450 | NA | Gpr173 | G-protein coupled receptor 173 | 70771 | ENSMUSG00000056679 |
| 56 | 17368550 | NA | Rxra | retinoid X receptor alpha | 20181 | ENSMUSG00000015846 |
| 57 | 17452139 | NA | Rph3a | rabphilin 3A | 19894 | ENSMUSG00000029608 |
| 58 | 17342999 | NA | Cpne5 | copine V | 240058 | ENSMUSG00000024008 |
| 59 | 17337513 | NA | Gabbr1 | gamma-aminobutyric acid (GABA) B receptor, 1 | 54393 | ENSMUSG00000024462 |
| 60 | 17498906 | NA | Myo16 | myosin XVI | 244281 | ENSMUSG00000039057 |
| 61 | 17510145 | NA | Pik3r2 | phosphatidylinositol 3-kinase, regulatory subunit, polypeptide 2 (p85 beta) | 18709 | ENSMUSG00000031834 |
| 62 | 17264124 | NA | Arhgap44 | Rho GTPase activating protein 44 | 216831 | ENSMUSG00000033389 |
| 63 | 17310772 | NA | Ctnnd2 | catenin (cadherin associated protein), delta 2 | 18163 | ENSMUSG00000022240 |
| 64 | 17489052 | NA | Aplp1 | amyloid beta (A4) precursor-like protein 1 | 11803 | ENSMUSG00000006651 |
| 65 | 17432808 | NA | Mfn2 | mitofusin 2 | 170731 | ENSMUSG00000029020 |
| 66 | 17468364 | NA | Rab11fip5 | RAB11 family interacting protein 5 (class I) | 52055 | ENSMUSG00000051343 |
| 67 | 17474400 | NA | Sympk | symplekin | 68188 | ENSMUSG00000023118 |
| 68 | 17214665 | NA | Sgpp2 | sphingosine-1-phosphate phosphotase 2 | 433323 | ENSMUSG00000032908 |
| 69 | 17335204 | NA | Anks1 | ankyrin repeat and SAM domain containing 1 | 224650 | ENSMUSG00000024219 |
| 70 | 17334205 | NA | Abca3 | ATP-binding cassette, sub-family A (ABC1), member 3 | 27410 | ENSMUSG00000024130 |
| 71 | 17322700 | NA | Rbfox1 | RNA binding protein, fox-1 homolog (C. elegans) 1 | 268859 | ENSMUSG00000008658 |
| 72 | 17446123 | NA | Kcnh2 | potassium voltage-gated channel, subfamily H (eag-related), member 2 | 16511 | ENSMUSG00000038319 |
| 73 | 17269464 | NA | Jup | junction plakoglobin | 16480 | ENSMUSG00000001552 |
| 74 | 17301823 | NA | Gfra2 | glial cell line derived neurotrophic factor family receptor alpha 2 | 14586 | ENSMUSG00000022103 |
| 75 | 17418732 | NA | Dlgap3 | discs, large (Drosophila) homolog-associated protein 3 | 242667 | ENSMUSG00000042388 |
| 76 | 17211347 | NA | Tfap2b | transcription factor AP-2 beta | 21419 | ENSMUSG00000025927 |
| 77 | 17259177 | NA | Rptor | regulatory associated protein of MTOR, complex 1 | 74370 | ENSMUSG00000025583 |
| 78 | 17435834 | NA | Dpysl5 | dihydropyrimidinase-like 5 | 65254 | ENSMUSG00000029168 |
| 79 | 17435528 | NA | Dpp6 | dipeptidylpeptidase 6 | 13483 | ENSMUSG00000061576 |
| 80 | 17354299 | NA | Sema6a | sema domain, transmembrane domain (TM), and cytoplasmic domain, (semaphorin) 6A | 20358 | ENSMUSG00000019647 |
| 81 | 17222625 | NA | Tgfbrap1 | transforming growth factor, beta receptor associated protein 1 | 73122 | ENSMUSG00000070939 |
| 82 | 17378242 | NA | Zfp341 | zinc finger protein 341 | 228807 | ENSMUSG00000059842 |
| 83 | 17535607 | NA | Slc6a8 | solute carrier family 6 (neurotransmitter transporter, creatine), member 8 | 102857 | ENSMUSG00000019558 |
| 84 | 17453383 | NA | Clip2 | CAP-GLY domain containing linker protein 2 | 269713 | ENSMUSG00000063146 |
| 85 | 17306666 | NA | Jph4 | junctophilin 4 | 319984 | ENSMUSG00000022208 |
| 86 | 17222465 | NA | Aff3 | AF4/FMR2 family, member 3 | 16764 | ENSMUSG00000037138 |
| 87 | 17407124 | NA | Chrnb2 | cholinergic receptor, nicotinic, beta polypeptide 2 (neuronal) | 11444 | ENSMUSG00000027950 |
| 88 | 17474389 | NA | Mypop | Myb-related transcription factor, partner of profilin | 232934 | ENSMUSG00000048481 |
| 89 | 17369305 | NA | Usp20 | ubiquitin specific peptidase 20 | 74270 | ENSMUSG00000026854 |
| 90 | 17226757 | NA | Rassf5 | Ras association (RalGDS/AF-6) domain family member 5 | 54354 | ENSMUSG00000026430 |
| 91 | 17500005 | NA | Ank1 | ankyrin 1, erythroid | 11733 | ENSMUSG00000031543 |
| 92 | 17361454 | NA | Cnih2 | cornichon homolog 2 (Drosophila) | 12794 | ENSMUSG00000024873 |
| 93 | 17440923 | NA | Ube3b | ubiquitin protein ligase E3B | 117146 | ENSMUSG00000029577 |
| 94 | 17357486 | NA | Syt7 | synaptotagmin VII | 54525 | ENSMUSG00000024743 |
| 95 | 17295987 | NA | Rgs7bp | regulator of G-protein signalling 7 binding protein | 52882 | ENSMUSG00000021719 |
| 96 | 17422138 | NA | Gpr153 | G protein-coupled receptor 153 | 100129 | ENSMUSG00000042804 |
| 97 | 17503333 | NA | Tnpo2 | transportin 2 (importin 3, karyopherin beta 2b) | 212999 | ENSMUSG00000031691 |
| 98 | 17451972 | NA | Plbd2 | phospholipase B domain containing 2 | 71772 | ENSMUSG00000029598 |
| 99 | 17490432 | NA | Med25 | mediator of RNA polymerase II transcription, subunit 25 homolog (yeast) | 75613 | ENSMUSG00000002968 |
| 100 | 17515277 | NA | Smarca4 | SWI/SNF related, matrix associated, actin dependent regulator of chromatin, subfamily a, member 4 | 20586 | ENSMUSG00000032187 |
| 101 | 17256579 | NA | Cntnap1 | contactin associated protein-like 1 | 53321 | ENSMUSG00000017167 |
| 102 | 17475218 | NA | Cic | capicua homolog (Drosophila) | 71722 | ENSMUSG00000005442 |
| 103 | 17475221 | NA | Cic | capicua homolog (Drosophila) | 71722 | ENSMUSG00000005442 |
| 104 | 17542695 | NA | G6pdx | glucose-6-phosphate dehydrogenase X-linked | 14381 | ENSMUSG00000031400 |
| 105 | 17477468 | NA | Lrrc4b | leucine rich repeat containing 4B | 272381 | ENSMUSG00000047085 |
| 106 | 17475182 | NA | Zfp526 | zinc finger protein 526 | 210172 | ENSMUSG00000046541 |
| 107 | 17504327 | NA | Ndrg4 | N-myc downstream regulated gene 4 | 234593 | ENSMUSG00000036564 |
| 108 | 17538891 | NA | Iqsec2 | IQ motif and Sec7 domain 2 | 245666 | ENSMUSG00000041115 |
| 109 | 17313504 | NA | Srebf2 | sterol regulatory element binding factor 2 | 20788 | ENSMUSG00000022463 |
| 110 | 17515843 | NA | Kirrel3 | kin of IRRE like 3 (Drosophila) | 67703 | ENSMUSG00000032036 |
| 111 | 17373521 | NA | Chst1 | carbohydrate (keratan sulfate Gal-6) sulfotransferase 1 | 76969 | ENSMUSG00000027221 |
| 112 | 17346427 | NA | Khsrp | KH-type splicing regulatory protein | 16549 | ENSMUSG00000007670 |
| 113 | 17342719 | NA | Grm4 | glutamate receptor, metabotropic 4 | 268934 | ENSMUSG00000063239 |
| 114 | 17314164 | NA | Mapk8ip2 | mitogen-activated protein kinase 8 interacting protein 2 | 60597 | ENSMUSG00000022619 |
| 115 | 17377661 | NA | Scrt2 | scratch homolog 2, zinc finger protein (Drosophila) | 545474 | ENSMUSG00000060257 |
| 116 | 17247117 | NA | Zmiz2 | zinc finger, MIZ-type containing 2 | 52915 | ENSMUSG00000041164 |
| 117 | 17503122 | NA | Cacna1a | calcium channel, voltage-dependent, P/Q type, alpha 1A subunit | 12286 | ENSMUSG00000034656 |
| 118 | 17400521 | NA | Sv2a | synaptic vesicle glycoprotein 2 a | 64051 | ENSMUSG00000038486 |
| 119 | 17494596 | NA | Tpp1 | tripeptidyl peptidase I | 12751 | ENSMUSG00000030894 |
| 120 | 17513297 | NA | Cdyl2 | chromodomain protein, Y chromosome-like 2 | 75796 | ENSMUSG00000031758 |
| 121 | 17540059 | NA | Porcn | porcupine homolog (Drosophila) | 53627 | ENSMUSG00000031169 |
| 122 | 17308413 | NA | Epb4.9 | erythrocyte protein band 4.9 | 13829 | ENSMUSG00000022099 |
| 123 | 17263011 | NA | Glra1 | glycine receptor, alpha 1 subunit | 14654 | ENSMUSG00000000263 |
| 124 | 17253376 | NA | Sez6 | seizure related gene 6 | 20370 | ENSMUSG00000000632 |
| 125 | 17268120 | NA | Pdk2 | pyruvate dehydrogenase kinase, isoenzyme 2 | 18604 | ENSMUSG00000038967 |
| 126 | 17222256 | NA | Actr1b | ARP1 actin-related protein 1B, centractin beta | 226977 | ENSMUSG00000037351 |
| 127 | 17327765 | NA | Coro7 | coronin 7 | 78885 | ENSMUSG00000039637 |
| 128 | 17268786 | NA | Neurod2 | neurogenic differentiation 2 | 18013 | ENSMUSG00000038255 |
| 129 | 17475564 | NA | Numbl | numb-like | 18223 | ENSMUSG00000063160 |
| 130 | 17258683 | NA | Mgat5b | mannoside acetylglucosaminyltransferase 5, isoenzyme B | 268510 | ENSMUSG00000043857 |
| 131 | 17484419 | NA | Kndc1 | kinase non-catalytic C-lobe domain (KIND) containing 1 | 76484 | ENSMUSG00000066129 |
| 132 | 17425058 | NA | Coro2a | coronin, actin binding protein 2A | 107684 | ENSMUSG00000028337 |
| 133 | 17517073 | NA | Drd2 | dopamine receptor D2 | 13489 | ENSMUSG00000032259 |
| 134 | 17321582 | NA | Faim2 | Fas apoptotic inhibitory molecule 2 | 72393 | ENSMUSG00000023011 |
| 135 | 17536720 | NA | Nlgn3 | neuroligin 3 | 245537 | ENSMUSG00000031302 |
| 136 | 17478181 | NA | Kcnc1 | potassium voltage gated channel, Shaw-related subfamily, member 1 | 16502 | ENSMUSG00000058975 |
| 137 | 17475616 | NA | Hipk4 | homeodomain interacting protein kinase 4 | 233020 | ENSMUSG00000040424 |
| 138 | 17435570 | NA | Htr5a | 5-hydroxytryptamine (serotonin) receptor 5A | 15563 | ENSMUSG00000039106 |
| 139 | 17465332 | NA | Lrrc4 | leucine rich repeat containing 4 | 192198 | ENSMUSG00000049939 |
| 140 | 17473161 | NA | Cacng8 | calcium channel, voltage-dependent, gamma subunit 8 | 81905 | ENSMUSG00000053395 |
| 141 | 17451356 | NA | Sgsm1 | small G protein signaling modulator 1 | 52850 | ENSMUSG00000042216 |
| 142 | 17539019 | NA | Ubqln2 | ubiquilin 2 | 54609 | ENSMUSG00000050148 |
| 143 | 17259344 | NA | Hgs | HGF-regulated tyrosine kinase substrate | 15239 | ENSMUSG00000025793 |
| 144 | 17441453 | NA | Nos1 | nitric oxide synthase 1, neuronal | 18125 | ENSMUSG00000029361 |
| 145 | 17257492 | NA | Dcaf7 | DDB1 and CUL4 associated factor 7 | 71833 | ENSMUSG00000049354 |
| 146 | 17300484 | NA | Cpne6 | copine VI | 12891 | ENSMUSG00000022212 |
| 147 | 17257197 | NA | Mapt | microtubule-associated protein tau | 17762 | ENSMUSG00000018411 |
| 148 | 17269583 | NA | Zfp385c | zinc finger protein 385C | 278304 | ENSMUSG00000014198 |
| 149 | 17436877 | NA | Ablim2 | actin-binding LIM protein 2 | 231148 | ENSMUSG00000029095 |
| 150 | 17409005 | NA | Slc6a17 | solute carrier family 6 (neurotransmitter transporter), member 17 | 229706 | ENSMUSG00000027894 |
| 151 | 17485152 | NA | Brsk2 | BR serine/threonine kinase 2 | 75770 | ENSMUSG00000053046 |
| 152 | 17235300 | NA | Apc2 | adenomatosis polyposis coli 2 | 23805 | ENSMUSG00000020135 |
| 153 | 17454574 | NA | Ttyh3 | tweety homolog 3 (Drosophila) | 78339 | ENSMUSG00000036565 |
| 154 | 17523281 | NA | Trak1 | trafficking protein, kinesin binding 1 | 67095 | ENSMUSG00000032536 |
| 155 | 17420316 | NA | Rap1gap | Rap1 GTPase-activating protein | 110351 | ENSMUSG00000041351 |
| 156 | 17531999 | NA | Rbms3 | RNA binding motif, single stranded interacting protein | 207181 | ENSMUSG00000039607 |
| 157 | 17384021 | NA | Stxbp1 | syntaxin binding protein 1 | 20910 | ENSMUSG00000026797 |
| 158 | 17520198 | NA | Rasgrf1 | RAS protein-specific guanine nucleotide-releasing factor 1 | 19417 | ENSMUSG00000032356 |
| 159 | 17491285 | NA | Ptpn5 | protein tyrosine phosphatase, non-receptor type 5 | 19259 | ENSMUSG00000030854 |
| 160 | 17530733 | NA | Grm2 | glutamate receptor, metabotropic 2 | 108068 | ENSMUSG00000023192 |
| 161 | 17535627 | NA | Abcd1 | ATP-binding cassette, sub-family D (ALD), member 1 | 11666 | ENSMUSG00000031378 |
| 162 | 17474067 | NA | Slc8a2 | solute carrier family 8 (sodium/calcium exchanger), member 2 | 110891 | ENSMUSG00000030376 |
| 163 | 17465620 | NA | Podxl | podocalyxin-like | 27205 | ENSMUSG00000025608 |
| 164 | 17357959 | NA | Gnaq | guanine nucleotide binding protein, alpha q polypeptide | 14682 | ENSMUSG00000024639 |
| 165 | 17451482 | NA | Svop | SV2 related protein | 68666 | ENSMUSG00000042078 |
| 166 | 17266452 | NA | Sarm1 | sterile alpha and HEAT/Armadillo motif containing 1 | 237868 | ENSMUSG00000050132 |
| 167 | 17531080 | NA | Rnf123 | ring finger protein 123 | 84585 | ENSMUSG00000041528 |
| 168 | 17419587 | NA | Slc9a1 | solute carrier family 9 (sodium/hydrogen exchanger), member 1 | 20544 | ENSMUSG00000028854 |
| 169 | 17235211 | NA | Midn | midnolin | 59090 | ENSMUSG00000035621 |
| 170 | 17496452 | NA | Taok2 | TAO kinase 2 | 381921 | ENSMUSG00000059981 |
| 171 | 17517634 | NA | Sin3a | transcriptional regulator, SIN3A (yeast) | 20466 | ENSMUSG00000042557 |
| 172 | 17452719 | NA | Abcb9 | ATP-binding cassette, sub-family B (MDR/TAP), member 9 | 56325 | ENSMUSG00000029408 |
| 173 | 17471155 | NA | Kcna1 | potassium voltage-gated channel, shaker-related subfamily, member 1 | 16485 | ENSMUSG00000047976 |
| 174 | 17450059 | NA | Sec31a | Sec31 homolog A (S. cerevisiae) | 69162 | ENSMUSG00000035325 |
| 175 | 17355463 | NA | Ctif | CBP80/20-dependent translation initiation factor | 269037 | ENSMUSG00000052928 |
| 176 | 17461868 | NA | Slc6a1 | solute carrier family 6 (neurotransmitter transporter, GABA), member 1 | 232333 | ENSMUSG00000030310 |
| 177 | 17251303 | NA | Pik3r5 | phosphoinositide-3-kinase, regulatory subunit 5, p101 | 320207 | ENSMUSG00000020901 |
| 178 | 17441595 | NA | Tbx3 | T-box 3 | 21386 | ENSMUSG00000018604 |
| 179 | 17407272 | NA | Ints3 | integrator complex subunit 3 | 229543 | ENSMUSG00000027933 |
| 180 | 17297462 | NA | Sec24c | Sec24 related gene family, member C (S. cerevisiae) | 218811 | ENSMUSG00000039367 |
| 181 | 17361470 | NA | Klc2 | kinesin light chain 2 | 16594 | ENSMUSG00000024862 |
| 182 | 17359583 | NA | Cnnm1 | cyclin M1 | 83674 | ENSMUSG00000025189 |
| 183 | 17336987 | NA | Bag6 | BCL2-associated athanogene 6 | 224727 | ENSMUSG00000024392 |
| 184 | 17345527 | NA | Klhdc3 | kelch domain containing 3 | 71765 | ENSMUSG00000063576 |
| 185 | 17533640 | NA | Cdk16 | cyclin-dependent kinase 16 | 18555 | ENSMUSG00000031065 |
| 186 | 17345740 | NA | Foxp4 | forkhead box P4 | 74123 | ENSMUSG00000023991 |
| 187 | 17309287 | NA | Pou4f1 | POU domain, class 4, transcription factor 1 | 18996 | ENSMUSG00000048349 |
| 188 | 17318923 | NA | Cacng2 | calcium channel, voltage-dependent, gamma subunit 2 | 12300 | ENSMUSG00000019146 |
| 189 | 17272798 | NA | Cant1 | calcium activated nucleotidase 1 | 76025 | ENSMUSG00000025575 |
| 190 | 17521371 | NA | Tmem115 | transmembrane protein 115 | 56395 | ENSMUSG00000010045 |
| 191 | 17334545 | NA | Clcn7 | chloride channel 7 | 26373 | ENSMUSG00000036636 |
| 192 | 17243717 | NA | Syn3 | synapsin III | 27204 | ENSMUSG00000059602 |
| 193 | 17436999 | NA | Crmp1 | collapsin response mediator protein 1 | 12933 | ENSMUSG00000029121 |
| 194 | 17399496 | NA | Adar | adenosine deaminase, RNA-specific | 56417 | ENSMUSG00000027951 |
| 195 | 17329516 | NA | Fgf12 | fibroblast growth factor 12 | 14167 | ENSMUSG00000022523 |
| 196 | 17342065 | NA | Mapk8ip3 | mitogen-activated protein kinase 8 interacting protein 3 | 30957 | ENSMUSG00000024163 |
| 197 | 17506808 | NA | Trim67 | tripartite motif-containing 67 | 330863 | ENSMUSG00000036913 |
| 198 | 17406892 | NA | Rusc1 | RUN and SH3 domain containing 1 | 72296 | ENSMUSG00000041263 |
| 199 | 17226891 | NA | Cntn2 | contactin 2 | 21367 | ENSMUSG00000053024 |
| 200 | 17318523 | NA | Scrt1 | scratch homolog 1, zinc finger protein (Drosophila) | 170729 | ENSMUSG00000048385 |
| 201 | 17395766 | NA | Eef1a2 | eukaryotic translation elongation factor 1 alpha 2 | 13628 | ENSMUSG00000016349 |
| 202 | 17522338 | NA | Scap | SREBF chaperone | 235623 | ENSMUSG00000032485 |
| 203 | 17505599 | NA | Vac14 | Vac14 homolog (S. cerevisiae) | 234729 | ENSMUSG00000010936 |
| 204 | 17416325 | NA | Dhcr24 | 24-dehydrocholesterol reductase | 74754 | ENSMUSG00000034926 |
| 205 | 17351262 | NA | St8sia3 | ST8 alpha-N-acetyl-neuraminide alpha-2,8-sialyltransferase 3 | 20451 | ENSMUSG00000056812 |
| 206 | 17424279 | NA | Cntfr | ciliary neurotrophic factor receptor | 12804 | ENSMUSG00000028444 |
| 207 | 17505689 | NA | Aars | alanyl-tRNA synthetase | 234734 | ENSMUSG00000031960 |
| 208 | 17361223 | NA | Adrbk1 | adrenergic receptor kinase, beta 1 | 110355 | ENSMUSG00000024858 |
| 209 | 17401394 | NA | Kcnd3 | potassium voltage-gated channel, Shal-related family, member 3 | 56543 | ENSMUSG00000040896 |
| 210 | 17292753 | NA | Gprin1 | G protein-regulated inducer of neurite outgrowth 1 | 26913 | ENSMUSG00000069227 |
| 211 | 17328958 | NA | Gp1bb | glycoprotein Ib, beta polypeptide | 14724 | ENSMUSG00000050761 |
| 212 | 17450536 | NA | Barhl2 | BarH-like 2 (Drosophila) | 104382 | ENSMUSG00000034384 |
| 213 | 17453222 | NA | Wbscr17 | Williams-Beuren syndrome chromosome region 17 homolog (human) | 212996 | ENSMUSG00000034040 |
| 214 | 17307905 | NA | Dpysl2 | dihydropyrimidinase-like 2 | 12934 | ENSMUSG00000022048 |
| 215 | 17253276 | NA | Git1 | G protein-coupled receptor kinase-interactor 1 | 216963 | ENSMUSG00000011877 |
| 216 | 17440538 | NA | Galnt9 | UDP-N-acetyl-alpha-D-galactosamine:polypeptide N-acetylgalactosaminyltransferase 9 | 231605 | ENSMUSG00000033316 |
| 217 | 17273348 | NA | Fasn | fatty acid synthase | 14104 | ENSMUSG00000025153 |
| 218 | 17521327 | NA | Cacna2d2 | calcium channel, voltage-dependent, alpha 2/delta subunit 2 | 56808 | ENSMUSG00000010066 |
| 219 | 17292011 | NA | Tfap2a | transcription factor AP-2, alpha | 21418 | ENSMUSG00000021359 |
| 220 | 17318895 | NA | Foxred2 | FAD-dependent oxidoreductase domain containing 2 | 239554 | ENSMUSG00000016552 |
| 221 | 17477714 | NA | Slc17a7 | solute carrier family 17 (sodium-dependent inorganic phosphate cotransporter), member 7 | 72961 | ENSMUSG00000070570 |
| 222 | 17361494 | NA | Pacs1 | phosphofurin acidic cluster sorting protein 1 | 107975 | ENSMUSG00000024855 |
| 223 | 17293045 | NA | Spock1 | sparc/osteonectin, cwcv and kazal-like domains proteoglycan 1 | 20745 | ENSMUSG00000056222 |
| 224 | 17532879 | NA | Syp | synaptophysin | 20977 | ENSMUSG00000031144 |
| 225 | 17483194 | NA | Sez6l2 | seizure related 6 homolog like 2 | 233878 | ENSMUSG00000030683 |
| 226 | 17397377 | NA | Pcdh10 | protocadherin 10 | 18526 | ENSMUSG00000049100 |
| 227 | 17302289 | NA | Pcdh17 | protocadherin 17 | 219228 | ENSMUSG00000035566 |
| 228 | 17490452 | NA | Ap2a1 | adaptor protein complex AP-2, alpha 1 subunit | 11771 | ENSMUSG00000060279 |
| 229 | 17288442 | NA | Irx2 | Iroquois related homeobox 2 (Drosophila) | 16372 | ENSMUSG00000001504 |
| 230 | 17376993 | NA | Pcsk2 | proprotein convertase subtilisin/kexin type 2 | 18549 | ENSMUSG00000027419 |
| 231 | 17237915 | NA | Agap2 | ArfGAP with GTPase domain, ankyrin repeat and PH domain 2 | 216439 | ENSMUSG00000025422 |
| 232 | 17488312 | NA | Map3k10 | mitogen-activated protein kinase kinase kinase 10 | 269881 | ENSMUSG00000040390 |
| 233 | 17268884 | NA | Nr1d1 | nuclear receptor subfamily 1, group D, member 1 | 217166 | ENSMUSG00000020889 |
| 234 | 17417702 | NA | Slc6a9 | solute carrier family 6 (neurotransmitter transporter, glycine), member 9 | 14664 | ENSMUSG00000028542 |
| 235 | 17510013 | NA | Upf1 | UPF1 regulator of nonsense transcripts homolog (yeast) | 19704 | ENSMUSG00000058301 |
| 236 | 17502603 | NA | Rasd2 | RASD family, member 2 | 75141 | ENSMUSG00000034472 |
| 237 | 17235037 | NA | Arid3a | AT rich interactive domain 3A (BRIGHT-like) | 13496 | ENSMUSG00000019564 |
| 238 | 17541383 | NA | Zdhhc9 | zinc finger, DHHC domain containing 9 | 208884 | ENSMUSG00000036985 |
| 239 | 17504130 | NA | Cx3cl1 | chemokine (C-X3-C motif) ligand 1 | 20312 | ENSMUSG00000031778 |
| 240 | 17382496 | NA | Grin1 | glutamate receptor, ionotropic, NMDA1 (zeta 1) | 14810 | ENSMUSG00000026959 |
| 241 | 17367921 | NA | Grin1 | glutamate receptor, ionotropic, NMDA1 (zeta 1) | 14810 | ENSMUSG00000026959 |
| 242 | 17475851 | NA | Lrfn1 | leucine rich repeat and fibronectin type III domain containing 1 | 80749 | NULL |
| 243 | 17266107 | NA | Abr | active BCR-related gene | 109934 | ENSMUSG00000017631 |
| 244 | 17279640 | NA | Ptprn2 | protein tyrosine phosphatase, receptor type, N polypeptide 2 | 19276 | ENSMUSG00000056553 |
| 245 | 17245539 | NA | Srgap1 | SLIT-ROBO Rho GTPase activating protein 1 | 117600 | ENSMUSG00000020121 |
| 246 | 17376167 | NA | Sirpa | signal-regulatory protein alpha | 19261 | ENSMUSG00000037902 |
| 247 | 17268849 | NA | Med24 | mediator complex subunit 24 | 23989 | ENSMUSG00000017210 |
| 248 | 17319207 | NA | Csnk1e | casein kinase 1, epsilon | 27373 | ENSMUSG00000022433 |
| 249 | 17518007 | NA | Pkm | pyruvate kinase, muscle | 18746 | ENSMUSG00000032294 |
| 250 | 17406760 | NA | Sema4a | sema domain, immunoglobulin domain (Ig), transmembrane domain (TM) and short cytoplasmic domain, (semaphorin) 4A | 20351 | ENSMUSG00000028064 |
| 251 | 17342509 | NA | Pigq | phosphatidylinositol glycan anchor biosynthesis, class Q | 14755 | ENSMUSG00000025728 |
| 252 | 17243330 | NA | Nfic | nuclear factor I/C | 18029 | ENSMUSG00000055053 |
| 253 | 17222156 | NA | Kansl3 | KAT8 regulatory NSL complex subunit 3 | 226976 | ENSMUSG00000010453 |
| 254 | 17385374 | NA | Nr4a2 | nuclear receptor subfamily 4, group A, member 2 | 18227 | ENSMUSG00000026826 |
| 255 | 17451223 | NA | Sez6l | seizure related 6 homolog like | 56747 | ENSMUSG00000058153 |
| 256 | 17436944 | NA | Ppp2r2c | protein phosphatase 2 (formerly 2A), regulatory subunit B (PR 52), gamma isoform | 269643 | ENSMUSG00000029120 |
| 257 | 17532953 | NA | Tfe3 | transcription factor E3 | 209446 | ENSMUSG00000000134 |
| 258 | 17262065 | NA | Cyfip2 | cytoplasmic FMR1 interacting protein 2 | 76884 | ENSMUSG00000020340 |
| 259 | 17425095 | NA | Gabbr2 | gamma-aminobutyric acid (GABA) B receptor, 2 | 242425 | ENSMUSG00000039809 |
| 260 | 17339554 | NA | Lbh | limb-bud and heart | 77889 | ENSMUSG00000024063 |
| 261 | 17354653 | NA | Tcof1 | Treacher Collins Franceschetti syndrome 1, homolog | 21453 | ENSMUSG00000024613 |
| 262 | 17408211 | NA | Pde4dip | phosphodiesterase 4D interacting protein (myomegalin) | 83679 | ENSMUSG00000038170 |
| 263 | 17335145 | NA | Pacsin1 | protein kinase C and casein kinase substrate in neurons 1 | 23969 | ENSMUSG00000040276 |
| 264 | 17243229 | NA | Atcay | ataxia, cerebellar, Cayman type homolog (human) | 16467 | ENSMUSG00000034958 |
| 265 | 17498607 | NA | Mcoln1 | mucolipin 1 | 94178 | ENSMUSG00000004567 |
| 266 | 17255788 | NA | Arhgap23 | Rho GTPase activating protein 23 | 58996 | ENSMUSG00000049807 |
| 267 | 17249811 | NA | Slc36a1 | solute carrier family 36 (proton/amino acid symporter), member 1 | 215335 | ENSMUSG00000020261 |
| 268 | 17503884 | NA | Gnao1 | guanine nucleotide binding protein, alpha O | 14681 | ENSMUSG00000031748 |
| 269 | 17248691 | NA | Ebf1 | early B cell factor 1 | 13591 | ENSMUSG00000057098 |
| 270 | 17383858 | NA | Dnm1 | dynamin 1 | 13429 | ENSMUSG00000026825 |
| 271 | 17364665 | NA | Slit1 | slit homolog 1 (Drosophila) | 20562 | ENSMUSG00000025020 |
| 272 | 17224500 | NA | Atg9a | autophagy related 9A | 245860 | ENSMUSG00000033124 |
| 273 | 17510696 | NA | Pou4f2 | POU domain, class 4, transcription factor 2 | 18997 | ENSMUSG00000031688 |
| 274 | 17504399 | NA | Cdh5 | cadherin 5 | 12562 | ENSMUSG00000031871 |
| 275 | 17498041 | NA | Dusp8 | dual specificity phosphatase 8 | 18218 | ENSMUSG00000037887 |
| 276 | 17497904 | NA | Slc25a22 | solute carrier family 25 (mitochondrial carrier, glutamate), member 22 | 68267 | ENSMUSG00000019082 |
| 277 | 17368685 | NA | Ralgds | ral guanine nucleotide dissociation stimulator | 19730 | ENSMUSG00000026821 |
| 278 | 17401650 | NA | Amigo1 | adhesion molecule with Ig like domain 1 | 229715 | ENSMUSG00000050947 |
| 279 | 17424128 | NA | Nol6 | nucleolar protein family 6 (RNA-associated) | 230082 | ENSMUSG00000028430 |
| 280 | 17529930 | NA | Clstn2 | calsyntenin 2 | 64085 | ENSMUSG00000032452 |
| 281 | 17516837 | NA | Bace1 | beta-site APP cleaving enzyme 1 | 23821 | ENSMUSG00000032086 |
| 282 | 17548746 | NA | Bace1 | beta-site APP cleaving enzyme 1 | 23821 | ENSMUSG00000032086 |
| 283 | 17234936 | NA | Hcn2 | hyperpolarization-activated, cyclic nucleotide-gated K+ 2 | 15166 | ENSMUSG00000020331 |
| 284 | 17272817 | NA | Rbfox3 | RNA binding protein, fox-1 homolog (C. elegans) 3 | 52897 | ENSMUSG00000025576 |
| 285 | 17267039 | NA | Lhx1 | LIM homeobox protein 1 | 16869 | ENSMUSG00000018698 |
| 286 | 17259810 | NA | Inpp5j | inositol polyphosphate 5-phosphatase J | 170835 | ENSMUSG00000034570 |
| 287 | 17235556 | NA | Zbtb7a | zinc finger and BTB domain containing 7a | 16969 | ENSMUSG00000035011 |
| 288 | 17242281 | NA | Adarb1 | adenosine deaminase, RNA-specific, B1 | 110532 | ENSMUSG00000020262 |
| 289 | 17454995 | NA | Tmem130 | transmembrane protein 130 | 243339 | ENSMUSG00000043388 |
| 290 | 17419097 | NA | Bai2 | brain-specific angiogenesis inhibitor 2 | 230775 | ENSMUSG00000028782 |
| 291 | 17516462 | NA | Thy1 | thymus cell antigen 1, theta | 21838 | ENSMUSG00000032011 |
| 292 | 17314051 | NA | Panx2 | pannexin 2 | 406218 | ENSMUSG00000058441 |
| 293 | 17461923 | NA | Syn2 | synapsin II | 20965 | ENSMUSG00000009394 |
| 294 | 17436545 | NA | Nat8l | N-acetyltransferase 8-like | 269642 | ENSMUSG00000048142 |
| 295 | 17388406 | NA | Slc35c1 | solute carrier family 35, member C1 | 228368 | ENSMUSG00000049922 |
| 296 | 17529218 | NA | Htr1b | 5-hydroxytryptamine (serotonin) receptor 1B | 15551 | ENSMUSG00000049511 |
| 297 | 17284065 | NA | Cdc42bpb | CDC42 binding protein kinase beta | 217866 | ENSMUSG00000021279 |
| 298 | 17238605 | NA | Itga7 | integrin alpha 7 | 16404 | ENSMUSG00000025348 |
| 299 | 17354629 | NA | Ndst1 | N-deacetylase/N-sulfotransferase (heparan glucosaminyl) 1 | 15531 | ENSMUSG00000054008 |
| 300 | 17328870 | NA | Zdhhc8 | zinc finger, DHHC domain containing 8 | 27801 | ENSMUSG00000060166 |
| 301 | 17270028 | NA | Mpp2 | membrane protein, palmitoylated 2 (MAGUK p55 subfamily member 2) | 50997 | ENSMUSG00000017314 |
| 302 | 17308939 | NA | Pcdh8 | protocadherin 8 | 18530 | ENSMUSG00000036422 |
| 303 | 17388261 | NA | Atg13 | autophagy related 13 | 51897 | ENSMUSG00000027244 |
| 304 | 17341608 | NA | Flywch1 | FLYWCH-type zinc finger 1 | 224613 | ENSMUSG00000040097 |
| 305 | 17509907 | NA | Gatad2a | GATA zinc finger domain containing 2A | 234366 | ENSMUSG00000036180 |
| 306 | 17303897 | NA | Ndst2 | N-deacetylase/N-sulfotransferase (heparan glucosaminyl) 2 | 17423 | ENSMUSG00000039308 |
| 307 | 17400617 | NA | Ankrd34a | ankyrin repeat domain 34A | 545554 | ENSMUSG00000049097 |
| 308 | 17461897 | NA | Atg7 | autophagy related 7 | 74244 | ENSMUSG00000030314 |
| 309 | 17488292 | NA | Pld3 | phospholipase D family, member 3 | 18807 | ENSMUSG00000003363 |
| 310 | 17542419 | NA | L1cam | L1 cell adhesion molecule | 16728 | ENSMUSG00000031391 |
| 311 | 17271158 | NA | Cacng5 | calcium channel, voltage-dependent, gamma subunit 5 | 140723 | ENSMUSG00000040373 |
| 312 | 17332495 | NA | Dscam | Down syndrome cell adhesion molecule | 13508 | ENSMUSG00000050272 |
| 313 | 17344251 | NA | Prrc2a | proline-rich coiled-coil 2A | 53761 | ENSMUSG00000024393 |
| 314 | 17378450 | NA | Mmp24 | matrix metallopeptidase 24 | 17391 | ENSMUSG00000027612 |
| 315 | 17338136 | NA | Cul7 | cullin 7 | 66515 | ENSMUSG00000038545 |
| 316 | 17314636 | NA | Cacnb3 | calcium channel, voltage-dependent, beta 3 subunit | 12297 | ENSMUSG00000003352 |
| 317 | 17273714 | NA | Adcy3 | adenylate cyclase 3 | 104111 | ENSMUSG00000020654 |
| 318 | 17477946 | NA | Mamstr | MEF2 activating motif and SAP domain containing transcriptional regulator | 74490 | ENSMUSG00000042918 |
| 319 | 17517752 | NA | Ulk3 | unc-51-like kinase 3 | 71742 | ENSMUSG00000032308 |
| 320 | 17515106 | NA | Pde4a | phosphodiesterase 4A, cAMP specific | 18577 | ENSMUSG00000032177 |
| 321 | 17243157 | NA | Diras1 | DIRAS family, GTP-binding RAS-like 1 | 208666 | ENSMUSG00000043670 |
| 322 | 17506443 | NA | Acsf3 | acyl-CoA synthetase family member 3 | 257633 | ENSMUSG00000015016 |
| 323 | 17290835 | NA | Pou6f2 | POU domain, class 6, transcription factor 2 | 218030 | ENSMUSG00000009734 |
| 324 | 17308299 | NA | Slc39a14 | solute carrier family 39 (zinc transporter), member 14 | 213053 | ENSMUSG00000022094 |
| 325 | 17369147 | NA | Nup188 | nucleoporin 188 | 227699 | ENSMUSG00000052533 |
| 326 | 17237937 | NA | B4galnt1 | beta-1,4-N-acetyl-galactosaminyl transferase 1 | 14421 | ENSMUSG00000006731 |
| 327 | 17283935 | NA | Wars | tryptophanyl-tRNA synthetase | 22375 | ENSMUSG00000021266 |
| 328 | 17474157 | NA | Strn4 | striatin, calmodulin binding protein 4 | 97387 | ENSMUSG00000030374 |
| 329 | 17292346 | NA | Phf2 | PHD finger protein 2 | 18676 | ENSMUSG00000038025 |
| 330 | 17383320 | NA | Brd3 | bromodomain containing 3 | 67382 | ENSMUSG00000026918 |
| 331 | 17377144 | NA | Slc24a3 | solute carrier family 24 (sodium/potassium/calcium exchanger), member 3 | 94249 | ENSMUSG00000063873 |
| 332 | 17513806 | NA | Cbfa2t3 | core-binding factor, runt domain, alpha subunit 2, translocated to, 3 (human) | 12398 | ENSMUSG00000006362 |
| 333 | 17346387 | NA | Mllt1 | myeloid/lymphoid or mixed-lineage leukemia (trithorax homolog, Drosophila); translocated to, 1 | 64144 | ENSMUSG00000024212 |
| 334 | 17243057 | NA | Ap3d1 | adaptor-related protein complex 3, delta 1 subunit | 11776 | ENSMUSG00000020198 |
| 335 | 17271622 | NA | Cdc42ep4 | CDC42 effector protein (Rho GTPase binding) 4 | 56699 | ENSMUSG00000041598 |
| 336 | 17272926 | NA | Nptx1 | neuronal pentraxin 1 | 18164 | ENSMUSG00000025582 |
| 337 | 17398785 | NA | Arhgef11 | Rho guanine nucleotide exchange factor (GEF) 11 | 213498 | ENSMUSG00000041977 |
| 338 | 17361779 | NA | Scyl1 | SCY1-like 1 (S. cerevisiae) | 78891 | ENSMUSG00000024941 |
| 339 | 17370883 | NA | Kcnj3 | potassium inwardly-rectifying channel, subfamily J, member 3 | 16519 | ENSMUSG00000026824 |
| 340 | 17234116 | NA | Ccdc6 | coiled-coil domain containing 6 | 76551 | ENSMUSG00000048701 |
| 341 | 17346155 | NA | Sema6b | sema domain, transmembrane domain (TM), and cytoplasmic domain, (semaphorin) 6B | 20359 | ENSMUSG00000001227 |
| 342 | 17516564 | NA | Hyou1 | hypoxia up-regulated 1 | 12282 | ENSMUSG00000032115 |
| 343 | 17346185 | NA | Dpp9 | dipeptidylpeptidase 9 | 224897 | ENSMUSG00000001229 |
| 344 | 17235663 | NA | Pip5k1c | phosphatidylinositol-4-phosphate 5-kinase, type 1 gamma | 18717 | ENSMUSG00000034902 |
| 345 | 17378721 | NA | Src | Rous sarcoma oncogene | 20779 | ENSMUSG00000027646 |
| 346 | 17313023 | NA | Gtpbp1 | GTP binding protein 1 | 14904 | ENSMUSG00000042535 |
| 347 | 17343405 | NA | Wiz | widely-interspaced zinc finger motifs | 22404 | ENSMUSG00000024050 |
| 348 | 17399044 | NA | Smg5 | Smg-5 homolog, nonsense mediated mRNA decay factor (C. elegans) | 229512 | ENSMUSG00000001415 |
| 349 | 17336660 | NA | Atf6b | activating transcription factor 6 beta | 12915 | ENSMUSG00000015461 |
| 350 | 17500301 | NA | Gpr124 | G protein-coupled receptor 124 | 78560 | ENSMUSG00000031486 |
| 351 | 17242729 | NA | Med16 | mediator complex subunit 16 | 216154 | ENSMUSG00000013833 |
| 352 | 17486930 | NA | Grlf1 | glucocorticoid receptor DNA binding factor 1 | 232906 | ENSMUSG00000058230 |
| 353 | 17334846 | NA | Tmem8 | transmembrane protein 8 (five membrane-spanning domains) | 60455 | ENSMUSG00000024180 |
| 354 | 17325206 | NA | Adcy5 | adenylate cyclase 5 | 224129 | ENSMUSG00000022840 |
| 355 | 17490312 | NA | Nr1h2 | nuclear receptor subfamily 1, group H, member 2 | 22260 | ENSMUSG00000060601 |
| 356 | 17264960 | NA | Fgf11 | fibroblast growth factor 11 | 14166 | ENSMUSG00000042826 |
| 357 | 17219519 | NA | Igsf8 | immunoglobulin superfamily, member 8 | 140559 | ENSMUSG00000038034 |
| 358 | 17359960 | NA | Gbf1 | golgi-specific brefeldin A-resistance factor 1 | 107338 | ENSMUSG00000025224 |
| 359 | 17516731 | NA | Scn2b | sodium channel, voltage-gated, type II, beta | 72821 | ENSMUSG00000070304 |
| 360 | 17338210 | NA | Trerf1 | transcriptional regulating factor 1 | 224829 | ENSMUSG00000064043 |
| 361 | 17227589 | NA | Lhx9 | LIM homeobox protein 9 | 16876 | ENSMUSG00000019230 |
| 362 | 17276622 | NA | Fntb | farnesyltransferase, CAAX box, beta | 110606 | ENSMUSG00000033373 |
| 363 | 17360440 | NA | Adra2a | adrenergic receptor, alpha 2a | 11551 | ENSMUSG00000033717 |
| 364 | 17260261 | NA | Camk2b | calcium/calmodulin-dependent protein kinase II, beta | 12323 | ENSMUSG00000057897 |
| 365 | 17322359 | NA | Zfp385a | zinc finger protein 385A | 29813 | ENSMUSG00000000552 |
| 366 | 17389647 | NA | Rasgrp1 | RAS guanyl releasing protein 1 | 19419 | ENSMUSG00000027347 |

  
  

| **Database:cellular component      &nbspName:membrane      &nbspID:GO:0016020** | | | | | | |
| --- | --- | --- | --- | --- | --- | --- |
| C=7887; O=258; E=178.53; R=1.45; rawP=3.37e-14; adjP=1.04e-12 | | | | | | |
| Index | UserID | Value | Gene Symbol | Gene Name | EntrezGene | Ensembl |
| 1 | 17318923 | NA | Cacng2 | calcium channel, voltage-dependent, gamma subunit 2 | 12300 | ENSMUSG00000019146 |
| 2 | 17378922 | NA | Slc32a1 | solute carrier family 32 (GABA vesicular transporter), member 1 | 22348 | ENSMUSG00000037771 |
| 3 | 17242822 | NA | Dos | downstream of Stk11 | 100503659 | ENSMUSG00000035640 |
| 4 | 17521371 | NA | Tmem115 | transmembrane protein 115 | 56395 | ENSMUSG00000010045 |
| 5 | 17272798 | NA | Cant1 | calcium activated nucleotidase 1 | 76025 | ENSMUSG00000025575 |
| 6 | 17334545 | NA | Clcn7 | chloride channel 7 | 26373 | ENSMUSG00000036636 |
| 7 | 17362646 | NA | Dagla | diacylglycerol lipase, alpha | 269060 | ENSMUSG00000035735 |
| 8 | 17461852 | NA | Slc6a11 | solute carrier family 6 (neurotransmitter transporter, GABA), member 11 | 243616 | ENSMUSG00000030307 |
| 9 | 17243717 | NA | Syn3 | synapsin III | 27204 | ENSMUSG00000059602 |
| 10 | 17376685 | NA | Plcb1 | phospholipase C, beta 1 | 18795 | ENSMUSG00000051177 |
| 11 | 17342065 | NA | Mapk8ip3 | mitogen-activated protein kinase 8 interacting protein 3 | 30957 | ENSMUSG00000024163 |
| 12 | 17406892 | NA | Rusc1 | RUN and SH3 domain containing 1 | 72296 | ENSMUSG00000041263 |
| 13 | 17515358 | NA | BC018242 | cDNA sequence BC018242 | 235044 | ENSMUSG00000040563 |
| 14 | 17226891 | NA | Cntn2 | contactin 2 | 21367 | ENSMUSG00000053024 |
| 15 | 17252995 | NA | Slc43a2 | solute carrier family 43, member 2 | 215113 | ENSMUSG00000038178 |
| 16 | 17522338 | NA | Scap | SREBF chaperone | 235623 | ENSMUSG00000032485 |
| 17 | 17361703 | NA | Pcnxl3 | pecanex-like 3 (Drosophila) | 104401 | ENSMUSG00000054874 |
| 18 | 17322600 | NA | Mgrn1 | mahogunin, ring finger 1 | 17237 | ENSMUSG00000022517 |
| 19 | 17505599 | NA | Vac14 | Vac14 homolog (S. cerevisiae) | 234729 | ENSMUSG00000010936 |
| 20 | 17416325 | NA | Dhcr24 | 24-dehydrocholesterol reductase | 74754 | ENSMUSG00000034926 |
| 21 | 17336636 | NA | Agpat1 | 1-acylglycerol-3-phosphate O-acyltransferase 1 (lysophosphatidic acid acyltransferase, alpha) | 55979 | ENSMUSG00000034254 |
| 22 | 17339313 | NA | Epb4.1l3 | erythrocyte protein band 4.1-like 3 | 13823 | ENSMUSG00000024044 |
| 23 | 17424279 | NA | Cntfr | ciliary neurotrophic factor receptor | 12804 | ENSMUSG00000028444 |
| 24 | 17351262 | NA | St8sia3 | ST8 alpha-N-acetyl-neuraminide alpha-2,8-sialyltransferase 3 | 20451 | ENSMUSG00000056812 |
| 25 | 17370234 | NA | Dab2ip | disabled 2 interacting protein | 69601 | ENSMUSG00000026883 |
| 26 | 17361223 | NA | Adrbk1 | adrenergic receptor kinase, beta 1 | 110355 | ENSMUSG00000024858 |
| 27 | 17320813 | NA | Nell2 | NEL-like 2 (chicken) | 54003 | ENSMUSG00000022454 |
| 28 | 17469814 | NA | Atp2b2 | ATPase, Ca++ transporting, plasma membrane 2 | 11941 | ENSMUSG00000030302 |
| 29 | 17401394 | NA | Kcnd3 | potassium voltage-gated channel, Shal-related family, member 3 | 56543 | ENSMUSG00000040896 |
| 30 | 17292753 | NA | Gprin1 | G protein-regulated inducer of neurite outgrowth 1 | 26913 | ENSMUSG00000069227 |
| 31 | 17346231 | NA | Ptprs | protein tyrosine phosphatase, receptor type, S | 19280 | ENSMUSG00000013236 |
| 32 | 17487805 | NA | Atp1a3 | ATPase, Na+/K+ transporting, alpha 3 polypeptide | 232975 | ENSMUSG00000040907 |
| 33 | 17328958 | NA | Gp1bb | glycoprotein Ib, beta polypeptide | 14724 | ENSMUSG00000050761 |
| 34 | 17502039 | NA | Rab3a | RAB3A, member RAS oncogene family | 19339 | ENSMUSG00000031840 |
| 35 | 17453222 | NA | Wbscr17 | Williams-Beuren syndrome chromosome region 17 homolog (human) | 212996 | ENSMUSG00000034040 |
| 36 | 17242707 | NA | BC005764 | cDNA sequence BC005764 | 216152 | ENSMUSG00000035835 |
| 37 | 17526206 | NA | C2cd2l | C2 calcium-dependent domain containing 2-like | 71764 | ENSMUSG00000032120 |
| 38 | 17239234 | NA | Grm1 | glutamate receptor, metabotropic 1 | 14816 | ENSMUSG00000019828 |
| 39 | 17409343 | NA | 5330417C22Rik | RIKEN cDNA 5330417C22 gene | 229722 | ENSMUSG00000040412 |
| 40 | 17224146 | NA | March4 | membrane-associated ring finger (C3HC4) 4 | 381270 | ENSMUSG00000039372 |
| 41 | 17236102 | NA | Btbd11 | BTB (POZ) domain containing 11 | 74007 | ENSMUSG00000020042 |
| 42 | 17440538 | NA | Galnt9 | UDP-N-acetyl-alpha-D-galactosamine:polypeptide N-acetylgalactosaminyltransferase 9 | 231605 | ENSMUSG00000033316 |
| 43 | 17345262 | NA | Tmem63b | transmembrane protein 63b | 224807 | ENSMUSG00000036026 |
| 44 | 17540501 | NA | Syn1 | synapsin I | 20964 | ENSMUSG00000037217 |
| 45 | 17298874 | NA | Grid1 | glutamate receptor, ionotropic, delta 1 | 14803 | ENSMUSG00000041078 |
| 46 | 17349607 | NA | Psd2 | pleckstrin and Sec7 domain containing 2 | 74002 | ENSMUSG00000024347 |
| 47 | 17413436 | NA | Tmem8b | transmembrane protein 8B | 242409 | ENSMUSG00000078716 |
| 48 | 17273348 | NA | Fasn | fatty acid synthase | 14104 | ENSMUSG00000025153 |
| 49 | 17521327 | NA | Cacna2d2 | calcium channel, voltage-dependent, alpha 2/delta subunit 2 | 56808 | ENSMUSG00000010066 |
| 50 | 17473155 | NA | Cacng7 | calcium channel, voltage-dependent, gamma subunit 7 | 81904 | ENSMUSG00000069806 |
| 51 | 17431720 | NA | Alpl | alkaline phosphatase, liver/bone/kidney | 11647 | ENSMUSG00000028766 |
| 52 | 17501692 | NA | Atp13a1 | ATPase type 13A1 | 170759 | ENSMUSG00000031862 |
| 53 | 17477714 | NA | Slc17a7 | solute carrier family 17 (sodium-dependent inorganic phosphate cotransporter), member 7 | 72961 | ENSMUSG00000070570 |
| 54 | 17525075 | NA | Herpud2 | HERPUD family member 2 | 80517 | ENSMUSG00000008429 |
| 55 | 17319339 | NA | Npcd | neuronal pentraxin chromo domain | 504193 | ENSMUSG00000089837 ENSMUSG00000022421 |
| 56 | 17243162 | NA | Slc39a3 | solute carrier family 39 (zinc transporter), member 3 | 106947 | ENSMUSG00000046822 |
| 57 | 17317801 | NA | Kcnk9 | potassium channel, subfamily K, member 9 | 223604 | ENSMUSG00000036760 |
| 58 | 17457465 | NA | Clec2l | C-type lectin domain family, member L | 665180 | ENSMUSG00000079598 |
| 59 | 17527520 | NA | Scamp5 | secretory carrier membrane protein 5 | 56807 | ENSMUSG00000040722 |
| 60 | 17532879 | NA | Syp | synaptophysin | 20977 | ENSMUSG00000031144 |
| 61 | 17483194 | NA | Sez6l2 | seizure related 6 homolog like 2 | 233878 | ENSMUSG00000030683 |
| 62 | 17215820 | NA | Gpc1 | glypican 1 | 14733 | ENSMUSG00000034220 |
| 63 | 17518342 | NA | Megf11 | multiple EGF-like-domains 11 | 214058 | ENSMUSG00000036466 |
| 64 | 17397377 | NA | Pcdh10 | protocadherin 10 | 18526 | ENSMUSG00000049100 |
| 65 | 17302289 | NA | Pcdh17 | protocadherin 17 | 219228 | ENSMUSG00000035566 |
| 66 | 17490452 | NA | Ap2a1 | adaptor protein complex AP-2, alpha 1 subunit | 11771 | ENSMUSG00000060279 |
| 67 | 17312341 | NA | Grina | glutamate receptor, ionotropic, N-methyl D-aspartate-associated protein 1 (glutamate binding) | 66168 | ENSMUSG00000022564 |
| 68 | 17376993 | NA | Pcsk2 | proprotein convertase subtilisin/kexin type 2 | 18549 | ENSMUSG00000027419 |
| 69 | 17313106 | NA | Cacna1i | calcium channel, voltage-dependent, alpha 1I subunit | 239556 | ENSMUSG00000022416 |
| 70 | 17484409 | NA | Gpr123 | G protein-coupled receptor 123 | 52389 | ENSMUSG00000025475 |
| 71 | 17373530 | NA | Syt13 | synaptotagmin XIII | 80976 | ENSMUSG00000027220 |
| 72 | 17451140 | NA | Ulk1 | unc-51 like kinase 1 | 22241 | ENSMUSG00000029512 |
| 73 | 17417702 | NA | Slc6a9 | solute carrier family 6 (neurotransmitter transporter, glycine), member 9 | 14664 | ENSMUSG00000028542 |
| 74 | 17477454 | NA | Syt3 | synaptotagmin III | 20981 | ENSMUSG00000030731 |
| 75 | 17545450 | NA | Gpr173 | G-protein coupled receptor 173 | 70771 | ENSMUSG00000056679 |
| 76 | 17452139 | NA | Rph3a | rabphilin 3A | 19894 | ENSMUSG00000029608 |
| 77 | 17337513 | NA | Gabbr1 | gamma-aminobutyric acid (GABA) B receptor, 1 | 54393 | ENSMUSG00000024462 |
| 78 | 17498906 | NA | Myo16 | myosin XVI | 244281 | ENSMUSG00000039057 |
| 79 | 17502603 | NA | Rasd2 | RASD family, member 2 | 75141 | ENSMUSG00000034472 |
| 80 | 17541383 | NA | Zdhhc9 | zinc finger, DHHC domain containing 9 | 208884 | ENSMUSG00000036985 |
| 81 | 17235037 | NA | Arid3a | AT rich interactive domain 3A (BRIGHT-like) | 13496 | ENSMUSG00000019564 |
| 82 | 17302600 | NA | Slitrk5 | SLIT and NTRK-like family, member 5 | 75409 | ENSMUSG00000033214 |
| 83 | 17489052 | NA | Aplp1 | amyloid beta (A4) precursor-like protein 1 | 11803 | ENSMUSG00000006651 |
| 84 | 17432808 | NA | Mfn2 | mitofusin 2 | 170731 | ENSMUSG00000029020 |
| 85 | 17504130 | NA | Cx3cl1 | chemokine (C-X3-C motif) ligand 1 | 20312 | ENSMUSG00000031778 |
| 86 | 17468364 | NA | Rab11fip5 | RAB11 family interacting protein 5 (class I) | 52055 | ENSMUSG00000051343 |
| 87 | 17382496 | NA | Grin1 | glutamate receptor, ionotropic, NMDA1 (zeta 1) | 14810 | ENSMUSG00000026959 |
| 88 | 17367921 | NA | Grin1 | glutamate receptor, ionotropic, NMDA1 (zeta 1) | 14810 | ENSMUSG00000026959 |
| 89 | 17475851 | NA | Lrfn1 | leucine rich repeat and fibronectin type III domain containing 1 | 80749 | NULL |
| 90 | 17266107 | NA | Abr | active BCR-related gene | 109934 | ENSMUSG00000017631 |
| 91 | 17474400 | NA | Sympk | symplekin | 68188 | ENSMUSG00000023118 |
| 92 | 17279640 | NA | Ptprn2 | protein tyrosine phosphatase, receptor type, N polypeptide 2 | 19276 | ENSMUSG00000056553 |
| 93 | 17214665 | NA | Sgpp2 | sphingosine-1-phosphate phosphotase 2 | 433323 | ENSMUSG00000032908 |
| 94 | 17347948 | NA | Kcnk12 | potassium channel, subfamily K, member 12 | 210741 | ENSMUSG00000050138 |
| 95 | 17334205 | NA | Abca3 | ATP-binding cassette, sub-family A (ABC1), member 3 | 27410 | ENSMUSG00000024130 |
| 96 | 17376167 | NA | Sirpa | signal-regulatory protein alpha | 19261 | ENSMUSG00000037902 |
| 97 | 17446123 | NA | Kcnh2 | potassium voltage-gated channel, subfamily H (eag-related), member 2 | 16511 | ENSMUSG00000038319 |
| 98 | 17269464 | NA | Jup | junction plakoglobin | 16480 | ENSMUSG00000001552 |
| 99 | 17418732 | NA | Dlgap3 | discs, large (Drosophila) homolog-associated protein 3 | 242667 | ENSMUSG00000042388 |
| 100 | 17301823 | NA | Gfra2 | glial cell line derived neurotrophic factor family receptor alpha 2 | 14586 | ENSMUSG00000022103 |
| 101 | 17404570 | NA | Slc7a14 | solute carrier family 7 (cationic amino acid transporter, y+ system), member 14 | 241919 | ENSMUSG00000069072 |
| 102 | 17406760 | NA | Sema4a | sema domain, immunoglobulin domain (Ig), transmembrane domain (TM) and short cytoplasmic domain, (semaphorin) 4A | 20351 | ENSMUSG00000028064 |
| 103 | 17518007 | NA | Pkm | pyruvate kinase, muscle | 18746 | ENSMUSG00000032294 |
| 104 | 17342509 | NA | Pigq | phosphatidylinositol glycan anchor biosynthesis, class Q | 14755 | ENSMUSG00000025728 |
| 105 | 17374618 | NA | Disp2 | dispatched homolog 2 (Drosophila) | 214240 | ENSMUSG00000040035 |
| 106 | 17535572 | NA | Atp2b3 | ATPase, Ca++ transporting, plasma membrane 3 | 320707 | ENSMUSG00000031376 |
| 107 | 17451223 | NA | Sez6l | seizure related 6 homolog like | 56747 | ENSMUSG00000058153 |
| 108 | 17222625 | NA | Tgfbrap1 | transforming growth factor, beta receptor associated protein 1 | 73122 | ENSMUSG00000070939 |
| 109 | 17354299 | NA | Sema6a | sema domain, transmembrane domain (TM), and cytoplasmic domain, (semaphorin) 6A | 20358 | ENSMUSG00000019647 |
| 110 | 17435528 | NA | Dpp6 | dipeptidylpeptidase 6 | 13483 | ENSMUSG00000061576 |
| 111 | 17535607 | NA | Slc6a8 | solute carrier family 6 (neurotransmitter transporter, creatine), member 8 | 102857 | ENSMUSG00000019558 |
| 112 | 17536496 | NA | Tmem28 | transmembrane protein 28 | 620592 | ENSMUSG00000071719 |
| 113 | 17407124 | NA | Chrnb2 | cholinergic receptor, nicotinic, beta polypeptide 2 (neuronal) | 11444 | ENSMUSG00000027950 |
| 114 | 17306666 | NA | Jph4 | junctophilin 4 | 319984 | ENSMUSG00000022208 |
| 115 | 17425095 | NA | Gabbr2 | gamma-aminobutyric acid (GABA) B receptor, 2 | 242425 | ENSMUSG00000039809 |
| 116 | 17500005 | NA | Ank1 | ankyrin 1, erythroid | 11733 | ENSMUSG00000031543 |
| 117 | 17361454 | NA | Cnih2 | cornichon homolog 2 (Drosophila) | 12794 | ENSMUSG00000024873 |
| 118 | 17335145 | NA | Pacsin1 | protein kinase C and casein kinase substrate in neurons 1 | 23969 | ENSMUSG00000040276 |
| 119 | 17258287 | NA | 2310067B10Rik | RIKEN cDNA 2310067B10 gene | 71947 | ENSMUSG00000020747 |
| 120 | 17243229 | NA | Atcay | ataxia, cerebellar, Cayman type homolog (human) | 16467 | ENSMUSG00000034958 |
| 121 | 17357486 | NA | Syt7 | synaptotagmin VII | 54525 | ENSMUSG00000024743 |
| 122 | 17498607 | NA | Mcoln1 | mucolipin 1 | 94178 | ENSMUSG00000004567 |
| 123 | 17295987 | NA | Rgs7bp | regulator of G-protein signalling 7 binding protein | 52882 | ENSMUSG00000021719 |
| 124 | 17447404 | NA | Sorcs2 | sortilin-related VPS10 domain containing receptor 2 | 81840 | ENSMUSG00000029093 |
| 125 | 17249811 | NA | Slc36a1 | solute carrier family 36 (proton/amino acid symporter), member 1 | 215335 | ENSMUSG00000020261 |
| 126 | 17422138 | NA | Gpr153 | G protein-coupled receptor 153 | 100129 | ENSMUSG00000042804 |
| 127 | 17503333 | NA | Tnpo2 | transportin 2 (importin 3, karyopherin beta 2b) | 212999 | ENSMUSG00000031691 |
| 128 | 17317904 | NA | Slc45a4 | solute carrier family 45, member 4 | 106068 | ENSMUSG00000079020 |
| 129 | 17503884 | NA | Gnao1 | guanine nucleotide binding protein, alpha O | 14681 | ENSMUSG00000031748 |
| 130 | 17383858 | NA | Dnm1 | dynamin 1 | 13429 | ENSMUSG00000026825 |
| 131 | 17256579 | NA | Cntnap1 | contactin associated protein-like 1 | 53321 | ENSMUSG00000017167 |
| 132 | 17504399 | NA | Cdh5 | cadherin 5 | 12562 | ENSMUSG00000031871 |
| 133 | 17542695 | NA | G6pdx | glucose-6-phosphate dehydrogenase X-linked | 14381 | ENSMUSG00000031400 |
| 134 | 17477468 | NA | Lrrc4b | leucine rich repeat containing 4B | 272381 | ENSMUSG00000047085 |
| 135 | 17243113 | NA | Lingo3 | leucine rich repeat and Ig domain containing 3 | 237403 | ENSMUSG00000051067 |
| 136 | 17497904 | NA | Slc25a22 | solute carrier family 25 (mitochondrial carrier, glutamate), member 22 | 68267 | ENSMUSG00000019082 |
| 137 | 17369126 | NA | Lrrc8a | leucine rich repeat containing 8A | 241296 | ENSMUSG00000007476 |
| 138 | 17401650 | NA | Amigo1 | adhesion molecule with Ig like domain 1 | 229715 | ENSMUSG00000050947 |
| 139 | 17529930 | NA | Clstn2 | calsyntenin 2 | 64085 | ENSMUSG00000032452 |
| 140 | 17516837 | NA | Bace1 | beta-site APP cleaving enzyme 1 | 23821 | ENSMUSG00000032086 |
| 141 | 17548746 | NA | Bace1 | beta-site APP cleaving enzyme 1 | 23821 | ENSMUSG00000032086 |
| 142 | 17356924 | NA | Nrxn2 | neurexin II | 18190 | ENSMUSG00000033768 |
| 143 | 17264282 | NA | Shisa6 | shisa homolog 6 (Xenopus laevis) | 380702 | ENSMUSG00000053930 |
| 144 | 17234936 | NA | Hcn2 | hyperpolarization-activated, cyclic nucleotide-gated K+ 2 | 15166 | ENSMUSG00000020331 |
| 145 | 17313504 | NA | Srebf2 | sterol regulatory element binding factor 2 | 20788 | ENSMUSG00000022463 |
| 146 | 17259810 | NA | Inpp5j | inositol polyphosphate 5-phosphatase J | 170835 | ENSMUSG00000034570 |
| 147 | 17515843 | NA | Kirrel3 | kin of IRRE like 3 (Drosophila) | 67703 | ENSMUSG00000032036 |
| 148 | 17373521 | NA | Chst1 | carbohydrate (keratan sulfate Gal-6) sulfotransferase 1 | 76969 | ENSMUSG00000027221 |
| 149 | 17454995 | NA | Tmem130 | transmembrane protein 130 | 243339 | ENSMUSG00000043388 |
| 150 | 17419097 | NA | Bai2 | brain-specific angiogenesis inhibitor 2 | 230775 | ENSMUSG00000028782 |
| 151 | 17516462 | NA | Thy1 | thymus cell antigen 1, theta | 21838 | ENSMUSG00000032011 |
| 152 | 17314051 | NA | Panx2 | pannexin 2 | 406218 | ENSMUSG00000058441 |
| 153 | 17461923 | NA | Syn2 | synapsin II | 20965 | ENSMUSG00000009394 |
| 154 | 17342719 | NA | Grm4 | glutamate receptor, metabotropic 4 | 268934 | ENSMUSG00000063239 |
| 155 | 17436545 | NA | Nat8l | N-acetyltransferase 8-like | 269642 | ENSMUSG00000048142 |
| 156 | 17388406 | NA | Slc35c1 | solute carrier family 35, member C1 | 228368 | ENSMUSG00000049922 |
| 157 | 17529218 | NA | Htr1b | 5-hydroxytryptamine (serotonin) receptor 1B | 15551 | ENSMUSG00000049511 |
| 158 | 17284065 | NA | Cdc42bpb | CDC42 binding protein kinase beta | 217866 | ENSMUSG00000021279 |
| 159 | 17238605 | NA | Itga7 | integrin alpha 7 | 16404 | ENSMUSG00000025348 |
| 160 | 17354629 | NA | Ndst1 | N-deacetylase/N-sulfotransferase (heparan glucosaminyl) 1 | 15531 | ENSMUSG00000054008 |
| 161 | 17503122 | NA | Cacna1a | calcium channel, voltage-dependent, P/Q type, alpha 1A subunit | 12286 | ENSMUSG00000034656 |
| 162 | 17328870 | NA | Zdhhc8 | zinc finger, DHHC domain containing 8 | 27801 | ENSMUSG00000060166 |
| 163 | 17400521 | NA | Sv2a | synaptic vesicle glycoprotein 2 a | 64051 | ENSMUSG00000038486 |
| 164 | 17308939 | NA | Pcdh8 | protocadherin 8 | 18530 | ENSMUSG00000036422 |
| 165 | 17233306 | NA | Slc35f1 | solute carrier family 35, member F1 | 215085 | ENSMUSG00000038602 |
| 166 | 17319045 | NA | Elfn2 | leucine rich repeat and fibronectin type III, extracellular 2 | 207393 | ENSMUSG00000043460 |
| 167 | 17527027 | NA | AI593442 | expressed sequence AI593442 | 330941 | ENSMUSG00000078307 |
| 168 | 17302747 | NA | Hs6st3 | heparan sulfate 6-O-sulfotransferase 3 | 50787 | ENSMUSG00000053465 |
| 169 | 17303897 | NA | Ndst2 | N-deacetylase/N-sulfotransferase (heparan glucosaminyl) 2 | 17423 | ENSMUSG00000039308 |
| 170 | 17540059 | NA | Porcn | porcupine homolog (Drosophila) | 53627 | ENSMUSG00000031169 |
| 171 | 17308413 | NA | Epb4.9 | erythrocyte protein band 4.9 | 13829 | ENSMUSG00000022099 |
| 172 | 17263011 | NA | Glra1 | glycine receptor, alpha 1 subunit | 14654 | ENSMUSG00000000263 |
| 173 | 17253376 | NA | Sez6 | seizure related gene 6 | 20370 | ENSMUSG00000000632 |
| 174 | 17246163 | NA | Esyt1 | extended synaptotagmin-like protein 1 | 23943 | ENSMUSG00000025366 |
| 175 | 17327765 | NA | Coro7 | coronin 7 | 78885 | ENSMUSG00000039637 |
| 176 | 17258683 | NA | Mgat5b | mannoside acetylglucosaminyltransferase 5, isoenzyme B | 268510 | ENSMUSG00000043857 |
| 177 | 17517073 | NA | Drd2 | dopamine receptor D2 | 13489 | ENSMUSG00000032259 |
| 178 | 17488292 | NA | Pld3 | phospholipase D family, member 3 | 18807 | ENSMUSG00000003363 |
| 179 | 17542419 | NA | L1cam | L1 cell adhesion molecule | 16728 | ENSMUSG00000031391 |
| 180 | 17321582 | NA | Faim2 | Fas apoptotic inhibitory molecule 2 | 72393 | ENSMUSG00000023011 |
| 181 | 17485943 | NA | Shisa7 | shisa homolog 7 (Xenopus laevis) | 232813 | ENSMUSG00000053550 |
| 182 | 17536720 | NA | Nlgn3 | neuroligin 3 | 245537 | ENSMUSG00000031302 |
| 183 | 17478181 | NA | Kcnc1 | potassium voltage gated channel, Shaw-related subfamily, member 1 | 16502 | ENSMUSG00000058975 |
| 184 | 17271158 | NA | Cacng5 | calcium channel, voltage-dependent, gamma subunit 5 | 140723 | ENSMUSG00000040373 |
| 185 | 17332495 | NA | Dscam | Down syndrome cell adhesion molecule | 13508 | ENSMUSG00000050272 |
| 186 | 17406925 | NA | Hcn3 | hyperpolarization-activated, cyclic nucleotide-gated K+ 3 | 15168 | ENSMUSG00000028051 |
| 187 | 17435570 | NA | Htr5a | 5-hydroxytryptamine (serotonin) receptor 5A | 15563 | ENSMUSG00000039106 |
| 188 | 17473161 | NA | Cacng8 | calcium channel, voltage-dependent, gamma subunit 8 | 81905 | ENSMUSG00000053395 |
| 189 | 17465332 | NA | Lrrc4 | leucine rich repeat containing 4 | 192198 | ENSMUSG00000049939 |
| 190 | 17254071 | NA | Tmem132e | transmembrane protein 132E | 270893 | ENSMUSG00000020701 |
| 191 | 17539019 | NA | Ubqln2 | ubiquilin 2 | 54609 | ENSMUSG00000050148 |
| 192 | 17496763 | NA | Stx1b | syntaxin 1B | 56216 | ENSMUSG00000030806 |
| 193 | 17378450 | NA | Mmp24 | matrix metallopeptidase 24 | 17391 | ENSMUSG00000027612 |
| 194 | 17440732 | NA | Wscd2 | WSC domain containing 2 | 320916 | ENSMUSG00000063430 |
| 195 | 17259344 | NA | Hgs | HGF-regulated tyrosine kinase substrate | 15239 | ENSMUSG00000025793 |
| 196 | 17441453 | NA | Nos1 | nitric oxide synthase 1, neuronal | 18125 | ENSMUSG00000029361 |
| 197 | 17257197 | NA | Mapt | microtubule-associated protein tau | 17762 | ENSMUSG00000018411 |
| 198 | 17300484 | NA | Cpne6 | copine VI | 12891 | ENSMUSG00000022212 |
| 199 | 17409005 | NA | Slc6a17 | solute carrier family 6 (neurotransmitter transporter), member 17 | 229706 | ENSMUSG00000027894 |
| 200 | 17514170 | NA | Pcnxl2 | pecanex-like 2 (Drosophila) | 270109 | ENSMUSG00000060212 |
| 201 | 17235300 | NA | Apc2 | adenomatosis polyposis coli 2 | 23805 | ENSMUSG00000020135 |
| 202 | 17494610 | NA | Dchs1 | dachsous 1 (Drosophila) | 233651 | ENSMUSG00000036862 |
| 203 | 17454574 | NA | Ttyh3 | tweety homolog 3 (Drosophila) | 78339 | ENSMUSG00000036565 |
| 204 | 17314636 | NA | Cacnb3 | calcium channel, voltage-dependent, beta 3 subunit | 12297 | ENSMUSG00000003352 |
| 205 | 17237336 | NA | Kcnc2 | potassium voltage gated channel, Shaw-related subfamily, member 2 | 268345 | ENSMUSG00000035681 |
| 206 | 17420316 | NA | Rap1gap | Rap1 GTPase-activating protein | 110351 | ENSMUSG00000041351 |
| 207 | 17394079 | NA | Rims4 | regulating synaptic membrane exocytosis 4 | 241770 | ENSMUSG00000035226 |
| 208 | 17273714 | NA | Adcy3 | adenylate cyclase 3 | 104111 | ENSMUSG00000020654 |
| 209 | 17243157 | NA | Diras1 | DIRAS family, GTP-binding RAS-like 1 | 208666 | ENSMUSG00000043670 |
| 210 | 17384021 | NA | Stxbp1 | syntaxin binding protein 1 | 20910 | ENSMUSG00000026797 |
| 211 | 17308299 | NA | Slc39a14 | solute carrier family 39 (zinc transporter), member 14 | 213053 | ENSMUSG00000022094 |
| 212 | 17491285 | NA | Ptpn5 | protein tyrosine phosphatase, non-receptor type 5 | 19259 | ENSMUSG00000030854 |
| 213 | 17432299 | NA | Dnajc16 | DnaJ (Hsp40) homolog, subfamily C, member 16 | 214063 | ENSMUSG00000040697 |
| 214 | 17369147 | NA | Nup188 | nucleoporin 188 | 227699 | ENSMUSG00000052533 |
| 215 | 17535627 | NA | Abcd1 | ATP-binding cassette, sub-family D (ALD), member 1 | 11666 | ENSMUSG00000031378 |
| 216 | 17530733 | NA | Grm2 | glutamate receptor, metabotropic 2 | 108068 | ENSMUSG00000023192 |
| 217 | 17237937 | NA | B4galnt1 | beta-1,4-N-acetyl-galactosaminyl transferase 1 | 14421 | ENSMUSG00000006731 |
| 218 | 17474067 | NA | Slc8a2 | solute carrier family 8 (sodium/calcium exchanger), member 2 | 110891 | ENSMUSG00000030376 |
| 219 | 17465620 | NA | Podxl | podocalyxin-like | 27205 | ENSMUSG00000025608 |
| 220 | 17474157 | NA | Strn4 | striatin, calmodulin binding protein 4 | 97387 | ENSMUSG00000030374 |
| 221 | 17504293 | NA | Mmp15 | matrix metallopeptidase 15 | 17388 | ENSMUSG00000031790 |
| 222 | 17357959 | NA | Gnaq | guanine nucleotide binding protein, alpha q polypeptide | 14682 | ENSMUSG00000024639 |
| 223 | 17377144 | NA | Slc24a3 | solute carrier family 24 (sodium/potassium/calcium exchanger), member 3 | 94249 | ENSMUSG00000063873 |
| 224 | 17513806 | NA | Cbfa2t3 | core-binding factor, runt domain, alpha subunit 2, translocated to, 3 (human) | 12398 | ENSMUSG00000006362 |
| 225 | 17266452 | NA | Sarm1 | sterile alpha and HEAT/Armadillo motif containing 1 | 237868 | ENSMUSG00000050132 |
| 226 | 17451482 | NA | Svop | SV2 related protein | 68666 | ENSMUSG00000042078 |
| 227 | 17243057 | NA | Ap3d1 | adaptor-related protein complex 3, delta 1 subunit | 11776 | ENSMUSG00000020198 |
| 228 | 17271622 | NA | Cdc42ep4 | CDC42 effector protein (Rho GTPase binding) 4 | 56699 | ENSMUSG00000041598 |
| 229 | 17419587 | NA | Slc9a1 | solute carrier family 9 (sodium/hydrogen exchanger), member 1 | 20544 | ENSMUSG00000028854 |
| 230 | 17361779 | NA | Scyl1 | SCY1-like 1 (S. cerevisiae) | 78891 | ENSMUSG00000024941 |
| 231 | 17370883 | NA | Kcnj3 | potassium inwardly-rectifying channel, subfamily J, member 3 | 16519 | ENSMUSG00000026824 |
| 232 | 17211790 | NA | Hs6st1 | heparan sulfate 6-O-sulfotransferase 1 | 50785 | ENSMUSG00000045216 |
| 233 | 17346155 | NA | Sema6b | sema domain, transmembrane domain (TM), and cytoplasmic domain, (semaphorin) 6B | 20359 | ENSMUSG00000001227 |
| 234 | 17383798 | NA | Fibcd1 | fibrinogen C domain containing 1 | 98970 | ENSMUSG00000026841 |
| 235 | 17496452 | NA | Taok2 | TAO kinase 2 | 381921 | ENSMUSG00000059981 |
| 236 | 17346185 | NA | Dpp9 | dipeptidylpeptidase 9 | 224897 | ENSMUSG00000001229 |
| 237 | 17235663 | NA | Pip5k1c | phosphatidylinositol-4-phosphate 5-kinase, type 1 gamma | 18717 | ENSMUSG00000034902 |
| 238 | 17378721 | NA | Src | Rous sarcoma oncogene | 20779 | ENSMUSG00000027646 |
| 239 | 17452719 | NA | Abcb9 | ATP-binding cassette, sub-family B (MDR/TAP), member 9 | 56325 | ENSMUSG00000029408 |
| 240 | 17336660 | NA | Atf6b | activating transcription factor 6 beta | 12915 | ENSMUSG00000015461 |
| 241 | 17500301 | NA | Gpr124 | G protein-coupled receptor 124 | 78560 | ENSMUSG00000031486 |
| 242 | 17471155 | NA | Kcna1 | potassium voltage-gated channel, shaker-related subfamily, member 1 | 16485 | ENSMUSG00000047976 |
| 243 | 17450059 | NA | Sec31a | Sec31 homolog A (S. cerevisiae) | 69162 | ENSMUSG00000035325 |
| 244 | 17461868 | NA | Slc6a1 | solute carrier family 6 (neurotransmitter transporter, GABA), member 1 | 232333 | ENSMUSG00000030310 |
| 245 | 17251303 | NA | Pik3r5 | phosphoinositide-3-kinase, regulatory subunit 5, p101 | 320207 | ENSMUSG00000020901 |
| 246 | 17415863 | NA | Cachd1 | cache domain containing 1 | 320508 | ENSMUSG00000028532 |
| 247 | 17334846 | NA | Tmem8 | transmembrane protein 8 (five membrane-spanning domains) | 60455 | ENSMUSG00000024180 |
| 248 | 17325206 | NA | Adcy5 | adenylate cyclase 5 | 224129 | ENSMUSG00000022840 |
| 249 | 17253175 | NA | Tusc5 | tumor suppressor candidate 5 | 237858 | ENSMUSG00000046275 |
| 250 | 17222925 | NA | Mfsd6 | major facilitator superfamily domain containing 6 | 98682 | ENSMUSG00000041439 |
| 251 | 17219519 | NA | Igsf8 | immunoglobulin superfamily, member 8 | 140559 | ENSMUSG00000038034 |
| 252 | 17426402 | NA | Astn2 | astrotactin 2 | 56079 | ENSMUSG00000028373 |
| 253 | 17359583 | NA | Cnnm1 | cyclin M1 | 83674 | ENSMUSG00000025189 |
| 254 | 17433287 | NA | Slc45a1 | solute carrier family 45, member 1 | 242773 | ENSMUSG00000039838 |
| 255 | 17516731 | NA | Scn2b | sodium channel, voltage-gated, type II, beta | 72821 | ENSMUSG00000070304 |
| 256 | 17533640 | NA | Cdk16 | cyclin-dependent kinase 16 | 18555 | ENSMUSG00000031065 |
| 257 | 17360440 | NA | Adra2a | adrenergic receptor, alpha 2a | 11551 | ENSMUSG00000033717 |
| 258 | 17485574 | NA | Mboat7 | membrane bound O-acyltransferase domain containing 7 | 77582 | ENSMUSG00000035596 |
| 259 | 17260261 | NA | Camk2b | calcium/calmodulin-dependent protein kinase II, beta | 12323 | ENSMUSG00000057897 |
| 260 | 17389647 | NA | Rasgrp1 | RAS guanyl releasing protein 1 | 19419 | ENSMUSG00000027347 |

  
  

| **Database:cellular component      &nbspName:axon      &nbspID:GO:0030424** | | | | | | |
| --- | --- | --- | --- | --- | --- | --- |
| C=322; O=34; E=7.29; R=4.66; rawP=9.12e-14; adjP=2.51e-12 | | | | | | |
| Index | UserID | Value | Gene Symbol | Gene Name | EntrezGene | Ensembl |
| 1 | 17378922 | NA | Slc32a1 | solute carrier family 32 (GABA vesicular transporter), member 1 | 22348 | ENSMUSG00000037771 |
| 2 | 17368550 | NA | Rxra | retinoid X receptor alpha | 20181 | ENSMUSG00000015846 |
| 3 | 17342719 | NA | Grm4 | glutamate receptor, metabotropic 4 | 268934 | ENSMUSG00000063239 |
| 4 | 17337513 | NA | Gabbr1 | gamma-aminobutyric acid (GABA) B receptor, 1 | 54393 | ENSMUSG00000024462 |
| 5 | 17530733 | NA | Grm2 | glutamate receptor, metabotropic 2 | 108068 | ENSMUSG00000023192 |
| 6 | 17342065 | NA | Mapk8ip3 | mitogen-activated protein kinase 8 interacting protein 3 | 30957 | ENSMUSG00000024163 |
| 7 | 17382496 | NA | Grin1 | glutamate receptor, ionotropic, NMDA1 (zeta 1) | 14810 | ENSMUSG00000026959 |
| 8 | 17367921 | NA | Grin1 | glutamate receptor, ionotropic, NMDA1 (zeta 1) | 14810 | ENSMUSG00000026959 |
| 9 | 17226891 | NA | Cntn2 | contactin 2 | 21367 | ENSMUSG00000053024 |
| 10 | 17279640 | NA | Ptprn2 | protein tyrosine phosphatase, receptor type, N polypeptide 2 | 19276 | ENSMUSG00000056553 |
| 11 | 17243057 | NA | Ap3d1 | adaptor-related protein complex 3, delta 1 subunit | 11776 | ENSMUSG00000020198 |
| 12 | 17339313 | NA | Epb4.1l3 | erythrocyte protein band 4.1-like 3 | 13823 | ENSMUSG00000024044 |
| 13 | 17517073 | NA | Drd2 | dopamine receptor D2 | 13489 | ENSMUSG00000032259 |
| 14 | 17361223 | NA | Adrbk1 | adrenergic receptor kinase, beta 1 | 110355 | ENSMUSG00000024858 |
| 15 | 17542419 | NA | L1cam | L1 cell adhesion molecule | 16728 | ENSMUSG00000031391 |
| 16 | 17354299 | NA | Sema6a | sema domain, transmembrane domain (TM), and cytoplasmic domain, (semaphorin) 6A | 20358 | ENSMUSG00000019647 |
| 17 | 17487805 | NA | Atp1a3 | ATPase, Na+/K+ transporting, alpha 3 polypeptide | 232975 | ENSMUSG00000040907 |
| 18 | 17478181 | NA | Kcnc1 | potassium voltage gated channel, Shaw-related subfamily, member 1 | 16502 | ENSMUSG00000058975 |
| 19 | 17471155 | NA | Kcna1 | potassium voltage-gated channel, shaker-related subfamily, member 1 | 16485 | ENSMUSG00000047976 |
| 20 | 17332495 | NA | Dscam | Down syndrome cell adhesion molecule | 13508 | ENSMUSG00000050272 |
| 21 | 17461868 | NA | Slc6a1 | solute carrier family 6 (neurotransmitter transporter, GABA), member 1 | 232333 | ENSMUSG00000030310 |
| 22 | 17290259 | NA | Ucn3 | urocortin 3 | 83428 | ENSMUSG00000044988 |
| 23 | 17307905 | NA | Dpysl2 | dihydropyrimidinase-like 2 | 12934 | ENSMUSG00000022048 |
| 24 | 17453430 | NA | Limk1 | LIM-domain containing, protein kinase | 16885 | ENSMUSG00000029674 |
| 25 | 17500005 | NA | Ank1 | ankyrin 1, erythroid | 11733 | ENSMUSG00000031543 |
| 26 | 17239234 | NA | Grm1 | glutamate receptor, metabotropic 1 | 14816 | ENSMUSG00000019828 |
| 27 | 17430140 | NA | Ncdn | neurochondrin | 26562 | ENSMUSG00000028833 |
| 28 | 17257197 | NA | Mapt | microtubule-associated protein tau | 17762 | ENSMUSG00000018411 |
| 29 | 17300484 | NA | Cpne6 | copine VI | 12891 | ENSMUSG00000022212 |
| 30 | 17256579 | NA | Cntnap1 | contactin associated protein-like 1 | 53321 | ENSMUSG00000017167 |
| 31 | 17293045 | NA | Spock1 | sparc/osteonectin, cwcv and kazal-like domains proteoglycan 1 | 20745 | ENSMUSG00000056222 |
| 32 | 17269439 | NA | Hap1 | huntingtin-associated protein 1 | 15114 | ENSMUSG00000006930 |
| 33 | 17516837 | NA | Bace1 | beta-site APP cleaving enzyme 1 | 23821 | ENSMUSG00000032086 |
| 34 | 17548746 | NA | Bace1 | beta-site APP cleaving enzyme 1 | 23821 | ENSMUSG00000032086 |
| 35 | 17234936 | NA | Hcn2 | hyperpolarization-activated, cyclic nucleotide-gated K+ 2 | 15166 | ENSMUSG00000020331 |
| 36 | 17515843 | NA | Kirrel3 | kin of IRRE like 3 (Drosophila) | 67703 | ENSMUSG00000032036 |

  
  

| **Database:cellular component      &nbspName:membrane part      &nbspID:GO:0044425** | | | | | | |
| --- | --- | --- | --- | --- | --- | --- |
| C=6262; O=214; E=141.74; R=1.51; rawP=7.94e-13; adjP=1.97e-11 | | | | | | |
| Index | UserID | Value | Gene Symbol | Gene Name | EntrezGene | Ensembl |
| 1 | 17318923 | NA | Cacng2 | calcium channel, voltage-dependent, gamma subunit 2 | 12300 | ENSMUSG00000019146 |
| 2 | 17378922 | NA | Slc32a1 | solute carrier family 32 (GABA vesicular transporter), member 1 | 22348 | ENSMUSG00000037771 |
| 3 | 17242822 | NA | Dos | downstream of Stk11 | 100503659 | ENSMUSG00000035640 |
| 4 | 17521371 | NA | Tmem115 | transmembrane protein 115 | 56395 | ENSMUSG00000010045 |
| 5 | 17272798 | NA | Cant1 | calcium activated nucleotidase 1 | 76025 | ENSMUSG00000025575 |
| 6 | 17334545 | NA | Clcn7 | chloride channel 7 | 26373 | ENSMUSG00000036636 |
| 7 | 17362646 | NA | Dagla | diacylglycerol lipase, alpha | 269060 | ENSMUSG00000035735 |
| 8 | 17461852 | NA | Slc6a11 | solute carrier family 6 (neurotransmitter transporter, GABA), member 11 | 243616 | ENSMUSG00000030307 |
| 9 | 17342065 | NA | Mapk8ip3 | mitogen-activated protein kinase 8 interacting protein 3 | 30957 | ENSMUSG00000024163 |
| 10 | 17515358 | NA | BC018242 | cDNA sequence BC018242 | 235044 | ENSMUSG00000040563 |
| 11 | 17226891 | NA | Cntn2 | contactin 2 | 21367 | ENSMUSG00000053024 |
| 12 | 17252995 | NA | Slc43a2 | solute carrier family 43, member 2 | 215113 | ENSMUSG00000038178 |
| 13 | 17522338 | NA | Scap | SREBF chaperone | 235623 | ENSMUSG00000032485 |
| 14 | 17361703 | NA | Pcnxl3 | pecanex-like 3 (Drosophila) | 104401 | ENSMUSG00000054874 |
| 15 | 17416325 | NA | Dhcr24 | 24-dehydrocholesterol reductase | 74754 | ENSMUSG00000034926 |
| 16 | 17336636 | NA | Agpat1 | 1-acylglycerol-3-phosphate O-acyltransferase 1 (lysophosphatidic acid acyltransferase, alpha) | 55979 | ENSMUSG00000034254 |
| 17 | 17339313 | NA | Epb4.1l3 | erythrocyte protein band 4.1-like 3 | 13823 | ENSMUSG00000024044 |
| 18 | 17424279 | NA | Cntfr | ciliary neurotrophic factor receptor | 12804 | ENSMUSG00000028444 |
| 19 | 17351262 | NA | St8sia3 | ST8 alpha-N-acetyl-neuraminide alpha-2,8-sialyltransferase 3 | 20451 | ENSMUSG00000056812 |
| 20 | 17361223 | NA | Adrbk1 | adrenergic receptor kinase, beta 1 | 110355 | ENSMUSG00000024858 |
| 21 | 17469814 | NA | Atp2b2 | ATPase, Ca++ transporting, plasma membrane 2 | 11941 | ENSMUSG00000030302 |
| 22 | 17401394 | NA | Kcnd3 | potassium voltage-gated channel, Shal-related family, member 3 | 56543 | ENSMUSG00000040896 |
| 23 | 17346231 | NA | Ptprs | protein tyrosine phosphatase, receptor type, S | 19280 | ENSMUSG00000013236 |
| 24 | 17487805 | NA | Atp1a3 | ATPase, Na+/K+ transporting, alpha 3 polypeptide | 232975 | ENSMUSG00000040907 |
| 25 | 17328958 | NA | Gp1bb | glycoprotein Ib, beta polypeptide | 14724 | ENSMUSG00000050761 |
| 26 | 17453222 | NA | Wbscr17 | Williams-Beuren syndrome chromosome region 17 homolog (human) | 212996 | ENSMUSG00000034040 |
| 27 | 17242707 | NA | BC005764 | cDNA sequence BC005764 | 216152 | ENSMUSG00000035835 |
| 28 | 17526206 | NA | C2cd2l | C2 calcium-dependent domain containing 2-like | 71764 | ENSMUSG00000032120 |
| 29 | 17239234 | NA | Grm1 | glutamate receptor, metabotropic 1 | 14816 | ENSMUSG00000019828 |
| 30 | 17409343 | NA | 5330417C22Rik | RIKEN cDNA 5330417C22 gene | 229722 | ENSMUSG00000040412 |
| 31 | 17224146 | NA | March4 | membrane-associated ring finger (C3HC4) 4 | 381270 | ENSMUSG00000039372 |
| 32 | 17236102 | NA | Btbd11 | BTB (POZ) domain containing 11 | 74007 | ENSMUSG00000020042 |
| 33 | 17440538 | NA | Galnt9 | UDP-N-acetyl-alpha-D-galactosamine:polypeptide N-acetylgalactosaminyltransferase 9 | 231605 | ENSMUSG00000033316 |
| 34 | 17345262 | NA | Tmem63b | transmembrane protein 63b | 224807 | ENSMUSG00000036026 |
| 35 | 17298874 | NA | Grid1 | glutamate receptor, ionotropic, delta 1 | 14803 | ENSMUSG00000041078 |
| 36 | 17349607 | NA | Psd2 | pleckstrin and Sec7 domain containing 2 | 74002 | ENSMUSG00000024347 |
| 37 | 17413436 | NA | Tmem8b | transmembrane protein 8B | 242409 | ENSMUSG00000078716 |
| 38 | 17521327 | NA | Cacna2d2 | calcium channel, voltage-dependent, alpha 2/delta subunit 2 | 56808 | ENSMUSG00000010066 |
| 39 | 17473155 | NA | Cacng7 | calcium channel, voltage-dependent, gamma subunit 7 | 81904 | ENSMUSG00000069806 |
| 40 | 17431720 | NA | Alpl | alkaline phosphatase, liver/bone/kidney | 11647 | ENSMUSG00000028766 |
| 41 | 17501692 | NA | Atp13a1 | ATPase type 13A1 | 170759 | ENSMUSG00000031862 |
| 42 | 17477714 | NA | Slc17a7 | solute carrier family 17 (sodium-dependent inorganic phosphate cotransporter), member 7 | 72961 | ENSMUSG00000070570 |
| 43 | 17525075 | NA | Herpud2 | HERPUD family member 2 | 80517 | ENSMUSG00000008429 |
| 44 | 17319339 | NA | Npcd | neuronal pentraxin chromo domain | 504193 | ENSMUSG00000089837 ENSMUSG00000022421 |
| 45 | 17243162 | NA | Slc39a3 | solute carrier family 39 (zinc transporter), member 3 | 106947 | ENSMUSG00000046822 |
| 46 | 17317801 | NA | Kcnk9 | potassium channel, subfamily K, member 9 | 223604 | ENSMUSG00000036760 |
| 47 | 17457465 | NA | Clec2l | C-type lectin domain family, member L | 665180 | ENSMUSG00000079598 |
| 48 | 17527520 | NA | Scamp5 | secretory carrier membrane protein 5 | 56807 | ENSMUSG00000040722 |
| 49 | 17532879 | NA | Syp | synaptophysin | 20977 | ENSMUSG00000031144 |
| 50 | 17483194 | NA | Sez6l2 | seizure related 6 homolog like 2 | 233878 | ENSMUSG00000030683 |
| 51 | 17518342 | NA | Megf11 | multiple EGF-like-domains 11 | 214058 | ENSMUSG00000036466 |
| 52 | 17215820 | NA | Gpc1 | glypican 1 | 14733 | ENSMUSG00000034220 |
| 53 | 17397377 | NA | Pcdh10 | protocadherin 10 | 18526 | ENSMUSG00000049100 |
| 54 | 17302289 | NA | Pcdh17 | protocadherin 17 | 219228 | ENSMUSG00000035566 |
| 55 | 17490452 | NA | Ap2a1 | adaptor protein complex AP-2, alpha 1 subunit | 11771 | ENSMUSG00000060279 |
| 56 | 17312341 | NA | Grina | glutamate receptor, ionotropic, N-methyl D-aspartate-associated protein 1 (glutamate binding) | 66168 | ENSMUSG00000022564 |
| 57 | 17373530 | NA | Syt13 | synaptotagmin XIII | 80976 | ENSMUSG00000027220 |
| 58 | 17484409 | NA | Gpr123 | G protein-coupled receptor 123 | 52389 | ENSMUSG00000025475 |
| 59 | 17313106 | NA | Cacna1i | calcium channel, voltage-dependent, alpha 1I subunit | 239556 | ENSMUSG00000022416 |
| 60 | 17417702 | NA | Slc6a9 | solute carrier family 6 (neurotransmitter transporter, glycine), member 9 | 14664 | ENSMUSG00000028542 |
| 61 | 17477454 | NA | Syt3 | synaptotagmin III | 20981 | ENSMUSG00000030731 |
| 62 | 17545450 | NA | Gpr173 | G-protein coupled receptor 173 | 70771 | ENSMUSG00000056679 |
| 63 | 17452139 | NA | Rph3a | rabphilin 3A | 19894 | ENSMUSG00000029608 |
| 64 | 17337513 | NA | Gabbr1 | gamma-aminobutyric acid (GABA) B receptor, 1 | 54393 | ENSMUSG00000024462 |
| 65 | 17541383 | NA | Zdhhc9 | zinc finger, DHHC domain containing 9 | 208884 | ENSMUSG00000036985 |
| 66 | 17235037 | NA | Arid3a | AT rich interactive domain 3A (BRIGHT-like) | 13496 | ENSMUSG00000019564 |
| 67 | 17302600 | NA | Slitrk5 | SLIT and NTRK-like family, member 5 | 75409 | ENSMUSG00000033214 |
| 68 | 17489052 | NA | Aplp1 | amyloid beta (A4) precursor-like protein 1 | 11803 | ENSMUSG00000006651 |
| 69 | 17432808 | NA | Mfn2 | mitofusin 2 | 170731 | ENSMUSG00000029020 |
| 70 | 17504130 | NA | Cx3cl1 | chemokine (C-X3-C motif) ligand 1 | 20312 | ENSMUSG00000031778 |
| 71 | 17382496 | NA | Grin1 | glutamate receptor, ionotropic, NMDA1 (zeta 1) | 14810 | ENSMUSG00000026959 |
| 72 | 17367921 | NA | Grin1 | glutamate receptor, ionotropic, NMDA1 (zeta 1) | 14810 | ENSMUSG00000026959 |
| 73 | 17475851 | NA | Lrfn1 | leucine rich repeat and fibronectin type III domain containing 1 | 80749 | NULL |
| 74 | 17279640 | NA | Ptprn2 | protein tyrosine phosphatase, receptor type, N polypeptide 2 | 19276 | ENSMUSG00000056553 |
| 75 | 17214665 | NA | Sgpp2 | sphingosine-1-phosphate phosphotase 2 | 433323 | ENSMUSG00000032908 |
| 76 | 17347948 | NA | Kcnk12 | potassium channel, subfamily K, member 12 | 210741 | ENSMUSG00000050138 |
| 77 | 17334205 | NA | Abca3 | ATP-binding cassette, sub-family A (ABC1), member 3 | 27410 | ENSMUSG00000024130 |
| 78 | 17376167 | NA | Sirpa | signal-regulatory protein alpha | 19261 | ENSMUSG00000037902 |
| 79 | 17446123 | NA | Kcnh2 | potassium voltage-gated channel, subfamily H (eag-related), member 2 | 16511 | ENSMUSG00000038319 |
| 80 | 17269464 | NA | Jup | junction plakoglobin | 16480 | ENSMUSG00000001552 |
| 81 | 17418732 | NA | Dlgap3 | discs, large (Drosophila) homolog-associated protein 3 | 242667 | ENSMUSG00000042388 |
| 82 | 17301823 | NA | Gfra2 | glial cell line derived neurotrophic factor family receptor alpha 2 | 14586 | ENSMUSG00000022103 |
| 83 | 17404570 | NA | Slc7a14 | solute carrier family 7 (cationic amino acid transporter, y+ system), member 14 | 241919 | ENSMUSG00000069072 |
| 84 | 17406760 | NA | Sema4a | sema domain, immunoglobulin domain (Ig), transmembrane domain (TM) and short cytoplasmic domain, (semaphorin) 4A | 20351 | ENSMUSG00000028064 |
| 85 | 17342509 | NA | Pigq | phosphatidylinositol glycan anchor biosynthesis, class Q | 14755 | ENSMUSG00000025728 |
| 86 | 17374618 | NA | Disp2 | dispatched homolog 2 (Drosophila) | 214240 | ENSMUSG00000040035 |
| 87 | 17535572 | NA | Atp2b3 | ATPase, Ca++ transporting, plasma membrane 3 | 320707 | ENSMUSG00000031376 |
| 88 | 17451223 | NA | Sez6l | seizure related 6 homolog like | 56747 | ENSMUSG00000058153 |
| 89 | 17354299 | NA | Sema6a | sema domain, transmembrane domain (TM), and cytoplasmic domain, (semaphorin) 6A | 20358 | ENSMUSG00000019647 |
| 90 | 17435528 | NA | Dpp6 | dipeptidylpeptidase 6 | 13483 | ENSMUSG00000061576 |
| 91 | 17535607 | NA | Slc6a8 | solute carrier family 6 (neurotransmitter transporter, creatine), member 8 | 102857 | ENSMUSG00000019558 |
| 92 | 17536496 | NA | Tmem28 | transmembrane protein 28 | 620592 | ENSMUSG00000071719 |
| 93 | 17407124 | NA | Chrnb2 | cholinergic receptor, nicotinic, beta polypeptide 2 (neuronal) | 11444 | ENSMUSG00000027950 |
| 94 | 17306666 | NA | Jph4 | junctophilin 4 | 319984 | ENSMUSG00000022208 |
| 95 | 17425095 | NA | Gabbr2 | gamma-aminobutyric acid (GABA) B receptor, 2 | 242425 | ENSMUSG00000039809 |
| 96 | 17500005 | NA | Ank1 | ankyrin 1, erythroid | 11733 | ENSMUSG00000031543 |
| 97 | 17361454 | NA | Cnih2 | cornichon homolog 2 (Drosophila) | 12794 | ENSMUSG00000024873 |
| 98 | 17258287 | NA | 2310067B10Rik | RIKEN cDNA 2310067B10 gene | 71947 | ENSMUSG00000020747 |
| 99 | 17357486 | NA | Syt7 | synaptotagmin VII | 54525 | ENSMUSG00000024743 |
| 100 | 17498607 | NA | Mcoln1 | mucolipin 1 | 94178 | ENSMUSG00000004567 |
| 101 | 17447404 | NA | Sorcs2 | sortilin-related VPS10 domain containing receptor 2 | 81840 | ENSMUSG00000029093 |
| 102 | 17249811 | NA | Slc36a1 | solute carrier family 36 (proton/amino acid symporter), member 1 | 215335 | ENSMUSG00000020261 |
| 103 | 17503333 | NA | Tnpo2 | transportin 2 (importin 3, karyopherin beta 2b) | 212999 | ENSMUSG00000031691 |
| 104 | 17422138 | NA | Gpr153 | G protein-coupled receptor 153 | 100129 | ENSMUSG00000042804 |
| 105 | 17317904 | NA | Slc45a4 | solute carrier family 45, member 4 | 106068 | ENSMUSG00000079020 |
| 106 | 17503884 | NA | Gnao1 | guanine nucleotide binding protein, alpha O | 14681 | ENSMUSG00000031748 |
| 107 | 17383858 | NA | Dnm1 | dynamin 1 | 13429 | ENSMUSG00000026825 |
| 108 | 17256579 | NA | Cntnap1 | contactin associated protein-like 1 | 53321 | ENSMUSG00000017167 |
| 109 | 17504399 | NA | Cdh5 | cadherin 5 | 12562 | ENSMUSG00000031871 |
| 110 | 17542695 | NA | G6pdx | glucose-6-phosphate dehydrogenase X-linked | 14381 | ENSMUSG00000031400 |
| 111 | 17477468 | NA | Lrrc4b | leucine rich repeat containing 4B | 272381 | ENSMUSG00000047085 |
| 112 | 17243113 | NA | Lingo3 | leucine rich repeat and Ig domain containing 3 | 237403 | ENSMUSG00000051067 |
| 113 | 17497904 | NA | Slc25a22 | solute carrier family 25 (mitochondrial carrier, glutamate), member 22 | 68267 | ENSMUSG00000019082 |
| 114 | 17369126 | NA | Lrrc8a | leucine rich repeat containing 8A | 241296 | ENSMUSG00000007476 |
| 115 | 17401650 | NA | Amigo1 | adhesion molecule with Ig like domain 1 | 229715 | ENSMUSG00000050947 |
| 116 | 17529930 | NA | Clstn2 | calsyntenin 2 | 64085 | ENSMUSG00000032452 |
| 117 | 17516837 | NA | Bace1 | beta-site APP cleaving enzyme 1 | 23821 | ENSMUSG00000032086 |
| 118 | 17548746 | NA | Bace1 | beta-site APP cleaving enzyme 1 | 23821 | ENSMUSG00000032086 |
| 119 | 17356924 | NA | Nrxn2 | neurexin II | 18190 | ENSMUSG00000033768 |
| 120 | 17264282 | NA | Shisa6 | shisa homolog 6 (Xenopus laevis) | 380702 | ENSMUSG00000053930 |
| 121 | 17234936 | NA | Hcn2 | hyperpolarization-activated, cyclic nucleotide-gated K+ 2 | 15166 | ENSMUSG00000020331 |
| 122 | 17313504 | NA | Srebf2 | sterol regulatory element binding factor 2 | 20788 | ENSMUSG00000022463 |
| 123 | 17259810 | NA | Inpp5j | inositol polyphosphate 5-phosphatase J | 170835 | ENSMUSG00000034570 |
| 124 | 17515843 | NA | Kirrel3 | kin of IRRE like 3 (Drosophila) | 67703 | ENSMUSG00000032036 |
| 125 | 17373521 | NA | Chst1 | carbohydrate (keratan sulfate Gal-6) sulfotransferase 1 | 76969 | ENSMUSG00000027221 |
| 126 | 17454995 | NA | Tmem130 | transmembrane protein 130 | 243339 | ENSMUSG00000043388 |
| 127 | 17419097 | NA | Bai2 | brain-specific angiogenesis inhibitor 2 | 230775 | ENSMUSG00000028782 |
| 128 | 17516462 | NA | Thy1 | thymus cell antigen 1, theta | 21838 | ENSMUSG00000032011 |
| 129 | 17314051 | NA | Panx2 | pannexin 2 | 406218 | ENSMUSG00000058441 |
| 130 | 17342719 | NA | Grm4 | glutamate receptor, metabotropic 4 | 268934 | ENSMUSG00000063239 |
| 131 | 17436545 | NA | Nat8l | N-acetyltransferase 8-like | 269642 | ENSMUSG00000048142 |
| 132 | 17388406 | NA | Slc35c1 | solute carrier family 35, member C1 | 228368 | ENSMUSG00000049922 |
| 133 | 17529218 | NA | Htr1b | 5-hydroxytryptamine (serotonin) receptor 1B | 15551 | ENSMUSG00000049511 |
| 134 | 17238605 | NA | Itga7 | integrin alpha 7 | 16404 | ENSMUSG00000025348 |
| 135 | 17354629 | NA | Ndst1 | N-deacetylase/N-sulfotransferase (heparan glucosaminyl) 1 | 15531 | ENSMUSG00000054008 |
| 136 | 17503122 | NA | Cacna1a | calcium channel, voltage-dependent, P/Q type, alpha 1A subunit | 12286 | ENSMUSG00000034656 |
| 137 | 17328870 | NA | Zdhhc8 | zinc finger, DHHC domain containing 8 | 27801 | ENSMUSG00000060166 |
| 138 | 17400521 | NA | Sv2a | synaptic vesicle glycoprotein 2 a | 64051 | ENSMUSG00000038486 |
| 139 | 17308939 | NA | Pcdh8 | protocadherin 8 | 18530 | ENSMUSG00000036422 |
| 140 | 17233306 | NA | Slc35f1 | solute carrier family 35, member F1 | 215085 | ENSMUSG00000038602 |
| 141 | 17319045 | NA | Elfn2 | leucine rich repeat and fibronectin type III, extracellular 2 | 207393 | ENSMUSG00000043460 |
| 142 | 17527027 | NA | AI593442 | expressed sequence AI593442 | 330941 | ENSMUSG00000078307 |
| 143 | 17302747 | NA | Hs6st3 | heparan sulfate 6-O-sulfotransferase 3 | 50787 | ENSMUSG00000053465 |
| 144 | 17303897 | NA | Ndst2 | N-deacetylase/N-sulfotransferase (heparan glucosaminyl) 2 | 17423 | ENSMUSG00000039308 |
| 145 | 17540059 | NA | Porcn | porcupine homolog (Drosophila) | 53627 | ENSMUSG00000031169 |
| 146 | 17263011 | NA | Glra1 | glycine receptor, alpha 1 subunit | 14654 | ENSMUSG00000000263 |
| 147 | 17253376 | NA | Sez6 | seizure related gene 6 | 20370 | ENSMUSG00000000632 |
| 148 | 17246163 | NA | Esyt1 | extended synaptotagmin-like protein 1 | 23943 | ENSMUSG00000025366 |
| 149 | 17327765 | NA | Coro7 | coronin 7 | 78885 | ENSMUSG00000039637 |
| 150 | 17258683 | NA | Mgat5b | mannoside acetylglucosaminyltransferase 5, isoenzyme B | 268510 | ENSMUSG00000043857 |
| 151 | 17517073 | NA | Drd2 | dopamine receptor D2 | 13489 | ENSMUSG00000032259 |
| 152 | 17488292 | NA | Pld3 | phospholipase D family, member 3 | 18807 | ENSMUSG00000003363 |
| 153 | 17542419 | NA | L1cam | L1 cell adhesion molecule | 16728 | ENSMUSG00000031391 |
| 154 | 17321582 | NA | Faim2 | Fas apoptotic inhibitory molecule 2 | 72393 | ENSMUSG00000023011 |
| 155 | 17485943 | NA | Shisa7 | shisa homolog 7 (Xenopus laevis) | 232813 | ENSMUSG00000053550 |
| 156 | 17536720 | NA | Nlgn3 | neuroligin 3 | 245537 | ENSMUSG00000031302 |
| 157 | 17478181 | NA | Kcnc1 | potassium voltage gated channel, Shaw-related subfamily, member 1 | 16502 | ENSMUSG00000058975 |
| 158 | 17271158 | NA | Cacng5 | calcium channel, voltage-dependent, gamma subunit 5 | 140723 | ENSMUSG00000040373 |
| 159 | 17332495 | NA | Dscam | Down syndrome cell adhesion molecule | 13508 | ENSMUSG00000050272 |
| 160 | 17406925 | NA | Hcn3 | hyperpolarization-activated, cyclic nucleotide-gated K+ 3 | 15168 | ENSMUSG00000028051 |
| 161 | 17435570 | NA | Htr5a | 5-hydroxytryptamine (serotonin) receptor 5A | 15563 | ENSMUSG00000039106 |
| 162 | 17473161 | NA | Cacng8 | calcium channel, voltage-dependent, gamma subunit 8 | 81905 | ENSMUSG00000053395 |
| 163 | 17465332 | NA | Lrrc4 | leucine rich repeat containing 4 | 192198 | ENSMUSG00000049939 |
| 164 | 17254071 | NA | Tmem132e | transmembrane protein 132E | 270893 | ENSMUSG00000020701 |
| 165 | 17496763 | NA | Stx1b | syntaxin 1B | 56216 | ENSMUSG00000030806 |
| 166 | 17378450 | NA | Mmp24 | matrix metallopeptidase 24 | 17391 | ENSMUSG00000027612 |
| 167 | 17440732 | NA | Wscd2 | WSC domain containing 2 | 320916 | ENSMUSG00000063430 |
| 168 | 17409005 | NA | Slc6a17 | solute carrier family 6 (neurotransmitter transporter), member 17 | 229706 | ENSMUSG00000027894 |
| 169 | 17514170 | NA | Pcnxl2 | pecanex-like 2 (Drosophila) | 270109 | ENSMUSG00000060212 |
| 170 | 17235300 | NA | Apc2 | adenomatosis polyposis coli 2 | 23805 | ENSMUSG00000020135 |
| 171 | 17494610 | NA | Dchs1 | dachsous 1 (Drosophila) | 233651 | ENSMUSG00000036862 |
| 172 | 17454574 | NA | Ttyh3 | tweety homolog 3 (Drosophila) | 78339 | ENSMUSG00000036565 |
| 173 | 17314636 | NA | Cacnb3 | calcium channel, voltage-dependent, beta 3 subunit | 12297 | ENSMUSG00000003352 |
| 174 | 17237336 | NA | Kcnc2 | potassium voltage gated channel, Shaw-related subfamily, member 2 | 268345 | ENSMUSG00000035681 |
| 175 | 17273714 | NA | Adcy3 | adenylate cyclase 3 | 104111 | ENSMUSG00000020654 |
| 176 | 17308299 | NA | Slc39a14 | solute carrier family 39 (zinc transporter), member 14 | 213053 | ENSMUSG00000022094 |
| 177 | 17491285 | NA | Ptpn5 | protein tyrosine phosphatase, non-receptor type 5 | 19259 | ENSMUSG00000030854 |
| 178 | 17432299 | NA | Dnajc16 | DnaJ (Hsp40) homolog, subfamily C, member 16 | 214063 | ENSMUSG00000040697 |
| 179 | 17369147 | NA | Nup188 | nucleoporin 188 | 227699 | ENSMUSG00000052533 |
| 180 | 17535627 | NA | Abcd1 | ATP-binding cassette, sub-family D (ALD), member 1 | 11666 | ENSMUSG00000031378 |
| 181 | 17530733 | NA | Grm2 | glutamate receptor, metabotropic 2 | 108068 | ENSMUSG00000023192 |
| 182 | 17237937 | NA | B4galnt1 | beta-1,4-N-acetyl-galactosaminyl transferase 1 | 14421 | ENSMUSG00000006731 |
| 183 | 17474067 | NA | Slc8a2 | solute carrier family 8 (sodium/calcium exchanger), member 2 | 110891 | ENSMUSG00000030376 |
| 184 | 17465620 | NA | Podxl | podocalyxin-like | 27205 | ENSMUSG00000025608 |
| 185 | 17504293 | NA | Mmp15 | matrix metallopeptidase 15 | 17388 | ENSMUSG00000031790 |
| 186 | 17357959 | NA | Gnaq | guanine nucleotide binding protein, alpha q polypeptide | 14682 | ENSMUSG00000024639 |
| 187 | 17377144 | NA | Slc24a3 | solute carrier family 24 (sodium/potassium/calcium exchanger), member 3 | 94249 | ENSMUSG00000063873 |
| 188 | 17266452 | NA | Sarm1 | sterile alpha and HEAT/Armadillo motif containing 1 | 237868 | ENSMUSG00000050132 |
| 189 | 17451482 | NA | Svop | SV2 related protein | 68666 | ENSMUSG00000042078 |
| 190 | 17243057 | NA | Ap3d1 | adaptor-related protein complex 3, delta 1 subunit | 11776 | ENSMUSG00000020198 |
| 191 | 17419587 | NA | Slc9a1 | solute carrier family 9 (sodium/hydrogen exchanger), member 1 | 20544 | ENSMUSG00000028854 |
| 192 | 17361779 | NA | Scyl1 | SCY1-like 1 (S. cerevisiae) | 78891 | ENSMUSG00000024941 |
| 193 | 17370883 | NA | Kcnj3 | potassium inwardly-rectifying channel, subfamily J, member 3 | 16519 | ENSMUSG00000026824 |
| 194 | 17211790 | NA | Hs6st1 | heparan sulfate 6-O-sulfotransferase 1 | 50785 | ENSMUSG00000045216 |
| 195 | 17346155 | NA | Sema6b | sema domain, transmembrane domain (TM), and cytoplasmic domain, (semaphorin) 6B | 20359 | ENSMUSG00000001227 |
| 196 | 17383798 | NA | Fibcd1 | fibrinogen C domain containing 1 | 98970 | ENSMUSG00000026841 |
| 197 | 17496452 | NA | Taok2 | TAO kinase 2 | 381921 | ENSMUSG00000059981 |
| 198 | 17378721 | NA | Src | Rous sarcoma oncogene | 20779 | ENSMUSG00000027646 |
| 199 | 17452719 | NA | Abcb9 | ATP-binding cassette, sub-family B (MDR/TAP), member 9 | 56325 | ENSMUSG00000029408 |
| 200 | 17336660 | NA | Atf6b | activating transcription factor 6 beta | 12915 | ENSMUSG00000015461 |
| 201 | 17500301 | NA | Gpr124 | G protein-coupled receptor 124 | 78560 | ENSMUSG00000031486 |
| 202 | 17471155 | NA | Kcna1 | potassium voltage-gated channel, shaker-related subfamily, member 1 | 16485 | ENSMUSG00000047976 |
| 203 | 17461868 | NA | Slc6a1 | solute carrier family 6 (neurotransmitter transporter, GABA), member 1 | 232333 | ENSMUSG00000030310 |
| 204 | 17415863 | NA | Cachd1 | cache domain containing 1 | 320508 | ENSMUSG00000028532 |
| 205 | 17334846 | NA | Tmem8 | transmembrane protein 8 (five membrane-spanning domains) | 60455 | ENSMUSG00000024180 |
| 206 | 17325206 | NA | Adcy5 | adenylate cyclase 5 | 224129 | ENSMUSG00000022840 |
| 207 | 17253175 | NA | Tusc5 | tumor suppressor candidate 5 | 237858 | ENSMUSG00000046275 |
| 208 | 17222925 | NA | Mfsd6 | major facilitator superfamily domain containing 6 | 98682 | ENSMUSG00000041439 |
| 209 | 17219519 | NA | Igsf8 | immunoglobulin superfamily, member 8 | 140559 | ENSMUSG00000038034 |
| 210 | 17426402 | NA | Astn2 | astrotactin 2 | 56079 | ENSMUSG00000028373 |
| 211 | 17359583 | NA | Cnnm1 | cyclin M1 | 83674 | ENSMUSG00000025189 |
| 212 | 17433287 | NA | Slc45a1 | solute carrier family 45, member 1 | 242773 | ENSMUSG00000039838 |
| 213 | 17516731 | NA | Scn2b | sodium channel, voltage-gated, type II, beta | 72821 | ENSMUSG00000070304 |
| 214 | 17533640 | NA | Cdk16 | cyclin-dependent kinase 16 | 18555 | ENSMUSG00000031065 |
| 215 | 17360440 | NA | Adra2a | adrenergic receptor, alpha 2a | 11551 | ENSMUSG00000033717 |
| 216 | 17485574 | NA | Mboat7 | membrane bound O-acyltransferase domain containing 7 | 77582 | ENSMUSG00000035596 |

  
  
  
  


---

WebGestalt is currently developed and maintained by Jing Wang and Bing Zhang at the  Zhang Lab. Other people who have made significant contribution to the project include Dexter Duncan, Stefan Kirov, Zhiao Shi, and Jay Snoddy.  
  
**Funding credits:** NIH/NIAAA (U01 AA016662, U01 AA013512); NIH/NIDA (P01 DA015027); NIH/NIMH (P50 MH078028, P50 MH096972); NIH/NCI (U24 CA159988); NIH/NIGMS (R01 GM088822).
