# Supplementary material for: Genetic Networks in Mouse Retinal Ganglion Cells
Source: Front Genet. 2016 Sep 28;7:169. doi: 10.3389/fgene.2016.00169 (PMC5039302; doi:10.3389/fgene.2016.00169)
Supplement: Supplementary file 3 [file DataSheet1.ZIP › Tubb3-network GO/files/final_sig_file_1470845142.html]

Anchored HTML File of EIDs


|  |  |
| --- | --- |
|  | WEB-based GEne SeT AnaLysis Toolkit |
|  |  |
|  | ***Translating gene lists into biological insights...*** |
|  |  |

---

  

| **Database:biological process      &nbspName:cellular metabolic process      &nbspID:GO:0044237** | | | | | | |
| --- | --- | --- | --- | --- | --- | --- |
| C=7363; O=187; E=141.60; R=1.32; rawP=1.42e-06; adjP=0.0018 | | | | | | |
| Index | UserID | Value | Gene Symbol | Gene Name | EntrezGene | Ensembl |
| 1 | 17476273 | NA | Zfp382 | zinc finger protein 382 | 233060 | ENSMUSG00000074220 |
| 2 | 17232843 | NA | Zbtb24 | zinc finger and BTB domain containing 24 | 268294 | ENSMUSG00000019826 |
| 3 | 17344794 | NA | Znrd1 | zinc ribbon domain containing, 1 | 66136 | ENSMUSG00000036315 |
| 4 | 17300261 | NA | Oxa1l | oxidase assembly 1-like | 69089 | ENSMUSG00000000959 |
| 5 | 17512740 | NA | Nob1 | NIN1/RPN12 binding protein 1 homolog (S. cerevisiae) | 67619 | ENSMUSG00000003848 |
| 6 | 17224540 | NA | Tuba4a | tubulin, alpha 4A | 22145 | ENSMUSG00000026202 |
| 7 | 17368171 | NA | Bmyc | brain expressed myelocytomatosis oncogene | 107771 | ENSMUSG00000049086 |
| 8 | 17350134 | NA | Pou4f3 | POU domain, class 4, transcription factor 3 | 18998 | ENSMUSG00000024497 |
| 9 | 17517723 | NA | Rpp25 | ribonuclease P 25 subunit (human) | 102614 | ENSMUSG00000062309 |
| 10 | 17307134 | NA | Cryl1 | crystallin, lambda 1 | 68631 | ENSMUSG00000021947 |
| 11 | 17443181 | NA | Dnajc30 | DnaJ (Hsp40) homolog, subfamily C, member 30 | 66114 | ENSMUSG00000061118 |
| 12 | 17512103 | NA | Got2 | glutamate oxaloacetate transaminase 2, mitochondrial | 14719 | ENSMUSG00000031672 |
| 13 | 17369862 | NA | Dpm2 | dolichol-phosphate (beta-D) mannosyltransferase 2 | 13481 | ENSMUSG00000026810 |
| 14 | 17336829 | NA | Lsm2 | LSM2 homolog, U6 small nuclear RNA associated (S. cerevisiae) | 27756 | ENSMUSG00000007050 |
| 15 | 17288454 | NA | Irx4 | Iroquois related homeobox 4 (Drosophila) | 50916 | ENSMUSG00000021604 |
| 16 | 17467996 | NA | Mrpl19 | mitochondrial ribosomal protein L19 | 56284 | ENSMUSG00000030045 |
| 17 | 17322559 | NA | Hmox2 | heme oxygenase (decycling) 2 | 15369 | ENSMUSG00000004070 |
| 18 | 17306758 | NA | Tm9sf1 | transmembrane 9 superfamily member 1 | 74140 | ENSMUSG00000002320 |
| 19 | 17404329 | NA | Gyg | glycogenin | 27357 | ENSMUSG00000019528 |
| 20 | 17321467 | NA | Tuba1b | tubulin, alpha 1B | 22143 | ENSMUSG00000023004 |
| 21 | 17288160 | NA | Cdk20 | cyclin-dependent kinase 20 | 105278 | ENSMUSG00000021483 |
| 22 | 17538096 | NA | Rnf128 | ring finger protein 128 | 66889 | ENSMUSG00000031438 |
| 23 | 17447099 | NA | Ctbp1 | C-terminal binding protein 1 | 13016 | ENSMUSG00000037373 |
| 24 | 17447089 | NA | Ctbp1 | C-terminal binding protein 1 | 13016 | ENSMUSG00000037373 |
| 25 | 17447100 | NA | Ctbp1 | C-terminal binding protein 1 | 13016 | ENSMUSG00000037373 |
| 26 | 17447098 | NA | Ctbp1 | C-terminal binding protein 1 | 13016 | ENSMUSG00000037373 |
| 27 | 17343617 | NA | Rab11b | RAB11B, member RAS oncogene family | 19326 | ENSMUSG00000077450 |
| 28 | 17379187 | NA | Ift52 | intraflagellar transport 52 | 245866 | ENSMUSG00000017858 |
| 29 | 17265082 | NA | Eif5a | eukaryotic translation initiation factor 5A | 276770 | ENSMUSG00000078812 |
| 30 | 17297750 | NA | Ppif | peptidylprolyl isomerase F (cyclophilin F) | 105675 | ENSMUSG00000021868 |
| 31 | 17233630 | NA | Psap | prosaposin | 19156 | ENSMUSG00000004207 |
| 32 | 17520177 | NA | Mthfs | 5, 10-methenyltetrahydrofolate synthetase | 107885 | ENSMUSG00000066442 |
| 33 | 17480102 | NA | Sytl2 | synaptotagmin-like 2 | 83671 | ENSMUSG00000030616 |
| 34 | 17521143 | NA | Wdr82 | WD repeat domain containing 82 | 77305 | ENSMUSG00000020257 |
| 35 | 17528778 | NA | Ccpg1 | cell cycle progression 1 | 72278 | ENSMUSG00000034563 |
| 36 | 17278188 | NA | Otub2 | OTU domain, ubiquitin aldehyde binding 2 | 68149 | ENSMUSG00000021203 |
| 37 | 17225499 | NA | Hes6 | hairy and enhancer of split 6 (Drosophila) | 55927 | ENSMUSG00000067071 |
| 38 | 17312944 | NA | Polr2f | polymerase (RNA) II (DNA directed) polypeptide F | 69833 | ENSMUSG00000033020 |
| 39 | 17312939 | NA | Polr2f | polymerase (RNA) II (DNA directed) polypeptide F | 69833 | ENSMUSG00000033020 |
| 40 | 17312941 | NA | Polr2f | polymerase (RNA) II (DNA directed) polypeptide F | 69833 | ENSMUSG00000033020 |
| 41 | 17334722 | NA | Rpusd1 | RNA pseudouridylate synthase domain containing 1 | 106707 | ENSMUSG00000041199 |
| 42 | 17517576 | NA | Hmg20a | high mobility group 20A | 66867 | ENSMUSG00000032329 |
| 43 | 17393225 | NA | Pigu | phosphatidylinositol glycan anchor biosynthesis, class U | 228812 | ENSMUSG00000038383 |
| 44 | 17404011 | NA | Hey1 | hairy/enhancer-of-split related with YRPW motif 1 | 15213 | ENSMUSG00000040289 |
| 45 | 17400222 | NA | Vps72 | vacuolar protein sorting 72 (yeast) | 21427 | ENSMUSG00000008958 |
| 46 | 17499396 | NA | Fbxo25 | F-box protein 25 | 66822 | ENSMUSG00000038365 |
| 47 | 17246850 | NA | Zmat5 | zinc finger, matrin type 5 | 67178 | ENSMUSG00000009076 |
| 48 | 17324664 | NA | Dlg1 | discs, large homolog 1 (Drosophila) | 13383 | ENSMUSG00000022770 |
| 49 | 17396369 | NA | Nceh1 | arylacetamide deacetylase-like 1 | 320024 | ENSMUSG00000027698 |
| 50 | 17502191 | NA | Mrpl34 | mitochondrial ribosomal protein L34 | 94065 | ENSMUSG00000034880 |
| 51 | 17529231 | NA | Phip | pleckstrin homology domain interacting protein | 83946 | ENSMUSG00000032253 |
| 52 | 17316625 | NA | Ubr5 | ubiquitin protein ligase E3 component n-recognin 5 | 70790 | ENSMUSG00000037487 |
| 53 | 17277370 | NA | Eif2b2 | eukaryotic translation initiation factor 2B, subunit 2 beta | 217715 | ENSMUSG00000004788 |
| 54 | 17483220 | NA | Cdipt | CDP-diacylglycerol--inositol 3-phosphatidyltransferase (phosphatidylinositol synthase) | 52858 | ENSMUSG00000030682 |
| 55 | 17245709 | NA | Os9 | amplified in osteosarcoma | 216440 | ENSMUSG00000040462 |
| 56 | 17512009 | NA | Csnk2a2 | casein kinase 2, alpha prime polypeptide | 13000 | ENSMUSG00000046707 |
| 57 | 17424319 | NA | Sigmar1 | sigma non-opioid intracellular receptor 1 | 18391 | ENSMUSG00000036078 |
| 58 | 17503816 | NA | Irx6 | Iroquois related homeobox 6 (Drosophila) | 64379 | ENSMUSG00000031738 |
| 59 | 17282649 | NA | Rps6kl1 | ribosomal protein S6 kinase-like 1 | 238323 | ENSMUSG00000019235 |
| 60 | 17260221 | NA | Pold2 | polymerase (DNA directed), delta 2, regulatory subunit | 18972 | ENSMUSG00000020471 |
| 61 | 17365493 | NA | Obfc1 | oligonucleotide/oligosaccharide-binding fold containing 1 | 108689 | ENSMUSG00000042694 |
| 62 | 17538790 | NA | Huwe1 | HECT, UBA and WWE domain containing 1 | 59026 | ENSMUSG00000025261 |
| 63 | 17446580 | NA | Shh | sonic hedgehog | 20423 | ENSMUSG00000002633 |
| 64 | 17470879 | NA | Tpi1 | triosephosphate isomerase 1 | 21991 | ENSMUSG00000023456 |
| 65 | 17281971 | NA | Sgpp1 | sphingosine-1-phosphate phosphatase 1 | 81535 | ENSMUSG00000021054 |
| 66 | 17230408 | NA | Adck3 | aarF domain containing kinase 3 | 67426 | ENSMUSG00000026489 |
| 67 | 17238549 | NA | Wibg | within bgcn homolog (Drosophila) | 78428 | ENSMUSG00000064030 |
| 68 | 17212355 | NA | Nck2 | non-catalytic region of tyrosine kinase adaptor protein 2 | 17974 | ENSMUSG00000066877 |
| 69 | 17431502 | NA | Lypla2 | lysophospholipase 2 | 26394 | ENSMUSG00000028670 |
| 70 | 17303496 | NA | Fezf2 | Fez family zinc finger 2 | 54713 | ENSMUSG00000021743 |
| 71 | 17256565 | NA | Tubg2 | tubulin, gamma 2 | 103768 | ENSMUSG00000045007 |
| 72 | 17500275 | NA | Erlin2 | ER lipid raft associated 2 | 244373 | ENSMUSG00000031483 |
| 73 | 17345519 | NA | Rrp36 | ribosomal RNA processing 36 homolog (S. cerevisiae) | 224823 | ENSMUSG00000023971 |
| 74 | 17463422 | NA | Nrip2 | nuclear receptor interacting protein 2 | 60345 | ENSMUSG00000001520 |
| 75 | 17211335 | NA | Tfap2d | transcription factor AP-2, delta | 226896 | ENSMUSG00000042596 |
| 76 | 17506631 | NA | Tubb3 | tubulin, beta 3 class III | 22152 | ENSMUSG00000062380 |
| 77 | 17321722 | NA | Tfcp2 | transcription factor CP2 | 21422 | ENSMUSG00000009733 |
| 78 | 17213153 | NA | Nif3l1 | Ngg1 interacting factor 3-like 1 (S. pombe) | 65102 | ENSMUSG00000026036 |
| 79 | 17235584 | NA | Dapk3 | death-associated protein kinase 3 | 13144 | ENSMUSG00000034974 |
| 80 | 17477670 | NA | Rras | Harvey rat sarcoma oncogene, subgroup R | 20130 | ENSMUSG00000038387 |
| 81 | 17405819 | NA | B3galnt1 | UDP-GalNAc:betaGlcNAc beta 1,3-galactosaminyltransferase, polypeptide 1 | 26879 | ENSMUSG00000043300 |
| 82 | 17300411 | NA | Thtpa | thiamine triphosphatase | 105663 | ENSMUSG00000045691 |
| 83 | 17498821 | NA | Snapc2 | small nuclear RNA activating complex, polypeptide 2 | 102209 | ENSMUSG00000011837 |
| 84 | 17460879 | NA | Hdac11 | histone deacetylase 11 | 232232 | ENSMUSG00000034245 |
| 85 | 17256618 | NA | Vps25 | vacuolar protein sorting 25 (yeast) | 28084 | ENSMUSG00000078656 |
| 86 | 17444100 | NA | Chst12 | carbohydrate sulfotransferase 12 | 59031 | ENSMUSG00000036599 |
| 87 | 17517532 | NA | Isl2 | insulin related protein 2 (islet 2) | 104360 | ENSMUSG00000032318 |
| 88 | 17494637 | NA | Mrpl17 | mitochondrial ribosomal protein L17 | 27397 | ENSMUSG00000030879 |
| 89 | 17513871 | NA | Chmp1a | charged multivesicular body protein 1A | 234852 | ENSMUSG00000000743 |
| 90 | 17359994 | NA | Fbxl15 | F-box and leucine-rich repeat protein 15 | 68431 | ENSMUSG00000025226 |
| 91 | 17484068 | NA | Lhpp | phospholysine phosphohistidine inorganic pyrophosphate phosphatase | 76429 | ENSMUSG00000030946 |
| 92 | 17497366 | NA | Ebf3 | early B cell factor 3 | 13593 | ENSMUSG00000010476 |
| 93 | 17446322 | NA | Prkag2 | protein kinase, AMP-activated, gamma 2 non-catalytic subunit | 108099 | ENSMUSG00000028944 |
| 94 | 17524523 | NA | Eif3g | eukaryotic translation initiation factor 3, subunit G | 53356 | ENSMUSG00000070319 |
| 95 | 17306835 | NA | Rabggta | Rab geranylgeranyl transferase, a subunit | 56187 | ENSMUSG00000040472 |
| 96 | 17269638 | NA | Rab5c | RAB5C, member RAS oncogene family | 19345 | ENSMUSG00000019173 |
| 97 | 17505367 | NA | Txnl4b | thioredoxin-like 4B | 234723 | ENSMUSG00000031723 |
| 98 | 17256549 | NA | Tubg1 | tubulin, gamma 1 | 103733 | ENSMUSG00000035198 |
| 99 | 17498370 | NA | Nadsyn1 | NAD synthetase 1 | 78914 | ENSMUSG00000031090 |
| 100 | 17494081 | NA | Rhog | ras homolog gene family, member G | 56212 | ENSMUSG00000073982 |
| 101 | 17235268 | NA | Ndufs7 | NADH dehydrogenase (ubiquinone) Fe-S protein 7 | 75406 | ENSMUSG00000020153 |
| 102 | 17532045 | NA | Plcd1 | phospholipase C, delta 1 | 18799 | ENSMUSG00000010660 |
| 103 | 17274448 | NA | Cpsf3 | cleavage and polyadenylation specificity factor 3 | 54451 | ENSMUSG00000054309 |
| 104 | 17422117 | NA | Acot7 | acyl-CoA thioesterase 7 | 70025 | ENSMUSG00000028937 |
| 105 | 17519868 | NA | Htr1b | 5-hydroxytryptamine (serotonin) receptor 1B | 15551 | ENSMUSG00000049511 |
| 106 | 17536264 | NA | Pcyt1b | phosphate cytidylyltransferase 1, choline, beta isoform | 236899 | ENSMUSG00000035246 |
| 107 | 17515170 | NA | Ilf3 | interleukin enhancer binding factor 3 | 16201 | ENSMUSG00000032178 |
| 108 | 17357213 | NA | Zbtb3 | zinc finger and BTB domain containing 3 | 75291 | ENSMUSG00000071661 |
| 109 | 17430853 | NA | Med18 | mediator of RNA polymerase II transcription, subunit 18 homolog (yeast) | 67219 | ENSMUSG00000066042 |
| 110 | 17225179 | NA | Pde6d | phosphodiesterase 6D, cGMP-specific, rod, delta | 18582 | ENSMUSG00000026239 |
| 111 | 17521014 | NA | Acad11 | acyl-Coenzyme A dehydrogenase family, member 11 | 102632 | ENSMUSG00000090150 |
| 112 | 17370285 | NA | Mrrf | mitochondrial ribosome recycling factor | 67871 | ENSMUSG00000026887 |
| 113 | 17361988 | NA | Arl2 | ADP-ribosylation factor-like 2 | 56327 | ENSMUSG00000024944 |
| 114 | 17229948 | NA | Dusp23 | dual specificity phosphatase 23 | 68440 | ENSMUSG00000026544 |
| 115 | 17502583 | NA | Mcm5 | minichromosome maintenance deficient 5, cell division cycle 46 (S. cerevisiae) | 17218 | ENSMUSG00000005410 |
| 116 | 17511296 | NA | Wdr83 | WD repeat domain containing 83 | 67836 | ENSMUSG00000005150 |
| 117 | 17539434 | NA | Ctps2 | cytidine 5'-triphosphate synthase 2 | 55936 | ENSMUSG00000031360 |
| 118 | 17341521 | NA | Thoc6 | THO complex 6 homolog (Drosophila) | 386612 | ENSMUSG00000041319 |
| 119 | 17250141 | NA | Zfp39 | zinc finger protein 39 | 22698 | ENSMUSG00000037001 |
| 120 | 17519718 | NA | Mto1 | mitochondrial translation optimization 1 homolog (S. cerevisiae) | 68291 | ENSMUSG00000032342 |
| 121 | 17521422 | NA | Hyal2 | hyaluronoglucosaminidase 2 | 15587 | ENSMUSG00000010047 |
| 122 | 17317208 | NA | Derl1 | Der1-like domain family, member 1 | 67819 | ENSMUSG00000022365 |
| 123 | 17235714 | NA | Dohh | deoxyhypusine hydroxylase/monooxygenase | 102115 | ENSMUSG00000078440 |
| 124 | 17540589 | NA | Klhl13 | kelch-like 13 (Drosophila) | 67455 | ENSMUSG00000036782 |
| 125 | 17428858 | NA | Rnf220 | ring finger protein 220 | 66743 | ENSMUSG00000028677 |
| 126 | 17428857 | NA | Rnf220 | ring finger protein 220 | 66743 | ENSMUSG00000028677 |
| 127 | 17468195 | NA | Stambp | STAM binding protein | 70527 | ENSMUSG00000006906 |
| 128 | 17408684 | NA | Dclre1b | DNA cross-link repair 1B, PSO2 homolog (S. cerevisiae) | 140917 | ENSMUSG00000027845 |
| 129 | 17436077 | NA | Nrbp1 | nuclear receptor binding protein 1 | 192292 | ENSMUSG00000029148 |
| 130 | 17342015 | NA | Tbl3 | transducin (beta)-like 3 | 213773 | ENSMUSG00000040688 |
| 131 | 17252170 | NA | Rnf167 | ring finger protein 167 | 70510 | ENSMUSG00000040746 |
| 132 | 17543988 | NA | Taf9b | TAF9B RNA polymerase II, TATA box binding protein (TBP)-associated factor | 407786 | ENSMUSG00000047242 |
| 133 | 17509721 | NA | Tufm | Tu translation elongation factor, mitochondrial | 233870 | ENSMUSG00000073838 |
| 134 | 17459676 | NA | Retsat | retinol saturase (all trans retinol 13,14 reductase) | 67442 | ENSMUSG00000056666 |
| 135 | 17279858 | NA | Fkbp1b | FK506 binding protein 1b | 14226 | ENSMUSG00000020635 |
| 136 | 17488463 | NA | Med29 | mediator complex subunit 29 | 67224 | ENSMUSG00000003444 |
| 137 | 17391270 | NA | Kcnip3 | Kv channel interacting protein 3, calsenilin | 56461 | ENSMUSG00000079056 |
| 138 | 17324576 | NA | Hrasls | HRAS-like suppressor | 27281 | ENSMUSG00000022525 |
| 139 | 17531834 | NA | Fbxl2 | F-box and leucine-rich repeat protein 2 | 72179 | ENSMUSG00000032507 |
| 140 | 17460099 | NA | Vax2 | ventral anterior homeobox containing gene 2 | 24113 | ENSMUSG00000034777 |
| 141 | 17257060 | NA | Nmt1 | N-myristoyltransferase 1 | 18107 | ENSMUSG00000020936 |
| 142 | 17292107 | NA | Tbc1d7 | TBC1 domain family, member 7 | 67046 | ENSMUSG00000021368 |
| 143 | 17498239 | NA | Kcnq1ot1 | KCNQ1 overlapping transcript 1 | 63830 | NULL |
| 144 | 17413573 | NA | Grhpr | glyoxylate reductase/hydroxypyruvate reductase | 76238 | ENSMUSG00000035637 |
| 145 | 17512479 | NA | Acd | adrenocortical dysplasia | 497652 | ENSMUSG00000038000 |
| 146 | 17288716 | NA | Glrx | glutaredoxin | 93692 | ENSMUSG00000021591 |
| 147 | 17429495 | NA | Nfyc | nuclear transcription factor-Y gamma | 18046 | ENSMUSG00000032897 |
| 148 | 17361855 | NA | Pola2 | polymerase (DNA directed), alpha 2 | 18969 | ENSMUSG00000024833 |
| 149 | 17527532 | NA | Mpi | mannose phosphate isomerase | 110119 | ENSMUSG00000032306 |
| 150 | 17353639 | NA | Dnajc18 | DnaJ (Hsp40) homolog, subfamily C, member 18 | 76594 | ENSMUSG00000024350 |
| 151 | 17512463 | NA | Atp6v0d1 | ATPase, H+ transporting, lysosomal V0 subunit D1 | 11972 | ENSMUSG00000013160 |
| 152 | 17512466 | NA | Atp6v0d1 | ATPase, H+ transporting, lysosomal V0 subunit D1 | 11972 | ENSMUSG00000013160 |
| 153 | 17252635 | NA | Shpk | sedoheptulokinase | 74637 | ENSMUSG00000005951 |
| 154 | 17212813 | NA | Mstn | myostatin | 17700 | ENSMUSG00000026100 |
| 155 | 17230945 | NA | Smyd2 | SET and MYND domain containing 2 | 226830 | ENSMUSG00000026603 |
| 156 | 17313008 | NA | Cby1 | chibby homolog 1 (Drosophila) | 73739 | ENSMUSG00000022428 |
| 157 | 17342359 | NA | Haghl | hydroxyacylglutathione hydrolase-like | 68977 | ENSMUSG00000061046 |
| 158 | 17468113 | NA | Ino80b | INO80 complex subunit B | 70020 | ENSMUSG00000030034 |
| 159 | 17347558 | NA | Cdkl4 | cyclin-dependent kinase-like 4 | 381113 | ENSMUSG00000033966 |
| 160 | 17503023 | NA | Asf1b | ASF1 anti-silencing function 1 homolog B (S. cerevisiae) | 66929 | ENSMUSG00000005470 |
| 161 | 17283939 | NA | Wars | tryptophanyl-tRNA synthetase | 22375 | ENSMUSG00000021266 |
| 162 | 17283941 | NA | Wars | tryptophanyl-tRNA synthetase | 22375 | ENSMUSG00000021266 |
| 163 | 17283930 | NA | Wars | tryptophanyl-tRNA synthetase | 22375 | ENSMUSG00000021266 |
| 164 | 17283938 | NA | Wars | tryptophanyl-tRNA synthetase | 22375 | ENSMUSG00000021266 |
| 165 | 17258457 | NA | Sap30bp | SAP30 binding protein | 57230 | ENSMUSG00000020755 |
| 166 | 17454256 | NA | Taf6 | TAF6 RNA polymerase II, TATA box binding protein (TBP)-associated factor | 21343 | ENSMUSG00000036980 |
| 167 | 17432967 | NA | Ubiad1 | UbiA prenyltransferase domain containing 1 | 71707 | ENSMUSG00000047719 |
| 168 | 17510365 | NA | Pgls | 6-phosphogluconolactonase | 66171 | ENSMUSG00000031807 |
| 169 | 17526273 | NA | Trappc4 | trafficking protein particle complex 4 | 60409 | ENSMUSG00000032112 |
| 170 | 17526271 | NA | Trappc4 | trafficking protein particle complex 4 | 60409 | ENSMUSG00000032112 |
| 171 | 17526272 | NA | Trappc4 | trafficking protein particle complex 4 | 60409 | ENSMUSG00000032112 |
| 172 | 17231118 | NA | Rcor3 | REST corepressor 3 | 214742 | ENSMUSG00000037395 |
| 173 | 17298407 | NA | Bap1 | Brca1 associated protein 1 | 104416 | ENSMUSG00000021901 |
| 174 | 17318877 | NA | Txn2 | thioredoxin 2 | 56551 | ENSMUSG00000005354 |
| 175 | 17329163 | NA | Camk2n2 | calcium/calmodulin-dependent protein kinase II inhibitor 2 | 73047 | ENSMUSG00000051146 |
| 176 | 17265186 | NA | Rnasek | ribonuclease, RNase K | 52898 | ENSMUSG00000093989 |
| 177 | 17221014 | NA | Cd34 | CD34 antigen | 12490 | ENSMUSG00000016494 |
| 178 | 17273280 | NA | Stra13 | stimulated by retinoic acid 13 | 20892 | ENSMUSG00000025144 |
| 179 | 17301342 | NA | Ints9 | integrator complex subunit 9 | 210925 | ENSMUSG00000021975 |
| 180 | 17393357 | NA | Eif6 | eukaryotic translation initiation factor 6 | 16418 | ENSMUSG00000027613 |
| 181 | 17412593 | NA | Srsf12 | serine/arginine-rich splicing factor 12 | 272009 | ENSMUSG00000054679 |
| 182 | 17213990 | NA | Atic | 5-aminoimidazole-4-carboxamide ribonucleotide formyltransferase/IMP cyclohydrolase | 108147 | ENSMUSG00000026192 |
| 183 | 17504160 | NA | Polr2c | polymerase (RNA) II (DNA directed) polypeptide C | 20021 | ENSMUSG00000031783 |
| 184 | 17455093 | NA | Zkscan14 | zinc finger with KRAB and SCAN domains 14 | 67235 | ENSMUSG00000029627 |
| 185 | 17508691 | NA | Rbpms | RNA binding protein gene with multiple splicing | 19663 | ENSMUSG00000031586 |
| 186 | 17222001 | NA | Prim2 | DNA primase, p58 subunit | 19076 | ENSMUSG00000026134 |
| 187 | 17232649 | NA | Fyn | Fyn proto-oncogene | 14360 | ENSMUSG00000019843 |
| 188 | 17328810 | NA | Dgcr14 | DiGeorge syndrome critical region gene 14 | 27886 | ENSMUSG00000003527 |
| 189 | 17395844 | NA | Stmn3 | stathmin-like 3 | 20262 | ENSMUSG00000027581 |
| 190 | 17219286 | NA | Dedd | death effector domain-containing | 21945 | ENSMUSG00000013973 |
| 191 | 17241780 | NA | Ube2d1 | ubiquitin-conjugating enzyme E2D 1 | 216080 | ENSMUSG00000019927 |
| 192 | 17334495 | NA | Nme3 | NME/NM23 nucleoside diphosphate kinase 3 | 79059 | ENSMUSG00000073435 |
| 193 | 17383588 | NA | Ccbl1 | cysteine conjugate-beta lyase 1 | 70266 | ENSMUSG00000039648 |
| 194 | 17313199 | NA | Adsl | adenylosuccinate lyase | 11564 | ENSMUSG00000022407 |
| 195 | 17232215 | NA | Moxd1 | monooxygenase, DBH-like 1 | 59012 | ENSMUSG00000020000 |
| 196 | 17418447 | NA | Meaf6 | MYST/Esa1-associated factor 6 | 70088 | ENSMUSG00000028863 |
| 197 | 17356202 | NA | Pold4 | polymerase (DNA-directed), delta 4 | 69745 | ENSMUSG00000024854 |
| 198 | 17421972 | NA | Errfi1 | ERBB receptor feedback inhibitor 1 | 74155 | ENSMUSG00000028967 |
| 199 | 17330373 | NA | Adprh | ADP-ribosylarginine hydrolase | 11544 | ENSMUSG00000002844 |

  
  

| **Database:biological process      &nbspName:single-organism metabolic process      &nbspID:GO:0044710** | | | | | | |
| --- | --- | --- | --- | --- | --- | --- |
| C=8188; O=200; E=157.46; R=1.27; rawP=7.71e-06; adjP=0.0044 | | | | | | |
| Index | UserID | Value | Gene Symbol | Gene Name | EntrezGene | Ensembl |
| 1 | 17476273 | NA | Zfp382 | zinc finger protein 382 | 233060 | ENSMUSG00000074220 |
| 2 | 17232843 | NA | Zbtb24 | zinc finger and BTB domain containing 24 | 268294 | ENSMUSG00000019826 |
| 3 | 17344794 | NA | Znrd1 | zinc ribbon domain containing, 1 | 66136 | ENSMUSG00000036315 |
| 4 | 17300261 | NA | Oxa1l | oxidase assembly 1-like | 69089 | ENSMUSG00000000959 |
| 5 | 17512740 | NA | Nob1 | NIN1/RPN12 binding protein 1 homolog (S. cerevisiae) | 67619 | ENSMUSG00000003848 |
| 6 | 17224540 | NA | Tuba4a | tubulin, alpha 4A | 22145 | ENSMUSG00000026202 |
| 7 | 17503910 | NA | Ogfod1 | 2-oxoglutarate and iron-dependent oxygenase domain containing 1 | 270086 | ENSMUSG00000033009 |
| 8 | 17368171 | NA | Bmyc | brain expressed myelocytomatosis oncogene | 107771 | ENSMUSG00000049086 |
| 9 | 17350134 | NA | Pou4f3 | POU domain, class 4, transcription factor 3 | 18998 | ENSMUSG00000024497 |
| 10 | 17517723 | NA | Rpp25 | ribonuclease P 25 subunit (human) | 102614 | ENSMUSG00000062309 |
| 11 | 17307134 | NA | Cryl1 | crystallin, lambda 1 | 68631 | ENSMUSG00000021947 |
| 12 | 17443181 | NA | Dnajc30 | DnaJ (Hsp40) homolog, subfamily C, member 30 | 66114 | ENSMUSG00000061118 |
| 13 | 17512103 | NA | Got2 | glutamate oxaloacetate transaminase 2, mitochondrial | 14719 | ENSMUSG00000031672 |
| 14 | 17369862 | NA | Dpm2 | dolichol-phosphate (beta-D) mannosyltransferase 2 | 13481 | ENSMUSG00000026810 |
| 15 | 17336829 | NA | Lsm2 | LSM2 homolog, U6 small nuclear RNA associated (S. cerevisiae) | 27756 | ENSMUSG00000007050 |
| 16 | 17288454 | NA | Irx4 | Iroquois related homeobox 4 (Drosophila) | 50916 | ENSMUSG00000021604 |
| 17 | 17467996 | NA | Mrpl19 | mitochondrial ribosomal protein L19 | 56284 | ENSMUSG00000030045 |
| 18 | 17322559 | NA | Hmox2 | heme oxygenase (decycling) 2 | 15369 | ENSMUSG00000004070 |
| 19 | 17404329 | NA | Gyg | glycogenin | 27357 | ENSMUSG00000019528 |
| 20 | 17321467 | NA | Tuba1b | tubulin, alpha 1B | 22143 | ENSMUSG00000023004 |
| 21 | 17288160 | NA | Cdk20 | cyclin-dependent kinase 20 | 105278 | ENSMUSG00000021483 |
| 22 | 17538096 | NA | Rnf128 | ring finger protein 128 | 66889 | ENSMUSG00000031438 |
| 23 | 17447099 | NA | Ctbp1 | C-terminal binding protein 1 | 13016 | ENSMUSG00000037373 |
| 24 | 17447089 | NA | Ctbp1 | C-terminal binding protein 1 | 13016 | ENSMUSG00000037373 |
| 25 | 17447100 | NA | Ctbp1 | C-terminal binding protein 1 | 13016 | ENSMUSG00000037373 |
| 26 | 17447098 | NA | Ctbp1 | C-terminal binding protein 1 | 13016 | ENSMUSG00000037373 |
| 27 | 17343617 | NA | Rab11b | RAB11B, member RAS oncogene family | 19326 | ENSMUSG00000077450 |
| 28 | 17379187 | NA | Ift52 | intraflagellar transport 52 | 245866 | ENSMUSG00000017858 |
| 29 | 17440361 | NA | Plcxd1 | phosphatidylinositol-specific phospholipase C, X domain containing 1 | 403178 | ENSMUSG00000064247 |
| 30 | 17246284 | NA | Suox | sulfite oxidase | 211389 | ENSMUSG00000049858 |
| 31 | 17437611 | NA | Pgm1 | phosphoglucomutase 1 | 66681 | ENSMUSG00000029171 |
| 32 | 17265082 | NA | Eif5a | eukaryotic translation initiation factor 5A | 276770 | ENSMUSG00000078812 |
| 33 | 17297750 | NA | Ppif | peptidylprolyl isomerase F (cyclophilin F) | 105675 | ENSMUSG00000021868 |
| 34 | 17233630 | NA | Psap | prosaposin | 19156 | ENSMUSG00000004207 |
| 35 | 17520177 | NA | Mthfs | 5, 10-methenyltetrahydrofolate synthetase | 107885 | ENSMUSG00000066442 |
| 36 | 17521143 | NA | Wdr82 | WD repeat domain containing 82 | 77305 | ENSMUSG00000020257 |
| 37 | 17528778 | NA | Ccpg1 | cell cycle progression 1 | 72278 | ENSMUSG00000034563 |
| 38 | 17278188 | NA | Otub2 | OTU domain, ubiquitin aldehyde binding 2 | 68149 | ENSMUSG00000021203 |
| 39 | 17225499 | NA | Hes6 | hairy and enhancer of split 6 (Drosophila) | 55927 | ENSMUSG00000067071 |
| 40 | 17312944 | NA | Polr2f | polymerase (RNA) II (DNA directed) polypeptide F | 69833 | ENSMUSG00000033020 |
| 41 | 17312939 | NA | Polr2f | polymerase (RNA) II (DNA directed) polypeptide F | 69833 | ENSMUSG00000033020 |
| 42 | 17312941 | NA | Polr2f | polymerase (RNA) II (DNA directed) polypeptide F | 69833 | ENSMUSG00000033020 |
| 43 | 17334722 | NA | Rpusd1 | RNA pseudouridylate synthase domain containing 1 | 106707 | ENSMUSG00000041199 |
| 44 | 17517576 | NA | Hmg20a | high mobility group 20A | 66867 | ENSMUSG00000032329 |
| 45 | 17393225 | NA | Pigu | phosphatidylinositol glycan anchor biosynthesis, class U | 228812 | ENSMUSG00000038383 |
| 46 | 17404011 | NA | Hey1 | hairy/enhancer-of-split related with YRPW motif 1 | 15213 | ENSMUSG00000040289 |
| 47 | 17400222 | NA | Vps72 | vacuolar protein sorting 72 (yeast) | 21427 | ENSMUSG00000008958 |
| 48 | 17499396 | NA | Fbxo25 | F-box protein 25 | 66822 | ENSMUSG00000038365 |
| 49 | 17246850 | NA | Zmat5 | zinc finger, matrin type 5 | 67178 | ENSMUSG00000009076 |
| 50 | 17324664 | NA | Dlg1 | discs, large homolog 1 (Drosophila) | 13383 | ENSMUSG00000022770 |
| 51 | 17396369 | NA | Nceh1 | arylacetamide deacetylase-like 1 | 320024 | ENSMUSG00000027698 |
| 52 | 17502191 | NA | Mrpl34 | mitochondrial ribosomal protein L34 | 94065 | ENSMUSG00000034880 |
| 53 | 17529231 | NA | Phip | pleckstrin homology domain interacting protein | 83946 | ENSMUSG00000032253 |
| 54 | 17316625 | NA | Ubr5 | ubiquitin protein ligase E3 component n-recognin 5 | 70790 | ENSMUSG00000037487 |
| 55 | 17277370 | NA | Eif2b2 | eukaryotic translation initiation factor 2B, subunit 2 beta | 217715 | ENSMUSG00000004788 |
| 56 | 17483220 | NA | Cdipt | CDP-diacylglycerol--inositol 3-phosphatidyltransferase (phosphatidylinositol synthase) | 52858 | ENSMUSG00000030682 |
| 57 | 17245709 | NA | Os9 | amplified in osteosarcoma | 216440 | ENSMUSG00000040462 |
| 58 | 17512009 | NA | Csnk2a2 | casein kinase 2, alpha prime polypeptide | 13000 | ENSMUSG00000046707 |
| 59 | 17424319 | NA | Sigmar1 | sigma non-opioid intracellular receptor 1 | 18391 | ENSMUSG00000036078 |
| 60 | 17503816 | NA | Irx6 | Iroquois related homeobox 6 (Drosophila) | 64379 | ENSMUSG00000031738 |
| 61 | 17282649 | NA | Rps6kl1 | ribosomal protein S6 kinase-like 1 | 238323 | ENSMUSG00000019235 |
| 62 | 17260221 | NA | Pold2 | polymerase (DNA directed), delta 2, regulatory subunit | 18972 | ENSMUSG00000020471 |
| 63 | 17365493 | NA | Obfc1 | oligonucleotide/oligosaccharide-binding fold containing 1 | 108689 | ENSMUSG00000042694 |
| 64 | 17538790 | NA | Huwe1 | HECT, UBA and WWE domain containing 1 | 59026 | ENSMUSG00000025261 |
| 65 | 17446580 | NA | Shh | sonic hedgehog | 20423 | ENSMUSG00000002633 |
| 66 | 17470879 | NA | Tpi1 | triosephosphate isomerase 1 | 21991 | ENSMUSG00000023456 |
| 67 | 17281971 | NA | Sgpp1 | sphingosine-1-phosphate phosphatase 1 | 81535 | ENSMUSG00000021054 |
| 68 | 17230408 | NA | Adck3 | aarF domain containing kinase 3 | 67426 | ENSMUSG00000026489 |
| 69 | 17238549 | NA | Wibg | within bgcn homolog (Drosophila) | 78428 | ENSMUSG00000064030 |
| 70 | 17212355 | NA | Nck2 | non-catalytic region of tyrosine kinase adaptor protein 2 | 17974 | ENSMUSG00000066877 |
| 71 | 17431502 | NA | Lypla2 | lysophospholipase 2 | 26394 | ENSMUSG00000028670 |
| 72 | 17307695 | NA | Msra | methionine sulfoxide reductase A | 110265 | ENSMUSG00000054733 |
| 73 | 17303496 | NA | Fezf2 | Fez family zinc finger 2 | 54713 | ENSMUSG00000021743 |
| 74 | 17256565 | NA | Tubg2 | tubulin, gamma 2 | 103768 | ENSMUSG00000045007 |
| 75 | 17500275 | NA | Erlin2 | ER lipid raft associated 2 | 244373 | ENSMUSG00000031483 |
| 76 | 17345519 | NA | Rrp36 | ribosomal RNA processing 36 homolog (S. cerevisiae) | 224823 | ENSMUSG00000023971 |
| 77 | 17463422 | NA | Nrip2 | nuclear receptor interacting protein 2 | 60345 | ENSMUSG00000001520 |
| 78 | 17211335 | NA | Tfap2d | transcription factor AP-2, delta | 226896 | ENSMUSG00000042596 |
| 79 | 17506631 | NA | Tubb3 | tubulin, beta 3 class III | 22152 | ENSMUSG00000062380 |
| 80 | 17309825 | NA | Plcxd3 | phosphatidylinositol-specific phospholipase C, X domain containing 3 | 239318 | ENSMUSG00000049148 |
| 81 | 17321722 | NA | Tfcp2 | transcription factor CP2 | 21422 | ENSMUSG00000009733 |
| 82 | 17213153 | NA | Nif3l1 | Ngg1 interacting factor 3-like 1 (S. pombe) | 65102 | ENSMUSG00000026036 |
| 83 | 17235584 | NA | Dapk3 | death-associated protein kinase 3 | 13144 | ENSMUSG00000034974 |
| 84 | 17477670 | NA | Rras | Harvey rat sarcoma oncogene, subgroup R | 20130 | ENSMUSG00000038387 |
| 85 | 17405819 | NA | B3galnt1 | UDP-GalNAc:betaGlcNAc beta 1,3-galactosaminyltransferase, polypeptide 1 | 26879 | ENSMUSG00000043300 |
| 86 | 17300411 | NA | Thtpa | thiamine triphosphatase | 105663 | ENSMUSG00000045691 |
| 87 | 17498821 | NA | Snapc2 | small nuclear RNA activating complex, polypeptide 2 | 102209 | ENSMUSG00000011837 |
| 88 | 17460879 | NA | Hdac11 | histone deacetylase 11 | 232232 | ENSMUSG00000034245 |
| 89 | 17256618 | NA | Vps25 | vacuolar protein sorting 25 (yeast) | 28084 | ENSMUSG00000078656 |
| 90 | 17444100 | NA | Chst12 | carbohydrate sulfotransferase 12 | 59031 | ENSMUSG00000036599 |
| 91 | 17517532 | NA | Isl2 | insulin related protein 2 (islet 2) | 104360 | ENSMUSG00000032318 |
| 92 | 17494637 | NA | Mrpl17 | mitochondrial ribosomal protein L17 | 27397 | ENSMUSG00000030879 |
| 93 | 17513871 | NA | Chmp1a | charged multivesicular body protein 1A | 234852 | ENSMUSG00000000743 |
| 94 | 17359994 | NA | Fbxl15 | F-box and leucine-rich repeat protein 15 | 68431 | ENSMUSG00000025226 |
| 95 | 17497366 | NA | Ebf3 | early B cell factor 3 | 13593 | ENSMUSG00000010476 |
| 96 | 17446322 | NA | Prkag2 | protein kinase, AMP-activated, gamma 2 non-catalytic subunit | 108099 | ENSMUSG00000028944 |
| 97 | 17524523 | NA | Eif3g | eukaryotic translation initiation factor 3, subunit G | 53356 | ENSMUSG00000070319 |
| 98 | 17306835 | NA | Rabggta | Rab geranylgeranyl transferase, a subunit | 56187 | ENSMUSG00000040472 |
| 99 | 17269638 | NA | Rab5c | RAB5C, member RAS oncogene family | 19345 | ENSMUSG00000019173 |
| 100 | 17505367 | NA | Txnl4b | thioredoxin-like 4B | 234723 | ENSMUSG00000031723 |
| 101 | 17256549 | NA | Tubg1 | tubulin, gamma 1 | 103733 | ENSMUSG00000035198 |
| 102 | 17498370 | NA | Nadsyn1 | NAD synthetase 1 | 78914 | ENSMUSG00000031090 |
| 103 | 17494081 | NA | Rhog | ras homolog gene family, member G | 56212 | ENSMUSG00000073982 |
| 104 | 17497957 | NA | Chid1 | chitinase domain containing 1 | 68038 | ENSMUSG00000025512 |
| 105 | 17235268 | NA | Ndufs7 | NADH dehydrogenase (ubiquinone) Fe-S protein 7 | 75406 | ENSMUSG00000020153 |
| 106 | 17532045 | NA | Plcd1 | phospholipase C, delta 1 | 18799 | ENSMUSG00000010660 |
| 107 | 17274448 | NA | Cpsf3 | cleavage and polyadenylation specificity factor 3 | 54451 | ENSMUSG00000054309 |
| 108 | 17422117 | NA | Acot7 | acyl-CoA thioesterase 7 | 70025 | ENSMUSG00000028937 |
| 109 | 17519868 | NA | Htr1b | 5-hydroxytryptamine (serotonin) receptor 1B | 15551 | ENSMUSG00000049511 |
| 110 | 17536264 | NA | Pcyt1b | phosphate cytidylyltransferase 1, choline, beta isoform | 236899 | ENSMUSG00000035246 |
| 111 | 17515170 | NA | Ilf3 | interleukin enhancer binding factor 3 | 16201 | ENSMUSG00000032178 |
| 112 | 17357213 | NA | Zbtb3 | zinc finger and BTB domain containing 3 | 75291 | ENSMUSG00000071661 |
| 113 | 17430853 | NA | Med18 | mediator of RNA polymerase II transcription, subunit 18 homolog (yeast) | 67219 | ENSMUSG00000066042 |
| 114 | 17225179 | NA | Pde6d | phosphodiesterase 6D, cGMP-specific, rod, delta | 18582 | ENSMUSG00000026239 |
| 115 | 17521014 | NA | Acad11 | acyl-Coenzyme A dehydrogenase family, member 11 | 102632 | ENSMUSG00000090150 |
| 116 | 17370285 | NA | Mrrf | mitochondrial ribosome recycling factor | 67871 | ENSMUSG00000026887 |
| 117 | 17361988 | NA | Arl2 | ADP-ribosylation factor-like 2 | 56327 | ENSMUSG00000024944 |
| 118 | 17229948 | NA | Dusp23 | dual specificity phosphatase 23 | 68440 | ENSMUSG00000026544 |
| 119 | 17502583 | NA | Mcm5 | minichromosome maintenance deficient 5, cell division cycle 46 (S. cerevisiae) | 17218 | ENSMUSG00000005410 |
| 120 | 17511296 | NA | Wdr83 | WD repeat domain containing 83 | 67836 | ENSMUSG00000005150 |
| 121 | 17539434 | NA | Ctps2 | cytidine 5'-triphosphate synthase 2 | 55936 | ENSMUSG00000031360 |
| 122 | 17341521 | NA | Thoc6 | THO complex 6 homolog (Drosophila) | 386612 | ENSMUSG00000041319 |
| 123 | 17250141 | NA | Zfp39 | zinc finger protein 39 | 22698 | ENSMUSG00000037001 |
| 124 | 17519718 | NA | Mto1 | mitochondrial translation optimization 1 homolog (S. cerevisiae) | 68291 | ENSMUSG00000032342 |
| 125 | 17521422 | NA | Hyal2 | hyaluronoglucosaminidase 2 | 15587 | ENSMUSG00000010047 |
| 126 | 17317208 | NA | Derl1 | Der1-like domain family, member 1 | 67819 | ENSMUSG00000022365 |
| 127 | 17501544 | NA | Npy1r | neuropeptide Y receptor Y1 | 18166 | ENSMUSG00000036437 |
| 128 | 17235714 | NA | Dohh | deoxyhypusine hydroxylase/monooxygenase | 102115 | ENSMUSG00000078440 |
| 129 | 17540589 | NA | Klhl13 | kelch-like 13 (Drosophila) | 67455 | ENSMUSG00000036782 |
| 130 | 17428858 | NA | Rnf220 | ring finger protein 220 | 66743 | ENSMUSG00000028677 |
| 131 | 17428857 | NA | Rnf220 | ring finger protein 220 | 66743 | ENSMUSG00000028677 |
| 132 | 17468195 | NA | Stambp | STAM binding protein | 70527 | ENSMUSG00000006906 |
| 133 | 17408684 | NA | Dclre1b | DNA cross-link repair 1B, PSO2 homolog (S. cerevisiae) | 140917 | ENSMUSG00000027845 |
| 134 | 17436077 | NA | Nrbp1 | nuclear receptor binding protein 1 | 192292 | ENSMUSG00000029148 |
| 135 | 17342015 | NA | Tbl3 | transducin (beta)-like 3 | 213773 | ENSMUSG00000040688 |
| 136 | 17252170 | NA | Rnf167 | ring finger protein 167 | 70510 | ENSMUSG00000040746 |
| 137 | 17543988 | NA | Taf9b | TAF9B RNA polymerase II, TATA box binding protein (TBP)-associated factor | 407786 | ENSMUSG00000047242 |
| 138 | 17509721 | NA | Tufm | Tu translation elongation factor, mitochondrial | 233870 | ENSMUSG00000073838 |
| 139 | 17459676 | NA | Retsat | retinol saturase (all trans retinol 13,14 reductase) | 67442 | ENSMUSG00000056666 |
| 140 | 17279858 | NA | Fkbp1b | FK506 binding protein 1b | 14226 | ENSMUSG00000020635 |
| 141 | 17488463 | NA | Med29 | mediator complex subunit 29 | 67224 | ENSMUSG00000003444 |
| 142 | 17391270 | NA | Kcnip3 | Kv channel interacting protein 3, calsenilin | 56461 | ENSMUSG00000079056 |
| 143 | 17324576 | NA | Hrasls | HRAS-like suppressor | 27281 | ENSMUSG00000022525 |
| 144 | 17531834 | NA | Fbxl2 | F-box and leucine-rich repeat protein 2 | 72179 | ENSMUSG00000032507 |
| 145 | 17460099 | NA | Vax2 | ventral anterior homeobox containing gene 2 | 24113 | ENSMUSG00000034777 |
| 146 | 17358777 | NA | Stambpl1 | STAM binding protein like 1 | 76630 | ENSMUSG00000024776 |
| 147 | 17257060 | NA | Nmt1 | N-myristoyltransferase 1 | 18107 | ENSMUSG00000020936 |
| 148 | 17292107 | NA | Tbc1d7 | TBC1 domain family, member 7 | 67046 | ENSMUSG00000021368 |
| 149 | 17498239 | NA | Kcnq1ot1 | KCNQ1 overlapping transcript 1 | 63830 | NULL |
| 150 | 17413573 | NA | Grhpr | glyoxylate reductase/hydroxypyruvate reductase | 76238 | ENSMUSG00000035637 |
| 151 | 17512479 | NA | Acd | adrenocortical dysplasia | 497652 | ENSMUSG00000038000 |
| 152 | 17288716 | NA | Glrx | glutaredoxin | 93692 | ENSMUSG00000021591 |
| 153 | 17540050 | NA | Ebp | phenylalkylamine Ca2+ antagonist (emopamil) binding protein | 13595 | ENSMUSG00000031168 |
| 154 | 17429495 | NA | Nfyc | nuclear transcription factor-Y gamma | 18046 | ENSMUSG00000032897 |
| 155 | 17361855 | NA | Pola2 | polymerase (DNA directed), alpha 2 | 18969 | ENSMUSG00000024833 |
| 156 | 17527532 | NA | Mpi | mannose phosphate isomerase | 110119 | ENSMUSG00000032306 |
| 157 | 17353639 | NA | Dnajc18 | DnaJ (Hsp40) homolog, subfamily C, member 18 | 76594 | ENSMUSG00000024350 |
| 158 | 17512463 | NA | Atp6v0d1 | ATPase, H+ transporting, lysosomal V0 subunit D1 | 11972 | ENSMUSG00000013160 |
| 159 | 17512466 | NA | Atp6v0d1 | ATPase, H+ transporting, lysosomal V0 subunit D1 | 11972 | ENSMUSG00000013160 |
| 160 | 17252635 | NA | Shpk | sedoheptulokinase | 74637 | ENSMUSG00000005951 |
| 161 | 17212813 | NA | Mstn | myostatin | 17700 | ENSMUSG00000026100 |
| 162 | 17230945 | NA | Smyd2 | SET and MYND domain containing 2 | 226830 | ENSMUSG00000026603 |
| 163 | 17313008 | NA | Cby1 | chibby homolog 1 (Drosophila) | 73739 | ENSMUSG00000022428 |
| 164 | 17521448 | NA | Hyal3 | hyaluronoglucosaminidase 3 | 109685 | ENSMUSG00000036091 |
| 165 | 17535434 | NA | Nsdhl | NAD(P) dependent steroid dehydrogenase-like | 18194 | ENSMUSG00000031349 |
| 166 | 17342359 | NA | Haghl | hydroxyacylglutathione hydrolase-like | 68977 | ENSMUSG00000061046 |
| 167 | 17468113 | NA | Ino80b | INO80 complex subunit B | 70020 | ENSMUSG00000030034 |
| 168 | 17347558 | NA | Cdkl4 | cyclin-dependent kinase-like 4 | 381113 | ENSMUSG00000033966 |
| 169 | 17503023 | NA | Asf1b | ASF1 anti-silencing function 1 homolog B (S. cerevisiae) | 66929 | ENSMUSG00000005470 |
| 170 | 17283939 | NA | Wars | tryptophanyl-tRNA synthetase | 22375 | ENSMUSG00000021266 |
| 171 | 17283941 | NA | Wars | tryptophanyl-tRNA synthetase | 22375 | ENSMUSG00000021266 |
| 172 | 17283930 | NA | Wars | tryptophanyl-tRNA synthetase | 22375 | ENSMUSG00000021266 |
| 173 | 17283938 | NA | Wars | tryptophanyl-tRNA synthetase | 22375 | ENSMUSG00000021266 |
| 174 | 17258457 | NA | Sap30bp | SAP30 binding protein | 57230 | ENSMUSG00000020755 |
| 175 | 17454256 | NA | Taf6 | TAF6 RNA polymerase II, TATA box binding protein (TBP)-associated factor | 21343 | ENSMUSG00000036980 |
| 176 | 17432967 | NA | Ubiad1 | UbiA prenyltransferase domain containing 1 | 71707 | ENSMUSG00000047719 |
| 177 | 17510365 | NA | Pgls | 6-phosphogluconolactonase | 66171 | ENSMUSG00000031807 |
| 178 | 17526273 | NA | Trappc4 | trafficking protein particle complex 4 | 60409 | ENSMUSG00000032112 |
| 179 | 17526271 | NA | Trappc4 | trafficking protein particle complex 4 | 60409 | ENSMUSG00000032112 |
| 180 | 17526272 | NA | Trappc4 | trafficking protein particle complex 4 | 60409 | ENSMUSG00000032112 |
| 181 | 17231118 | NA | Rcor3 | REST corepressor 3 | 214742 | ENSMUSG00000037395 |
| 182 | 17298407 | NA | Bap1 | Brca1 associated protein 1 | 104416 | ENSMUSG00000021901 |
| 183 | 17318877 | NA | Txn2 | thioredoxin 2 | 56551 | ENSMUSG00000005354 |
| 184 | 17265186 | NA | Rnasek | ribonuclease, RNase K | 52898 | ENSMUSG00000093989 |
| 185 | 17405174 | NA | Cog6 | component of oligomeric golgi complex 6 | 67542 | ENSMUSG00000027742 |
| 186 | 17221014 | NA | Cd34 | CD34 antigen | 12490 | ENSMUSG00000016494 |
| 187 | 17273280 | NA | Stra13 | stimulated by retinoic acid 13 | 20892 | ENSMUSG00000025144 |
| 188 | 17301342 | NA | Ints9 | integrator complex subunit 9 | 210925 | ENSMUSG00000021975 |
| 189 | 17241409 | NA | Srgn | serglycin | 19073 | ENSMUSG00000020077 |
| 190 | 17393357 | NA | Eif6 | eukaryotic translation initiation factor 6 | 16418 | ENSMUSG00000027613 |
| 191 | 17412593 | NA | Srsf12 | serine/arginine-rich splicing factor 12 | 272009 | ENSMUSG00000054679 |
| 192 | 17213990 | NA | Atic | 5-aminoimidazole-4-carboxamide ribonucleotide formyltransferase/IMP cyclohydrolase | 108147 | ENSMUSG00000026192 |
| 193 | 17504160 | NA | Polr2c | polymerase (RNA) II (DNA directed) polypeptide C | 20021 | ENSMUSG00000031783 |
| 194 | 17455093 | NA | Zkscan14 | zinc finger with KRAB and SCAN domains 14 | 67235 | ENSMUSG00000029627 |
| 195 | 17508691 | NA | Rbpms | RNA binding protein gene with multiple splicing | 19663 | ENSMUSG00000031586 |
| 196 | 17222001 | NA | Prim2 | DNA primase, p58 subunit | 19076 | ENSMUSG00000026134 |
| 197 | 17232649 | NA | Fyn | Fyn proto-oncogene | 14360 | ENSMUSG00000019843 |
| 198 | 17328810 | NA | Dgcr14 | DiGeorge syndrome critical region gene 14 | 27886 | ENSMUSG00000003527 |
| 199 | 17395844 | NA | Stmn3 | stathmin-like 3 | 20262 | ENSMUSG00000027581 |
| 200 | 17229466 | NA | Hsd17b7 | hydroxysteroid (17-beta) dehydrogenase 7 | 15490 | ENSMUSG00000026675 |
| 201 | 17219286 | NA | Dedd | death effector domain-containing | 21945 | ENSMUSG00000013973 |
| 202 | 17306861 | NA | Dhrs1 | dehydrogenase/reductase (SDR family) member 1 | 52585 | ENSMUSG00000002332 |
| 203 | 17306864 | NA | Dhrs1 | dehydrogenase/reductase (SDR family) member 1 | 52585 | ENSMUSG00000002332 |
| 204 | 17306860 | NA | Dhrs1 | dehydrogenase/reductase (SDR family) member 1 | 52585 | ENSMUSG00000002332 |
| 205 | 17306856 | NA | Dhrs1 | dehydrogenase/reductase (SDR family) member 1 | 52585 | ENSMUSG00000002332 |
| 206 | 17306865 | NA | Dhrs1 | dehydrogenase/reductase (SDR family) member 1 | 52585 | ENSMUSG00000002332 |
| 207 | 17479069 | NA | Lysmd4 | LysM, putative peptidoglycan-binding, domain containing 4 | 75099 | ENSMUSG00000043831 |
| 208 | 17241780 | NA | Ube2d1 | ubiquitin-conjugating enzyme E2D 1 | 216080 | ENSMUSG00000019927 |
| 209 | 17334495 | NA | Nme3 | NME/NM23 nucleoside diphosphate kinase 3 | 79059 | ENSMUSG00000073435 |
| 210 | 17383588 | NA | Ccbl1 | cysteine conjugate-beta lyase 1 | 70266 | ENSMUSG00000039648 |
| 211 | 17313199 | NA | Adsl | adenylosuccinate lyase | 11564 | ENSMUSG00000022407 |
| 212 | 17232215 | NA | Moxd1 | monooxygenase, DBH-like 1 | 59012 | ENSMUSG00000020000 |
| 213 | 17418447 | NA | Meaf6 | MYST/Esa1-associated factor 6 | 70088 | ENSMUSG00000028863 |
| 214 | 17356202 | NA | Pold4 | polymerase (DNA-directed), delta 4 | 69745 | ENSMUSG00000024854 |
| 215 | 17421972 | NA | Errfi1 | ERBB receptor feedback inhibitor 1 | 74155 | ENSMUSG00000028967 |
| 216 | 17330373 | NA | Adprh | ADP-ribosylarginine hydrolase | 11544 | ENSMUSG00000002844 |

  
  

| **Database:biological process      &nbspName:organic substance metabolic process      &nbspID:GO:0071704** | | | | | | |
| --- | --- | --- | --- | --- | --- | --- |
| C=7866; O=193; E=151.27; R=1.28; rawP=1.02e-05; adjP=0.0044 | | | | | | |
| Index | UserID | Value | Gene Symbol | Gene Name | EntrezGene | Ensembl |
| 1 | 17476273 | NA | Zfp382 | zinc finger protein 382 | 233060 | ENSMUSG00000074220 |
| 2 | 17232843 | NA | Zbtb24 | zinc finger and BTB domain containing 24 | 268294 | ENSMUSG00000019826 |
| 3 | 17344794 | NA | Znrd1 | zinc ribbon domain containing, 1 | 66136 | ENSMUSG00000036315 |
| 4 | 17300261 | NA | Oxa1l | oxidase assembly 1-like | 69089 | ENSMUSG00000000959 |
| 5 | 17512740 | NA | Nob1 | NIN1/RPN12 binding protein 1 homolog (S. cerevisiae) | 67619 | ENSMUSG00000003848 |
| 6 | 17224540 | NA | Tuba4a | tubulin, alpha 4A | 22145 | ENSMUSG00000026202 |
| 7 | 17368171 | NA | Bmyc | brain expressed myelocytomatosis oncogene | 107771 | ENSMUSG00000049086 |
| 8 | 17350134 | NA | Pou4f3 | POU domain, class 4, transcription factor 3 | 18998 | ENSMUSG00000024497 |
| 9 | 17517723 | NA | Rpp25 | ribonuclease P 25 subunit (human) | 102614 | ENSMUSG00000062309 |
| 10 | 17307134 | NA | Cryl1 | crystallin, lambda 1 | 68631 | ENSMUSG00000021947 |
| 11 | 17443181 | NA | Dnajc30 | DnaJ (Hsp40) homolog, subfamily C, member 30 | 66114 | ENSMUSG00000061118 |
| 12 | 17512103 | NA | Got2 | glutamate oxaloacetate transaminase 2, mitochondrial | 14719 | ENSMUSG00000031672 |
| 13 | 17369862 | NA | Dpm2 | dolichol-phosphate (beta-D) mannosyltransferase 2 | 13481 | ENSMUSG00000026810 |
| 14 | 17336829 | NA | Lsm2 | LSM2 homolog, U6 small nuclear RNA associated (S. cerevisiae) | 27756 | ENSMUSG00000007050 |
| 15 | 17288454 | NA | Irx4 | Iroquois related homeobox 4 (Drosophila) | 50916 | ENSMUSG00000021604 |
| 16 | 17467996 | NA | Mrpl19 | mitochondrial ribosomal protein L19 | 56284 | ENSMUSG00000030045 |
| 17 | 17322559 | NA | Hmox2 | heme oxygenase (decycling) 2 | 15369 | ENSMUSG00000004070 |
| 18 | 17404329 | NA | Gyg | glycogenin | 27357 | ENSMUSG00000019528 |
| 19 | 17321467 | NA | Tuba1b | tubulin, alpha 1B | 22143 | ENSMUSG00000023004 |
| 20 | 17288160 | NA | Cdk20 | cyclin-dependent kinase 20 | 105278 | ENSMUSG00000021483 |
| 21 | 17538096 | NA | Rnf128 | ring finger protein 128 | 66889 | ENSMUSG00000031438 |
| 22 | 17447099 | NA | Ctbp1 | C-terminal binding protein 1 | 13016 | ENSMUSG00000037373 |
| 23 | 17447089 | NA | Ctbp1 | C-terminal binding protein 1 | 13016 | ENSMUSG00000037373 |
| 24 | 17447100 | NA | Ctbp1 | C-terminal binding protein 1 | 13016 | ENSMUSG00000037373 |
| 25 | 17447098 | NA | Ctbp1 | C-terminal binding protein 1 | 13016 | ENSMUSG00000037373 |
| 26 | 17343617 | NA | Rab11b | RAB11B, member RAS oncogene family | 19326 | ENSMUSG00000077450 |
| 27 | 17379187 | NA | Ift52 | intraflagellar transport 52 | 245866 | ENSMUSG00000017858 |
| 28 | 17440361 | NA | Plcxd1 | phosphatidylinositol-specific phospholipase C, X domain containing 1 | 403178 | ENSMUSG00000064247 |
| 29 | 17437611 | NA | Pgm1 | phosphoglucomutase 1 | 66681 | ENSMUSG00000029171 |
| 30 | 17265082 | NA | Eif5a | eukaryotic translation initiation factor 5A | 276770 | ENSMUSG00000078812 |
| 31 | 17297750 | NA | Ppif | peptidylprolyl isomerase F (cyclophilin F) | 105675 | ENSMUSG00000021868 |
| 32 | 17233630 | NA | Psap | prosaposin | 19156 | ENSMUSG00000004207 |
| 33 | 17520177 | NA | Mthfs | 5, 10-methenyltetrahydrofolate synthetase | 107885 | ENSMUSG00000066442 |
| 34 | 17521143 | NA | Wdr82 | WD repeat domain containing 82 | 77305 | ENSMUSG00000020257 |
| 35 | 17528778 | NA | Ccpg1 | cell cycle progression 1 | 72278 | ENSMUSG00000034563 |
| 36 | 17278188 | NA | Otub2 | OTU domain, ubiquitin aldehyde binding 2 | 68149 | ENSMUSG00000021203 |
| 37 | 17225499 | NA | Hes6 | hairy and enhancer of split 6 (Drosophila) | 55927 | ENSMUSG00000067071 |
| 38 | 17312944 | NA | Polr2f | polymerase (RNA) II (DNA directed) polypeptide F | 69833 | ENSMUSG00000033020 |
| 39 | 17312939 | NA | Polr2f | polymerase (RNA) II (DNA directed) polypeptide F | 69833 | ENSMUSG00000033020 |
| 40 | 17312941 | NA | Polr2f | polymerase (RNA) II (DNA directed) polypeptide F | 69833 | ENSMUSG00000033020 |
| 41 | 17334722 | NA | Rpusd1 | RNA pseudouridylate synthase domain containing 1 | 106707 | ENSMUSG00000041199 |
| 42 | 17517576 | NA | Hmg20a | high mobility group 20A | 66867 | ENSMUSG00000032329 |
| 43 | 17393225 | NA | Pigu | phosphatidylinositol glycan anchor biosynthesis, class U | 228812 | ENSMUSG00000038383 |
| 44 | 17404011 | NA | Hey1 | hairy/enhancer-of-split related with YRPW motif 1 | 15213 | ENSMUSG00000040289 |
| 45 | 17400222 | NA | Vps72 | vacuolar protein sorting 72 (yeast) | 21427 | ENSMUSG00000008958 |
| 46 | 17499396 | NA | Fbxo25 | F-box protein 25 | 66822 | ENSMUSG00000038365 |
| 47 | 17246850 | NA | Zmat5 | zinc finger, matrin type 5 | 67178 | ENSMUSG00000009076 |
| 48 | 17324664 | NA | Dlg1 | discs, large homolog 1 (Drosophila) | 13383 | ENSMUSG00000022770 |
| 49 | 17396369 | NA | Nceh1 | arylacetamide deacetylase-like 1 | 320024 | ENSMUSG00000027698 |
| 50 | 17502191 | NA | Mrpl34 | mitochondrial ribosomal protein L34 | 94065 | ENSMUSG00000034880 |
| 51 | 17529231 | NA | Phip | pleckstrin homology domain interacting protein | 83946 | ENSMUSG00000032253 |
| 52 | 17316625 | NA | Ubr5 | ubiquitin protein ligase E3 component n-recognin 5 | 70790 | ENSMUSG00000037487 |
| 53 | 17277370 | NA | Eif2b2 | eukaryotic translation initiation factor 2B, subunit 2 beta | 217715 | ENSMUSG00000004788 |
| 54 | 17483220 | NA | Cdipt | CDP-diacylglycerol--inositol 3-phosphatidyltransferase (phosphatidylinositol synthase) | 52858 | ENSMUSG00000030682 |
| 55 | 17245709 | NA | Os9 | amplified in osteosarcoma | 216440 | ENSMUSG00000040462 |
| 56 | 17512009 | NA | Csnk2a2 | casein kinase 2, alpha prime polypeptide | 13000 | ENSMUSG00000046707 |
| 57 | 17424319 | NA | Sigmar1 | sigma non-opioid intracellular receptor 1 | 18391 | ENSMUSG00000036078 |
| 58 | 17503816 | NA | Irx6 | Iroquois related homeobox 6 (Drosophila) | 64379 | ENSMUSG00000031738 |
| 59 | 17282649 | NA | Rps6kl1 | ribosomal protein S6 kinase-like 1 | 238323 | ENSMUSG00000019235 |
| 60 | 17260221 | NA | Pold2 | polymerase (DNA directed), delta 2, regulatory subunit | 18972 | ENSMUSG00000020471 |
| 61 | 17365493 | NA | Obfc1 | oligonucleotide/oligosaccharide-binding fold containing 1 | 108689 | ENSMUSG00000042694 |
| 62 | 17538790 | NA | Huwe1 | HECT, UBA and WWE domain containing 1 | 59026 | ENSMUSG00000025261 |
| 63 | 17446580 | NA | Shh | sonic hedgehog | 20423 | ENSMUSG00000002633 |
| 64 | 17470879 | NA | Tpi1 | triosephosphate isomerase 1 | 21991 | ENSMUSG00000023456 |
| 65 | 17281971 | NA | Sgpp1 | sphingosine-1-phosphate phosphatase 1 | 81535 | ENSMUSG00000021054 |
| 66 | 17230408 | NA | Adck3 | aarF domain containing kinase 3 | 67426 | ENSMUSG00000026489 |
| 67 | 17238549 | NA | Wibg | within bgcn homolog (Drosophila) | 78428 | ENSMUSG00000064030 |
| 68 | 17212355 | NA | Nck2 | non-catalytic region of tyrosine kinase adaptor protein 2 | 17974 | ENSMUSG00000066877 |
| 69 | 17431502 | NA | Lypla2 | lysophospholipase 2 | 26394 | ENSMUSG00000028670 |
| 70 | 17303496 | NA | Fezf2 | Fez family zinc finger 2 | 54713 | ENSMUSG00000021743 |
| 71 | 17256565 | NA | Tubg2 | tubulin, gamma 2 | 103768 | ENSMUSG00000045007 |
| 72 | 17500275 | NA | Erlin2 | ER lipid raft associated 2 | 244373 | ENSMUSG00000031483 |
| 73 | 17345519 | NA | Rrp36 | ribosomal RNA processing 36 homolog (S. cerevisiae) | 224823 | ENSMUSG00000023971 |
| 74 | 17463422 | NA | Nrip2 | nuclear receptor interacting protein 2 | 60345 | ENSMUSG00000001520 |
| 75 | 17211335 | NA | Tfap2d | transcription factor AP-2, delta | 226896 | ENSMUSG00000042596 |
| 76 | 17506631 | NA | Tubb3 | tubulin, beta 3 class III | 22152 | ENSMUSG00000062380 |
| 77 | 17309825 | NA | Plcxd3 | phosphatidylinositol-specific phospholipase C, X domain containing 3 | 239318 | ENSMUSG00000049148 |
| 78 | 17321722 | NA | Tfcp2 | transcription factor CP2 | 21422 | ENSMUSG00000009733 |
| 79 | 17213153 | NA | Nif3l1 | Ngg1 interacting factor 3-like 1 (S. pombe) | 65102 | ENSMUSG00000026036 |
| 80 | 17235584 | NA | Dapk3 | death-associated protein kinase 3 | 13144 | ENSMUSG00000034974 |
| 81 | 17477670 | NA | Rras | Harvey rat sarcoma oncogene, subgroup R | 20130 | ENSMUSG00000038387 |
| 82 | 17405819 | NA | B3galnt1 | UDP-GalNAc:betaGlcNAc beta 1,3-galactosaminyltransferase, polypeptide 1 | 26879 | ENSMUSG00000043300 |
| 83 | 17300411 | NA | Thtpa | thiamine triphosphatase | 105663 | ENSMUSG00000045691 |
| 84 | 17498821 | NA | Snapc2 | small nuclear RNA activating complex, polypeptide 2 | 102209 | ENSMUSG00000011837 |
| 85 | 17460879 | NA | Hdac11 | histone deacetylase 11 | 232232 | ENSMUSG00000034245 |
| 86 | 17256618 | NA | Vps25 | vacuolar protein sorting 25 (yeast) | 28084 | ENSMUSG00000078656 |
| 87 | 17444100 | NA | Chst12 | carbohydrate sulfotransferase 12 | 59031 | ENSMUSG00000036599 |
| 88 | 17517532 | NA | Isl2 | insulin related protein 2 (islet 2) | 104360 | ENSMUSG00000032318 |
| 89 | 17494637 | NA | Mrpl17 | mitochondrial ribosomal protein L17 | 27397 | ENSMUSG00000030879 |
| 90 | 17513871 | NA | Chmp1a | charged multivesicular body protein 1A | 234852 | ENSMUSG00000000743 |
| 91 | 17359994 | NA | Fbxl15 | F-box and leucine-rich repeat protein 15 | 68431 | ENSMUSG00000025226 |
| 92 | 17497366 | NA | Ebf3 | early B cell factor 3 | 13593 | ENSMUSG00000010476 |
| 93 | 17446322 | NA | Prkag2 | protein kinase, AMP-activated, gamma 2 non-catalytic subunit | 108099 | ENSMUSG00000028944 |
| 94 | 17524523 | NA | Eif3g | eukaryotic translation initiation factor 3, subunit G | 53356 | ENSMUSG00000070319 |
| 95 | 17306835 | NA | Rabggta | Rab geranylgeranyl transferase, a subunit | 56187 | ENSMUSG00000040472 |
| 96 | 17269638 | NA | Rab5c | RAB5C, member RAS oncogene family | 19345 | ENSMUSG00000019173 |
| 97 | 17505367 | NA | Txnl4b | thioredoxin-like 4B | 234723 | ENSMUSG00000031723 |
| 98 | 17256549 | NA | Tubg1 | tubulin, gamma 1 | 103733 | ENSMUSG00000035198 |
| 99 | 17498370 | NA | Nadsyn1 | NAD synthetase 1 | 78914 | ENSMUSG00000031090 |
| 100 | 17494081 | NA | Rhog | ras homolog gene family, member G | 56212 | ENSMUSG00000073982 |
| 101 | 17497957 | NA | Chid1 | chitinase domain containing 1 | 68038 | ENSMUSG00000025512 |
| 102 | 17532045 | NA | Plcd1 | phospholipase C, delta 1 | 18799 | ENSMUSG00000010660 |
| 103 | 17274448 | NA | Cpsf3 | cleavage and polyadenylation specificity factor 3 | 54451 | ENSMUSG00000054309 |
| 104 | 17422117 | NA | Acot7 | acyl-CoA thioesterase 7 | 70025 | ENSMUSG00000028937 |
| 105 | 17519868 | NA | Htr1b | 5-hydroxytryptamine (serotonin) receptor 1B | 15551 | ENSMUSG00000049511 |
| 106 | 17536264 | NA | Pcyt1b | phosphate cytidylyltransferase 1, choline, beta isoform | 236899 | ENSMUSG00000035246 |
| 107 | 17515170 | NA | Ilf3 | interleukin enhancer binding factor 3 | 16201 | ENSMUSG00000032178 |
| 108 | 17357213 | NA | Zbtb3 | zinc finger and BTB domain containing 3 | 75291 | ENSMUSG00000071661 |
| 109 | 17430853 | NA | Med18 | mediator of RNA polymerase II transcription, subunit 18 homolog (yeast) | 67219 | ENSMUSG00000066042 |
| 110 | 17225179 | NA | Pde6d | phosphodiesterase 6D, cGMP-specific, rod, delta | 18582 | ENSMUSG00000026239 |
| 111 | 17521014 | NA | Acad11 | acyl-Coenzyme A dehydrogenase family, member 11 | 102632 | ENSMUSG00000090150 |
| 112 | 17370285 | NA | Mrrf | mitochondrial ribosome recycling factor | 67871 | ENSMUSG00000026887 |
| 113 | 17361988 | NA | Arl2 | ADP-ribosylation factor-like 2 | 56327 | ENSMUSG00000024944 |
| 114 | 17229948 | NA | Dusp23 | dual specificity phosphatase 23 | 68440 | ENSMUSG00000026544 |
| 115 | 17502583 | NA | Mcm5 | minichromosome maintenance deficient 5, cell division cycle 46 (S. cerevisiae) | 17218 | ENSMUSG00000005410 |
| 116 | 17511296 | NA | Wdr83 | WD repeat domain containing 83 | 67836 | ENSMUSG00000005150 |
| 117 | 17539434 | NA | Ctps2 | cytidine 5'-triphosphate synthase 2 | 55936 | ENSMUSG00000031360 |
| 118 | 17341521 | NA | Thoc6 | THO complex 6 homolog (Drosophila) | 386612 | ENSMUSG00000041319 |
| 119 | 17250141 | NA | Zfp39 | zinc finger protein 39 | 22698 | ENSMUSG00000037001 |
| 120 | 17519718 | NA | Mto1 | mitochondrial translation optimization 1 homolog (S. cerevisiae) | 68291 | ENSMUSG00000032342 |
| 121 | 17521422 | NA | Hyal2 | hyaluronoglucosaminidase 2 | 15587 | ENSMUSG00000010047 |
| 122 | 17317208 | NA | Derl1 | Der1-like domain family, member 1 | 67819 | ENSMUSG00000022365 |
| 123 | 17501544 | NA | Npy1r | neuropeptide Y receptor Y1 | 18166 | ENSMUSG00000036437 |
| 124 | 17235714 | NA | Dohh | deoxyhypusine hydroxylase/monooxygenase | 102115 | ENSMUSG00000078440 |
| 125 | 17540589 | NA | Klhl13 | kelch-like 13 (Drosophila) | 67455 | ENSMUSG00000036782 |
| 126 | 17428858 | NA | Rnf220 | ring finger protein 220 | 66743 | ENSMUSG00000028677 |
| 127 | 17428857 | NA | Rnf220 | ring finger protein 220 | 66743 | ENSMUSG00000028677 |
| 128 | 17468195 | NA | Stambp | STAM binding protein | 70527 | ENSMUSG00000006906 |
| 129 | 17408684 | NA | Dclre1b | DNA cross-link repair 1B, PSO2 homolog (S. cerevisiae) | 140917 | ENSMUSG00000027845 |
| 130 | 17436077 | NA | Nrbp1 | nuclear receptor binding protein 1 | 192292 | ENSMUSG00000029148 |
| 131 | 17342015 | NA | Tbl3 | transducin (beta)-like 3 | 213773 | ENSMUSG00000040688 |
| 132 | 17252170 | NA | Rnf167 | ring finger protein 167 | 70510 | ENSMUSG00000040746 |
| 133 | 17543988 | NA | Taf9b | TAF9B RNA polymerase II, TATA box binding protein (TBP)-associated factor | 407786 | ENSMUSG00000047242 |
| 134 | 17509721 | NA | Tufm | Tu translation elongation factor, mitochondrial | 233870 | ENSMUSG00000073838 |
| 135 | 17459676 | NA | Retsat | retinol saturase (all trans retinol 13,14 reductase) | 67442 | ENSMUSG00000056666 |
| 136 | 17279858 | NA | Fkbp1b | FK506 binding protein 1b | 14226 | ENSMUSG00000020635 |
| 137 | 17488463 | NA | Med29 | mediator complex subunit 29 | 67224 | ENSMUSG00000003444 |
| 138 | 17391270 | NA | Kcnip3 | Kv channel interacting protein 3, calsenilin | 56461 | ENSMUSG00000079056 |
| 139 | 17324576 | NA | Hrasls | HRAS-like suppressor | 27281 | ENSMUSG00000022525 |
| 140 | 17531834 | NA | Fbxl2 | F-box and leucine-rich repeat protein 2 | 72179 | ENSMUSG00000032507 |
| 141 | 17460099 | NA | Vax2 | ventral anterior homeobox containing gene 2 | 24113 | ENSMUSG00000034777 |
| 142 | 17358777 | NA | Stambpl1 | STAM binding protein like 1 | 76630 | ENSMUSG00000024776 |
| 143 | 17257060 | NA | Nmt1 | N-myristoyltransferase 1 | 18107 | ENSMUSG00000020936 |
| 144 | 17292107 | NA | Tbc1d7 | TBC1 domain family, member 7 | 67046 | ENSMUSG00000021368 |
| 145 | 17498239 | NA | Kcnq1ot1 | KCNQ1 overlapping transcript 1 | 63830 | NULL |
| 146 | 17512479 | NA | Acd | adrenocortical dysplasia | 497652 | ENSMUSG00000038000 |
| 147 | 17540050 | NA | Ebp | phenylalkylamine Ca2+ antagonist (emopamil) binding protein | 13595 | ENSMUSG00000031168 |
| 148 | 17429495 | NA | Nfyc | nuclear transcription factor-Y gamma | 18046 | ENSMUSG00000032897 |
| 149 | 17361855 | NA | Pola2 | polymerase (DNA directed), alpha 2 | 18969 | ENSMUSG00000024833 |
| 150 | 17527532 | NA | Mpi | mannose phosphate isomerase | 110119 | ENSMUSG00000032306 |
| 151 | 17353639 | NA | Dnajc18 | DnaJ (Hsp40) homolog, subfamily C, member 18 | 76594 | ENSMUSG00000024350 |
| 152 | 17512463 | NA | Atp6v0d1 | ATPase, H+ transporting, lysosomal V0 subunit D1 | 11972 | ENSMUSG00000013160 |
| 153 | 17512466 | NA | Atp6v0d1 | ATPase, H+ transporting, lysosomal V0 subunit D1 | 11972 | ENSMUSG00000013160 |
| 154 | 17252635 | NA | Shpk | sedoheptulokinase | 74637 | ENSMUSG00000005951 |
| 155 | 17212813 | NA | Mstn | myostatin | 17700 | ENSMUSG00000026100 |
| 156 | 17230945 | NA | Smyd2 | SET and MYND domain containing 2 | 226830 | ENSMUSG00000026603 |
| 157 | 17313008 | NA | Cby1 | chibby homolog 1 (Drosophila) | 73739 | ENSMUSG00000022428 |
| 158 | 17521448 | NA | Hyal3 | hyaluronoglucosaminidase 3 | 109685 | ENSMUSG00000036091 |
| 159 | 17535434 | NA | Nsdhl | NAD(P) dependent steroid dehydrogenase-like | 18194 | ENSMUSG00000031349 |
| 160 | 17342359 | NA | Haghl | hydroxyacylglutathione hydrolase-like | 68977 | ENSMUSG00000061046 |
| 161 | 17468113 | NA | Ino80b | INO80 complex subunit B | 70020 | ENSMUSG00000030034 |
| 162 | 17347558 | NA | Cdkl4 | cyclin-dependent kinase-like 4 | 381113 | ENSMUSG00000033966 |
| 163 | 17503023 | NA | Asf1b | ASF1 anti-silencing function 1 homolog B (S. cerevisiae) | 66929 | ENSMUSG00000005470 |
| 164 | 17283939 | NA | Wars | tryptophanyl-tRNA synthetase | 22375 | ENSMUSG00000021266 |
| 165 | 17283941 | NA | Wars | tryptophanyl-tRNA synthetase | 22375 | ENSMUSG00000021266 |
| 166 | 17283930 | NA | Wars | tryptophanyl-tRNA synthetase | 22375 | ENSMUSG00000021266 |
| 167 | 17283938 | NA | Wars | tryptophanyl-tRNA synthetase | 22375 | ENSMUSG00000021266 |
| 168 | 17258457 | NA | Sap30bp | SAP30 binding protein | 57230 | ENSMUSG00000020755 |
| 169 | 17454256 | NA | Taf6 | TAF6 RNA polymerase II, TATA box binding protein (TBP)-associated factor | 21343 | ENSMUSG00000036980 |
| 170 | 17432967 | NA | Ubiad1 | UbiA prenyltransferase domain containing 1 | 71707 | ENSMUSG00000047719 |
| 171 | 17510365 | NA | Pgls | 6-phosphogluconolactonase | 66171 | ENSMUSG00000031807 |
| 172 | 17526273 | NA | Trappc4 | trafficking protein particle complex 4 | 60409 | ENSMUSG00000032112 |
| 173 | 17526271 | NA | Trappc4 | trafficking protein particle complex 4 | 60409 | ENSMUSG00000032112 |
| 174 | 17526272 | NA | Trappc4 | trafficking protein particle complex 4 | 60409 | ENSMUSG00000032112 |
| 175 | 17231118 | NA | Rcor3 | REST corepressor 3 | 214742 | ENSMUSG00000037395 |
| 176 | 17298407 | NA | Bap1 | Brca1 associated protein 1 | 104416 | ENSMUSG00000021901 |
| 177 | 17318877 | NA | Txn2 | thioredoxin 2 | 56551 | ENSMUSG00000005354 |
| 178 | 17265186 | NA | Rnasek | ribonuclease, RNase K | 52898 | ENSMUSG00000093989 |
| 179 | 17405174 | NA | Cog6 | component of oligomeric golgi complex 6 | 67542 | ENSMUSG00000027742 |
| 180 | 17221014 | NA | Cd34 | CD34 antigen | 12490 | ENSMUSG00000016494 |
| 181 | 17273280 | NA | Stra13 | stimulated by retinoic acid 13 | 20892 | ENSMUSG00000025144 |
| 182 | 17301342 | NA | Ints9 | integrator complex subunit 9 | 210925 | ENSMUSG00000021975 |
| 183 | 17241409 | NA | Srgn | serglycin | 19073 | ENSMUSG00000020077 |
| 184 | 17393357 | NA | Eif6 | eukaryotic translation initiation factor 6 | 16418 | ENSMUSG00000027613 |
| 185 | 17412593 | NA | Srsf12 | serine/arginine-rich splicing factor 12 | 272009 | ENSMUSG00000054679 |
| 186 | 17213990 | NA | Atic | 5-aminoimidazole-4-carboxamide ribonucleotide formyltransferase/IMP cyclohydrolase | 108147 | ENSMUSG00000026192 |
| 187 | 17504160 | NA | Polr2c | polymerase (RNA) II (DNA directed) polypeptide C | 20021 | ENSMUSG00000031783 |
| 188 | 17455093 | NA | Zkscan14 | zinc finger with KRAB and SCAN domains 14 | 67235 | ENSMUSG00000029627 |
| 189 | 17508691 | NA | Rbpms | RNA binding protein gene with multiple splicing | 19663 | ENSMUSG00000031586 |
| 190 | 17222001 | NA | Prim2 | DNA primase, p58 subunit | 19076 | ENSMUSG00000026134 |
| 191 | 17232649 | NA | Fyn | Fyn proto-oncogene | 14360 | ENSMUSG00000019843 |
| 192 | 17328810 | NA | Dgcr14 | DiGeorge syndrome critical region gene 14 | 27886 | ENSMUSG00000003527 |
| 193 | 17395844 | NA | Stmn3 | stathmin-like 3 | 20262 | ENSMUSG00000027581 |
| 194 | 17229466 | NA | Hsd17b7 | hydroxysteroid (17-beta) dehydrogenase 7 | 15490 | ENSMUSG00000026675 |
| 195 | 17219286 | NA | Dedd | death effector domain-containing | 21945 | ENSMUSG00000013973 |
| 196 | 17479069 | NA | Lysmd4 | LysM, putative peptidoglycan-binding, domain containing 4 | 75099 | ENSMUSG00000043831 |
| 197 | 17241780 | NA | Ube2d1 | ubiquitin-conjugating enzyme E2D 1 | 216080 | ENSMUSG00000019927 |
| 198 | 17334495 | NA | Nme3 | NME/NM23 nucleoside diphosphate kinase 3 | 79059 | ENSMUSG00000073435 |
| 199 | 17383588 | NA | Ccbl1 | cysteine conjugate-beta lyase 1 | 70266 | ENSMUSG00000039648 |
| 200 | 17313199 | NA | Adsl | adenylosuccinate lyase | 11564 | ENSMUSG00000022407 |
| 201 | 17232215 | NA | Moxd1 | monooxygenase, DBH-like 1 | 59012 | ENSMUSG00000020000 |
| 202 | 17418447 | NA | Meaf6 | MYST/Esa1-associated factor 6 | 70088 | ENSMUSG00000028863 |
| 203 | 17356202 | NA | Pold4 | polymerase (DNA-directed), delta 4 | 69745 | ENSMUSG00000024854 |
| 204 | 17421972 | NA | Errfi1 | ERBB receptor feedback inhibitor 1 | 74155 | ENSMUSG00000028967 |
| 205 | 17330373 | NA | Adprh | ADP-ribosylarginine hydrolase | 11544 | ENSMUSG00000002844 |

  
  

| **Database:biological process      &nbspName:primary metabolic process      &nbspID:GO:0044238** | | | | | | |
| --- | --- | --- | --- | --- | --- | --- |
| C=7451; O=183; E=143.29; R=1.28; rawP=2.18e-05; adjP=0.0070 | | | | | | |
| Index | UserID | Value | Gene Symbol | Gene Name | EntrezGene | Ensembl |
| 1 | 17476273 | NA | Zfp382 | zinc finger protein 382 | 233060 | ENSMUSG00000074220 |
| 2 | 17232843 | NA | Zbtb24 | zinc finger and BTB domain containing 24 | 268294 | ENSMUSG00000019826 |
| 3 | 17344794 | NA | Znrd1 | zinc ribbon domain containing, 1 | 66136 | ENSMUSG00000036315 |
| 4 | 17300261 | NA | Oxa1l | oxidase assembly 1-like | 69089 | ENSMUSG00000000959 |
| 5 | 17512740 | NA | Nob1 | NIN1/RPN12 binding protein 1 homolog (S. cerevisiae) | 67619 | ENSMUSG00000003848 |
| 6 | 17224540 | NA | Tuba4a | tubulin, alpha 4A | 22145 | ENSMUSG00000026202 |
| 7 | 17368171 | NA | Bmyc | brain expressed myelocytomatosis oncogene | 107771 | ENSMUSG00000049086 |
| 8 | 17350134 | NA | Pou4f3 | POU domain, class 4, transcription factor 3 | 18998 | ENSMUSG00000024497 |
| 9 | 17517723 | NA | Rpp25 | ribonuclease P 25 subunit (human) | 102614 | ENSMUSG00000062309 |
| 10 | 17307134 | NA | Cryl1 | crystallin, lambda 1 | 68631 | ENSMUSG00000021947 |
| 11 | 17443181 | NA | Dnajc30 | DnaJ (Hsp40) homolog, subfamily C, member 30 | 66114 | ENSMUSG00000061118 |
| 12 | 17512103 | NA | Got2 | glutamate oxaloacetate transaminase 2, mitochondrial | 14719 | ENSMUSG00000031672 |
| 13 | 17369862 | NA | Dpm2 | dolichol-phosphate (beta-D) mannosyltransferase 2 | 13481 | ENSMUSG00000026810 |
| 14 | 17336829 | NA | Lsm2 | LSM2 homolog, U6 small nuclear RNA associated (S. cerevisiae) | 27756 | ENSMUSG00000007050 |
| 15 | 17288454 | NA | Irx4 | Iroquois related homeobox 4 (Drosophila) | 50916 | ENSMUSG00000021604 |
| 16 | 17467996 | NA | Mrpl19 | mitochondrial ribosomal protein L19 | 56284 | ENSMUSG00000030045 |
| 17 | 17404329 | NA | Gyg | glycogenin | 27357 | ENSMUSG00000019528 |
| 18 | 17321467 | NA | Tuba1b | tubulin, alpha 1B | 22143 | ENSMUSG00000023004 |
| 19 | 17288160 | NA | Cdk20 | cyclin-dependent kinase 20 | 105278 | ENSMUSG00000021483 |
| 20 | 17538096 | NA | Rnf128 | ring finger protein 128 | 66889 | ENSMUSG00000031438 |
| 21 | 17447099 | NA | Ctbp1 | C-terminal binding protein 1 | 13016 | ENSMUSG00000037373 |
| 22 | 17447089 | NA | Ctbp1 | C-terminal binding protein 1 | 13016 | ENSMUSG00000037373 |
| 23 | 17447100 | NA | Ctbp1 | C-terminal binding protein 1 | 13016 | ENSMUSG00000037373 |
| 24 | 17447098 | NA | Ctbp1 | C-terminal binding protein 1 | 13016 | ENSMUSG00000037373 |
| 25 | 17343617 | NA | Rab11b | RAB11B, member RAS oncogene family | 19326 | ENSMUSG00000077450 |
| 26 | 17379187 | NA | Ift52 | intraflagellar transport 52 | 245866 | ENSMUSG00000017858 |
| 27 | 17440361 | NA | Plcxd1 | phosphatidylinositol-specific phospholipase C, X domain containing 1 | 403178 | ENSMUSG00000064247 |
| 28 | 17437611 | NA | Pgm1 | phosphoglucomutase 1 | 66681 | ENSMUSG00000029171 |
| 29 | 17265082 | NA | Eif5a | eukaryotic translation initiation factor 5A | 276770 | ENSMUSG00000078812 |
| 30 | 17297750 | NA | Ppif | peptidylprolyl isomerase F (cyclophilin F) | 105675 | ENSMUSG00000021868 |
| 31 | 17233630 | NA | Psap | prosaposin | 19156 | ENSMUSG00000004207 |
| 32 | 17521143 | NA | Wdr82 | WD repeat domain containing 82 | 77305 | ENSMUSG00000020257 |
| 33 | 17528778 | NA | Ccpg1 | cell cycle progression 1 | 72278 | ENSMUSG00000034563 |
| 34 | 17278188 | NA | Otub2 | OTU domain, ubiquitin aldehyde binding 2 | 68149 | ENSMUSG00000021203 |
| 35 | 17225499 | NA | Hes6 | hairy and enhancer of split 6 (Drosophila) | 55927 | ENSMUSG00000067071 |
| 36 | 17312944 | NA | Polr2f | polymerase (RNA) II (DNA directed) polypeptide F | 69833 | ENSMUSG00000033020 |
| 37 | 17312939 | NA | Polr2f | polymerase (RNA) II (DNA directed) polypeptide F | 69833 | ENSMUSG00000033020 |
| 38 | 17312941 | NA | Polr2f | polymerase (RNA) II (DNA directed) polypeptide F | 69833 | ENSMUSG00000033020 |
| 39 | 17334722 | NA | Rpusd1 | RNA pseudouridylate synthase domain containing 1 | 106707 | ENSMUSG00000041199 |
| 40 | 17517576 | NA | Hmg20a | high mobility group 20A | 66867 | ENSMUSG00000032329 |
| 41 | 17393225 | NA | Pigu | phosphatidylinositol glycan anchor biosynthesis, class U | 228812 | ENSMUSG00000038383 |
| 42 | 17404011 | NA | Hey1 | hairy/enhancer-of-split related with YRPW motif 1 | 15213 | ENSMUSG00000040289 |
| 43 | 17400222 | NA | Vps72 | vacuolar protein sorting 72 (yeast) | 21427 | ENSMUSG00000008958 |
| 44 | 17499396 | NA | Fbxo25 | F-box protein 25 | 66822 | ENSMUSG00000038365 |
| 45 | 17246850 | NA | Zmat5 | zinc finger, matrin type 5 | 67178 | ENSMUSG00000009076 |
| 46 | 17324664 | NA | Dlg1 | discs, large homolog 1 (Drosophila) | 13383 | ENSMUSG00000022770 |
| 47 | 17396369 | NA | Nceh1 | arylacetamide deacetylase-like 1 | 320024 | ENSMUSG00000027698 |
| 48 | 17502191 | NA | Mrpl34 | mitochondrial ribosomal protein L34 | 94065 | ENSMUSG00000034880 |
| 49 | 17529231 | NA | Phip | pleckstrin homology domain interacting protein | 83946 | ENSMUSG00000032253 |
| 50 | 17316625 | NA | Ubr5 | ubiquitin protein ligase E3 component n-recognin 5 | 70790 | ENSMUSG00000037487 |
| 51 | 17277370 | NA | Eif2b2 | eukaryotic translation initiation factor 2B, subunit 2 beta | 217715 | ENSMUSG00000004788 |
| 52 | 17483220 | NA | Cdipt | CDP-diacylglycerol--inositol 3-phosphatidyltransferase (phosphatidylinositol synthase) | 52858 | ENSMUSG00000030682 |
| 53 | 17245709 | NA | Os9 | amplified in osteosarcoma | 216440 | ENSMUSG00000040462 |
| 54 | 17512009 | NA | Csnk2a2 | casein kinase 2, alpha prime polypeptide | 13000 | ENSMUSG00000046707 |
| 55 | 17424319 | NA | Sigmar1 | sigma non-opioid intracellular receptor 1 | 18391 | ENSMUSG00000036078 |
| 56 | 17503816 | NA | Irx6 | Iroquois related homeobox 6 (Drosophila) | 64379 | ENSMUSG00000031738 |
| 57 | 17282649 | NA | Rps6kl1 | ribosomal protein S6 kinase-like 1 | 238323 | ENSMUSG00000019235 |
| 58 | 17260221 | NA | Pold2 | polymerase (DNA directed), delta 2, regulatory subunit | 18972 | ENSMUSG00000020471 |
| 59 | 17365493 | NA | Obfc1 | oligonucleotide/oligosaccharide-binding fold containing 1 | 108689 | ENSMUSG00000042694 |
| 60 | 17538790 | NA | Huwe1 | HECT, UBA and WWE domain containing 1 | 59026 | ENSMUSG00000025261 |
| 61 | 17446580 | NA | Shh | sonic hedgehog | 20423 | ENSMUSG00000002633 |
| 62 | 17470879 | NA | Tpi1 | triosephosphate isomerase 1 | 21991 | ENSMUSG00000023456 |
| 63 | 17281971 | NA | Sgpp1 | sphingosine-1-phosphate phosphatase 1 | 81535 | ENSMUSG00000021054 |
| 64 | 17230408 | NA | Adck3 | aarF domain containing kinase 3 | 67426 | ENSMUSG00000026489 |
| 65 | 17238549 | NA | Wibg | within bgcn homolog (Drosophila) | 78428 | ENSMUSG00000064030 |
| 66 | 17212355 | NA | Nck2 | non-catalytic region of tyrosine kinase adaptor protein 2 | 17974 | ENSMUSG00000066877 |
| 67 | 17431502 | NA | Lypla2 | lysophospholipase 2 | 26394 | ENSMUSG00000028670 |
| 68 | 17303496 | NA | Fezf2 | Fez family zinc finger 2 | 54713 | ENSMUSG00000021743 |
| 69 | 17256565 | NA | Tubg2 | tubulin, gamma 2 | 103768 | ENSMUSG00000045007 |
| 70 | 17500275 | NA | Erlin2 | ER lipid raft associated 2 | 244373 | ENSMUSG00000031483 |
| 71 | 17345519 | NA | Rrp36 | ribosomal RNA processing 36 homolog (S. cerevisiae) | 224823 | ENSMUSG00000023971 |
| 72 | 17463422 | NA | Nrip2 | nuclear receptor interacting protein 2 | 60345 | ENSMUSG00000001520 |
| 73 | 17211335 | NA | Tfap2d | transcription factor AP-2, delta | 226896 | ENSMUSG00000042596 |
| 74 | 17506631 | NA | Tubb3 | tubulin, beta 3 class III | 22152 | ENSMUSG00000062380 |
| 75 | 17309825 | NA | Plcxd3 | phosphatidylinositol-specific phospholipase C, X domain containing 3 | 239318 | ENSMUSG00000049148 |
| 76 | 17321722 | NA | Tfcp2 | transcription factor CP2 | 21422 | ENSMUSG00000009733 |
| 77 | 17213153 | NA | Nif3l1 | Ngg1 interacting factor 3-like 1 (S. pombe) | 65102 | ENSMUSG00000026036 |
| 78 | 17235584 | NA | Dapk3 | death-associated protein kinase 3 | 13144 | ENSMUSG00000034974 |
| 79 | 17477670 | NA | Rras | Harvey rat sarcoma oncogene, subgroup R | 20130 | ENSMUSG00000038387 |
| 80 | 17405819 | NA | B3galnt1 | UDP-GalNAc:betaGlcNAc beta 1,3-galactosaminyltransferase, polypeptide 1 | 26879 | ENSMUSG00000043300 |
| 81 | 17498821 | NA | Snapc2 | small nuclear RNA activating complex, polypeptide 2 | 102209 | ENSMUSG00000011837 |
| 82 | 17460879 | NA | Hdac11 | histone deacetylase 11 | 232232 | ENSMUSG00000034245 |
| 83 | 17256618 | NA | Vps25 | vacuolar protein sorting 25 (yeast) | 28084 | ENSMUSG00000078656 |
| 84 | 17444100 | NA | Chst12 | carbohydrate sulfotransferase 12 | 59031 | ENSMUSG00000036599 |
| 85 | 17517532 | NA | Isl2 | insulin related protein 2 (islet 2) | 104360 | ENSMUSG00000032318 |
| 86 | 17494637 | NA | Mrpl17 | mitochondrial ribosomal protein L17 | 27397 | ENSMUSG00000030879 |
| 87 | 17513871 | NA | Chmp1a | charged multivesicular body protein 1A | 234852 | ENSMUSG00000000743 |
| 88 | 17359994 | NA | Fbxl15 | F-box and leucine-rich repeat protein 15 | 68431 | ENSMUSG00000025226 |
| 89 | 17497366 | NA | Ebf3 | early B cell factor 3 | 13593 | ENSMUSG00000010476 |
| 90 | 17446322 | NA | Prkag2 | protein kinase, AMP-activated, gamma 2 non-catalytic subunit | 108099 | ENSMUSG00000028944 |
| 91 | 17524523 | NA | Eif3g | eukaryotic translation initiation factor 3, subunit G | 53356 | ENSMUSG00000070319 |
| 92 | 17306835 | NA | Rabggta | Rab geranylgeranyl transferase, a subunit | 56187 | ENSMUSG00000040472 |
| 93 | 17269638 | NA | Rab5c | RAB5C, member RAS oncogene family | 19345 | ENSMUSG00000019173 |
| 94 | 17505367 | NA | Txnl4b | thioredoxin-like 4B | 234723 | ENSMUSG00000031723 |
| 95 | 17256549 | NA | Tubg1 | tubulin, gamma 1 | 103733 | ENSMUSG00000035198 |
| 96 | 17498370 | NA | Nadsyn1 | NAD synthetase 1 | 78914 | ENSMUSG00000031090 |
| 97 | 17494081 | NA | Rhog | ras homolog gene family, member G | 56212 | ENSMUSG00000073982 |
| 98 | 17497957 | NA | Chid1 | chitinase domain containing 1 | 68038 | ENSMUSG00000025512 |
| 99 | 17532045 | NA | Plcd1 | phospholipase C, delta 1 | 18799 | ENSMUSG00000010660 |
| 100 | 17274448 | NA | Cpsf3 | cleavage and polyadenylation specificity factor 3 | 54451 | ENSMUSG00000054309 |
| 101 | 17422117 | NA | Acot7 | acyl-CoA thioesterase 7 | 70025 | ENSMUSG00000028937 |
| 102 | 17519868 | NA | Htr1b | 5-hydroxytryptamine (serotonin) receptor 1B | 15551 | ENSMUSG00000049511 |
| 103 | 17536264 | NA | Pcyt1b | phosphate cytidylyltransferase 1, choline, beta isoform | 236899 | ENSMUSG00000035246 |
| 104 | 17515170 | NA | Ilf3 | interleukin enhancer binding factor 3 | 16201 | ENSMUSG00000032178 |
| 105 | 17357213 | NA | Zbtb3 | zinc finger and BTB domain containing 3 | 75291 | ENSMUSG00000071661 |
| 106 | 17430853 | NA | Med18 | mediator of RNA polymerase II transcription, subunit 18 homolog (yeast) | 67219 | ENSMUSG00000066042 |
| 107 | 17225179 | NA | Pde6d | phosphodiesterase 6D, cGMP-specific, rod, delta | 18582 | ENSMUSG00000026239 |
| 108 | 17521014 | NA | Acad11 | acyl-Coenzyme A dehydrogenase family, member 11 | 102632 | ENSMUSG00000090150 |
| 109 | 17370285 | NA | Mrrf | mitochondrial ribosome recycling factor | 67871 | ENSMUSG00000026887 |
| 110 | 17361988 | NA | Arl2 | ADP-ribosylation factor-like 2 | 56327 | ENSMUSG00000024944 |
| 111 | 17229948 | NA | Dusp23 | dual specificity phosphatase 23 | 68440 | ENSMUSG00000026544 |
| 112 | 17502583 | NA | Mcm5 | minichromosome maintenance deficient 5, cell division cycle 46 (S. cerevisiae) | 17218 | ENSMUSG00000005410 |
| 113 | 17511296 | NA | Wdr83 | WD repeat domain containing 83 | 67836 | ENSMUSG00000005150 |
| 114 | 17539434 | NA | Ctps2 | cytidine 5'-triphosphate synthase 2 | 55936 | ENSMUSG00000031360 |
| 115 | 17341521 | NA | Thoc6 | THO complex 6 homolog (Drosophila) | 386612 | ENSMUSG00000041319 |
| 116 | 17250141 | NA | Zfp39 | zinc finger protein 39 | 22698 | ENSMUSG00000037001 |
| 117 | 17519718 | NA | Mto1 | mitochondrial translation optimization 1 homolog (S. cerevisiae) | 68291 | ENSMUSG00000032342 |
| 118 | 17521422 | NA | Hyal2 | hyaluronoglucosaminidase 2 | 15587 | ENSMUSG00000010047 |
| 119 | 17317208 | NA | Derl1 | Der1-like domain family, member 1 | 67819 | ENSMUSG00000022365 |
| 120 | 17501544 | NA | Npy1r | neuropeptide Y receptor Y1 | 18166 | ENSMUSG00000036437 |
| 121 | 17235714 | NA | Dohh | deoxyhypusine hydroxylase/monooxygenase | 102115 | ENSMUSG00000078440 |
| 122 | 17540589 | NA | Klhl13 | kelch-like 13 (Drosophila) | 67455 | ENSMUSG00000036782 |
| 123 | 17428858 | NA | Rnf220 | ring finger protein 220 | 66743 | ENSMUSG00000028677 |
| 124 | 17428857 | NA | Rnf220 | ring finger protein 220 | 66743 | ENSMUSG00000028677 |
| 125 | 17468195 | NA | Stambp | STAM binding protein | 70527 | ENSMUSG00000006906 |
| 126 | 17408684 | NA | Dclre1b | DNA cross-link repair 1B, PSO2 homolog (S. cerevisiae) | 140917 | ENSMUSG00000027845 |
| 127 | 17436077 | NA | Nrbp1 | nuclear receptor binding protein 1 | 192292 | ENSMUSG00000029148 |
| 128 | 17342015 | NA | Tbl3 | transducin (beta)-like 3 | 213773 | ENSMUSG00000040688 |
| 129 | 17252170 | NA | Rnf167 | ring finger protein 167 | 70510 | ENSMUSG00000040746 |
| 130 | 17543988 | NA | Taf9b | TAF9B RNA polymerase II, TATA box binding protein (TBP)-associated factor | 407786 | ENSMUSG00000047242 |
| 131 | 17509721 | NA | Tufm | Tu translation elongation factor, mitochondrial | 233870 | ENSMUSG00000073838 |
| 132 | 17459676 | NA | Retsat | retinol saturase (all trans retinol 13,14 reductase) | 67442 | ENSMUSG00000056666 |
| 133 | 17279858 | NA | Fkbp1b | FK506 binding protein 1b | 14226 | ENSMUSG00000020635 |
| 134 | 17488463 | NA | Med29 | mediator complex subunit 29 | 67224 | ENSMUSG00000003444 |
| 135 | 17391270 | NA | Kcnip3 | Kv channel interacting protein 3, calsenilin | 56461 | ENSMUSG00000079056 |
| 136 | 17324576 | NA | Hrasls | HRAS-like suppressor | 27281 | ENSMUSG00000022525 |
| 137 | 17531834 | NA | Fbxl2 | F-box and leucine-rich repeat protein 2 | 72179 | ENSMUSG00000032507 |
| 138 | 17460099 | NA | Vax2 | ventral anterior homeobox containing gene 2 | 24113 | ENSMUSG00000034777 |
| 139 | 17358777 | NA | Stambpl1 | STAM binding protein like 1 | 76630 | ENSMUSG00000024776 |
| 140 | 17257060 | NA | Nmt1 | N-myristoyltransferase 1 | 18107 | ENSMUSG00000020936 |
| 141 | 17292107 | NA | Tbc1d7 | TBC1 domain family, member 7 | 67046 | ENSMUSG00000021368 |
| 142 | 17498239 | NA | Kcnq1ot1 | KCNQ1 overlapping transcript 1 | 63830 | NULL |
| 143 | 17512479 | NA | Acd | adrenocortical dysplasia | 497652 | ENSMUSG00000038000 |
| 144 | 17540050 | NA | Ebp | phenylalkylamine Ca2+ antagonist (emopamil) binding protein | 13595 | ENSMUSG00000031168 |
| 145 | 17429495 | NA | Nfyc | nuclear transcription factor-Y gamma | 18046 | ENSMUSG00000032897 |
| 146 | 17361855 | NA | Pola2 | polymerase (DNA directed), alpha 2 | 18969 | ENSMUSG00000024833 |
| 147 | 17527532 | NA | Mpi | mannose phosphate isomerase | 110119 | ENSMUSG00000032306 |
| 148 | 17353639 | NA | Dnajc18 | DnaJ (Hsp40) homolog, subfamily C, member 18 | 76594 | ENSMUSG00000024350 |
| 149 | 17512463 | NA | Atp6v0d1 | ATPase, H+ transporting, lysosomal V0 subunit D1 | 11972 | ENSMUSG00000013160 |
| 150 | 17512466 | NA | Atp6v0d1 | ATPase, H+ transporting, lysosomal V0 subunit D1 | 11972 | ENSMUSG00000013160 |
| 151 | 17252635 | NA | Shpk | sedoheptulokinase | 74637 | ENSMUSG00000005951 |
| 152 | 17212813 | NA | Mstn | myostatin | 17700 | ENSMUSG00000026100 |
| 153 | 17230945 | NA | Smyd2 | SET and MYND domain containing 2 | 226830 | ENSMUSG00000026603 |
| 154 | 17313008 | NA | Cby1 | chibby homolog 1 (Drosophila) | 73739 | ENSMUSG00000022428 |
| 155 | 17521448 | NA | Hyal3 | hyaluronoglucosaminidase 3 | 109685 | ENSMUSG00000036091 |
| 156 | 17535434 | NA | Nsdhl | NAD(P) dependent steroid dehydrogenase-like | 18194 | ENSMUSG00000031349 |
| 157 | 17468113 | NA | Ino80b | INO80 complex subunit B | 70020 | ENSMUSG00000030034 |
| 158 | 17347558 | NA | Cdkl4 | cyclin-dependent kinase-like 4 | 381113 | ENSMUSG00000033966 |
| 159 | 17503023 | NA | Asf1b | ASF1 anti-silencing function 1 homolog B (S. cerevisiae) | 66929 | ENSMUSG00000005470 |
| 160 | 17283939 | NA | Wars | tryptophanyl-tRNA synthetase | 22375 | ENSMUSG00000021266 |
| 161 | 17283941 | NA | Wars | tryptophanyl-tRNA synthetase | 22375 | ENSMUSG00000021266 |
| 162 | 17283930 | NA | Wars | tryptophanyl-tRNA synthetase | 22375 | ENSMUSG00000021266 |
| 163 | 17283938 | NA | Wars | tryptophanyl-tRNA synthetase | 22375 | ENSMUSG00000021266 |
| 164 | 17258457 | NA | Sap30bp | SAP30 binding protein | 57230 | ENSMUSG00000020755 |
| 165 | 17454256 | NA | Taf6 | TAF6 RNA polymerase II, TATA box binding protein (TBP)-associated factor | 21343 | ENSMUSG00000036980 |
| 166 | 17510365 | NA | Pgls | 6-phosphogluconolactonase | 66171 | ENSMUSG00000031807 |
| 167 | 17231118 | NA | Rcor3 | REST corepressor 3 | 214742 | ENSMUSG00000037395 |
| 168 | 17298407 | NA | Bap1 | Brca1 associated protein 1 | 104416 | ENSMUSG00000021901 |
| 169 | 17265186 | NA | Rnasek | ribonuclease, RNase K | 52898 | ENSMUSG00000093989 |
| 170 | 17405174 | NA | Cog6 | component of oligomeric golgi complex 6 | 67542 | ENSMUSG00000027742 |
| 171 | 17221014 | NA | Cd34 | CD34 antigen | 12490 | ENSMUSG00000016494 |
| 172 | 17273280 | NA | Stra13 | stimulated by retinoic acid 13 | 20892 | ENSMUSG00000025144 |
| 173 | 17301342 | NA | Ints9 | integrator complex subunit 9 | 210925 | ENSMUSG00000021975 |
| 174 | 17241409 | NA | Srgn | serglycin | 19073 | ENSMUSG00000020077 |
| 175 | 17393357 | NA | Eif6 | eukaryotic translation initiation factor 6 | 16418 | ENSMUSG00000027613 |
| 176 | 17412593 | NA | Srsf12 | serine/arginine-rich splicing factor 12 | 272009 | ENSMUSG00000054679 |
| 177 | 17213990 | NA | Atic | 5-aminoimidazole-4-carboxamide ribonucleotide formyltransferase/IMP cyclohydrolase | 108147 | ENSMUSG00000026192 |
| 178 | 17504160 | NA | Polr2c | polymerase (RNA) II (DNA directed) polypeptide C | 20021 | ENSMUSG00000031783 |
| 179 | 17455093 | NA | Zkscan14 | zinc finger with KRAB and SCAN domains 14 | 67235 | ENSMUSG00000029627 |
| 180 | 17508691 | NA | Rbpms | RNA binding protein gene with multiple splicing | 19663 | ENSMUSG00000031586 |
| 181 | 17222001 | NA | Prim2 | DNA primase, p58 subunit | 19076 | ENSMUSG00000026134 |
| 182 | 17232649 | NA | Fyn | Fyn proto-oncogene | 14360 | ENSMUSG00000019843 |
| 183 | 17328810 | NA | Dgcr14 | DiGeorge syndrome critical region gene 14 | 27886 | ENSMUSG00000003527 |
| 184 | 17395844 | NA | Stmn3 | stathmin-like 3 | 20262 | ENSMUSG00000027581 |
| 185 | 17229466 | NA | Hsd17b7 | hydroxysteroid (17-beta) dehydrogenase 7 | 15490 | ENSMUSG00000026675 |
| 186 | 17219286 | NA | Dedd | death effector domain-containing | 21945 | ENSMUSG00000013973 |
| 187 | 17241780 | NA | Ube2d1 | ubiquitin-conjugating enzyme E2D 1 | 216080 | ENSMUSG00000019927 |
| 188 | 17334495 | NA | Nme3 | NME/NM23 nucleoside diphosphate kinase 3 | 79059 | ENSMUSG00000073435 |
| 189 | 17313199 | NA | Adsl | adenylosuccinate lyase | 11564 | ENSMUSG00000022407 |
| 190 | 17418447 | NA | Meaf6 | MYST/Esa1-associated factor 6 | 70088 | ENSMUSG00000028863 |
| 191 | 17356202 | NA | Pold4 | polymerase (DNA-directed), delta 4 | 69745 | ENSMUSG00000024854 |
| 192 | 17421972 | NA | Errfi1 | ERBB receptor feedback inhibitor 1 | 74155 | ENSMUSG00000028967 |
| 193 | 17330373 | NA | Adprh | ADP-ribosylarginine hydrolase | 11544 | ENSMUSG00000002844 |

  
  

| **Database:biological process      &nbspName:metabolic process      &nbspID:GO:0008152** | | | | | | |
| --- | --- | --- | --- | --- | --- | --- |
| C=8787; O=209; E=168.98; R=1.24; rawP=2.69e-05; adjP=0.0070 | | | | | | |
| Index | UserID | Value | Gene Symbol | Gene Name | EntrezGene | Ensembl |
| 1 | 17476273 | NA | Zfp382 | zinc finger protein 382 | 233060 | ENSMUSG00000074220 |
| 2 | 17232843 | NA | Zbtb24 | zinc finger and BTB domain containing 24 | 268294 | ENSMUSG00000019826 |
| 3 | 17344794 | NA | Znrd1 | zinc ribbon domain containing, 1 | 66136 | ENSMUSG00000036315 |
| 4 | 17300261 | NA | Oxa1l | oxidase assembly 1-like | 69089 | ENSMUSG00000000959 |
| 5 | 17512740 | NA | Nob1 | NIN1/RPN12 binding protein 1 homolog (S. cerevisiae) | 67619 | ENSMUSG00000003848 |
| 6 | 17224540 | NA | Tuba4a | tubulin, alpha 4A | 22145 | ENSMUSG00000026202 |
| 7 | 17503910 | NA | Ogfod1 | 2-oxoglutarate and iron-dependent oxygenase domain containing 1 | 270086 | ENSMUSG00000033009 |
| 8 | 17368171 | NA | Bmyc | brain expressed myelocytomatosis oncogene | 107771 | ENSMUSG00000049086 |
| 9 | 17350134 | NA | Pou4f3 | POU domain, class 4, transcription factor 3 | 18998 | ENSMUSG00000024497 |
| 10 | 17517723 | NA | Rpp25 | ribonuclease P 25 subunit (human) | 102614 | ENSMUSG00000062309 |
| 11 | 17307134 | NA | Cryl1 | crystallin, lambda 1 | 68631 | ENSMUSG00000021947 |
| 12 | 17443181 | NA | Dnajc30 | DnaJ (Hsp40) homolog, subfamily C, member 30 | 66114 | ENSMUSG00000061118 |
| 13 | 17512103 | NA | Got2 | glutamate oxaloacetate transaminase 2, mitochondrial | 14719 | ENSMUSG00000031672 |
| 14 | 17369862 | NA | Dpm2 | dolichol-phosphate (beta-D) mannosyltransferase 2 | 13481 | ENSMUSG00000026810 |
| 15 | 17336829 | NA | Lsm2 | LSM2 homolog, U6 small nuclear RNA associated (S. cerevisiae) | 27756 | ENSMUSG00000007050 |
| 16 | 17288454 | NA | Irx4 | Iroquois related homeobox 4 (Drosophila) | 50916 | ENSMUSG00000021604 |
| 17 | 17467996 | NA | Mrpl19 | mitochondrial ribosomal protein L19 | 56284 | ENSMUSG00000030045 |
| 18 | 17322559 | NA | Hmox2 | heme oxygenase (decycling) 2 | 15369 | ENSMUSG00000004070 |
| 19 | 17306758 | NA | Tm9sf1 | transmembrane 9 superfamily member 1 | 74140 | ENSMUSG00000002320 |
| 20 | 17404329 | NA | Gyg | glycogenin | 27357 | ENSMUSG00000019528 |
| 21 | 17321467 | NA | Tuba1b | tubulin, alpha 1B | 22143 | ENSMUSG00000023004 |
| 22 | 17288160 | NA | Cdk20 | cyclin-dependent kinase 20 | 105278 | ENSMUSG00000021483 |
| 23 | 17538096 | NA | Rnf128 | ring finger protein 128 | 66889 | ENSMUSG00000031438 |
| 24 | 17447099 | NA | Ctbp1 | C-terminal binding protein 1 | 13016 | ENSMUSG00000037373 |
| 25 | 17447089 | NA | Ctbp1 | C-terminal binding protein 1 | 13016 | ENSMUSG00000037373 |
| 26 | 17447100 | NA | Ctbp1 | C-terminal binding protein 1 | 13016 | ENSMUSG00000037373 |
| 27 | 17447098 | NA | Ctbp1 | C-terminal binding protein 1 | 13016 | ENSMUSG00000037373 |
| 28 | 17343617 | NA | Rab11b | RAB11B, member RAS oncogene family | 19326 | ENSMUSG00000077450 |
| 29 | 17379187 | NA | Ift52 | intraflagellar transport 52 | 245866 | ENSMUSG00000017858 |
| 30 | 17440361 | NA | Plcxd1 | phosphatidylinositol-specific phospholipase C, X domain containing 1 | 403178 | ENSMUSG00000064247 |
| 31 | 17246284 | NA | Suox | sulfite oxidase | 211389 | ENSMUSG00000049858 |
| 32 | 17437611 | NA | Pgm1 | phosphoglucomutase 1 | 66681 | ENSMUSG00000029171 |
| 33 | 17265082 | NA | Eif5a | eukaryotic translation initiation factor 5A | 276770 | ENSMUSG00000078812 |
| 34 | 17297750 | NA | Ppif | peptidylprolyl isomerase F (cyclophilin F) | 105675 | ENSMUSG00000021868 |
| 35 | 17233630 | NA | Psap | prosaposin | 19156 | ENSMUSG00000004207 |
| 36 | 17520177 | NA | Mthfs | 5, 10-methenyltetrahydrofolate synthetase | 107885 | ENSMUSG00000066442 |
| 37 | 17480102 | NA | Sytl2 | synaptotagmin-like 2 | 83671 | ENSMUSG00000030616 |
| 38 | 17521143 | NA | Wdr82 | WD repeat domain containing 82 | 77305 | ENSMUSG00000020257 |
| 39 | 17528778 | NA | Ccpg1 | cell cycle progression 1 | 72278 | ENSMUSG00000034563 |
| 40 | 17278188 | NA | Otub2 | OTU domain, ubiquitin aldehyde binding 2 | 68149 | ENSMUSG00000021203 |
| 41 | 17225499 | NA | Hes6 | hairy and enhancer of split 6 (Drosophila) | 55927 | ENSMUSG00000067071 |
| 42 | 17312944 | NA | Polr2f | polymerase (RNA) II (DNA directed) polypeptide F | 69833 | ENSMUSG00000033020 |
| 43 | 17312939 | NA | Polr2f | polymerase (RNA) II (DNA directed) polypeptide F | 69833 | ENSMUSG00000033020 |
| 44 | 17312941 | NA | Polr2f | polymerase (RNA) II (DNA directed) polypeptide F | 69833 | ENSMUSG00000033020 |
| 45 | 17334722 | NA | Rpusd1 | RNA pseudouridylate synthase domain containing 1 | 106707 | ENSMUSG00000041199 |
| 46 | 17517576 | NA | Hmg20a | high mobility group 20A | 66867 | ENSMUSG00000032329 |
| 47 | 17393225 | NA | Pigu | phosphatidylinositol glycan anchor biosynthesis, class U | 228812 | ENSMUSG00000038383 |
| 48 | 17404011 | NA | Hey1 | hairy/enhancer-of-split related with YRPW motif 1 | 15213 | ENSMUSG00000040289 |
| 49 | 17400222 | NA | Vps72 | vacuolar protein sorting 72 (yeast) | 21427 | ENSMUSG00000008958 |
| 50 | 17499396 | NA | Fbxo25 | F-box protein 25 | 66822 | ENSMUSG00000038365 |
| 51 | 17246850 | NA | Zmat5 | zinc finger, matrin type 5 | 67178 | ENSMUSG00000009076 |
| 52 | 17324664 | NA | Dlg1 | discs, large homolog 1 (Drosophila) | 13383 | ENSMUSG00000022770 |
| 53 | 17396369 | NA | Nceh1 | arylacetamide deacetylase-like 1 | 320024 | ENSMUSG00000027698 |
| 54 | 17502191 | NA | Mrpl34 | mitochondrial ribosomal protein L34 | 94065 | ENSMUSG00000034880 |
| 55 | 17529231 | NA | Phip | pleckstrin homology domain interacting protein | 83946 | ENSMUSG00000032253 |
| 56 | 17316625 | NA | Ubr5 | ubiquitin protein ligase E3 component n-recognin 5 | 70790 | ENSMUSG00000037487 |
| 57 | 17277370 | NA | Eif2b2 | eukaryotic translation initiation factor 2B, subunit 2 beta | 217715 | ENSMUSG00000004788 |
| 58 | 17483220 | NA | Cdipt | CDP-diacylglycerol--inositol 3-phosphatidyltransferase (phosphatidylinositol synthase) | 52858 | ENSMUSG00000030682 |
| 59 | 17245709 | NA | Os9 | amplified in osteosarcoma | 216440 | ENSMUSG00000040462 |
| 60 | 17512009 | NA | Csnk2a2 | casein kinase 2, alpha prime polypeptide | 13000 | ENSMUSG00000046707 |
| 61 | 17424319 | NA | Sigmar1 | sigma non-opioid intracellular receptor 1 | 18391 | ENSMUSG00000036078 |
| 62 | 17503816 | NA | Irx6 | Iroquois related homeobox 6 (Drosophila) | 64379 | ENSMUSG00000031738 |
| 63 | 17321578 | NA | Bcdin3d | BCDIN3 domain containing | 75284 | ENSMUSG00000037525 |
| 64 | 17282649 | NA | Rps6kl1 | ribosomal protein S6 kinase-like 1 | 238323 | ENSMUSG00000019235 |
| 65 | 17260221 | NA | Pold2 | polymerase (DNA directed), delta 2, regulatory subunit | 18972 | ENSMUSG00000020471 |
| 66 | 17365493 | NA | Obfc1 | oligonucleotide/oligosaccharide-binding fold containing 1 | 108689 | ENSMUSG00000042694 |
| 67 | 17362216 | NA | Nudt22 | nudix (nucleoside diphosphate linked moiety X)-type motif 22 | 68323 | ENSMUSG00000037349 |
| 68 | 17538790 | NA | Huwe1 | HECT, UBA and WWE domain containing 1 | 59026 | ENSMUSG00000025261 |
| 69 | 17446580 | NA | Shh | sonic hedgehog | 20423 | ENSMUSG00000002633 |
| 70 | 17470879 | NA | Tpi1 | triosephosphate isomerase 1 | 21991 | ENSMUSG00000023456 |
| 71 | 17281971 | NA | Sgpp1 | sphingosine-1-phosphate phosphatase 1 | 81535 | ENSMUSG00000021054 |
| 72 | 17230408 | NA | Adck3 | aarF domain containing kinase 3 | 67426 | ENSMUSG00000026489 |
| 73 | 17238549 | NA | Wibg | within bgcn homolog (Drosophila) | 78428 | ENSMUSG00000064030 |
| 74 | 17212355 | NA | Nck2 | non-catalytic region of tyrosine kinase adaptor protein 2 | 17974 | ENSMUSG00000066877 |
| 75 | 17431502 | NA | Lypla2 | lysophospholipase 2 | 26394 | ENSMUSG00000028670 |
| 76 | 17307695 | NA | Msra | methionine sulfoxide reductase A | 110265 | ENSMUSG00000054733 |
| 77 | 17303496 | NA | Fezf2 | Fez family zinc finger 2 | 54713 | ENSMUSG00000021743 |
| 78 | 17256565 | NA | Tubg2 | tubulin, gamma 2 | 103768 | ENSMUSG00000045007 |
| 79 | 17500275 | NA | Erlin2 | ER lipid raft associated 2 | 244373 | ENSMUSG00000031483 |
| 80 | 17345519 | NA | Rrp36 | ribosomal RNA processing 36 homolog (S. cerevisiae) | 224823 | ENSMUSG00000023971 |
| 81 | 17463422 | NA | Nrip2 | nuclear receptor interacting protein 2 | 60345 | ENSMUSG00000001520 |
| 82 | 17211335 | NA | Tfap2d | transcription factor AP-2, delta | 226896 | ENSMUSG00000042596 |
| 83 | 17506631 | NA | Tubb3 | tubulin, beta 3 class III | 22152 | ENSMUSG00000062380 |
| 84 | 17309825 | NA | Plcxd3 | phosphatidylinositol-specific phospholipase C, X domain containing 3 | 239318 | ENSMUSG00000049148 |
| 85 | 17321722 | NA | Tfcp2 | transcription factor CP2 | 21422 | ENSMUSG00000009733 |
| 86 | 17213153 | NA | Nif3l1 | Ngg1 interacting factor 3-like 1 (S. pombe) | 65102 | ENSMUSG00000026036 |
| 87 | 17235584 | NA | Dapk3 | death-associated protein kinase 3 | 13144 | ENSMUSG00000034974 |
| 88 | 17477670 | NA | Rras | Harvey rat sarcoma oncogene, subgroup R | 20130 | ENSMUSG00000038387 |
| 89 | 17405819 | NA | B3galnt1 | UDP-GalNAc:betaGlcNAc beta 1,3-galactosaminyltransferase, polypeptide 1 | 26879 | ENSMUSG00000043300 |
| 90 | 17300411 | NA | Thtpa | thiamine triphosphatase | 105663 | ENSMUSG00000045691 |
| 91 | 17498821 | NA | Snapc2 | small nuclear RNA activating complex, polypeptide 2 | 102209 | ENSMUSG00000011837 |
| 92 | 17460879 | NA | Hdac11 | histone deacetylase 11 | 232232 | ENSMUSG00000034245 |
| 93 | 17256618 | NA | Vps25 | vacuolar protein sorting 25 (yeast) | 28084 | ENSMUSG00000078656 |
| 94 | 17444100 | NA | Chst12 | carbohydrate sulfotransferase 12 | 59031 | ENSMUSG00000036599 |
| 95 | 17517532 | NA | Isl2 | insulin related protein 2 (islet 2) | 104360 | ENSMUSG00000032318 |
| 96 | 17494637 | NA | Mrpl17 | mitochondrial ribosomal protein L17 | 27397 | ENSMUSG00000030879 |
| 97 | 17513871 | NA | Chmp1a | charged multivesicular body protein 1A | 234852 | ENSMUSG00000000743 |
| 98 | 17359994 | NA | Fbxl15 | F-box and leucine-rich repeat protein 15 | 68431 | ENSMUSG00000025226 |
| 99 | 17484068 | NA | Lhpp | phospholysine phosphohistidine inorganic pyrophosphate phosphatase | 76429 | ENSMUSG00000030946 |
| 100 | 17497366 | NA | Ebf3 | early B cell factor 3 | 13593 | ENSMUSG00000010476 |
| 101 | 17446322 | NA | Prkag2 | protein kinase, AMP-activated, gamma 2 non-catalytic subunit | 108099 | ENSMUSG00000028944 |
| 102 | 17524523 | NA | Eif3g | eukaryotic translation initiation factor 3, subunit G | 53356 | ENSMUSG00000070319 |
| 103 | 17306835 | NA | Rabggta | Rab geranylgeranyl transferase, a subunit | 56187 | ENSMUSG00000040472 |
| 104 | 17269638 | NA | Rab5c | RAB5C, member RAS oncogene family | 19345 | ENSMUSG00000019173 |
| 105 | 17505367 | NA | Txnl4b | thioredoxin-like 4B | 234723 | ENSMUSG00000031723 |
| 106 | 17256549 | NA | Tubg1 | tubulin, gamma 1 | 103733 | ENSMUSG00000035198 |
| 107 | 17498370 | NA | Nadsyn1 | NAD synthetase 1 | 78914 | ENSMUSG00000031090 |
| 108 | 17494081 | NA | Rhog | ras homolog gene family, member G | 56212 | ENSMUSG00000073982 |
| 109 | 17497957 | NA | Chid1 | chitinase domain containing 1 | 68038 | ENSMUSG00000025512 |
| 110 | 17235268 | NA | Ndufs7 | NADH dehydrogenase (ubiquinone) Fe-S protein 7 | 75406 | ENSMUSG00000020153 |
| 111 | 17532045 | NA | Plcd1 | phospholipase C, delta 1 | 18799 | ENSMUSG00000010660 |
| 112 | 17274448 | NA | Cpsf3 | cleavage and polyadenylation specificity factor 3 | 54451 | ENSMUSG00000054309 |
| 113 | 17422117 | NA | Acot7 | acyl-CoA thioesterase 7 | 70025 | ENSMUSG00000028937 |
| 114 | 17519868 | NA | Htr1b | 5-hydroxytryptamine (serotonin) receptor 1B | 15551 | ENSMUSG00000049511 |
| 115 | 17536264 | NA | Pcyt1b | phosphate cytidylyltransferase 1, choline, beta isoform | 236899 | ENSMUSG00000035246 |
| 116 | 17515170 | NA | Ilf3 | interleukin enhancer binding factor 3 | 16201 | ENSMUSG00000032178 |
| 117 | 17357213 | NA | Zbtb3 | zinc finger and BTB domain containing 3 | 75291 | ENSMUSG00000071661 |
| 118 | 17430853 | NA | Med18 | mediator of RNA polymerase II transcription, subunit 18 homolog (yeast) | 67219 | ENSMUSG00000066042 |
| 119 | 17225179 | NA | Pde6d | phosphodiesterase 6D, cGMP-specific, rod, delta | 18582 | ENSMUSG00000026239 |
| 120 | 17322735 | NA | Mettl22 | methyltransferase like 22 | 239706 | ENSMUSG00000039345 |
| 121 | 17521014 | NA | Acad11 | acyl-Coenzyme A dehydrogenase family, member 11 | 102632 | ENSMUSG00000090150 |
| 122 | 17370285 | NA | Mrrf | mitochondrial ribosome recycling factor | 67871 | ENSMUSG00000026887 |
| 123 | 17361988 | NA | Arl2 | ADP-ribosylation factor-like 2 | 56327 | ENSMUSG00000024944 |
| 124 | 17229948 | NA | Dusp23 | dual specificity phosphatase 23 | 68440 | ENSMUSG00000026544 |
| 125 | 17502583 | NA | Mcm5 | minichromosome maintenance deficient 5, cell division cycle 46 (S. cerevisiae) | 17218 | ENSMUSG00000005410 |
| 126 | 17511296 | NA | Wdr83 | WD repeat domain containing 83 | 67836 | ENSMUSG00000005150 |
| 127 | 17539434 | NA | Ctps2 | cytidine 5'-triphosphate synthase 2 | 55936 | ENSMUSG00000031360 |
| 128 | 17341521 | NA | Thoc6 | THO complex 6 homolog (Drosophila) | 386612 | ENSMUSG00000041319 |
| 129 | 17250141 | NA | Zfp39 | zinc finger protein 39 | 22698 | ENSMUSG00000037001 |
| 130 | 17519718 | NA | Mto1 | mitochondrial translation optimization 1 homolog (S. cerevisiae) | 68291 | ENSMUSG00000032342 |
| 131 | 17521422 | NA | Hyal2 | hyaluronoglucosaminidase 2 | 15587 | ENSMUSG00000010047 |
| 132 | 17317208 | NA | Derl1 | Der1-like domain family, member 1 | 67819 | ENSMUSG00000022365 |
| 133 | 17501544 | NA | Npy1r | neuropeptide Y receptor Y1 | 18166 | ENSMUSG00000036437 |
| 134 | 17235714 | NA | Dohh | deoxyhypusine hydroxylase/monooxygenase | 102115 | ENSMUSG00000078440 |
| 135 | 17540589 | NA | Klhl13 | kelch-like 13 (Drosophila) | 67455 | ENSMUSG00000036782 |
| 136 | 17428858 | NA | Rnf220 | ring finger protein 220 | 66743 | ENSMUSG00000028677 |
| 137 | 17428857 | NA | Rnf220 | ring finger protein 220 | 66743 | ENSMUSG00000028677 |
| 138 | 17468195 | NA | Stambp | STAM binding protein | 70527 | ENSMUSG00000006906 |
| 139 | 17408684 | NA | Dclre1b | DNA cross-link repair 1B, PSO2 homolog (S. cerevisiae) | 140917 | ENSMUSG00000027845 |
| 140 | 17436077 | NA | Nrbp1 | nuclear receptor binding protein 1 | 192292 | ENSMUSG00000029148 |
| 141 | 17342015 | NA | Tbl3 | transducin (beta)-like 3 | 213773 | ENSMUSG00000040688 |
| 142 | 17252170 | NA | Rnf167 | ring finger protein 167 | 70510 | ENSMUSG00000040746 |
| 143 | 17543988 | NA | Taf9b | TAF9B RNA polymerase II, TATA box binding protein (TBP)-associated factor | 407786 | ENSMUSG00000047242 |
| 144 | 17342617 | NA | Arhgdig | Rho GDP dissociation inhibitor (GDI) gamma | 14570 | ENSMUSG00000073433 |
| 145 | 17509721 | NA | Tufm | Tu translation elongation factor, mitochondrial | 233870 | ENSMUSG00000073838 |
| 146 | 17459676 | NA | Retsat | retinol saturase (all trans retinol 13,14 reductase) | 67442 | ENSMUSG00000056666 |
| 147 | 17279858 | NA | Fkbp1b | FK506 binding protein 1b | 14226 | ENSMUSG00000020635 |
| 148 | 17488463 | NA | Med29 | mediator complex subunit 29 | 67224 | ENSMUSG00000003444 |
| 149 | 17391270 | NA | Kcnip3 | Kv channel interacting protein 3, calsenilin | 56461 | ENSMUSG00000079056 |
| 150 | 17324576 | NA | Hrasls | HRAS-like suppressor | 27281 | ENSMUSG00000022525 |
| 151 | 17531834 | NA | Fbxl2 | F-box and leucine-rich repeat protein 2 | 72179 | ENSMUSG00000032507 |
| 152 | 17460099 | NA | Vax2 | ventral anterior homeobox containing gene 2 | 24113 | ENSMUSG00000034777 |
| 153 | 17358777 | NA | Stambpl1 | STAM binding protein like 1 | 76630 | ENSMUSG00000024776 |
| 154 | 17257060 | NA | Nmt1 | N-myristoyltransferase 1 | 18107 | ENSMUSG00000020936 |
| 155 | 17292107 | NA | Tbc1d7 | TBC1 domain family, member 7 | 67046 | ENSMUSG00000021368 |
| 156 | 17498239 | NA | Kcnq1ot1 | KCNQ1 overlapping transcript 1 | 63830 | NULL |
| 157 | 17413573 | NA | Grhpr | glyoxylate reductase/hydroxypyruvate reductase | 76238 | ENSMUSG00000035637 |
| 158 | 17512479 | NA | Acd | adrenocortical dysplasia | 497652 | ENSMUSG00000038000 |
| 159 | 17288716 | NA | Glrx | glutaredoxin | 93692 | ENSMUSG00000021591 |
| 160 | 17540050 | NA | Ebp | phenylalkylamine Ca2+ antagonist (emopamil) binding protein | 13595 | ENSMUSG00000031168 |
| 161 | 17429495 | NA | Nfyc | nuclear transcription factor-Y gamma | 18046 | ENSMUSG00000032897 |
| 162 | 17361855 | NA | Pola2 | polymerase (DNA directed), alpha 2 | 18969 | ENSMUSG00000024833 |
| 163 | 17527532 | NA | Mpi | mannose phosphate isomerase | 110119 | ENSMUSG00000032306 |
| 164 | 17353639 | NA | Dnajc18 | DnaJ (Hsp40) homolog, subfamily C, member 18 | 76594 | ENSMUSG00000024350 |
| 165 | 17232426 | NA | Echdc1 | enoyl Coenzyme A hydratase domain containing 1 | 52665 | ENSMUSG00000019883 |
| 166 | 17512463 | NA | Atp6v0d1 | ATPase, H+ transporting, lysosomal V0 subunit D1 | 11972 | ENSMUSG00000013160 |
| 167 | 17512466 | NA | Atp6v0d1 | ATPase, H+ transporting, lysosomal V0 subunit D1 | 11972 | ENSMUSG00000013160 |
| 168 | 17252635 | NA | Shpk | sedoheptulokinase | 74637 | ENSMUSG00000005951 |
| 169 | 17212813 | NA | Mstn | myostatin | 17700 | ENSMUSG00000026100 |
| 170 | 17230945 | NA | Smyd2 | SET and MYND domain containing 2 | 226830 | ENSMUSG00000026603 |
| 171 | 17313008 | NA | Cby1 | chibby homolog 1 (Drosophila) | 73739 | ENSMUSG00000022428 |
| 172 | 17521448 | NA | Hyal3 | hyaluronoglucosaminidase 3 | 109685 | ENSMUSG00000036091 |
| 173 | 17535434 | NA | Nsdhl | NAD(P) dependent steroid dehydrogenase-like | 18194 | ENSMUSG00000031349 |
| 174 | 17342359 | NA | Haghl | hydroxyacylglutathione hydrolase-like | 68977 | ENSMUSG00000061046 |
| 175 | 17468113 | NA | Ino80b | INO80 complex subunit B | 70020 | ENSMUSG00000030034 |
| 176 | 17347558 | NA | Cdkl4 | cyclin-dependent kinase-like 4 | 381113 | ENSMUSG00000033966 |
| 177 | 17503023 | NA | Asf1b | ASF1 anti-silencing function 1 homolog B (S. cerevisiae) | 66929 | ENSMUSG00000005470 |
| 178 | 17283939 | NA | Wars | tryptophanyl-tRNA synthetase | 22375 | ENSMUSG00000021266 |
| 179 | 17283941 | NA | Wars | tryptophanyl-tRNA synthetase | 22375 | ENSMUSG00000021266 |
| 180 | 17283930 | NA | Wars | tryptophanyl-tRNA synthetase | 22375 | ENSMUSG00000021266 |
| 181 | 17283938 | NA | Wars | tryptophanyl-tRNA synthetase | 22375 | ENSMUSG00000021266 |
| 182 | 17258457 | NA | Sap30bp | SAP30 binding protein | 57230 | ENSMUSG00000020755 |
| 183 | 17454256 | NA | Taf6 | TAF6 RNA polymerase II, TATA box binding protein (TBP)-associated factor | 21343 | ENSMUSG00000036980 |
| 184 | 17432967 | NA | Ubiad1 | UbiA prenyltransferase domain containing 1 | 71707 | ENSMUSG00000047719 |
| 185 | 17510365 | NA | Pgls | 6-phosphogluconolactonase | 66171 | ENSMUSG00000031807 |
| 186 | 17526273 | NA | Trappc4 | trafficking protein particle complex 4 | 60409 | ENSMUSG00000032112 |
| 187 | 17526271 | NA | Trappc4 | trafficking protein particle complex 4 | 60409 | ENSMUSG00000032112 |
| 188 | 17526272 | NA | Trappc4 | trafficking protein particle complex 4 | 60409 | ENSMUSG00000032112 |
| 189 | 17231118 | NA | Rcor3 | REST corepressor 3 | 214742 | ENSMUSG00000037395 |
| 190 | 17298407 | NA | Bap1 | Brca1 associated protein 1 | 104416 | ENSMUSG00000021901 |
| 191 | 17318877 | NA | Txn2 | thioredoxin 2 | 56551 | ENSMUSG00000005354 |
| 192 | 17329163 | NA | Camk2n2 | calcium/calmodulin-dependent protein kinase II inhibitor 2 | 73047 | ENSMUSG00000051146 |
| 193 | 17265186 | NA | Rnasek | ribonuclease, RNase K | 52898 | ENSMUSG00000093989 |
| 194 | 17405174 | NA | Cog6 | component of oligomeric golgi complex 6 | 67542 | ENSMUSG00000027742 |
| 195 | 17221014 | NA | Cd34 | CD34 antigen | 12490 | ENSMUSG00000016494 |
| 196 | 17273280 | NA | Stra13 | stimulated by retinoic acid 13 | 20892 | ENSMUSG00000025144 |
| 197 | 17301342 | NA | Ints9 | integrator complex subunit 9 | 210925 | ENSMUSG00000021975 |
| 198 | 17241409 | NA | Srgn | serglycin | 19073 | ENSMUSG00000020077 |
| 199 | 17393357 | NA | Eif6 | eukaryotic translation initiation factor 6 | 16418 | ENSMUSG00000027613 |
| 200 | 17412593 | NA | Srsf12 | serine/arginine-rich splicing factor 12 | 272009 | ENSMUSG00000054679 |
| 201 | 17213990 | NA | Atic | 5-aminoimidazole-4-carboxamide ribonucleotide formyltransferase/IMP cyclohydrolase | 108147 | ENSMUSG00000026192 |
| 202 | 17504160 | NA | Polr2c | polymerase (RNA) II (DNA directed) polypeptide C | 20021 | ENSMUSG00000031783 |
| 203 | 17455093 | NA | Zkscan14 | zinc finger with KRAB and SCAN domains 14 | 67235 | ENSMUSG00000029627 |
| 204 | 17508691 | NA | Rbpms | RNA binding protein gene with multiple splicing | 19663 | ENSMUSG00000031586 |
| 205 | 17222001 | NA | Prim2 | DNA primase, p58 subunit | 19076 | ENSMUSG00000026134 |
| 206 | 17232649 | NA | Fyn | Fyn proto-oncogene | 14360 | ENSMUSG00000019843 |
| 207 | 17328810 | NA | Dgcr14 | DiGeorge syndrome critical region gene 14 | 27886 | ENSMUSG00000003527 |
| 208 | 17395844 | NA | Stmn3 | stathmin-like 3 | 20262 | ENSMUSG00000027581 |
| 209 | 17229466 | NA | Hsd17b7 | hydroxysteroid (17-beta) dehydrogenase 7 | 15490 | ENSMUSG00000026675 |
| 210 | 17219286 | NA | Dedd | death effector domain-containing | 21945 | ENSMUSG00000013973 |
| 211 | 17306861 | NA | Dhrs1 | dehydrogenase/reductase (SDR family) member 1 | 52585 | ENSMUSG00000002332 |
| 212 | 17306864 | NA | Dhrs1 | dehydrogenase/reductase (SDR family) member 1 | 52585 | ENSMUSG00000002332 |
| 213 | 17306860 | NA | Dhrs1 | dehydrogenase/reductase (SDR family) member 1 | 52585 | ENSMUSG00000002332 |
| 214 | 17306856 | NA | Dhrs1 | dehydrogenase/reductase (SDR family) member 1 | 52585 | ENSMUSG00000002332 |
| 215 | 17306865 | NA | Dhrs1 | dehydrogenase/reductase (SDR family) member 1 | 52585 | ENSMUSG00000002332 |
| 216 | 17479069 | NA | Lysmd4 | LysM, putative peptidoglycan-binding, domain containing 4 | 75099 | ENSMUSG00000043831 |
| 217 | 17241780 | NA | Ube2d1 | ubiquitin-conjugating enzyme E2D 1 | 216080 | ENSMUSG00000019927 |
| 218 | 17334495 | NA | Nme3 | NME/NM23 nucleoside diphosphate kinase 3 | 79059 | ENSMUSG00000073435 |
| 219 | 17383588 | NA | Ccbl1 | cysteine conjugate-beta lyase 1 | 70266 | ENSMUSG00000039648 |
| 220 | 17313199 | NA | Adsl | adenylosuccinate lyase | 11564 | ENSMUSG00000022407 |
| 221 | 17232215 | NA | Moxd1 | monooxygenase, DBH-like 1 | 59012 | ENSMUSG00000020000 |
| 222 | 17418447 | NA | Meaf6 | MYST/Esa1-associated factor 6 | 70088 | ENSMUSG00000028863 |
| 223 | 17356202 | NA | Pold4 | polymerase (DNA-directed), delta 4 | 69745 | ENSMUSG00000024854 |
| 224 | 17421972 | NA | Errfi1 | ERBB receptor feedback inhibitor 1 | 74155 | ENSMUSG00000028967 |
| 225 | 17330373 | NA | Adprh | ADP-ribosylarginine hydrolase | 11544 | ENSMUSG00000002844 |

  
  

| **Database:biological process      &nbspName:cellular component organization or biogenesis at cellular level      &nbspID:GO:0071841** | | | | | | |
| --- | --- | --- | --- | --- | --- | --- |
| C=2788; O=81; E=53.62; R=1.51; rawP=7.92e-05; adjP=0.0094 | | | | | | |
| Index | UserID | Value | Gene Symbol | Gene Name | EntrezGene | Ensembl |
| 1 | 17235268 | NA | Ndufs7 | NADH dehydrogenase (ubiquinone) Fe-S protein 7 | 75406 | ENSMUSG00000020153 |
| 2 | 17255719 | NA | Mrpl10 | mitochondrial ribosomal protein L10 | 107732 | ENSMUSG00000001445 |
| 3 | 17413221 | NA | Unc13b | unc-13 homolog B (C. elegans) | 22249 | ENSMUSG00000028456 |
| 4 | 17300261 | NA | Oxa1l | oxidase assembly 1-like | 69089 | ENSMUSG00000000959 |
| 5 | 17335357 | NA | Lhfpl5 | lipoma HMGIC fusion partner-like 5 | 328789 | ENSMUSG00000062252 |
| 6 | 17512740 | NA | Nob1 | NIN1/RPN12 binding protein 1 homolog (S. cerevisiae) | 67619 | ENSMUSG00000003848 |
| 7 | 17224540 | NA | Tuba4a | tubulin, alpha 4A | 22145 | ENSMUSG00000026202 |
| 8 | 17350134 | NA | Pou4f3 | POU domain, class 4, transcription factor 3 | 18998 | ENSMUSG00000024497 |
| 9 | 17336829 | NA | Lsm2 | LSM2 homolog, U6 small nuclear RNA associated (S. cerevisiae) | 27756 | ENSMUSG00000007050 |
| 10 | 17370285 | NA | Mrrf | mitochondrial ribosome recycling factor | 67871 | ENSMUSG00000026887 |
| 11 | 17361988 | NA | Arl2 | ADP-ribosylation factor-like 2 | 56327 | ENSMUSG00000024944 |
| 12 | 17519718 | NA | Mto1 | mitochondrial translation optimization 1 homolog (S. cerevisiae) | 68291 | ENSMUSG00000032342 |
| 13 | 17266322 | NA | Eral1 | Era (G-protein)-like 1 (E. coli) | 57837 | ENSMUSG00000020832 |
| 14 | 17321467 | NA | Tuba1b | tubulin, alpha 1B | 22143 | ENSMUSG00000023004 |
| 15 | 17376272 | NA | Nop56 | NOP56 ribonucleoprotein homolog (yeast) | 67134 | ENSMUSG00000027405 |
| 16 | 17540589 | NA | Klhl13 | kelch-like 13 (Drosophila) | 67455 | ENSMUSG00000036782 |
| 17 | 17408684 | NA | Dclre1b | DNA cross-link repair 1B, PSO2 homolog (S. cerevisiae) | 140917 | ENSMUSG00000027845 |
| 18 | 17541597 | NA | Frmd7 | FERM domain containing 7 | 385354 | ENSMUSG00000036131 |
| 19 | 17263594 | NA | Atpaf2 | ATP synthase mitochondrial F1 complex assembly factor 2 | 246782 | ENSMUSG00000042709 |
| 20 | 17342015 | NA | Tbl3 | transducin (beta)-like 3 | 213773 | ENSMUSG00000040688 |
| 21 | 17447099 | NA | Ctbp1 | C-terminal binding protein 1 | 13016 | ENSMUSG00000037373 |
| 22 | 17447089 | NA | Ctbp1 | C-terminal binding protein 1 | 13016 | ENSMUSG00000037373 |
| 23 | 17447100 | NA | Ctbp1 | C-terminal binding protein 1 | 13016 | ENSMUSG00000037373 |
| 24 | 17447098 | NA | Ctbp1 | C-terminal binding protein 1 | 13016 | ENSMUSG00000037373 |
| 25 | 17265082 | NA | Eif5a | eukaryotic translation initiation factor 5A | 276770 | ENSMUSG00000078812 |
| 26 | 17297750 | NA | Ppif | peptidylprolyl isomerase F (cyclophilin F) | 105675 | ENSMUSG00000021868 |
| 27 | 17324576 | NA | Hrasls | HRAS-like suppressor | 27281 | ENSMUSG00000022525 |
| 28 | 17217580 | NA | Arl8a | ADP-ribosylation factor-like 8A | 68724 | ENSMUSG00000026426 |
| 29 | 17460099 | NA | Vax2 | ventral anterior homeobox containing gene 2 | 24113 | ENSMUSG00000034777 |
| 30 | 17521143 | NA | Wdr82 | WD repeat domain containing 82 | 77305 | ENSMUSG00000020257 |
| 31 | 17348933 | NA | Mapre2 | microtubule-associated protein, RP/EB family, member 2 | 212307 | ENSMUSG00000024277 |
| 32 | 17512434 | NA | Tppp3 | tubulin polymerization-promoting protein family member 3 | 67971 | ENSMUSG00000014846 |
| 33 | 17377583 | NA | Nsfl1c | NSFL1 (p97) cofactor (p47) | 386649 | ENSMUSG00000027455 |
| 34 | 17265175 | NA | 0610010K14Rik | RIKEN cDNA 0610010K14 gene | 104457 | ENSMUSG00000020831 |
| 35 | 17517576 | NA | Hmg20a | high mobility group 20A | 66867 | ENSMUSG00000032329 |
| 36 | 17512479 | NA | Acd | adrenocortical dysplasia | 497652 | ENSMUSG00000038000 |
| 37 | 17400222 | NA | Vps72 | vacuolar protein sorting 72 (yeast) | 21427 | ENSMUSG00000008958 |
| 38 | 17230945 | NA | Smyd2 | SET and MYND domain containing 2 | 226830 | ENSMUSG00000026603 |
| 39 | 17313008 | NA | Cby1 | chibby homolog 1 (Drosophila) | 73739 | ENSMUSG00000022428 |
| 40 | 17324664 | NA | Dlg1 | discs, large homolog 1 (Drosophila) | 13383 | ENSMUSG00000022770 |
| 41 | 17502390 | NA | Rab8a | RAB8A, member RAS oncogene family | 17274 | ENSMUSG00000003037 |
| 42 | 17238846 | NA | Syne1 | synaptic nuclear envelope 1 | 64009 | ENSMUSG00000019769 ENSMUSG00000096054 |
| 43 | 17238906 | NA | Syne1 | synaptic nuclear envelope 1 | 64009 | ENSMUSG00000019769 ENSMUSG00000096054 |
| 44 | 17238890 | NA | Syne1 | synaptic nuclear envelope 1 | 64009 | ENSMUSG00000019769 ENSMUSG00000096054 |
| 45 | 17503023 | NA | Asf1b | ASF1 anti-silencing function 1 homolog B (S. cerevisiae) | 66929 | ENSMUSG00000005470 |
| 46 | 17529231 | NA | Phip | pleckstrin homology domain interacting protein | 83946 | ENSMUSG00000032253 |
| 47 | 17527666 | NA | Islr2 | immunoglobulin superfamily containing leucine-rich repeat 2 | 320563 | ENSMUSG00000051243 |
| 48 | 17526273 | NA | Trappc4 | trafficking protein particle complex 4 | 60409 | ENSMUSG00000032112 |
| 49 | 17526271 | NA | Trappc4 | trafficking protein particle complex 4 | 60409 | ENSMUSG00000032112 |
| 50 | 17526272 | NA | Trappc4 | trafficking protein particle complex 4 | 60409 | ENSMUSG00000032112 |
| 51 | 17316625 | NA | Ubr5 | ubiquitin protein ligase E3 component n-recognin 5 | 70790 | ENSMUSG00000037487 |
| 52 | 17365493 | NA | Obfc1 | oligonucleotide/oligosaccharide-binding fold containing 1 | 108689 | ENSMUSG00000042694 |
| 53 | 17298407 | NA | Bap1 | Brca1 associated protein 1 | 104416 | ENSMUSG00000021901 |
| 54 | 17538790 | NA | Huwe1 | HECT, UBA and WWE domain containing 1 | 59026 | ENSMUSG00000025261 |
| 55 | 17359143 | NA | Lgi1 | leucine-rich repeat LGI family, member 1 | 56839 | ENSMUSG00000067242 |
| 56 | 17446580 | NA | Shh | sonic hedgehog | 20423 | ENSMUSG00000002633 |
| 57 | 17451443 | NA | Coro1c | coronin, actin binding protein 1C | 23790 | ENSMUSG00000004530 |
| 58 | 17221014 | NA | Cd34 | CD34 antigen | 12490 | ENSMUSG00000016494 |
| 59 | 17273280 | NA | Stra13 | stimulated by retinoic acid 13 | 20892 | ENSMUSG00000025144 |
| 60 | 17241409 | NA | Srgn | serglycin | 19073 | ENSMUSG00000020077 |
| 61 | 17420582 | NA | Capzb | capping protein (actin filament) muscle Z-line, beta | 12345 | ENSMUSG00000028745 |
| 62 | 17273086 | NA | Nploc4 | nuclear protein localization 4 homolog (S. cerevisiae) | 217365 | ENSMUSG00000039703 |
| 63 | 17212355 | NA | Nck2 | non-catalytic region of tyrosine kinase adaptor protein 2 | 17974 | ENSMUSG00000066877 |
| 64 | 17393357 | NA | Eif6 | eukaryotic translation initiation factor 6 | 16418 | ENSMUSG00000027613 |
| 65 | 17412593 | NA | Srsf12 | serine/arginine-rich splicing factor 12 | 272009 | ENSMUSG00000054679 |
| 66 | 17275955 | NA | Atl1 | atlastin GTPase 1 | 73991 | ENSMUSG00000021066 |
| 67 | 17303496 | NA | Fezf2 | Fez family zinc finger 2 | 54713 | ENSMUSG00000021743 |
| 68 | 17345519 | NA | Rrp36 | ribosomal RNA processing 36 homolog (S. cerevisiae) | 224823 | ENSMUSG00000023971 |
| 69 | 17256565 | NA | Tubg2 | tubulin, gamma 2 | 103768 | ENSMUSG00000045007 |
| 70 | 17506631 | NA | Tubb3 | tubulin, beta 3 class III | 22152 | ENSMUSG00000062380 |
| 71 | 17232649 | NA | Fyn | Fyn proto-oncogene | 14360 | ENSMUSG00000019843 |
| 72 | 17253674 | NA | Poldip2 | polymerase (DNA-directed), delta interacting protein 2 | 67811 | ENSMUSG00000001100 |
| 73 | 17395844 | NA | Stmn3 | stathmin-like 3 | 20262 | ENSMUSG00000027581 |
| 74 | 17338670 | NA | Fsd1 | fibronectin type 3 and SPRY domain-containing protein | 240121 | ENSMUSG00000011589 |
| 75 | 17235584 | NA | Dapk3 | death-associated protein kinase 3 | 13144 | ENSMUSG00000034974 |
| 76 | 17460879 | NA | Hdac11 | histone deacetylase 11 | 232232 | ENSMUSG00000034245 |
| 77 | 17494637 | NA | Mrpl17 | mitochondrial ribosomal protein L17 | 27397 | ENSMUSG00000030879 |
| 78 | 17517532 | NA | Isl2 | insulin related protein 2 (islet 2) | 104360 | ENSMUSG00000032318 |
| 79 | 17513871 | NA | Chmp1a | charged multivesicular body protein 1A | 234852 | ENSMUSG00000000743 |
| 80 | 17288616 | NA | Tppp | tubulin polymerization promoting protein | 72948 | ENSMUSG00000021573 |
| 81 | 17396024 | NA | Stmn2 | stathmin-like 2 | 20257 | ENSMUSG00000027500 |
| 82 | 17452552 | NA | Rhof | ras homolog gene family, member f | 23912 | ENSMUSG00000029449 |
| 83 | 17418447 | NA | Meaf6 | MYST/Esa1-associated factor 6 | 70088 | ENSMUSG00000028863 |
| 84 | 17269638 | NA | Rab5c | RAB5C, member RAS oncogene family | 19345 | ENSMUSG00000019173 |
| 85 | 17400638 | NA | Pex11b | peroxisomal biogenesis factor 11 beta | 18632 | ENSMUSG00000028102 |
| 86 | 17256549 | NA | Tubg1 | tubulin, gamma 1 | 103733 | ENSMUSG00000035198 |
| 87 | 17214293 | NA | Bcs1l | BCS1-like (yeast) | 66821 | ENSMUSG00000026172 |
| 88 | 17494081 | NA | Rhog | ras homolog gene family, member G | 56212 | ENSMUSG00000073982 |

  
  

| **Database:biological process      &nbspName:protein polymerization      &nbspID:GO:0051258** | | | | | | |
| --- | --- | --- | --- | --- | --- | --- |
| C=139; O=11; E=2.67; R=4.12; rawP=8.01e-05; adjP=0.0094 | | | | | | |
| Index | UserID | Value | Gene Symbol | Gene Name | EntrezGene | Ensembl |
| 1 | 17256565 | NA | Tubg2 | tubulin, gamma 2 | 103768 | ENSMUSG00000045007 |
| 2 | 17361988 | NA | Arl2 | ADP-ribosylation factor-like 2 | 56327 | ENSMUSG00000024944 |
| 3 | 17324664 | NA | Dlg1 | discs, large homolog 1 (Drosophila) | 13383 | ENSMUSG00000022770 |
| 4 | 17506631 | NA | Tubb3 | tubulin, beta 3 class III | 22152 | ENSMUSG00000062380 |
| 5 | 17396024 | NA | Stmn2 | stathmin-like 2 | 20257 | ENSMUSG00000027500 |
| 6 | 17288616 | NA | Tppp | tubulin polymerization promoting protein | 72948 | ENSMUSG00000021573 |
| 7 | 17224540 | NA | Tuba4a | tubulin, alpha 4A | 22145 | ENSMUSG00000026202 |
| 8 | 17256549 | NA | Tubg1 | tubulin, gamma 1 | 103733 | ENSMUSG00000035198 |
| 9 | 17420582 | NA | Capzb | capping protein (actin filament) muscle Z-line, beta | 12345 | ENSMUSG00000028745 |
| 10 | 17321467 | NA | Tuba1b | tubulin, alpha 1B | 22143 | ENSMUSG00000023004 |
| 11 | 17212355 | NA | Nck2 | non-catalytic region of tyrosine kinase adaptor protein 2 | 17974 | ENSMUSG00000066877 |

  
  

| **Database:biological process      &nbspName:nitrogen compound metabolic process      &nbspID:GO:0006807** | | | | | | |
| --- | --- | --- | --- | --- | --- | --- |
| C=4787; O=125; E=92.06; R=1.36; rawP=7.94e-05; adjP=0.0094 | | | | | | |
| Index | UserID | Value | Gene Symbol | Gene Name | EntrezGene | Ensembl |
| 1 | 17476273 | NA | Zfp382 | zinc finger protein 382 | 233060 | ENSMUSG00000074220 |
| 2 | 17274448 | NA | Cpsf3 | cleavage and polyadenylation specificity factor 3 | 54451 | ENSMUSG00000054309 |
| 3 | 17519868 | NA | Htr1b | 5-hydroxytryptamine (serotonin) receptor 1B | 15551 | ENSMUSG00000049511 |
| 4 | 17232843 | NA | Zbtb24 | zinc finger and BTB domain containing 24 | 268294 | ENSMUSG00000019826 |
| 5 | 17344794 | NA | Znrd1 | zinc ribbon domain containing, 1 | 66136 | ENSMUSG00000036315 |
| 6 | 17515170 | NA | Ilf3 | interleukin enhancer binding factor 3 | 16201 | ENSMUSG00000032178 |
| 7 | 17300261 | NA | Oxa1l | oxidase assembly 1-like | 69089 | ENSMUSG00000000959 |
| 8 | 17357213 | NA | Zbtb3 | zinc finger and BTB domain containing 3 | 75291 | ENSMUSG00000071661 |
| 9 | 17430853 | NA | Med18 | mediator of RNA polymerase II transcription, subunit 18 homolog (yeast) | 67219 | ENSMUSG00000066042 |
| 10 | 17512740 | NA | Nob1 | NIN1/RPN12 binding protein 1 homolog (S. cerevisiae) | 67619 | ENSMUSG00000003848 |
| 11 | 17224540 | NA | Tuba4a | tubulin, alpha 4A | 22145 | ENSMUSG00000026202 |
| 12 | 17225179 | NA | Pde6d | phosphodiesterase 6D, cGMP-specific, rod, delta | 18582 | ENSMUSG00000026239 |
| 13 | 17368171 | NA | Bmyc | brain expressed myelocytomatosis oncogene | 107771 | ENSMUSG00000049086 |
| 14 | 17350134 | NA | Pou4f3 | POU domain, class 4, transcription factor 3 | 18998 | ENSMUSG00000024497 |
| 15 | 17517723 | NA | Rpp25 | ribonuclease P 25 subunit (human) | 102614 | ENSMUSG00000062309 |
| 16 | 17512103 | NA | Got2 | glutamate oxaloacetate transaminase 2, mitochondrial | 14719 | ENSMUSG00000031672 |
| 17 | 17336829 | NA | Lsm2 | LSM2 homolog, U6 small nuclear RNA associated (S. cerevisiae) | 27756 | ENSMUSG00000007050 |
| 18 | 17361988 | NA | Arl2 | ADP-ribosylation factor-like 2 | 56327 | ENSMUSG00000024944 |
| 19 | 17288454 | NA | Irx4 | Iroquois related homeobox 4 (Drosophila) | 50916 | ENSMUSG00000021604 |
| 20 | 17502583 | NA | Mcm5 | minichromosome maintenance deficient 5, cell division cycle 46 (S. cerevisiae) | 17218 | ENSMUSG00000005410 |
| 21 | 17511296 | NA | Wdr83 | WD repeat domain containing 83 | 67836 | ENSMUSG00000005150 |
| 22 | 17322559 | NA | Hmox2 | heme oxygenase (decycling) 2 | 15369 | ENSMUSG00000004070 |
| 23 | 17539434 | NA | Ctps2 | cytidine 5'-triphosphate synthase 2 | 55936 | ENSMUSG00000031360 |
| 24 | 17341521 | NA | Thoc6 | THO complex 6 homolog (Drosophila) | 386612 | ENSMUSG00000041319 |
| 25 | 17250141 | NA | Zfp39 | zinc finger protein 39 | 22698 | ENSMUSG00000037001 |
| 26 | 17519718 | NA | Mto1 | mitochondrial translation optimization 1 homolog (S. cerevisiae) | 68291 | ENSMUSG00000032342 |
| 27 | 17521422 | NA | Hyal2 | hyaluronoglucosaminidase 2 | 15587 | ENSMUSG00000010047 |
| 28 | 17235714 | NA | Dohh | deoxyhypusine hydroxylase/monooxygenase | 102115 | ENSMUSG00000078440 |
| 29 | 17321467 | NA | Tuba1b | tubulin, alpha 1B | 22143 | ENSMUSG00000023004 |
| 30 | 17408684 | NA | Dclre1b | DNA cross-link repair 1B, PSO2 homolog (S. cerevisiae) | 140917 | ENSMUSG00000027845 |
| 31 | 17342015 | NA | Tbl3 | transducin (beta)-like 3 | 213773 | ENSMUSG00000040688 |
| 32 | 17447099 | NA | Ctbp1 | C-terminal binding protein 1 | 13016 | ENSMUSG00000037373 |
| 33 | 17447089 | NA | Ctbp1 | C-terminal binding protein 1 | 13016 | ENSMUSG00000037373 |
| 34 | 17447100 | NA | Ctbp1 | C-terminal binding protein 1 | 13016 | ENSMUSG00000037373 |
| 35 | 17447098 | NA | Ctbp1 | C-terminal binding protein 1 | 13016 | ENSMUSG00000037373 |
| 36 | 17343617 | NA | Rab11b | RAB11B, member RAS oncogene family | 19326 | ENSMUSG00000077450 |
| 37 | 17543988 | NA | Taf9b | TAF9B RNA polymerase II, TATA box binding protein (TBP)-associated factor | 407786 | ENSMUSG00000047242 |
| 38 | 17265082 | NA | Eif5a | eukaryotic translation initiation factor 5A | 276770 | ENSMUSG00000078812 |
| 39 | 17233630 | NA | Psap | prosaposin | 19156 | ENSMUSG00000004207 |
| 40 | 17391270 | NA | Kcnip3 | Kv channel interacting protein 3, calsenilin | 56461 | ENSMUSG00000079056 |
| 41 | 17488463 | NA | Med29 | mediator complex subunit 29 | 67224 | ENSMUSG00000003444 |
| 42 | 17520177 | NA | Mthfs | 5, 10-methenyltetrahydrofolate synthetase | 107885 | ENSMUSG00000066442 |
| 43 | 17460099 | NA | Vax2 | ventral anterior homeobox containing gene 2 | 24113 | ENSMUSG00000034777 |
| 44 | 17528778 | NA | Ccpg1 | cell cycle progression 1 | 72278 | ENSMUSG00000034563 |
| 45 | 17278188 | NA | Otub2 | OTU domain, ubiquitin aldehyde binding 2 | 68149 | ENSMUSG00000021203 |
| 46 | 17292107 | NA | Tbc1d7 | TBC1 domain family, member 7 | 67046 | ENSMUSG00000021368 |
| 47 | 17225499 | NA | Hes6 | hairy and enhancer of split 6 (Drosophila) | 55927 | ENSMUSG00000067071 |
| 48 | 17498239 | NA | Kcnq1ot1 | KCNQ1 overlapping transcript 1 | 63830 | NULL |
| 49 | 17312944 | NA | Polr2f | polymerase (RNA) II (DNA directed) polypeptide F | 69833 | ENSMUSG00000033020 |
| 50 | 17312939 | NA | Polr2f | polymerase (RNA) II (DNA directed) polypeptide F | 69833 | ENSMUSG00000033020 |
| 51 | 17312941 | NA | Polr2f | polymerase (RNA) II (DNA directed) polypeptide F | 69833 | ENSMUSG00000033020 |
| 52 | 17334722 | NA | Rpusd1 | RNA pseudouridylate synthase domain containing 1 | 106707 | ENSMUSG00000041199 |
| 53 | 17517576 | NA | Hmg20a | high mobility group 20A | 66867 | ENSMUSG00000032329 |
| 54 | 17512479 | NA | Acd | adrenocortical dysplasia | 497652 | ENSMUSG00000038000 |
| 55 | 17361855 | NA | Pola2 | polymerase (DNA directed), alpha 2 | 18969 | ENSMUSG00000024833 |
| 56 | 17429495 | NA | Nfyc | nuclear transcription factor-Y gamma | 18046 | ENSMUSG00000032897 |
| 57 | 17527532 | NA | Mpi | mannose phosphate isomerase | 110119 | ENSMUSG00000032306 |
| 58 | 17404011 | NA | Hey1 | hairy/enhancer-of-split related with YRPW motif 1 | 15213 | ENSMUSG00000040289 |
| 59 | 17252635 | NA | Shpk | sedoheptulokinase | 74637 | ENSMUSG00000005951 |
| 60 | 17512463 | NA | Atp6v0d1 | ATPase, H+ transporting, lysosomal V0 subunit D1 | 11972 | ENSMUSG00000013160 |
| 61 | 17512466 | NA | Atp6v0d1 | ATPase, H+ transporting, lysosomal V0 subunit D1 | 11972 | ENSMUSG00000013160 |
| 62 | 17212813 | NA | Mstn | myostatin | 17700 | ENSMUSG00000026100 |
| 63 | 17400222 | NA | Vps72 | vacuolar protein sorting 72 (yeast) | 21427 | ENSMUSG00000008958 |
| 64 | 17230945 | NA | Smyd2 | SET and MYND domain containing 2 | 226830 | ENSMUSG00000026603 |
| 65 | 17246850 | NA | Zmat5 | zinc finger, matrin type 5 | 67178 | ENSMUSG00000009076 |
| 66 | 17313008 | NA | Cby1 | chibby homolog 1 (Drosophila) | 73739 | ENSMUSG00000022428 |
| 67 | 17521448 | NA | Hyal3 | hyaluronoglucosaminidase 3 | 109685 | ENSMUSG00000036091 |
| 68 | 17342359 | NA | Haghl | hydroxyacylglutathione hydrolase-like | 68977 | ENSMUSG00000061046 |
| 69 | 17468113 | NA | Ino80b | INO80 complex subunit B | 70020 | ENSMUSG00000030034 |
| 70 | 17503023 | NA | Asf1b | ASF1 anti-silencing function 1 homolog B (S. cerevisiae) | 66929 | ENSMUSG00000005470 |
| 71 | 17283939 | NA | Wars | tryptophanyl-tRNA synthetase | 22375 | ENSMUSG00000021266 |
| 72 | 17283941 | NA | Wars | tryptophanyl-tRNA synthetase | 22375 | ENSMUSG00000021266 |
| 73 | 17283930 | NA | Wars | tryptophanyl-tRNA synthetase | 22375 | ENSMUSG00000021266 |
| 74 | 17283938 | NA | Wars | tryptophanyl-tRNA synthetase | 22375 | ENSMUSG00000021266 |
| 75 | 17258457 | NA | Sap30bp | SAP30 binding protein | 57230 | ENSMUSG00000020755 |
| 76 | 17529231 | NA | Phip | pleckstrin homology domain interacting protein | 83946 | ENSMUSG00000032253 |
| 77 | 17454256 | NA | Taf6 | TAF6 RNA polymerase II, TATA box binding protein (TBP)-associated factor | 21343 | ENSMUSG00000036980 |
| 78 | 17510365 | NA | Pgls | 6-phosphogluconolactonase | 66171 | ENSMUSG00000031807 |
| 79 | 17316625 | NA | Ubr5 | ubiquitin protein ligase E3 component n-recognin 5 | 70790 | ENSMUSG00000037487 |
| 80 | 17512009 | NA | Csnk2a2 | casein kinase 2, alpha prime polypeptide | 13000 | ENSMUSG00000046707 |
| 81 | 17503816 | NA | Irx6 | Iroquois related homeobox 6 (Drosophila) | 64379 | ENSMUSG00000031738 |
| 82 | 17260221 | NA | Pold2 | polymerase (DNA directed), delta 2, regulatory subunit | 18972 | ENSMUSG00000020471 |
| 83 | 17365493 | NA | Obfc1 | oligonucleotide/oligosaccharide-binding fold containing 1 | 108689 | ENSMUSG00000042694 |
| 84 | 17231118 | NA | Rcor3 | REST corepressor 3 | 214742 | ENSMUSG00000037395 |
| 85 | 17538790 | NA | Huwe1 | HECT, UBA and WWE domain containing 1 | 59026 | ENSMUSG00000025261 |
| 86 | 17446580 | NA | Shh | sonic hedgehog | 20423 | ENSMUSG00000002633 |
| 87 | 17265186 | NA | Rnasek | ribonuclease, RNase K | 52898 | ENSMUSG00000093989 |
| 88 | 17470879 | NA | Tpi1 | triosephosphate isomerase 1 | 21991 | ENSMUSG00000023456 |
| 89 | 17281971 | NA | Sgpp1 | sphingosine-1-phosphate phosphatase 1 | 81535 | ENSMUSG00000021054 |
| 90 | 17221014 | NA | Cd34 | CD34 antigen | 12490 | ENSMUSG00000016494 |
| 91 | 17273280 | NA | Stra13 | stimulated by retinoic acid 13 | 20892 | ENSMUSG00000025144 |
| 92 | 17301342 | NA | Ints9 | integrator complex subunit 9 | 210925 | ENSMUSG00000021975 |
| 93 | 17238549 | NA | Wibg | within bgcn homolog (Drosophila) | 78428 | ENSMUSG00000064030 |
| 94 | 17212355 | NA | Nck2 | non-catalytic region of tyrosine kinase adaptor protein 2 | 17974 | ENSMUSG00000066877 |
| 95 | 17412593 | NA | Srsf12 | serine/arginine-rich splicing factor 12 | 272009 | ENSMUSG00000054679 |
| 96 | 17213990 | NA | Atic | 5-aminoimidazole-4-carboxamide ribonucleotide formyltransferase/IMP cyclohydrolase | 108147 | ENSMUSG00000026192 |
| 97 | 17303496 | NA | Fezf2 | Fez family zinc finger 2 | 54713 | ENSMUSG00000021743 |
| 98 | 17504160 | NA | Polr2c | polymerase (RNA) II (DNA directed) polypeptide C | 20021 | ENSMUSG00000031783 |
| 99 | 17455093 | NA | Zkscan14 | zinc finger with KRAB and SCAN domains 14 | 67235 | ENSMUSG00000029627 |
| 100 | 17508691 | NA | Rbpms | RNA binding protein gene with multiple splicing | 19663 | ENSMUSG00000031586 |
| 101 | 17256565 | NA | Tubg2 | tubulin, gamma 2 | 103768 | ENSMUSG00000045007 |
| 102 | 17345519 | NA | Rrp36 | ribosomal RNA processing 36 homolog (S. cerevisiae) | 224823 | ENSMUSG00000023971 |
| 103 | 17463422 | NA | Nrip2 | nuclear receptor interacting protein 2 | 60345 | ENSMUSG00000001520 |
| 104 | 17211335 | NA | Tfap2d | transcription factor AP-2, delta | 226896 | ENSMUSG00000042596 |
| 105 | 17222001 | NA | Prim2 | DNA primase, p58 subunit | 19076 | ENSMUSG00000026134 |
| 106 | 17506631 | NA | Tubb3 | tubulin, beta 3 class III | 22152 | ENSMUSG00000062380 |
| 107 | 17328810 | NA | Dgcr14 | DiGeorge syndrome critical region gene 14 | 27886 | ENSMUSG00000003527 |
| 108 | 17395844 | NA | Stmn3 | stathmin-like 3 | 20262 | ENSMUSG00000027581 |
| 109 | 17321722 | NA | Tfcp2 | transcription factor CP2 | 21422 | ENSMUSG00000009733 |
| 110 | 17213153 | NA | Nif3l1 | Ngg1 interacting factor 3-like 1 (S. pombe) | 65102 | ENSMUSG00000026036 |
| 111 | 17235584 | NA | Dapk3 | death-associated protein kinase 3 | 13144 | ENSMUSG00000034974 |
| 112 | 17219286 | NA | Dedd | death effector domain-containing | 21945 | ENSMUSG00000013973 |
| 113 | 17477670 | NA | Rras | Harvey rat sarcoma oncogene, subgroup R | 20130 | ENSMUSG00000038387 |
| 114 | 17300411 | NA | Thtpa | thiamine triphosphatase | 105663 | ENSMUSG00000045691 |
| 115 | 17498821 | NA | Snapc2 | small nuclear RNA activating complex, polypeptide 2 | 102209 | ENSMUSG00000011837 |
| 116 | 17460879 | NA | Hdac11 | histone deacetylase 11 | 232232 | ENSMUSG00000034245 |
| 117 | 17256618 | NA | Vps25 | vacuolar protein sorting 25 (yeast) | 28084 | ENSMUSG00000078656 |
| 118 | 17444100 | NA | Chst12 | carbohydrate sulfotransferase 12 | 59031 | ENSMUSG00000036599 |
| 119 | 17517532 | NA | Isl2 | insulin related protein 2 (islet 2) | 104360 | ENSMUSG00000032318 |
| 120 | 17513871 | NA | Chmp1a | charged multivesicular body protein 1A | 234852 | ENSMUSG00000000743 |
| 121 | 17359994 | NA | Fbxl15 | F-box and leucine-rich repeat protein 15 | 68431 | ENSMUSG00000025226 |
| 122 | 17334495 | NA | Nme3 | NME/NM23 nucleoside diphosphate kinase 3 | 79059 | ENSMUSG00000073435 |
| 123 | 17497366 | NA | Ebf3 | early B cell factor 3 | 13593 | ENSMUSG00000010476 |
| 124 | 17383588 | NA | Ccbl1 | cysteine conjugate-beta lyase 1 | 70266 | ENSMUSG00000039648 |
| 125 | 17313199 | NA | Adsl | adenylosuccinate lyase | 11564 | ENSMUSG00000022407 |
| 126 | 17232215 | NA | Moxd1 | monooxygenase, DBH-like 1 | 59012 | ENSMUSG00000020000 |
| 127 | 17418447 | NA | Meaf6 | MYST/Esa1-associated factor 6 | 70088 | ENSMUSG00000028863 |
| 128 | 17269638 | NA | Rab5c | RAB5C, member RAS oncogene family | 19345 | ENSMUSG00000019173 |
| 129 | 17356202 | NA | Pold4 | polymerase (DNA-directed), delta 4 | 69745 | ENSMUSG00000024854 |
| 130 | 17505367 | NA | Txnl4b | thioredoxin-like 4B | 234723 | ENSMUSG00000031723 |
| 131 | 17256549 | NA | Tubg1 | tubulin, gamma 1 | 103733 | ENSMUSG00000035198 |
| 132 | 17498370 | NA | Nadsyn1 | NAD synthetase 1 | 78914 | ENSMUSG00000031090 |
| 133 | 17494081 | NA | Rhog | ras homolog gene family, member G | 56212 | ENSMUSG00000073982 |
| 134 | 17497957 | NA | Chid1 | chitinase domain containing 1 | 68038 | ENSMUSG00000025512 |

  
  

| **Database:biological process      &nbspName:cellular macromolecular complex subunit organization      &nbspID:GO:0034621** | | | | | | |
| --- | --- | --- | --- | --- | --- | --- |
| C=475; O=23; E=9.13; R=2.52; rawP=5.29e-05; adjP=0.0094 | | | | | | |
| Index | UserID | Value | Gene Symbol | Gene Name | EntrezGene | Ensembl |
| 1 | 17263594 | NA | Atpaf2 | ATP synthase mitochondrial F1 complex assembly factor 2 | 246782 | ENSMUSG00000042709 |
| 2 | 17235268 | NA | Ndufs7 | NADH dehydrogenase (ubiquinone) Fe-S protein 7 | 75406 | ENSMUSG00000020153 |
| 3 | 17413221 | NA | Unc13b | unc-13 homolog B (C. elegans) | 22249 | ENSMUSG00000028456 |
| 4 | 17256565 | NA | Tubg2 | tubulin, gamma 2 | 103768 | ENSMUSG00000045007 |
| 5 | 17324664 | NA | Dlg1 | discs, large homolog 1 (Drosophila) | 13383 | ENSMUSG00000022770 |
| 6 | 17506631 | NA | Tubb3 | tubulin, beta 3 class III | 22152 | ENSMUSG00000062380 |
| 7 | 17265082 | NA | Eif5a | eukaryotic translation initiation factor 5A | 276770 | ENSMUSG00000078812 |
| 8 | 17300261 | NA | Oxa1l | oxidase assembly 1-like | 69089 | ENSMUSG00000000959 |
| 9 | 17503023 | NA | Asf1b | ASF1 anti-silencing function 1 homolog B (S. cerevisiae) | 66929 | ENSMUSG00000005470 |
| 10 | 17224540 | NA | Tuba4a | tubulin, alpha 4A | 22145 | ENSMUSG00000026202 |
| 11 | 17336829 | NA | Lsm2 | LSM2 homolog, U6 small nuclear RNA associated (S. cerevisiae) | 27756 | ENSMUSG00000007050 |
| 12 | 17370285 | NA | Mrrf | mitochondrial ribosome recycling factor | 67871 | ENSMUSG00000026887 |
| 13 | 17361988 | NA | Arl2 | ADP-ribosylation factor-like 2 | 56327 | ENSMUSG00000024944 |
| 14 | 17288616 | NA | Tppp | tubulin polymerization promoting protein | 72948 | ENSMUSG00000021573 |
| 15 | 17396024 | NA | Stmn2 | stathmin-like 2 | 20257 | ENSMUSG00000027500 |
| 16 | 17266322 | NA | Eral1 | Era (G-protein)-like 1 (E. coli) | 57837 | ENSMUSG00000020832 |
| 17 | 17256549 | NA | Tubg1 | tubulin, gamma 1 | 103733 | ENSMUSG00000035198 |
| 18 | 17420582 | NA | Capzb | capping protein (actin filament) muscle Z-line, beta | 12345 | ENSMUSG00000028745 |
| 19 | 17214293 | NA | Bcs1l | BCS1-like (yeast) | 66821 | ENSMUSG00000026172 |
| 20 | 17321467 | NA | Tuba1b | tubulin, alpha 1B | 22143 | ENSMUSG00000023004 |
| 21 | 17212355 | NA | Nck2 | non-catalytic region of tyrosine kinase adaptor protein 2 | 17974 | ENSMUSG00000066877 |
| 22 | 17393357 | NA | Eif6 | eukaryotic translation initiation factor 6 | 16418 | ENSMUSG00000027613 |
| 23 | 17412593 | NA | Srsf12 | serine/arginine-rich splicing factor 12 | 272009 | ENSMUSG00000054679 |

  
  

| **Database:biological process      &nbspName:cellular macromolecular complex assembly      &nbspID:GO:0034622** | | | | | | |
| --- | --- | --- | --- | --- | --- | --- |
| C=409; O=21; E=7.87; R=2.67; rawP=4.86e-05; adjP=0.0094 | | | | | | |
| Index | UserID | Value | Gene Symbol | Gene Name | EntrezGene | Ensembl |
| 1 | 17263594 | NA | Atpaf2 | ATP synthase mitochondrial F1 complex assembly factor 2 | 246782 | ENSMUSG00000042709 |
| 2 | 17235268 | NA | Ndufs7 | NADH dehydrogenase (ubiquinone) Fe-S protein 7 | 75406 | ENSMUSG00000020153 |
| 3 | 17413221 | NA | Unc13b | unc-13 homolog B (C. elegans) | 22249 | ENSMUSG00000028456 |
| 4 | 17256565 | NA | Tubg2 | tubulin, gamma 2 | 103768 | ENSMUSG00000045007 |
| 5 | 17324664 | NA | Dlg1 | discs, large homolog 1 (Drosophila) | 13383 | ENSMUSG00000022770 |
| 6 | 17506631 | NA | Tubb3 | tubulin, beta 3 class III | 22152 | ENSMUSG00000062380 |
| 7 | 17300261 | NA | Oxa1l | oxidase assembly 1-like | 69089 | ENSMUSG00000000959 |
| 8 | 17503023 | NA | Asf1b | ASF1 anti-silencing function 1 homolog B (S. cerevisiae) | 66929 | ENSMUSG00000005470 |
| 9 | 17224540 | NA | Tuba4a | tubulin, alpha 4A | 22145 | ENSMUSG00000026202 |
| 10 | 17336829 | NA | Lsm2 | LSM2 homolog, U6 small nuclear RNA associated (S. cerevisiae) | 27756 | ENSMUSG00000007050 |
| 11 | 17361988 | NA | Arl2 | ADP-ribosylation factor-like 2 | 56327 | ENSMUSG00000024944 |
| 12 | 17288616 | NA | Tppp | tubulin polymerization promoting protein | 72948 | ENSMUSG00000021573 |
| 13 | 17396024 | NA | Stmn2 | stathmin-like 2 | 20257 | ENSMUSG00000027500 |
| 14 | 17266322 | NA | Eral1 | Era (G-protein)-like 1 (E. coli) | 57837 | ENSMUSG00000020832 |
| 15 | 17256549 | NA | Tubg1 | tubulin, gamma 1 | 103733 | ENSMUSG00000035198 |
| 16 | 17420582 | NA | Capzb | capping protein (actin filament) muscle Z-line, beta | 12345 | ENSMUSG00000028745 |
| 17 | 17214293 | NA | Bcs1l | BCS1-like (yeast) | 66821 | ENSMUSG00000026172 |
| 18 | 17321467 | NA | Tuba1b | tubulin, alpha 1B | 22143 | ENSMUSG00000023004 |
| 19 | 17212355 | NA | Nck2 | non-catalytic region of tyrosine kinase adaptor protein 2 | 17974 | ENSMUSG00000066877 |
| 20 | 17393357 | NA | Eif6 | eukaryotic translation initiation factor 6 | 16418 | ENSMUSG00000027613 |
| 21 | 17412593 | NA | Srsf12 | serine/arginine-rich splicing factor 12 | 272009 | ENSMUSG00000054679 |

  
  

| **Database:molecular function      &nbspName:guanyl ribonucleotide binding      &nbspID:GO:0032561** | | | | | | |
| --- | --- | --- | --- | --- | --- | --- |
| C=349; O=23; E=6.82; R=3.37; rawP=4.31e-07; adjP=3.92e-05 | | | | | | |
| Index | UserID | Value | Gene Symbol | Gene Name | EntrezGene | Ensembl |
| 1 | 17343617 | NA | Rab11b | RAB11B, member RAS oncogene family | 19326 | ENSMUSG00000077450 |
| 2 | 17256565 | NA | Tubg2 | tubulin, gamma 2 | 103768 | ENSMUSG00000045007 |
| 3 | 17502390 | NA | Rab8a | RAB8A, member RAS oncogene family | 17274 | ENSMUSG00000003037 |
| 4 | 17506631 | NA | Tubb3 | tubulin, beta 3 class III | 22152 | ENSMUSG00000062380 |
| 5 | 17282074 | NA | Rab15 | RAB15, member RAS oncogene family | 104886 | ENSMUSG00000021062 |
| 6 | 17509721 | NA | Tufm | Tu translation elongation factor, mitochondrial | 233870 | ENSMUSG00000073838 |
| 7 | 17443461 | NA | Rabl5 | RAB, member of RAS oncogene family-like 5 | 67286 | ENSMUSG00000007987 |
| 8 | 17361463 | NA | Rab1b | RAB1B, member RAS oncogene family | 76308 | ENSMUSG00000024870 |
| 9 | 17217580 | NA | Arl8a | ADP-ribosylation factor-like 8A | 68724 | ENSMUSG00000026426 |
| 10 | 17224540 | NA | Tuba4a | tubulin, alpha 4A | 22145 | ENSMUSG00000026202 |
| 11 | 17477670 | NA | Rras | Harvey rat sarcoma oncogene, subgroup R | 20130 | ENSMUSG00000038387 |
| 12 | 17277370 | NA | Eif2b2 | eukaryotic translation initiation factor 2B, subunit 2 beta | 217715 | ENSMUSG00000004788 |
| 13 | 17534615 | NA | Rab33a | RAB33A, member of RAS oncogene family | 19337 | ENSMUSG00000031104 |
| 14 | 17361988 | NA | Arl2 | ADP-ribosylation factor-like 2 | 56327 | ENSMUSG00000024944 |
| 15 | 17452552 | NA | Rhof | ras homolog gene family, member f | 23912 | ENSMUSG00000029449 |
| 16 | 17269638 | NA | Rab5c | RAB5C, member RAS oncogene family | 19345 | ENSMUSG00000019173 |
| 17 | 17266322 | NA | Eral1 | Era (G-protein)-like 1 (E. coli) | 57837 | ENSMUSG00000020832 |
| 18 | 17488134 | NA | Rab4b | RAB4B, member RAS oncogene family | 19342 | ENSMUSG00000053291 |
| 19 | 17256549 | NA | Tubg1 | tubulin, gamma 1 | 103733 | ENSMUSG00000035198 |
| 20 | 17321467 | NA | Tuba1b | tubulin, alpha 1B | 22143 | ENSMUSG00000023004 |
| 21 | 17494081 | NA | Rhog | ras homolog gene family, member G | 56212 | ENSMUSG00000073982 |
| 22 | 17540982 | NA | Sept6 | septin 6 | 56526 | ENSMUSG00000050379 |
| 23 | 17275955 | NA | Atl1 | atlastin GTPase 1 | 73991 | ENSMUSG00000021066 |

  
  

| **Database:molecular function      &nbspName:GTP binding      &nbspID:GO:0005525** | | | | | | |
| --- | --- | --- | --- | --- | --- | --- |
| C=333; O=23; E=6.51; R=3.54; rawP=1.88e-07; adjP=3.92e-05 | | | | | | |
| Index | UserID | Value | Gene Symbol | Gene Name | EntrezGene | Ensembl |
| 1 | 17343617 | NA | Rab11b | RAB11B, member RAS oncogene family | 19326 | ENSMUSG00000077450 |
| 2 | 17256565 | NA | Tubg2 | tubulin, gamma 2 | 103768 | ENSMUSG00000045007 |
| 3 | 17502390 | NA | Rab8a | RAB8A, member RAS oncogene family | 17274 | ENSMUSG00000003037 |
| 4 | 17506631 | NA | Tubb3 | tubulin, beta 3 class III | 22152 | ENSMUSG00000062380 |
| 5 | 17282074 | NA | Rab15 | RAB15, member RAS oncogene family | 104886 | ENSMUSG00000021062 |
| 6 | 17509721 | NA | Tufm | Tu translation elongation factor, mitochondrial | 233870 | ENSMUSG00000073838 |
| 7 | 17443461 | NA | Rabl5 | RAB, member of RAS oncogene family-like 5 | 67286 | ENSMUSG00000007987 |
| 8 | 17361463 | NA | Rab1b | RAB1B, member RAS oncogene family | 76308 | ENSMUSG00000024870 |
| 9 | 17217580 | NA | Arl8a | ADP-ribosylation factor-like 8A | 68724 | ENSMUSG00000026426 |
| 10 | 17224540 | NA | Tuba4a | tubulin, alpha 4A | 22145 | ENSMUSG00000026202 |
| 11 | 17477670 | NA | Rras | Harvey rat sarcoma oncogene, subgroup R | 20130 | ENSMUSG00000038387 |
| 12 | 17277370 | NA | Eif2b2 | eukaryotic translation initiation factor 2B, subunit 2 beta | 217715 | ENSMUSG00000004788 |
| 13 | 17534615 | NA | Rab33a | RAB33A, member of RAS oncogene family | 19337 | ENSMUSG00000031104 |
| 14 | 17361988 | NA | Arl2 | ADP-ribosylation factor-like 2 | 56327 | ENSMUSG00000024944 |
| 15 | 17452552 | NA | Rhof | ras homolog gene family, member f | 23912 | ENSMUSG00000029449 |
| 16 | 17269638 | NA | Rab5c | RAB5C, member RAS oncogene family | 19345 | ENSMUSG00000019173 |
| 17 | 17266322 | NA | Eral1 | Era (G-protein)-like 1 (E. coli) | 57837 | ENSMUSG00000020832 |
| 18 | 17488134 | NA | Rab4b | RAB4B, member RAS oncogene family | 19342 | ENSMUSG00000053291 |
| 19 | 17256549 | NA | Tubg1 | tubulin, gamma 1 | 103733 | ENSMUSG00000035198 |
| 20 | 17321467 | NA | Tuba1b | tubulin, alpha 1B | 22143 | ENSMUSG00000023004 |
| 21 | 17494081 | NA | Rhog | ras homolog gene family, member G | 56212 | ENSMUSG00000073982 |
| 22 | 17540982 | NA | Sept6 | septin 6 | 56526 | ENSMUSG00000050379 |
| 23 | 17275955 | NA | Atl1 | atlastin GTPase 1 | 73991 | ENSMUSG00000021066 |

  
  

| **Database:molecular function      &nbspName:guanyl nucleotide binding      &nbspID:GO:0019001** | | | | | | |
| --- | --- | --- | --- | --- | --- | --- |
| C=349; O=23; E=6.82; R=3.37; rawP=4.31e-07; adjP=3.92e-05 | | | | | | |
| Index | UserID | Value | Gene Symbol | Gene Name | EntrezGene | Ensembl |
| 1 | 17343617 | NA | Rab11b | RAB11B, member RAS oncogene family | 19326 | ENSMUSG00000077450 |
| 2 | 17256565 | NA | Tubg2 | tubulin, gamma 2 | 103768 | ENSMUSG00000045007 |
| 3 | 17502390 | NA | Rab8a | RAB8A, member RAS oncogene family | 17274 | ENSMUSG00000003037 |
| 4 | 17506631 | NA | Tubb3 | tubulin, beta 3 class III | 22152 | ENSMUSG00000062380 |
| 5 | 17282074 | NA | Rab15 | RAB15, member RAS oncogene family | 104886 | ENSMUSG00000021062 |
| 6 | 17509721 | NA | Tufm | Tu translation elongation factor, mitochondrial | 233870 | ENSMUSG00000073838 |
| 7 | 17443461 | NA | Rabl5 | RAB, member of RAS oncogene family-like 5 | 67286 | ENSMUSG00000007987 |
| 8 | 17361463 | NA | Rab1b | RAB1B, member RAS oncogene family | 76308 | ENSMUSG00000024870 |
| 9 | 17217580 | NA | Arl8a | ADP-ribosylation factor-like 8A | 68724 | ENSMUSG00000026426 |
| 10 | 17224540 | NA | Tuba4a | tubulin, alpha 4A | 22145 | ENSMUSG00000026202 |
| 11 | 17477670 | NA | Rras | Harvey rat sarcoma oncogene, subgroup R | 20130 | ENSMUSG00000038387 |
| 12 | 17277370 | NA | Eif2b2 | eukaryotic translation initiation factor 2B, subunit 2 beta | 217715 | ENSMUSG00000004788 |
| 13 | 17534615 | NA | Rab33a | RAB33A, member of RAS oncogene family | 19337 | ENSMUSG00000031104 |
| 14 | 17361988 | NA | Arl2 | ADP-ribosylation factor-like 2 | 56327 | ENSMUSG00000024944 |
| 15 | 17452552 | NA | Rhof | ras homolog gene family, member f | 23912 | ENSMUSG00000029449 |
| 16 | 17269638 | NA | Rab5c | RAB5C, member RAS oncogene family | 19345 | ENSMUSG00000019173 |
| 17 | 17266322 | NA | Eral1 | Era (G-protein)-like 1 (E. coli) | 57837 | ENSMUSG00000020832 |
| 18 | 17488134 | NA | Rab4b | RAB4B, member RAS oncogene family | 19342 | ENSMUSG00000053291 |
| 19 | 17256549 | NA | Tubg1 | tubulin, gamma 1 | 103733 | ENSMUSG00000035198 |
| 20 | 17321467 | NA | Tuba1b | tubulin, alpha 1B | 22143 | ENSMUSG00000023004 |
| 21 | 17494081 | NA | Rhog | ras homolog gene family, member G | 56212 | ENSMUSG00000073982 |
| 22 | 17540982 | NA | Sept6 | septin 6 | 56526 | ENSMUSG00000050379 |
| 23 | 17275955 | NA | Atl1 | atlastin GTPase 1 | 73991 | ENSMUSG00000021066 |

  
  

| **Database:molecular function      &nbspName:GTPase activity      &nbspID:GO:0003924** | | | | | | |
| --- | --- | --- | --- | --- | --- | --- |
| C=168; O=12; E=3.28; R=3.66; rawP=0.0001; adjP=0.0068 | | | | | | |
| Index | UserID | Value | Gene Symbol | Gene Name | EntrezGene | Ensembl |
| 1 | 17343617 | NA | Rab11b | RAB11B, member RAS oncogene family | 19326 | ENSMUSG00000077450 |
| 2 | 17256565 | NA | Tubg2 | tubulin, gamma 2 | 103768 | ENSMUSG00000045007 |
| 3 | 17361988 | NA | Arl2 | ADP-ribosylation factor-like 2 | 56327 | ENSMUSG00000024944 |
| 4 | 17506631 | NA | Tubb3 | tubulin, beta 3 class III | 22152 | ENSMUSG00000062380 |
| 5 | 17509721 | NA | Tufm | Tu translation elongation factor, mitochondrial | 233870 | ENSMUSG00000073838 |
| 6 | 17269638 | NA | Rab5c | RAB5C, member RAS oncogene family | 19345 | ENSMUSG00000019173 |
| 7 | 17224540 | NA | Tuba4a | tubulin, alpha 4A | 22145 | ENSMUSG00000026202 |
| 8 | 17256549 | NA | Tubg1 | tubulin, gamma 1 | 103733 | ENSMUSG00000035198 |
| 9 | 17477670 | NA | Rras | Harvey rat sarcoma oncogene, subgroup R | 20130 | ENSMUSG00000038387 |
| 10 | 17321467 | NA | Tuba1b | tubulin, alpha 1B | 22143 | ENSMUSG00000023004 |
| 11 | 17494081 | NA | Rhog | ras homolog gene family, member G | 56212 | ENSMUSG00000073982 |
| 12 | 17275955 | NA | Atl1 | atlastin GTPase 1 | 73991 | ENSMUSG00000021066 |

  
  

| **Database:molecular function      &nbspName:C-8 sterol isomerase activity      &nbspID:GO:0000247** | | | | | | |
| --- | --- | --- | --- | --- | --- | --- |
| C=2; O=2; E=0.04; R=51.19; rawP=0.0004; adjP=0.0137 | | | | | | |
| Index | UserID | Value | Gene Symbol | Gene Name | EntrezGene | Ensembl |
| 1 | 17540050 | NA | Ebp | phenylalkylamine Ca2+ antagonist (emopamil) binding protein | 13595 | ENSMUSG00000031168 |
| 2 | 17424319 | NA | Sigmar1 | sigma non-opioid intracellular receptor 1 | 18391 | ENSMUSG00000036078 |

  
  

| **Database:molecular function      &nbspName:peptide-methionine (S)-S-oxide reductase activity      &nbspID:GO:0008113** | | | | | | |
| --- | --- | --- | --- | --- | --- | --- |
| C=2; O=2; E=0.04; R=51.19; rawP=0.0004; adjP=0.0137 | | | | | | |
| Index | UserID | Value | Gene Symbol | Gene Name | EntrezGene | Ensembl |
| 1 | 17307695 | NA | Msra | methionine sulfoxide reductase A | 110265 | ENSMUSG00000054733 |
| 2 | 17318877 | NA | Txn2 | thioredoxin 2 | 56551 | ENSMUSG00000005354 |

  
  

| **Database:molecular function      &nbspName:binding      &nbspID:GO:0005488** | | | | | | |
| --- | --- | --- | --- | --- | --- | --- |
| C=10559; O=240; E=206.27; R=1.16; rawP=0.0003; adjP=0.0137 | | | | | | |
| Index | UserID | Value | Gene Symbol | Gene Name | EntrezGene | Ensembl |
| 1 | 17476273 | NA | Zfp382 | zinc finger protein 382 | 233060 | ENSMUSG00000074220 |
| 2 | 17380060 | NA | Dok5 | docking protein 5 | 76829 | ENSMUSG00000027560 |
| 3 | 17413221 | NA | Unc13b | unc-13 homolog B (C. elegans) | 22249 | ENSMUSG00000028456 |
| 4 | 17257962 | NA | Sstr2 | somatostatin receptor 2 | 20606 | ENSMUSG00000047904 |
| 5 | 17232843 | NA | Zbtb24 | zinc finger and BTB domain containing 24 | 268294 | ENSMUSG00000019826 |
| 6 | 17344794 | NA | Znrd1 | zinc ribbon domain containing, 1 | 66136 | ENSMUSG00000036315 |
| 7 | 17344538 | NA | Prr3 | proline-rich polypeptide 3 | 75210 | ENSMUSG00000038500 |
| 8 | 17300261 | NA | Oxa1l | oxidase assembly 1-like | 69089 | ENSMUSG00000000959 |
| 9 | 17512740 | NA | Nob1 | NIN1/RPN12 binding protein 1 homolog (S. cerevisiae) | 67619 | ENSMUSG00000003848 |
| 10 | 17224540 | NA | Tuba4a | tubulin, alpha 4A | 22145 | ENSMUSG00000026202 |
| 11 | 17503910 | NA | Ogfod1 | 2-oxoglutarate and iron-dependent oxygenase domain containing 1 | 270086 | ENSMUSG00000033009 |
| 12 | 17456308 | NA | Kcnd2 | potassium voltage-gated channel, Shal-related family, member 2 | 16508 | ENSMUSG00000060882 |
| 13 | 17368171 | NA | Bmyc | brain expressed myelocytomatosis oncogene | 107771 | ENSMUSG00000049086 |
| 14 | 17350134 | NA | Pou4f3 | POU domain, class 4, transcription factor 3 | 18998 | ENSMUSG00000024497 |
| 15 | 17517723 | NA | Rpp25 | ribonuclease P 25 subunit (human) | 102614 | ENSMUSG00000062309 |
| 16 | 17460275 | NA | Smyd5 | SET and MYND domain containing 5 | 232187 | ENSMUSG00000033706 |
| 17 | 17307134 | NA | Cryl1 | crystallin, lambda 1 | 68631 | ENSMUSG00000021947 |
| 18 | 17443181 | NA | Dnajc30 | DnaJ (Hsp40) homolog, subfamily C, member 30 | 66114 | ENSMUSG00000061118 |
| 19 | 17512103 | NA | Got2 | glutamate oxaloacetate transaminase 2, mitochondrial | 14719 | ENSMUSG00000031672 |
| 20 | 17369862 | NA | Dpm2 | dolichol-phosphate (beta-D) mannosyltransferase 2 | 13481 | ENSMUSG00000026810 |
| 21 | 17336829 | NA | Lsm2 | LSM2 homolog, U6 small nuclear RNA associated (S. cerevisiae) | 27756 | ENSMUSG00000007050 |
| 22 | 17288454 | NA | Irx4 | Iroquois related homeobox 4 (Drosophila) | 50916 | ENSMUSG00000021604 |
| 23 | 17322559 | NA | Hmox2 | heme oxygenase (decycling) 2 | 15369 | ENSMUSG00000004070 |
| 24 | 17537677 | NA | Drp2 | dystrophin related protein 2 | 13497 | ENSMUSG00000000223 |
| 25 | 17328225 | NA | Cpped1 | calcineurin-like phosphoesterase domain containing 1 | 223978 | ENSMUSG00000065979 |
| 26 | 17404329 | NA | Gyg | glycogenin | 27357 | ENSMUSG00000019528 |
| 27 | 17504712 | NA | Exoc3l | exocyst complex component 3-like | 277978 | ENSMUSG00000043251 |
| 28 | 17321467 | NA | Tuba1b | tubulin, alpha 1B | 22143 | ENSMUSG00000023004 |
| 29 | 17457310 | NA | Chrm2 | cholinergic receptor, muscarinic 2, cardiac | 243764 | ENSMUSG00000045613 |
| 30 | 17288160 | NA | Cdk20 | cyclin-dependent kinase 20 | 105278 | ENSMUSG00000021483 |
| 31 | 17538096 | NA | Rnf128 | ring finger protein 128 | 66889 | ENSMUSG00000031438 |
| 32 | 17448565 | NA | Gabrg1 | gamma-aminobutyric acid (GABA) A receptor, subunit gamma 1 | 14405 | ENSMUSG00000001260 |
| 33 | 17447099 | NA | Ctbp1 | C-terminal binding protein 1 | 13016 | ENSMUSG00000037373 |
| 34 | 17447089 | NA | Ctbp1 | C-terminal binding protein 1 | 13016 | ENSMUSG00000037373 |
| 35 | 17447100 | NA | Ctbp1 | C-terminal binding protein 1 | 13016 | ENSMUSG00000037373 |
| 36 | 17447098 | NA | Ctbp1 | C-terminal binding protein 1 | 13016 | ENSMUSG00000037373 |
| 37 | 17343617 | NA | Rab11b | RAB11B, member RAS oncogene family | 19326 | ENSMUSG00000077450 |
| 38 | 17246284 | NA | Suox | sulfite oxidase | 211389 | ENSMUSG00000049858 |
| 39 | 17437611 | NA | Pgm1 | phosphoglucomutase 1 | 66681 | ENSMUSG00000029171 |
| 40 | 17287579 | NA | Zfp346 | zinc finger protein 346 | 26919 | ENSMUSG00000021481 |
| 41 | 17265082 | NA | Eif5a | eukaryotic translation initiation factor 5A | 276770 | ENSMUSG00000078812 |
| 42 | 17433602 | NA | Tprgl | transformation related protein 63 regulated like | 67808 | ENSMUSG00000029030 |
| 43 | 17297750 | NA | Ppif | peptidylprolyl isomerase F (cyclophilin F) | 105675 | ENSMUSG00000021868 |
| 44 | 17520177 | NA | Mthfs | 5, 10-methenyltetrahydrofolate synthetase | 107885 | ENSMUSG00000066442 |
| 45 | 17480102 | NA | Sytl2 | synaptotagmin-like 2 | 83671 | ENSMUSG00000030616 |
| 46 | 17521143 | NA | Wdr82 | WD repeat domain containing 82 | 77305 | ENSMUSG00000020257 |
| 47 | 17528778 | NA | Ccpg1 | cell cycle progression 1 | 72278 | ENSMUSG00000034563 |
| 48 | 17527261 | NA | Cib2 | calcium and integrin binding family member 2 | 56506 | ENSMUSG00000037493 |
| 49 | 17225499 | NA | Hes6 | hairy and enhancer of split 6 (Drosophila) | 55927 | ENSMUSG00000067071 |
| 50 | 17512434 | NA | Tppp3 | tubulin polymerization-promoting protein family member 3 | 67971 | ENSMUSG00000014846 |
| 51 | 17377583 | NA | Nsfl1c | NSFL1 (p97) cofactor (p47) | 386649 | ENSMUSG00000027455 |
| 52 | 17265175 | NA | 0610010K14Rik | RIKEN cDNA 0610010K14 gene | 104457 | ENSMUSG00000020831 |
| 53 | 17404628 | NA | Pex5l | peroxisomal biogenesis factor 5-like | 58869 | ENSMUSG00000027674 |
| 54 | 17312944 | NA | Polr2f | polymerase (RNA) II (DNA directed) polypeptide F | 69833 | ENSMUSG00000033020 |
| 55 | 17312939 | NA | Polr2f | polymerase (RNA) II (DNA directed) polypeptide F | 69833 | ENSMUSG00000033020 |
| 56 | 17312941 | NA | Polr2f | polymerase (RNA) II (DNA directed) polypeptide F | 69833 | ENSMUSG00000033020 |
| 57 | 17334722 | NA | Rpusd1 | RNA pseudouridylate synthase domain containing 1 | 106707 | ENSMUSG00000041199 |
| 58 | 17517576 | NA | Hmg20a | high mobility group 20A | 66867 | ENSMUSG00000032329 |
| 59 | 17500391 | NA | Rnf122 | ring finger protein 122 | 68867 | ENSMUSG00000039328 |
| 60 | 17404011 | NA | Hey1 | hairy/enhancer-of-split related with YRPW motif 1 | 15213 | ENSMUSG00000040289 |
| 61 | 17400222 | NA | Vps72 | vacuolar protein sorting 72 (yeast) | 21427 | ENSMUSG00000008958 |
| 62 | 17499396 | NA | Fbxo25 | F-box protein 25 | 66822 | ENSMUSG00000038365 |
| 63 | 17246850 | NA | Zmat5 | zinc finger, matrin type 5 | 67178 | ENSMUSG00000009076 |
| 64 | 17324664 | NA | Dlg1 | discs, large homolog 1 (Drosophila) | 13383 | ENSMUSG00000022770 |
| 65 | 17396369 | NA | Nceh1 | arylacetamide deacetylase-like 1 | 320024 | ENSMUSG00000027698 |
| 66 | 17238846 | NA | Syne1 | synaptic nuclear envelope 1 | 64009 | ENSMUSG00000019769 ENSMUSG00000096054 |
| 67 | 17238906 | NA | Syne1 | synaptic nuclear envelope 1 | 64009 | ENSMUSG00000019769 ENSMUSG00000096054 |
| 68 | 17238890 | NA | Syne1 | synaptic nuclear envelope 1 | 64009 | ENSMUSG00000019769 ENSMUSG00000096054 |
| 69 | 17529231 | NA | Phip | pleckstrin homology domain interacting protein | 83946 | ENSMUSG00000032253 |
| 70 | 17385073 | NA | Rbm43 | RNA binding motif protein 43 | 71684 | ENSMUSG00000036249 |
| 71 | 17316625 | NA | Ubr5 | ubiquitin protein ligase E3 component n-recognin 5 | 70790 | ENSMUSG00000037487 |
| 72 | 17277370 | NA | Eif2b2 | eukaryotic translation initiation factor 2B, subunit 2 beta | 217715 | ENSMUSG00000004788 |
| 73 | 17483220 | NA | Cdipt | CDP-diacylglycerol--inositol 3-phosphatidyltransferase (phosphatidylinositol synthase) | 52858 | ENSMUSG00000030682 |
| 74 | 17361975 | NA | Snx15 | sorting nexin 15 | 69024 | ENSMUSG00000024787 |
| 75 | 17245709 | NA | Os9 | amplified in osteosarcoma | 216440 | ENSMUSG00000040462 |
| 76 | 17512009 | NA | Csnk2a2 | casein kinase 2, alpha prime polypeptide | 13000 | ENSMUSG00000046707 |
| 77 | 17396078 | NA | Ralyl | RALY RNA binding protein-like | 76897 | ENSMUSG00000039717 |
| 78 | 17424319 | NA | Sigmar1 | sigma non-opioid intracellular receptor 1 | 18391 | ENSMUSG00000036078 |
| 79 | 17503816 | NA | Irx6 | Iroquois related homeobox 6 (Drosophila) | 64379 | ENSMUSG00000031738 |
| 80 | 17282649 | NA | Rps6kl1 | ribosomal protein S6 kinase-like 1 | 238323 | ENSMUSG00000019235 |
| 81 | 17534615 | NA | Rab33a | RAB33A, member of RAS oncogene family | 19337 | ENSMUSG00000031104 |
| 82 | 17497036 | NA | 2310057M21Rik | RIKEN cDNA 2310057M21 gene | 68277 | ENSMUSG00000040177 |
| 83 | 17260221 | NA | Pold2 | polymerase (DNA directed), delta 2, regulatory subunit | 18972 | ENSMUSG00000020471 |
| 84 | 17365493 | NA | Obfc1 | oligonucleotide/oligosaccharide-binding fold containing 1 | 108689 | ENSMUSG00000042694 |
| 85 | 17538790 | NA | Huwe1 | HECT, UBA and WWE domain containing 1 | 59026 | ENSMUSG00000025261 |
| 86 | 17446580 | NA | Shh | sonic hedgehog | 20423 | ENSMUSG00000002633 |
| 87 | 17522887 | NA | Golga4 | golgi autoantigen, golgin subfamily a, 4 | 54214 | ENSMUSG00000038708 |
| 88 | 17230408 | NA | Adck3 | aarF domain containing kinase 3 | 67426 | ENSMUSG00000026489 |
| 89 | 17420582 | NA | Capzb | capping protein (actin filament) muscle Z-line, beta | 12345 | ENSMUSG00000028745 |
| 90 | 17273086 | NA | Nploc4 | nuclear protein localization 4 homolog (S. cerevisiae) | 217365 | ENSMUSG00000039703 |
| 91 | 17238549 | NA | Wibg | within bgcn homolog (Drosophila) | 78428 | ENSMUSG00000064030 |
| 92 | 17275955 | NA | Atl1 | atlastin GTPase 1 | 73991 | ENSMUSG00000021066 |
| 93 | 17490785 | NA | Lin7b | lin-7 homolog B (C. elegans) | 22342 | ENSMUSG00000003872 |
| 94 | 17303496 | NA | Fezf2 | Fez family zinc finger 2 | 54713 | ENSMUSG00000021743 |
| 95 | 17256565 | NA | Tubg2 | tubulin, gamma 2 | 103768 | ENSMUSG00000045007 |
| 96 | 17463422 | NA | Nrip2 | nuclear receptor interacting protein 2 | 60345 | ENSMUSG00000001520 |
| 97 | 17211335 | NA | Tfap2d | transcription factor AP-2, delta | 226896 | ENSMUSG00000042596 |
| 98 | 17282074 | NA | Rab15 | RAB15, member RAS oncogene family | 104886 | ENSMUSG00000021062 |
| 99 | 17506631 | NA | Tubb3 | tubulin, beta 3 class III | 22152 | ENSMUSG00000062380 |
| 100 | 17253674 | NA | Poldip2 | polymerase (DNA-directed), delta interacting protein 2 | 67811 | ENSMUSG00000001100 |
| 101 | 17321722 | NA | Tfcp2 | transcription factor CP2 | 21422 | ENSMUSG00000009733 |
| 102 | 17213153 | NA | Nif3l1 | Ngg1 interacting factor 3-like 1 (S. pombe) | 65102 | ENSMUSG00000026036 |
| 103 | 17235584 | NA | Dapk3 | death-associated protein kinase 3 | 13144 | ENSMUSG00000034974 |
| 104 | 17477670 | NA | Rras | Harvey rat sarcoma oncogene, subgroup R | 20130 | ENSMUSG00000038387 |
| 105 | 17300411 | NA | Thtpa | thiamine triphosphatase | 105663 | ENSMUSG00000045691 |
| 106 | 17460879 | NA | Hdac11 | histone deacetylase 11 | 232232 | ENSMUSG00000034245 |
| 107 | 17517532 | NA | Isl2 | insulin related protein 2 (islet 2) | 104360 | ENSMUSG00000032318 |
| 108 | 17494637 | NA | Mrpl17 | mitochondrial ribosomal protein L17 | 27397 | ENSMUSG00000030879 |
| 109 | 17513871 | NA | Chmp1a | charged multivesicular body protein 1A | 234852 | ENSMUSG00000000743 |
| 110 | 17484068 | NA | Lhpp | phospholysine phosphohistidine inorganic pyrophosphate phosphatase | 76429 | ENSMUSG00000030946 |
| 111 | 17497366 | NA | Ebf3 | early B cell factor 3 | 13593 | ENSMUSG00000010476 |
| 112 | 17288616 | NA | Tppp | tubulin polymerization promoting protein | 72948 | ENSMUSG00000021573 |
| 113 | 17446322 | NA | Prkag2 | protein kinase, AMP-activated, gamma 2 non-catalytic subunit | 108099 | ENSMUSG00000028944 |
| 114 | 17452552 | NA | Rhof | ras homolog gene family, member f | 23912 | ENSMUSG00000029449 |
| 115 | 17407764 | NA | Prune | prune homolog (Drosophila) | 229589 | ENSMUSG00000015711 |
| 116 | 17524523 | NA | Eif3g | eukaryotic translation initiation factor 3, subunit G | 53356 | ENSMUSG00000070319 |
| 117 | 17306835 | NA | Rabggta | Rab geranylgeranyl transferase, a subunit | 56187 | ENSMUSG00000040472 |
| 118 | 17269638 | NA | Rab5c | RAB5C, member RAS oncogene family | 19345 | ENSMUSG00000019173 |
| 119 | 17256549 | NA | Tubg1 | tubulin, gamma 1 | 103733 | ENSMUSG00000035198 |
| 120 | 17498370 | NA | Nadsyn1 | NAD synthetase 1 | 78914 | ENSMUSG00000031090 |
| 121 | 17214293 | NA | Bcs1l | BCS1-like (yeast) | 66821 | ENSMUSG00000026172 |
| 122 | 17494081 | NA | Rhog | ras homolog gene family, member G | 56212 | ENSMUSG00000073982 |
| 123 | 17540982 | NA | Sept6 | septin 6 | 56526 | ENSMUSG00000050379 |
| 124 | 17257937 | NA | Kcnj2 | potassium inwardly-rectifying channel, subfamily J, member 2 | 16518 | ENSMUSG00000041695 |
| 125 | 17497957 | NA | Chid1 | chitinase domain containing 1 | 68038 | ENSMUSG00000025512 |
| 126 | 17235268 | NA | Ndufs7 | NADH dehydrogenase (ubiquinone) Fe-S protein 7 | 75406 | ENSMUSG00000020153 |
| 127 | 17532045 | NA | Plcd1 | phospholipase C, delta 1 | 18799 | ENSMUSG00000010660 |
| 128 | 17274448 | NA | Cpsf3 | cleavage and polyadenylation specificity factor 3 | 54451 | ENSMUSG00000054309 |
| 129 | 17454416 | NA | Zfand2a | zinc finger, AN1-type domain 2A | 100494 | ENSMUSG00000053581 |
| 130 | 17519868 | NA | Htr1b | 5-hydroxytryptamine (serotonin) receptor 1B | 15551 | ENSMUSG00000049511 |
| 131 | 17515170 | NA | Ilf3 | interleukin enhancer binding factor 3 | 16201 | ENSMUSG00000032178 |
| 132 | 17357213 | NA | Zbtb3 | zinc finger and BTB domain containing 3 | 75291 | ENSMUSG00000071661 |
| 133 | 17453106 | NA | Zfp11 | zinc finger protein 11 | 22648 | ENSMUSG00000051034 |
| 134 | 17461968 | NA | Mkrn2 | makorin, ring finger protein, 2 | 67027 | ENSMUSG00000000439 |
| 135 | 17225179 | NA | Pde6d | phosphodiesterase 6D, cGMP-specific, rod, delta | 18582 | ENSMUSG00000026239 |
| 136 | 17361771 | NA | Sssca1 | Sjogren's syndrome/scleroderma autoantigen 1 homolog (human) | 56390 | ENSMUSG00000079478 |
| 137 | 17521014 | NA | Acad11 | acyl-Coenzyme A dehydrogenase family, member 11 | 102632 | ENSMUSG00000090150 |
| 138 | 17361988 | NA | Arl2 | ADP-ribosylation factor-like 2 | 56327 | ENSMUSG00000024944 |
| 139 | 17502583 | NA | Mcm5 | minichromosome maintenance deficient 5, cell division cycle 46 (S. cerevisiae) | 17218 | ENSMUSG00000005410 |
| 140 | 17539434 | NA | Ctps2 | cytidine 5'-triphosphate synthase 2 | 55936 | ENSMUSG00000031360 |
| 141 | 17341521 | NA | Thoc6 | THO complex 6 homolog (Drosophila) | 386612 | ENSMUSG00000041319 |
| 142 | 17250141 | NA | Zfp39 | zinc finger protein 39 | 22698 | ENSMUSG00000037001 |
| 143 | 17485815 | NA | Syt5 | synaptotagmin V | 53420 | ENSMUSG00000004961 |
| 144 | 17519718 | NA | Mto1 | mitochondrial translation optimization 1 homolog (S. cerevisiae) | 68291 | ENSMUSG00000032342 |
| 145 | 17266322 | NA | Eral1 | Era (G-protein)-like 1 (E. coli) | 57837 | ENSMUSG00000020832 |
| 146 | 17488134 | NA | Rab4b | RAB4B, member RAS oncogene family | 19342 | ENSMUSG00000053291 |
| 147 | 17521422 | NA | Hyal2 | hyaluronoglucosaminidase 2 | 15587 | ENSMUSG00000010047 |
| 148 | 17344453 | NA | Ppp1r18 | protein phosphatase 1, regulatory subunit 18 | 76448 | ENSMUSG00000034595 |
| 149 | 17317208 | NA | Derl1 | Der1-like domain family, member 1 | 67819 | ENSMUSG00000022365 |
| 150 | 17235714 | NA | Dohh | deoxyhypusine hydroxylase/monooxygenase | 102115 | ENSMUSG00000078440 |
| 151 | 17376272 | NA | Nop56 | NOP56 ribonucleoprotein homolog (yeast) | 67134 | ENSMUSG00000027405 |
| 152 | 17379547 | NA | Zswim3 | zinc finger, SWIM domain containing 3 | 67538 | ENSMUSG00000045822 |
| 153 | 17428858 | NA | Rnf220 | ring finger protein 220 | 66743 | ENSMUSG00000028677 |
| 154 | 17428857 | NA | Rnf220 | ring finger protein 220 | 66743 | ENSMUSG00000028677 |
| 155 | 17468195 | NA | Stambp | STAM binding protein | 70527 | ENSMUSG00000006906 |
| 156 | 17408684 | NA | Dclre1b | DNA cross-link repair 1B, PSO2 homolog (S. cerevisiae) | 140917 | ENSMUSG00000027845 |
| 157 | 17443047 | NA | Caln1 | calneuron 1 | 140904 | ENSMUSG00000060371 |
| 158 | 17436077 | NA | Nrbp1 | nuclear receptor binding protein 1 | 192292 | ENSMUSG00000029148 |
| 159 | 17252170 | NA | Rnf167 | ring finger protein 167 | 70510 | ENSMUSG00000040746 |
| 160 | 17543988 | NA | Taf9b | TAF9B RNA polymerase II, TATA box binding protein (TBP)-associated factor | 407786 | ENSMUSG00000047242 |
| 161 | 17342617 | NA | Arhgdig | Rho GDP dissociation inhibitor (GDI) gamma | 14570 | ENSMUSG00000073433 |
| 162 | 17509721 | NA | Tufm | Tu translation elongation factor, mitochondrial | 233870 | ENSMUSG00000073838 |
| 163 | 17361463 | NA | Rab1b | RAB1B, member RAS oncogene family | 76308 | ENSMUSG00000024870 |
| 164 | 17279858 | NA | Fkbp1b | FK506 binding protein 1b | 14226 | ENSMUSG00000020635 |
| 165 | 17391270 | NA | Kcnip3 | Kv channel interacting protein 3, calsenilin | 56461 | ENSMUSG00000079056 |
| 166 | 17217580 | NA | Arl8a | ADP-ribosylation factor-like 8A | 68724 | ENSMUSG00000026426 |
| 167 | 17412579 | NA | Gabrr1 | gamma-aminobutyric acid (GABA) C receptor, subunit rho 1 | 14408 | ENSMUSG00000028280 |
| 168 | 17531834 | NA | Fbxl2 | F-box and leucine-rich repeat protein 2 | 72179 | ENSMUSG00000032507 |
| 169 | 17460099 | NA | Vax2 | ventral anterior homeobox containing gene 2 | 24113 | ENSMUSG00000034777 |
| 170 | 17358777 | NA | Stambpl1 | STAM binding protein like 1 | 76630 | ENSMUSG00000024776 |
| 171 | 17348933 | NA | Mapre2 | microtubule-associated protein, RP/EB family, member 2 | 212307 | ENSMUSG00000024277 |
| 172 | 17299353 | NA | Ktn1 | kinectin 1 | 16709 | ENSMUSG00000021843 |
| 173 | 17506854 | NA | Tsnax | translin-associated factor X | 53424 | ENSMUSG00000056820 |
| 174 | 17498239 | NA | Kcnq1ot1 | KCNQ1 overlapping transcript 1 | 63830 | NULL |
| 175 | 17413573 | NA | Grhpr | glyoxylate reductase/hydroxypyruvate reductase | 76238 | ENSMUSG00000035637 |
| 176 | 17512479 | NA | Acd | adrenocortical dysplasia | 497652 | ENSMUSG00000038000 |
| 177 | 17288716 | NA | Glrx | glutaredoxin | 93692 | ENSMUSG00000021591 |
| 178 | 17429495 | NA | Nfyc | nuclear transcription factor-Y gamma | 18046 | ENSMUSG00000032897 |
| 179 | 17361855 | NA | Pola2 | polymerase (DNA directed), alpha 2 | 18969 | ENSMUSG00000024833 |
| 180 | 17527532 | NA | Mpi | mannose phosphate isomerase | 110119 | ENSMUSG00000032306 |
| 181 | 17353639 | NA | Dnajc18 | DnaJ (Hsp40) homolog, subfamily C, member 18 | 76594 | ENSMUSG00000024350 |
| 182 | 17512463 | NA | Atp6v0d1 | ATPase, H+ transporting, lysosomal V0 subunit D1 | 11972 | ENSMUSG00000013160 |
| 183 | 17512466 | NA | Atp6v0d1 | ATPase, H+ transporting, lysosomal V0 subunit D1 | 11972 | ENSMUSG00000013160 |
| 184 | 17252635 | NA | Shpk | sedoheptulokinase | 74637 | ENSMUSG00000005951 |
| 185 | 17212813 | NA | Mstn | myostatin | 17700 | ENSMUSG00000026100 |
| 186 | 17230945 | NA | Smyd2 | SET and MYND domain containing 2 | 226830 | ENSMUSG00000026603 |
| 187 | 17508554 | NA | Chrna6 | cholinergic receptor, nicotinic, alpha polypeptide 6 | 11440 | ENSMUSG00000031491 |
| 188 | 17313008 | NA | Cby1 | chibby homolog 1 (Drosophila) | 73739 | ENSMUSG00000022428 |
| 189 | 17251607 | NA | Trappc1 | trafficking protein particle complex 1 | 245828 | ENSMUSG00000049299 |
| 190 | 17535434 | NA | Nsdhl | NAD(P) dependent steroid dehydrogenase-like | 18194 | ENSMUSG00000031349 |
| 191 | 17342359 | NA | Haghl | hydroxyacylglutathione hydrolase-like | 68977 | ENSMUSG00000061046 |
| 192 | 17361638 | NA | Snx32 | sorting nexin 32 | 225861 | ENSMUSG00000056185 |
| 193 | 17299750 | NA | Tox4 | TOX high mobility group box family member 4 | 268741 | ENSMUSG00000016831 |
| 194 | 17468113 | NA | Ino80b | INO80 complex subunit B | 70020 | ENSMUSG00000030034 |
| 195 | 17502390 | NA | Rab8a | RAB8A, member RAS oncogene family | 17274 | ENSMUSG00000003037 |
| 196 | 17443461 | NA | Rabl5 | RAB, member of RAS oncogene family-like 5 | 67286 | ENSMUSG00000007987 |
| 197 | 17347558 | NA | Cdkl4 | cyclin-dependent kinase-like 4 | 381113 | ENSMUSG00000033966 |
| 198 | 17503023 | NA | Asf1b | ASF1 anti-silencing function 1 homolog B (S. cerevisiae) | 66929 | ENSMUSG00000005470 |
| 199 | 17283939 | NA | Wars | tryptophanyl-tRNA synthetase | 22375 | ENSMUSG00000021266 |
| 200 | 17283941 | NA | Wars | tryptophanyl-tRNA synthetase | 22375 | ENSMUSG00000021266 |
| 201 | 17283930 | NA | Wars | tryptophanyl-tRNA synthetase | 22375 | ENSMUSG00000021266 |
| 202 | 17283938 | NA | Wars | tryptophanyl-tRNA synthetase | 22375 | ENSMUSG00000021266 |
| 203 | 17454256 | NA | Taf6 | TAF6 RNA polymerase II, TATA box binding protein (TBP)-associated factor | 21343 | ENSMUSG00000036980 |
| 204 | 17512872 | NA | Calb2 | calbindin 2 | 12308 | ENSMUSG00000003657 |
| 205 | 17510365 | NA | Pgls | 6-phosphogluconolactonase | 66171 | ENSMUSG00000031807 |
| 206 | 17527666 | NA | Islr2 | immunoglobulin superfamily containing leucine-rich repeat 2 | 320563 | ENSMUSG00000051243 |
| 207 | 17526273 | NA | Trappc4 | trafficking protein particle complex 4 | 60409 | ENSMUSG00000032112 |
| 208 | 17526271 | NA | Trappc4 | trafficking protein particle complex 4 | 60409 | ENSMUSG00000032112 |
| 209 | 17526272 | NA | Trappc4 | trafficking protein particle complex 4 | 60409 | ENSMUSG00000032112 |
| 210 | 17279499 | NA | Crip2 | cysteine rich protein 2 | 68337 | ENSMUSG00000006356 |
| 211 | 17231118 | NA | Rcor3 | REST corepressor 3 | 214742 | ENSMUSG00000037395 |
| 212 | 17379554 | NA | Zswim1 | zinc finger, SWIM domain containing 1 | 71971 | ENSMUSG00000017764 |
| 213 | 17298407 | NA | Bap1 | Brca1 associated protein 1 | 104416 | ENSMUSG00000021901 |
| 214 | 17318877 | NA | Txn2 | thioredoxin 2 | 56551 | ENSMUSG00000005354 |
| 215 | 17359143 | NA | Lgi1 | leucine-rich repeat LGI family, member 1 | 56839 | ENSMUSG00000067242 |
| 216 | 17453809 | NA | Ap1s1 | adaptor protein complex AP-1, sigma 1 | 11769 | ENSMUSG00000004849 |
| 217 | 17543321 | NA | Zc4h2 | zinc finger, C4H2 domain containing | 245522 | ENSMUSG00000035062 |
| 218 | 17329163 | NA | Camk2n2 | calcium/calmodulin-dependent protein kinase II inhibitor 2 | 73047 | ENSMUSG00000051146 |
| 219 | 17382914 | NA | Dnlz | DNL-type zinc finger | 52838 | ENSMUSG00000075467 |
| 220 | 17451443 | NA | Coro1c | coronin, actin binding protein 1C | 23790 | ENSMUSG00000004530 |
| 221 | 17221014 | NA | Cd34 | CD34 antigen | 12490 | ENSMUSG00000016494 |
| 222 | 17273280 | NA | Stra13 | stimulated by retinoic acid 13 | 20892 | ENSMUSG00000025144 |
| 223 | 17241409 | NA | Srgn | serglycin | 19073 | ENSMUSG00000020077 |
| 224 | 17408805 | NA | Wnt2b | wingless related MMTV integration site 2b | 22414 | ENSMUSG00000027840 |
| 225 | 17518616 | NA | Rbpms2 | RNA binding protein with multiple splicing 2 | 71973 | ENSMUSG00000032387 |
| 226 | 17393357 | NA | Eif6 | eukaryotic translation initiation factor 6 | 16418 | ENSMUSG00000027613 |
| 227 | 17451816 | NA | Hspb8 | heat shock protein 8 | 80888 | ENSMUSG00000041548 |
| 228 | 17213990 | NA | Atic | 5-aminoimidazole-4-carboxamide ribonucleotide formyltransferase/IMP cyclohydrolase | 108147 | ENSMUSG00000026192 |
| 229 | 17504160 | NA | Polr2c | polymerase (RNA) II (DNA directed) polypeptide C | 20021 | ENSMUSG00000031783 |
| 230 | 17455093 | NA | Zkscan14 | zinc finger with KRAB and SCAN domains 14 | 67235 | ENSMUSG00000029627 |
| 231 | 17488544 | NA | Nfkbib | nuclear factor of kappa light polypeptide gene enhancer in B cells inhibitor, beta | 18036 | ENSMUSG00000030595 |
| 232 | 17508691 | NA | Rbpms | RNA binding protein gene with multiple splicing | 19663 | ENSMUSG00000031586 |
| 233 | 17222001 | NA | Prim2 | DNA primase, p58 subunit | 19076 | ENSMUSG00000026134 |
| 234 | 17232649 | NA | Fyn | Fyn proto-oncogene | 14360 | ENSMUSG00000019843 |
| 235 | 17395844 | NA | Stmn3 | stathmin-like 3 | 20262 | ENSMUSG00000027581 |
| 236 | 17229466 | NA | Hsd17b7 | hydroxysteroid (17-beta) dehydrogenase 7 | 15490 | ENSMUSG00000026675 |
| 237 | 17219286 | NA | Dedd | death effector domain-containing | 21945 | ENSMUSG00000013973 |
| 238 | 17306861 | NA | Dhrs1 | dehydrogenase/reductase (SDR family) member 1 | 52585 | ENSMUSG00000002332 |
| 239 | 17306864 | NA | Dhrs1 | dehydrogenase/reductase (SDR family) member 1 | 52585 | ENSMUSG00000002332 |
| 240 | 17306860 | NA | Dhrs1 | dehydrogenase/reductase (SDR family) member 1 | 52585 | ENSMUSG00000002332 |
| 241 | 17306856 | NA | Dhrs1 | dehydrogenase/reductase (SDR family) member 1 | 52585 | ENSMUSG00000002332 |
| 242 | 17306865 | NA | Dhrs1 | dehydrogenase/reductase (SDR family) member 1 | 52585 | ENSMUSG00000002332 |
| 243 | 17305182 | NA | Nrg3 | neuregulin 3 | 18183 | ENSMUSG00000041014 |
| 244 | 17318942 | NA | Pvalb | parvalbumin | 19293 | ENSMUSG00000005716 |
| 245 | 17418571 | NA | Trappc3 | trafficking protein particle complex 3 | 27096 | ENSMUSG00000028847 |
| 246 | 17241780 | NA | Ube2d1 | ubiquitin-conjugating enzyme E2D 1 | 216080 | ENSMUSG00000019927 |
| 247 | 17359945 | NA | Gbf1 | golgi-specific brefeldin A-resistance factor 1 | 107338 | ENSMUSG00000025224 |
| 248 | 17419206 | NA | Pef1 | penta-EF hand domain containing 1 | 67898 | ENSMUSG00000028779 |
| 249 | 17334495 | NA | Nme3 | NME/NM23 nucleoside diphosphate kinase 3 | 79059 | ENSMUSG00000073435 |
| 250 | 17396024 | NA | Stmn2 | stathmin-like 2 | 20257 | ENSMUSG00000027500 |
| 251 | 17535752 | NA | Emd | emerin | 13726 | ENSMUSG00000001964 |
| 252 | 17383588 | NA | Ccbl1 | cysteine conjugate-beta lyase 1 | 70266 | ENSMUSG00000039648 |
| 253 | 17232215 | NA | Moxd1 | monooxygenase, DBH-like 1 | 59012 | ENSMUSG00000020000 |
| 254 | 17400638 | NA | Pex11b | peroxisomal biogenesis factor 11 beta | 18632 | ENSMUSG00000028102 |
| 255 | 17240123 | NA | Clvs2 | clavesin 2 | 215890 | ENSMUSG00000019785 |
| 256 | 17369952 | NA | Sh2d3c | SH2 domain containing 3C | 27387 | ENSMUSG00000059013 |
| 257 | 17421972 | NA | Errfi1 | ERBB receptor feedback inhibitor 1 | 74155 | ENSMUSG00000028967 |
| 258 | 17330373 | NA | Adprh | ADP-ribosylarginine hydrolase | 11544 | ENSMUSG00000002844 |

  
  

| **Database:molecular function      &nbspName:hyaluronoglucuronidase activity      &nbspID:GO:0033906** | | | | | | |
| --- | --- | --- | --- | --- | --- | --- |
| C=2; O=2; E=0.04; R=51.19; rawP=0.0004; adjP=0.0137 | | | | | | |
| Index | UserID | Value | Gene Symbol | Gene Name | EntrezGene | Ensembl |
| 1 | 17521448 | NA | Hyal3 | hyaluronoglucosaminidase 3 | 109685 | ENSMUSG00000036091 |
| 2 | 17521422 | NA | Hyal2 | hyaluronoglucosaminidase 2 | 15587 | ENSMUSG00000010047 |

  
  

| **Database:molecular function      &nbspName:oxidoreductase activity, acting on a sulfur group of donors, disulfide as acceptor      &nbspID:GO:0016671** | | | | | | |
| --- | --- | --- | --- | --- | --- | --- |
| C=9; O=3; E=0.18; R=17.06; rawP=0.0006; adjP=0.0164 | | | | | | |
| Index | UserID | Value | Gene Symbol | Gene Name | EntrezGene | Ensembl |
| 1 | 17307695 | NA | Msra | methionine sulfoxide reductase A | 110265 | ENSMUSG00000054733 |
| 2 | 17288716 | NA | Glrx | glutaredoxin | 93692 | ENSMUSG00000021591 |
| 3 | 17318877 | NA | Txn2 | thioredoxin 2 | 56551 | ENSMUSG00000005354 |

  
  

| **Database:molecular function      &nbspName:catalytic activity      &nbspID:GO:0003824** | | | | | | |
| --- | --- | --- | --- | --- | --- | --- |
| C=4976; O=126; E=97.20; R=1.30; rawP=0.0006; adjP=0.0164 | | | | | | |
| Index | UserID | Value | Gene Symbol | Gene Name | EntrezGene | Ensembl |
| 1 | 17235268 | NA | Ndufs7 | NADH dehydrogenase (ubiquinone) Fe-S protein 7 | 75406 | ENSMUSG00000020153 |
| 2 | 17532045 | NA | Plcd1 | phospholipase C, delta 1 | 18799 | ENSMUSG00000010660 |
| 3 | 17447726 | NA | Hs3st1 | heparan sulfate (glucosamine) 3-O-sulfotransferase 1 | 15476 | ENSMUSG00000051022 |
| 4 | 17274448 | NA | Cpsf3 | cleavage and polyadenylation specificity factor 3 | 54451 | ENSMUSG00000054309 |
| 5 | 17422117 | NA | Acot7 | acyl-CoA thioesterase 7 | 70025 | ENSMUSG00000028937 |
| 6 | 17344794 | NA | Znrd1 | zinc ribbon domain containing, 1 | 66136 | ENSMUSG00000036315 |
| 7 | 17536264 | NA | Pcyt1b | phosphate cytidylyltransferase 1, choline, beta isoform | 236899 | ENSMUSG00000035246 |
| 8 | 17461968 | NA | Mkrn2 | makorin, ring finger protein, 2 | 67027 | ENSMUSG00000000439 |
| 9 | 17402099 | NA | 4833424O15Rik | RIKEN cDNA 4833424O15 gene | 75769 | ENSMUSG00000033342 |
| 10 | 17414045 | NA | E130309F12Rik | RIKEN cDNA E130309F12 gene | 272031 | ENSMUSG00000063446 |
| 11 | 17224540 | NA | Tuba4a | tubulin, alpha 4A | 22145 | ENSMUSG00000026202 |
| 12 | 17503910 | NA | Ogfod1 | 2-oxoglutarate and iron-dependent oxygenase domain containing 1 | 270086 | ENSMUSG00000033009 |
| 13 | 17225179 | NA | Pde6d | phosphodiesterase 6D, cGMP-specific, rod, delta | 18582 | ENSMUSG00000026239 |
| 14 | 17517723 | NA | Rpp25 | ribonuclease P 25 subunit (human) | 102614 | ENSMUSG00000062309 |
| 15 | 17307134 | NA | Cryl1 | crystallin, lambda 1 | 68631 | ENSMUSG00000021947 |
| 16 | 17322735 | NA | Mettl22 | methyltransferase like 22 | 239706 | ENSMUSG00000039345 |
| 17 | 17319324 | NA | Dnalc4 | dynein, axonemal, light chain 4 | 54152 | ENSMUSG00000022420 |
| 18 | 17521014 | NA | Acad11 | acyl-Coenzyme A dehydrogenase family, member 11 | 102632 | ENSMUSG00000090150 |
| 19 | 17512103 | NA | Got2 | glutamate oxaloacetate transaminase 2, mitochondrial | 14719 | ENSMUSG00000031672 |
| 20 | 17369862 | NA | Dpm2 | dolichol-phosphate (beta-D) mannosyltransferase 2 | 13481 | ENSMUSG00000026810 |
| 21 | 17361988 | NA | Arl2 | ADP-ribosylation factor-like 2 | 56327 | ENSMUSG00000024944 |
| 22 | 17229948 | NA | Dusp23 | dual specificity phosphatase 23 | 68440 | ENSMUSG00000026544 |
| 23 | 17502583 | NA | Mcm5 | minichromosome maintenance deficient 5, cell division cycle 46 (S. cerevisiae) | 17218 | ENSMUSG00000005410 |
| 24 | 17322559 | NA | Hmox2 | heme oxygenase (decycling) 2 | 15369 | ENSMUSG00000004070 |
| 25 | 17539434 | NA | Ctps2 | cytidine 5'-triphosphate synthase 2 | 55936 | ENSMUSG00000031360 |
| 26 | 17328225 | NA | Cpped1 | calcineurin-like phosphoesterase domain containing 1 | 223978 | ENSMUSG00000065979 |
| 27 | 17521422 | NA | Hyal2 | hyaluronoglucosaminidase 2 | 15587 | ENSMUSG00000010047 |
| 28 | 17404329 | NA | Gyg | glycogenin | 27357 | ENSMUSG00000019528 |
| 29 | 17235714 | NA | Dohh | deoxyhypusine hydroxylase/monooxygenase | 102115 | ENSMUSG00000078440 |
| 30 | 17321467 | NA | Tuba1b | tubulin, alpha 1B | 22143 | ENSMUSG00000023004 |
| 31 | 17428858 | NA | Rnf220 | ring finger protein 220 | 66743 | ENSMUSG00000028677 |
| 32 | 17428857 | NA | Rnf220 | ring finger protein 220 | 66743 | ENSMUSG00000028677 |
| 33 | 17468195 | NA | Stambp | STAM binding protein | 70527 | ENSMUSG00000006906 |
| 34 | 17288160 | NA | Cdk20 | cyclin-dependent kinase 20 | 105278 | ENSMUSG00000021483 |
| 35 | 17538096 | NA | Rnf128 | ring finger protein 128 | 66889 | ENSMUSG00000031438 |
| 36 | 17408684 | NA | Dclre1b | DNA cross-link repair 1B, PSO2 homolog (S. cerevisiae) | 140917 | ENSMUSG00000027845 |
| 37 | 17436077 | NA | Nrbp1 | nuclear receptor binding protein 1 | 192292 | ENSMUSG00000029148 |
| 38 | 17447099 | NA | Ctbp1 | C-terminal binding protein 1 | 13016 | ENSMUSG00000037373 |
| 39 | 17447089 | NA | Ctbp1 | C-terminal binding protein 1 | 13016 | ENSMUSG00000037373 |
| 40 | 17447100 | NA | Ctbp1 | C-terminal binding protein 1 | 13016 | ENSMUSG00000037373 |
| 41 | 17447098 | NA | Ctbp1 | C-terminal binding protein 1 | 13016 | ENSMUSG00000037373 |
| 42 | 17343617 | NA | Rab11b | RAB11B, member RAS oncogene family | 19326 | ENSMUSG00000077450 |
| 43 | 17440361 | NA | Plcxd1 | phosphatidylinositol-specific phospholipase C, X domain containing 1 | 403178 | ENSMUSG00000064247 |
| 44 | 17252170 | NA | Rnf167 | ring finger protein 167 | 70510 | ENSMUSG00000040746 |
| 45 | 17246284 | NA | Suox | sulfite oxidase | 211389 | ENSMUSG00000049858 |
| 46 | 17437611 | NA | Pgm1 | phosphoglucomutase 1 | 66681 | ENSMUSG00000029171 |
| 47 | 17509721 | NA | Tufm | Tu translation elongation factor, mitochondrial | 233870 | ENSMUSG00000073838 |
| 48 | 17459676 | NA | Retsat | retinol saturase (all trans retinol 13,14 reductase) | 67442 | ENSMUSG00000056666 |
| 49 | 17297750 | NA | Ppif | peptidylprolyl isomerase F (cyclophilin F) | 105675 | ENSMUSG00000021868 |
| 50 | 17279858 | NA | Fkbp1b | FK506 binding protein 1b | 14226 | ENSMUSG00000020635 |
| 51 | 17324576 | NA | Hrasls | HRAS-like suppressor | 27281 | ENSMUSG00000022525 |
| 52 | 17520177 | NA | Mthfs | 5, 10-methenyltetrahydrofolate synthetase | 107885 | ENSMUSG00000066442 |
| 53 | 17531834 | NA | Fbxl2 | F-box and leucine-rich repeat protein 2 | 72179 | ENSMUSG00000032507 |
| 54 | 17369672 | NA | Ppapdc3 | phosphatidic acid phosphatase type 2 domain containing 3 | 227721 | ENSMUSG00000051373 |
| 55 | 17358777 | NA | Stambpl1 | STAM binding protein like 1 | 76630 | ENSMUSG00000024776 |
| 56 | 17257060 | NA | Nmt1 | N-myristoyltransferase 1 | 18107 | ENSMUSG00000020936 |
| 57 | 17278188 | NA | Otub2 | OTU domain, ubiquitin aldehyde binding 2 | 68149 | ENSMUSG00000021203 |
| 58 | 17413573 | NA | Grhpr | glyoxylate reductase/hydroxypyruvate reductase | 76238 | ENSMUSG00000035637 |
| 59 | 17312944 | NA | Polr2f | polymerase (RNA) II (DNA directed) polypeptide F | 69833 | ENSMUSG00000033020 |
| 60 | 17312939 | NA | Polr2f | polymerase (RNA) II (DNA directed) polypeptide F | 69833 | ENSMUSG00000033020 |
| 61 | 17312941 | NA | Polr2f | polymerase (RNA) II (DNA directed) polypeptide F | 69833 | ENSMUSG00000033020 |
| 62 | 17329151 | NA | Alg3 | asparagine-linked glycosylation 3 (alpha-1,3-mannosyltransferase) | 208624 | ENSMUSG00000033809 |
| 63 | 17334722 | NA | Rpusd1 | RNA pseudouridylate synthase domain containing 1 | 106707 | ENSMUSG00000041199 |
| 64 | 17288716 | NA | Glrx | glutaredoxin | 93692 | ENSMUSG00000021591 |
| 65 | 17540050 | NA | Ebp | phenylalkylamine Ca2+ antagonist (emopamil) binding protein | 13595 | ENSMUSG00000031168 |
| 66 | 17361855 | NA | Pola2 | polymerase (DNA directed), alpha 2 | 18969 | ENSMUSG00000024833 |
| 67 | 17527532 | NA | Mpi | mannose phosphate isomerase | 110119 | ENSMUSG00000032306 |
| 68 | 17232426 | NA | Echdc1 | enoyl Coenzyme A hydratase domain containing 1 | 52665 | ENSMUSG00000019883 |
| 69 | 17252635 | NA | Shpk | sedoheptulokinase | 74637 | ENSMUSG00000005951 |
| 70 | 17512463 | NA | Atp6v0d1 | ATPase, H+ transporting, lysosomal V0 subunit D1 | 11972 | ENSMUSG00000013160 |
| 71 | 17512466 | NA | Atp6v0d1 | ATPase, H+ transporting, lysosomal V0 subunit D1 | 11972 | ENSMUSG00000013160 |
| 72 | 17230945 | NA | Smyd2 | SET and MYND domain containing 2 | 226830 | ENSMUSG00000026603 |
| 73 | 17521448 | NA | Hyal3 | hyaluronoglucosaminidase 3 | 109685 | ENSMUSG00000036091 |
| 74 | 17535434 | NA | Nsdhl | NAD(P) dependent steroid dehydrogenase-like | 18194 | ENSMUSG00000031349 |
| 75 | 17342359 | NA | Haghl | hydroxyacylglutathione hydrolase-like | 68977 | ENSMUSG00000061046 |
| 76 | 17396369 | NA | Nceh1 | arylacetamide deacetylase-like 1 | 320024 | ENSMUSG00000027698 |
| 77 | 17347558 | NA | Cdkl4 | cyclin-dependent kinase-like 4 | 381113 | ENSMUSG00000033966 |
| 78 | 17283939 | NA | Wars | tryptophanyl-tRNA synthetase | 22375 | ENSMUSG00000021266 |
| 79 | 17283941 | NA | Wars | tryptophanyl-tRNA synthetase | 22375 | ENSMUSG00000021266 |
| 80 | 17283930 | NA | Wars | tryptophanyl-tRNA synthetase | 22375 | ENSMUSG00000021266 |
| 81 | 17283938 | NA | Wars | tryptophanyl-tRNA synthetase | 22375 | ENSMUSG00000021266 |
| 82 | 17432967 | NA | Ubiad1 | UbiA prenyltransferase domain containing 1 | 71707 | ENSMUSG00000047719 |
| 83 | 17510365 | NA | Pgls | 6-phosphogluconolactonase | 66171 | ENSMUSG00000031807 |
| 84 | 17316625 | NA | Ubr5 | ubiquitin protein ligase E3 component n-recognin 5 | 70790 | ENSMUSG00000037487 |
| 85 | 17483220 | NA | Cdipt | CDP-diacylglycerol--inositol 3-phosphatidyltransferase (phosphatidylinositol synthase) | 52858 | ENSMUSG00000030682 |
| 86 | 17512009 | NA | Csnk2a2 | casein kinase 2, alpha prime polypeptide | 13000 | ENSMUSG00000046707 |
| 87 | 17424319 | NA | Sigmar1 | sigma non-opioid intracellular receptor 1 | 18391 | ENSMUSG00000036078 |
| 88 | 17321578 | NA | Bcdin3d | BCDIN3 domain containing | 75284 | ENSMUSG00000037525 |
| 89 | 17282649 | NA | Rps6kl1 | ribosomal protein S6 kinase-like 1 | 238323 | ENSMUSG00000019235 |
| 90 | 17260221 | NA | Pold2 | polymerase (DNA directed), delta 2, regulatory subunit | 18972 | ENSMUSG00000020471 |
| 91 | 17362216 | NA | Nudt22 | nudix (nucleoside diphosphate linked moiety X)-type motif 22 | 68323 | ENSMUSG00000037349 |
| 92 | 17298407 | NA | Bap1 | Brca1 associated protein 1 | 104416 | ENSMUSG00000021901 |
| 93 | 17538790 | NA | Huwe1 | HECT, UBA and WWE domain containing 1 | 59026 | ENSMUSG00000025261 |
| 94 | 17318877 | NA | Txn2 | thioredoxin 2 | 56551 | ENSMUSG00000005354 |
| 95 | 17446580 | NA | Shh | sonic hedgehog | 20423 | ENSMUSG00000002633 |
| 96 | 17329163 | NA | Camk2n2 | calcium/calmodulin-dependent protein kinase II inhibitor 2 | 73047 | ENSMUSG00000051146 |
| 97 | 17265186 | NA | Rnasek | ribonuclease, RNase K | 52898 | ENSMUSG00000093989 |
| 98 | 17470879 | NA | Tpi1 | triosephosphate isomerase 1 | 21991 | ENSMUSG00000023456 |
| 99 | 17281971 | NA | Sgpp1 | sphingosine-1-phosphate phosphatase 1 | 81535 | ENSMUSG00000021054 |
| 100 | 17230408 | NA | Adck3 | aarF domain containing kinase 3 | 67426 | ENSMUSG00000026489 |
| 101 | 17275955 | NA | Atl1 | atlastin GTPase 1 | 73991 | ENSMUSG00000021066 |
| 102 | 17213990 | NA | Atic | 5-aminoimidazole-4-carboxamide ribonucleotide formyltransferase/IMP cyclohydrolase | 108147 | ENSMUSG00000026192 |
| 103 | 17431502 | NA | Lypla2 | lysophospholipase 2 | 26394 | ENSMUSG00000028670 |
| 104 | 17307695 | NA | Msra | methionine sulfoxide reductase A | 110265 | ENSMUSG00000054733 |
| 105 | 17256565 | NA | Tubg2 | tubulin, gamma 2 | 103768 | ENSMUSG00000045007 |
| 106 | 17463422 | NA | Nrip2 | nuclear receptor interacting protein 2 | 60345 | ENSMUSG00000001520 |
| 107 | 17222001 | NA | Prim2 | DNA primase, p58 subunit | 19076 | ENSMUSG00000026134 |
| 108 | 17299542 | NA | Tmem55b | transmembrane protein 55b | 219024 | ENSMUSG00000035953 |
| 109 | 17506631 | NA | Tubb3 | tubulin, beta 3 class III | 22152 | ENSMUSG00000062380 |
| 110 | 17309825 | NA | Plcxd3 | phosphatidylinositol-specific phospholipase C, X domain containing 3 | 239318 | ENSMUSG00000049148 |
| 111 | 17232649 | NA | Fyn | Fyn proto-oncogene | 14360 | ENSMUSG00000019843 |
| 112 | 17229466 | NA | Hsd17b7 | hydroxysteroid (17-beta) dehydrogenase 7 | 15490 | ENSMUSG00000026675 |
| 113 | 17235584 | NA | Dapk3 | death-associated protein kinase 3 | 13144 | ENSMUSG00000034974 |
| 114 | 17477670 | NA | Rras | Harvey rat sarcoma oncogene, subgroup R | 20130 | ENSMUSG00000038387 |
| 115 | 17405819 | NA | B3galnt1 | UDP-GalNAc:betaGlcNAc beta 1,3-galactosaminyltransferase, polypeptide 1 | 26879 | ENSMUSG00000043300 |
| 116 | 17300411 | NA | Thtpa | thiamine triphosphatase | 105663 | ENSMUSG00000045691 |
| 117 | 17306861 | NA | Dhrs1 | dehydrogenase/reductase (SDR family) member 1 | 52585 | ENSMUSG00000002332 |
| 118 | 17306864 | NA | Dhrs1 | dehydrogenase/reductase (SDR family) member 1 | 52585 | ENSMUSG00000002332 |
| 119 | 17306860 | NA | Dhrs1 | dehydrogenase/reductase (SDR family) member 1 | 52585 | ENSMUSG00000002332 |
| 120 | 17306856 | NA | Dhrs1 | dehydrogenase/reductase (SDR family) member 1 | 52585 | ENSMUSG00000002332 |
| 121 | 17306865 | NA | Dhrs1 | dehydrogenase/reductase (SDR family) member 1 | 52585 | ENSMUSG00000002332 |
| 122 | 17460879 | NA | Hdac11 | histone deacetylase 11 | 232232 | ENSMUSG00000034245 |
| 123 | 17241780 | NA | Ube2d1 | ubiquitin-conjugating enzyme E2D 1 | 216080 | ENSMUSG00000019927 |
| 124 | 17444100 | NA | Chst12 | carbohydrate sulfotransferase 12 | 59031 | ENSMUSG00000036599 |
| 125 | 17334495 | NA | Nme3 | NME/NM23 nucleoside diphosphate kinase 3 | 79059 | ENSMUSG00000073435 |
| 126 | 17484068 | NA | Lhpp | phospholysine phosphohistidine inorganic pyrophosphate phosphatase | 76429 | ENSMUSG00000030946 |
| 127 | 17383588 | NA | Ccbl1 | cysteine conjugate-beta lyase 1 | 70266 | ENSMUSG00000039648 |
| 128 | 17407764 | NA | Prune | prune homolog (Drosophila) | 229589 | ENSMUSG00000015711 |
| 129 | 17446322 | NA | Prkag2 | protein kinase, AMP-activated, gamma 2 non-catalytic subunit | 108099 | ENSMUSG00000028944 |
| 130 | 17313199 | NA | Adsl | adenylosuccinate lyase | 11564 | ENSMUSG00000022407 |
| 131 | 17306835 | NA | Rabggta | Rab geranylgeranyl transferase, a subunit | 56187 | ENSMUSG00000040472 |
| 132 | 17232215 | NA | Moxd1 | monooxygenase, DBH-like 1 | 59012 | ENSMUSG00000020000 |
| 133 | 17269638 | NA | Rab5c | RAB5C, member RAS oncogene family | 19345 | ENSMUSG00000019173 |
| 134 | 17356202 | NA | Pold4 | polymerase (DNA-directed), delta 4 | 69745 | ENSMUSG00000024854 |
| 135 | 17256549 | NA | Tubg1 | tubulin, gamma 1 | 103733 | ENSMUSG00000035198 |
| 136 | 17498370 | NA | Nadsyn1 | NAD synthetase 1 | 78914 | ENSMUSG00000031090 |
| 137 | 17214293 | NA | Bcs1l | BCS1-like (yeast) | 66821 | ENSMUSG00000026172 |
| 138 | 17330373 | NA | Adprh | ADP-ribosylarginine hydrolase | 11544 | ENSMUSG00000002844 |
| 139 | 17494081 | NA | Rhog | ras homolog gene family, member G | 56212 | ENSMUSG00000073982 |
| 140 | 17497957 | NA | Chid1 | chitinase domain containing 1 | 68038 | ENSMUSG00000025512 |

  
  

| **Database:cellular component      &nbspName:intracellular part      &nbspID:GO:0044424** | | | | | | |
| --- | --- | --- | --- | --- | --- | --- |
| C=10721; O=282; E=207.19; R=1.36; rawP=8.40e-15; adjP=1.08e-12 | | | | | | |
| Index | UserID | Value | Gene Symbol | Gene Name | EntrezGene | Ensembl |
| 1 | 17476273 | NA | Zfp382 | zinc finger protein 382 | 233060 | ENSMUSG00000074220 |
| 2 | 17413221 | NA | Unc13b | unc-13 homolog B (C. elegans) | 22249 | ENSMUSG00000028456 |
| 3 | 17474547 | NA | Rtn2 | reticulon 2 (Z-band associated protein) | 20167 | ENSMUSG00000030401 |
| 4 | 17512740 | NA | Nob1 | NIN1/RPN12 binding protein 1 homolog (S. cerevisiae) | 67619 | ENSMUSG00000003848 |
| 5 | 17503910 | NA | Ogfod1 | 2-oxoglutarate and iron-dependent oxygenase domain containing 1 | 270086 | ENSMUSG00000033009 |
| 6 | 17368171 | NA | Bmyc | brain expressed myelocytomatosis oncogene | 107771 | ENSMUSG00000049086 |
| 7 | 17350134 | NA | Pou4f3 | POU domain, class 4, transcription factor 3 | 18998 | ENSMUSG00000024497 |
| 8 | 17443181 | NA | Dnajc30 | DnaJ (Hsp40) homolog, subfamily C, member 30 | 66114 | ENSMUSG00000061118 |
| 9 | 17319324 | NA | Dnalc4 | dynein, axonemal, light chain 4 | 54152 | ENSMUSG00000022420 |
| 10 | 17512103 | NA | Got2 | glutamate oxaloacetate transaminase 2, mitochondrial | 14719 | ENSMUSG00000031672 |
| 11 | 17336829 | NA | Lsm2 | LSM2 homolog, U6 small nuclear RNA associated (S. cerevisiae) | 27756 | ENSMUSG00000007050 |
| 12 | 17288454 | NA | Irx4 | Iroquois related homeobox 4 (Drosophila) | 50916 | ENSMUSG00000021604 |
| 13 | 17467996 | NA | Mrpl19 | mitochondrial ribosomal protein L19 | 56284 | ENSMUSG00000030045 |
| 14 | 17306758 | NA | Tm9sf1 | transmembrane 9 superfamily member 1 | 74140 | ENSMUSG00000002320 |
| 15 | 17504712 | NA | Exoc3l | exocyst complex component 3-like | 277978 | ENSMUSG00000043251 |
| 16 | 17321467 | NA | Tuba1b | tubulin, alpha 1B | 22143 | ENSMUSG00000023004 |
| 17 | 17541597 | NA | Frmd7 | FERM domain containing 7 | 385354 | ENSMUSG00000036131 |
| 18 | 17379187 | NA | Ift52 | intraflagellar transport 52 | 245866 | ENSMUSG00000017858 |
| 19 | 17246284 | NA | Suox | sulfite oxidase | 211389 | ENSMUSG00000049858 |
| 20 | 17437611 | NA | Pgm1 | phosphoglucomutase 1 | 66681 | ENSMUSG00000029171 |
| 21 | 17287579 | NA | Zfp346 | zinc finger protein 346 | 26919 | ENSMUSG00000021481 |
| 22 | 17224577 | NA | Resp18 | regulated endocrine-specific protein 18 | 19711 | ENSMUSG00000033061 |
| 23 | 17297750 | NA | Ppif | peptidylprolyl isomerase F (cyclophilin F) | 105675 | ENSMUSG00000021868 |
| 24 | 17520177 | NA | Mthfs | 5, 10-methenyltetrahydrofolate synthetase | 107885 | ENSMUSG00000066442 |
| 25 | 17480102 | NA | Sytl2 | synaptotagmin-like 2 | 83671 | ENSMUSG00000030616 |
| 26 | 17521143 | NA | Wdr82 | WD repeat domain containing 82 | 77305 | ENSMUSG00000020257 |
| 27 | 17312944 | NA | Polr2f | polymerase (RNA) II (DNA directed) polypeptide F | 69833 | ENSMUSG00000033020 |
| 28 | 17312939 | NA | Polr2f | polymerase (RNA) II (DNA directed) polypeptide F | 69833 | ENSMUSG00000033020 |
| 29 | 17312941 | NA | Polr2f | polymerase (RNA) II (DNA directed) polypeptide F | 69833 | ENSMUSG00000033020 |
| 30 | 17329151 | NA | Alg3 | asparagine-linked glycosylation 3 (alpha-1,3-mannosyltransferase) | 208624 | ENSMUSG00000033809 |
| 31 | 17500391 | NA | Rnf122 | ring finger protein 122 | 68867 | ENSMUSG00000039328 |
| 32 | 17400222 | NA | Vps72 | vacuolar protein sorting 72 (yeast) | 21427 | ENSMUSG00000008958 |
| 33 | 17499396 | NA | Fbxo25 | F-box protein 25 | 66822 | ENSMUSG00000038365 |
| 34 | 17324664 | NA | Dlg1 | discs, large homolog 1 (Drosophila) | 13383 | ENSMUSG00000022770 |
| 35 | 17396369 | NA | Nceh1 | arylacetamide deacetylase-like 1 | 320024 | ENSMUSG00000027698 |
| 36 | 17314556 | NA | Slc48a1 | solute carrier family 48 (heme transporter), member 1 | 67739 | ENSMUSG00000081534 |
| 37 | 17246209 | NA | Rpl41 | ribosomal protein L41 | 67945 | ENSMUSG00000093674 |
| 38 | 17529231 | NA | Phip | pleckstrin homology domain interacting protein | 83946 | ENSMUSG00000032253 |
| 39 | 17483220 | NA | Cdipt | CDP-diacylglycerol--inositol 3-phosphatidyltransferase (phosphatidylinositol synthase) | 52858 | ENSMUSG00000030682 |
| 40 | 17361975 | NA | Snx15 | sorting nexin 15 | 69024 | ENSMUSG00000024787 |
| 41 | 17424319 | NA | Sigmar1 | sigma non-opioid intracellular receptor 1 | 18391 | ENSMUSG00000036078 |
| 42 | 17260221 | NA | Pold2 | polymerase (DNA directed), delta 2, regulatory subunit | 18972 | ENSMUSG00000020471 |
| 43 | 17446580 | NA | Shh | sonic hedgehog | 20423 | ENSMUSG00000002633 |
| 44 | 17281971 | NA | Sgpp1 | sphingosine-1-phosphate phosphatase 1 | 81535 | ENSMUSG00000021054 |
| 45 | 17238549 | NA | Wibg | within bgcn homolog (Drosophila) | 78428 | ENSMUSG00000064030 |
| 46 | 17212355 | NA | Nck2 | non-catalytic region of tyrosine kinase adaptor protein 2 | 17974 | ENSMUSG00000066877 |
| 47 | 17307695 | NA | Msra | methionine sulfoxide reductase A | 110265 | ENSMUSG00000054733 |
| 48 | 17303496 | NA | Fezf2 | Fez family zinc finger 2 | 54713 | ENSMUSG00000021743 |
| 49 | 17345519 | NA | Rrp36 | ribosomal RNA processing 36 homolog (S. cerevisiae) | 224823 | ENSMUSG00000023971 |
| 50 | 17211335 | NA | Tfap2d | transcription factor AP-2, delta | 226896 | ENSMUSG00000042596 |
| 51 | 17506631 | NA | Tubb3 | tubulin, beta 3 class III | 22152 | ENSMUSG00000062380 |
| 52 | 17235584 | NA | Dapk3 | death-associated protein kinase 3 | 13144 | ENSMUSG00000034974 |
| 53 | 17338670 | NA | Fsd1 | fibronectin type 3 and SPRY domain-containing protein | 240121 | ENSMUSG00000011589 |
| 54 | 17300411 | NA | Thtpa | thiamine triphosphatase | 105663 | ENSMUSG00000045691 |
| 55 | 17460879 | NA | Hdac11 | histone deacetylase 11 | 232232 | ENSMUSG00000034245 |
| 56 | 17444100 | NA | Chst12 | carbohydrate sulfotransferase 12 | 59031 | ENSMUSG00000036599 |
| 57 | 17359994 | NA | Fbxl15 | F-box and leucine-rich repeat protein 15 | 68431 | ENSMUSG00000025226 |
| 58 | 17217666 | NA | Tmem9 | transmembrane protein 9 | 66241 | ENSMUSG00000026411 |
| 59 | 17484068 | NA | Lhpp | phospholysine phosphohistidine inorganic pyrophosphate phosphatase | 76429 | ENSMUSG00000030946 |
| 60 | 17497366 | NA | Ebf3 | early B cell factor 3 | 13593 | ENSMUSG00000010476 |
| 61 | 17288616 | NA | Tppp | tubulin polymerization promoting protein | 72948 | ENSMUSG00000021573 |
| 62 | 17446322 | NA | Prkag2 | protein kinase, AMP-activated, gamma 2 non-catalytic subunit | 108099 | ENSMUSG00000028944 |
| 63 | 17452552 | NA | Rhof | ras homolog gene family, member f | 23912 | ENSMUSG00000029449 |
| 64 | 17407764 | NA | Prune | prune homolog (Drosophila) | 229589 | ENSMUSG00000015711 |
| 65 | 17524523 | NA | Eif3g | eukaryotic translation initiation factor 3, subunit G | 53356 | ENSMUSG00000070319 |
| 66 | 17268995 | NA | Krt222 | keratin 222 | 268481 | ENSMUSG00000035849 |
| 67 | 17505367 | NA | Txnl4b | thioredoxin-like 4B | 234723 | ENSMUSG00000031723 |
| 68 | 17540982 | NA | Sept6 | septin 6 | 56526 | ENSMUSG00000050379 |
| 69 | 17235268 | NA | Ndufs7 | NADH dehydrogenase (ubiquinone) Fe-S protein 7 | 75406 | ENSMUSG00000020153 |
| 70 | 17447726 | NA | Hs3st1 | heparan sulfate (glucosamine) 3-O-sulfotransferase 1 | 15476 | ENSMUSG00000051022 |
| 71 | 17454416 | NA | Zfand2a | zinc finger, AN1-type domain 2A | 100494 | ENSMUSG00000053581 |
| 72 | 17422117 | NA | Acot7 | acyl-CoA thioesterase 7 | 70025 | ENSMUSG00000028937 |
| 73 | 17536264 | NA | Pcyt1b | phosphate cytidylyltransferase 1, choline, beta isoform | 236899 | ENSMUSG00000035246 |
| 74 | 17357213 | NA | Zbtb3 | zinc finger and BTB domain containing 3 | 75291 | ENSMUSG00000071661 |
| 75 | 17453106 | NA | Zfp11 | zinc finger protein 11 | 22648 | ENSMUSG00000051034 |
| 76 | 17315558 | NA | Copz1 | coatomer protein complex, subunit zeta 1 | 56447 | ENSMUSG00000060992 |
| 77 | 17361988 | NA | Arl2 | ADP-ribosylation factor-like 2 | 56327 | ENSMUSG00000024944 |
| 78 | 17502583 | NA | Mcm5 | minichromosome maintenance deficient 5, cell division cycle 46 (S. cerevisiae) | 17218 | ENSMUSG00000005410 |
| 79 | 17485815 | NA | Syt5 | synaptotagmin V | 53420 | ENSMUSG00000004961 |
| 80 | 17519718 | NA | Mto1 | mitochondrial translation optimization 1 homolog (S. cerevisiae) | 68291 | ENSMUSG00000032342 |
| 81 | 17488134 | NA | Rab4b | RAB4B, member RAS oncogene family | 19342 | ENSMUSG00000053291 |
| 82 | 17344453 | NA | Ppp1r18 | protein phosphatase 1, regulatory subunit 18 | 76448 | ENSMUSG00000034595 |
| 83 | 17317208 | NA | Derl1 | Der1-like domain family, member 1 | 67819 | ENSMUSG00000022365 |
| 84 | 17501544 | NA | Npy1r | neuropeptide Y receptor Y1 | 18166 | ENSMUSG00000036437 |
| 85 | 17428858 | NA | Rnf220 | ring finger protein 220 | 66743 | ENSMUSG00000028677 |
| 86 | 17428857 | NA | Rnf220 | ring finger protein 220 | 66743 | ENSMUSG00000028677 |
| 87 | 17408684 | NA | Dclre1b | DNA cross-link repair 1B, PSO2 homolog (S. cerevisiae) | 140917 | ENSMUSG00000027845 |
| 88 | 17443047 | NA | Caln1 | calneuron 1 | 140904 | ENSMUSG00000060371 |
| 89 | 17436077 | NA | Nrbp1 | nuclear receptor binding protein 1 | 192292 | ENSMUSG00000029148 |
| 90 | 17252170 | NA | Rnf167 | ring finger protein 167 | 70510 | ENSMUSG00000040746 |
| 91 | 17543988 | NA | Taf9b | TAF9B RNA polymerase II, TATA box binding protein (TBP)-associated factor | 407786 | ENSMUSG00000047242 |
| 92 | 17459676 | NA | Retsat | retinol saturase (all trans retinol 13,14 reductase) | 67442 | ENSMUSG00000056666 |
| 93 | 17337269 | NA | Nrm | nurim (nuclear envelope membrane protein) | 106582 | ENSMUSG00000059791 |
| 94 | 17488463 | NA | Med29 | mediator complex subunit 29 | 67224 | ENSMUSG00000003444 |
| 95 | 17391270 | NA | Kcnip3 | Kv channel interacting protein 3, calsenilin | 56461 | ENSMUSG00000079056 |
| 96 | 17324576 | NA | Hrasls | HRAS-like suppressor | 27281 | ENSMUSG00000022525 |
| 97 | 17348933 | NA | Mapre2 | microtubule-associated protein, RP/EB family, member 2 | 212307 | ENSMUSG00000024277 |
| 98 | 17266489 | NA | Tmem97 | transmembrane protein 97 | 69071 | ENSMUSG00000037278 |
| 99 | 17299353 | NA | Ktn1 | kinectin 1 | 16709 | ENSMUSG00000021843 |
| 100 | 17506854 | NA | Tsnax | translin-associated factor X | 53424 | ENSMUSG00000056820 |
| 101 | 17413573 | NA | Grhpr | glyoxylate reductase/hydroxypyruvate reductase | 76238 | ENSMUSG00000035637 |
| 102 | 17288716 | NA | Glrx | glutaredoxin | 93692 | ENSMUSG00000021591 |
| 103 | 17429495 | NA | Nfyc | nuclear transcription factor-Y gamma | 18046 | ENSMUSG00000032897 |
| 104 | 17361855 | NA | Pola2 | polymerase (DNA directed), alpha 2 | 18969 | ENSMUSG00000024833 |
| 105 | 17527532 | NA | Mpi | mannose phosphate isomerase | 110119 | ENSMUSG00000032306 |
| 106 | 17512463 | NA | Atp6v0d1 | ATPase, H+ transporting, lysosomal V0 subunit D1 | 11972 | ENSMUSG00000013160 |
| 107 | 17512466 | NA | Atp6v0d1 | ATPase, H+ transporting, lysosomal V0 subunit D1 | 11972 | ENSMUSG00000013160 |
| 108 | 17252635 | NA | Shpk | sedoheptulokinase | 74637 | ENSMUSG00000005951 |
| 109 | 17313008 | NA | Cby1 | chibby homolog 1 (Drosophila) | 73739 | ENSMUSG00000022428 |
| 110 | 17535434 | NA | Nsdhl | NAD(P) dependent steroid dehydrogenase-like | 18194 | ENSMUSG00000031349 |
| 111 | 17503023 | NA | Asf1b | ASF1 anti-silencing function 1 homolog B (S. cerevisiae) | 66929 | ENSMUSG00000005470 |
| 112 | 17328829 | NA | Slc25a1 | solute carrier family 25 (mitochondrial carrier, citrate transporter), member 1 | 13358 | ENSMUSG00000003528 |
| 113 | 17454256 | NA | Taf6 | TAF6 RNA polymerase II, TATA box binding protein (TBP)-associated factor | 21343 | ENSMUSG00000036980 |
| 114 | 17432967 | NA | Ubiad1 | UbiA prenyltransferase domain containing 1 | 71707 | ENSMUSG00000047719 |
| 115 | 17279499 | NA | Crip2 | cysteine rich protein 2 | 68337 | ENSMUSG00000006356 |
| 116 | 17231118 | NA | Rcor3 | REST corepressor 3 | 214742 | ENSMUSG00000037395 |
| 117 | 17379554 | NA | Zswim1 | zinc finger, SWIM domain containing 1 | 71971 | ENSMUSG00000017764 |
| 118 | 17298407 | NA | Bap1 | Brca1 associated protein 1 | 104416 | ENSMUSG00000021901 |
| 119 | 17280897 | NA | Stxbp6 | syntaxin binding protein 6 (amisyn) | 217517 | ENSMUSG00000046314 |
| 120 | 17451443 | NA | Coro1c | coronin, actin binding protein 1C | 23790 | ENSMUSG00000004530 |
| 121 | 17273280 | NA | Stra13 | stimulated by retinoic acid 13 | 20892 | ENSMUSG00000025144 |
| 122 | 17301342 | NA | Ints9 | integrator complex subunit 9 | 210925 | ENSMUSG00000021975 |
| 123 | 17393357 | NA | Eif6 | eukaryotic translation initiation factor 6 | 16418 | ENSMUSG00000027613 |
| 124 | 17412593 | NA | Srsf12 | serine/arginine-rich splicing factor 12 | 272009 | ENSMUSG00000054679 |
| 125 | 17451816 | NA | Hspb8 | heat shock protein 8 | 80888 | ENSMUSG00000041548 |
| 126 | 17504160 | NA | Polr2c | polymerase (RNA) II (DNA directed) polypeptide C | 20021 | ENSMUSG00000031783 |
| 127 | 17455093 | NA | Zkscan14 | zinc finger with KRAB and SCAN domains 14 | 67235 | ENSMUSG00000029627 |
| 128 | 17488544 | NA | Nfkbib | nuclear factor of kappa light polypeptide gene enhancer in B cells inhibitor, beta | 18036 | ENSMUSG00000030595 |
| 129 | 17328810 | NA | Dgcr14 | DiGeorge syndrome critical region gene 14 | 27886 | ENSMUSG00000003527 |
| 130 | 17229466 | NA | Hsd17b7 | hydroxysteroid (17-beta) dehydrogenase 7 | 15490 | ENSMUSG00000026675 |
| 131 | 17242318 | NA | Gm7138 | predicted gene 7138 | 634517 | ENSMUSG00000095593 |
| 132 | 17463150 | NA | Vamp1 | vesicle-associated membrane protein 1 | 22317 | ENSMUSG00000030337 |
| 133 | 17306861 | NA | Dhrs1 | dehydrogenase/reductase (SDR family) member 1 | 52585 | ENSMUSG00000002332 |
| 134 | 17306864 | NA | Dhrs1 | dehydrogenase/reductase (SDR family) member 1 | 52585 | ENSMUSG00000002332 |
| 135 | 17306860 | NA | Dhrs1 | dehydrogenase/reductase (SDR family) member 1 | 52585 | ENSMUSG00000002332 |
| 136 | 17306856 | NA | Dhrs1 | dehydrogenase/reductase (SDR family) member 1 | 52585 | ENSMUSG00000002332 |
| 137 | 17306865 | NA | Dhrs1 | dehydrogenase/reductase (SDR family) member 1 | 52585 | ENSMUSG00000002332 |
| 138 | 17418571 | NA | Trappc3 | trafficking protein particle complex 3 | 27096 | ENSMUSG00000028847 |
| 139 | 17318942 | NA | Pvalb | parvalbumin | 19293 | ENSMUSG00000005716 |
| 140 | 17419206 | NA | Pef1 | penta-EF hand domain containing 1 | 67898 | ENSMUSG00000028779 |
| 141 | 17535752 | NA | Emd | emerin | 13726 | ENSMUSG00000001964 |
| 142 | 17396024 | NA | Stmn2 | stathmin-like 2 | 20257 | ENSMUSG00000027500 |
| 143 | 17338043 | NA | Yipf3 | Yip1 domain family, member 3 | 28064 | ENSMUSG00000071074 |
| 144 | 17313199 | NA | Adsl | adenylosuccinate lyase | 11564 | ENSMUSG00000022407 |
| 145 | 17418447 | NA | Meaf6 | MYST/Esa1-associated factor 6 | 70088 | ENSMUSG00000028863 |
| 146 | 17232215 | NA | Moxd1 | monooxygenase, DBH-like 1 | 59012 | ENSMUSG00000020000 |
| 147 | 17356202 | NA | Pold4 | polymerase (DNA-directed), delta 4 | 69745 | ENSMUSG00000024854 |
| 148 | 17240123 | NA | Clvs2 | clavesin 2 | 215890 | ENSMUSG00000019785 |
| 149 | 17462975 | NA | Mlf2 | myeloid leukemia factor 2 | 30853 | ENSMUSG00000030120 |
| 150 | 17255719 | NA | Mrpl10 | mitochondrial ribosomal protein L10 | 107732 | ENSMUSG00000001445 |
| 151 | 17257962 | NA | Sstr2 | somatostatin receptor 2 | 20606 | ENSMUSG00000047904 |
| 152 | 17344794 | NA | Znrd1 | zinc ribbon domain containing, 1 | 66136 | ENSMUSG00000036315 |
| 153 | 17232843 | NA | Zbtb24 | zinc finger and BTB domain containing 24 | 268294 | ENSMUSG00000019826 |
| 154 | 17300261 | NA | Oxa1l | oxidase assembly 1-like | 69089 | ENSMUSG00000000959 |
| 155 | 17224540 | NA | Tuba4a | tubulin, alpha 4A | 22145 | ENSMUSG00000026202 |
| 156 | 17456308 | NA | Kcnd2 | potassium voltage-gated channel, Shal-related family, member 2 | 16508 | ENSMUSG00000060882 |
| 157 | 17517723 | NA | Rpp25 | ribonuclease P 25 subunit (human) | 102614 | ENSMUSG00000062309 |
| 158 | 17307134 | NA | Cryl1 | crystallin, lambda 1 | 68631 | ENSMUSG00000021947 |
| 159 | 17521652 | NA | Nicn1 | nicolin 1 | 66257 | ENSMUSG00000032606 |
| 160 | 17369862 | NA | Dpm2 | dolichol-phosphate (beta-D) mannosyltransferase 2 | 13481 | ENSMUSG00000026810 |
| 161 | 17322559 | NA | Hmox2 | heme oxygenase (decycling) 2 | 15369 | ENSMUSG00000004070 |
| 162 | 17537677 | NA | Drp2 | dystrophin related protein 2 | 13497 | ENSMUSG00000000223 |
| 163 | 17375833 | NA | Tmem127 | transmembrane protein 127 | 69470 | ENSMUSG00000034850 |
| 164 | 17538096 | NA | Rnf128 | ring finger protein 128 | 66889 | ENSMUSG00000031438 |
| 165 | 17288160 | NA | Cdk20 | cyclin-dependent kinase 20 | 105278 | ENSMUSG00000021483 |
| 166 | 17263594 | NA | Atpaf2 | ATP synthase mitochondrial F1 complex assembly factor 2 | 246782 | ENSMUSG00000042709 |
| 167 | 17343617 | NA | Rab11b | RAB11B, member RAS oncogene family | 19326 | ENSMUSG00000077450 |
| 168 | 17447099 | NA | Ctbp1 | C-terminal binding protein 1 | 13016 | ENSMUSG00000037373 |
| 169 | 17447089 | NA | Ctbp1 | C-terminal binding protein 1 | 13016 | ENSMUSG00000037373 |
| 170 | 17447100 | NA | Ctbp1 | C-terminal binding protein 1 | 13016 | ENSMUSG00000037373 |
| 171 | 17447098 | NA | Ctbp1 | C-terminal binding protein 1 | 13016 | ENSMUSG00000037373 |
| 172 | 17344336 | NA | Tcf19 | transcription factor 19 | 106795 | ENSMUSG00000050410 |
| 173 | 17433602 | NA | Tprgl | transformation related protein 63 regulated like | 67808 | ENSMUSG00000029030 |
| 174 | 17265082 | NA | Eif5a | eukaryotic translation initiation factor 5A | 276770 | ENSMUSG00000078812 |
| 175 | 17233630 | NA | Psap | prosaposin | 19156 | ENSMUSG00000004207 |
| 176 | 17235368 | NA | Scamp4 | secretory carrier membrane protein 4 | 56214 | ENSMUSG00000078441 |
| 177 | 17369672 | NA | Ppapdc3 | phosphatidic acid phosphatase type 2 domain containing 3 | 227721 | ENSMUSG00000051373 |
| 178 | 17512434 | NA | Tppp3 | tubulin polymerization-promoting protein family member 3 | 67971 | ENSMUSG00000014846 |
| 179 | 17225499 | NA | Hes6 | hairy and enhancer of split 6 (Drosophila) | 55927 | ENSMUSG00000067071 |
| 180 | 17265175 | NA | 0610010K14Rik | RIKEN cDNA 0610010K14 gene | 104457 | ENSMUSG00000020831 |
| 181 | 17377583 | NA | Nsfl1c | NSFL1 (p97) cofactor (p47) | 386649 | ENSMUSG00000027455 |
| 182 | 17404628 | NA | Pex5l | peroxisomal biogenesis factor 5-like | 58869 | ENSMUSG00000027674 |
| 183 | 17517576 | NA | Hmg20a | high mobility group 20A | 66867 | ENSMUSG00000032329 |
| 184 | 17393225 | NA | Pigu | phosphatidylinositol glycan anchor biosynthesis, class U | 228812 | ENSMUSG00000038383 |
| 185 | 17404011 | NA | Hey1 | hairy/enhancer-of-split related with YRPW motif 1 | 15213 | ENSMUSG00000040289 |
| 186 | 17246850 | NA | Zmat5 | zinc finger, matrin type 5 | 67178 | ENSMUSG00000009076 |
| 187 | 17238846 | NA | Syne1 | synaptic nuclear envelope 1 | 64009 | ENSMUSG00000019769 ENSMUSG00000096054 |
| 188 | 17238906 | NA | Syne1 | synaptic nuclear envelope 1 | 64009 | ENSMUSG00000019769 ENSMUSG00000096054 |
| 189 | 17238890 | NA | Syne1 | synaptic nuclear envelope 1 | 64009 | ENSMUSG00000019769 ENSMUSG00000096054 |
| 190 | 17502191 | NA | Mrpl34 | mitochondrial ribosomal protein L34 | 94065 | ENSMUSG00000034880 |
| 191 | 17277370 | NA | Eif2b2 | eukaryotic translation initiation factor 2B, subunit 2 beta | 217715 | ENSMUSG00000004788 |
| 192 | 17316625 | NA | Ubr5 | ubiquitin protein ligase E3 component n-recognin 5 | 70790 | ENSMUSG00000037487 |
| 193 | 17512009 | NA | Csnk2a2 | casein kinase 2, alpha prime polypeptide | 13000 | ENSMUSG00000046707 |
| 194 | 17245709 | NA | Os9 | amplified in osteosarcoma | 216440 | ENSMUSG00000040462 |
| 195 | 17503816 | NA | Irx6 | Iroquois related homeobox 6 (Drosophila) | 64379 | ENSMUSG00000031738 |
| 196 | 17282649 | NA | Rps6kl1 | ribosomal protein S6 kinase-like 1 | 238323 | ENSMUSG00000019235 |
| 197 | 17365493 | NA | Obfc1 | oligonucleotide/oligosaccharide-binding fold containing 1 | 108689 | ENSMUSG00000042694 |
| 198 | 17538790 | NA | Huwe1 | HECT, UBA and WWE domain containing 1 | 59026 | ENSMUSG00000025261 |
| 199 | 17470879 | NA | Tpi1 | triosephosphate isomerase 1 | 21991 | ENSMUSG00000023456 |
| 200 | 17229644 | NA | Tomm40l | translocase of outer mitochondrial membrane 40 homolog-like (yeast) | 641376 | ENSMUSG00000005674 |
| 201 | 17522887 | NA | Golga4 | golgi autoantigen, golgin subfamily a, 4 | 54214 | ENSMUSG00000038708 |
| 202 | 17230408 | NA | Adck3 | aarF domain containing kinase 3 | 67426 | ENSMUSG00000026489 |
| 203 | 17352132 | NA | Txnl4a | thioredoxin-like 4A | 27366 | ENSMUSG00000057130 |
| 204 | 17420582 | NA | Capzb | capping protein (actin filament) muscle Z-line, beta | 12345 | ENSMUSG00000028745 |
| 205 | 17273086 | NA | Nploc4 | nuclear protein localization 4 homolog (S. cerevisiae) | 217365 | ENSMUSG00000039703 |
| 206 | 17275955 | NA | Atl1 | atlastin GTPase 1 | 73991 | ENSMUSG00000021066 |
| 207 | 17431502 | NA | Lypla2 | lysophospholipase 2 | 26394 | ENSMUSG00000028670 |
| 208 | 17500275 | NA | Erlin2 | ER lipid raft associated 2 | 244373 | ENSMUSG00000031483 |
| 209 | 17256565 | NA | Tubg2 | tubulin, gamma 2 | 103768 | ENSMUSG00000045007 |
| 210 | 17463422 | NA | Nrip2 | nuclear receptor interacting protein 2 | 60345 | ENSMUSG00000001520 |
| 211 | 17299542 | NA | Tmem55b | transmembrane protein 55b | 219024 | ENSMUSG00000035953 |
| 212 | 17485194 | NA | Krtap5-4 | keratin associated protein 5-4 | 50775 | ENSMUSG00000045236 |
| 213 | 17253674 | NA | Poldip2 | polymerase (DNA-directed), delta interacting protein 2 | 67811 | ENSMUSG00000001100 |
| 214 | 17213153 | NA | Nif3l1 | Ngg1 interacting factor 3-like 1 (S. pombe) | 65102 | ENSMUSG00000026036 |
| 215 | 17321722 | NA | Tfcp2 | transcription factor CP2 | 21422 | ENSMUSG00000009733 |
| 216 | 17405819 | NA | B3galnt1 | UDP-GalNAc:betaGlcNAc beta 1,3-galactosaminyltransferase, polypeptide 1 | 26879 | ENSMUSG00000043300 |
| 217 | 17498821 | NA | Snapc2 | small nuclear RNA activating complex, polypeptide 2 | 102209 | ENSMUSG00000011837 |
| 218 | 17328451 | NA | Mzt2 | mitotic spindle organizing protein 2 | 72083 | ENSMUSG00000022671 |
| 219 | 17256618 | NA | Vps25 | vacuolar protein sorting 25 (yeast) | 28084 | ENSMUSG00000078656 |
| 220 | 17494637 | NA | Mrpl17 | mitochondrial ribosomal protein L17 | 27397 | ENSMUSG00000030879 |
| 221 | 17517532 | NA | Isl2 | insulin related protein 2 (islet 2) | 104360 | ENSMUSG00000032318 |
| 222 | 17513871 | NA | Chmp1a | charged multivesicular body protein 1A | 234852 | ENSMUSG00000000743 |
| 223 | 17269638 | NA | Rab5c | RAB5C, member RAS oncogene family | 19345 | ENSMUSG00000019173 |
| 224 | 17256549 | NA | Tubg1 | tubulin, gamma 1 | 103733 | ENSMUSG00000035198 |
| 225 | 17214293 | NA | Bcs1l | BCS1-like (yeast) | 66821 | ENSMUSG00000026172 |
| 226 | 17497957 | NA | Chid1 | chitinase domain containing 1 | 68038 | ENSMUSG00000025512 |
| 227 | 17532045 | NA | Plcd1 | phospholipase C, delta 1 | 18799 | ENSMUSG00000010660 |
| 228 | 17274448 | NA | Cpsf3 | cleavage and polyadenylation specificity factor 3 | 54451 | ENSMUSG00000054309 |
| 229 | 17332333 | NA | Dscr3 | Down syndrome critical region gene 3 | 13185 | ENSMUSG00000022898 |
| 230 | 17332341 | NA | Dscr3 | Down syndrome critical region gene 3 | 13185 | ENSMUSG00000022898 |
| 231 | 17332343 | NA | Dscr3 | Down syndrome critical region gene 3 | 13185 | ENSMUSG00000022898 |
| 232 | 17332336 | NA | Dscr3 | Down syndrome critical region gene 3 | 13185 | ENSMUSG00000022898 |
| 233 | 17519868 | NA | Htr1b | 5-hydroxytryptamine (serotonin) receptor 1B | 15551 | ENSMUSG00000049511 |
| 234 | 17515170 | NA | Ilf3 | interleukin enhancer binding factor 3 | 16201 | ENSMUSG00000032178 |
| 235 | 17533055 | NA | Timm17b | translocase of inner mitochondrial membrane 17b | 21855 | ENSMUSG00000031158 |
| 236 | 17430853 | NA | Med18 | mediator of RNA polymerase II transcription, subunit 18 homolog (yeast) | 67219 | ENSMUSG00000066042 |
| 237 | 17442714 | NA | Bri3bp | Bri3 binding protein | 76809 | ENSMUSG00000037905 |
| 238 | 17521014 | NA | Acad11 | acyl-Coenzyme A dehydrogenase family, member 11 | 102632 | ENSMUSG00000090150 |
| 239 | 17370285 | NA | Mrrf | mitochondrial ribosome recycling factor | 67871 | ENSMUSG00000026887 |
| 240 | 17229948 | NA | Dusp23 | dual specificity phosphatase 23 | 68440 | ENSMUSG00000026544 |
| 241 | 17511296 | NA | Wdr83 | WD repeat domain containing 83 | 67836 | ENSMUSG00000005150 |
| 242 | 17539434 | NA | Ctps2 | cytidine 5'-triphosphate synthase 2 | 55936 | ENSMUSG00000031360 |
| 243 | 17250141 | NA | Zfp39 | zinc finger protein 39 | 22698 | ENSMUSG00000037001 |
| 244 | 17341521 | NA | Thoc6 | THO complex 6 homolog (Drosophila) | 386612 | ENSMUSG00000041319 |
| 245 | 17266322 | NA | Eral1 | Era (G-protein)-like 1 (E. coli) | 57837 | ENSMUSG00000020832 |
| 246 | 17521422 | NA | Hyal2 | hyaluronoglucosaminidase 2 | 15587 | ENSMUSG00000010047 |
| 247 | 17540589 | NA | Klhl13 | kelch-like 13 (Drosophila) | 67455 | ENSMUSG00000036782 |
| 248 | 17376272 | NA | Nop56 | NOP56 ribonucleoprotein homolog (yeast) | 67134 | ENSMUSG00000027405 |
| 249 | 17468195 | NA | Stambp | STAM binding protein | 70527 | ENSMUSG00000006906 |
| 250 | 17342015 | NA | Tbl3 | transducin (beta)-like 3 | 213773 | ENSMUSG00000040688 |
| 251 | 17509721 | NA | Tufm | Tu translation elongation factor, mitochondrial | 233870 | ENSMUSG00000073838 |
| 252 | 17342617 | NA | Arhgdig | Rho GDP dissociation inhibitor (GDI) gamma | 14570 | ENSMUSG00000073433 |
| 253 | 17361463 | NA | Rab1b | RAB1B, member RAS oncogene family | 76308 | ENSMUSG00000024870 |
| 254 | 17279858 | NA | Fkbp1b | FK506 binding protein 1b | 14226 | ENSMUSG00000020635 |
| 255 | 17217580 | NA | Arl8a | ADP-ribosylation factor-like 8A | 68724 | ENSMUSG00000026426 |
| 256 | 17460099 | NA | Vax2 | ventral anterior homeobox containing gene 2 | 24113 | ENSMUSG00000034777 |
| 257 | 17257060 | NA | Nmt1 | N-myristoyltransferase 1 | 18107 | ENSMUSG00000020936 |
| 258 | 17365369 | NA | Cuedc2 | CUE domain containing 2 | 67116 | ENSMUSG00000036748 |
| 259 | 17292107 | NA | Tbc1d7 | TBC1 domain family, member 7 | 67046 | ENSMUSG00000021368 |
| 260 | 17512479 | NA | Acd | adrenocortical dysplasia | 497652 | ENSMUSG00000038000 |
| 261 | 17540050 | NA | Ebp | phenylalkylamine Ca2+ antagonist (emopamil) binding protein | 13595 | ENSMUSG00000031168 |
| 262 | 17232426 | NA | Echdc1 | enoyl Coenzyme A hydratase domain containing 1 | 52665 | ENSMUSG00000019883 |
| 263 | 17212813 | NA | Mstn | myostatin | 17700 | ENSMUSG00000026100 |
| 264 | 17230945 | NA | Smyd2 | SET and MYND domain containing 2 | 226830 | ENSMUSG00000026603 |
| 265 | 17521448 | NA | Hyal3 | hyaluronoglucosaminidase 3 | 109685 | ENSMUSG00000036091 |
| 266 | 17251607 | NA | Trappc1 | trafficking protein particle complex 1 | 245828 | ENSMUSG00000049299 |
| 267 | 17502390 | NA | Rab8a | RAB8A, member RAS oncogene family | 17274 | ENSMUSG00000003037 |
| 268 | 17468113 | NA | Ino80b | INO80 complex subunit B | 70020 | ENSMUSG00000030034 |
| 269 | 17299750 | NA | Tox4 | TOX high mobility group box family member 4 | 268741 | ENSMUSG00000016831 |
| 270 | 17347558 | NA | Cdkl4 | cyclin-dependent kinase-like 4 | 381113 | ENSMUSG00000033966 |
| 271 | 17258457 | NA | Sap30bp | SAP30 binding protein | 57230 | ENSMUSG00000020755 |
| 272 | 17283939 | NA | Wars | tryptophanyl-tRNA synthetase | 22375 | ENSMUSG00000021266 |
| 273 | 17283941 | NA | Wars | tryptophanyl-tRNA synthetase | 22375 | ENSMUSG00000021266 |
| 274 | 17283930 | NA | Wars | tryptophanyl-tRNA synthetase | 22375 | ENSMUSG00000021266 |
| 275 | 17283938 | NA | Wars | tryptophanyl-tRNA synthetase | 22375 | ENSMUSG00000021266 |
| 276 | 17512872 | NA | Calb2 | calbindin 2 | 12308 | ENSMUSG00000003657 |
| 277 | 17510365 | NA | Pgls | 6-phosphogluconolactonase | 66171 | ENSMUSG00000031807 |
| 278 | 17526273 | NA | Trappc4 | trafficking protein particle complex 4 | 60409 | ENSMUSG00000032112 |
| 279 | 17526271 | NA | Trappc4 | trafficking protein particle complex 4 | 60409 | ENSMUSG00000032112 |
| 280 | 17526272 | NA | Trappc4 | trafficking protein particle complex 4 | 60409 | ENSMUSG00000032112 |
| 281 | 17465696 | NA | Slc35b4 | solute carrier family 35, member B4 | 58246 | ENSMUSG00000018999 |
| 282 | 17318877 | NA | Txn2 | thioredoxin 2 | 56551 | ENSMUSG00000005354 |
| 283 | 17453809 | NA | Ap1s1 | adaptor protein complex AP-1, sigma 1 | 11769 | ENSMUSG00000004849 |
| 284 | 17329163 | NA | Camk2n2 | calcium/calmodulin-dependent protein kinase II inhibitor 2 | 73047 | ENSMUSG00000051146 |
| 285 | 17405174 | NA | Cog6 | component of oligomeric golgi complex 6 | 67542 | ENSMUSG00000027742 |
| 286 | 17382914 | NA | Dnlz | DNL-type zinc finger | 52838 | ENSMUSG00000075467 |
| 287 | 17221014 | NA | Cd34 | CD34 antigen | 12490 | ENSMUSG00000016494 |
| 288 | 17241409 | NA | Srgn | serglycin | 19073 | ENSMUSG00000020077 |
| 289 | 17322200 | NA | Aaas | achalasia, adrenocortical insufficiency, alacrimia | 223921 | ENSMUSG00000036678 |
| 290 | 17213990 | NA | Atic | 5-aminoimidazole-4-carboxamide ribonucleotide formyltransferase/IMP cyclohydrolase | 108147 | ENSMUSG00000026192 |
| 291 | 17508691 | NA | Rbpms | RNA binding protein gene with multiple splicing | 19663 | ENSMUSG00000031586 |
| 292 | 17222001 | NA | Prim2 | DNA primase, p58 subunit | 19076 | ENSMUSG00000026134 |
| 293 | 17232649 | NA | Fyn | Fyn proto-oncogene | 14360 | ENSMUSG00000019843 |
| 294 | 17474534 | NA | Opa3 | optic atrophy 3 | 403187 | ENSMUSG00000052214 |
| 295 | 17395844 | NA | Stmn3 | stathmin-like 3 | 20262 | ENSMUSG00000027581 |
| 296 | 17219286 | NA | Dedd | death effector domain-containing | 21945 | ENSMUSG00000013973 |
| 297 | 17241780 | NA | Ube2d1 | ubiquitin-conjugating enzyme E2D 1 | 216080 | ENSMUSG00000019927 |
| 298 | 17359945 | NA | Gbf1 | golgi-specific brefeldin A-resistance factor 1 | 107338 | ENSMUSG00000025224 |
| 299 | 17334495 | NA | Nme3 | NME/NM23 nucleoside diphosphate kinase 3 | 79059 | ENSMUSG00000073435 |
| 300 | 17383588 | NA | Ccbl1 | cysteine conjugate-beta lyase 1 | 70266 | ENSMUSG00000039648 |
| 301 | 17400638 | NA | Pex11b | peroxisomal biogenesis factor 11 beta | 18632 | ENSMUSG00000028102 |
| 302 | 17369952 | NA | Sh2d3c | SH2 domain containing 3C | 27387 | ENSMUSG00000059013 |
| 303 | 17421972 | NA | Errfi1 | ERBB receptor feedback inhibitor 1 | 74155 | ENSMUSG00000028967 |

  
  

| **Database:cellular component      &nbspName:intracellular      &nbspID:GO:0005622** | | | | | | |
| --- | --- | --- | --- | --- | --- | --- |
| C=10950; O=286; E=211.61; R=1.35; rawP=9.30e-15; adjP=1.08e-12 | | | | | | |
| Index | UserID | Value | Gene Symbol | Gene Name | EntrezGene | Ensembl |
| 1 | 17476273 | NA | Zfp382 | zinc finger protein 382 | 233060 | ENSMUSG00000074220 |
| 2 | 17413221 | NA | Unc13b | unc-13 homolog B (C. elegans) | 22249 | ENSMUSG00000028456 |
| 3 | 17474547 | NA | Rtn2 | reticulon 2 (Z-band associated protein) | 20167 | ENSMUSG00000030401 |
| 4 | 17512740 | NA | Nob1 | NIN1/RPN12 binding protein 1 homolog (S. cerevisiae) | 67619 | ENSMUSG00000003848 |
| 5 | 17503910 | NA | Ogfod1 | 2-oxoglutarate and iron-dependent oxygenase domain containing 1 | 270086 | ENSMUSG00000033009 |
| 6 | 17368171 | NA | Bmyc | brain expressed myelocytomatosis oncogene | 107771 | ENSMUSG00000049086 |
| 7 | 17350134 | NA | Pou4f3 | POU domain, class 4, transcription factor 3 | 18998 | ENSMUSG00000024497 |
| 8 | 17443181 | NA | Dnajc30 | DnaJ (Hsp40) homolog, subfamily C, member 30 | 66114 | ENSMUSG00000061118 |
| 9 | 17319324 | NA | Dnalc4 | dynein, axonemal, light chain 4 | 54152 | ENSMUSG00000022420 |
| 10 | 17512103 | NA | Got2 | glutamate oxaloacetate transaminase 2, mitochondrial | 14719 | ENSMUSG00000031672 |
| 11 | 17336829 | NA | Lsm2 | LSM2 homolog, U6 small nuclear RNA associated (S. cerevisiae) | 27756 | ENSMUSG00000007050 |
| 12 | 17288454 | NA | Irx4 | Iroquois related homeobox 4 (Drosophila) | 50916 | ENSMUSG00000021604 |
| 13 | 17467996 | NA | Mrpl19 | mitochondrial ribosomal protein L19 | 56284 | ENSMUSG00000030045 |
| 14 | 17306758 | NA | Tm9sf1 | transmembrane 9 superfamily member 1 | 74140 | ENSMUSG00000002320 |
| 15 | 17504712 | NA | Exoc3l | exocyst complex component 3-like | 277978 | ENSMUSG00000043251 |
| 16 | 17321467 | NA | Tuba1b | tubulin, alpha 1B | 22143 | ENSMUSG00000023004 |
| 17 | 17541597 | NA | Frmd7 | FERM domain containing 7 | 385354 | ENSMUSG00000036131 |
| 18 | 17379187 | NA | Ift52 | intraflagellar transport 52 | 245866 | ENSMUSG00000017858 |
| 19 | 17246284 | NA | Suox | sulfite oxidase | 211389 | ENSMUSG00000049858 |
| 20 | 17437611 | NA | Pgm1 | phosphoglucomutase 1 | 66681 | ENSMUSG00000029171 |
| 21 | 17287579 | NA | Zfp346 | zinc finger protein 346 | 26919 | ENSMUSG00000021481 |
| 22 | 17224577 | NA | Resp18 | regulated endocrine-specific protein 18 | 19711 | ENSMUSG00000033061 |
| 23 | 17297750 | NA | Ppif | peptidylprolyl isomerase F (cyclophilin F) | 105675 | ENSMUSG00000021868 |
| 24 | 17520177 | NA | Mthfs | 5, 10-methenyltetrahydrofolate synthetase | 107885 | ENSMUSG00000066442 |
| 25 | 17480102 | NA | Sytl2 | synaptotagmin-like 2 | 83671 | ENSMUSG00000030616 |
| 26 | 17521143 | NA | Wdr82 | WD repeat domain containing 82 | 77305 | ENSMUSG00000020257 |
| 27 | 17312944 | NA | Polr2f | polymerase (RNA) II (DNA directed) polypeptide F | 69833 | ENSMUSG00000033020 |
| 28 | 17312939 | NA | Polr2f | polymerase (RNA) II (DNA directed) polypeptide F | 69833 | ENSMUSG00000033020 |
| 29 | 17312941 | NA | Polr2f | polymerase (RNA) II (DNA directed) polypeptide F | 69833 | ENSMUSG00000033020 |
| 30 | 17329151 | NA | Alg3 | asparagine-linked glycosylation 3 (alpha-1,3-mannosyltransferase) | 208624 | ENSMUSG00000033809 |
| 31 | 17500391 | NA | Rnf122 | ring finger protein 122 | 68867 | ENSMUSG00000039328 |
| 32 | 17400222 | NA | Vps72 | vacuolar protein sorting 72 (yeast) | 21427 | ENSMUSG00000008958 |
| 33 | 17499396 | NA | Fbxo25 | F-box protein 25 | 66822 | ENSMUSG00000038365 |
| 34 | 17324664 | NA | Dlg1 | discs, large homolog 1 (Drosophila) | 13383 | ENSMUSG00000022770 |
| 35 | 17396369 | NA | Nceh1 | arylacetamide deacetylase-like 1 | 320024 | ENSMUSG00000027698 |
| 36 | 17314556 | NA | Slc48a1 | solute carrier family 48 (heme transporter), member 1 | 67739 | ENSMUSG00000081534 |
| 37 | 17246209 | NA | Rpl41 | ribosomal protein L41 | 67945 | ENSMUSG00000093674 |
| 38 | 17529231 | NA | Phip | pleckstrin homology domain interacting protein | 83946 | ENSMUSG00000032253 |
| 39 | 17483220 | NA | Cdipt | CDP-diacylglycerol--inositol 3-phosphatidyltransferase (phosphatidylinositol synthase) | 52858 | ENSMUSG00000030682 |
| 40 | 17361975 | NA | Snx15 | sorting nexin 15 | 69024 | ENSMUSG00000024787 |
| 41 | 17424319 | NA | Sigmar1 | sigma non-opioid intracellular receptor 1 | 18391 | ENSMUSG00000036078 |
| 42 | 17260221 | NA | Pold2 | polymerase (DNA directed), delta 2, regulatory subunit | 18972 | ENSMUSG00000020471 |
| 43 | 17446580 | NA | Shh | sonic hedgehog | 20423 | ENSMUSG00000002633 |
| 44 | 17281971 | NA | Sgpp1 | sphingosine-1-phosphate phosphatase 1 | 81535 | ENSMUSG00000021054 |
| 45 | 17238549 | NA | Wibg | within bgcn homolog (Drosophila) | 78428 | ENSMUSG00000064030 |
| 46 | 17212355 | NA | Nck2 | non-catalytic region of tyrosine kinase adaptor protein 2 | 17974 | ENSMUSG00000066877 |
| 47 | 17307695 | NA | Msra | methionine sulfoxide reductase A | 110265 | ENSMUSG00000054733 |
| 48 | 17303496 | NA | Fezf2 | Fez family zinc finger 2 | 54713 | ENSMUSG00000021743 |
| 49 | 17345519 | NA | Rrp36 | ribosomal RNA processing 36 homolog (S. cerevisiae) | 224823 | ENSMUSG00000023971 |
| 50 | 17211335 | NA | Tfap2d | transcription factor AP-2, delta | 226896 | ENSMUSG00000042596 |
| 51 | 17506631 | NA | Tubb3 | tubulin, beta 3 class III | 22152 | ENSMUSG00000062380 |
| 52 | 17235584 | NA | Dapk3 | death-associated protein kinase 3 | 13144 | ENSMUSG00000034974 |
| 53 | 17338670 | NA | Fsd1 | fibronectin type 3 and SPRY domain-containing protein | 240121 | ENSMUSG00000011589 |
| 54 | 17300411 | NA | Thtpa | thiamine triphosphatase | 105663 | ENSMUSG00000045691 |
| 55 | 17460879 | NA | Hdac11 | histone deacetylase 11 | 232232 | ENSMUSG00000034245 |
| 56 | 17444100 | NA | Chst12 | carbohydrate sulfotransferase 12 | 59031 | ENSMUSG00000036599 |
| 57 | 17359994 | NA | Fbxl15 | F-box and leucine-rich repeat protein 15 | 68431 | ENSMUSG00000025226 |
| 58 | 17217666 | NA | Tmem9 | transmembrane protein 9 | 66241 | ENSMUSG00000026411 |
| 59 | 17484068 | NA | Lhpp | phospholysine phosphohistidine inorganic pyrophosphate phosphatase | 76429 | ENSMUSG00000030946 |
| 60 | 17497366 | NA | Ebf3 | early B cell factor 3 | 13593 | ENSMUSG00000010476 |
| 61 | 17288616 | NA | Tppp | tubulin polymerization promoting protein | 72948 | ENSMUSG00000021573 |
| 62 | 17446322 | NA | Prkag2 | protein kinase, AMP-activated, gamma 2 non-catalytic subunit | 108099 | ENSMUSG00000028944 |
| 63 | 17452552 | NA | Rhof | ras homolog gene family, member f | 23912 | ENSMUSG00000029449 |
| 64 | 17407764 | NA | Prune | prune homolog (Drosophila) | 229589 | ENSMUSG00000015711 |
| 65 | 17524523 | NA | Eif3g | eukaryotic translation initiation factor 3, subunit G | 53356 | ENSMUSG00000070319 |
| 66 | 17268995 | NA | Krt222 | keratin 222 | 268481 | ENSMUSG00000035849 |
| 67 | 17505367 | NA | Txnl4b | thioredoxin-like 4B | 234723 | ENSMUSG00000031723 |
| 68 | 17540982 | NA | Sept6 | septin 6 | 56526 | ENSMUSG00000050379 |
| 69 | 17235268 | NA | Ndufs7 | NADH dehydrogenase (ubiquinone) Fe-S protein 7 | 75406 | ENSMUSG00000020153 |
| 70 | 17447726 | NA | Hs3st1 | heparan sulfate (glucosamine) 3-O-sulfotransferase 1 | 15476 | ENSMUSG00000051022 |
| 71 | 17454416 | NA | Zfand2a | zinc finger, AN1-type domain 2A | 100494 | ENSMUSG00000053581 |
| 72 | 17422117 | NA | Acot7 | acyl-CoA thioesterase 7 | 70025 | ENSMUSG00000028937 |
| 73 | 17536264 | NA | Pcyt1b | phosphate cytidylyltransferase 1, choline, beta isoform | 236899 | ENSMUSG00000035246 |
| 74 | 17357213 | NA | Zbtb3 | zinc finger and BTB domain containing 3 | 75291 | ENSMUSG00000071661 |
| 75 | 17453106 | NA | Zfp11 | zinc finger protein 11 | 22648 | ENSMUSG00000051034 |
| 76 | 17315558 | NA | Copz1 | coatomer protein complex, subunit zeta 1 | 56447 | ENSMUSG00000060992 |
| 77 | 17361988 | NA | Arl2 | ADP-ribosylation factor-like 2 | 56327 | ENSMUSG00000024944 |
| 78 | 17502583 | NA | Mcm5 | minichromosome maintenance deficient 5, cell division cycle 46 (S. cerevisiae) | 17218 | ENSMUSG00000005410 |
| 79 | 17485815 | NA | Syt5 | synaptotagmin V | 53420 | ENSMUSG00000004961 |
| 80 | 17519718 | NA | Mto1 | mitochondrial translation optimization 1 homolog (S. cerevisiae) | 68291 | ENSMUSG00000032342 |
| 81 | 17488134 | NA | Rab4b | RAB4B, member RAS oncogene family | 19342 | ENSMUSG00000053291 |
| 82 | 17344453 | NA | Ppp1r18 | protein phosphatase 1, regulatory subunit 18 | 76448 | ENSMUSG00000034595 |
| 83 | 17317208 | NA | Derl1 | Der1-like domain family, member 1 | 67819 | ENSMUSG00000022365 |
| 84 | 17501544 | NA | Npy1r | neuropeptide Y receptor Y1 | 18166 | ENSMUSG00000036437 |
| 85 | 17428858 | NA | Rnf220 | ring finger protein 220 | 66743 | ENSMUSG00000028677 |
| 86 | 17428857 | NA | Rnf220 | ring finger protein 220 | 66743 | ENSMUSG00000028677 |
| 87 | 17408684 | NA | Dclre1b | DNA cross-link repair 1B, PSO2 homolog (S. cerevisiae) | 140917 | ENSMUSG00000027845 |
| 88 | 17443047 | NA | Caln1 | calneuron 1 | 140904 | ENSMUSG00000060371 |
| 89 | 17436077 | NA | Nrbp1 | nuclear receptor binding protein 1 | 192292 | ENSMUSG00000029148 |
| 90 | 17252170 | NA | Rnf167 | ring finger protein 167 | 70510 | ENSMUSG00000040746 |
| 91 | 17543988 | NA | Taf9b | TAF9B RNA polymerase II, TATA box binding protein (TBP)-associated factor | 407786 | ENSMUSG00000047242 |
| 92 | 17459676 | NA | Retsat | retinol saturase (all trans retinol 13,14 reductase) | 67442 | ENSMUSG00000056666 |
| 93 | 17337269 | NA | Nrm | nurim (nuclear envelope membrane protein) | 106582 | ENSMUSG00000059791 |
| 94 | 17488463 | NA | Med29 | mediator complex subunit 29 | 67224 | ENSMUSG00000003444 |
| 95 | 17391270 | NA | Kcnip3 | Kv channel interacting protein 3, calsenilin | 56461 | ENSMUSG00000079056 |
| 96 | 17324576 | NA | Hrasls | HRAS-like suppressor | 27281 | ENSMUSG00000022525 |
| 97 | 17348933 | NA | Mapre2 | microtubule-associated protein, RP/EB family, member 2 | 212307 | ENSMUSG00000024277 |
| 98 | 17266489 | NA | Tmem97 | transmembrane protein 97 | 69071 | ENSMUSG00000037278 |
| 99 | 17299353 | NA | Ktn1 | kinectin 1 | 16709 | ENSMUSG00000021843 |
| 100 | 17506854 | NA | Tsnax | translin-associated factor X | 53424 | ENSMUSG00000056820 |
| 101 | 17413573 | NA | Grhpr | glyoxylate reductase/hydroxypyruvate reductase | 76238 | ENSMUSG00000035637 |
| 102 | 17288716 | NA | Glrx | glutaredoxin | 93692 | ENSMUSG00000021591 |
| 103 | 17429495 | NA | Nfyc | nuclear transcription factor-Y gamma | 18046 | ENSMUSG00000032897 |
| 104 | 17361855 | NA | Pola2 | polymerase (DNA directed), alpha 2 | 18969 | ENSMUSG00000024833 |
| 105 | 17527532 | NA | Mpi | mannose phosphate isomerase | 110119 | ENSMUSG00000032306 |
| 106 | 17512463 | NA | Atp6v0d1 | ATPase, H+ transporting, lysosomal V0 subunit D1 | 11972 | ENSMUSG00000013160 |
| 107 | 17512466 | NA | Atp6v0d1 | ATPase, H+ transporting, lysosomal V0 subunit D1 | 11972 | ENSMUSG00000013160 |
| 108 | 17252635 | NA | Shpk | sedoheptulokinase | 74637 | ENSMUSG00000005951 |
| 109 | 17313008 | NA | Cby1 | chibby homolog 1 (Drosophila) | 73739 | ENSMUSG00000022428 |
| 110 | 17535434 | NA | Nsdhl | NAD(P) dependent steroid dehydrogenase-like | 18194 | ENSMUSG00000031349 |
| 111 | 17503023 | NA | Asf1b | ASF1 anti-silencing function 1 homolog B (S. cerevisiae) | 66929 | ENSMUSG00000005470 |
| 112 | 17328829 | NA | Slc25a1 | solute carrier family 25 (mitochondrial carrier, citrate transporter), member 1 | 13358 | ENSMUSG00000003528 |
| 113 | 17454256 | NA | Taf6 | TAF6 RNA polymerase II, TATA box binding protein (TBP)-associated factor | 21343 | ENSMUSG00000036980 |
| 114 | 17432967 | NA | Ubiad1 | UbiA prenyltransferase domain containing 1 | 71707 | ENSMUSG00000047719 |
| 115 | 17279499 | NA | Crip2 | cysteine rich protein 2 | 68337 | ENSMUSG00000006356 |
| 116 | 17231118 | NA | Rcor3 | REST corepressor 3 | 214742 | ENSMUSG00000037395 |
| 117 | 17379554 | NA | Zswim1 | zinc finger, SWIM domain containing 1 | 71971 | ENSMUSG00000017764 |
| 118 | 17298407 | NA | Bap1 | Brca1 associated protein 1 | 104416 | ENSMUSG00000021901 |
| 119 | 17280897 | NA | Stxbp6 | syntaxin binding protein 6 (amisyn) | 217517 | ENSMUSG00000046314 |
| 120 | 17451443 | NA | Coro1c | coronin, actin binding protein 1C | 23790 | ENSMUSG00000004530 |
| 121 | 17273280 | NA | Stra13 | stimulated by retinoic acid 13 | 20892 | ENSMUSG00000025144 |
| 122 | 17301342 | NA | Ints9 | integrator complex subunit 9 | 210925 | ENSMUSG00000021975 |
| 123 | 17393357 | NA | Eif6 | eukaryotic translation initiation factor 6 | 16418 | ENSMUSG00000027613 |
| 124 | 17412593 | NA | Srsf12 | serine/arginine-rich splicing factor 12 | 272009 | ENSMUSG00000054679 |
| 125 | 17451816 | NA | Hspb8 | heat shock protein 8 | 80888 | ENSMUSG00000041548 |
| 126 | 17504160 | NA | Polr2c | polymerase (RNA) II (DNA directed) polypeptide C | 20021 | ENSMUSG00000031783 |
| 127 | 17455093 | NA | Zkscan14 | zinc finger with KRAB and SCAN domains 14 | 67235 | ENSMUSG00000029627 |
| 128 | 17488544 | NA | Nfkbib | nuclear factor of kappa light polypeptide gene enhancer in B cells inhibitor, beta | 18036 | ENSMUSG00000030595 |
| 129 | 17328810 | NA | Dgcr14 | DiGeorge syndrome critical region gene 14 | 27886 | ENSMUSG00000003527 |
| 130 | 17229466 | NA | Hsd17b7 | hydroxysteroid (17-beta) dehydrogenase 7 | 15490 | ENSMUSG00000026675 |
| 131 | 17242318 | NA | Gm7138 | predicted gene 7138 | 634517 | ENSMUSG00000095593 |
| 132 | 17463150 | NA | Vamp1 | vesicle-associated membrane protein 1 | 22317 | ENSMUSG00000030337 |
| 133 | 17306861 | NA | Dhrs1 | dehydrogenase/reductase (SDR family) member 1 | 52585 | ENSMUSG00000002332 |
| 134 | 17306864 | NA | Dhrs1 | dehydrogenase/reductase (SDR family) member 1 | 52585 | ENSMUSG00000002332 |
| 135 | 17306860 | NA | Dhrs1 | dehydrogenase/reductase (SDR family) member 1 | 52585 | ENSMUSG00000002332 |
| 136 | 17306856 | NA | Dhrs1 | dehydrogenase/reductase (SDR family) member 1 | 52585 | ENSMUSG00000002332 |
| 137 | 17306865 | NA | Dhrs1 | dehydrogenase/reductase (SDR family) member 1 | 52585 | ENSMUSG00000002332 |
| 138 | 17418571 | NA | Trappc3 | trafficking protein particle complex 3 | 27096 | ENSMUSG00000028847 |
| 139 | 17318942 | NA | Pvalb | parvalbumin | 19293 | ENSMUSG00000005716 |
| 140 | 17419206 | NA | Pef1 | penta-EF hand domain containing 1 | 67898 | ENSMUSG00000028779 |
| 141 | 17535752 | NA | Emd | emerin | 13726 | ENSMUSG00000001964 |
| 142 | 17396024 | NA | Stmn2 | stathmin-like 2 | 20257 | ENSMUSG00000027500 |
| 143 | 17338043 | NA | Yipf3 | Yip1 domain family, member 3 | 28064 | ENSMUSG00000071074 |
| 144 | 17313199 | NA | Adsl | adenylosuccinate lyase | 11564 | ENSMUSG00000022407 |
| 145 | 17418447 | NA | Meaf6 | MYST/Esa1-associated factor 6 | 70088 | ENSMUSG00000028863 |
| 146 | 17232215 | NA | Moxd1 | monooxygenase, DBH-like 1 | 59012 | ENSMUSG00000020000 |
| 147 | 17356202 | NA | Pold4 | polymerase (DNA-directed), delta 4 | 69745 | ENSMUSG00000024854 |
| 148 | 17240123 | NA | Clvs2 | clavesin 2 | 215890 | ENSMUSG00000019785 |
| 149 | 17462975 | NA | Mlf2 | myeloid leukemia factor 2 | 30853 | ENSMUSG00000030120 |
| 150 | 17255719 | NA | Mrpl10 | mitochondrial ribosomal protein L10 | 107732 | ENSMUSG00000001445 |
| 151 | 17257962 | NA | Sstr2 | somatostatin receptor 2 | 20606 | ENSMUSG00000047904 |
| 152 | 17344794 | NA | Znrd1 | zinc ribbon domain containing, 1 | 66136 | ENSMUSG00000036315 |
| 153 | 17232843 | NA | Zbtb24 | zinc finger and BTB domain containing 24 | 268294 | ENSMUSG00000019826 |
| 154 | 17300261 | NA | Oxa1l | oxidase assembly 1-like | 69089 | ENSMUSG00000000959 |
| 155 | 17224540 | NA | Tuba4a | tubulin, alpha 4A | 22145 | ENSMUSG00000026202 |
| 156 | 17456308 | NA | Kcnd2 | potassium voltage-gated channel, Shal-related family, member 2 | 16508 | ENSMUSG00000060882 |
| 157 | 17517723 | NA | Rpp25 | ribonuclease P 25 subunit (human) | 102614 | ENSMUSG00000062309 |
| 158 | 17307134 | NA | Cryl1 | crystallin, lambda 1 | 68631 | ENSMUSG00000021947 |
| 159 | 17521652 | NA | Nicn1 | nicolin 1 | 66257 | ENSMUSG00000032606 |
| 160 | 17369862 | NA | Dpm2 | dolichol-phosphate (beta-D) mannosyltransferase 2 | 13481 | ENSMUSG00000026810 |
| 161 | 17322559 | NA | Hmox2 | heme oxygenase (decycling) 2 | 15369 | ENSMUSG00000004070 |
| 162 | 17537677 | NA | Drp2 | dystrophin related protein 2 | 13497 | ENSMUSG00000000223 |
| 163 | 17375833 | NA | Tmem127 | transmembrane protein 127 | 69470 | ENSMUSG00000034850 |
| 164 | 17538096 | NA | Rnf128 | ring finger protein 128 | 66889 | ENSMUSG00000031438 |
| 165 | 17288160 | NA | Cdk20 | cyclin-dependent kinase 20 | 105278 | ENSMUSG00000021483 |
| 166 | 17263594 | NA | Atpaf2 | ATP synthase mitochondrial F1 complex assembly factor 2 | 246782 | ENSMUSG00000042709 |
| 167 | 17343617 | NA | Rab11b | RAB11B, member RAS oncogene family | 19326 | ENSMUSG00000077450 |
| 168 | 17447099 | NA | Ctbp1 | C-terminal binding protein 1 | 13016 | ENSMUSG00000037373 |
| 169 | 17447089 | NA | Ctbp1 | C-terminal binding protein 1 | 13016 | ENSMUSG00000037373 |
| 170 | 17447100 | NA | Ctbp1 | C-terminal binding protein 1 | 13016 | ENSMUSG00000037373 |
| 171 | 17447098 | NA | Ctbp1 | C-terminal binding protein 1 | 13016 | ENSMUSG00000037373 |
| 172 | 17344336 | NA | Tcf19 | transcription factor 19 | 106795 | ENSMUSG00000050410 |
| 173 | 17433602 | NA | Tprgl | transformation related protein 63 regulated like | 67808 | ENSMUSG00000029030 |
| 174 | 17265082 | NA | Eif5a | eukaryotic translation initiation factor 5A | 276770 | ENSMUSG00000078812 |
| 175 | 17233630 | NA | Psap | prosaposin | 19156 | ENSMUSG00000004207 |
| 176 | 17235368 | NA | Scamp4 | secretory carrier membrane protein 4 | 56214 | ENSMUSG00000078441 |
| 177 | 17369672 | NA | Ppapdc3 | phosphatidic acid phosphatase type 2 domain containing 3 | 227721 | ENSMUSG00000051373 |
| 178 | 17512434 | NA | Tppp3 | tubulin polymerization-promoting protein family member 3 | 67971 | ENSMUSG00000014846 |
| 179 | 17225499 | NA | Hes6 | hairy and enhancer of split 6 (Drosophila) | 55927 | ENSMUSG00000067071 |
| 180 | 17265175 | NA | 0610010K14Rik | RIKEN cDNA 0610010K14 gene | 104457 | ENSMUSG00000020831 |
| 181 | 17377583 | NA | Nsfl1c | NSFL1 (p97) cofactor (p47) | 386649 | ENSMUSG00000027455 |
| 182 | 17404628 | NA | Pex5l | peroxisomal biogenesis factor 5-like | 58869 | ENSMUSG00000027674 |
| 183 | 17517576 | NA | Hmg20a | high mobility group 20A | 66867 | ENSMUSG00000032329 |
| 184 | 17393225 | NA | Pigu | phosphatidylinositol glycan anchor biosynthesis, class U | 228812 | ENSMUSG00000038383 |
| 185 | 17404011 | NA | Hey1 | hairy/enhancer-of-split related with YRPW motif 1 | 15213 | ENSMUSG00000040289 |
| 186 | 17246850 | NA | Zmat5 | zinc finger, matrin type 5 | 67178 | ENSMUSG00000009076 |
| 187 | 17238846 | NA | Syne1 | synaptic nuclear envelope 1 | 64009 | ENSMUSG00000019769 ENSMUSG00000096054 |
| 188 | 17238906 | NA | Syne1 | synaptic nuclear envelope 1 | 64009 | ENSMUSG00000019769 ENSMUSG00000096054 |
| 189 | 17238890 | NA | Syne1 | synaptic nuclear envelope 1 | 64009 | ENSMUSG00000019769 ENSMUSG00000096054 |
| 190 | 17502191 | NA | Mrpl34 | mitochondrial ribosomal protein L34 | 94065 | ENSMUSG00000034880 |
| 191 | 17277370 | NA | Eif2b2 | eukaryotic translation initiation factor 2B, subunit 2 beta | 217715 | ENSMUSG00000004788 |
| 192 | 17316625 | NA | Ubr5 | ubiquitin protein ligase E3 component n-recognin 5 | 70790 | ENSMUSG00000037487 |
| 193 | 17512009 | NA | Csnk2a2 | casein kinase 2, alpha prime polypeptide | 13000 | ENSMUSG00000046707 |
| 194 | 17245709 | NA | Os9 | amplified in osteosarcoma | 216440 | ENSMUSG00000040462 |
| 195 | 17503816 | NA | Irx6 | Iroquois related homeobox 6 (Drosophila) | 64379 | ENSMUSG00000031738 |
| 196 | 17282649 | NA | Rps6kl1 | ribosomal protein S6 kinase-like 1 | 238323 | ENSMUSG00000019235 |
| 197 | 17365493 | NA | Obfc1 | oligonucleotide/oligosaccharide-binding fold containing 1 | 108689 | ENSMUSG00000042694 |
| 198 | 17538790 | NA | Huwe1 | HECT, UBA and WWE domain containing 1 | 59026 | ENSMUSG00000025261 |
| 199 | 17470879 | NA | Tpi1 | triosephosphate isomerase 1 | 21991 | ENSMUSG00000023456 |
| 200 | 17229644 | NA | Tomm40l | translocase of outer mitochondrial membrane 40 homolog-like (yeast) | 641376 | ENSMUSG00000005674 |
| 201 | 17522887 | NA | Golga4 | golgi autoantigen, golgin subfamily a, 4 | 54214 | ENSMUSG00000038708 |
| 202 | 17230408 | NA | Adck3 | aarF domain containing kinase 3 | 67426 | ENSMUSG00000026489 |
| 203 | 17532418 | NA | Ano10 | anoctamin 10 | 102566 | ENSMUSG00000037949 |
| 204 | 17352132 | NA | Txnl4a | thioredoxin-like 4A | 27366 | ENSMUSG00000057130 |
| 205 | 17420582 | NA | Capzb | capping protein (actin filament) muscle Z-line, beta | 12345 | ENSMUSG00000028745 |
| 206 | 17273086 | NA | Nploc4 | nuclear protein localization 4 homolog (S. cerevisiae) | 217365 | ENSMUSG00000039703 |
| 207 | 17275955 | NA | Atl1 | atlastin GTPase 1 | 73991 | ENSMUSG00000021066 |
| 208 | 17431502 | NA | Lypla2 | lysophospholipase 2 | 26394 | ENSMUSG00000028670 |
| 209 | 17500275 | NA | Erlin2 | ER lipid raft associated 2 | 244373 | ENSMUSG00000031483 |
| 210 | 17256565 | NA | Tubg2 | tubulin, gamma 2 | 103768 | ENSMUSG00000045007 |
| 211 | 17463422 | NA | Nrip2 | nuclear receptor interacting protein 2 | 60345 | ENSMUSG00000001520 |
| 212 | 17299542 | NA | Tmem55b | transmembrane protein 55b | 219024 | ENSMUSG00000035953 |
| 213 | 17485194 | NA | Krtap5-4 | keratin associated protein 5-4 | 50775 | ENSMUSG00000045236 |
| 214 | 17253674 | NA | Poldip2 | polymerase (DNA-directed), delta interacting protein 2 | 67811 | ENSMUSG00000001100 |
| 215 | 17213153 | NA | Nif3l1 | Ngg1 interacting factor 3-like 1 (S. pombe) | 65102 | ENSMUSG00000026036 |
| 216 | 17321722 | NA | Tfcp2 | transcription factor CP2 | 21422 | ENSMUSG00000009733 |
| 217 | 17405819 | NA | B3galnt1 | UDP-GalNAc:betaGlcNAc beta 1,3-galactosaminyltransferase, polypeptide 1 | 26879 | ENSMUSG00000043300 |
| 218 | 17498821 | NA | Snapc2 | small nuclear RNA activating complex, polypeptide 2 | 102209 | ENSMUSG00000011837 |
| 219 | 17328451 | NA | Mzt2 | mitotic spindle organizing protein 2 | 72083 | ENSMUSG00000022671 |
| 220 | 17379291 | NA | Ttpal | tocopherol (alpha) transfer protein-like | 76080 | ENSMUSG00000017679 |
| 221 | 17256618 | NA | Vps25 | vacuolar protein sorting 25 (yeast) | 28084 | ENSMUSG00000078656 |
| 222 | 17494637 | NA | Mrpl17 | mitochondrial ribosomal protein L17 | 27397 | ENSMUSG00000030879 |
| 223 | 17517532 | NA | Isl2 | insulin related protein 2 (islet 2) | 104360 | ENSMUSG00000032318 |
| 224 | 17513871 | NA | Chmp1a | charged multivesicular body protein 1A | 234852 | ENSMUSG00000000743 |
| 225 | 17269638 | NA | Rab5c | RAB5C, member RAS oncogene family | 19345 | ENSMUSG00000019173 |
| 226 | 17256549 | NA | Tubg1 | tubulin, gamma 1 | 103733 | ENSMUSG00000035198 |
| 227 | 17214293 | NA | Bcs1l | BCS1-like (yeast) | 66821 | ENSMUSG00000026172 |
| 228 | 17494081 | NA | Rhog | ras homolog gene family, member G | 56212 | ENSMUSG00000073982 |
| 229 | 17497957 | NA | Chid1 | chitinase domain containing 1 | 68038 | ENSMUSG00000025512 |
| 230 | 17532045 | NA | Plcd1 | phospholipase C, delta 1 | 18799 | ENSMUSG00000010660 |
| 231 | 17274448 | NA | Cpsf3 | cleavage and polyadenylation specificity factor 3 | 54451 | ENSMUSG00000054309 |
| 232 | 17332333 | NA | Dscr3 | Down syndrome critical region gene 3 | 13185 | ENSMUSG00000022898 |
| 233 | 17332341 | NA | Dscr3 | Down syndrome critical region gene 3 | 13185 | ENSMUSG00000022898 |
| 234 | 17332343 | NA | Dscr3 | Down syndrome critical region gene 3 | 13185 | ENSMUSG00000022898 |
| 235 | 17332336 | NA | Dscr3 | Down syndrome critical region gene 3 | 13185 | ENSMUSG00000022898 |
| 236 | 17519868 | NA | Htr1b | 5-hydroxytryptamine (serotonin) receptor 1B | 15551 | ENSMUSG00000049511 |
| 237 | 17515170 | NA | Ilf3 | interleukin enhancer binding factor 3 | 16201 | ENSMUSG00000032178 |
| 238 | 17533055 | NA | Timm17b | translocase of inner mitochondrial membrane 17b | 21855 | ENSMUSG00000031158 |
| 239 | 17461968 | NA | Mkrn2 | makorin, ring finger protein, 2 | 67027 | ENSMUSG00000000439 |
| 240 | 17430853 | NA | Med18 | mediator of RNA polymerase II transcription, subunit 18 homolog (yeast) | 67219 | ENSMUSG00000066042 |
| 241 | 17442714 | NA | Bri3bp | Bri3 binding protein | 76809 | ENSMUSG00000037905 |
| 242 | 17521014 | NA | Acad11 | acyl-Coenzyme A dehydrogenase family, member 11 | 102632 | ENSMUSG00000090150 |
| 243 | 17370285 | NA | Mrrf | mitochondrial ribosome recycling factor | 67871 | ENSMUSG00000026887 |
| 244 | 17229948 | NA | Dusp23 | dual specificity phosphatase 23 | 68440 | ENSMUSG00000026544 |
| 245 | 17511296 | NA | Wdr83 | WD repeat domain containing 83 | 67836 | ENSMUSG00000005150 |
| 246 | 17539434 | NA | Ctps2 | cytidine 5'-triphosphate synthase 2 | 55936 | ENSMUSG00000031360 |
| 247 | 17250141 | NA | Zfp39 | zinc finger protein 39 | 22698 | ENSMUSG00000037001 |
| 248 | 17341521 | NA | Thoc6 | THO complex 6 homolog (Drosophila) | 386612 | ENSMUSG00000041319 |
| 249 | 17266322 | NA | Eral1 | Era (G-protein)-like 1 (E. coli) | 57837 | ENSMUSG00000020832 |
| 250 | 17521422 | NA | Hyal2 | hyaluronoglucosaminidase 2 | 15587 | ENSMUSG00000010047 |
| 251 | 17540589 | NA | Klhl13 | kelch-like 13 (Drosophila) | 67455 | ENSMUSG00000036782 |
| 252 | 17376272 | NA | Nop56 | NOP56 ribonucleoprotein homolog (yeast) | 67134 | ENSMUSG00000027405 |
| 253 | 17468195 | NA | Stambp | STAM binding protein | 70527 | ENSMUSG00000006906 |
| 254 | 17342015 | NA | Tbl3 | transducin (beta)-like 3 | 213773 | ENSMUSG00000040688 |
| 255 | 17509721 | NA | Tufm | Tu translation elongation factor, mitochondrial | 233870 | ENSMUSG00000073838 |
| 256 | 17342617 | NA | Arhgdig | Rho GDP dissociation inhibitor (GDI) gamma | 14570 | ENSMUSG00000073433 |
| 257 | 17361463 | NA | Rab1b | RAB1B, member RAS oncogene family | 76308 | ENSMUSG00000024870 |
| 258 | 17279858 | NA | Fkbp1b | FK506 binding protein 1b | 14226 | ENSMUSG00000020635 |
| 259 | 17217580 | NA | Arl8a | ADP-ribosylation factor-like 8A | 68724 | ENSMUSG00000026426 |
| 260 | 17460099 | NA | Vax2 | ventral anterior homeobox containing gene 2 | 24113 | ENSMUSG00000034777 |
| 261 | 17257060 | NA | Nmt1 | N-myristoyltransferase 1 | 18107 | ENSMUSG00000020936 |
| 262 | 17365369 | NA | Cuedc2 | CUE domain containing 2 | 67116 | ENSMUSG00000036748 |
| 263 | 17292107 | NA | Tbc1d7 | TBC1 domain family, member 7 | 67046 | ENSMUSG00000021368 |
| 264 | 17512479 | NA | Acd | adrenocortical dysplasia | 497652 | ENSMUSG00000038000 |
| 265 | 17540050 | NA | Ebp | phenylalkylamine Ca2+ antagonist (emopamil) binding protein | 13595 | ENSMUSG00000031168 |
| 266 | 17232426 | NA | Echdc1 | enoyl Coenzyme A hydratase domain containing 1 | 52665 | ENSMUSG00000019883 |
| 267 | 17212813 | NA | Mstn | myostatin | 17700 | ENSMUSG00000026100 |
| 268 | 17230945 | NA | Smyd2 | SET and MYND domain containing 2 | 226830 | ENSMUSG00000026603 |
| 269 | 17521448 | NA | Hyal3 | hyaluronoglucosaminidase 3 | 109685 | ENSMUSG00000036091 |
| 270 | 17251607 | NA | Trappc1 | trafficking protein particle complex 1 | 245828 | ENSMUSG00000049299 |
| 271 | 17299750 | NA | Tox4 | TOX high mobility group box family member 4 | 268741 | ENSMUSG00000016831 |
| 272 | 17502390 | NA | Rab8a | RAB8A, member RAS oncogene family | 17274 | ENSMUSG00000003037 |
| 273 | 17468113 | NA | Ino80b | INO80 complex subunit B | 70020 | ENSMUSG00000030034 |
| 274 | 17347558 | NA | Cdkl4 | cyclin-dependent kinase-like 4 | 381113 | ENSMUSG00000033966 |
| 275 | 17283939 | NA | Wars | tryptophanyl-tRNA synthetase | 22375 | ENSMUSG00000021266 |
| 276 | 17283941 | NA | Wars | tryptophanyl-tRNA synthetase | 22375 | ENSMUSG00000021266 |
| 277 | 17283930 | NA | Wars | tryptophanyl-tRNA synthetase | 22375 | ENSMUSG00000021266 |
| 278 | 17283938 | NA | Wars | tryptophanyl-tRNA synthetase | 22375 | ENSMUSG00000021266 |
| 279 | 17258457 | NA | Sap30bp | SAP30 binding protein | 57230 | ENSMUSG00000020755 |
| 280 | 17512872 | NA | Calb2 | calbindin 2 | 12308 | ENSMUSG00000003657 |
| 281 | 17510365 | NA | Pgls | 6-phosphogluconolactonase | 66171 | ENSMUSG00000031807 |
| 282 | 17526273 | NA | Trappc4 | trafficking protein particle complex 4 | 60409 | ENSMUSG00000032112 |
| 283 | 17526271 | NA | Trappc4 | trafficking protein particle complex 4 | 60409 | ENSMUSG00000032112 |
| 284 | 17526272 | NA | Trappc4 | trafficking protein particle complex 4 | 60409 | ENSMUSG00000032112 |
| 285 | 17465696 | NA | Slc35b4 | solute carrier family 35, member B4 | 58246 | ENSMUSG00000018999 |
| 286 | 17318877 | NA | Txn2 | thioredoxin 2 | 56551 | ENSMUSG00000005354 |
| 287 | 17453809 | NA | Ap1s1 | adaptor protein complex AP-1, sigma 1 | 11769 | ENSMUSG00000004849 |
| 288 | 17329163 | NA | Camk2n2 | calcium/calmodulin-dependent protein kinase II inhibitor 2 | 73047 | ENSMUSG00000051146 |
| 289 | 17405174 | NA | Cog6 | component of oligomeric golgi complex 6 | 67542 | ENSMUSG00000027742 |
| 290 | 17382914 | NA | Dnlz | DNL-type zinc finger | 52838 | ENSMUSG00000075467 |
| 291 | 17221014 | NA | Cd34 | CD34 antigen | 12490 | ENSMUSG00000016494 |
| 292 | 17241409 | NA | Srgn | serglycin | 19073 | ENSMUSG00000020077 |
| 293 | 17322200 | NA | Aaas | achalasia, adrenocortical insufficiency, alacrimia | 223921 | ENSMUSG00000036678 |
| 294 | 17213990 | NA | Atic | 5-aminoimidazole-4-carboxamide ribonucleotide formyltransferase/IMP cyclohydrolase | 108147 | ENSMUSG00000026192 |
| 295 | 17508691 | NA | Rbpms | RNA binding protein gene with multiple splicing | 19663 | ENSMUSG00000031586 |
| 296 | 17222001 | NA | Prim2 | DNA primase, p58 subunit | 19076 | ENSMUSG00000026134 |
| 297 | 17232649 | NA | Fyn | Fyn proto-oncogene | 14360 | ENSMUSG00000019843 |
| 298 | 17474534 | NA | Opa3 | optic atrophy 3 | 403187 | ENSMUSG00000052214 |
| 299 | 17395844 | NA | Stmn3 | stathmin-like 3 | 20262 | ENSMUSG00000027581 |
| 300 | 17219286 | NA | Dedd | death effector domain-containing | 21945 | ENSMUSG00000013973 |
| 301 | 17241780 | NA | Ube2d1 | ubiquitin-conjugating enzyme E2D 1 | 216080 | ENSMUSG00000019927 |
| 302 | 17359945 | NA | Gbf1 | golgi-specific brefeldin A-resistance factor 1 | 107338 | ENSMUSG00000025224 |
| 303 | 17334495 | NA | Nme3 | NME/NM23 nucleoside diphosphate kinase 3 | 79059 | ENSMUSG00000073435 |
| 304 | 17383588 | NA | Ccbl1 | cysteine conjugate-beta lyase 1 | 70266 | ENSMUSG00000039648 |
| 305 | 17400638 | NA | Pex11b | peroxisomal biogenesis factor 11 beta | 18632 | ENSMUSG00000028102 |
| 306 | 17369952 | NA | Sh2d3c | SH2 domain containing 3C | 27387 | ENSMUSG00000059013 |
| 307 | 17421972 | NA | Errfi1 | ERBB receptor feedback inhibitor 1 | 74155 | ENSMUSG00000028967 |

  
  

| **Database:cellular component      &nbspName:intracellular organelle      &nbspID:GO:0043229** | | | | | | |
| --- | --- | --- | --- | --- | --- | --- |
| C=9371; O=251; E=181.10; R=1.39; rawP=8.20e-13; adjP=6.34e-11 | | | | | | |
| Index | UserID | Value | Gene Symbol | Gene Name | EntrezGene | Ensembl |
| 1 | 17476273 | NA | Zfp382 | zinc finger protein 382 | 233060 | ENSMUSG00000074220 |
| 2 | 17255719 | NA | Mrpl10 | mitochondrial ribosomal protein L10 | 107732 | ENSMUSG00000001445 |
| 3 | 17413221 | NA | Unc13b | unc-13 homolog B (C. elegans) | 22249 | ENSMUSG00000028456 |
| 4 | 17232843 | NA | Zbtb24 | zinc finger and BTB domain containing 24 | 268294 | ENSMUSG00000019826 |
| 5 | 17344794 | NA | Znrd1 | zinc ribbon domain containing, 1 | 66136 | ENSMUSG00000036315 |
| 6 | 17300261 | NA | Oxa1l | oxidase assembly 1-like | 69089 | ENSMUSG00000000959 |
| 7 | 17474547 | NA | Rtn2 | reticulon 2 (Z-band associated protein) | 20167 | ENSMUSG00000030401 |
| 8 | 17512740 | NA | Nob1 | NIN1/RPN12 binding protein 1 homolog (S. cerevisiae) | 67619 | ENSMUSG00000003848 |
| 9 | 17224540 | NA | Tuba4a | tubulin, alpha 4A | 22145 | ENSMUSG00000026202 |
| 10 | 17503910 | NA | Ogfod1 | 2-oxoglutarate and iron-dependent oxygenase domain containing 1 | 270086 | ENSMUSG00000033009 |
| 11 | 17456308 | NA | Kcnd2 | potassium voltage-gated channel, Shal-related family, member 2 | 16508 | ENSMUSG00000060882 |
| 12 | 17368171 | NA | Bmyc | brain expressed myelocytomatosis oncogene | 107771 | ENSMUSG00000049086 |
| 13 | 17350134 | NA | Pou4f3 | POU domain, class 4, transcription factor 3 | 18998 | ENSMUSG00000024497 |
| 14 | 17517723 | NA | Rpp25 | ribonuclease P 25 subunit (human) | 102614 | ENSMUSG00000062309 |
| 15 | 17443181 | NA | Dnajc30 | DnaJ (Hsp40) homolog, subfamily C, member 30 | 66114 | ENSMUSG00000061118 |
| 16 | 17319324 | NA | Dnalc4 | dynein, axonemal, light chain 4 | 54152 | ENSMUSG00000022420 |
| 17 | 17512103 | NA | Got2 | glutamate oxaloacetate transaminase 2, mitochondrial | 14719 | ENSMUSG00000031672 |
| 18 | 17369862 | NA | Dpm2 | dolichol-phosphate (beta-D) mannosyltransferase 2 | 13481 | ENSMUSG00000026810 |
| 19 | 17521652 | NA | Nicn1 | nicolin 1 | 66257 | ENSMUSG00000032606 |
| 20 | 17336829 | NA | Lsm2 | LSM2 homolog, U6 small nuclear RNA associated (S. cerevisiae) | 27756 | ENSMUSG00000007050 |
| 21 | 17288454 | NA | Irx4 | Iroquois related homeobox 4 (Drosophila) | 50916 | ENSMUSG00000021604 |
| 22 | 17467996 | NA | Mrpl19 | mitochondrial ribosomal protein L19 | 56284 | ENSMUSG00000030045 |
| 23 | 17322559 | NA | Hmox2 | heme oxygenase (decycling) 2 | 15369 | ENSMUSG00000004070 |
| 24 | 17537677 | NA | Drp2 | dystrophin related protein 2 | 13497 | ENSMUSG00000000223 |
| 25 | 17306758 | NA | Tm9sf1 | transmembrane 9 superfamily member 1 | 74140 | ENSMUSG00000002320 |
| 26 | 17504712 | NA | Exoc3l | exocyst complex component 3-like | 277978 | ENSMUSG00000043251 |
| 27 | 17321467 | NA | Tuba1b | tubulin, alpha 1B | 22143 | ENSMUSG00000023004 |
| 28 | 17288160 | NA | Cdk20 | cyclin-dependent kinase 20 | 105278 | ENSMUSG00000021483 |
| 29 | 17538096 | NA | Rnf128 | ring finger protein 128 | 66889 | ENSMUSG00000031438 |
| 30 | 17541597 | NA | Frmd7 | FERM domain containing 7 | 385354 | ENSMUSG00000036131 |
| 31 | 17263594 | NA | Atpaf2 | ATP synthase mitochondrial F1 complex assembly factor 2 | 246782 | ENSMUSG00000042709 |
| 32 | 17447099 | NA | Ctbp1 | C-terminal binding protein 1 | 13016 | ENSMUSG00000037373 |
| 33 | 17447089 | NA | Ctbp1 | C-terminal binding protein 1 | 13016 | ENSMUSG00000037373 |
| 34 | 17447100 | NA | Ctbp1 | C-terminal binding protein 1 | 13016 | ENSMUSG00000037373 |
| 35 | 17447098 | NA | Ctbp1 | C-terminal binding protein 1 | 13016 | ENSMUSG00000037373 |
| 36 | 17343617 | NA | Rab11b | RAB11B, member RAS oncogene family | 19326 | ENSMUSG00000077450 |
| 37 | 17379187 | NA | Ift52 | intraflagellar transport 52 | 245866 | ENSMUSG00000017858 |
| 38 | 17246284 | NA | Suox | sulfite oxidase | 211389 | ENSMUSG00000049858 |
| 39 | 17287579 | NA | Zfp346 | zinc finger protein 346 | 26919 | ENSMUSG00000021481 |
| 40 | 17224577 | NA | Resp18 | regulated endocrine-specific protein 18 | 19711 | ENSMUSG00000033061 |
| 41 | 17265082 | NA | Eif5a | eukaryotic translation initiation factor 5A | 276770 | ENSMUSG00000078812 |
| 42 | 17433602 | NA | Tprgl | transformation related protein 63 regulated like | 67808 | ENSMUSG00000029030 |
| 43 | 17344336 | NA | Tcf19 | transcription factor 19 | 106795 | ENSMUSG00000050410 |
| 44 | 17297750 | NA | Ppif | peptidylprolyl isomerase F (cyclophilin F) | 105675 | ENSMUSG00000021868 |
| 45 | 17233630 | NA | Psap | prosaposin | 19156 | ENSMUSG00000004207 |
| 46 | 17520177 | NA | Mthfs | 5, 10-methenyltetrahydrofolate synthetase | 107885 | ENSMUSG00000066442 |
| 47 | 17235368 | NA | Scamp4 | secretory carrier membrane protein 4 | 56214 | ENSMUSG00000078441 |
| 48 | 17369672 | NA | Ppapdc3 | phosphatidic acid phosphatase type 2 domain containing 3 | 227721 | ENSMUSG00000051373 |
| 49 | 17480102 | NA | Sytl2 | synaptotagmin-like 2 | 83671 | ENSMUSG00000030616 |
| 50 | 17521143 | NA | Wdr82 | WD repeat domain containing 82 | 77305 | ENSMUSG00000020257 |
| 51 | 17225499 | NA | Hes6 | hairy and enhancer of split 6 (Drosophila) | 55927 | ENSMUSG00000067071 |
| 52 | 17512434 | NA | Tppp3 | tubulin polymerization-promoting protein family member 3 | 67971 | ENSMUSG00000014846 |
| 53 | 17377583 | NA | Nsfl1c | NSFL1 (p97) cofactor (p47) | 386649 | ENSMUSG00000027455 |
| 54 | 17265175 | NA | 0610010K14Rik | RIKEN cDNA 0610010K14 gene | 104457 | ENSMUSG00000020831 |
| 55 | 17312944 | NA | Polr2f | polymerase (RNA) II (DNA directed) polypeptide F | 69833 | ENSMUSG00000033020 |
| 56 | 17312939 | NA | Polr2f | polymerase (RNA) II (DNA directed) polypeptide F | 69833 | ENSMUSG00000033020 |
| 57 | 17312941 | NA | Polr2f | polymerase (RNA) II (DNA directed) polypeptide F | 69833 | ENSMUSG00000033020 |
| 58 | 17329151 | NA | Alg3 | asparagine-linked glycosylation 3 (alpha-1,3-mannosyltransferase) | 208624 | ENSMUSG00000033809 |
| 59 | 17517576 | NA | Hmg20a | high mobility group 20A | 66867 | ENSMUSG00000032329 |
| 60 | 17393225 | NA | Pigu | phosphatidylinositol glycan anchor biosynthesis, class U | 228812 | ENSMUSG00000038383 |
| 61 | 17500391 | NA | Rnf122 | ring finger protein 122 | 68867 | ENSMUSG00000039328 |
| 62 | 17404011 | NA | Hey1 | hairy/enhancer-of-split related with YRPW motif 1 | 15213 | ENSMUSG00000040289 |
| 63 | 17400222 | NA | Vps72 | vacuolar protein sorting 72 (yeast) | 21427 | ENSMUSG00000008958 |
| 64 | 17499396 | NA | Fbxo25 | F-box protein 25 | 66822 | ENSMUSG00000038365 |
| 65 | 17246850 | NA | Zmat5 | zinc finger, matrin type 5 | 67178 | ENSMUSG00000009076 |
| 66 | 17324664 | NA | Dlg1 | discs, large homolog 1 (Drosophila) | 13383 | ENSMUSG00000022770 |
| 67 | 17396369 | NA | Nceh1 | arylacetamide deacetylase-like 1 | 320024 | ENSMUSG00000027698 |
| 68 | 17314556 | NA | Slc48a1 | solute carrier family 48 (heme transporter), member 1 | 67739 | ENSMUSG00000081534 |
| 69 | 17238846 | NA | Syne1 | synaptic nuclear envelope 1 | 64009 | ENSMUSG00000019769 ENSMUSG00000096054 |
| 70 | 17238906 | NA | Syne1 | synaptic nuclear envelope 1 | 64009 | ENSMUSG00000019769 ENSMUSG00000096054 |
| 71 | 17238890 | NA | Syne1 | synaptic nuclear envelope 1 | 64009 | ENSMUSG00000019769 ENSMUSG00000096054 |
| 72 | 17246209 | NA | Rpl41 | ribosomal protein L41 | 67945 | ENSMUSG00000093674 |
| 73 | 17502191 | NA | Mrpl34 | mitochondrial ribosomal protein L34 | 94065 | ENSMUSG00000034880 |
| 74 | 17529231 | NA | Phip | pleckstrin homology domain interacting protein | 83946 | ENSMUSG00000032253 |
| 75 | 17316625 | NA | Ubr5 | ubiquitin protein ligase E3 component n-recognin 5 | 70790 | ENSMUSG00000037487 |
| 76 | 17483220 | NA | Cdipt | CDP-diacylglycerol--inositol 3-phosphatidyltransferase (phosphatidylinositol synthase) | 52858 | ENSMUSG00000030682 |
| 77 | 17361975 | NA | Snx15 | sorting nexin 15 | 69024 | ENSMUSG00000024787 |
| 78 | 17245709 | NA | Os9 | amplified in osteosarcoma | 216440 | ENSMUSG00000040462 |
| 79 | 17512009 | NA | Csnk2a2 | casein kinase 2, alpha prime polypeptide | 13000 | ENSMUSG00000046707 |
| 80 | 17424319 | NA | Sigmar1 | sigma non-opioid intracellular receptor 1 | 18391 | ENSMUSG00000036078 |
| 81 | 17503816 | NA | Irx6 | Iroquois related homeobox 6 (Drosophila) | 64379 | ENSMUSG00000031738 |
| 82 | 17282649 | NA | Rps6kl1 | ribosomal protein S6 kinase-like 1 | 238323 | ENSMUSG00000019235 |
| 83 | 17260221 | NA | Pold2 | polymerase (DNA directed), delta 2, regulatory subunit | 18972 | ENSMUSG00000020471 |
| 84 | 17365493 | NA | Obfc1 | oligonucleotide/oligosaccharide-binding fold containing 1 | 108689 | ENSMUSG00000042694 |
| 85 | 17538790 | NA | Huwe1 | HECT, UBA and WWE domain containing 1 | 59026 | ENSMUSG00000025261 |
| 86 | 17446580 | NA | Shh | sonic hedgehog | 20423 | ENSMUSG00000002633 |
| 87 | 17229644 | NA | Tomm40l | translocase of outer mitochondrial membrane 40 homolog-like (yeast) | 641376 | ENSMUSG00000005674 |
| 88 | 17470879 | NA | Tpi1 | triosephosphate isomerase 1 | 21991 | ENSMUSG00000023456 |
| 89 | 17281971 | NA | Sgpp1 | sphingosine-1-phosphate phosphatase 1 | 81535 | ENSMUSG00000021054 |
| 90 | 17522887 | NA | Golga4 | golgi autoantigen, golgin subfamily a, 4 | 54214 | ENSMUSG00000038708 |
| 91 | 17230408 | NA | Adck3 | aarF domain containing kinase 3 | 67426 | ENSMUSG00000026489 |
| 92 | 17420582 | NA | Capzb | capping protein (actin filament) muscle Z-line, beta | 12345 | ENSMUSG00000028745 |
| 93 | 17352132 | NA | Txnl4a | thioredoxin-like 4A | 27366 | ENSMUSG00000057130 |
| 94 | 17273086 | NA | Nploc4 | nuclear protein localization 4 homolog (S. cerevisiae) | 217365 | ENSMUSG00000039703 |
| 95 | 17238549 | NA | Wibg | within bgcn homolog (Drosophila) | 78428 | ENSMUSG00000064030 |
| 96 | 17212355 | NA | Nck2 | non-catalytic region of tyrosine kinase adaptor protein 2 | 17974 | ENSMUSG00000066877 |
| 97 | 17275955 | NA | Atl1 | atlastin GTPase 1 | 73991 | ENSMUSG00000021066 |
| 98 | 17307695 | NA | Msra | methionine sulfoxide reductase A | 110265 | ENSMUSG00000054733 |
| 99 | 17303496 | NA | Fezf2 | Fez family zinc finger 2 | 54713 | ENSMUSG00000021743 |
| 100 | 17256565 | NA | Tubg2 | tubulin, gamma 2 | 103768 | ENSMUSG00000045007 |
| 101 | 17500275 | NA | Erlin2 | ER lipid raft associated 2 | 244373 | ENSMUSG00000031483 |
| 102 | 17345519 | NA | Rrp36 | ribosomal RNA processing 36 homolog (S. cerevisiae) | 224823 | ENSMUSG00000023971 |
| 103 | 17463422 | NA | Nrip2 | nuclear receptor interacting protein 2 | 60345 | ENSMUSG00000001520 |
| 104 | 17211335 | NA | Tfap2d | transcription factor AP-2, delta | 226896 | ENSMUSG00000042596 |
| 105 | 17299542 | NA | Tmem55b | transmembrane protein 55b | 219024 | ENSMUSG00000035953 |
| 106 | 17506631 | NA | Tubb3 | tubulin, beta 3 class III | 22152 | ENSMUSG00000062380 |
| 107 | 17253674 | NA | Poldip2 | polymerase (DNA-directed), delta interacting protein 2 | 67811 | ENSMUSG00000001100 |
| 108 | 17485194 | NA | Krtap5-4 | keratin associated protein 5-4 | 50775 | ENSMUSG00000045236 |
| 109 | 17321722 | NA | Tfcp2 | transcription factor CP2 | 21422 | ENSMUSG00000009733 |
| 110 | 17213153 | NA | Nif3l1 | Ngg1 interacting factor 3-like 1 (S. pombe) | 65102 | ENSMUSG00000026036 |
| 111 | 17235584 | NA | Dapk3 | death-associated protein kinase 3 | 13144 | ENSMUSG00000034974 |
| 112 | 17338670 | NA | Fsd1 | fibronectin type 3 and SPRY domain-containing protein | 240121 | ENSMUSG00000011589 |
| 113 | 17405819 | NA | B3galnt1 | UDP-GalNAc:betaGlcNAc beta 1,3-galactosaminyltransferase, polypeptide 1 | 26879 | ENSMUSG00000043300 |
| 114 | 17300411 | NA | Thtpa | thiamine triphosphatase | 105663 | ENSMUSG00000045691 |
| 115 | 17498821 | NA | Snapc2 | small nuclear RNA activating complex, polypeptide 2 | 102209 | ENSMUSG00000011837 |
| 116 | 17460879 | NA | Hdac11 | histone deacetylase 11 | 232232 | ENSMUSG00000034245 |
| 117 | 17328451 | NA | Mzt2 | mitotic spindle organizing protein 2 | 72083 | ENSMUSG00000022671 |
| 118 | 17256618 | NA | Vps25 | vacuolar protein sorting 25 (yeast) | 28084 | ENSMUSG00000078656 |
| 119 | 17444100 | NA | Chst12 | carbohydrate sulfotransferase 12 | 59031 | ENSMUSG00000036599 |
| 120 | 17517532 | NA | Isl2 | insulin related protein 2 (islet 2) | 104360 | ENSMUSG00000032318 |
| 121 | 17494637 | NA | Mrpl17 | mitochondrial ribosomal protein L17 | 27397 | ENSMUSG00000030879 |
| 122 | 17513871 | NA | Chmp1a | charged multivesicular body protein 1A | 234852 | ENSMUSG00000000743 |
| 123 | 17217666 | NA | Tmem9 | transmembrane protein 9 | 66241 | ENSMUSG00000026411 |
| 124 | 17484068 | NA | Lhpp | phospholysine phosphohistidine inorganic pyrophosphate phosphatase | 76429 | ENSMUSG00000030946 |
| 125 | 17497366 | NA | Ebf3 | early B cell factor 3 | 13593 | ENSMUSG00000010476 |
| 126 | 17288616 | NA | Tppp | tubulin polymerization promoting protein | 72948 | ENSMUSG00000021573 |
| 127 | 17452552 | NA | Rhof | ras homolog gene family, member f | 23912 | ENSMUSG00000029449 |
| 128 | 17407764 | NA | Prune | prune homolog (Drosophila) | 229589 | ENSMUSG00000015711 |
| 129 | 17524523 | NA | Eif3g | eukaryotic translation initiation factor 3, subunit G | 53356 | ENSMUSG00000070319 |
| 130 | 17269638 | NA | Rab5c | RAB5C, member RAS oncogene family | 19345 | ENSMUSG00000019173 |
| 131 | 17268995 | NA | Krt222 | keratin 222 | 268481 | ENSMUSG00000035849 |
| 132 | 17505367 | NA | Txnl4b | thioredoxin-like 4B | 234723 | ENSMUSG00000031723 |
| 133 | 17256549 | NA | Tubg1 | tubulin, gamma 1 | 103733 | ENSMUSG00000035198 |
| 134 | 17214293 | NA | Bcs1l | BCS1-like (yeast) | 66821 | ENSMUSG00000026172 |
| 135 | 17540982 | NA | Sept6 | septin 6 | 56526 | ENSMUSG00000050379 |
| 136 | 17497957 | NA | Chid1 | chitinase domain containing 1 | 68038 | ENSMUSG00000025512 |
| 137 | 17235268 | NA | Ndufs7 | NADH dehydrogenase (ubiquinone) Fe-S protein 7 | 75406 | ENSMUSG00000020153 |
| 138 | 17532045 | NA | Plcd1 | phospholipase C, delta 1 | 18799 | ENSMUSG00000010660 |
| 139 | 17447726 | NA | Hs3st1 | heparan sulfate (glucosamine) 3-O-sulfotransferase 1 | 15476 | ENSMUSG00000051022 |
| 140 | 17274448 | NA | Cpsf3 | cleavage and polyadenylation specificity factor 3 | 54451 | ENSMUSG00000054309 |
| 141 | 17454416 | NA | Zfand2a | zinc finger, AN1-type domain 2A | 100494 | ENSMUSG00000053581 |
| 142 | 17332333 | NA | Dscr3 | Down syndrome critical region gene 3 | 13185 | ENSMUSG00000022898 |
| 143 | 17332341 | NA | Dscr3 | Down syndrome critical region gene 3 | 13185 | ENSMUSG00000022898 |
| 144 | 17332343 | NA | Dscr3 | Down syndrome critical region gene 3 | 13185 | ENSMUSG00000022898 |
| 145 | 17332336 | NA | Dscr3 | Down syndrome critical region gene 3 | 13185 | ENSMUSG00000022898 |
| 146 | 17536264 | NA | Pcyt1b | phosphate cytidylyltransferase 1, choline, beta isoform | 236899 | ENSMUSG00000035246 |
| 147 | 17515170 | NA | Ilf3 | interleukin enhancer binding factor 3 | 16201 | ENSMUSG00000032178 |
| 148 | 17533055 | NA | Timm17b | translocase of inner mitochondrial membrane 17b | 21855 | ENSMUSG00000031158 |
| 149 | 17357213 | NA | Zbtb3 | zinc finger and BTB domain containing 3 | 75291 | ENSMUSG00000071661 |
| 150 | 17453106 | NA | Zfp11 | zinc finger protein 11 | 22648 | ENSMUSG00000051034 |
| 151 | 17430853 | NA | Med18 | mediator of RNA polymerase II transcription, subunit 18 homolog (yeast) | 67219 | ENSMUSG00000066042 |
| 152 | 17315558 | NA | Copz1 | coatomer protein complex, subunit zeta 1 | 56447 | ENSMUSG00000060992 |
| 153 | 17442714 | NA | Bri3bp | Bri3 binding protein | 76809 | ENSMUSG00000037905 |
| 154 | 17521014 | NA | Acad11 | acyl-Coenzyme A dehydrogenase family, member 11 | 102632 | ENSMUSG00000090150 |
| 155 | 17370285 | NA | Mrrf | mitochondrial ribosome recycling factor | 67871 | ENSMUSG00000026887 |
| 156 | 17361988 | NA | Arl2 | ADP-ribosylation factor-like 2 | 56327 | ENSMUSG00000024944 |
| 157 | 17229948 | NA | Dusp23 | dual specificity phosphatase 23 | 68440 | ENSMUSG00000026544 |
| 158 | 17502583 | NA | Mcm5 | minichromosome maintenance deficient 5, cell division cycle 46 (S. cerevisiae) | 17218 | ENSMUSG00000005410 |
| 159 | 17511296 | NA | Wdr83 | WD repeat domain containing 83 | 67836 | ENSMUSG00000005150 |
| 160 | 17539434 | NA | Ctps2 | cytidine 5'-triphosphate synthase 2 | 55936 | ENSMUSG00000031360 |
| 161 | 17341521 | NA | Thoc6 | THO complex 6 homolog (Drosophila) | 386612 | ENSMUSG00000041319 |
| 162 | 17250141 | NA | Zfp39 | zinc finger protein 39 | 22698 | ENSMUSG00000037001 |
| 163 | 17485815 | NA | Syt5 | synaptotagmin V | 53420 | ENSMUSG00000004961 |
| 164 | 17519718 | NA | Mto1 | mitochondrial translation optimization 1 homolog (S. cerevisiae) | 68291 | ENSMUSG00000032342 |
| 165 | 17266322 | NA | Eral1 | Era (G-protein)-like 1 (E. coli) | 57837 | ENSMUSG00000020832 |
| 166 | 17488134 | NA | Rab4b | RAB4B, member RAS oncogene family | 19342 | ENSMUSG00000053291 |
| 167 | 17521422 | NA | Hyal2 | hyaluronoglucosaminidase 2 | 15587 | ENSMUSG00000010047 |
| 168 | 17344453 | NA | Ppp1r18 | protein phosphatase 1, regulatory subunit 18 | 76448 | ENSMUSG00000034595 |
| 169 | 17317208 | NA | Derl1 | Der1-like domain family, member 1 | 67819 | ENSMUSG00000022365 |
| 170 | 17501544 | NA | Npy1r | neuropeptide Y receptor Y1 | 18166 | ENSMUSG00000036437 |
| 171 | 17376272 | NA | Nop56 | NOP56 ribonucleoprotein homolog (yeast) | 67134 | ENSMUSG00000027405 |
| 172 | 17468195 | NA | Stambp | STAM binding protein | 70527 | ENSMUSG00000006906 |
| 173 | 17408684 | NA | Dclre1b | DNA cross-link repair 1B, PSO2 homolog (S. cerevisiae) | 140917 | ENSMUSG00000027845 |
| 174 | 17443047 | NA | Caln1 | calneuron 1 | 140904 | ENSMUSG00000060371 |
| 175 | 17436077 | NA | Nrbp1 | nuclear receptor binding protein 1 | 192292 | ENSMUSG00000029148 |
| 176 | 17342015 | NA | Tbl3 | transducin (beta)-like 3 | 213773 | ENSMUSG00000040688 |
| 177 | 17543988 | NA | Taf9b | TAF9B RNA polymerase II, TATA box binding protein (TBP)-associated factor | 407786 | ENSMUSG00000047242 |
| 178 | 17509721 | NA | Tufm | Tu translation elongation factor, mitochondrial | 233870 | ENSMUSG00000073838 |
| 179 | 17459676 | NA | Retsat | retinol saturase (all trans retinol 13,14 reductase) | 67442 | ENSMUSG00000056666 |
| 180 | 17337269 | NA | Nrm | nurim (nuclear envelope membrane protein) | 106582 | ENSMUSG00000059791 |
| 181 | 17361463 | NA | Rab1b | RAB1B, member RAS oncogene family | 76308 | ENSMUSG00000024870 |
| 182 | 17279858 | NA | Fkbp1b | FK506 binding protein 1b | 14226 | ENSMUSG00000020635 |
| 183 | 17488463 | NA | Med29 | mediator complex subunit 29 | 67224 | ENSMUSG00000003444 |
| 184 | 17391270 | NA | Kcnip3 | Kv channel interacting protein 3, calsenilin | 56461 | ENSMUSG00000079056 |
| 185 | 17324576 | NA | Hrasls | HRAS-like suppressor | 27281 | ENSMUSG00000022525 |
| 186 | 17217580 | NA | Arl8a | ADP-ribosylation factor-like 8A | 68724 | ENSMUSG00000026426 |
| 187 | 17460099 | NA | Vax2 | ventral anterior homeobox containing gene 2 | 24113 | ENSMUSG00000034777 |
| 188 | 17348933 | NA | Mapre2 | microtubule-associated protein, RP/EB family, member 2 | 212307 | ENSMUSG00000024277 |
| 189 | 17257060 | NA | Nmt1 | N-myristoyltransferase 1 | 18107 | ENSMUSG00000020936 |
| 190 | 17266489 | NA | Tmem97 | transmembrane protein 97 | 69071 | ENSMUSG00000037278 |
| 191 | 17365369 | NA | Cuedc2 | CUE domain containing 2 | 67116 | ENSMUSG00000036748 |
| 192 | 17299353 | NA | Ktn1 | kinectin 1 | 16709 | ENSMUSG00000021843 |
| 193 | 17292107 | NA | Tbc1d7 | TBC1 domain family, member 7 | 67046 | ENSMUSG00000021368 |
| 194 | 17506854 | NA | Tsnax | translin-associated factor X | 53424 | ENSMUSG00000056820 |
| 195 | 17512479 | NA | Acd | adrenocortical dysplasia | 497652 | ENSMUSG00000038000 |
| 196 | 17288716 | NA | Glrx | glutaredoxin | 93692 | ENSMUSG00000021591 |
| 197 | 17540050 | NA | Ebp | phenylalkylamine Ca2+ antagonist (emopamil) binding protein | 13595 | ENSMUSG00000031168 |
| 198 | 17429495 | NA | Nfyc | nuclear transcription factor-Y gamma | 18046 | ENSMUSG00000032897 |
| 199 | 17361855 | NA | Pola2 | polymerase (DNA directed), alpha 2 | 18969 | ENSMUSG00000024833 |
| 200 | 17512463 | NA | Atp6v0d1 | ATPase, H+ transporting, lysosomal V0 subunit D1 | 11972 | ENSMUSG00000013160 |
| 201 | 17512466 | NA | Atp6v0d1 | ATPase, H+ transporting, lysosomal V0 subunit D1 | 11972 | ENSMUSG00000013160 |
| 202 | 17230945 | NA | Smyd2 | SET and MYND domain containing 2 | 226830 | ENSMUSG00000026603 |
| 203 | 17313008 | NA | Cby1 | chibby homolog 1 (Drosophila) | 73739 | ENSMUSG00000022428 |
| 204 | 17251607 | NA | Trappc1 | trafficking protein particle complex 1 | 245828 | ENSMUSG00000049299 |
| 205 | 17521448 | NA | Hyal3 | hyaluronoglucosaminidase 3 | 109685 | ENSMUSG00000036091 |
| 206 | 17535434 | NA | Nsdhl | NAD(P) dependent steroid dehydrogenase-like | 18194 | ENSMUSG00000031349 |
| 207 | 17299750 | NA | Tox4 | TOX high mobility group box family member 4 | 268741 | ENSMUSG00000016831 |
| 208 | 17468113 | NA | Ino80b | INO80 complex subunit B | 70020 | ENSMUSG00000030034 |
| 209 | 17502390 | NA | Rab8a | RAB8A, member RAS oncogene family | 17274 | ENSMUSG00000003037 |
| 210 | 17503023 | NA | Asf1b | ASF1 anti-silencing function 1 homolog B (S. cerevisiae) | 66929 | ENSMUSG00000005470 |
| 211 | 17258457 | NA | Sap30bp | SAP30 binding protein | 57230 | ENSMUSG00000020755 |
| 212 | 17328829 | NA | Slc25a1 | solute carrier family 25 (mitochondrial carrier, citrate transporter), member 1 | 13358 | ENSMUSG00000003528 |
| 213 | 17454256 | NA | Taf6 | TAF6 RNA polymerase II, TATA box binding protein (TBP)-associated factor | 21343 | ENSMUSG00000036980 |
| 214 | 17432967 | NA | Ubiad1 | UbiA prenyltransferase domain containing 1 | 71707 | ENSMUSG00000047719 |
| 215 | 17526273 | NA | Trappc4 | trafficking protein particle complex 4 | 60409 | ENSMUSG00000032112 |
| 216 | 17526271 | NA | Trappc4 | trafficking protein particle complex 4 | 60409 | ENSMUSG00000032112 |
| 217 | 17526272 | NA | Trappc4 | trafficking protein particle complex 4 | 60409 | ENSMUSG00000032112 |
| 218 | 17465696 | NA | Slc35b4 | solute carrier family 35, member B4 | 58246 | ENSMUSG00000018999 |
| 219 | 17231118 | NA | Rcor3 | REST corepressor 3 | 214742 | ENSMUSG00000037395 |
| 220 | 17379554 | NA | Zswim1 | zinc finger, SWIM domain containing 1 | 71971 | ENSMUSG00000017764 |
| 221 | 17298407 | NA | Bap1 | Brca1 associated protein 1 | 104416 | ENSMUSG00000021901 |
| 222 | 17318877 | NA | Txn2 | thioredoxin 2 | 56551 | ENSMUSG00000005354 |
| 223 | 17453809 | NA | Ap1s1 | adaptor protein complex AP-1, sigma 1 | 11769 | ENSMUSG00000004849 |
| 224 | 17329163 | NA | Camk2n2 | calcium/calmodulin-dependent protein kinase II inhibitor 2 | 73047 | ENSMUSG00000051146 |
| 225 | 17405174 | NA | Cog6 | component of oligomeric golgi complex 6 | 67542 | ENSMUSG00000027742 |
| 226 | 17382914 | NA | Dnlz | DNL-type zinc finger | 52838 | ENSMUSG00000075467 |
| 227 | 17451443 | NA | Coro1c | coronin, actin binding protein 1C | 23790 | ENSMUSG00000004530 |
| 228 | 17273280 | NA | Stra13 | stimulated by retinoic acid 13 | 20892 | ENSMUSG00000025144 |
| 229 | 17301342 | NA | Ints9 | integrator complex subunit 9 | 210925 | ENSMUSG00000021975 |
| 230 | 17241409 | NA | Srgn | serglycin | 19073 | ENSMUSG00000020077 |
| 231 | 17322200 | NA | Aaas | achalasia, adrenocortical insufficiency, alacrimia | 223921 | ENSMUSG00000036678 |
| 232 | 17393357 | NA | Eif6 | eukaryotic translation initiation factor 6 | 16418 | ENSMUSG00000027613 |
| 233 | 17412593 | NA | Srsf12 | serine/arginine-rich splicing factor 12 | 272009 | ENSMUSG00000054679 |
| 234 | 17451816 | NA | Hspb8 | heat shock protein 8 | 80888 | ENSMUSG00000041548 |
| 235 | 17213990 | NA | Atic | 5-aminoimidazole-4-carboxamide ribonucleotide formyltransferase/IMP cyclohydrolase | 108147 | ENSMUSG00000026192 |
| 236 | 17504160 | NA | Polr2c | polymerase (RNA) II (DNA directed) polypeptide C | 20021 | ENSMUSG00000031783 |
| 237 | 17455093 | NA | Zkscan14 | zinc finger with KRAB and SCAN domains 14 | 67235 | ENSMUSG00000029627 |
| 238 | 17488544 | NA | Nfkbib | nuclear factor of kappa light polypeptide gene enhancer in B cells inhibitor, beta | 18036 | ENSMUSG00000030595 |
| 239 | 17508691 | NA | Rbpms | RNA binding protein gene with multiple splicing | 19663 | ENSMUSG00000031586 |
| 240 | 17222001 | NA | Prim2 | DNA primase, p58 subunit | 19076 | ENSMUSG00000026134 |
| 241 | 17232649 | NA | Fyn | Fyn proto-oncogene | 14360 | ENSMUSG00000019843 |
| 242 | 17328810 | NA | Dgcr14 | DiGeorge syndrome critical region gene 14 | 27886 | ENSMUSG00000003527 |
| 243 | 17474534 | NA | Opa3 | optic atrophy 3 | 403187 | ENSMUSG00000052214 |
| 244 | 17395844 | NA | Stmn3 | stathmin-like 3 | 20262 | ENSMUSG00000027581 |
| 245 | 17229466 | NA | Hsd17b7 | hydroxysteroid (17-beta) dehydrogenase 7 | 15490 | ENSMUSG00000026675 |
| 246 | 17242318 | NA | Gm7138 | predicted gene 7138 | 634517 | ENSMUSG00000095593 |
| 247 | 17219286 | NA | Dedd | death effector domain-containing | 21945 | ENSMUSG00000013973 |
| 248 | 17463150 | NA | Vamp1 | vesicle-associated membrane protein 1 | 22317 | ENSMUSG00000030337 |
| 249 | 17306861 | NA | Dhrs1 | dehydrogenase/reductase (SDR family) member 1 | 52585 | ENSMUSG00000002332 |
| 250 | 17306864 | NA | Dhrs1 | dehydrogenase/reductase (SDR family) member 1 | 52585 | ENSMUSG00000002332 |
| 251 | 17306860 | NA | Dhrs1 | dehydrogenase/reductase (SDR family) member 1 | 52585 | ENSMUSG00000002332 |
| 252 | 17306856 | NA | Dhrs1 | dehydrogenase/reductase (SDR family) member 1 | 52585 | ENSMUSG00000002332 |
| 253 | 17306865 | NA | Dhrs1 | dehydrogenase/reductase (SDR family) member 1 | 52585 | ENSMUSG00000002332 |
| 254 | 17418571 | NA | Trappc3 | trafficking protein particle complex 3 | 27096 | ENSMUSG00000028847 |
| 255 | 17359945 | NA | Gbf1 | golgi-specific brefeldin A-resistance factor 1 | 107338 | ENSMUSG00000025224 |
| 256 | 17334495 | NA | Nme3 | NME/NM23 nucleoside diphosphate kinase 3 | 79059 | ENSMUSG00000073435 |
| 257 | 17338043 | NA | Yipf3 | Yip1 domain family, member 3 | 28064 | ENSMUSG00000071074 |
| 258 | 17396024 | NA | Stmn2 | stathmin-like 2 | 20257 | ENSMUSG00000027500 |
| 259 | 17535752 | NA | Emd | emerin | 13726 | ENSMUSG00000001964 |
| 260 | 17383588 | NA | Ccbl1 | cysteine conjugate-beta lyase 1 | 70266 | ENSMUSG00000039648 |
| 261 | 17313199 | NA | Adsl | adenylosuccinate lyase | 11564 | ENSMUSG00000022407 |
| 262 | 17232215 | NA | Moxd1 | monooxygenase, DBH-like 1 | 59012 | ENSMUSG00000020000 |
| 263 | 17418447 | NA | Meaf6 | MYST/Esa1-associated factor 6 | 70088 | ENSMUSG00000028863 |
| 264 | 17356202 | NA | Pold4 | polymerase (DNA-directed), delta 4 | 69745 | ENSMUSG00000024854 |
| 265 | 17400638 | NA | Pex11b | peroxisomal biogenesis factor 11 beta | 18632 | ENSMUSG00000028102 |
| 266 | 17240123 | NA | Clvs2 | clavesin 2 | 215890 | ENSMUSG00000019785 |
| 267 | 17421972 | NA | Errfi1 | ERBB receptor feedback inhibitor 1 | 74155 | ENSMUSG00000028967 |
| 268 | 17462975 | NA | Mlf2 | myeloid leukemia factor 2 | 30853 | ENSMUSG00000030120 |

  
  

| **Database:cellular component      &nbspName:organelle      &nbspID:GO:0043226** | | | | | | |
| --- | --- | --- | --- | --- | --- | --- |
| C=9394; O=251; E=181.54; R=1.38; rawP=1.13e-12; adjP=6.55e-11 | | | | | | |
| Index | UserID | Value | Gene Symbol | Gene Name | EntrezGene | Ensembl |
| 1 | 17476273 | NA | Zfp382 | zinc finger protein 382 | 233060 | ENSMUSG00000074220 |
| 2 | 17255719 | NA | Mrpl10 | mitochondrial ribosomal protein L10 | 107732 | ENSMUSG00000001445 |
| 3 | 17413221 | NA | Unc13b | unc-13 homolog B (C. elegans) | 22249 | ENSMUSG00000028456 |
| 4 | 17232843 | NA | Zbtb24 | zinc finger and BTB domain containing 24 | 268294 | ENSMUSG00000019826 |
| 5 | 17344794 | NA | Znrd1 | zinc ribbon domain containing, 1 | 66136 | ENSMUSG00000036315 |
| 6 | 17300261 | NA | Oxa1l | oxidase assembly 1-like | 69089 | ENSMUSG00000000959 |
| 7 | 17474547 | NA | Rtn2 | reticulon 2 (Z-band associated protein) | 20167 | ENSMUSG00000030401 |
| 8 | 17512740 | NA | Nob1 | NIN1/RPN12 binding protein 1 homolog (S. cerevisiae) | 67619 | ENSMUSG00000003848 |
| 9 | 17224540 | NA | Tuba4a | tubulin, alpha 4A | 22145 | ENSMUSG00000026202 |
| 10 | 17503910 | NA | Ogfod1 | 2-oxoglutarate and iron-dependent oxygenase domain containing 1 | 270086 | ENSMUSG00000033009 |
| 11 | 17456308 | NA | Kcnd2 | potassium voltage-gated channel, Shal-related family, member 2 | 16508 | ENSMUSG00000060882 |
| 12 | 17368171 | NA | Bmyc | brain expressed myelocytomatosis oncogene | 107771 | ENSMUSG00000049086 |
| 13 | 17350134 | NA | Pou4f3 | POU domain, class 4, transcription factor 3 | 18998 | ENSMUSG00000024497 |
| 14 | 17517723 | NA | Rpp25 | ribonuclease P 25 subunit (human) | 102614 | ENSMUSG00000062309 |
| 15 | 17443181 | NA | Dnajc30 | DnaJ (Hsp40) homolog, subfamily C, member 30 | 66114 | ENSMUSG00000061118 |
| 16 | 17319324 | NA | Dnalc4 | dynein, axonemal, light chain 4 | 54152 | ENSMUSG00000022420 |
| 17 | 17512103 | NA | Got2 | glutamate oxaloacetate transaminase 2, mitochondrial | 14719 | ENSMUSG00000031672 |
| 18 | 17369862 | NA | Dpm2 | dolichol-phosphate (beta-D) mannosyltransferase 2 | 13481 | ENSMUSG00000026810 |
| 19 | 17521652 | NA | Nicn1 | nicolin 1 | 66257 | ENSMUSG00000032606 |
| 20 | 17336829 | NA | Lsm2 | LSM2 homolog, U6 small nuclear RNA associated (S. cerevisiae) | 27756 | ENSMUSG00000007050 |
| 21 | 17288454 | NA | Irx4 | Iroquois related homeobox 4 (Drosophila) | 50916 | ENSMUSG00000021604 |
| 22 | 17467996 | NA | Mrpl19 | mitochondrial ribosomal protein L19 | 56284 | ENSMUSG00000030045 |
| 23 | 17322559 | NA | Hmox2 | heme oxygenase (decycling) 2 | 15369 | ENSMUSG00000004070 |
| 24 | 17537677 | NA | Drp2 | dystrophin related protein 2 | 13497 | ENSMUSG00000000223 |
| 25 | 17306758 | NA | Tm9sf1 | transmembrane 9 superfamily member 1 | 74140 | ENSMUSG00000002320 |
| 26 | 17504712 | NA | Exoc3l | exocyst complex component 3-like | 277978 | ENSMUSG00000043251 |
| 27 | 17321467 | NA | Tuba1b | tubulin, alpha 1B | 22143 | ENSMUSG00000023004 |
| 28 | 17288160 | NA | Cdk20 | cyclin-dependent kinase 20 | 105278 | ENSMUSG00000021483 |
| 29 | 17538096 | NA | Rnf128 | ring finger protein 128 | 66889 | ENSMUSG00000031438 |
| 30 | 17541597 | NA | Frmd7 | FERM domain containing 7 | 385354 | ENSMUSG00000036131 |
| 31 | 17263594 | NA | Atpaf2 | ATP synthase mitochondrial F1 complex assembly factor 2 | 246782 | ENSMUSG00000042709 |
| 32 | 17447099 | NA | Ctbp1 | C-terminal binding protein 1 | 13016 | ENSMUSG00000037373 |
| 33 | 17447089 | NA | Ctbp1 | C-terminal binding protein 1 | 13016 | ENSMUSG00000037373 |
| 34 | 17447100 | NA | Ctbp1 | C-terminal binding protein 1 | 13016 | ENSMUSG00000037373 |
| 35 | 17447098 | NA | Ctbp1 | C-terminal binding protein 1 | 13016 | ENSMUSG00000037373 |
| 36 | 17343617 | NA | Rab11b | RAB11B, member RAS oncogene family | 19326 | ENSMUSG00000077450 |
| 37 | 17379187 | NA | Ift52 | intraflagellar transport 52 | 245866 | ENSMUSG00000017858 |
| 38 | 17246284 | NA | Suox | sulfite oxidase | 211389 | ENSMUSG00000049858 |
| 39 | 17287579 | NA | Zfp346 | zinc finger protein 346 | 26919 | ENSMUSG00000021481 |
| 40 | 17224577 | NA | Resp18 | regulated endocrine-specific protein 18 | 19711 | ENSMUSG00000033061 |
| 41 | 17265082 | NA | Eif5a | eukaryotic translation initiation factor 5A | 276770 | ENSMUSG00000078812 |
| 42 | 17433602 | NA | Tprgl | transformation related protein 63 regulated like | 67808 | ENSMUSG00000029030 |
| 43 | 17344336 | NA | Tcf19 | transcription factor 19 | 106795 | ENSMUSG00000050410 |
| 44 | 17297750 | NA | Ppif | peptidylprolyl isomerase F (cyclophilin F) | 105675 | ENSMUSG00000021868 |
| 45 | 17233630 | NA | Psap | prosaposin | 19156 | ENSMUSG00000004207 |
| 46 | 17520177 | NA | Mthfs | 5, 10-methenyltetrahydrofolate synthetase | 107885 | ENSMUSG00000066442 |
| 47 | 17235368 | NA | Scamp4 | secretory carrier membrane protein 4 | 56214 | ENSMUSG00000078441 |
| 48 | 17369672 | NA | Ppapdc3 | phosphatidic acid phosphatase type 2 domain containing 3 | 227721 | ENSMUSG00000051373 |
| 49 | 17480102 | NA | Sytl2 | synaptotagmin-like 2 | 83671 | ENSMUSG00000030616 |
| 50 | 17521143 | NA | Wdr82 | WD repeat domain containing 82 | 77305 | ENSMUSG00000020257 |
| 51 | 17225499 | NA | Hes6 | hairy and enhancer of split 6 (Drosophila) | 55927 | ENSMUSG00000067071 |
| 52 | 17512434 | NA | Tppp3 | tubulin polymerization-promoting protein family member 3 | 67971 | ENSMUSG00000014846 |
| 53 | 17377583 | NA | Nsfl1c | NSFL1 (p97) cofactor (p47) | 386649 | ENSMUSG00000027455 |
| 54 | 17265175 | NA | 0610010K14Rik | RIKEN cDNA 0610010K14 gene | 104457 | ENSMUSG00000020831 |
| 55 | 17312944 | NA | Polr2f | polymerase (RNA) II (DNA directed) polypeptide F | 69833 | ENSMUSG00000033020 |
| 56 | 17312939 | NA | Polr2f | polymerase (RNA) II (DNA directed) polypeptide F | 69833 | ENSMUSG00000033020 |
| 57 | 17312941 | NA | Polr2f | polymerase (RNA) II (DNA directed) polypeptide F | 69833 | ENSMUSG00000033020 |
| 58 | 17329151 | NA | Alg3 | asparagine-linked glycosylation 3 (alpha-1,3-mannosyltransferase) | 208624 | ENSMUSG00000033809 |
| 59 | 17517576 | NA | Hmg20a | high mobility group 20A | 66867 | ENSMUSG00000032329 |
| 60 | 17393225 | NA | Pigu | phosphatidylinositol glycan anchor biosynthesis, class U | 228812 | ENSMUSG00000038383 |
| 61 | 17500391 | NA | Rnf122 | ring finger protein 122 | 68867 | ENSMUSG00000039328 |
| 62 | 17404011 | NA | Hey1 | hairy/enhancer-of-split related with YRPW motif 1 | 15213 | ENSMUSG00000040289 |
| 63 | 17400222 | NA | Vps72 | vacuolar protein sorting 72 (yeast) | 21427 | ENSMUSG00000008958 |
| 64 | 17499396 | NA | Fbxo25 | F-box protein 25 | 66822 | ENSMUSG00000038365 |
| 65 | 17246850 | NA | Zmat5 | zinc finger, matrin type 5 | 67178 | ENSMUSG00000009076 |
| 66 | 17324664 | NA | Dlg1 | discs, large homolog 1 (Drosophila) | 13383 | ENSMUSG00000022770 |
| 67 | 17396369 | NA | Nceh1 | arylacetamide deacetylase-like 1 | 320024 | ENSMUSG00000027698 |
| 68 | 17314556 | NA | Slc48a1 | solute carrier family 48 (heme transporter), member 1 | 67739 | ENSMUSG00000081534 |
| 69 | 17238846 | NA | Syne1 | synaptic nuclear envelope 1 | 64009 | ENSMUSG00000019769 ENSMUSG00000096054 |
| 70 | 17238906 | NA | Syne1 | synaptic nuclear envelope 1 | 64009 | ENSMUSG00000019769 ENSMUSG00000096054 |
| 71 | 17238890 | NA | Syne1 | synaptic nuclear envelope 1 | 64009 | ENSMUSG00000019769 ENSMUSG00000096054 |
| 72 | 17246209 | NA | Rpl41 | ribosomal protein L41 | 67945 | ENSMUSG00000093674 |
| 73 | 17502191 | NA | Mrpl34 | mitochondrial ribosomal protein L34 | 94065 | ENSMUSG00000034880 |
| 74 | 17529231 | NA | Phip | pleckstrin homology domain interacting protein | 83946 | ENSMUSG00000032253 |
| 75 | 17316625 | NA | Ubr5 | ubiquitin protein ligase E3 component n-recognin 5 | 70790 | ENSMUSG00000037487 |
| 76 | 17483220 | NA | Cdipt | CDP-diacylglycerol--inositol 3-phosphatidyltransferase (phosphatidylinositol synthase) | 52858 | ENSMUSG00000030682 |
| 77 | 17361975 | NA | Snx15 | sorting nexin 15 | 69024 | ENSMUSG00000024787 |
| 78 | 17245709 | NA | Os9 | amplified in osteosarcoma | 216440 | ENSMUSG00000040462 |
| 79 | 17512009 | NA | Csnk2a2 | casein kinase 2, alpha prime polypeptide | 13000 | ENSMUSG00000046707 |
| 80 | 17424319 | NA | Sigmar1 | sigma non-opioid intracellular receptor 1 | 18391 | ENSMUSG00000036078 |
| 81 | 17503816 | NA | Irx6 | Iroquois related homeobox 6 (Drosophila) | 64379 | ENSMUSG00000031738 |
| 82 | 17282649 | NA | Rps6kl1 | ribosomal protein S6 kinase-like 1 | 238323 | ENSMUSG00000019235 |
| 83 | 17260221 | NA | Pold2 | polymerase (DNA directed), delta 2, regulatory subunit | 18972 | ENSMUSG00000020471 |
| 84 | 17365493 | NA | Obfc1 | oligonucleotide/oligosaccharide-binding fold containing 1 | 108689 | ENSMUSG00000042694 |
| 85 | 17538790 | NA | Huwe1 | HECT, UBA and WWE domain containing 1 | 59026 | ENSMUSG00000025261 |
| 86 | 17446580 | NA | Shh | sonic hedgehog | 20423 | ENSMUSG00000002633 |
| 87 | 17229644 | NA | Tomm40l | translocase of outer mitochondrial membrane 40 homolog-like (yeast) | 641376 | ENSMUSG00000005674 |
| 88 | 17470879 | NA | Tpi1 | triosephosphate isomerase 1 | 21991 | ENSMUSG00000023456 |
| 89 | 17281971 | NA | Sgpp1 | sphingosine-1-phosphate phosphatase 1 | 81535 | ENSMUSG00000021054 |
| 90 | 17522887 | NA | Golga4 | golgi autoantigen, golgin subfamily a, 4 | 54214 | ENSMUSG00000038708 |
| 91 | 17230408 | NA | Adck3 | aarF domain containing kinase 3 | 67426 | ENSMUSG00000026489 |
| 92 | 17420582 | NA | Capzb | capping protein (actin filament) muscle Z-line, beta | 12345 | ENSMUSG00000028745 |
| 93 | 17352132 | NA | Txnl4a | thioredoxin-like 4A | 27366 | ENSMUSG00000057130 |
| 94 | 17273086 | NA | Nploc4 | nuclear protein localization 4 homolog (S. cerevisiae) | 217365 | ENSMUSG00000039703 |
| 95 | 17238549 | NA | Wibg | within bgcn homolog (Drosophila) | 78428 | ENSMUSG00000064030 |
| 96 | 17212355 | NA | Nck2 | non-catalytic region of tyrosine kinase adaptor protein 2 | 17974 | ENSMUSG00000066877 |
| 97 | 17275955 | NA | Atl1 | atlastin GTPase 1 | 73991 | ENSMUSG00000021066 |
| 98 | 17307695 | NA | Msra | methionine sulfoxide reductase A | 110265 | ENSMUSG00000054733 |
| 99 | 17303496 | NA | Fezf2 | Fez family zinc finger 2 | 54713 | ENSMUSG00000021743 |
| 100 | 17256565 | NA | Tubg2 | tubulin, gamma 2 | 103768 | ENSMUSG00000045007 |
| 101 | 17500275 | NA | Erlin2 | ER lipid raft associated 2 | 244373 | ENSMUSG00000031483 |
| 102 | 17345519 | NA | Rrp36 | ribosomal RNA processing 36 homolog (S. cerevisiae) | 224823 | ENSMUSG00000023971 |
| 103 | 17463422 | NA | Nrip2 | nuclear receptor interacting protein 2 | 60345 | ENSMUSG00000001520 |
| 104 | 17211335 | NA | Tfap2d | transcription factor AP-2, delta | 226896 | ENSMUSG00000042596 |
| 105 | 17299542 | NA | Tmem55b | transmembrane protein 55b | 219024 | ENSMUSG00000035953 |
| 106 | 17506631 | NA | Tubb3 | tubulin, beta 3 class III | 22152 | ENSMUSG00000062380 |
| 107 | 17253674 | NA | Poldip2 | polymerase (DNA-directed), delta interacting protein 2 | 67811 | ENSMUSG00000001100 |
| 108 | 17485194 | NA | Krtap5-4 | keratin associated protein 5-4 | 50775 | ENSMUSG00000045236 |
| 109 | 17321722 | NA | Tfcp2 | transcription factor CP2 | 21422 | ENSMUSG00000009733 |
| 110 | 17213153 | NA | Nif3l1 | Ngg1 interacting factor 3-like 1 (S. pombe) | 65102 | ENSMUSG00000026036 |
| 111 | 17235584 | NA | Dapk3 | death-associated protein kinase 3 | 13144 | ENSMUSG00000034974 |
| 112 | 17338670 | NA | Fsd1 | fibronectin type 3 and SPRY domain-containing protein | 240121 | ENSMUSG00000011589 |
| 113 | 17405819 | NA | B3galnt1 | UDP-GalNAc:betaGlcNAc beta 1,3-galactosaminyltransferase, polypeptide 1 | 26879 | ENSMUSG00000043300 |
| 114 | 17300411 | NA | Thtpa | thiamine triphosphatase | 105663 | ENSMUSG00000045691 |
| 115 | 17498821 | NA | Snapc2 | small nuclear RNA activating complex, polypeptide 2 | 102209 | ENSMUSG00000011837 |
| 116 | 17460879 | NA | Hdac11 | histone deacetylase 11 | 232232 | ENSMUSG00000034245 |
| 117 | 17328451 | NA | Mzt2 | mitotic spindle organizing protein 2 | 72083 | ENSMUSG00000022671 |
| 118 | 17256618 | NA | Vps25 | vacuolar protein sorting 25 (yeast) | 28084 | ENSMUSG00000078656 |
| 119 | 17444100 | NA | Chst12 | carbohydrate sulfotransferase 12 | 59031 | ENSMUSG00000036599 |
| 120 | 17517532 | NA | Isl2 | insulin related protein 2 (islet 2) | 104360 | ENSMUSG00000032318 |
| 121 | 17494637 | NA | Mrpl17 | mitochondrial ribosomal protein L17 | 27397 | ENSMUSG00000030879 |
| 122 | 17513871 | NA | Chmp1a | charged multivesicular body protein 1A | 234852 | ENSMUSG00000000743 |
| 123 | 17217666 | NA | Tmem9 | transmembrane protein 9 | 66241 | ENSMUSG00000026411 |
| 124 | 17484068 | NA | Lhpp | phospholysine phosphohistidine inorganic pyrophosphate phosphatase | 76429 | ENSMUSG00000030946 |
| 125 | 17497366 | NA | Ebf3 | early B cell factor 3 | 13593 | ENSMUSG00000010476 |
| 126 | 17288616 | NA | Tppp | tubulin polymerization promoting protein | 72948 | ENSMUSG00000021573 |
| 127 | 17452552 | NA | Rhof | ras homolog gene family, member f | 23912 | ENSMUSG00000029449 |
| 128 | 17407764 | NA | Prune | prune homolog (Drosophila) | 229589 | ENSMUSG00000015711 |
| 129 | 17524523 | NA | Eif3g | eukaryotic translation initiation factor 3, subunit G | 53356 | ENSMUSG00000070319 |
| 130 | 17269638 | NA | Rab5c | RAB5C, member RAS oncogene family | 19345 | ENSMUSG00000019173 |
| 131 | 17268995 | NA | Krt222 | keratin 222 | 268481 | ENSMUSG00000035849 |
| 132 | 17505367 | NA | Txnl4b | thioredoxin-like 4B | 234723 | ENSMUSG00000031723 |
| 133 | 17256549 | NA | Tubg1 | tubulin, gamma 1 | 103733 | ENSMUSG00000035198 |
| 134 | 17214293 | NA | Bcs1l | BCS1-like (yeast) | 66821 | ENSMUSG00000026172 |
| 135 | 17540982 | NA | Sept6 | septin 6 | 56526 | ENSMUSG00000050379 |
| 136 | 17497957 | NA | Chid1 | chitinase domain containing 1 | 68038 | ENSMUSG00000025512 |
| 137 | 17235268 | NA | Ndufs7 | NADH dehydrogenase (ubiquinone) Fe-S protein 7 | 75406 | ENSMUSG00000020153 |
| 138 | 17532045 | NA | Plcd1 | phospholipase C, delta 1 | 18799 | ENSMUSG00000010660 |
| 139 | 17447726 | NA | Hs3st1 | heparan sulfate (glucosamine) 3-O-sulfotransferase 1 | 15476 | ENSMUSG00000051022 |
| 140 | 17274448 | NA | Cpsf3 | cleavage and polyadenylation specificity factor 3 | 54451 | ENSMUSG00000054309 |
| 141 | 17454416 | NA | Zfand2a | zinc finger, AN1-type domain 2A | 100494 | ENSMUSG00000053581 |
| 142 | 17332333 | NA | Dscr3 | Down syndrome critical region gene 3 | 13185 | ENSMUSG00000022898 |
| 143 | 17332341 | NA | Dscr3 | Down syndrome critical region gene 3 | 13185 | ENSMUSG00000022898 |
| 144 | 17332343 | NA | Dscr3 | Down syndrome critical region gene 3 | 13185 | ENSMUSG00000022898 |
| 145 | 17332336 | NA | Dscr3 | Down syndrome critical region gene 3 | 13185 | ENSMUSG00000022898 |
| 146 | 17536264 | NA | Pcyt1b | phosphate cytidylyltransferase 1, choline, beta isoform | 236899 | ENSMUSG00000035246 |
| 147 | 17515170 | NA | Ilf3 | interleukin enhancer binding factor 3 | 16201 | ENSMUSG00000032178 |
| 148 | 17533055 | NA | Timm17b | translocase of inner mitochondrial membrane 17b | 21855 | ENSMUSG00000031158 |
| 149 | 17357213 | NA | Zbtb3 | zinc finger and BTB domain containing 3 | 75291 | ENSMUSG00000071661 |
| 150 | 17453106 | NA | Zfp11 | zinc finger protein 11 | 22648 | ENSMUSG00000051034 |
| 151 | 17430853 | NA | Med18 | mediator of RNA polymerase II transcription, subunit 18 homolog (yeast) | 67219 | ENSMUSG00000066042 |
| 152 | 17315558 | NA | Copz1 | coatomer protein complex, subunit zeta 1 | 56447 | ENSMUSG00000060992 |
| 153 | 17442714 | NA | Bri3bp | Bri3 binding protein | 76809 | ENSMUSG00000037905 |
| 154 | 17521014 | NA | Acad11 | acyl-Coenzyme A dehydrogenase family, member 11 | 102632 | ENSMUSG00000090150 |
| 155 | 17370285 | NA | Mrrf | mitochondrial ribosome recycling factor | 67871 | ENSMUSG00000026887 |
| 156 | 17361988 | NA | Arl2 | ADP-ribosylation factor-like 2 | 56327 | ENSMUSG00000024944 |
| 157 | 17229948 | NA | Dusp23 | dual specificity phosphatase 23 | 68440 | ENSMUSG00000026544 |
| 158 | 17502583 | NA | Mcm5 | minichromosome maintenance deficient 5, cell division cycle 46 (S. cerevisiae) | 17218 | ENSMUSG00000005410 |
| 159 | 17511296 | NA | Wdr83 | WD repeat domain containing 83 | 67836 | ENSMUSG00000005150 |
| 160 | 17539434 | NA | Ctps2 | cytidine 5'-triphosphate synthase 2 | 55936 | ENSMUSG00000031360 |
| 161 | 17341521 | NA | Thoc6 | THO complex 6 homolog (Drosophila) | 386612 | ENSMUSG00000041319 |
| 162 | 17250141 | NA | Zfp39 | zinc finger protein 39 | 22698 | ENSMUSG00000037001 |
| 163 | 17485815 | NA | Syt5 | synaptotagmin V | 53420 | ENSMUSG00000004961 |
| 164 | 17519718 | NA | Mto1 | mitochondrial translation optimization 1 homolog (S. cerevisiae) | 68291 | ENSMUSG00000032342 |
| 165 | 17266322 | NA | Eral1 | Era (G-protein)-like 1 (E. coli) | 57837 | ENSMUSG00000020832 |
| 166 | 17488134 | NA | Rab4b | RAB4B, member RAS oncogene family | 19342 | ENSMUSG00000053291 |
| 167 | 17521422 | NA | Hyal2 | hyaluronoglucosaminidase 2 | 15587 | ENSMUSG00000010047 |
| 168 | 17344453 | NA | Ppp1r18 | protein phosphatase 1, regulatory subunit 18 | 76448 | ENSMUSG00000034595 |
| 169 | 17317208 | NA | Derl1 | Der1-like domain family, member 1 | 67819 | ENSMUSG00000022365 |
| 170 | 17501544 | NA | Npy1r | neuropeptide Y receptor Y1 | 18166 | ENSMUSG00000036437 |
| 171 | 17376272 | NA | Nop56 | NOP56 ribonucleoprotein homolog (yeast) | 67134 | ENSMUSG00000027405 |
| 172 | 17468195 | NA | Stambp | STAM binding protein | 70527 | ENSMUSG00000006906 |
| 173 | 17408684 | NA | Dclre1b | DNA cross-link repair 1B, PSO2 homolog (S. cerevisiae) | 140917 | ENSMUSG00000027845 |
| 174 | 17443047 | NA | Caln1 | calneuron 1 | 140904 | ENSMUSG00000060371 |
| 175 | 17436077 | NA | Nrbp1 | nuclear receptor binding protein 1 | 192292 | ENSMUSG00000029148 |
| 176 | 17342015 | NA | Tbl3 | transducin (beta)-like 3 | 213773 | ENSMUSG00000040688 |
| 177 | 17543988 | NA | Taf9b | TAF9B RNA polymerase II, TATA box binding protein (TBP)-associated factor | 407786 | ENSMUSG00000047242 |
| 178 | 17509721 | NA | Tufm | Tu translation elongation factor, mitochondrial | 233870 | ENSMUSG00000073838 |
| 179 | 17459676 | NA | Retsat | retinol saturase (all trans retinol 13,14 reductase) | 67442 | ENSMUSG00000056666 |
| 180 | 17337269 | NA | Nrm | nurim (nuclear envelope membrane protein) | 106582 | ENSMUSG00000059791 |
| 181 | 17361463 | NA | Rab1b | RAB1B, member RAS oncogene family | 76308 | ENSMUSG00000024870 |
| 182 | 17279858 | NA | Fkbp1b | FK506 binding protein 1b | 14226 | ENSMUSG00000020635 |
| 183 | 17488463 | NA | Med29 | mediator complex subunit 29 | 67224 | ENSMUSG00000003444 |
| 184 | 17391270 | NA | Kcnip3 | Kv channel interacting protein 3, calsenilin | 56461 | ENSMUSG00000079056 |
| 185 | 17324576 | NA | Hrasls | HRAS-like suppressor | 27281 | ENSMUSG00000022525 |
| 186 | 17217580 | NA | Arl8a | ADP-ribosylation factor-like 8A | 68724 | ENSMUSG00000026426 |
| 187 | 17460099 | NA | Vax2 | ventral anterior homeobox containing gene 2 | 24113 | ENSMUSG00000034777 |
| 188 | 17348933 | NA | Mapre2 | microtubule-associated protein, RP/EB family, member 2 | 212307 | ENSMUSG00000024277 |
| 189 | 17257060 | NA | Nmt1 | N-myristoyltransferase 1 | 18107 | ENSMUSG00000020936 |
| 190 | 17266489 | NA | Tmem97 | transmembrane protein 97 | 69071 | ENSMUSG00000037278 |
| 191 | 17365369 | NA | Cuedc2 | CUE domain containing 2 | 67116 | ENSMUSG00000036748 |
| 192 | 17299353 | NA | Ktn1 | kinectin 1 | 16709 | ENSMUSG00000021843 |
| 193 | 17292107 | NA | Tbc1d7 | TBC1 domain family, member 7 | 67046 | ENSMUSG00000021368 |
| 194 | 17506854 | NA | Tsnax | translin-associated factor X | 53424 | ENSMUSG00000056820 |
| 195 | 17512479 | NA | Acd | adrenocortical dysplasia | 497652 | ENSMUSG00000038000 |
| 196 | 17288716 | NA | Glrx | glutaredoxin | 93692 | ENSMUSG00000021591 |
| 197 | 17540050 | NA | Ebp | phenylalkylamine Ca2+ antagonist (emopamil) binding protein | 13595 | ENSMUSG00000031168 |
| 198 | 17429495 | NA | Nfyc | nuclear transcription factor-Y gamma | 18046 | ENSMUSG00000032897 |
| 199 | 17361855 | NA | Pola2 | polymerase (DNA directed), alpha 2 | 18969 | ENSMUSG00000024833 |
| 200 | 17512463 | NA | Atp6v0d1 | ATPase, H+ transporting, lysosomal V0 subunit D1 | 11972 | ENSMUSG00000013160 |
| 201 | 17512466 | NA | Atp6v0d1 | ATPase, H+ transporting, lysosomal V0 subunit D1 | 11972 | ENSMUSG00000013160 |
| 202 | 17230945 | NA | Smyd2 | SET and MYND domain containing 2 | 226830 | ENSMUSG00000026603 |
| 203 | 17313008 | NA | Cby1 | chibby homolog 1 (Drosophila) | 73739 | ENSMUSG00000022428 |
| 204 | 17251607 | NA | Trappc1 | trafficking protein particle complex 1 | 245828 | ENSMUSG00000049299 |
| 205 | 17521448 | NA | Hyal3 | hyaluronoglucosaminidase 3 | 109685 | ENSMUSG00000036091 |
| 206 | 17535434 | NA | Nsdhl | NAD(P) dependent steroid dehydrogenase-like | 18194 | ENSMUSG00000031349 |
| 207 | 17299750 | NA | Tox4 | TOX high mobility group box family member 4 | 268741 | ENSMUSG00000016831 |
| 208 | 17468113 | NA | Ino80b | INO80 complex subunit B | 70020 | ENSMUSG00000030034 |
| 209 | 17502390 | NA | Rab8a | RAB8A, member RAS oncogene family | 17274 | ENSMUSG00000003037 |
| 210 | 17503023 | NA | Asf1b | ASF1 anti-silencing function 1 homolog B (S. cerevisiae) | 66929 | ENSMUSG00000005470 |
| 211 | 17258457 | NA | Sap30bp | SAP30 binding protein | 57230 | ENSMUSG00000020755 |
| 212 | 17328829 | NA | Slc25a1 | solute carrier family 25 (mitochondrial carrier, citrate transporter), member 1 | 13358 | ENSMUSG00000003528 |
| 213 | 17454256 | NA | Taf6 | TAF6 RNA polymerase II, TATA box binding protein (TBP)-associated factor | 21343 | ENSMUSG00000036980 |
| 214 | 17432967 | NA | Ubiad1 | UbiA prenyltransferase domain containing 1 | 71707 | ENSMUSG00000047719 |
| 215 | 17526273 | NA | Trappc4 | trafficking protein particle complex 4 | 60409 | ENSMUSG00000032112 |
| 216 | 17526271 | NA | Trappc4 | trafficking protein particle complex 4 | 60409 | ENSMUSG00000032112 |
| 217 | 17526272 | NA | Trappc4 | trafficking protein particle complex 4 | 60409 | ENSMUSG00000032112 |
| 218 | 17465696 | NA | Slc35b4 | solute carrier family 35, member B4 | 58246 | ENSMUSG00000018999 |
| 219 | 17231118 | NA | Rcor3 | REST corepressor 3 | 214742 | ENSMUSG00000037395 |
| 220 | 17379554 | NA | Zswim1 | zinc finger, SWIM domain containing 1 | 71971 | ENSMUSG00000017764 |
| 221 | 17298407 | NA | Bap1 | Brca1 associated protein 1 | 104416 | ENSMUSG00000021901 |
| 222 | 17318877 | NA | Txn2 | thioredoxin 2 | 56551 | ENSMUSG00000005354 |
| 223 | 17453809 | NA | Ap1s1 | adaptor protein complex AP-1, sigma 1 | 11769 | ENSMUSG00000004849 |
| 224 | 17329163 | NA | Camk2n2 | calcium/calmodulin-dependent protein kinase II inhibitor 2 | 73047 | ENSMUSG00000051146 |
| 225 | 17405174 | NA | Cog6 | component of oligomeric golgi complex 6 | 67542 | ENSMUSG00000027742 |
| 226 | 17382914 | NA | Dnlz | DNL-type zinc finger | 52838 | ENSMUSG00000075467 |
| 227 | 17451443 | NA | Coro1c | coronin, actin binding protein 1C | 23790 | ENSMUSG00000004530 |
| 228 | 17273280 | NA | Stra13 | stimulated by retinoic acid 13 | 20892 | ENSMUSG00000025144 |
| 229 | 17301342 | NA | Ints9 | integrator complex subunit 9 | 210925 | ENSMUSG00000021975 |
| 230 | 17241409 | NA | Srgn | serglycin | 19073 | ENSMUSG00000020077 |
| 231 | 17322200 | NA | Aaas | achalasia, adrenocortical insufficiency, alacrimia | 223921 | ENSMUSG00000036678 |
| 232 | 17393357 | NA | Eif6 | eukaryotic translation initiation factor 6 | 16418 | ENSMUSG00000027613 |
| 233 | 17412593 | NA | Srsf12 | serine/arginine-rich splicing factor 12 | 272009 | ENSMUSG00000054679 |
| 234 | 17451816 | NA | Hspb8 | heat shock protein 8 | 80888 | ENSMUSG00000041548 |
| 235 | 17213990 | NA | Atic | 5-aminoimidazole-4-carboxamide ribonucleotide formyltransferase/IMP cyclohydrolase | 108147 | ENSMUSG00000026192 |
| 236 | 17504160 | NA | Polr2c | polymerase (RNA) II (DNA directed) polypeptide C | 20021 | ENSMUSG00000031783 |
| 237 | 17455093 | NA | Zkscan14 | zinc finger with KRAB and SCAN domains 14 | 67235 | ENSMUSG00000029627 |
| 238 | 17488544 | NA | Nfkbib | nuclear factor of kappa light polypeptide gene enhancer in B cells inhibitor, beta | 18036 | ENSMUSG00000030595 |
| 239 | 17508691 | NA | Rbpms | RNA binding protein gene with multiple splicing | 19663 | ENSMUSG00000031586 |
| 240 | 17222001 | NA | Prim2 | DNA primase, p58 subunit | 19076 | ENSMUSG00000026134 |
| 241 | 17232649 | NA | Fyn | Fyn proto-oncogene | 14360 | ENSMUSG00000019843 |
| 242 | 17328810 | NA | Dgcr14 | DiGeorge syndrome critical region gene 14 | 27886 | ENSMUSG00000003527 |
| 243 | 17474534 | NA | Opa3 | optic atrophy 3 | 403187 | ENSMUSG00000052214 |
| 244 | 17395844 | NA | Stmn3 | stathmin-like 3 | 20262 | ENSMUSG00000027581 |
| 245 | 17229466 | NA | Hsd17b7 | hydroxysteroid (17-beta) dehydrogenase 7 | 15490 | ENSMUSG00000026675 |
| 246 | 17242318 | NA | Gm7138 | predicted gene 7138 | 634517 | ENSMUSG00000095593 |
| 247 | 17219286 | NA | Dedd | death effector domain-containing | 21945 | ENSMUSG00000013973 |
| 248 | 17463150 | NA | Vamp1 | vesicle-associated membrane protein 1 | 22317 | ENSMUSG00000030337 |
| 249 | 17306861 | NA | Dhrs1 | dehydrogenase/reductase (SDR family) member 1 | 52585 | ENSMUSG00000002332 |
| 250 | 17306864 | NA | Dhrs1 | dehydrogenase/reductase (SDR family) member 1 | 52585 | ENSMUSG00000002332 |
| 251 | 17306860 | NA | Dhrs1 | dehydrogenase/reductase (SDR family) member 1 | 52585 | ENSMUSG00000002332 |
| 252 | 17306856 | NA | Dhrs1 | dehydrogenase/reductase (SDR family) member 1 | 52585 | ENSMUSG00000002332 |
| 253 | 17306865 | NA | Dhrs1 | dehydrogenase/reductase (SDR family) member 1 | 52585 | ENSMUSG00000002332 |
| 254 | 17418571 | NA | Trappc3 | trafficking protein particle complex 3 | 27096 | ENSMUSG00000028847 |
| 255 | 17359945 | NA | Gbf1 | golgi-specific brefeldin A-resistance factor 1 | 107338 | ENSMUSG00000025224 |
| 256 | 17334495 | NA | Nme3 | NME/NM23 nucleoside diphosphate kinase 3 | 79059 | ENSMUSG00000073435 |
| 257 | 17338043 | NA | Yipf3 | Yip1 domain family, member 3 | 28064 | ENSMUSG00000071074 |
| 258 | 17396024 | NA | Stmn2 | stathmin-like 2 | 20257 | ENSMUSG00000027500 |
| 259 | 17535752 | NA | Emd | emerin | 13726 | ENSMUSG00000001964 |
| 260 | 17383588 | NA | Ccbl1 | cysteine conjugate-beta lyase 1 | 70266 | ENSMUSG00000039648 |
| 261 | 17313199 | NA | Adsl | adenylosuccinate lyase | 11564 | ENSMUSG00000022407 |
| 262 | 17232215 | NA | Moxd1 | monooxygenase, DBH-like 1 | 59012 | ENSMUSG00000020000 |
| 263 | 17418447 | NA | Meaf6 | MYST/Esa1-associated factor 6 | 70088 | ENSMUSG00000028863 |
| 264 | 17356202 | NA | Pold4 | polymerase (DNA-directed), delta 4 | 69745 | ENSMUSG00000024854 |
| 265 | 17400638 | NA | Pex11b | peroxisomal biogenesis factor 11 beta | 18632 | ENSMUSG00000028102 |
| 266 | 17240123 | NA | Clvs2 | clavesin 2 | 215890 | ENSMUSG00000019785 |
| 267 | 17421972 | NA | Errfi1 | ERBB receptor feedback inhibitor 1 | 74155 | ENSMUSG00000028967 |
| 268 | 17462975 | NA | Mlf2 | myeloid leukemia factor 2 | 30853 | ENSMUSG00000030120 |

  
  

| **Database:cellular component      &nbspName:intracellular membrane-bounded organelle      &nbspID:GO:0043231** | | | | | | |
| --- | --- | --- | --- | --- | --- | --- |
| C=8342; O=228; E=161.21; R=1.41; rawP=6.73e-12; adjP=3.12e-10 | | | | | | |
| Index | UserID | Value | Gene Symbol | Gene Name | EntrezGene | Ensembl |
| 1 | 17476273 | NA | Zfp382 | zinc finger protein 382 | 233060 | ENSMUSG00000074220 |
| 2 | 17255719 | NA | Mrpl10 | mitochondrial ribosomal protein L10 | 107732 | ENSMUSG00000001445 |
| 3 | 17413221 | NA | Unc13b | unc-13 homolog B (C. elegans) | 22249 | ENSMUSG00000028456 |
| 4 | 17232843 | NA | Zbtb24 | zinc finger and BTB domain containing 24 | 268294 | ENSMUSG00000019826 |
| 5 | 17344794 | NA | Znrd1 | zinc ribbon domain containing, 1 | 66136 | ENSMUSG00000036315 |
| 6 | 17300261 | NA | Oxa1l | oxidase assembly 1-like | 69089 | ENSMUSG00000000959 |
| 7 | 17474547 | NA | Rtn2 | reticulon 2 (Z-band associated protein) | 20167 | ENSMUSG00000030401 |
| 8 | 17512740 | NA | Nob1 | NIN1/RPN12 binding protein 1 homolog (S. cerevisiae) | 67619 | ENSMUSG00000003848 |
| 9 | 17503910 | NA | Ogfod1 | 2-oxoglutarate and iron-dependent oxygenase domain containing 1 | 270086 | ENSMUSG00000033009 |
| 10 | 17368171 | NA | Bmyc | brain expressed myelocytomatosis oncogene | 107771 | ENSMUSG00000049086 |
| 11 | 17350134 | NA | Pou4f3 | POU domain, class 4, transcription factor 3 | 18998 | ENSMUSG00000024497 |
| 12 | 17517723 | NA | Rpp25 | ribonuclease P 25 subunit (human) | 102614 | ENSMUSG00000062309 |
| 13 | 17443181 | NA | Dnajc30 | DnaJ (Hsp40) homolog, subfamily C, member 30 | 66114 | ENSMUSG00000061118 |
| 14 | 17512103 | NA | Got2 | glutamate oxaloacetate transaminase 2, mitochondrial | 14719 | ENSMUSG00000031672 |
| 15 | 17369862 | NA | Dpm2 | dolichol-phosphate (beta-D) mannosyltransferase 2 | 13481 | ENSMUSG00000026810 |
| 16 | 17521652 | NA | Nicn1 | nicolin 1 | 66257 | ENSMUSG00000032606 |
| 17 | 17336829 | NA | Lsm2 | LSM2 homolog, U6 small nuclear RNA associated (S. cerevisiae) | 27756 | ENSMUSG00000007050 |
| 18 | 17288454 | NA | Irx4 | Iroquois related homeobox 4 (Drosophila) | 50916 | ENSMUSG00000021604 |
| 19 | 17467996 | NA | Mrpl19 | mitochondrial ribosomal protein L19 | 56284 | ENSMUSG00000030045 |
| 20 | 17322559 | NA | Hmox2 | heme oxygenase (decycling) 2 | 15369 | ENSMUSG00000004070 |
| 21 | 17306758 | NA | Tm9sf1 | transmembrane 9 superfamily member 1 | 74140 | ENSMUSG00000002320 |
| 22 | 17504712 | NA | Exoc3l | exocyst complex component 3-like | 277978 | ENSMUSG00000043251 |
| 23 | 17288160 | NA | Cdk20 | cyclin-dependent kinase 20 | 105278 | ENSMUSG00000021483 |
| 24 | 17538096 | NA | Rnf128 | ring finger protein 128 | 66889 | ENSMUSG00000031438 |
| 25 | 17263594 | NA | Atpaf2 | ATP synthase mitochondrial F1 complex assembly factor 2 | 246782 | ENSMUSG00000042709 |
| 26 | 17447099 | NA | Ctbp1 | C-terminal binding protein 1 | 13016 | ENSMUSG00000037373 |
| 27 | 17447089 | NA | Ctbp1 | C-terminal binding protein 1 | 13016 | ENSMUSG00000037373 |
| 28 | 17447100 | NA | Ctbp1 | C-terminal binding protein 1 | 13016 | ENSMUSG00000037373 |
| 29 | 17447098 | NA | Ctbp1 | C-terminal binding protein 1 | 13016 | ENSMUSG00000037373 |
| 30 | 17343617 | NA | Rab11b | RAB11B, member RAS oncogene family | 19326 | ENSMUSG00000077450 |
| 31 | 17246284 | NA | Suox | sulfite oxidase | 211389 | ENSMUSG00000049858 |
| 32 | 17287579 | NA | Zfp346 | zinc finger protein 346 | 26919 | ENSMUSG00000021481 |
| 33 | 17224577 | NA | Resp18 | regulated endocrine-specific protein 18 | 19711 | ENSMUSG00000033061 |
| 34 | 17265082 | NA | Eif5a | eukaryotic translation initiation factor 5A | 276770 | ENSMUSG00000078812 |
| 35 | 17433602 | NA | Tprgl | transformation related protein 63 regulated like | 67808 | ENSMUSG00000029030 |
| 36 | 17344336 | NA | Tcf19 | transcription factor 19 | 106795 | ENSMUSG00000050410 |
| 37 | 17297750 | NA | Ppif | peptidylprolyl isomerase F (cyclophilin F) | 105675 | ENSMUSG00000021868 |
| 38 | 17233630 | NA | Psap | prosaposin | 19156 | ENSMUSG00000004207 |
| 39 | 17520177 | NA | Mthfs | 5, 10-methenyltetrahydrofolate synthetase | 107885 | ENSMUSG00000066442 |
| 40 | 17235368 | NA | Scamp4 | secretory carrier membrane protein 4 | 56214 | ENSMUSG00000078441 |
| 41 | 17369672 | NA | Ppapdc3 | phosphatidic acid phosphatase type 2 domain containing 3 | 227721 | ENSMUSG00000051373 |
| 42 | 17480102 | NA | Sytl2 | synaptotagmin-like 2 | 83671 | ENSMUSG00000030616 |
| 43 | 17521143 | NA | Wdr82 | WD repeat domain containing 82 | 77305 | ENSMUSG00000020257 |
| 44 | 17225499 | NA | Hes6 | hairy and enhancer of split 6 (Drosophila) | 55927 | ENSMUSG00000067071 |
| 45 | 17377583 | NA | Nsfl1c | NSFL1 (p97) cofactor (p47) | 386649 | ENSMUSG00000027455 |
| 46 | 17265175 | NA | 0610010K14Rik | RIKEN cDNA 0610010K14 gene | 104457 | ENSMUSG00000020831 |
| 47 | 17312944 | NA | Polr2f | polymerase (RNA) II (DNA directed) polypeptide F | 69833 | ENSMUSG00000033020 |
| 48 | 17312939 | NA | Polr2f | polymerase (RNA) II (DNA directed) polypeptide F | 69833 | ENSMUSG00000033020 |
| 49 | 17312941 | NA | Polr2f | polymerase (RNA) II (DNA directed) polypeptide F | 69833 | ENSMUSG00000033020 |
| 50 | 17329151 | NA | Alg3 | asparagine-linked glycosylation 3 (alpha-1,3-mannosyltransferase) | 208624 | ENSMUSG00000033809 |
| 51 | 17517576 | NA | Hmg20a | high mobility group 20A | 66867 | ENSMUSG00000032329 |
| 52 | 17393225 | NA | Pigu | phosphatidylinositol glycan anchor biosynthesis, class U | 228812 | ENSMUSG00000038383 |
| 53 | 17500391 | NA | Rnf122 | ring finger protein 122 | 68867 | ENSMUSG00000039328 |
| 54 | 17404011 | NA | Hey1 | hairy/enhancer-of-split related with YRPW motif 1 | 15213 | ENSMUSG00000040289 |
| 55 | 17400222 | NA | Vps72 | vacuolar protein sorting 72 (yeast) | 21427 | ENSMUSG00000008958 |
| 56 | 17499396 | NA | Fbxo25 | F-box protein 25 | 66822 | ENSMUSG00000038365 |
| 57 | 17246850 | NA | Zmat5 | zinc finger, matrin type 5 | 67178 | ENSMUSG00000009076 |
| 58 | 17324664 | NA | Dlg1 | discs, large homolog 1 (Drosophila) | 13383 | ENSMUSG00000022770 |
| 59 | 17396369 | NA | Nceh1 | arylacetamide deacetylase-like 1 | 320024 | ENSMUSG00000027698 |
| 60 | 17314556 | NA | Slc48a1 | solute carrier family 48 (heme transporter), member 1 | 67739 | ENSMUSG00000081534 |
| 61 | 17238846 | NA | Syne1 | synaptic nuclear envelope 1 | 64009 | ENSMUSG00000019769 ENSMUSG00000096054 |
| 62 | 17238906 | NA | Syne1 | synaptic nuclear envelope 1 | 64009 | ENSMUSG00000019769 ENSMUSG00000096054 |
| 63 | 17238890 | NA | Syne1 | synaptic nuclear envelope 1 | 64009 | ENSMUSG00000019769 ENSMUSG00000096054 |
| 64 | 17502191 | NA | Mrpl34 | mitochondrial ribosomal protein L34 | 94065 | ENSMUSG00000034880 |
| 65 | 17529231 | NA | Phip | pleckstrin homology domain interacting protein | 83946 | ENSMUSG00000032253 |
| 66 | 17316625 | NA | Ubr5 | ubiquitin protein ligase E3 component n-recognin 5 | 70790 | ENSMUSG00000037487 |
| 67 | 17483220 | NA | Cdipt | CDP-diacylglycerol--inositol 3-phosphatidyltransferase (phosphatidylinositol synthase) | 52858 | ENSMUSG00000030682 |
| 68 | 17361975 | NA | Snx15 | sorting nexin 15 | 69024 | ENSMUSG00000024787 |
| 69 | 17245709 | NA | Os9 | amplified in osteosarcoma | 216440 | ENSMUSG00000040462 |
| 70 | 17512009 | NA | Csnk2a2 | casein kinase 2, alpha prime polypeptide | 13000 | ENSMUSG00000046707 |
| 71 | 17424319 | NA | Sigmar1 | sigma non-opioid intracellular receptor 1 | 18391 | ENSMUSG00000036078 |
| 72 | 17503816 | NA | Irx6 | Iroquois related homeobox 6 (Drosophila) | 64379 | ENSMUSG00000031738 |
| 73 | 17260221 | NA | Pold2 | polymerase (DNA directed), delta 2, regulatory subunit | 18972 | ENSMUSG00000020471 |
| 74 | 17365493 | NA | Obfc1 | oligonucleotide/oligosaccharide-binding fold containing 1 | 108689 | ENSMUSG00000042694 |
| 75 | 17538790 | NA | Huwe1 | HECT, UBA and WWE domain containing 1 | 59026 | ENSMUSG00000025261 |
| 76 | 17446580 | NA | Shh | sonic hedgehog | 20423 | ENSMUSG00000002633 |
| 77 | 17229644 | NA | Tomm40l | translocase of outer mitochondrial membrane 40 homolog-like (yeast) | 641376 | ENSMUSG00000005674 |
| 78 | 17470879 | NA | Tpi1 | triosephosphate isomerase 1 | 21991 | ENSMUSG00000023456 |
| 79 | 17281971 | NA | Sgpp1 | sphingosine-1-phosphate phosphatase 1 | 81535 | ENSMUSG00000021054 |
| 80 | 17522887 | NA | Golga4 | golgi autoantigen, golgin subfamily a, 4 | 54214 | ENSMUSG00000038708 |
| 81 | 17230408 | NA | Adck3 | aarF domain containing kinase 3 | 67426 | ENSMUSG00000026489 |
| 82 | 17352132 | NA | Txnl4a | thioredoxin-like 4A | 27366 | ENSMUSG00000057130 |
| 83 | 17273086 | NA | Nploc4 | nuclear protein localization 4 homolog (S. cerevisiae) | 217365 | ENSMUSG00000039703 |
| 84 | 17238549 | NA | Wibg | within bgcn homolog (Drosophila) | 78428 | ENSMUSG00000064030 |
| 85 | 17212355 | NA | Nck2 | non-catalytic region of tyrosine kinase adaptor protein 2 | 17974 | ENSMUSG00000066877 |
| 86 | 17275955 | NA | Atl1 | atlastin GTPase 1 | 73991 | ENSMUSG00000021066 |
| 87 | 17307695 | NA | Msra | methionine sulfoxide reductase A | 110265 | ENSMUSG00000054733 |
| 88 | 17303496 | NA | Fezf2 | Fez family zinc finger 2 | 54713 | ENSMUSG00000021743 |
| 89 | 17500275 | NA | Erlin2 | ER lipid raft associated 2 | 244373 | ENSMUSG00000031483 |
| 90 | 17345519 | NA | Rrp36 | ribosomal RNA processing 36 homolog (S. cerevisiae) | 224823 | ENSMUSG00000023971 |
| 91 | 17463422 | NA | Nrip2 | nuclear receptor interacting protein 2 | 60345 | ENSMUSG00000001520 |
| 92 | 17211335 | NA | Tfap2d | transcription factor AP-2, delta | 226896 | ENSMUSG00000042596 |
| 93 | 17299542 | NA | Tmem55b | transmembrane protein 55b | 219024 | ENSMUSG00000035953 |
| 94 | 17253674 | NA | Poldip2 | polymerase (DNA-directed), delta interacting protein 2 | 67811 | ENSMUSG00000001100 |
| 95 | 17321722 | NA | Tfcp2 | transcription factor CP2 | 21422 | ENSMUSG00000009733 |
| 96 | 17213153 | NA | Nif3l1 | Ngg1 interacting factor 3-like 1 (S. pombe) | 65102 | ENSMUSG00000026036 |
| 97 | 17235584 | NA | Dapk3 | death-associated protein kinase 3 | 13144 | ENSMUSG00000034974 |
| 98 | 17338670 | NA | Fsd1 | fibronectin type 3 and SPRY domain-containing protein | 240121 | ENSMUSG00000011589 |
| 99 | 17405819 | NA | B3galnt1 | UDP-GalNAc:betaGlcNAc beta 1,3-galactosaminyltransferase, polypeptide 1 | 26879 | ENSMUSG00000043300 |
| 100 | 17300411 | NA | Thtpa | thiamine triphosphatase | 105663 | ENSMUSG00000045691 |
| 101 | 17498821 | NA | Snapc2 | small nuclear RNA activating complex, polypeptide 2 | 102209 | ENSMUSG00000011837 |
| 102 | 17460879 | NA | Hdac11 | histone deacetylase 11 | 232232 | ENSMUSG00000034245 |
| 103 | 17256618 | NA | Vps25 | vacuolar protein sorting 25 (yeast) | 28084 | ENSMUSG00000078656 |
| 104 | 17444100 | NA | Chst12 | carbohydrate sulfotransferase 12 | 59031 | ENSMUSG00000036599 |
| 105 | 17517532 | NA | Isl2 | insulin related protein 2 (islet 2) | 104360 | ENSMUSG00000032318 |
| 106 | 17494637 | NA | Mrpl17 | mitochondrial ribosomal protein L17 | 27397 | ENSMUSG00000030879 |
| 107 | 17513871 | NA | Chmp1a | charged multivesicular body protein 1A | 234852 | ENSMUSG00000000743 |
| 108 | 17217666 | NA | Tmem9 | transmembrane protein 9 | 66241 | ENSMUSG00000026411 |
| 109 | 17484068 | NA | Lhpp | phospholysine phosphohistidine inorganic pyrophosphate phosphatase | 76429 | ENSMUSG00000030946 |
| 110 | 17497366 | NA | Ebf3 | early B cell factor 3 | 13593 | ENSMUSG00000010476 |
| 111 | 17288616 | NA | Tppp | tubulin polymerization promoting protein | 72948 | ENSMUSG00000021573 |
| 112 | 17407764 | NA | Prune | prune homolog (Drosophila) | 229589 | ENSMUSG00000015711 |
| 113 | 17524523 | NA | Eif3g | eukaryotic translation initiation factor 3, subunit G | 53356 | ENSMUSG00000070319 |
| 114 | 17269638 | NA | Rab5c | RAB5C, member RAS oncogene family | 19345 | ENSMUSG00000019173 |
| 115 | 17505367 | NA | Txnl4b | thioredoxin-like 4B | 234723 | ENSMUSG00000031723 |
| 116 | 17256549 | NA | Tubg1 | tubulin, gamma 1 | 103733 | ENSMUSG00000035198 |
| 117 | 17214293 | NA | Bcs1l | BCS1-like (yeast) | 66821 | ENSMUSG00000026172 |
| 118 | 17540982 | NA | Sept6 | septin 6 | 56526 | ENSMUSG00000050379 |
| 119 | 17497957 | NA | Chid1 | chitinase domain containing 1 | 68038 | ENSMUSG00000025512 |
| 120 | 17235268 | NA | Ndufs7 | NADH dehydrogenase (ubiquinone) Fe-S protein 7 | 75406 | ENSMUSG00000020153 |
| 121 | 17532045 | NA | Plcd1 | phospholipase C, delta 1 | 18799 | ENSMUSG00000010660 |
| 122 | 17447726 | NA | Hs3st1 | heparan sulfate (glucosamine) 3-O-sulfotransferase 1 | 15476 | ENSMUSG00000051022 |
| 123 | 17274448 | NA | Cpsf3 | cleavage and polyadenylation specificity factor 3 | 54451 | ENSMUSG00000054309 |
| 124 | 17454416 | NA | Zfand2a | zinc finger, AN1-type domain 2A | 100494 | ENSMUSG00000053581 |
| 125 | 17332333 | NA | Dscr3 | Down syndrome critical region gene 3 | 13185 | ENSMUSG00000022898 |
| 126 | 17332341 | NA | Dscr3 | Down syndrome critical region gene 3 | 13185 | ENSMUSG00000022898 |
| 127 | 17332343 | NA | Dscr3 | Down syndrome critical region gene 3 | 13185 | ENSMUSG00000022898 |
| 128 | 17332336 | NA | Dscr3 | Down syndrome critical region gene 3 | 13185 | ENSMUSG00000022898 |
| 129 | 17536264 | NA | Pcyt1b | phosphate cytidylyltransferase 1, choline, beta isoform | 236899 | ENSMUSG00000035246 |
| 130 | 17515170 | NA | Ilf3 | interleukin enhancer binding factor 3 | 16201 | ENSMUSG00000032178 |
| 131 | 17533055 | NA | Timm17b | translocase of inner mitochondrial membrane 17b | 21855 | ENSMUSG00000031158 |
| 132 | 17357213 | NA | Zbtb3 | zinc finger and BTB domain containing 3 | 75291 | ENSMUSG00000071661 |
| 133 | 17453106 | NA | Zfp11 | zinc finger protein 11 | 22648 | ENSMUSG00000051034 |
| 134 | 17430853 | NA | Med18 | mediator of RNA polymerase II transcription, subunit 18 homolog (yeast) | 67219 | ENSMUSG00000066042 |
| 135 | 17315558 | NA | Copz1 | coatomer protein complex, subunit zeta 1 | 56447 | ENSMUSG00000060992 |
| 136 | 17442714 | NA | Bri3bp | Bri3 binding protein | 76809 | ENSMUSG00000037905 |
| 137 | 17521014 | NA | Acad11 | acyl-Coenzyme A dehydrogenase family, member 11 | 102632 | ENSMUSG00000090150 |
| 138 | 17370285 | NA | Mrrf | mitochondrial ribosome recycling factor | 67871 | ENSMUSG00000026887 |
| 139 | 17361988 | NA | Arl2 | ADP-ribosylation factor-like 2 | 56327 | ENSMUSG00000024944 |
| 140 | 17229948 | NA | Dusp23 | dual specificity phosphatase 23 | 68440 | ENSMUSG00000026544 |
| 141 | 17502583 | NA | Mcm5 | minichromosome maintenance deficient 5, cell division cycle 46 (S. cerevisiae) | 17218 | ENSMUSG00000005410 |
| 142 | 17511296 | NA | Wdr83 | WD repeat domain containing 83 | 67836 | ENSMUSG00000005150 |
| 143 | 17539434 | NA | Ctps2 | cytidine 5'-triphosphate synthase 2 | 55936 | ENSMUSG00000031360 |
| 144 | 17341521 | NA | Thoc6 | THO complex 6 homolog (Drosophila) | 386612 | ENSMUSG00000041319 |
| 145 | 17250141 | NA | Zfp39 | zinc finger protein 39 | 22698 | ENSMUSG00000037001 |
| 146 | 17485815 | NA | Syt5 | synaptotagmin V | 53420 | ENSMUSG00000004961 |
| 147 | 17519718 | NA | Mto1 | mitochondrial translation optimization 1 homolog (S. cerevisiae) | 68291 | ENSMUSG00000032342 |
| 148 | 17266322 | NA | Eral1 | Era (G-protein)-like 1 (E. coli) | 57837 | ENSMUSG00000020832 |
| 149 | 17488134 | NA | Rab4b | RAB4B, member RAS oncogene family | 19342 | ENSMUSG00000053291 |
| 150 | 17521422 | NA | Hyal2 | hyaluronoglucosaminidase 2 | 15587 | ENSMUSG00000010047 |
| 151 | 17317208 | NA | Derl1 | Der1-like domain family, member 1 | 67819 | ENSMUSG00000022365 |
| 152 | 17501544 | NA | Npy1r | neuropeptide Y receptor Y1 | 18166 | ENSMUSG00000036437 |
| 153 | 17376272 | NA | Nop56 | NOP56 ribonucleoprotein homolog (yeast) | 67134 | ENSMUSG00000027405 |
| 154 | 17468195 | NA | Stambp | STAM binding protein | 70527 | ENSMUSG00000006906 |
| 155 | 17408684 | NA | Dclre1b | DNA cross-link repair 1B, PSO2 homolog (S. cerevisiae) | 140917 | ENSMUSG00000027845 |
| 156 | 17443047 | NA | Caln1 | calneuron 1 | 140904 | ENSMUSG00000060371 |
| 157 | 17436077 | NA | Nrbp1 | nuclear receptor binding protein 1 | 192292 | ENSMUSG00000029148 |
| 158 | 17342015 | NA | Tbl3 | transducin (beta)-like 3 | 213773 | ENSMUSG00000040688 |
| 159 | 17543988 | NA | Taf9b | TAF9B RNA polymerase II, TATA box binding protein (TBP)-associated factor | 407786 | ENSMUSG00000047242 |
| 160 | 17509721 | NA | Tufm | Tu translation elongation factor, mitochondrial | 233870 | ENSMUSG00000073838 |
| 161 | 17459676 | NA | Retsat | retinol saturase (all trans retinol 13,14 reductase) | 67442 | ENSMUSG00000056666 |
| 162 | 17337269 | NA | Nrm | nurim (nuclear envelope membrane protein) | 106582 | ENSMUSG00000059791 |
| 163 | 17361463 | NA | Rab1b | RAB1B, member RAS oncogene family | 76308 | ENSMUSG00000024870 |
| 164 | 17279858 | NA | Fkbp1b | FK506 binding protein 1b | 14226 | ENSMUSG00000020635 |
| 165 | 17488463 | NA | Med29 | mediator complex subunit 29 | 67224 | ENSMUSG00000003444 |
| 166 | 17391270 | NA | Kcnip3 | Kv channel interacting protein 3, calsenilin | 56461 | ENSMUSG00000079056 |
| 167 | 17324576 | NA | Hrasls | HRAS-like suppressor | 27281 | ENSMUSG00000022525 |
| 168 | 17217580 | NA | Arl8a | ADP-ribosylation factor-like 8A | 68724 | ENSMUSG00000026426 |
| 169 | 17460099 | NA | Vax2 | ventral anterior homeobox containing gene 2 | 24113 | ENSMUSG00000034777 |
| 170 | 17266489 | NA | Tmem97 | transmembrane protein 97 | 69071 | ENSMUSG00000037278 |
| 171 | 17365369 | NA | Cuedc2 | CUE domain containing 2 | 67116 | ENSMUSG00000036748 |
| 172 | 17299353 | NA | Ktn1 | kinectin 1 | 16709 | ENSMUSG00000021843 |
| 173 | 17506854 | NA | Tsnax | translin-associated factor X | 53424 | ENSMUSG00000056820 |
| 174 | 17512479 | NA | Acd | adrenocortical dysplasia | 497652 | ENSMUSG00000038000 |
| 175 | 17288716 | NA | Glrx | glutaredoxin | 93692 | ENSMUSG00000021591 |
| 176 | 17540050 | NA | Ebp | phenylalkylamine Ca2+ antagonist (emopamil) binding protein | 13595 | ENSMUSG00000031168 |
| 177 | 17429495 | NA | Nfyc | nuclear transcription factor-Y gamma | 18046 | ENSMUSG00000032897 |
| 178 | 17361855 | NA | Pola2 | polymerase (DNA directed), alpha 2 | 18969 | ENSMUSG00000024833 |
| 179 | 17512463 | NA | Atp6v0d1 | ATPase, H+ transporting, lysosomal V0 subunit D1 | 11972 | ENSMUSG00000013160 |
| 180 | 17512466 | NA | Atp6v0d1 | ATPase, H+ transporting, lysosomal V0 subunit D1 | 11972 | ENSMUSG00000013160 |
| 181 | 17230945 | NA | Smyd2 | SET and MYND domain containing 2 | 226830 | ENSMUSG00000026603 |
| 182 | 17313008 | NA | Cby1 | chibby homolog 1 (Drosophila) | 73739 | ENSMUSG00000022428 |
| 183 | 17251607 | NA | Trappc1 | trafficking protein particle complex 1 | 245828 | ENSMUSG00000049299 |
| 184 | 17521448 | NA | Hyal3 | hyaluronoglucosaminidase 3 | 109685 | ENSMUSG00000036091 |
| 185 | 17535434 | NA | Nsdhl | NAD(P) dependent steroid dehydrogenase-like | 18194 | ENSMUSG00000031349 |
| 186 | 17299750 | NA | Tox4 | TOX high mobility group box family member 4 | 268741 | ENSMUSG00000016831 |
| 187 | 17468113 | NA | Ino80b | INO80 complex subunit B | 70020 | ENSMUSG00000030034 |
| 188 | 17502390 | NA | Rab8a | RAB8A, member RAS oncogene family | 17274 | ENSMUSG00000003037 |
| 189 | 17503023 | NA | Asf1b | ASF1 anti-silencing function 1 homolog B (S. cerevisiae) | 66929 | ENSMUSG00000005470 |
| 190 | 17258457 | NA | Sap30bp | SAP30 binding protein | 57230 | ENSMUSG00000020755 |
| 191 | 17328829 | NA | Slc25a1 | solute carrier family 25 (mitochondrial carrier, citrate transporter), member 1 | 13358 | ENSMUSG00000003528 |
| 192 | 17454256 | NA | Taf6 | TAF6 RNA polymerase II, TATA box binding protein (TBP)-associated factor | 21343 | ENSMUSG00000036980 |
| 193 | 17432967 | NA | Ubiad1 | UbiA prenyltransferase domain containing 1 | 71707 | ENSMUSG00000047719 |
| 194 | 17526273 | NA | Trappc4 | trafficking protein particle complex 4 | 60409 | ENSMUSG00000032112 |
| 195 | 17526271 | NA | Trappc4 | trafficking protein particle complex 4 | 60409 | ENSMUSG00000032112 |
| 196 | 17526272 | NA | Trappc4 | trafficking protein particle complex 4 | 60409 | ENSMUSG00000032112 |
| 197 | 17465696 | NA | Slc35b4 | solute carrier family 35, member B4 | 58246 | ENSMUSG00000018999 |
| 198 | 17231118 | NA | Rcor3 | REST corepressor 3 | 214742 | ENSMUSG00000037395 |
| 199 | 17379554 | NA | Zswim1 | zinc finger, SWIM domain containing 1 | 71971 | ENSMUSG00000017764 |
| 200 | 17298407 | NA | Bap1 | Brca1 associated protein 1 | 104416 | ENSMUSG00000021901 |
| 201 | 17318877 | NA | Txn2 | thioredoxin 2 | 56551 | ENSMUSG00000005354 |
| 202 | 17453809 | NA | Ap1s1 | adaptor protein complex AP-1, sigma 1 | 11769 | ENSMUSG00000004849 |
| 203 | 17329163 | NA | Camk2n2 | calcium/calmodulin-dependent protein kinase II inhibitor 2 | 73047 | ENSMUSG00000051146 |
| 204 | 17405174 | NA | Cog6 | component of oligomeric golgi complex 6 | 67542 | ENSMUSG00000027742 |
| 205 | 17382914 | NA | Dnlz | DNL-type zinc finger | 52838 | ENSMUSG00000075467 |
| 206 | 17273280 | NA | Stra13 | stimulated by retinoic acid 13 | 20892 | ENSMUSG00000025144 |
| 207 | 17301342 | NA | Ints9 | integrator complex subunit 9 | 210925 | ENSMUSG00000021975 |
| 208 | 17241409 | NA | Srgn | serglycin | 19073 | ENSMUSG00000020077 |
| 209 | 17322200 | NA | Aaas | achalasia, adrenocortical insufficiency, alacrimia | 223921 | ENSMUSG00000036678 |
| 210 | 17393357 | NA | Eif6 | eukaryotic translation initiation factor 6 | 16418 | ENSMUSG00000027613 |
| 211 | 17412593 | NA | Srsf12 | serine/arginine-rich splicing factor 12 | 272009 | ENSMUSG00000054679 |
| 212 | 17451816 | NA | Hspb8 | heat shock protein 8 | 80888 | ENSMUSG00000041548 |
| 213 | 17213990 | NA | Atic | 5-aminoimidazole-4-carboxamide ribonucleotide formyltransferase/IMP cyclohydrolase | 108147 | ENSMUSG00000026192 |
| 214 | 17504160 | NA | Polr2c | polymerase (RNA) II (DNA directed) polypeptide C | 20021 | ENSMUSG00000031783 |
| 215 | 17455093 | NA | Zkscan14 | zinc finger with KRAB and SCAN domains 14 | 67235 | ENSMUSG00000029627 |
| 216 | 17488544 | NA | Nfkbib | nuclear factor of kappa light polypeptide gene enhancer in B cells inhibitor, beta | 18036 | ENSMUSG00000030595 |
| 217 | 17508691 | NA | Rbpms | RNA binding protein gene with multiple splicing | 19663 | ENSMUSG00000031586 |
| 218 | 17222001 | NA | Prim2 | DNA primase, p58 subunit | 19076 | ENSMUSG00000026134 |
| 219 | 17232649 | NA | Fyn | Fyn proto-oncogene | 14360 | ENSMUSG00000019843 |
| 220 | 17328810 | NA | Dgcr14 | DiGeorge syndrome critical region gene 14 | 27886 | ENSMUSG00000003527 |
| 221 | 17474534 | NA | Opa3 | optic atrophy 3 | 403187 | ENSMUSG00000052214 |
| 222 | 17395844 | NA | Stmn3 | stathmin-like 3 | 20262 | ENSMUSG00000027581 |
| 223 | 17229466 | NA | Hsd17b7 | hydroxysteroid (17-beta) dehydrogenase 7 | 15490 | ENSMUSG00000026675 |
| 224 | 17219286 | NA | Dedd | death effector domain-containing | 21945 | ENSMUSG00000013973 |
| 225 | 17463150 | NA | Vamp1 | vesicle-associated membrane protein 1 | 22317 | ENSMUSG00000030337 |
| 226 | 17306861 | NA | Dhrs1 | dehydrogenase/reductase (SDR family) member 1 | 52585 | ENSMUSG00000002332 |
| 227 | 17306864 | NA | Dhrs1 | dehydrogenase/reductase (SDR family) member 1 | 52585 | ENSMUSG00000002332 |
| 228 | 17306860 | NA | Dhrs1 | dehydrogenase/reductase (SDR family) member 1 | 52585 | ENSMUSG00000002332 |
| 229 | 17306856 | NA | Dhrs1 | dehydrogenase/reductase (SDR family) member 1 | 52585 | ENSMUSG00000002332 |
| 230 | 17306865 | NA | Dhrs1 | dehydrogenase/reductase (SDR family) member 1 | 52585 | ENSMUSG00000002332 |
| 231 | 17418571 | NA | Trappc3 | trafficking protein particle complex 3 | 27096 | ENSMUSG00000028847 |
| 232 | 17359945 | NA | Gbf1 | golgi-specific brefeldin A-resistance factor 1 | 107338 | ENSMUSG00000025224 |
| 233 | 17334495 | NA | Nme3 | NME/NM23 nucleoside diphosphate kinase 3 | 79059 | ENSMUSG00000073435 |
| 234 | 17338043 | NA | Yipf3 | Yip1 domain family, member 3 | 28064 | ENSMUSG00000071074 |
| 235 | 17396024 | NA | Stmn2 | stathmin-like 2 | 20257 | ENSMUSG00000027500 |
| 236 | 17535752 | NA | Emd | emerin | 13726 | ENSMUSG00000001964 |
| 237 | 17383588 | NA | Ccbl1 | cysteine conjugate-beta lyase 1 | 70266 | ENSMUSG00000039648 |
| 238 | 17313199 | NA | Adsl | adenylosuccinate lyase | 11564 | ENSMUSG00000022407 |
| 239 | 17232215 | NA | Moxd1 | monooxygenase, DBH-like 1 | 59012 | ENSMUSG00000020000 |
| 240 | 17418447 | NA | Meaf6 | MYST/Esa1-associated factor 6 | 70088 | ENSMUSG00000028863 |
| 241 | 17356202 | NA | Pold4 | polymerase (DNA-directed), delta 4 | 69745 | ENSMUSG00000024854 |
| 242 | 17400638 | NA | Pex11b | peroxisomal biogenesis factor 11 beta | 18632 | ENSMUSG00000028102 |
| 243 | 17240123 | NA | Clvs2 | clavesin 2 | 215890 | ENSMUSG00000019785 |
| 244 | 17421972 | NA | Errfi1 | ERBB receptor feedback inhibitor 1 | 74155 | ENSMUSG00000028967 |
| 245 | 17462975 | NA | Mlf2 | myeloid leukemia factor 2 | 30853 | ENSMUSG00000030120 |

  
  

| **Database:cellular component      &nbspName:membrane-bounded organelle      &nbspID:GO:0043227** | | | | | | |
| --- | --- | --- | --- | --- | --- | --- |
| C=8360; O=228; E=161.56; R=1.41; rawP=8.64e-12; adjP=3.34e-10 | | | | | | |
| Index | UserID | Value | Gene Symbol | Gene Name | EntrezGene | Ensembl |
| 1 | 17476273 | NA | Zfp382 | zinc finger protein 382 | 233060 | ENSMUSG00000074220 |
| 2 | 17255719 | NA | Mrpl10 | mitochondrial ribosomal protein L10 | 107732 | ENSMUSG00000001445 |
| 3 | 17413221 | NA | Unc13b | unc-13 homolog B (C. elegans) | 22249 | ENSMUSG00000028456 |
| 4 | 17232843 | NA | Zbtb24 | zinc finger and BTB domain containing 24 | 268294 | ENSMUSG00000019826 |
| 5 | 17344794 | NA | Znrd1 | zinc ribbon domain containing, 1 | 66136 | ENSMUSG00000036315 |
| 6 | 17300261 | NA | Oxa1l | oxidase assembly 1-like | 69089 | ENSMUSG00000000959 |
| 7 | 17474547 | NA | Rtn2 | reticulon 2 (Z-band associated protein) | 20167 | ENSMUSG00000030401 |
| 8 | 17512740 | NA | Nob1 | NIN1/RPN12 binding protein 1 homolog (S. cerevisiae) | 67619 | ENSMUSG00000003848 |
| 9 | 17503910 | NA | Ogfod1 | 2-oxoglutarate and iron-dependent oxygenase domain containing 1 | 270086 | ENSMUSG00000033009 |
| 10 | 17368171 | NA | Bmyc | brain expressed myelocytomatosis oncogene | 107771 | ENSMUSG00000049086 |
| 11 | 17350134 | NA | Pou4f3 | POU domain, class 4, transcription factor 3 | 18998 | ENSMUSG00000024497 |
| 12 | 17517723 | NA | Rpp25 | ribonuclease P 25 subunit (human) | 102614 | ENSMUSG00000062309 |
| 13 | 17443181 | NA | Dnajc30 | DnaJ (Hsp40) homolog, subfamily C, member 30 | 66114 | ENSMUSG00000061118 |
| 14 | 17512103 | NA | Got2 | glutamate oxaloacetate transaminase 2, mitochondrial | 14719 | ENSMUSG00000031672 |
| 15 | 17369862 | NA | Dpm2 | dolichol-phosphate (beta-D) mannosyltransferase 2 | 13481 | ENSMUSG00000026810 |
| 16 | 17521652 | NA | Nicn1 | nicolin 1 | 66257 | ENSMUSG00000032606 |
| 17 | 17336829 | NA | Lsm2 | LSM2 homolog, U6 small nuclear RNA associated (S. cerevisiae) | 27756 | ENSMUSG00000007050 |
| 18 | 17288454 | NA | Irx4 | Iroquois related homeobox 4 (Drosophila) | 50916 | ENSMUSG00000021604 |
| 19 | 17467996 | NA | Mrpl19 | mitochondrial ribosomal protein L19 | 56284 | ENSMUSG00000030045 |
| 20 | 17322559 | NA | Hmox2 | heme oxygenase (decycling) 2 | 15369 | ENSMUSG00000004070 |
| 21 | 17306758 | NA | Tm9sf1 | transmembrane 9 superfamily member 1 | 74140 | ENSMUSG00000002320 |
| 22 | 17504712 | NA | Exoc3l | exocyst complex component 3-like | 277978 | ENSMUSG00000043251 |
| 23 | 17288160 | NA | Cdk20 | cyclin-dependent kinase 20 | 105278 | ENSMUSG00000021483 |
| 24 | 17538096 | NA | Rnf128 | ring finger protein 128 | 66889 | ENSMUSG00000031438 |
| 25 | 17263594 | NA | Atpaf2 | ATP synthase mitochondrial F1 complex assembly factor 2 | 246782 | ENSMUSG00000042709 |
| 26 | 17447099 | NA | Ctbp1 | C-terminal binding protein 1 | 13016 | ENSMUSG00000037373 |
| 27 | 17447089 | NA | Ctbp1 | C-terminal binding protein 1 | 13016 | ENSMUSG00000037373 |
| 28 | 17447100 | NA | Ctbp1 | C-terminal binding protein 1 | 13016 | ENSMUSG00000037373 |
| 29 | 17447098 | NA | Ctbp1 | C-terminal binding protein 1 | 13016 | ENSMUSG00000037373 |
| 30 | 17343617 | NA | Rab11b | RAB11B, member RAS oncogene family | 19326 | ENSMUSG00000077450 |
| 31 | 17246284 | NA | Suox | sulfite oxidase | 211389 | ENSMUSG00000049858 |
| 32 | 17287579 | NA | Zfp346 | zinc finger protein 346 | 26919 | ENSMUSG00000021481 |
| 33 | 17224577 | NA | Resp18 | regulated endocrine-specific protein 18 | 19711 | ENSMUSG00000033061 |
| 34 | 17265082 | NA | Eif5a | eukaryotic translation initiation factor 5A | 276770 | ENSMUSG00000078812 |
| 35 | 17433602 | NA | Tprgl | transformation related protein 63 regulated like | 67808 | ENSMUSG00000029030 |
| 36 | 17344336 | NA | Tcf19 | transcription factor 19 | 106795 | ENSMUSG00000050410 |
| 37 | 17297750 | NA | Ppif | peptidylprolyl isomerase F (cyclophilin F) | 105675 | ENSMUSG00000021868 |
| 38 | 17233630 | NA | Psap | prosaposin | 19156 | ENSMUSG00000004207 |
| 39 | 17520177 | NA | Mthfs | 5, 10-methenyltetrahydrofolate synthetase | 107885 | ENSMUSG00000066442 |
| 40 | 17235368 | NA | Scamp4 | secretory carrier membrane protein 4 | 56214 | ENSMUSG00000078441 |
| 41 | 17369672 | NA | Ppapdc3 | phosphatidic acid phosphatase type 2 domain containing 3 | 227721 | ENSMUSG00000051373 |
| 42 | 17480102 | NA | Sytl2 | synaptotagmin-like 2 | 83671 | ENSMUSG00000030616 |
| 43 | 17521143 | NA | Wdr82 | WD repeat domain containing 82 | 77305 | ENSMUSG00000020257 |
| 44 | 17225499 | NA | Hes6 | hairy and enhancer of split 6 (Drosophila) | 55927 | ENSMUSG00000067071 |
| 45 | 17377583 | NA | Nsfl1c | NSFL1 (p97) cofactor (p47) | 386649 | ENSMUSG00000027455 |
| 46 | 17265175 | NA | 0610010K14Rik | RIKEN cDNA 0610010K14 gene | 104457 | ENSMUSG00000020831 |
| 47 | 17312944 | NA | Polr2f | polymerase (RNA) II (DNA directed) polypeptide F | 69833 | ENSMUSG00000033020 |
| 48 | 17312939 | NA | Polr2f | polymerase (RNA) II (DNA directed) polypeptide F | 69833 | ENSMUSG00000033020 |
| 49 | 17312941 | NA | Polr2f | polymerase (RNA) II (DNA directed) polypeptide F | 69833 | ENSMUSG00000033020 |
| 50 | 17329151 | NA | Alg3 | asparagine-linked glycosylation 3 (alpha-1,3-mannosyltransferase) | 208624 | ENSMUSG00000033809 |
| 51 | 17517576 | NA | Hmg20a | high mobility group 20A | 66867 | ENSMUSG00000032329 |
| 52 | 17393225 | NA | Pigu | phosphatidylinositol glycan anchor biosynthesis, class U | 228812 | ENSMUSG00000038383 |
| 53 | 17500391 | NA | Rnf122 | ring finger protein 122 | 68867 | ENSMUSG00000039328 |
| 54 | 17404011 | NA | Hey1 | hairy/enhancer-of-split related with YRPW motif 1 | 15213 | ENSMUSG00000040289 |
| 55 | 17400222 | NA | Vps72 | vacuolar protein sorting 72 (yeast) | 21427 | ENSMUSG00000008958 |
| 56 | 17499396 | NA | Fbxo25 | F-box protein 25 | 66822 | ENSMUSG00000038365 |
| 57 | 17246850 | NA | Zmat5 | zinc finger, matrin type 5 | 67178 | ENSMUSG00000009076 |
| 58 | 17324664 | NA | Dlg1 | discs, large homolog 1 (Drosophila) | 13383 | ENSMUSG00000022770 |
| 59 | 17396369 | NA | Nceh1 | arylacetamide deacetylase-like 1 | 320024 | ENSMUSG00000027698 |
| 60 | 17314556 | NA | Slc48a1 | solute carrier family 48 (heme transporter), member 1 | 67739 | ENSMUSG00000081534 |
| 61 | 17238846 | NA | Syne1 | synaptic nuclear envelope 1 | 64009 | ENSMUSG00000019769 ENSMUSG00000096054 |
| 62 | 17238906 | NA | Syne1 | synaptic nuclear envelope 1 | 64009 | ENSMUSG00000019769 ENSMUSG00000096054 |
| 63 | 17238890 | NA | Syne1 | synaptic nuclear envelope 1 | 64009 | ENSMUSG00000019769 ENSMUSG00000096054 |
| 64 | 17502191 | NA | Mrpl34 | mitochondrial ribosomal protein L34 | 94065 | ENSMUSG00000034880 |
| 65 | 17529231 | NA | Phip | pleckstrin homology domain interacting protein | 83946 | ENSMUSG00000032253 |
| 66 | 17316625 | NA | Ubr5 | ubiquitin protein ligase E3 component n-recognin 5 | 70790 | ENSMUSG00000037487 |
| 67 | 17483220 | NA | Cdipt | CDP-diacylglycerol--inositol 3-phosphatidyltransferase (phosphatidylinositol synthase) | 52858 | ENSMUSG00000030682 |
| 68 | 17361975 | NA | Snx15 | sorting nexin 15 | 69024 | ENSMUSG00000024787 |
| 69 | 17245709 | NA | Os9 | amplified in osteosarcoma | 216440 | ENSMUSG00000040462 |
| 70 | 17512009 | NA | Csnk2a2 | casein kinase 2, alpha prime polypeptide | 13000 | ENSMUSG00000046707 |
| 71 | 17424319 | NA | Sigmar1 | sigma non-opioid intracellular receptor 1 | 18391 | ENSMUSG00000036078 |
| 72 | 17503816 | NA | Irx6 | Iroquois related homeobox 6 (Drosophila) | 64379 | ENSMUSG00000031738 |
| 73 | 17260221 | NA | Pold2 | polymerase (DNA directed), delta 2, regulatory subunit | 18972 | ENSMUSG00000020471 |
| 74 | 17365493 | NA | Obfc1 | oligonucleotide/oligosaccharide-binding fold containing 1 | 108689 | ENSMUSG00000042694 |
| 75 | 17538790 | NA | Huwe1 | HECT, UBA and WWE domain containing 1 | 59026 | ENSMUSG00000025261 |
| 76 | 17446580 | NA | Shh | sonic hedgehog | 20423 | ENSMUSG00000002633 |
| 77 | 17229644 | NA | Tomm40l | translocase of outer mitochondrial membrane 40 homolog-like (yeast) | 641376 | ENSMUSG00000005674 |
| 78 | 17470879 | NA | Tpi1 | triosephosphate isomerase 1 | 21991 | ENSMUSG00000023456 |
| 79 | 17281971 | NA | Sgpp1 | sphingosine-1-phosphate phosphatase 1 | 81535 | ENSMUSG00000021054 |
| 80 | 17522887 | NA | Golga4 | golgi autoantigen, golgin subfamily a, 4 | 54214 | ENSMUSG00000038708 |
| 81 | 17230408 | NA | Adck3 | aarF domain containing kinase 3 | 67426 | ENSMUSG00000026489 |
| 82 | 17352132 | NA | Txnl4a | thioredoxin-like 4A | 27366 | ENSMUSG00000057130 |
| 83 | 17273086 | NA | Nploc4 | nuclear protein localization 4 homolog (S. cerevisiae) | 217365 | ENSMUSG00000039703 |
| 84 | 17238549 | NA | Wibg | within bgcn homolog (Drosophila) | 78428 | ENSMUSG00000064030 |
| 85 | 17212355 | NA | Nck2 | non-catalytic region of tyrosine kinase adaptor protein 2 | 17974 | ENSMUSG00000066877 |
| 86 | 17275955 | NA | Atl1 | atlastin GTPase 1 | 73991 | ENSMUSG00000021066 |
| 87 | 17307695 | NA | Msra | methionine sulfoxide reductase A | 110265 | ENSMUSG00000054733 |
| 88 | 17303496 | NA | Fezf2 | Fez family zinc finger 2 | 54713 | ENSMUSG00000021743 |
| 89 | 17500275 | NA | Erlin2 | ER lipid raft associated 2 | 244373 | ENSMUSG00000031483 |
| 90 | 17345519 | NA | Rrp36 | ribosomal RNA processing 36 homolog (S. cerevisiae) | 224823 | ENSMUSG00000023971 |
| 91 | 17463422 | NA | Nrip2 | nuclear receptor interacting protein 2 | 60345 | ENSMUSG00000001520 |
| 92 | 17211335 | NA | Tfap2d | transcription factor AP-2, delta | 226896 | ENSMUSG00000042596 |
| 93 | 17299542 | NA | Tmem55b | transmembrane protein 55b | 219024 | ENSMUSG00000035953 |
| 94 | 17253674 | NA | Poldip2 | polymerase (DNA-directed), delta interacting protein 2 | 67811 | ENSMUSG00000001100 |
| 95 | 17321722 | NA | Tfcp2 | transcription factor CP2 | 21422 | ENSMUSG00000009733 |
| 96 | 17213153 | NA | Nif3l1 | Ngg1 interacting factor 3-like 1 (S. pombe) | 65102 | ENSMUSG00000026036 |
| 97 | 17235584 | NA | Dapk3 | death-associated protein kinase 3 | 13144 | ENSMUSG00000034974 |
| 98 | 17338670 | NA | Fsd1 | fibronectin type 3 and SPRY domain-containing protein | 240121 | ENSMUSG00000011589 |
| 99 | 17405819 | NA | B3galnt1 | UDP-GalNAc:betaGlcNAc beta 1,3-galactosaminyltransferase, polypeptide 1 | 26879 | ENSMUSG00000043300 |
| 100 | 17300411 | NA | Thtpa | thiamine triphosphatase | 105663 | ENSMUSG00000045691 |
| 101 | 17498821 | NA | Snapc2 | small nuclear RNA activating complex, polypeptide 2 | 102209 | ENSMUSG00000011837 |
| 102 | 17460879 | NA | Hdac11 | histone deacetylase 11 | 232232 | ENSMUSG00000034245 |
| 103 | 17256618 | NA | Vps25 | vacuolar protein sorting 25 (yeast) | 28084 | ENSMUSG00000078656 |
| 104 | 17444100 | NA | Chst12 | carbohydrate sulfotransferase 12 | 59031 | ENSMUSG00000036599 |
| 105 | 17517532 | NA | Isl2 | insulin related protein 2 (islet 2) | 104360 | ENSMUSG00000032318 |
| 106 | 17494637 | NA | Mrpl17 | mitochondrial ribosomal protein L17 | 27397 | ENSMUSG00000030879 |
| 107 | 17513871 | NA | Chmp1a | charged multivesicular body protein 1A | 234852 | ENSMUSG00000000743 |
| 108 | 17217666 | NA | Tmem9 | transmembrane protein 9 | 66241 | ENSMUSG00000026411 |
| 109 | 17484068 | NA | Lhpp | phospholysine phosphohistidine inorganic pyrophosphate phosphatase | 76429 | ENSMUSG00000030946 |
| 110 | 17497366 | NA | Ebf3 | early B cell factor 3 | 13593 | ENSMUSG00000010476 |
| 111 | 17288616 | NA | Tppp | tubulin polymerization promoting protein | 72948 | ENSMUSG00000021573 |
| 112 | 17407764 | NA | Prune | prune homolog (Drosophila) | 229589 | ENSMUSG00000015711 |
| 113 | 17524523 | NA | Eif3g | eukaryotic translation initiation factor 3, subunit G | 53356 | ENSMUSG00000070319 |
| 114 | 17269638 | NA | Rab5c | RAB5C, member RAS oncogene family | 19345 | ENSMUSG00000019173 |
| 115 | 17505367 | NA | Txnl4b | thioredoxin-like 4B | 234723 | ENSMUSG00000031723 |
| 116 | 17256549 | NA | Tubg1 | tubulin, gamma 1 | 103733 | ENSMUSG00000035198 |
| 117 | 17214293 | NA | Bcs1l | BCS1-like (yeast) | 66821 | ENSMUSG00000026172 |
| 118 | 17540982 | NA | Sept6 | septin 6 | 56526 | ENSMUSG00000050379 |
| 119 | 17497957 | NA | Chid1 | chitinase domain containing 1 | 68038 | ENSMUSG00000025512 |
| 120 | 17235268 | NA | Ndufs7 | NADH dehydrogenase (ubiquinone) Fe-S protein 7 | 75406 | ENSMUSG00000020153 |
| 121 | 17532045 | NA | Plcd1 | phospholipase C, delta 1 | 18799 | ENSMUSG00000010660 |
| 122 | 17447726 | NA | Hs3st1 | heparan sulfate (glucosamine) 3-O-sulfotransferase 1 | 15476 | ENSMUSG00000051022 |
| 123 | 17274448 | NA | Cpsf3 | cleavage and polyadenylation specificity factor 3 | 54451 | ENSMUSG00000054309 |
| 124 | 17454416 | NA | Zfand2a | zinc finger, AN1-type domain 2A | 100494 | ENSMUSG00000053581 |
| 125 | 17332333 | NA | Dscr3 | Down syndrome critical region gene 3 | 13185 | ENSMUSG00000022898 |
| 126 | 17332341 | NA | Dscr3 | Down syndrome critical region gene 3 | 13185 | ENSMUSG00000022898 |
| 127 | 17332343 | NA | Dscr3 | Down syndrome critical region gene 3 | 13185 | ENSMUSG00000022898 |
| 128 | 17332336 | NA | Dscr3 | Down syndrome critical region gene 3 | 13185 | ENSMUSG00000022898 |
| 129 | 17536264 | NA | Pcyt1b | phosphate cytidylyltransferase 1, choline, beta isoform | 236899 | ENSMUSG00000035246 |
| 130 | 17515170 | NA | Ilf3 | interleukin enhancer binding factor 3 | 16201 | ENSMUSG00000032178 |
| 131 | 17533055 | NA | Timm17b | translocase of inner mitochondrial membrane 17b | 21855 | ENSMUSG00000031158 |
| 132 | 17357213 | NA | Zbtb3 | zinc finger and BTB domain containing 3 | 75291 | ENSMUSG00000071661 |
| 133 | 17453106 | NA | Zfp11 | zinc finger protein 11 | 22648 | ENSMUSG00000051034 |
| 134 | 17430853 | NA | Med18 | mediator of RNA polymerase II transcription, subunit 18 homolog (yeast) | 67219 | ENSMUSG00000066042 |
| 135 | 17315558 | NA | Copz1 | coatomer protein complex, subunit zeta 1 | 56447 | ENSMUSG00000060992 |
| 136 | 17442714 | NA | Bri3bp | Bri3 binding protein | 76809 | ENSMUSG00000037905 |
| 137 | 17521014 | NA | Acad11 | acyl-Coenzyme A dehydrogenase family, member 11 | 102632 | ENSMUSG00000090150 |
| 138 | 17370285 | NA | Mrrf | mitochondrial ribosome recycling factor | 67871 | ENSMUSG00000026887 |
| 139 | 17361988 | NA | Arl2 | ADP-ribosylation factor-like 2 | 56327 | ENSMUSG00000024944 |
| 140 | 17229948 | NA | Dusp23 | dual specificity phosphatase 23 | 68440 | ENSMUSG00000026544 |
| 141 | 17502583 | NA | Mcm5 | minichromosome maintenance deficient 5, cell division cycle 46 (S. cerevisiae) | 17218 | ENSMUSG00000005410 |
| 142 | 17511296 | NA | Wdr83 | WD repeat domain containing 83 | 67836 | ENSMUSG00000005150 |
| 143 | 17539434 | NA | Ctps2 | cytidine 5'-triphosphate synthase 2 | 55936 | ENSMUSG00000031360 |
| 144 | 17341521 | NA | Thoc6 | THO complex 6 homolog (Drosophila) | 386612 | ENSMUSG00000041319 |
| 145 | 17250141 | NA | Zfp39 | zinc finger protein 39 | 22698 | ENSMUSG00000037001 |
| 146 | 17485815 | NA | Syt5 | synaptotagmin V | 53420 | ENSMUSG00000004961 |
| 147 | 17519718 | NA | Mto1 | mitochondrial translation optimization 1 homolog (S. cerevisiae) | 68291 | ENSMUSG00000032342 |
| 148 | 17266322 | NA | Eral1 | Era (G-protein)-like 1 (E. coli) | 57837 | ENSMUSG00000020832 |
| 149 | 17488134 | NA | Rab4b | RAB4B, member RAS oncogene family | 19342 | ENSMUSG00000053291 |
| 150 | 17521422 | NA | Hyal2 | hyaluronoglucosaminidase 2 | 15587 | ENSMUSG00000010047 |
| 151 | 17317208 | NA | Derl1 | Der1-like domain family, member 1 | 67819 | ENSMUSG00000022365 |
| 152 | 17501544 | NA | Npy1r | neuropeptide Y receptor Y1 | 18166 | ENSMUSG00000036437 |
| 153 | 17376272 | NA | Nop56 | NOP56 ribonucleoprotein homolog (yeast) | 67134 | ENSMUSG00000027405 |
| 154 | 17468195 | NA | Stambp | STAM binding protein | 70527 | ENSMUSG00000006906 |
| 155 | 17408684 | NA | Dclre1b | DNA cross-link repair 1B, PSO2 homolog (S. cerevisiae) | 140917 | ENSMUSG00000027845 |
| 156 | 17443047 | NA | Caln1 | calneuron 1 | 140904 | ENSMUSG00000060371 |
| 157 | 17436077 | NA | Nrbp1 | nuclear receptor binding protein 1 | 192292 | ENSMUSG00000029148 |
| 158 | 17342015 | NA | Tbl3 | transducin (beta)-like 3 | 213773 | ENSMUSG00000040688 |
| 159 | 17543988 | NA | Taf9b | TAF9B RNA polymerase II, TATA box binding protein (TBP)-associated factor | 407786 | ENSMUSG00000047242 |
| 160 | 17509721 | NA | Tufm | Tu translation elongation factor, mitochondrial | 233870 | ENSMUSG00000073838 |
| 161 | 17459676 | NA | Retsat | retinol saturase (all trans retinol 13,14 reductase) | 67442 | ENSMUSG00000056666 |
| 162 | 17337269 | NA | Nrm | nurim (nuclear envelope membrane protein) | 106582 | ENSMUSG00000059791 |
| 163 | 17361463 | NA | Rab1b | RAB1B, member RAS oncogene family | 76308 | ENSMUSG00000024870 |
| 164 | 17279858 | NA | Fkbp1b | FK506 binding protein 1b | 14226 | ENSMUSG00000020635 |
| 165 | 17488463 | NA | Med29 | mediator complex subunit 29 | 67224 | ENSMUSG00000003444 |
| 166 | 17391270 | NA | Kcnip3 | Kv channel interacting protein 3, calsenilin | 56461 | ENSMUSG00000079056 |
| 167 | 17324576 | NA | Hrasls | HRAS-like suppressor | 27281 | ENSMUSG00000022525 |
| 168 | 17217580 | NA | Arl8a | ADP-ribosylation factor-like 8A | 68724 | ENSMUSG00000026426 |
| 169 | 17460099 | NA | Vax2 | ventral anterior homeobox containing gene 2 | 24113 | ENSMUSG00000034777 |
| 170 | 17266489 | NA | Tmem97 | transmembrane protein 97 | 69071 | ENSMUSG00000037278 |
| 171 | 17365369 | NA | Cuedc2 | CUE domain containing 2 | 67116 | ENSMUSG00000036748 |
| 172 | 17299353 | NA | Ktn1 | kinectin 1 | 16709 | ENSMUSG00000021843 |
| 173 | 17506854 | NA | Tsnax | translin-associated factor X | 53424 | ENSMUSG00000056820 |
| 174 | 17512479 | NA | Acd | adrenocortical dysplasia | 497652 | ENSMUSG00000038000 |
| 175 | 17288716 | NA | Glrx | glutaredoxin | 93692 | ENSMUSG00000021591 |
| 176 | 17540050 | NA | Ebp | phenylalkylamine Ca2+ antagonist (emopamil) binding protein | 13595 | ENSMUSG00000031168 |
| 177 | 17429495 | NA | Nfyc | nuclear transcription factor-Y gamma | 18046 | ENSMUSG00000032897 |
| 178 | 17361855 | NA | Pola2 | polymerase (DNA directed), alpha 2 | 18969 | ENSMUSG00000024833 |
| 179 | 17512463 | NA | Atp6v0d1 | ATPase, H+ transporting, lysosomal V0 subunit D1 | 11972 | ENSMUSG00000013160 |
| 180 | 17512466 | NA | Atp6v0d1 | ATPase, H+ transporting, lysosomal V0 subunit D1 | 11972 | ENSMUSG00000013160 |
| 181 | 17230945 | NA | Smyd2 | SET and MYND domain containing 2 | 226830 | ENSMUSG00000026603 |
| 182 | 17313008 | NA | Cby1 | chibby homolog 1 (Drosophila) | 73739 | ENSMUSG00000022428 |
| 183 | 17251607 | NA | Trappc1 | trafficking protein particle complex 1 | 245828 | ENSMUSG00000049299 |
| 184 | 17521448 | NA | Hyal3 | hyaluronoglucosaminidase 3 | 109685 | ENSMUSG00000036091 |
| 185 | 17535434 | NA | Nsdhl | NAD(P) dependent steroid dehydrogenase-like | 18194 | ENSMUSG00000031349 |
| 186 | 17299750 | NA | Tox4 | TOX high mobility group box family member 4 | 268741 | ENSMUSG00000016831 |
| 187 | 17468113 | NA | Ino80b | INO80 complex subunit B | 70020 | ENSMUSG00000030034 |
| 188 | 17502390 | NA | Rab8a | RAB8A, member RAS oncogene family | 17274 | ENSMUSG00000003037 |
| 189 | 17503023 | NA | Asf1b | ASF1 anti-silencing function 1 homolog B (S. cerevisiae) | 66929 | ENSMUSG00000005470 |
| 190 | 17258457 | NA | Sap30bp | SAP30 binding protein | 57230 | ENSMUSG00000020755 |
| 191 | 17328829 | NA | Slc25a1 | solute carrier family 25 (mitochondrial carrier, citrate transporter), member 1 | 13358 | ENSMUSG00000003528 |
| 192 | 17454256 | NA | Taf6 | TAF6 RNA polymerase II, TATA box binding protein (TBP)-associated factor | 21343 | ENSMUSG00000036980 |
| 193 | 17432967 | NA | Ubiad1 | UbiA prenyltransferase domain containing 1 | 71707 | ENSMUSG00000047719 |
| 194 | 17526273 | NA | Trappc4 | trafficking protein particle complex 4 | 60409 | ENSMUSG00000032112 |
| 195 | 17526271 | NA | Trappc4 | trafficking protein particle complex 4 | 60409 | ENSMUSG00000032112 |
| 196 | 17526272 | NA | Trappc4 | trafficking protein particle complex 4 | 60409 | ENSMUSG00000032112 |
| 197 | 17465696 | NA | Slc35b4 | solute carrier family 35, member B4 | 58246 | ENSMUSG00000018999 |
| 198 | 17231118 | NA | Rcor3 | REST corepressor 3 | 214742 | ENSMUSG00000037395 |
| 199 | 17379554 | NA | Zswim1 | zinc finger, SWIM domain containing 1 | 71971 | ENSMUSG00000017764 |
| 200 | 17298407 | NA | Bap1 | Brca1 associated protein 1 | 104416 | ENSMUSG00000021901 |
| 201 | 17318877 | NA | Txn2 | thioredoxin 2 | 56551 | ENSMUSG00000005354 |
| 202 | 17453809 | NA | Ap1s1 | adaptor protein complex AP-1, sigma 1 | 11769 | ENSMUSG00000004849 |
| 203 | 17329163 | NA | Camk2n2 | calcium/calmodulin-dependent protein kinase II inhibitor 2 | 73047 | ENSMUSG00000051146 |
| 204 | 17405174 | NA | Cog6 | component of oligomeric golgi complex 6 | 67542 | ENSMUSG00000027742 |
| 205 | 17382914 | NA | Dnlz | DNL-type zinc finger | 52838 | ENSMUSG00000075467 |
| 206 | 17273280 | NA | Stra13 | stimulated by retinoic acid 13 | 20892 | ENSMUSG00000025144 |
| 207 | 17301342 | NA | Ints9 | integrator complex subunit 9 | 210925 | ENSMUSG00000021975 |
| 208 | 17241409 | NA | Srgn | serglycin | 19073 | ENSMUSG00000020077 |
| 209 | 17322200 | NA | Aaas | achalasia, adrenocortical insufficiency, alacrimia | 223921 | ENSMUSG00000036678 |
| 210 | 17393357 | NA | Eif6 | eukaryotic translation initiation factor 6 | 16418 | ENSMUSG00000027613 |
| 211 | 17412593 | NA | Srsf12 | serine/arginine-rich splicing factor 12 | 272009 | ENSMUSG00000054679 |
| 212 | 17451816 | NA | Hspb8 | heat shock protein 8 | 80888 | ENSMUSG00000041548 |
| 213 | 17213990 | NA | Atic | 5-aminoimidazole-4-carboxamide ribonucleotide formyltransferase/IMP cyclohydrolase | 108147 | ENSMUSG00000026192 |
| 214 | 17504160 | NA | Polr2c | polymerase (RNA) II (DNA directed) polypeptide C | 20021 | ENSMUSG00000031783 |
| 215 | 17455093 | NA | Zkscan14 | zinc finger with KRAB and SCAN domains 14 | 67235 | ENSMUSG00000029627 |
| 216 | 17488544 | NA | Nfkbib | nuclear factor of kappa light polypeptide gene enhancer in B cells inhibitor, beta | 18036 | ENSMUSG00000030595 |
| 217 | 17508691 | NA | Rbpms | RNA binding protein gene with multiple splicing | 19663 | ENSMUSG00000031586 |
| 218 | 17222001 | NA | Prim2 | DNA primase, p58 subunit | 19076 | ENSMUSG00000026134 |
| 219 | 17232649 | NA | Fyn | Fyn proto-oncogene | 14360 | ENSMUSG00000019843 |
| 220 | 17328810 | NA | Dgcr14 | DiGeorge syndrome critical region gene 14 | 27886 | ENSMUSG00000003527 |
| 221 | 17474534 | NA | Opa3 | optic atrophy 3 | 403187 | ENSMUSG00000052214 |
| 222 | 17395844 | NA | Stmn3 | stathmin-like 3 | 20262 | ENSMUSG00000027581 |
| 223 | 17229466 | NA | Hsd17b7 | hydroxysteroid (17-beta) dehydrogenase 7 | 15490 | ENSMUSG00000026675 |
| 224 | 17219286 | NA | Dedd | death effector domain-containing | 21945 | ENSMUSG00000013973 |
| 225 | 17463150 | NA | Vamp1 | vesicle-associated membrane protein 1 | 22317 | ENSMUSG00000030337 |
| 226 | 17306861 | NA | Dhrs1 | dehydrogenase/reductase (SDR family) member 1 | 52585 | ENSMUSG00000002332 |
| 227 | 17306864 | NA | Dhrs1 | dehydrogenase/reductase (SDR family) member 1 | 52585 | ENSMUSG00000002332 |
| 228 | 17306860 | NA | Dhrs1 | dehydrogenase/reductase (SDR family) member 1 | 52585 | ENSMUSG00000002332 |
| 229 | 17306856 | NA | Dhrs1 | dehydrogenase/reductase (SDR family) member 1 | 52585 | ENSMUSG00000002332 |
| 230 | 17306865 | NA | Dhrs1 | dehydrogenase/reductase (SDR family) member 1 | 52585 | ENSMUSG00000002332 |
| 231 | 17418571 | NA | Trappc3 | trafficking protein particle complex 3 | 27096 | ENSMUSG00000028847 |
| 232 | 17359945 | NA | Gbf1 | golgi-specific brefeldin A-resistance factor 1 | 107338 | ENSMUSG00000025224 |
| 233 | 17334495 | NA | Nme3 | NME/NM23 nucleoside diphosphate kinase 3 | 79059 | ENSMUSG00000073435 |
| 234 | 17338043 | NA | Yipf3 | Yip1 domain family, member 3 | 28064 | ENSMUSG00000071074 |
| 235 | 17396024 | NA | Stmn2 | stathmin-like 2 | 20257 | ENSMUSG00000027500 |
| 236 | 17535752 | NA | Emd | emerin | 13726 | ENSMUSG00000001964 |
| 237 | 17383588 | NA | Ccbl1 | cysteine conjugate-beta lyase 1 | 70266 | ENSMUSG00000039648 |
| 238 | 17313199 | NA | Adsl | adenylosuccinate lyase | 11564 | ENSMUSG00000022407 |
| 239 | 17232215 | NA | Moxd1 | monooxygenase, DBH-like 1 | 59012 | ENSMUSG00000020000 |
| 240 | 17418447 | NA | Meaf6 | MYST/Esa1-associated factor 6 | 70088 | ENSMUSG00000028863 |
| 241 | 17356202 | NA | Pold4 | polymerase (DNA-directed), delta 4 | 69745 | ENSMUSG00000024854 |
| 242 | 17400638 | NA | Pex11b | peroxisomal biogenesis factor 11 beta | 18632 | ENSMUSG00000028102 |
| 243 | 17240123 | NA | Clvs2 | clavesin 2 | 215890 | ENSMUSG00000019785 |
| 244 | 17421972 | NA | Errfi1 | ERBB receptor feedback inhibitor 1 | 74155 | ENSMUSG00000028967 |
| 245 | 17462975 | NA | Mlf2 | myeloid leukemia factor 2 | 30853 | ENSMUSG00000030120 |

  
  

| **Database:cellular component      &nbspName:cell      &nbspID:GO:0005623** | | | | | | |
| --- | --- | --- | --- | --- | --- | --- |
| C=12714; O=304; E=245.70; R=1.24; rawP=2.27e-10; adjP=6.58e-09 | | | | | | |
| Index | UserID | Value | Gene Symbol | Gene Name | EntrezGene | Ensembl |
| 1 | 17476273 | NA | Zfp382 | zinc finger protein 382 | 233060 | ENSMUSG00000074220 |
| 2 | 17413221 | NA | Unc13b | unc-13 homolog B (C. elegans) | 22249 | ENSMUSG00000028456 |
| 3 | 17474547 | NA | Rtn2 | reticulon 2 (Z-band associated protein) | 20167 | ENSMUSG00000030401 |
| 4 | 17512740 | NA | Nob1 | NIN1/RPN12 binding protein 1 homolog (S. cerevisiae) | 67619 | ENSMUSG00000003848 |
| 5 | 17503910 | NA | Ogfod1 | 2-oxoglutarate and iron-dependent oxygenase domain containing 1 | 270086 | ENSMUSG00000033009 |
| 6 | 17368171 | NA | Bmyc | brain expressed myelocytomatosis oncogene | 107771 | ENSMUSG00000049086 |
| 7 | 17350134 | NA | Pou4f3 | POU domain, class 4, transcription factor 3 | 18998 | ENSMUSG00000024497 |
| 8 | 17443181 | NA | Dnajc30 | DnaJ (Hsp40) homolog, subfamily C, member 30 | 66114 | ENSMUSG00000061118 |
| 9 | 17319324 | NA | Dnalc4 | dynein, axonemal, light chain 4 | 54152 | ENSMUSG00000022420 |
| 10 | 17512103 | NA | Got2 | glutamate oxaloacetate transaminase 2, mitochondrial | 14719 | ENSMUSG00000031672 |
| 11 | 17336829 | NA | Lsm2 | LSM2 homolog, U6 small nuclear RNA associated (S. cerevisiae) | 27756 | ENSMUSG00000007050 |
| 12 | 17288454 | NA | Irx4 | Iroquois related homeobox 4 (Drosophila) | 50916 | ENSMUSG00000021604 |
| 13 | 17467996 | NA | Mrpl19 | mitochondrial ribosomal protein L19 | 56284 | ENSMUSG00000030045 |
| 14 | 17306758 | NA | Tm9sf1 | transmembrane 9 superfamily member 1 | 74140 | ENSMUSG00000002320 |
| 15 | 17504712 | NA | Exoc3l | exocyst complex component 3-like | 277978 | ENSMUSG00000043251 |
| 16 | 17321467 | NA | Tuba1b | tubulin, alpha 1B | 22143 | ENSMUSG00000023004 |
| 17 | 17541597 | NA | Frmd7 | FERM domain containing 7 | 385354 | ENSMUSG00000036131 |
| 18 | 17457603 | NA | Tas2r108 | taste receptor, type 2, member 108 | 57253 | ENSMUSG00000037140 |
| 19 | 17379187 | NA | Ift52 | intraflagellar transport 52 | 245866 | ENSMUSG00000017858 |
| 20 | 17246284 | NA | Suox | sulfite oxidase | 211389 | ENSMUSG00000049858 |
| 21 | 17437611 | NA | Pgm1 | phosphoglucomutase 1 | 66681 | ENSMUSG00000029171 |
| 22 | 17287579 | NA | Zfp346 | zinc finger protein 346 | 26919 | ENSMUSG00000021481 |
| 23 | 17224577 | NA | Resp18 | regulated endocrine-specific protein 18 | 19711 | ENSMUSG00000033061 |
| 24 | 17297750 | NA | Ppif | peptidylprolyl isomerase F (cyclophilin F) | 105675 | ENSMUSG00000021868 |
| 25 | 17520177 | NA | Mthfs | 5, 10-methenyltetrahydrofolate synthetase | 107885 | ENSMUSG00000066442 |
| 26 | 17480102 | NA | Sytl2 | synaptotagmin-like 2 | 83671 | ENSMUSG00000030616 |
| 27 | 17521143 | NA | Wdr82 | WD repeat domain containing 82 | 77305 | ENSMUSG00000020257 |
| 28 | 17312944 | NA | Polr2f | polymerase (RNA) II (DNA directed) polypeptide F | 69833 | ENSMUSG00000033020 |
| 29 | 17312939 | NA | Polr2f | polymerase (RNA) II (DNA directed) polypeptide F | 69833 | ENSMUSG00000033020 |
| 30 | 17312941 | NA | Polr2f | polymerase (RNA) II (DNA directed) polypeptide F | 69833 | ENSMUSG00000033020 |
| 31 | 17329151 | NA | Alg3 | asparagine-linked glycosylation 3 (alpha-1,3-mannosyltransferase) | 208624 | ENSMUSG00000033809 |
| 32 | 17500391 | NA | Rnf122 | ring finger protein 122 | 68867 | ENSMUSG00000039328 |
| 33 | 17400222 | NA | Vps72 | vacuolar protein sorting 72 (yeast) | 21427 | ENSMUSG00000008958 |
| 34 | 17499396 | NA | Fbxo25 | F-box protein 25 | 66822 | ENSMUSG00000038365 |
| 35 | 17324664 | NA | Dlg1 | discs, large homolog 1 (Drosophila) | 13383 | ENSMUSG00000022770 |
| 36 | 17396369 | NA | Nceh1 | arylacetamide deacetylase-like 1 | 320024 | ENSMUSG00000027698 |
| 37 | 17314556 | NA | Slc48a1 | solute carrier family 48 (heme transporter), member 1 | 67739 | ENSMUSG00000081534 |
| 38 | 17246209 | NA | Rpl41 | ribosomal protein L41 | 67945 | ENSMUSG00000093674 |
| 39 | 17529231 | NA | Phip | pleckstrin homology domain interacting protein | 83946 | ENSMUSG00000032253 |
| 40 | 17483220 | NA | Cdipt | CDP-diacylglycerol--inositol 3-phosphatidyltransferase (phosphatidylinositol synthase) | 52858 | ENSMUSG00000030682 |
| 41 | 17361975 | NA | Snx15 | sorting nexin 15 | 69024 | ENSMUSG00000024787 |
| 42 | 17424319 | NA | Sigmar1 | sigma non-opioid intracellular receptor 1 | 18391 | ENSMUSG00000036078 |
| 43 | 17260221 | NA | Pold2 | polymerase (DNA directed), delta 2, regulatory subunit | 18972 | ENSMUSG00000020471 |
| 44 | 17446580 | NA | Shh | sonic hedgehog | 20423 | ENSMUSG00000002633 |
| 45 | 17281971 | NA | Sgpp1 | sphingosine-1-phosphate phosphatase 1 | 81535 | ENSMUSG00000021054 |
| 46 | 17238549 | NA | Wibg | within bgcn homolog (Drosophila) | 78428 | ENSMUSG00000064030 |
| 47 | 17212355 | NA | Nck2 | non-catalytic region of tyrosine kinase adaptor protein 2 | 17974 | ENSMUSG00000066877 |
| 48 | 17307695 | NA | Msra | methionine sulfoxide reductase A | 110265 | ENSMUSG00000054733 |
| 49 | 17303496 | NA | Fezf2 | Fez family zinc finger 2 | 54713 | ENSMUSG00000021743 |
| 50 | 17345519 | NA | Rrp36 | ribosomal RNA processing 36 homolog (S. cerevisiae) | 224823 | ENSMUSG00000023971 |
| 51 | 17211335 | NA | Tfap2d | transcription factor AP-2, delta | 226896 | ENSMUSG00000042596 |
| 52 | 17506631 | NA | Tubb3 | tubulin, beta 3 class III | 22152 | ENSMUSG00000062380 |
| 53 | 17235584 | NA | Dapk3 | death-associated protein kinase 3 | 13144 | ENSMUSG00000034974 |
| 54 | 17338670 | NA | Fsd1 | fibronectin type 3 and SPRY domain-containing protein | 240121 | ENSMUSG00000011589 |
| 55 | 17300411 | NA | Thtpa | thiamine triphosphatase | 105663 | ENSMUSG00000045691 |
| 56 | 17460879 | NA | Hdac11 | histone deacetylase 11 | 232232 | ENSMUSG00000034245 |
| 57 | 17444100 | NA | Chst12 | carbohydrate sulfotransferase 12 | 59031 | ENSMUSG00000036599 |
| 58 | 17359994 | NA | Fbxl15 | F-box and leucine-rich repeat protein 15 | 68431 | ENSMUSG00000025226 |
| 59 | 17217666 | NA | Tmem9 | transmembrane protein 9 | 66241 | ENSMUSG00000026411 |
| 60 | 17484068 | NA | Lhpp | phospholysine phosphohistidine inorganic pyrophosphate phosphatase | 76429 | ENSMUSG00000030946 |
| 61 | 17497366 | NA | Ebf3 | early B cell factor 3 | 13593 | ENSMUSG00000010476 |
| 62 | 17288616 | NA | Tppp | tubulin polymerization promoting protein | 72948 | ENSMUSG00000021573 |
| 63 | 17446322 | NA | Prkag2 | protein kinase, AMP-activated, gamma 2 non-catalytic subunit | 108099 | ENSMUSG00000028944 |
| 64 | 17452552 | NA | Rhof | ras homolog gene family, member f | 23912 | ENSMUSG00000029449 |
| 65 | 17407764 | NA | Prune | prune homolog (Drosophila) | 229589 | ENSMUSG00000015711 |
| 66 | 17524523 | NA | Eif3g | eukaryotic translation initiation factor 3, subunit G | 53356 | ENSMUSG00000070319 |
| 67 | 17268995 | NA | Krt222 | keratin 222 | 268481 | ENSMUSG00000035849 |
| 68 | 17505367 | NA | Txnl4b | thioredoxin-like 4B | 234723 | ENSMUSG00000031723 |
| 69 | 17540982 | NA | Sept6 | septin 6 | 56526 | ENSMUSG00000050379 |
| 70 | 17235268 | NA | Ndufs7 | NADH dehydrogenase (ubiquinone) Fe-S protein 7 | 75406 | ENSMUSG00000020153 |
| 71 | 17447726 | NA | Hs3st1 | heparan sulfate (glucosamine) 3-O-sulfotransferase 1 | 15476 | ENSMUSG00000051022 |
| 72 | 17454416 | NA | Zfand2a | zinc finger, AN1-type domain 2A | 100494 | ENSMUSG00000053581 |
| 73 | 17422117 | NA | Acot7 | acyl-CoA thioesterase 7 | 70025 | ENSMUSG00000028937 |
| 74 | 17536264 | NA | Pcyt1b | phosphate cytidylyltransferase 1, choline, beta isoform | 236899 | ENSMUSG00000035246 |
| 75 | 17357213 | NA | Zbtb3 | zinc finger and BTB domain containing 3 | 75291 | ENSMUSG00000071661 |
| 76 | 17453106 | NA | Zfp11 | zinc finger protein 11 | 22648 | ENSMUSG00000051034 |
| 77 | 17335357 | NA | Lhfpl5 | lipoma HMGIC fusion partner-like 5 | 328789 | ENSMUSG00000062252 |
| 78 | 17497626 | NA | Sprn | shadow of prion protein | 212518 | ENSMUSG00000045733 |
| 79 | 17315558 | NA | Copz1 | coatomer protein complex, subunit zeta 1 | 56447 | ENSMUSG00000060992 |
| 80 | 17361988 | NA | Arl2 | ADP-ribosylation factor-like 2 | 56327 | ENSMUSG00000024944 |
| 81 | 17502583 | NA | Mcm5 | minichromosome maintenance deficient 5, cell division cycle 46 (S. cerevisiae) | 17218 | ENSMUSG00000005410 |
| 82 | 17485815 | NA | Syt5 | synaptotagmin V | 53420 | ENSMUSG00000004961 |
| 83 | 17519718 | NA | Mto1 | mitochondrial translation optimization 1 homolog (S. cerevisiae) | 68291 | ENSMUSG00000032342 |
| 84 | 17488134 | NA | Rab4b | RAB4B, member RAS oncogene family | 19342 | ENSMUSG00000053291 |
| 85 | 17344453 | NA | Ppp1r18 | protein phosphatase 1, regulatory subunit 18 | 76448 | ENSMUSG00000034595 |
| 86 | 17317208 | NA | Derl1 | Der1-like domain family, member 1 | 67819 | ENSMUSG00000022365 |
| 87 | 17501544 | NA | Npy1r | neuropeptide Y receptor Y1 | 18166 | ENSMUSG00000036437 |
| 88 | 17428858 | NA | Rnf220 | ring finger protein 220 | 66743 | ENSMUSG00000028677 |
| 89 | 17428857 | NA | Rnf220 | ring finger protein 220 | 66743 | ENSMUSG00000028677 |
| 90 | 17408684 | NA | Dclre1b | DNA cross-link repair 1B, PSO2 homolog (S. cerevisiae) | 140917 | ENSMUSG00000027845 |
| 91 | 17443047 | NA | Caln1 | calneuron 1 | 140904 | ENSMUSG00000060371 |
| 92 | 17436077 | NA | Nrbp1 | nuclear receptor binding protein 1 | 192292 | ENSMUSG00000029148 |
| 93 | 17252170 | NA | Rnf167 | ring finger protein 167 | 70510 | ENSMUSG00000040746 |
| 94 | 17543988 | NA | Taf9b | TAF9B RNA polymerase II, TATA box binding protein (TBP)-associated factor | 407786 | ENSMUSG00000047242 |
| 95 | 17459676 | NA | Retsat | retinol saturase (all trans retinol 13,14 reductase) | 67442 | ENSMUSG00000056666 |
| 96 | 17337269 | NA | Nrm | nurim (nuclear envelope membrane protein) | 106582 | ENSMUSG00000059791 |
| 97 | 17488463 | NA | Med29 | mediator complex subunit 29 | 67224 | ENSMUSG00000003444 |
| 98 | 17391270 | NA | Kcnip3 | Kv channel interacting protein 3, calsenilin | 56461 | ENSMUSG00000079056 |
| 99 | 17324576 | NA | Hrasls | HRAS-like suppressor | 27281 | ENSMUSG00000022525 |
| 100 | 17412579 | NA | Gabrr1 | gamma-aminobutyric acid (GABA) C receptor, subunit rho 1 | 14408 | ENSMUSG00000028280 |
| 101 | 17348933 | NA | Mapre2 | microtubule-associated protein, RP/EB family, member 2 | 212307 | ENSMUSG00000024277 |
| 102 | 17266489 | NA | Tmem97 | transmembrane protein 97 | 69071 | ENSMUSG00000037278 |
| 103 | 17299353 | NA | Ktn1 | kinectin 1 | 16709 | ENSMUSG00000021843 |
| 104 | 17506854 | NA | Tsnax | translin-associated factor X | 53424 | ENSMUSG00000056820 |
| 105 | 17413573 | NA | Grhpr | glyoxylate reductase/hydroxypyruvate reductase | 76238 | ENSMUSG00000035637 |
| 106 | 17288716 | NA | Glrx | glutaredoxin | 93692 | ENSMUSG00000021591 |
| 107 | 17429495 | NA | Nfyc | nuclear transcription factor-Y gamma | 18046 | ENSMUSG00000032897 |
| 108 | 17361855 | NA | Pola2 | polymerase (DNA directed), alpha 2 | 18969 | ENSMUSG00000024833 |
| 109 | 17527532 | NA | Mpi | mannose phosphate isomerase | 110119 | ENSMUSG00000032306 |
| 110 | 17512463 | NA | Atp6v0d1 | ATPase, H+ transporting, lysosomal V0 subunit D1 | 11972 | ENSMUSG00000013160 |
| 111 | 17512466 | NA | Atp6v0d1 | ATPase, H+ transporting, lysosomal V0 subunit D1 | 11972 | ENSMUSG00000013160 |
| 112 | 17252635 | NA | Shpk | sedoheptulokinase | 74637 | ENSMUSG00000005951 |
| 113 | 17313008 | NA | Cby1 | chibby homolog 1 (Drosophila) | 73739 | ENSMUSG00000022428 |
| 114 | 17535434 | NA | Nsdhl | NAD(P) dependent steroid dehydrogenase-like | 18194 | ENSMUSG00000031349 |
| 115 | 17503023 | NA | Asf1b | ASF1 anti-silencing function 1 homolog B (S. cerevisiae) | 66929 | ENSMUSG00000005470 |
| 116 | 17328829 | NA | Slc25a1 | solute carrier family 25 (mitochondrial carrier, citrate transporter), member 1 | 13358 | ENSMUSG00000003528 |
| 117 | 17454256 | NA | Taf6 | TAF6 RNA polymerase II, TATA box binding protein (TBP)-associated factor | 21343 | ENSMUSG00000036980 |
| 118 | 17432967 | NA | Ubiad1 | UbiA prenyltransferase domain containing 1 | 71707 | ENSMUSG00000047719 |
| 119 | 17527666 | NA | Islr2 | immunoglobulin superfamily containing leucine-rich repeat 2 | 320563 | ENSMUSG00000051243 |
| 120 | 17279499 | NA | Crip2 | cysteine rich protein 2 | 68337 | ENSMUSG00000006356 |
| 121 | 17542220 | NA | Gabra3 | gamma-aminobutyric acid (GABA) A receptor, subunit alpha 3 | 14396 | ENSMUSG00000031343 |
| 122 | 17231118 | NA | Rcor3 | REST corepressor 3 | 214742 | ENSMUSG00000037395 |
| 123 | 17379554 | NA | Zswim1 | zinc finger, SWIM domain containing 1 | 71971 | ENSMUSG00000017764 |
| 124 | 17298407 | NA | Bap1 | Brca1 associated protein 1 | 104416 | ENSMUSG00000021901 |
| 125 | 17280897 | NA | Stxbp6 | syntaxin binding protein 6 (amisyn) | 217517 | ENSMUSG00000046314 |
| 126 | 17451443 | NA | Coro1c | coronin, actin binding protein 1C | 23790 | ENSMUSG00000004530 |
| 127 | 17273280 | NA | Stra13 | stimulated by retinoic acid 13 | 20892 | ENSMUSG00000025144 |
| 128 | 17301342 | NA | Ints9 | integrator complex subunit 9 | 210925 | ENSMUSG00000021975 |
| 129 | 17393357 | NA | Eif6 | eukaryotic translation initiation factor 6 | 16418 | ENSMUSG00000027613 |
| 130 | 17412593 | NA | Srsf12 | serine/arginine-rich splicing factor 12 | 272009 | ENSMUSG00000054679 |
| 131 | 17451816 | NA | Hspb8 | heat shock protein 8 | 80888 | ENSMUSG00000041548 |
| 132 | 17488544 | NA | Nfkbib | nuclear factor of kappa light polypeptide gene enhancer in B cells inhibitor, beta | 18036 | ENSMUSG00000030595 |
| 133 | 17504160 | NA | Polr2c | polymerase (RNA) II (DNA directed) polypeptide C | 20021 | ENSMUSG00000031783 |
| 134 | 17455093 | NA | Zkscan14 | zinc finger with KRAB and SCAN domains 14 | 67235 | ENSMUSG00000029627 |
| 135 | 17328810 | NA | Dgcr14 | DiGeorge syndrome critical region gene 14 | 27886 | ENSMUSG00000003527 |
| 136 | 17229466 | NA | Hsd17b7 | hydroxysteroid (17-beta) dehydrogenase 7 | 15490 | ENSMUSG00000026675 |
| 137 | 17242318 | NA | Gm7138 | predicted gene 7138 | 634517 | ENSMUSG00000095593 |
| 138 | 17463150 | NA | Vamp1 | vesicle-associated membrane protein 1 | 22317 | ENSMUSG00000030337 |
| 139 | 17306861 | NA | Dhrs1 | dehydrogenase/reductase (SDR family) member 1 | 52585 | ENSMUSG00000002332 |
| 140 | 17306864 | NA | Dhrs1 | dehydrogenase/reductase (SDR family) member 1 | 52585 | ENSMUSG00000002332 |
| 141 | 17306860 | NA | Dhrs1 | dehydrogenase/reductase (SDR family) member 1 | 52585 | ENSMUSG00000002332 |
| 142 | 17306856 | NA | Dhrs1 | dehydrogenase/reductase (SDR family) member 1 | 52585 | ENSMUSG00000002332 |
| 143 | 17306865 | NA | Dhrs1 | dehydrogenase/reductase (SDR family) member 1 | 52585 | ENSMUSG00000002332 |
| 144 | 17305182 | NA | Nrg3 | neuregulin 3 | 18183 | ENSMUSG00000041014 |
| 145 | 17418571 | NA | Trappc3 | trafficking protein particle complex 3 | 27096 | ENSMUSG00000028847 |
| 146 | 17318942 | NA | Pvalb | parvalbumin | 19293 | ENSMUSG00000005716 |
| 147 | 17419206 | NA | Pef1 | penta-EF hand domain containing 1 | 67898 | ENSMUSG00000028779 |
| 148 | 17535752 | NA | Emd | emerin | 13726 | ENSMUSG00000001964 |
| 149 | 17396024 | NA | Stmn2 | stathmin-like 2 | 20257 | ENSMUSG00000027500 |
| 150 | 17338043 | NA | Yipf3 | Yip1 domain family, member 3 | 28064 | ENSMUSG00000071074 |
| 151 | 17313199 | NA | Adsl | adenylosuccinate lyase | 11564 | ENSMUSG00000022407 |
| 152 | 17418447 | NA | Meaf6 | MYST/Esa1-associated factor 6 | 70088 | ENSMUSG00000028863 |
| 153 | 17232215 | NA | Moxd1 | monooxygenase, DBH-like 1 | 59012 | ENSMUSG00000020000 |
| 154 | 17356202 | NA | Pold4 | polymerase (DNA-directed), delta 4 | 69745 | ENSMUSG00000024854 |
| 155 | 17240123 | NA | Clvs2 | clavesin 2 | 215890 | ENSMUSG00000019785 |
| 156 | 17462975 | NA | Mlf2 | myeloid leukemia factor 2 | 30853 | ENSMUSG00000030120 |
| 157 | 17255719 | NA | Mrpl10 | mitochondrial ribosomal protein L10 | 107732 | ENSMUSG00000001445 |
| 158 | 17257962 | NA | Sstr2 | somatostatin receptor 2 | 20606 | ENSMUSG00000047904 |
| 159 | 17344794 | NA | Znrd1 | zinc ribbon domain containing, 1 | 66136 | ENSMUSG00000036315 |
| 160 | 17232843 | NA | Zbtb24 | zinc finger and BTB domain containing 24 | 268294 | ENSMUSG00000019826 |
| 161 | 17300261 | NA | Oxa1l | oxidase assembly 1-like | 69089 | ENSMUSG00000000959 |
| 162 | 17224540 | NA | Tuba4a | tubulin, alpha 4A | 22145 | ENSMUSG00000026202 |
| 163 | 17456308 | NA | Kcnd2 | potassium voltage-gated channel, Shal-related family, member 2 | 16508 | ENSMUSG00000060882 |
| 164 | 17517723 | NA | Rpp25 | ribonuclease P 25 subunit (human) | 102614 | ENSMUSG00000062309 |
| 165 | 17307134 | NA | Cryl1 | crystallin, lambda 1 | 68631 | ENSMUSG00000021947 |
| 166 | 17521652 | NA | Nicn1 | nicolin 1 | 66257 | ENSMUSG00000032606 |
| 167 | 17369862 | NA | Dpm2 | dolichol-phosphate (beta-D) mannosyltransferase 2 | 13481 | ENSMUSG00000026810 |
| 168 | 17322559 | NA | Hmox2 | heme oxygenase (decycling) 2 | 15369 | ENSMUSG00000004070 |
| 169 | 17537677 | NA | Drp2 | dystrophin related protein 2 | 13497 | ENSMUSG00000000223 |
| 170 | 17375833 | NA | Tmem127 | transmembrane protein 127 | 69470 | ENSMUSG00000034850 |
| 171 | 17457310 | NA | Chrm2 | cholinergic receptor, muscarinic 2, cardiac | 243764 | ENSMUSG00000045613 |
| 172 | 17538096 | NA | Rnf128 | ring finger protein 128 | 66889 | ENSMUSG00000031438 |
| 173 | 17288160 | NA | Cdk20 | cyclin-dependent kinase 20 | 105278 | ENSMUSG00000021483 |
| 174 | 17263594 | NA | Atpaf2 | ATP synthase mitochondrial F1 complex assembly factor 2 | 246782 | ENSMUSG00000042709 |
| 175 | 17448565 | NA | Gabrg1 | gamma-aminobutyric acid (GABA) A receptor, subunit gamma 1 | 14405 | ENSMUSG00000001260 |
| 176 | 17343617 | NA | Rab11b | RAB11B, member RAS oncogene family | 19326 | ENSMUSG00000077450 |
| 177 | 17447099 | NA | Ctbp1 | C-terminal binding protein 1 | 13016 | ENSMUSG00000037373 |
| 178 | 17447089 | NA | Ctbp1 | C-terminal binding protein 1 | 13016 | ENSMUSG00000037373 |
| 179 | 17447100 | NA | Ctbp1 | C-terminal binding protein 1 | 13016 | ENSMUSG00000037373 |
| 180 | 17447098 | NA | Ctbp1 | C-terminal binding protein 1 | 13016 | ENSMUSG00000037373 |
| 181 | 17344336 | NA | Tcf19 | transcription factor 19 | 106795 | ENSMUSG00000050410 |
| 182 | 17433602 | NA | Tprgl | transformation related protein 63 regulated like | 67808 | ENSMUSG00000029030 |
| 183 | 17265082 | NA | Eif5a | eukaryotic translation initiation factor 5A | 276770 | ENSMUSG00000078812 |
| 184 | 17233630 | NA | Psap | prosaposin | 19156 | ENSMUSG00000004207 |
| 185 | 17235368 | NA | Scamp4 | secretory carrier membrane protein 4 | 56214 | ENSMUSG00000078441 |
| 186 | 17369672 | NA | Ppapdc3 | phosphatidic acid phosphatase type 2 domain containing 3 | 227721 | ENSMUSG00000051373 |
| 187 | 17512434 | NA | Tppp3 | tubulin polymerization-promoting protein family member 3 | 67971 | ENSMUSG00000014846 |
| 188 | 17225499 | NA | Hes6 | hairy and enhancer of split 6 (Drosophila) | 55927 | ENSMUSG00000067071 |
| 189 | 17265175 | NA | 0610010K14Rik | RIKEN cDNA 0610010K14 gene | 104457 | ENSMUSG00000020831 |
| 190 | 17377583 | NA | Nsfl1c | NSFL1 (p97) cofactor (p47) | 386649 | ENSMUSG00000027455 |
| 191 | 17404628 | NA | Pex5l | peroxisomal biogenesis factor 5-like | 58869 | ENSMUSG00000027674 |
| 192 | 17517576 | NA | Hmg20a | high mobility group 20A | 66867 | ENSMUSG00000032329 |
| 193 | 17393225 | NA | Pigu | phosphatidylinositol glycan anchor biosynthesis, class U | 228812 | ENSMUSG00000038383 |
| 194 | 17404011 | NA | Hey1 | hairy/enhancer-of-split related with YRPW motif 1 | 15213 | ENSMUSG00000040289 |
| 195 | 17246850 | NA | Zmat5 | zinc finger, matrin type 5 | 67178 | ENSMUSG00000009076 |
| 196 | 17238846 | NA | Syne1 | synaptic nuclear envelope 1 | 64009 | ENSMUSG00000019769 ENSMUSG00000096054 |
| 197 | 17238906 | NA | Syne1 | synaptic nuclear envelope 1 | 64009 | ENSMUSG00000019769 ENSMUSG00000096054 |
| 198 | 17238890 | NA | Syne1 | synaptic nuclear envelope 1 | 64009 | ENSMUSG00000019769 ENSMUSG00000096054 |
| 199 | 17502191 | NA | Mrpl34 | mitochondrial ribosomal protein L34 | 94065 | ENSMUSG00000034880 |
| 200 | 17277370 | NA | Eif2b2 | eukaryotic translation initiation factor 2B, subunit 2 beta | 217715 | ENSMUSG00000004788 |
| 201 | 17316625 | NA | Ubr5 | ubiquitin protein ligase E3 component n-recognin 5 | 70790 | ENSMUSG00000037487 |
| 202 | 17512009 | NA | Csnk2a2 | casein kinase 2, alpha prime polypeptide | 13000 | ENSMUSG00000046707 |
| 203 | 17245709 | NA | Os9 | amplified in osteosarcoma | 216440 | ENSMUSG00000040462 |
| 204 | 17503816 | NA | Irx6 | Iroquois related homeobox 6 (Drosophila) | 64379 | ENSMUSG00000031738 |
| 205 | 17534615 | NA | Rab33a | RAB33A, member of RAS oncogene family | 19337 | ENSMUSG00000031104 |
| 206 | 17282649 | NA | Rps6kl1 | ribosomal protein S6 kinase-like 1 | 238323 | ENSMUSG00000019235 |
| 207 | 17482793 | NA | Cacng3 | calcium channel, voltage-dependent, gamma subunit 3 | 54376 | ENSMUSG00000066189 |
| 208 | 17365493 | NA | Obfc1 | oligonucleotide/oligosaccharide-binding fold containing 1 | 108689 | ENSMUSG00000042694 |
| 209 | 17538790 | NA | Huwe1 | HECT, UBA and WWE domain containing 1 | 59026 | ENSMUSG00000025261 |
| 210 | 17470879 | NA | Tpi1 | triosephosphate isomerase 1 | 21991 | ENSMUSG00000023456 |
| 211 | 17229644 | NA | Tomm40l | translocase of outer mitochondrial membrane 40 homolog-like (yeast) | 641376 | ENSMUSG00000005674 |
| 212 | 17522887 | NA | Golga4 | golgi autoantigen, golgin subfamily a, 4 | 54214 | ENSMUSG00000038708 |
| 213 | 17230408 | NA | Adck3 | aarF domain containing kinase 3 | 67426 | ENSMUSG00000026489 |
| 214 | 17532418 | NA | Ano10 | anoctamin 10 | 102566 | ENSMUSG00000037949 |
| 215 | 17352132 | NA | Txnl4a | thioredoxin-like 4A | 27366 | ENSMUSG00000057130 |
| 216 | 17418519 | NA | Oscp1 | organic solute carrier partner 1 | 230751 | ENSMUSG00000042616 |
| 217 | 17420582 | NA | Capzb | capping protein (actin filament) muscle Z-line, beta | 12345 | ENSMUSG00000028745 |
| 218 | 17273086 | NA | Nploc4 | nuclear protein localization 4 homolog (S. cerevisiae) | 217365 | ENSMUSG00000039703 |
| 219 | 17275955 | NA | Atl1 | atlastin GTPase 1 | 73991 | ENSMUSG00000021066 |
| 220 | 17431502 | NA | Lypla2 | lysophospholipase 2 | 26394 | ENSMUSG00000028670 |
| 221 | 17490785 | NA | Lin7b | lin-7 homolog B (C. elegans) | 22342 | ENSMUSG00000003872 |
| 222 | 17500275 | NA | Erlin2 | ER lipid raft associated 2 | 244373 | ENSMUSG00000031483 |
| 223 | 17256565 | NA | Tubg2 | tubulin, gamma 2 | 103768 | ENSMUSG00000045007 |
| 224 | 17463422 | NA | Nrip2 | nuclear receptor interacting protein 2 | 60345 | ENSMUSG00000001520 |
| 225 | 17299542 | NA | Tmem55b | transmembrane protein 55b | 219024 | ENSMUSG00000035953 |
| 226 | 17485194 | NA | Krtap5-4 | keratin associated protein 5-4 | 50775 | ENSMUSG00000045236 |
| 227 | 17253674 | NA | Poldip2 | polymerase (DNA-directed), delta interacting protein 2 | 67811 | ENSMUSG00000001100 |
| 228 | 17213153 | NA | Nif3l1 | Ngg1 interacting factor 3-like 1 (S. pombe) | 65102 | ENSMUSG00000026036 |
| 229 | 17321722 | NA | Tfcp2 | transcription factor CP2 | 21422 | ENSMUSG00000009733 |
| 230 | 17477670 | NA | Rras | Harvey rat sarcoma oncogene, subgroup R | 20130 | ENSMUSG00000038387 |
| 231 | 17405819 | NA | B3galnt1 | UDP-GalNAc:betaGlcNAc beta 1,3-galactosaminyltransferase, polypeptide 1 | 26879 | ENSMUSG00000043300 |
| 232 | 17498821 | NA | Snapc2 | small nuclear RNA activating complex, polypeptide 2 | 102209 | ENSMUSG00000011837 |
| 233 | 17328451 | NA | Mzt2 | mitotic spindle organizing protein 2 | 72083 | ENSMUSG00000022671 |
| 234 | 17379291 | NA | Ttpal | tocopherol (alpha) transfer protein-like | 76080 | ENSMUSG00000017679 |
| 235 | 17256618 | NA | Vps25 | vacuolar protein sorting 25 (yeast) | 28084 | ENSMUSG00000078656 |
| 236 | 17494637 | NA | Mrpl17 | mitochondrial ribosomal protein L17 | 27397 | ENSMUSG00000030879 |
| 237 | 17517532 | NA | Isl2 | insulin related protein 2 (islet 2) | 104360 | ENSMUSG00000032318 |
| 238 | 17513871 | NA | Chmp1a | charged multivesicular body protein 1A | 234852 | ENSMUSG00000000743 |
| 239 | 17269638 | NA | Rab5c | RAB5C, member RAS oncogene family | 19345 | ENSMUSG00000019173 |
| 240 | 17256549 | NA | Tubg1 | tubulin, gamma 1 | 103733 | ENSMUSG00000035198 |
| 241 | 17381128 | NA | Oprl1 | opioid receptor-like 1 | 18389 | ENSMUSG00000027584 |
| 242 | 17214293 | NA | Bcs1l | BCS1-like (yeast) | 66821 | ENSMUSG00000026172 |
| 243 | 17494081 | NA | Rhog | ras homolog gene family, member G | 56212 | ENSMUSG00000073982 |
| 244 | 17497957 | NA | Chid1 | chitinase domain containing 1 | 68038 | ENSMUSG00000025512 |
| 245 | 17257937 | NA | Kcnj2 | potassium inwardly-rectifying channel, subfamily J, member 2 | 16518 | ENSMUSG00000041695 |
| 246 | 17532045 | NA | Plcd1 | phospholipase C, delta 1 | 18799 | ENSMUSG00000010660 |
| 247 | 17274448 | NA | Cpsf3 | cleavage and polyadenylation specificity factor 3 | 54451 | ENSMUSG00000054309 |
| 248 | 17332333 | NA | Dscr3 | Down syndrome critical region gene 3 | 13185 | ENSMUSG00000022898 |
| 249 | 17332341 | NA | Dscr3 | Down syndrome critical region gene 3 | 13185 | ENSMUSG00000022898 |
| 250 | 17332343 | NA | Dscr3 | Down syndrome critical region gene 3 | 13185 | ENSMUSG00000022898 |
| 251 | 17332336 | NA | Dscr3 | Down syndrome critical region gene 3 | 13185 | ENSMUSG00000022898 |
| 252 | 17519868 | NA | Htr1b | 5-hydroxytryptamine (serotonin) receptor 1B | 15551 | ENSMUSG00000049511 |
| 253 | 17515170 | NA | Ilf3 | interleukin enhancer binding factor 3 | 16201 | ENSMUSG00000032178 |
| 254 | 17533055 | NA | Timm17b | translocase of inner mitochondrial membrane 17b | 21855 | ENSMUSG00000031158 |
| 255 | 17461968 | NA | Mkrn2 | makorin, ring finger protein, 2 | 67027 | ENSMUSG00000000439 |
| 256 | 17430853 | NA | Med18 | mediator of RNA polymerase II transcription, subunit 18 homolog (yeast) | 67219 | ENSMUSG00000066042 |
| 257 | 17442714 | NA | Bri3bp | Bri3 binding protein | 76809 | ENSMUSG00000037905 |
| 258 | 17521014 | NA | Acad11 | acyl-Coenzyme A dehydrogenase family, member 11 | 102632 | ENSMUSG00000090150 |
| 259 | 17370285 | NA | Mrrf | mitochondrial ribosome recycling factor | 67871 | ENSMUSG00000026887 |
| 260 | 17229948 | NA | Dusp23 | dual specificity phosphatase 23 | 68440 | ENSMUSG00000026544 |
| 261 | 17511296 | NA | Wdr83 | WD repeat domain containing 83 | 67836 | ENSMUSG00000005150 |
| 262 | 17539434 | NA | Ctps2 | cytidine 5'-triphosphate synthase 2 | 55936 | ENSMUSG00000031360 |
| 263 | 17250141 | NA | Zfp39 | zinc finger protein 39 | 22698 | ENSMUSG00000037001 |
| 264 | 17341521 | NA | Thoc6 | THO complex 6 homolog (Drosophila) | 386612 | ENSMUSG00000041319 |
| 265 | 17266322 | NA | Eral1 | Era (G-protein)-like 1 (E. coli) | 57837 | ENSMUSG00000020832 |
| 266 | 17521422 | NA | Hyal2 | hyaluronoglucosaminidase 2 | 15587 | ENSMUSG00000010047 |
| 267 | 17540589 | NA | Klhl13 | kelch-like 13 (Drosophila) | 67455 | ENSMUSG00000036782 |
| 268 | 17376272 | NA | Nop56 | NOP56 ribonucleoprotein homolog (yeast) | 67134 | ENSMUSG00000027405 |
| 269 | 17468195 | NA | Stambp | STAM binding protein | 70527 | ENSMUSG00000006906 |
| 270 | 17342015 | NA | Tbl3 | transducin (beta)-like 3 | 213773 | ENSMUSG00000040688 |
| 271 | 17509721 | NA | Tufm | Tu translation elongation factor, mitochondrial | 233870 | ENSMUSG00000073838 |
| 272 | 17342617 | NA | Arhgdig | Rho GDP dissociation inhibitor (GDI) gamma | 14570 | ENSMUSG00000073433 |
| 273 | 17361463 | NA | Rab1b | RAB1B, member RAS oncogene family | 76308 | ENSMUSG00000024870 |
| 274 | 17279858 | NA | Fkbp1b | FK506 binding protein 1b | 14226 | ENSMUSG00000020635 |
| 275 | 17217580 | NA | Arl8a | ADP-ribosylation factor-like 8A | 68724 | ENSMUSG00000026426 |
| 276 | 17460099 | NA | Vax2 | ventral anterior homeobox containing gene 2 | 24113 | ENSMUSG00000034777 |
| 277 | 17257060 | NA | Nmt1 | N-myristoyltransferase 1 | 18107 | ENSMUSG00000020936 |
| 278 | 17365369 | NA | Cuedc2 | CUE domain containing 2 | 67116 | ENSMUSG00000036748 |
| 279 | 17292107 | NA | Tbc1d7 | TBC1 domain family, member 7 | 67046 | ENSMUSG00000021368 |
| 280 | 17512479 | NA | Acd | adrenocortical dysplasia | 497652 | ENSMUSG00000038000 |
| 281 | 17540050 | NA | Ebp | phenylalkylamine Ca2+ antagonist (emopamil) binding protein | 13595 | ENSMUSG00000031168 |
| 282 | 17232426 | NA | Echdc1 | enoyl Coenzyme A hydratase domain containing 1 | 52665 | ENSMUSG00000019883 |
| 283 | 17212813 | NA | Mstn | myostatin | 17700 | ENSMUSG00000026100 |
| 284 | 17230945 | NA | Smyd2 | SET and MYND domain containing 2 | 226830 | ENSMUSG00000026603 |
| 285 | 17508554 | NA | Chrna6 | cholinergic receptor, nicotinic, alpha polypeptide 6 | 11440 | ENSMUSG00000031491 |
| 286 | 17251607 | NA | Trappc1 | trafficking protein particle complex 1 | 245828 | ENSMUSG00000049299 |
| 287 | 17521448 | NA | Hyal3 | hyaluronoglucosaminidase 3 | 109685 | ENSMUSG00000036091 |
| 288 | 17299750 | NA | Tox4 | TOX high mobility group box family member 4 | 268741 | ENSMUSG00000016831 |
| 289 | 17468113 | NA | Ino80b | INO80 complex subunit B | 70020 | ENSMUSG00000030034 |
| 290 | 17502390 | NA | Rab8a | RAB8A, member RAS oncogene family | 17274 | ENSMUSG00000003037 |
| 291 | 17347558 | NA | Cdkl4 | cyclin-dependent kinase-like 4 | 381113 | ENSMUSG00000033966 |
| 292 | 17283939 | NA | Wars | tryptophanyl-tRNA synthetase | 22375 | ENSMUSG00000021266 |
| 293 | 17283941 | NA | Wars | tryptophanyl-tRNA synthetase | 22375 | ENSMUSG00000021266 |
| 294 | 17283930 | NA | Wars | tryptophanyl-tRNA synthetase | 22375 | ENSMUSG00000021266 |
| 295 | 17283938 | NA | Wars | tryptophanyl-tRNA synthetase | 22375 | ENSMUSG00000021266 |
| 296 | 17258457 | NA | Sap30bp | SAP30 binding protein | 57230 | ENSMUSG00000020755 |
| 297 | 17512872 | NA | Calb2 | calbindin 2 | 12308 | ENSMUSG00000003657 |
| 298 | 17510365 | NA | Pgls | 6-phosphogluconolactonase | 66171 | ENSMUSG00000031807 |
| 299 | 17526273 | NA | Trappc4 | trafficking protein particle complex 4 | 60409 | ENSMUSG00000032112 |
| 300 | 17526271 | NA | Trappc4 | trafficking protein particle complex 4 | 60409 | ENSMUSG00000032112 |
| 301 | 17526272 | NA | Trappc4 | trafficking protein particle complex 4 | 60409 | ENSMUSG00000032112 |
| 302 | 17465696 | NA | Slc35b4 | solute carrier family 35, member B4 | 58246 | ENSMUSG00000018999 |
| 303 | 17318877 | NA | Txn2 | thioredoxin 2 | 56551 | ENSMUSG00000005354 |
| 304 | 17453809 | NA | Ap1s1 | adaptor protein complex AP-1, sigma 1 | 11769 | ENSMUSG00000004849 |
| 305 | 17329163 | NA | Camk2n2 | calcium/calmodulin-dependent protein kinase II inhibitor 2 | 73047 | ENSMUSG00000051146 |
| 306 | 17405174 | NA | Cog6 | component of oligomeric golgi complex 6 | 67542 | ENSMUSG00000027742 |
| 307 | 17382914 | NA | Dnlz | DNL-type zinc finger | 52838 | ENSMUSG00000075467 |
| 308 | 17221014 | NA | Cd34 | CD34 antigen | 12490 | ENSMUSG00000016494 |
| 309 | 17241409 | NA | Srgn | serglycin | 19073 | ENSMUSG00000020077 |
| 310 | 17322200 | NA | Aaas | achalasia, adrenocortical insufficiency, alacrimia | 223921 | ENSMUSG00000036678 |
| 311 | 17213990 | NA | Atic | 5-aminoimidazole-4-carboxamide ribonucleotide formyltransferase/IMP cyclohydrolase | 108147 | ENSMUSG00000026192 |
| 312 | 17508691 | NA | Rbpms | RNA binding protein gene with multiple splicing | 19663 | ENSMUSG00000031586 |
| 313 | 17464922 | NA | Gpr85 | G protein-coupled receptor 85 | 64450 | ENSMUSG00000048216 |
| 314 | 17222001 | NA | Prim2 | DNA primase, p58 subunit | 19076 | ENSMUSG00000026134 |
| 315 | 17232649 | NA | Fyn | Fyn proto-oncogene | 14360 | ENSMUSG00000019843 |
| 316 | 17474534 | NA | Opa3 | optic atrophy 3 | 403187 | ENSMUSG00000052214 |
| 317 | 17395844 | NA | Stmn3 | stathmin-like 3 | 20262 | ENSMUSG00000027581 |
| 318 | 17219286 | NA | Dedd | death effector domain-containing | 21945 | ENSMUSG00000013973 |
| 319 | 17241780 | NA | Ube2d1 | ubiquitin-conjugating enzyme E2D 1 | 216080 | ENSMUSG00000019927 |
| 320 | 17359945 | NA | Gbf1 | golgi-specific brefeldin A-resistance factor 1 | 107338 | ENSMUSG00000025224 |
| 321 | 17334495 | NA | Nme3 | NME/NM23 nucleoside diphosphate kinase 3 | 79059 | ENSMUSG00000073435 |
| 322 | 17383588 | NA | Ccbl1 | cysteine conjugate-beta lyase 1 | 70266 | ENSMUSG00000039648 |
| 323 | 17400638 | NA | Pex11b | peroxisomal biogenesis factor 11 beta | 18632 | ENSMUSG00000028102 |
| 324 | 17369952 | NA | Sh2d3c | SH2 domain containing 3C | 27387 | ENSMUSG00000059013 |
| 325 | 17421972 | NA | Errfi1 | ERBB receptor feedback inhibitor 1 | 74155 | ENSMUSG00000028967 |

  
  

| **Database:cellular component      &nbspName:cell part      &nbspID:GO:0044464** | | | | | | |
| --- | --- | --- | --- | --- | --- | --- |
| C=12713; O=304; E=245.68; R=1.24; rawP=2.24e-10; adjP=6.58e-09 | | | | | | |
| Index | UserID | Value | Gene Symbol | Gene Name | EntrezGene | Ensembl |
| 1 | 17476273 | NA | Zfp382 | zinc finger protein 382 | 233060 | ENSMUSG00000074220 |
| 2 | 17413221 | NA | Unc13b | unc-13 homolog B (C. elegans) | 22249 | ENSMUSG00000028456 |
| 3 | 17474547 | NA | Rtn2 | reticulon 2 (Z-band associated protein) | 20167 | ENSMUSG00000030401 |
| 4 | 17512740 | NA | Nob1 | NIN1/RPN12 binding protein 1 homolog (S. cerevisiae) | 67619 | ENSMUSG00000003848 |
| 5 | 17503910 | NA | Ogfod1 | 2-oxoglutarate and iron-dependent oxygenase domain containing 1 | 270086 | ENSMUSG00000033009 |
| 6 | 17368171 | NA | Bmyc | brain expressed myelocytomatosis oncogene | 107771 | ENSMUSG00000049086 |
| 7 | 17350134 | NA | Pou4f3 | POU domain, class 4, transcription factor 3 | 18998 | ENSMUSG00000024497 |
| 8 | 17443181 | NA | Dnajc30 | DnaJ (Hsp40) homolog, subfamily C, member 30 | 66114 | ENSMUSG00000061118 |
| 9 | 17319324 | NA | Dnalc4 | dynein, axonemal, light chain 4 | 54152 | ENSMUSG00000022420 |
| 10 | 17512103 | NA | Got2 | glutamate oxaloacetate transaminase 2, mitochondrial | 14719 | ENSMUSG00000031672 |
| 11 | 17336829 | NA | Lsm2 | LSM2 homolog, U6 small nuclear RNA associated (S. cerevisiae) | 27756 | ENSMUSG00000007050 |
| 12 | 17288454 | NA | Irx4 | Iroquois related homeobox 4 (Drosophila) | 50916 | ENSMUSG00000021604 |
| 13 | 17467996 | NA | Mrpl19 | mitochondrial ribosomal protein L19 | 56284 | ENSMUSG00000030045 |
| 14 | 17306758 | NA | Tm9sf1 | transmembrane 9 superfamily member 1 | 74140 | ENSMUSG00000002320 |
| 15 | 17504712 | NA | Exoc3l | exocyst complex component 3-like | 277978 | ENSMUSG00000043251 |
| 16 | 17321467 | NA | Tuba1b | tubulin, alpha 1B | 22143 | ENSMUSG00000023004 |
| 17 | 17541597 | NA | Frmd7 | FERM domain containing 7 | 385354 | ENSMUSG00000036131 |
| 18 | 17457603 | NA | Tas2r108 | taste receptor, type 2, member 108 | 57253 | ENSMUSG00000037140 |
| 19 | 17379187 | NA | Ift52 | intraflagellar transport 52 | 245866 | ENSMUSG00000017858 |
| 20 | 17246284 | NA | Suox | sulfite oxidase | 211389 | ENSMUSG00000049858 |
| 21 | 17437611 | NA | Pgm1 | phosphoglucomutase 1 | 66681 | ENSMUSG00000029171 |
| 22 | 17287579 | NA | Zfp346 | zinc finger protein 346 | 26919 | ENSMUSG00000021481 |
| 23 | 17224577 | NA | Resp18 | regulated endocrine-specific protein 18 | 19711 | ENSMUSG00000033061 |
| 24 | 17297750 | NA | Ppif | peptidylprolyl isomerase F (cyclophilin F) | 105675 | ENSMUSG00000021868 |
| 25 | 17520177 | NA | Mthfs | 5, 10-methenyltetrahydrofolate synthetase | 107885 | ENSMUSG00000066442 |
| 26 | 17480102 | NA | Sytl2 | synaptotagmin-like 2 | 83671 | ENSMUSG00000030616 |
| 27 | 17521143 | NA | Wdr82 | WD repeat domain containing 82 | 77305 | ENSMUSG00000020257 |
| 28 | 17312944 | NA | Polr2f | polymerase (RNA) II (DNA directed) polypeptide F | 69833 | ENSMUSG00000033020 |
| 29 | 17312939 | NA | Polr2f | polymerase (RNA) II (DNA directed) polypeptide F | 69833 | ENSMUSG00000033020 |
| 30 | 17312941 | NA | Polr2f | polymerase (RNA) II (DNA directed) polypeptide F | 69833 | ENSMUSG00000033020 |
| 31 | 17329151 | NA | Alg3 | asparagine-linked glycosylation 3 (alpha-1,3-mannosyltransferase) | 208624 | ENSMUSG00000033809 |
| 32 | 17500391 | NA | Rnf122 | ring finger protein 122 | 68867 | ENSMUSG00000039328 |
| 33 | 17400222 | NA | Vps72 | vacuolar protein sorting 72 (yeast) | 21427 | ENSMUSG00000008958 |
| 34 | 17499396 | NA | Fbxo25 | F-box protein 25 | 66822 | ENSMUSG00000038365 |
| 35 | 17324664 | NA | Dlg1 | discs, large homolog 1 (Drosophila) | 13383 | ENSMUSG00000022770 |
| 36 | 17396369 | NA | Nceh1 | arylacetamide deacetylase-like 1 | 320024 | ENSMUSG00000027698 |
| 37 | 17314556 | NA | Slc48a1 | solute carrier family 48 (heme transporter), member 1 | 67739 | ENSMUSG00000081534 |
| 38 | 17246209 | NA | Rpl41 | ribosomal protein L41 | 67945 | ENSMUSG00000093674 |
| 39 | 17529231 | NA | Phip | pleckstrin homology domain interacting protein | 83946 | ENSMUSG00000032253 |
| 40 | 17483220 | NA | Cdipt | CDP-diacylglycerol--inositol 3-phosphatidyltransferase (phosphatidylinositol synthase) | 52858 | ENSMUSG00000030682 |
| 41 | 17361975 | NA | Snx15 | sorting nexin 15 | 69024 | ENSMUSG00000024787 |
| 42 | 17424319 | NA | Sigmar1 | sigma non-opioid intracellular receptor 1 | 18391 | ENSMUSG00000036078 |
| 43 | 17260221 | NA | Pold2 | polymerase (DNA directed), delta 2, regulatory subunit | 18972 | ENSMUSG00000020471 |
| 44 | 17446580 | NA | Shh | sonic hedgehog | 20423 | ENSMUSG00000002633 |
| 45 | 17281971 | NA | Sgpp1 | sphingosine-1-phosphate phosphatase 1 | 81535 | ENSMUSG00000021054 |
| 46 | 17238549 | NA | Wibg | within bgcn homolog (Drosophila) | 78428 | ENSMUSG00000064030 |
| 47 | 17212355 | NA | Nck2 | non-catalytic region of tyrosine kinase adaptor protein 2 | 17974 | ENSMUSG00000066877 |
| 48 | 17307695 | NA | Msra | methionine sulfoxide reductase A | 110265 | ENSMUSG00000054733 |
| 49 | 17303496 | NA | Fezf2 | Fez family zinc finger 2 | 54713 | ENSMUSG00000021743 |
| 50 | 17345519 | NA | Rrp36 | ribosomal RNA processing 36 homolog (S. cerevisiae) | 224823 | ENSMUSG00000023971 |
| 51 | 17211335 | NA | Tfap2d | transcription factor AP-2, delta | 226896 | ENSMUSG00000042596 |
| 52 | 17506631 | NA | Tubb3 | tubulin, beta 3 class III | 22152 | ENSMUSG00000062380 |
| 53 | 17235584 | NA | Dapk3 | death-associated protein kinase 3 | 13144 | ENSMUSG00000034974 |
| 54 | 17338670 | NA | Fsd1 | fibronectin type 3 and SPRY domain-containing protein | 240121 | ENSMUSG00000011589 |
| 55 | 17300411 | NA | Thtpa | thiamine triphosphatase | 105663 | ENSMUSG00000045691 |
| 56 | 17460879 | NA | Hdac11 | histone deacetylase 11 | 232232 | ENSMUSG00000034245 |
| 57 | 17444100 | NA | Chst12 | carbohydrate sulfotransferase 12 | 59031 | ENSMUSG00000036599 |
| 58 | 17359994 | NA | Fbxl15 | F-box and leucine-rich repeat protein 15 | 68431 | ENSMUSG00000025226 |
| 59 | 17217666 | NA | Tmem9 | transmembrane protein 9 | 66241 | ENSMUSG00000026411 |
| 60 | 17484068 | NA | Lhpp | phospholysine phosphohistidine inorganic pyrophosphate phosphatase | 76429 | ENSMUSG00000030946 |
| 61 | 17497366 | NA | Ebf3 | early B cell factor 3 | 13593 | ENSMUSG00000010476 |
| 62 | 17288616 | NA | Tppp | tubulin polymerization promoting protein | 72948 | ENSMUSG00000021573 |
| 63 | 17446322 | NA | Prkag2 | protein kinase, AMP-activated, gamma 2 non-catalytic subunit | 108099 | ENSMUSG00000028944 |
| 64 | 17452552 | NA | Rhof | ras homolog gene family, member f | 23912 | ENSMUSG00000029449 |
| 65 | 17407764 | NA | Prune | prune homolog (Drosophila) | 229589 | ENSMUSG00000015711 |
| 66 | 17524523 | NA | Eif3g | eukaryotic translation initiation factor 3, subunit G | 53356 | ENSMUSG00000070319 |
| 67 | 17268995 | NA | Krt222 | keratin 222 | 268481 | ENSMUSG00000035849 |
| 68 | 17505367 | NA | Txnl4b | thioredoxin-like 4B | 234723 | ENSMUSG00000031723 |
| 69 | 17540982 | NA | Sept6 | septin 6 | 56526 | ENSMUSG00000050379 |
| 70 | 17235268 | NA | Ndufs7 | NADH dehydrogenase (ubiquinone) Fe-S protein 7 | 75406 | ENSMUSG00000020153 |
| 71 | 17447726 | NA | Hs3st1 | heparan sulfate (glucosamine) 3-O-sulfotransferase 1 | 15476 | ENSMUSG00000051022 |
| 72 | 17454416 | NA | Zfand2a | zinc finger, AN1-type domain 2A | 100494 | ENSMUSG00000053581 |
| 73 | 17422117 | NA | Acot7 | acyl-CoA thioesterase 7 | 70025 | ENSMUSG00000028937 |
| 74 | 17536264 | NA | Pcyt1b | phosphate cytidylyltransferase 1, choline, beta isoform | 236899 | ENSMUSG00000035246 |
| 75 | 17357213 | NA | Zbtb3 | zinc finger and BTB domain containing 3 | 75291 | ENSMUSG00000071661 |
| 76 | 17453106 | NA | Zfp11 | zinc finger protein 11 | 22648 | ENSMUSG00000051034 |
| 77 | 17335357 | NA | Lhfpl5 | lipoma HMGIC fusion partner-like 5 | 328789 | ENSMUSG00000062252 |
| 78 | 17497626 | NA | Sprn | shadow of prion protein | 212518 | ENSMUSG00000045733 |
| 79 | 17315558 | NA | Copz1 | coatomer protein complex, subunit zeta 1 | 56447 | ENSMUSG00000060992 |
| 80 | 17361988 | NA | Arl2 | ADP-ribosylation factor-like 2 | 56327 | ENSMUSG00000024944 |
| 81 | 17502583 | NA | Mcm5 | minichromosome maintenance deficient 5, cell division cycle 46 (S. cerevisiae) | 17218 | ENSMUSG00000005410 |
| 82 | 17485815 | NA | Syt5 | synaptotagmin V | 53420 | ENSMUSG00000004961 |
| 83 | 17519718 | NA | Mto1 | mitochondrial translation optimization 1 homolog (S. cerevisiae) | 68291 | ENSMUSG00000032342 |
| 84 | 17488134 | NA | Rab4b | RAB4B, member RAS oncogene family | 19342 | ENSMUSG00000053291 |
| 85 | 17344453 | NA | Ppp1r18 | protein phosphatase 1, regulatory subunit 18 | 76448 | ENSMUSG00000034595 |
| 86 | 17317208 | NA | Derl1 | Der1-like domain family, member 1 | 67819 | ENSMUSG00000022365 |
| 87 | 17501544 | NA | Npy1r | neuropeptide Y receptor Y1 | 18166 | ENSMUSG00000036437 |
| 88 | 17428858 | NA | Rnf220 | ring finger protein 220 | 66743 | ENSMUSG00000028677 |
| 89 | 17428857 | NA | Rnf220 | ring finger protein 220 | 66743 | ENSMUSG00000028677 |
| 90 | 17408684 | NA | Dclre1b | DNA cross-link repair 1B, PSO2 homolog (S. cerevisiae) | 140917 | ENSMUSG00000027845 |
| 91 | 17443047 | NA | Caln1 | calneuron 1 | 140904 | ENSMUSG00000060371 |
| 92 | 17436077 | NA | Nrbp1 | nuclear receptor binding protein 1 | 192292 | ENSMUSG00000029148 |
| 93 | 17252170 | NA | Rnf167 | ring finger protein 167 | 70510 | ENSMUSG00000040746 |
| 94 | 17543988 | NA | Taf9b | TAF9B RNA polymerase II, TATA box binding protein (TBP)-associated factor | 407786 | ENSMUSG00000047242 |
| 95 | 17459676 | NA | Retsat | retinol saturase (all trans retinol 13,14 reductase) | 67442 | ENSMUSG00000056666 |
| 96 | 17337269 | NA | Nrm | nurim (nuclear envelope membrane protein) | 106582 | ENSMUSG00000059791 |
| 97 | 17488463 | NA | Med29 | mediator complex subunit 29 | 67224 | ENSMUSG00000003444 |
| 98 | 17391270 | NA | Kcnip3 | Kv channel interacting protein 3, calsenilin | 56461 | ENSMUSG00000079056 |
| 99 | 17324576 | NA | Hrasls | HRAS-like suppressor | 27281 | ENSMUSG00000022525 |
| 100 | 17412579 | NA | Gabrr1 | gamma-aminobutyric acid (GABA) C receptor, subunit rho 1 | 14408 | ENSMUSG00000028280 |
| 101 | 17348933 | NA | Mapre2 | microtubule-associated protein, RP/EB family, member 2 | 212307 | ENSMUSG00000024277 |
| 102 | 17266489 | NA | Tmem97 | transmembrane protein 97 | 69071 | ENSMUSG00000037278 |
| 103 | 17299353 | NA | Ktn1 | kinectin 1 | 16709 | ENSMUSG00000021843 |
| 104 | 17506854 | NA | Tsnax | translin-associated factor X | 53424 | ENSMUSG00000056820 |
| 105 | 17413573 | NA | Grhpr | glyoxylate reductase/hydroxypyruvate reductase | 76238 | ENSMUSG00000035637 |
| 106 | 17288716 | NA | Glrx | glutaredoxin | 93692 | ENSMUSG00000021591 |
| 107 | 17429495 | NA | Nfyc | nuclear transcription factor-Y gamma | 18046 | ENSMUSG00000032897 |
| 108 | 17361855 | NA | Pola2 | polymerase (DNA directed), alpha 2 | 18969 | ENSMUSG00000024833 |
| 109 | 17527532 | NA | Mpi | mannose phosphate isomerase | 110119 | ENSMUSG00000032306 |
| 110 | 17512463 | NA | Atp6v0d1 | ATPase, H+ transporting, lysosomal V0 subunit D1 | 11972 | ENSMUSG00000013160 |
| 111 | 17512466 | NA | Atp6v0d1 | ATPase, H+ transporting, lysosomal V0 subunit D1 | 11972 | ENSMUSG00000013160 |
| 112 | 17252635 | NA | Shpk | sedoheptulokinase | 74637 | ENSMUSG00000005951 |
| 113 | 17313008 | NA | Cby1 | chibby homolog 1 (Drosophila) | 73739 | ENSMUSG00000022428 |
| 114 | 17535434 | NA | Nsdhl | NAD(P) dependent steroid dehydrogenase-like | 18194 | ENSMUSG00000031349 |
| 115 | 17503023 | NA | Asf1b | ASF1 anti-silencing function 1 homolog B (S. cerevisiae) | 66929 | ENSMUSG00000005470 |
| 116 | 17328829 | NA | Slc25a1 | solute carrier family 25 (mitochondrial carrier, citrate transporter), member 1 | 13358 | ENSMUSG00000003528 |
| 117 | 17454256 | NA | Taf6 | TAF6 RNA polymerase II, TATA box binding protein (TBP)-associated factor | 21343 | ENSMUSG00000036980 |
| 118 | 17432967 | NA | Ubiad1 | UbiA prenyltransferase domain containing 1 | 71707 | ENSMUSG00000047719 |
| 119 | 17527666 | NA | Islr2 | immunoglobulin superfamily containing leucine-rich repeat 2 | 320563 | ENSMUSG00000051243 |
| 120 | 17279499 | NA | Crip2 | cysteine rich protein 2 | 68337 | ENSMUSG00000006356 |
| 121 | 17542220 | NA | Gabra3 | gamma-aminobutyric acid (GABA) A receptor, subunit alpha 3 | 14396 | ENSMUSG00000031343 |
| 122 | 17231118 | NA | Rcor3 | REST corepressor 3 | 214742 | ENSMUSG00000037395 |
| 123 | 17379554 | NA | Zswim1 | zinc finger, SWIM domain containing 1 | 71971 | ENSMUSG00000017764 |
| 124 | 17298407 | NA | Bap1 | Brca1 associated protein 1 | 104416 | ENSMUSG00000021901 |
| 125 | 17280897 | NA | Stxbp6 | syntaxin binding protein 6 (amisyn) | 217517 | ENSMUSG00000046314 |
| 126 | 17451443 | NA | Coro1c | coronin, actin binding protein 1C | 23790 | ENSMUSG00000004530 |
| 127 | 17273280 | NA | Stra13 | stimulated by retinoic acid 13 | 20892 | ENSMUSG00000025144 |
| 128 | 17301342 | NA | Ints9 | integrator complex subunit 9 | 210925 | ENSMUSG00000021975 |
| 129 | 17393357 | NA | Eif6 | eukaryotic translation initiation factor 6 | 16418 | ENSMUSG00000027613 |
| 130 | 17412593 | NA | Srsf12 | serine/arginine-rich splicing factor 12 | 272009 | ENSMUSG00000054679 |
| 131 | 17451816 | NA | Hspb8 | heat shock protein 8 | 80888 | ENSMUSG00000041548 |
| 132 | 17488544 | NA | Nfkbib | nuclear factor of kappa light polypeptide gene enhancer in B cells inhibitor, beta | 18036 | ENSMUSG00000030595 |
| 133 | 17504160 | NA | Polr2c | polymerase (RNA) II (DNA directed) polypeptide C | 20021 | ENSMUSG00000031783 |
| 134 | 17455093 | NA | Zkscan14 | zinc finger with KRAB and SCAN domains 14 | 67235 | ENSMUSG00000029627 |
| 135 | 17328810 | NA | Dgcr14 | DiGeorge syndrome critical region gene 14 | 27886 | ENSMUSG00000003527 |
| 136 | 17229466 | NA | Hsd17b7 | hydroxysteroid (17-beta) dehydrogenase 7 | 15490 | ENSMUSG00000026675 |
| 137 | 17242318 | NA | Gm7138 | predicted gene 7138 | 634517 | ENSMUSG00000095593 |
| 138 | 17463150 | NA | Vamp1 | vesicle-associated membrane protein 1 | 22317 | ENSMUSG00000030337 |
| 139 | 17306861 | NA | Dhrs1 | dehydrogenase/reductase (SDR family) member 1 | 52585 | ENSMUSG00000002332 |
| 140 | 17306864 | NA | Dhrs1 | dehydrogenase/reductase (SDR family) member 1 | 52585 | ENSMUSG00000002332 |
| 141 | 17306860 | NA | Dhrs1 | dehydrogenase/reductase (SDR family) member 1 | 52585 | ENSMUSG00000002332 |
| 142 | 17306856 | NA | Dhrs1 | dehydrogenase/reductase (SDR family) member 1 | 52585 | ENSMUSG00000002332 |
| 143 | 17306865 | NA | Dhrs1 | dehydrogenase/reductase (SDR family) member 1 | 52585 | ENSMUSG00000002332 |
| 144 | 17305182 | NA | Nrg3 | neuregulin 3 | 18183 | ENSMUSG00000041014 |
| 145 | 17418571 | NA | Trappc3 | trafficking protein particle complex 3 | 27096 | ENSMUSG00000028847 |
| 146 | 17318942 | NA | Pvalb | parvalbumin | 19293 | ENSMUSG00000005716 |
| 147 | 17419206 | NA | Pef1 | penta-EF hand domain containing 1 | 67898 | ENSMUSG00000028779 |
| 148 | 17535752 | NA | Emd | emerin | 13726 | ENSMUSG00000001964 |
| 149 | 17396024 | NA | Stmn2 | stathmin-like 2 | 20257 | ENSMUSG00000027500 |
| 150 | 17338043 | NA | Yipf3 | Yip1 domain family, member 3 | 28064 | ENSMUSG00000071074 |
| 151 | 17313199 | NA | Adsl | adenylosuccinate lyase | 11564 | ENSMUSG00000022407 |
| 152 | 17418447 | NA | Meaf6 | MYST/Esa1-associated factor 6 | 70088 | ENSMUSG00000028863 |
| 153 | 17232215 | NA | Moxd1 | monooxygenase, DBH-like 1 | 59012 | ENSMUSG00000020000 |
| 154 | 17356202 | NA | Pold4 | polymerase (DNA-directed), delta 4 | 69745 | ENSMUSG00000024854 |
| 155 | 17240123 | NA | Clvs2 | clavesin 2 | 215890 | ENSMUSG00000019785 |
| 156 | 17462975 | NA | Mlf2 | myeloid leukemia factor 2 | 30853 | ENSMUSG00000030120 |
| 157 | 17255719 | NA | Mrpl10 | mitochondrial ribosomal protein L10 | 107732 | ENSMUSG00000001445 |
| 158 | 17257962 | NA | Sstr2 | somatostatin receptor 2 | 20606 | ENSMUSG00000047904 |
| 159 | 17344794 | NA | Znrd1 | zinc ribbon domain containing, 1 | 66136 | ENSMUSG00000036315 |
| 160 | 17232843 | NA | Zbtb24 | zinc finger and BTB domain containing 24 | 268294 | ENSMUSG00000019826 |
| 161 | 17300261 | NA | Oxa1l | oxidase assembly 1-like | 69089 | ENSMUSG00000000959 |
| 162 | 17224540 | NA | Tuba4a | tubulin, alpha 4A | 22145 | ENSMUSG00000026202 |
| 163 | 17456308 | NA | Kcnd2 | potassium voltage-gated channel, Shal-related family, member 2 | 16508 | ENSMUSG00000060882 |
| 164 | 17517723 | NA | Rpp25 | ribonuclease P 25 subunit (human) | 102614 | ENSMUSG00000062309 |
| 165 | 17307134 | NA | Cryl1 | crystallin, lambda 1 | 68631 | ENSMUSG00000021947 |
| 166 | 17521652 | NA | Nicn1 | nicolin 1 | 66257 | ENSMUSG00000032606 |
| 167 | 17369862 | NA | Dpm2 | dolichol-phosphate (beta-D) mannosyltransferase 2 | 13481 | ENSMUSG00000026810 |
| 168 | 17322559 | NA | Hmox2 | heme oxygenase (decycling) 2 | 15369 | ENSMUSG00000004070 |
| 169 | 17537677 | NA | Drp2 | dystrophin related protein 2 | 13497 | ENSMUSG00000000223 |
| 170 | 17375833 | NA | Tmem127 | transmembrane protein 127 | 69470 | ENSMUSG00000034850 |
| 171 | 17457310 | NA | Chrm2 | cholinergic receptor, muscarinic 2, cardiac | 243764 | ENSMUSG00000045613 |
| 172 | 17538096 | NA | Rnf128 | ring finger protein 128 | 66889 | ENSMUSG00000031438 |
| 173 | 17288160 | NA | Cdk20 | cyclin-dependent kinase 20 | 105278 | ENSMUSG00000021483 |
[truncated: 53,209 more chars]
