# Supplementary figures and images for: Genetic Networks in Mouse Retinal Ganglion Cells
Source: Front Genet. 2016 Sep 28;7:169. doi: 10.3389/fgene.2016.00169 (PMC5039302; doi:10.3389/fgene.2016.00169)

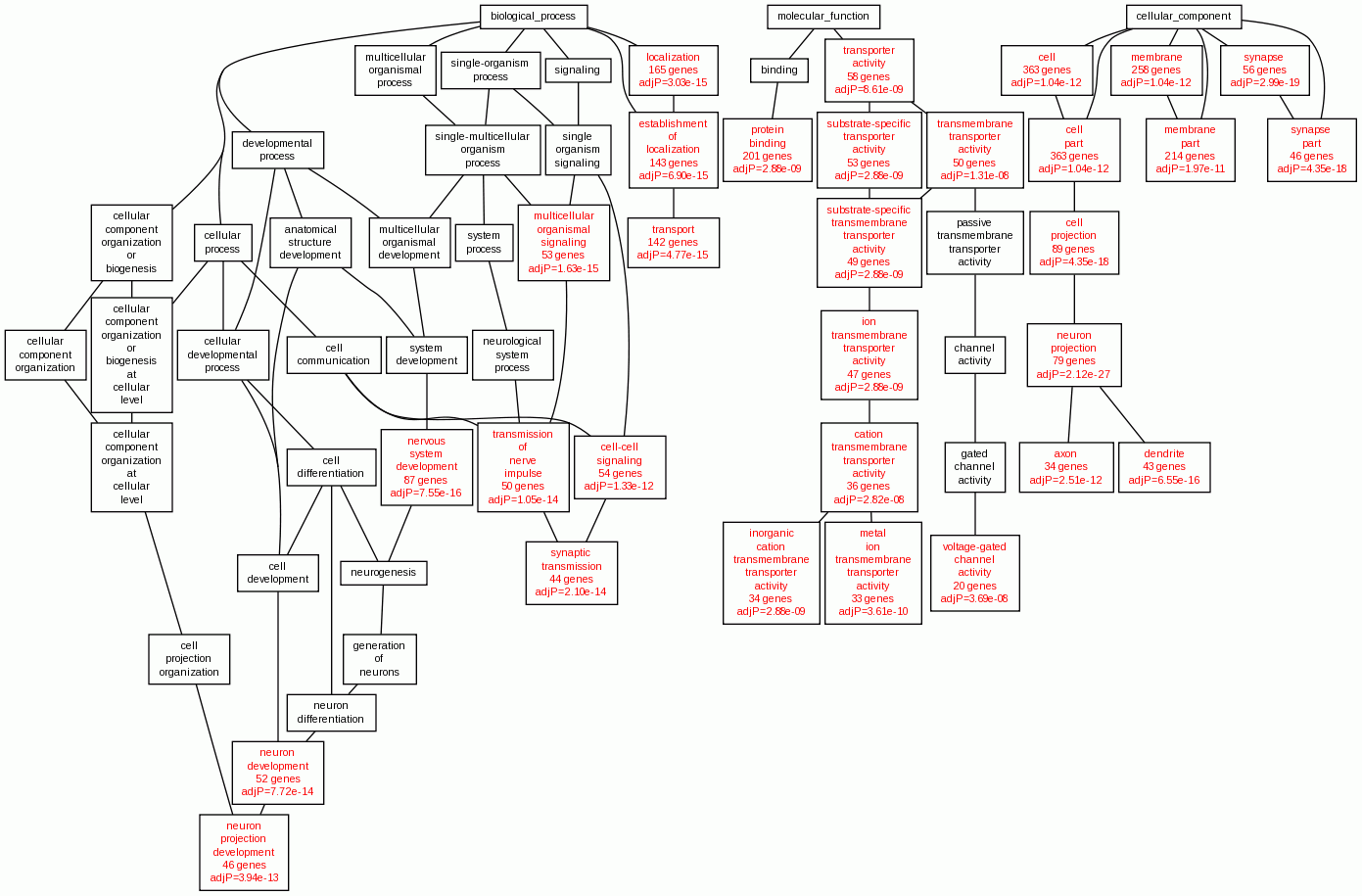

Supplement: Supplementary file 3 [file DataSheet1.ZIP › Thy1-network GO/files/final_DAG_file_1470844800.gif]

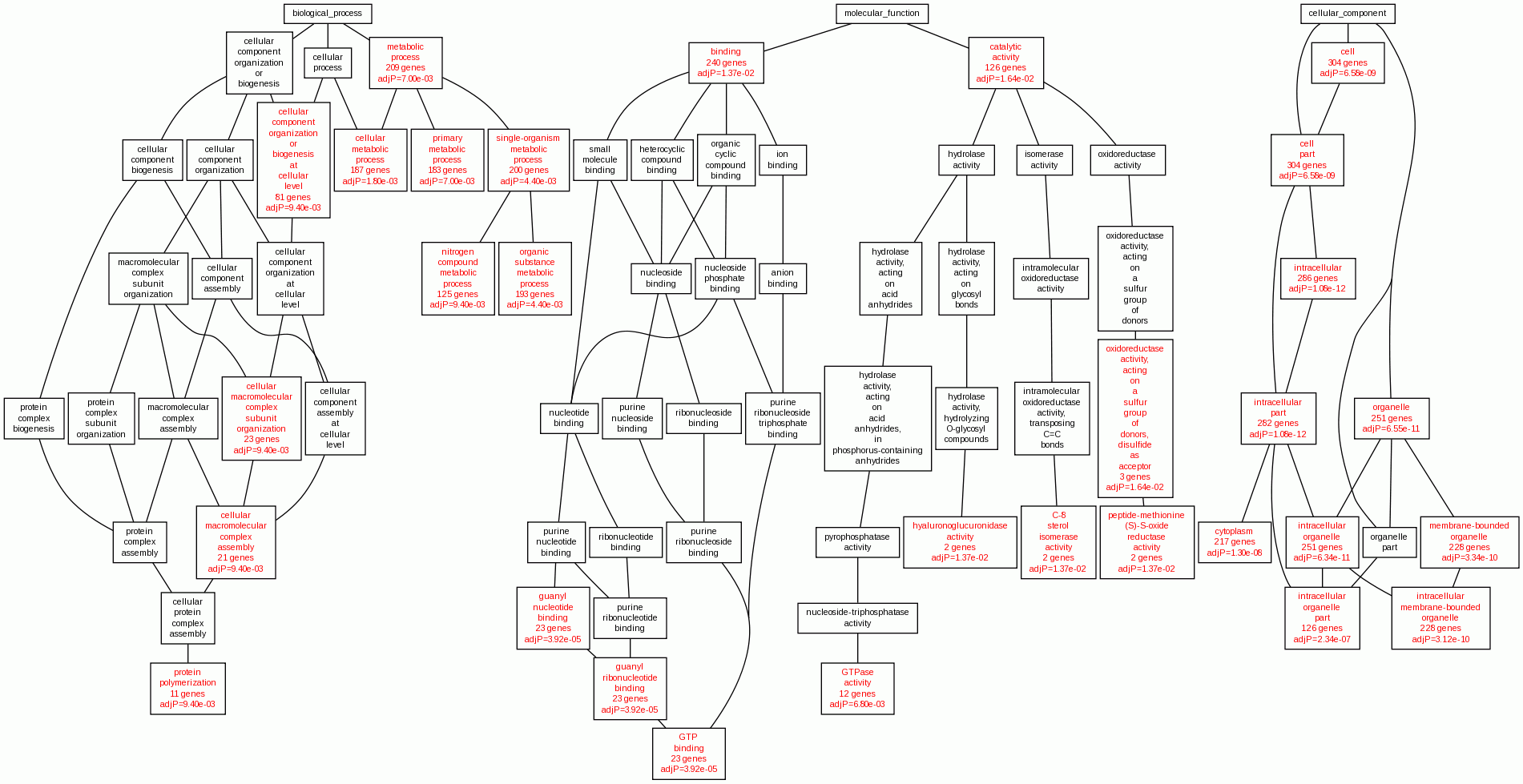

Supplement: Supplementary file 3 [file DataSheet1.ZIP › Tubb3-network GO/files/final_DAG_file_1470845142.gif]

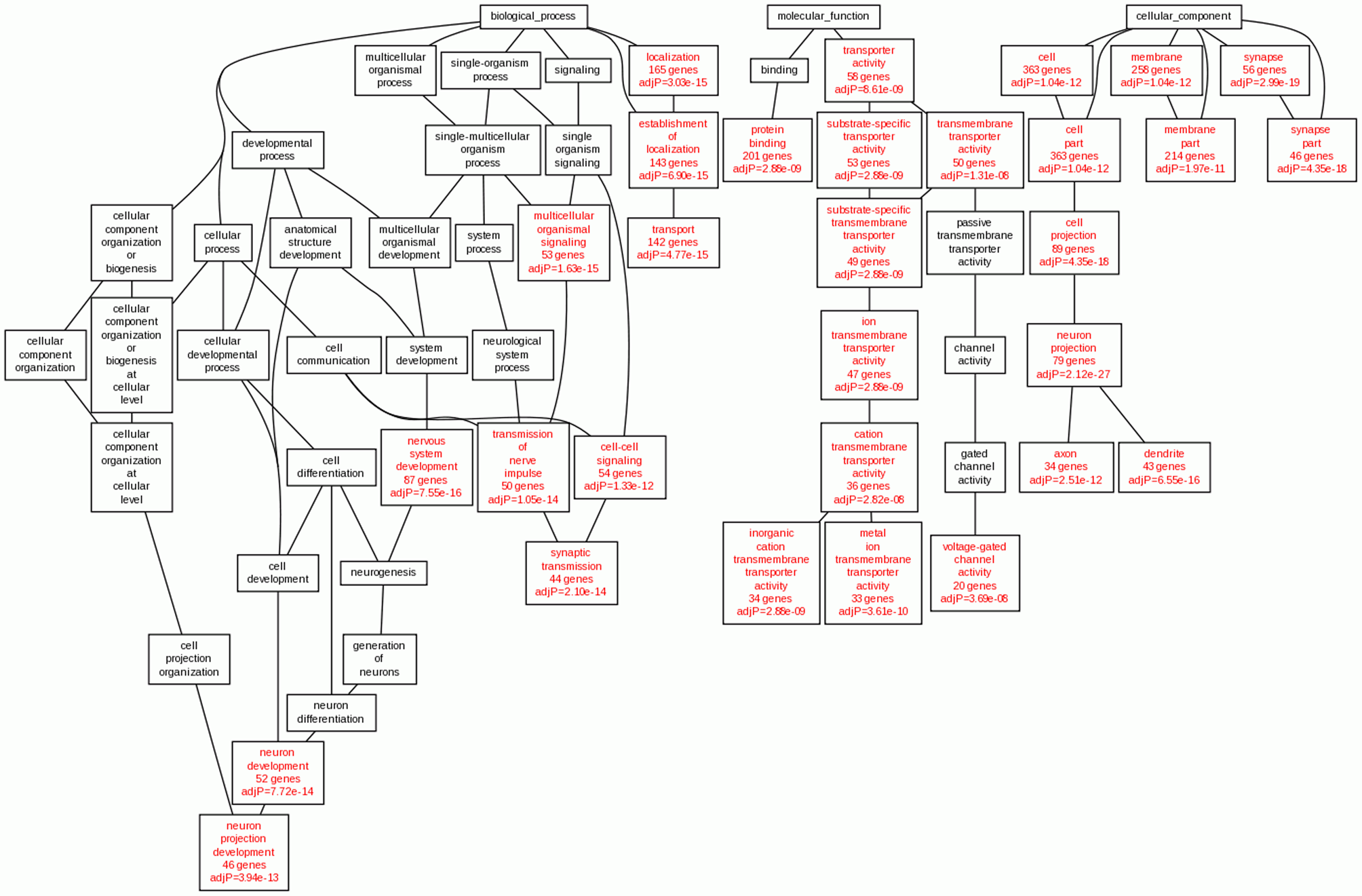

Supplement: Supplementary file 4 [file Image1.TIFF]

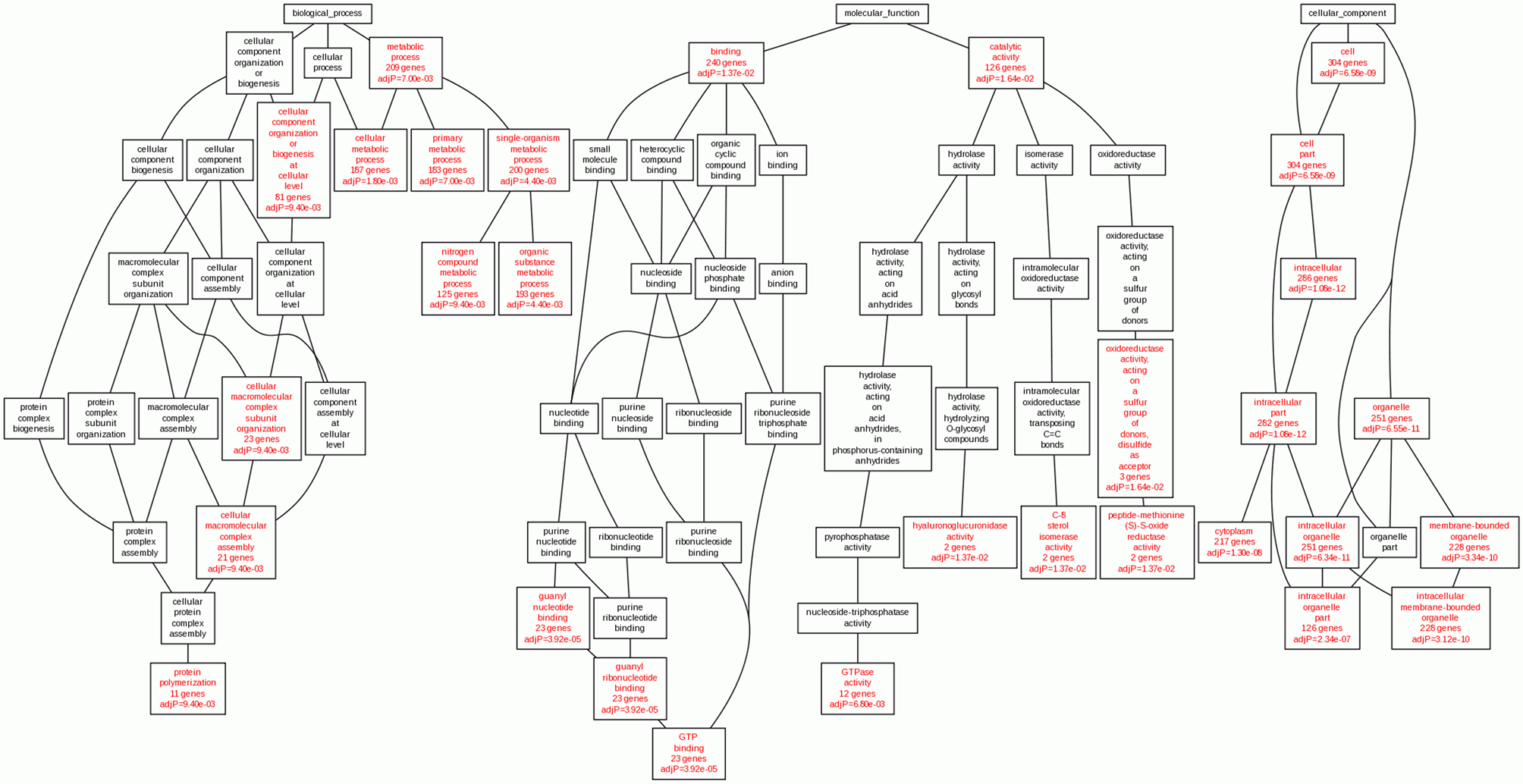

Supplement: Supplementary file 5 [file Image2.TIFF]
